# Stereospecific Transition‐Metal‐Free Alkylation of Chiral Non‐Racemic Secondary Tosylates with Cyanohydrins: Convenient Access to Enantiomerically Enriched α‐Tertiary Ketones

**DOI:** 10.1002/anie.202520674

**Published:** 2026-01-13

**Authors:** Jinjin Ma, Hui Li, Jadab Majhi, P. Andrew Evans

**Affiliations:** ^1^ Department of Chemistry Queen's University 90 Bader Lane Kingston K7L 3N6 Canada; ^2^ College of Chemistry & Pharmacy Northwest A&F University Yangling Shaanxi 712100 P. R. of China; ^3^ Xiangya School of Pharmaceutical Sciences Central South University Changsha Hunan 410013 P. R. of China

**Keywords:** Cyanohydrins, Enantioenriched α‐tertiary ketones, S_N_2, Stereospecific alkylation, Transition‐metal‐free

## Abstract

A novel stereospecific, transition‐metal‐free alkylation of cyanohydrins with enantiomerically enriched secondary tosylates is described. This method enables the general, efficient, and operationally simple synthesis of enantioenriched α‐tertiary ketones by leveraging the nucleophilicity of cyanohydrin anions, which serve as umpolung carbonyl equivalents to facilitate highly stereospecific S_N_2 substitutions. Reactions involving alkenyl cyanohydrins proceed through a stereoconvergent olefin synthesis (SCOS) from a geometric *E/Z* mixture, furnishing stereochemically defined, conjugated α‐tertiary ketones. This methodology exhibits broad functional group tolerance, accommodating unactivated and sterically hindered acyclic and cyclic secondary tosylates. The resulting chiral ketones are configurationally stable and can be readily converted into synthetically versatile intermediates using classical transformations. The utility of this approach is exemplified by a concise, stereospecific synthesis of (*R*)‐cyclamen aldehyde, underscoring its potential to streamline the preparation of enantioenriched α‐tertiary ketones—structural motifs that feature prominently in both natural and non‐natural products and serve as key intermediates in target‐directed synthesis.

## Graphical abstract

A stereospecific, *transition‐metal‐free S_N_2 alkylation* of cyanohydrins with chiral non‐racemic secondary tosylates is reported, providing efficient access to enantioenriched α‐tertiary ketones. The method exhibits broad substrate scope, operational simplicity, and excellent stereospecificity, including stereoconvergent reactions of *E/Z* mixtures of alkenyl cyanohydrins. The utility of the strategy is demonstrated through a concise synthesis of (*R*)‐cyclamen aldehyde.

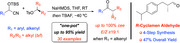

Enantioenriched α‐tertiary ketones are key structural motifs in natural products, pharmaceuticals, and agrochemicals, and serve as versatile intermediates for constructing stereochemically complex molecules (Scheme 1a).^[^ ^1^, ^2^, ^3^, ^4^, ^5^ ^]^ Despite significant advances in enantioselective enolate alkylation, the stereoselective synthesis of *acyclic* α‐tertiary ketones—particularly those bearing unactivated alkyl substituents—remains a formidable challenge (Scheme 1b).^[^ ^6^, ^7^, ^8^, ^9^, ^10^ ^]^ A central problem is the propensity to racemize under both acidic and basic conditions, complicating the preservation of enantiopurity. Classical strategies also suffer from poor regioselectivity during the enolization of unsymmetrical ketones and often require strict control of enolate geometry to achieve stereoselectivity. Competing side reactions, including aldol condensation, *O*‐alkylation, and polyalkylation, further limit chemoselectivity. As a result, enolate alkylation remains largely restricted to relatively small and activated electrophiles.^[^ ^11^ ^]^ Stereospecific substitution of enantioenriched alkyl electrophiles offers a conceptually distinct and increasingly powerful strategy for accessing chiral α‐tertiary ketones. These methods invert pre‐existing stereocenters with high stereochemical fidelity, while avoiding the intrinsic limitations of enolate intermediates.^[^ ^12^, ^13^, ^14^, ^15^, ^16^, ^17^, ^18^ ^]^ However, direct stereospecific alkylation of nucleophilic acyl anions remains largely unexplored (Scheme 1c). To date, only two such examples have been reported, both involving the formal coupling of two electrophilic partners. Collman^[^ ^12^ ^]^ and Alexanian^[^ ^13^ ^]^ independently described iron‐mediated and cobalt‐catalyzed carbonyl insertions with enantioenriched tosylates followed by alkylation of the resulting acyl metal intermediates with alkyl halides and butadienes, respectively (Scheme 1c). These transformations require transition metal complexes in combination with additional reagents, such as carbon monoxide, HMPA, and methyl iodide, among others. Moreover, the scope is limited to linear alkyl groups (R_2_/R_3_), restricting access to more hindered enantioenriched α‐tertiary ketone derivatives. Despite these limitations, stereospecific substitution offers a powerful alternative to enolate chemistry, providing a broader substrate scope and greater functional group compatibility under mild reaction conditions.

**Scheme 1:**
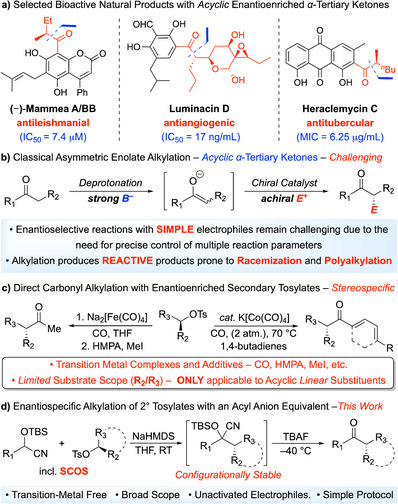
Conceptual background for the transition‐metal‐free enantiospecific construction of cyclic and acyclic enantioenriched α‐tertiary ketones

Building on the previous demonstration that cyanohydrins serve as privileged nucleophilic acyl equivalents in alkylation and arylation reactions,^[^ ^19^, ^20^ ^]^ including metal‐catalyzed allylic alkylation variants,^[^ ^14^, ^21^, ^22^, ^23^, ^24^, ^25^ ^]^ we envisioned they could be repurposed for stereospecific alkylation. Although a single example of an ethyl vinyl ether–protected cyanohydrin undergoing substitution with an enantioenriched alkyl bromide has been reported, the reaction proceeded in low yield—presumably due to the harsh acidic conditions required for deprotection—and the stereochemical outcome was not determined.^[^ ^26^ ^]^ To overcome these limitations, *tert*‐butyldimethylsilyl‐protected cyanohydrins, which are readily unmasked under mild, fluorine–mediated conditions, were employed as nucleophilic acyl equivalents.^[^ ^20^, ^21^, ^22^, ^23^, ^24^, ^25^ ^]^ Moreover, enantioenriched secondary tosylates derived from readily available chiral epoxides represent attractive chiral‐pool electrophiles; however, their use remains comparatively rare relative to the corresponding halides, despite their excellent leaving‐group ability, because they behave as hard electrophiles in alkylation reactions. We envisioned that cyanohydrins, as borderline hard nucleophiles, would be well‐matched to form a trigonal‐bipyramidal‐like transition state, thereby enabling smooth S_N_2 displacement at these enantioenriched secondary tosylates. This stereoinvertive substitution provides a direct and efficient route to enantioenriched α‐tertiary ketones, thereby redefining the chiral‐pool paradigm for the transition‐metal‐free construction of α‐tertiary carbonyl frameworks.^[^ ^27^, ^28^, ^29^ ^]^ The mild deprotection step provides additional versatility, as the reaction conditions can be tailored to suppress racemization and improve overall efficiency. Herein, we report a transition‐metal‐free, highly enantiospecific alkylation of cyanohydrins with a range of acyclic and cyclic enantioenriched secondary tosylates. Notably, alkenyl cyanohydrins undergo alkylation with concomitant stereoconvergent olefin synthesis (SCOS),^[^ ^25^, ^30^ ^]^ enabling the stereodefined construction of *E*‐alkenyl α‐tertiary ketones from geometric *E/Z* mixtures (Scheme 1d). This work establishes a broadly applicable and mechanistically distinct platform for constructing α‐chiral ketones, underscoring the versatility of cyanohydrins as enantiospecific acylation agents in the synthesis of complex molecules.

In line with our hypothesis, we initially explored the feasibility of an enantiospecific S_N_2 alkylation of cyanohydrins with an enantiomerically enriched secondary tosylate (Table 1). Optimization studies commenced with phenyl cyanohydrin **1a** and (*R*)‐phenylprop‐2‐yl tosylate **2a** ^[^ ^31^ ^]^ as substrates, employing NaHMDS in THF at room temperature. The reaction proceeded efficiently, yielding the desired chiral ketone **3aa** in 87% yield and 100% *cee* (entry 1)^[^ ^32^, ^33^, ^34^, ^35^ ^]^ following in situ deprotection with TBAF at –40 °C. Substitution of NaHMDS with LiHMDS or KHMDS (entries 2 and 3) resulted in slightly lower yields, while maintaining comparable enantiospecificity, suggesting that the counterion's nature influences efficiency but not stereochemical fidelity. Replacing the tosylate with a chiral secondary mesylate (entry 4) afforded a 74% yield and 100% *cee*, confirming the compatibility of alternative leaving groups. Furthermore, using 2‐methyl tetrahydrofuran—a preferred solvent for industrial‐scale synthesis—provided a comparable yield (86%) and enantiopurity (100% *cee*, entry 5), highlighting the potential of this method for preparative‐scale reactions. Studies on temperature variation revealed that lowering or raising the reaction temperature for the alkylation step (entries 6 and 7) yielded consistent results of 75% and 83%, respectively, without any loss in stereospecificity (100% *cee*). However, conducting the TBAF‐mediated deprotection at elevated temperatures (entry 8) significantly reduced the yield to 60%, although enantiospecificity was maintained at 100% *cee*. Hence, optimal deprotection is achieved at –40 °C. To evaluate the robustness of the deprotection step, we substituted TBAF with alternative reagents, such as CsF (entry 9), or concentrated HCl (entry 10); however, these conditions afforded only trace product, and the cyanohydrin intermediate **A** was isolated as a 1:1 mixture of diastereomers. Finally, we examined the influence of the cyanohydrin anion's electronic nature, given its borderline‐hard character. Notably, the *softer* 4‐bromo‐substituted phenyl cyanohydrin **1b** delivered product **3ba** in 81% yield with slightly diminished enantiopurity (entry 11, 88% *cee*), while the *harder* 4‐methoxy derivative afforded **3ca** in 91% yield and 100% *cee* (entry 12). Overall, these studies establish optimal conditions for the stereospecific S_N_2 alkylation of cyanohydrins with enantiomerically enriched secondary tosylates, affording *acyclic* α‐tertiary ketones in excellent yield and stereochemical fidelity under mild, *transition‐metal‐free* conditions.

**Table 1:** Optimization of the enantiospecific alkylation of cyanohydrin **1** with chiral, non‐racemic tosylate (*R*)‐**2a**.^a)^

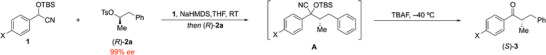							
Entry	Cyanohydrin **1** (X =)		Variations in the optimized conditions	Yield of **3** (%)^b)^		*ee* (%)^c)^	*cee* (%)^c)^
1	H	**a**	*None*	87	**aa**	99	100
2	“	“	LiHMDS *instead of* NaHMDS	78	“	99	100
3	“	“	KHMDS *instead of* NaHMDS	77	“	99	100
4	“	“	MsO *instead of* TsO	74	“	99	100
5	“	“	2‐Me‐THF *instead of* THF	86	“	99	100
6	“	“	0 °C *instead of* RT	75	“	99	100
7	“	“	50 °C *instead of* RT	83	“	99	100
8	“	“	TBAF at –10 °C *instead of* –40 °C	60	“	99	100
9	“	“	CsF^d)^ *instead of* TBAF	Trace^e)^	“	–	–
10	“	“	*conc*. HCl^f)^ *instead of* TBAF	Trace^e)^	“	–	–
11	Br	**b**	*None*	81	**ba**	87	88
***12***	***MeO***	***c***	***None***	***91***	***ca***	***99***	***100***

^a)^ Reactions were performed on a 0.25 mmol scale using 1.5 equiv. of **1a** and 2 equiv. of NaHMDS in THF (2 mL) for *ca*. 10 min., followed by addition of tosylate (*R*)‐**2a**. After 16 h, 4.0 equiv. of TBAF was added at –40 °C followed by stirring for *ca*. 1 h.

^b)^ Yield of isolated product after two steps (one‐pot).

^c)^ The enantiomeric excess (*ee*) was determined by chiral HPLC analysis of the isolated product. The conservation of enantiomeric excess (*cee*) = (*ee* of product/*ee* of starting material) x 100.^[^ ^32^ ^]^

^d)^ 1.0 M Aqueous solution or solid CsF was stirred for *ca*. 24 h.

^e)^ Trace amount of deprotected products was observed based on crude 500 MHz ^1^H‐NMR.

^f)^ 20 equiv. of conc. 12 M HCl was added and the reaction was stirred for *ca*. 24 h.

Table 2 highlights the broad applicability of the optimized conditions (Table 1, entry 12) across a diverse range of enantiomerically enriched secondary tosylates paired with aryl and alkenyl cyanohydrins. The reaction exhibits excellent functional group tolerance, accommodating alkyl‐substituted tosylates without compromising efficiency or enantiospecificity. Substituents such as benzyl, alkyl, allyl, propargyl, and alkoxymethyl groups at R_3_ (Table 2a, entries 1–5) are well tolerated, affording the corresponding α‐tertiary ketones in 75–95% yield and ≥97% *cee* (with R_2_ = Me). To further evaluate the electrophilic partner, the methyl group at R_2_ was replaced with a benzyloxymethyl substituent, while systematically varying the R_3_ group (entries 6–10). This modification introduces additional steric and electronic complexity, providing a more stringent test of the electrophilic component. Remarkably, the transformation proceeded smoothly across a series of α‐ and β‐branched acyclic and cyclic groups, encompassing linear and branched alkyl chains, as well as methallyl, cyclopentylmethyl, and dithianylmethyl substituents, affording products in 82–95% yield and ≥97% *cee*. Notably, even a highly sterically encumbered substrate, typically challenging for S_N_2 reactions, underwent efficient displacement with excellent yield and stereospecificity (entry 7). However, increasing steric demand with a *tert*‐butyl substituent led to no reaction under the optimized conditions (see Supporting Information for unsuccessful substrates). Collectively, these results underscore the robustness and expanded synthetic scope of this enantiospecific alkylation process.

**Table 2:** Scope of the enantiospecific alkylation of aryl cyanohydrins **1** with chiral nonracemic secondary tosylates **2**.^a)^, ^b)^, ^c)^, ^d)^, ^e)^

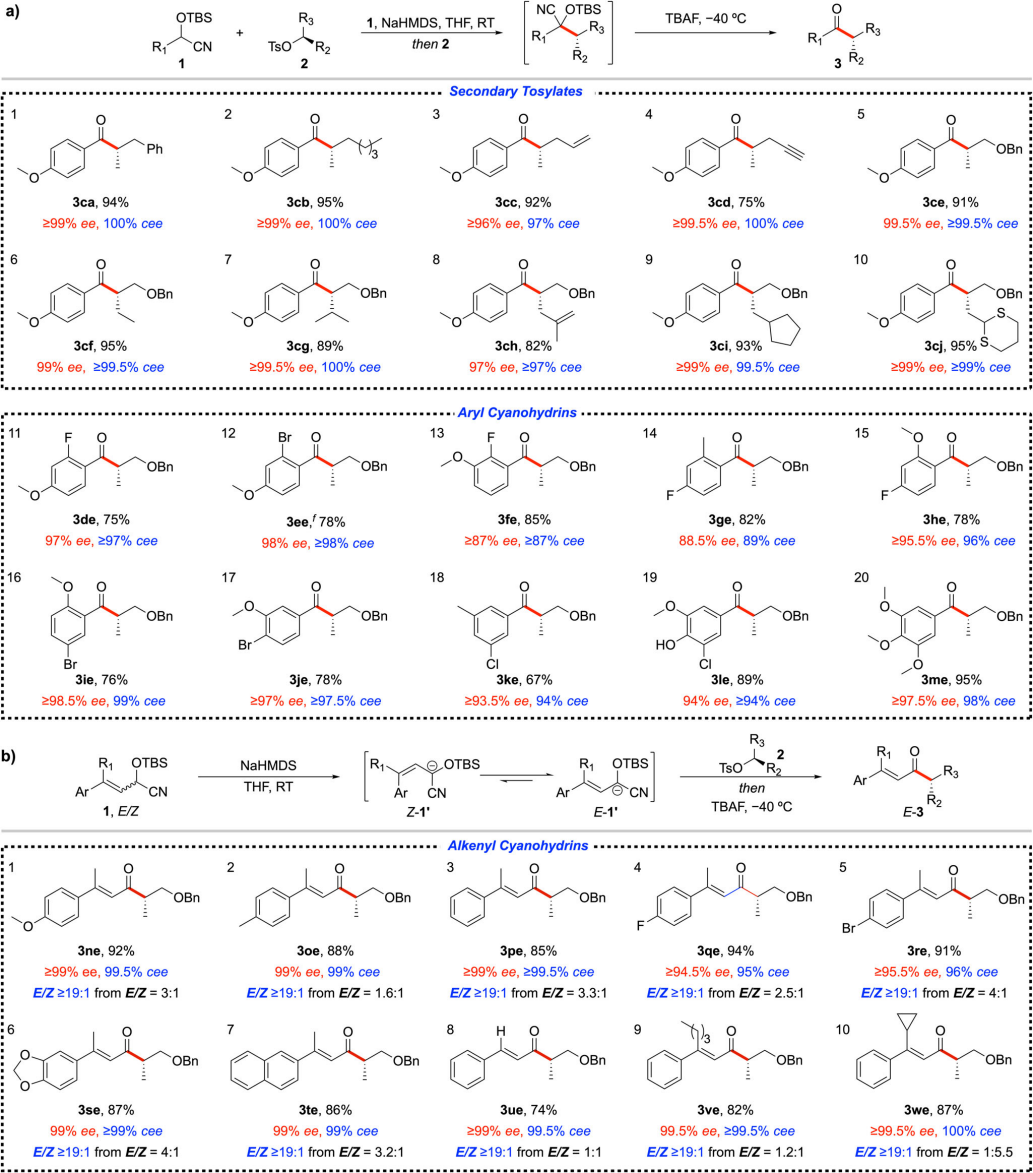

^a)^ All reactions were performed on a 0.25 mmol scale using 1.5 equiv. of cyanohydrin **1** and 2 equiv. NaHMDS in THF (2 mL) for *ca*. 10 min followed by addition of the tosylates **2**. After 16 h, 4.0 equiv. of TBAF was added at –40 °C, followed by stirring for *ca*. 1 h.

^b)^ Yield of isolated product.

^c)^ The enantiomeric excess (*ee*) was determined by chiral HPLC analysis of the isolated product. The conservation of enantiomeric excess (*cee*) = (*ee* of product/*ee* of starting material) x 100.^[^ ^32^ ^]^

^d)^ *E/Z* isomer ratios determined by 500 MHz ^1^H‐NMR.

^e)^ For unsuccessful substrates, see Supporting Information.

^f)^ The reaction was quenched in situ with HCl (2.5 M in Et_2_O, 2 equiv.) at –40 °C before deprotection with TBAF, as the standard procedure yielded 82% yield with only 69% *ee*.

The influence of steric and electronic effects on enantiospecificity was further examined using polysubstituted aryl cyanohydrins (Table 2a, entries 11–20). To specifically probe steric effects, a 4‐methoxy group was held constant while the 2‐substituent was varied. The 2‐fluoro derivative (entry 11) delivered the enantioenriched α‐tertiary ketone in 75% yield and ≥97% *cee*. In contrast, the bulkier 2‐bromo analog (entry 12) gave a comparable yield but significantly reduced stereospecificity (69% *cee*), indicating that increased steric hindrance at the 2‐position promotes racemization during the deprotection. Gratifyingly, in situ quenching with HCl prior to deprotection restored the enantiospecificity to ≥98% *cee*, highlighting the value of this protocol for substrates prone to racemization. Comparison of methoxy positional isomers further clarified electronic effects: shifting the methoxy group from the 4‐ to the 3‐position (entry 13), while retaining the 2‐fluoro substituent, reduced enantiospecificity to ≥87% *cee*, consistent with the stronger *p*‐donating character of the 4‐methoxy group and the higher α‐proton p*K*_a_ of the ketone. Importantly, enantiospecificity can be restored (99% *cee*) with in situ quenching with HCl prior deprotection. To probe the electronic influence of the 2‐substituent in the presence of a 4‐fluoro group, 2‐methyl (entry 14) and 2‐methoxy (entry 15) derivatives were compared. The 2‐methoxy variant afforded 96% *cee*, compared with 89% *cee* for the 2‐methyl analog, indicating that an *ortho*‐electron‐donating group can attenuate the influence of a *para*‐electron‐withdrawing substituent; however, this effect can be circumvented by in situ quenching prior to deprotection (*vide supra*). Collectively, these data reveal a coherent structure‐enantiospecificity relationship: electron‐donating groups at the 4‐position suppress racemization, whereas 2‐substitution can promote racemization during the deprotection step, albeit this effect can be mitigated by in situ quenching. Despite these effects, the method displays broad substitution tolerance, with trisubstituted (entries 16–18) and tetrasubstituted aryl cyanohydrins (entries 19 and 20) affording products in high yield and enantiomeric purity. These findings establish practical guidelines for minimizing racemization and highlight the flexibility of this deprotection protocol, an enabling feature for the stereospecific alkylation of sensitive systems.

Alkenyl cyanohydrins also proved to be competent coupling partners, undergoing stereospecific alkylation in conjunction with a stereoconvergent transformation of the *Z/E*‐alkene (Table 2b). The method is effective for trisubstituted alkenyl cyanohydrins bearing either electron‐donating (entries 1 and 2) or electron‐withdrawing groups (entries 4 and 5) on the aryl ring, affording products in yields of 88–94% with excellent stereoselectivity (*E/Z* ≥19:1) and stereospecificity (95 to ≥99.5% *cee*). Furthermore, polyaryl‐substituted alkenyl cyanohydrins (entries 6 and 7) yielded products with high geometric purity and almost complete stereospecificity (*E/Z* ≥19:1, ≥99% *cee*). Notably, both 1,2‐disubstituted alkenyl cyanohydrins (entry 8) and substrates bearing bulkier branched alkyl groups (entries 9 and 10) underwent smooth transformation, delivering products in 74–87% yield with outstanding stereoselectivity (*E/Z* ≥19:1) and enantiospecificity (≥99.5% *cee*). Overall, these findings establish a general and broadly applicable strategy for accessing stereodefined, enantiomerically enriched *acyclic E*‐alkenyl α‐tertiary ketones—synthetic targets that remain challenging to prepare via traditional carbonyl olefination chemistry.

To further extend the scope, a series of cyclic electrophiles anticipated to be more prone to competing E2 elimination, were evaluated. Remarkably, the reaction furnished the corresponding tetrahydrothiophene, pyrroline, and tetrahydrofuran derivatives in excellent yields (90–93%), highlighting its compatibility with elimination‐prone cyclic systems (Scheme 2a). However, the fixed dihedral angles in these cyclic products render the α‐proton more accessible, thereby enhancing racemization. For example, the tetrahydrothiophene system (entry 1) afforded **3ck** with ≥88% *cee*. In comparison, the pyrroline system gave **3cl** with increased racemization (86% *cee*). Gratifyingly, this problem was effectively addressed using the earlier protocol, which involved prequenching with HCl before TBAF deprotection, thereby restoring enantiospecificity to 99% *cee* (entry 2; see Supporting Information for experimental details). In the case of the tetrahydrofuran derivative, racemization was more pronounced (76% *cee*), necessitating the isolation and purification of the intermediate cyanohydrin before deprotection to afford **3** **cm** with excellent enantiospecificity (entry 3, ≥98.5% *cee*, see Supporting Information for experimental details). These results establish a versatile, metal‐free alkylation platform that accommodates *cyclic* secondary tosylates with high stereochemical fidelity.

**Scheme 2:**
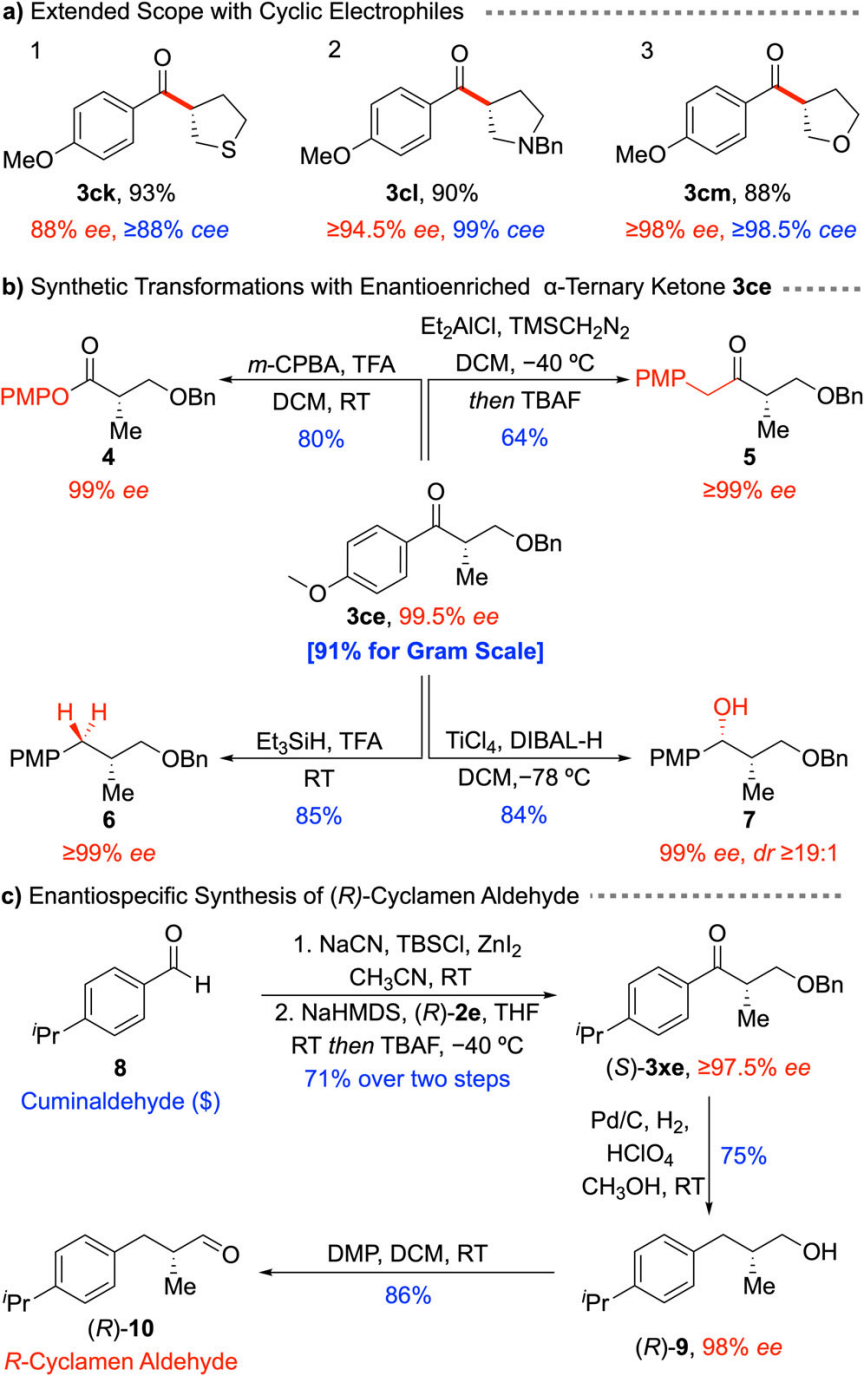
Expanded scope, functional group reactions, and application in synthesis.

To highlight the synthetic potential of this transformation, we explored representative functional group manipulations of the resulting chiral *α*‐tertiary ketone (Scheme 2b). The reaction was successfully scaled up to a gram scale without any loss in efficiency or enantiospecificity, affording ketone **3ce** in 91% yield with ≥99.5% *ee* (Scheme 2b, center). The asymmetric alkylation of prochiral ester enolates with simple electrophiles remains challenging.^[^ ^6^, ^7^ ^]^ Accordingly, we envisioned that converting aryl ketones to the corresponding esters would provide a valuable and complementary strategy. The Baeyer–Villiger oxidation^[^ ^36^ ^]^ of enantioenriched aryl ketone **3ce** with *m*‐CPBA furnished aryl ester **4** in 80% yield with 99% *ee* (Scheme 2b, top left). Alternatively, homologation of ketone **3ce** using TMSCH_2_N_2_ followed by in situ deprotection afforded benzylic ketone **5** (Scheme 2b, top right) in 64% yield without any erosion of enantiomeric excess.^[^ ^37^ ^]^ Deoxygenation of **3ce** with triethylsilane in TFA delivered the benzylic adduct **6** with 85% yield and ≥99% *ee* (Scheme 2b, bottom left).^[^ ^38^ ^]^ Chelation‐controlled reduction of aryl ketone **3ce** with TiCl_4_/DIBAL‐H at low temperature provided the differentially protected 1,3‐diol **7** in 84% yield with excellent selectivity (*dr* ≥19:1, 99% *ee*; Scheme 2b, bottom right).^[^ ^39^ ^]^ Overall, these transformations proceed with high stereochemical fidelity under diverse reaction conditions, highlighting the remarkable stability of the enantiomerically enriched α‐tertiary ketones and their broad utility in asymmetric synthesis.

(*R*)‐Cyclamen aldehyde and its analogs are among the most widely used fragrance compounds in the perfume industry.^[^ ^40^ ^]^ Although commercially available cyclamen aldehyde is generally used as a racemate, the enantiopure form possesses an olfactory profile that differs significantly from both the racemate and the opposite enantiomer. The previous synthesis of enantiopure cyclamen aldehyde relied on lipase‐catalyzed desymmetrization as a key step in a nine‐step sequence.^[^ ^41^ ^]^ We now disclose a concise and efficient synthesis of (*R*)‐cyclamen aldehyde leveraging our enantiospecific alkylation strategy (Scheme 2c). Starting from commercially available cuminaldehyde **8**, cyanohydrin **1x** was prepared in 88% yield via a reported procedure.^[^ ^20^, ^22^ ^]^ Enantiospecific alkylation with enantiomerically enriched tosylate (*R*)‐**2e** afforded ketone (*S*)‐**3x** **e** in 81% yield with excellent stereospecificity (98% *cee*). One‐pot deprotection and palladium‐catalyzed reduction of benzyl‐protected ketone (*S*)‐**3x** **e** under ambient pressure delivered primary alcohol (*R*)‐**9** in 75% yield. Oxidation of (*R*)‐**9** with Dess‐Martin periodinane afforded cyclamen aldehyde (*R*)‐**10** in excellent yield without loss of enantiopurity. This route enables rapid access to enantiopure cyclamen aldehyde **10** from inexpensive starting materials using operationally simple transformations. The synthesis also unequivocally establishes that the alkylation proceeds with *inversion of configuration* at the electrophilic carbon. Notably, the modular nature of this strategy facilitates the synthesis of structurally diverse enantioenriched cyclamen aldehyde derivatives by varying the cyanohydrin and tosylate components.

In conclusion, we have developed a straightforward enantiospecific alkylation of cyanohydrins with readily available enantioenriched secondary tosylates under transition‐metal‐free conditions. This method enables the efficient construction of enantiomerically enriched α‐tertiary ketones with excellent stereochemical fidelity and broad functional group tolerance in both the nucleophile and electrophile—a transformation that is typically challenging due to the inherent susceptibility of α‐tertiary ketones to racemization. Notably, cyanohydrins bearing di‐ or trisubstituted alkenyl groups undergo a cooperative stereoconvergent transformation of the *Z/E*‐olefin, furnishing α,β‐unsaturated chiral ketones with excellent stereoselectivity, and exquisite enantiospecificity. The resulting enantioenriched α‐tertiary ketones can be readily converted into a variety of synthetically valuable intermediates using classical transformations without racemization. The utility and flexibility of the method are further exemplified by a concise synthesis of (*R*)‐cyclamen aldehyde. Overall, although many enantioselective methods are well‐established, we anticipate that this *transition‐metal‐free* enantiospecific alkylation will prove broadly valuable, both for its synthetic utility and operational simplicity.

## Supporting Information

Experimental procedures, characterization data, and copies of NMR and HPLC spectra for all compounds. The authors have cited additional references within the Supporting Information.^[^ ^20^, ^22^, ^36^, ^37^, ^38^, ^39^, ^42^, ^43^, ^44^, ^45^, ^46^, ^47^, ^48^, ^49^, ^50^ ^]^

## Conflict of Interests

The authors declare no conflict of interest.

## Supporting information



Supporting Information

## Data Availability

The data that support the findings of this study are available in the Supporting Information of this article.
