# Supplementary material for: Stereospecific Transition‐Metal‐Free Alkylation of Chiral Non‐Racemic Secondary Tosylates with Cyanohydrins: Convenient Access to Enantiomerically Enriched α‐Tertiary Ketones
Source: Angew Chem Int Ed Engl. 2026 Jan 13;65(6):e20674. doi: 10.1002/anie.202520674 (PMC12865256; doi:10.1002/anie.202520674)
Supplement: Supplementary file 1 — Supporting Information [file ANIE-65-e20674-s001.pdf]

Stereospecific Transition-Metal-Free Alkylation of Chiral Non-Racemic Secondary Tosylates with Cyanohydrins: Convenient Access to Enantiomerically Enriched α-Tertiary Ketones

Jinjin Ma,[a] Hui Li,[a,b] Jadab Majhi,[a] and P. Andrew Evans*[,a,c]

[a] Department of Chemistry, Queen's University, 90 Bader Lane, Kingston ON, K7L 3N6, Canada

[b] College of Chemistry & Pharmacy, Northwest A&F University, Yangling 712100, Shaanxi, P. R. of China

[c] Xiangya School of Pharmaceutical Sciences, Central South University, Changsha 410013, Hunan, P. R. of China

Contents of Supporting Information:

1	General Information	S2
2	Unsuccessful Substrates	S3
3	Proof of Absolute Configuration and Inversion of Stereochemistry	S3
4	General Experimental Procedures for the Synthesis of Cyanohydrins	S4
5	Spectral Data for the Cyanohydrins **1a-1w**	S4
6	General Experimental Procedure for the Synthesis of Chiral Non-Racemic Secondary Tosylates	S16
7	Spectral Data for the Secondary Tosylates **2a-2m**	S17
8	General Experimental Procedures for the Alkylation of Chiral Non-Racemic Secondary Tosylates with Cyanohydrins (Methods A-C)	S24
9	Spectral Data for the Enantioenriched *α*-Tertiary Ketones **3aa-3cm**	S26
10	Experimental Procedures and Characterization Data for Ester **4**, Homologated Ketone **5**, Ether **6**, and Benzylic Alcohol **7**	S48
11	Experimental Procedures for the Preparation and Characterization Data for (*R*)-Cyclamen Aldehyde **(10)**	S51
12	Copies of Proton and Carbon NMR Spectra	S54
13	HPLC Chromatograms	S201

1. General Information

All reactions were conducted under an argon atmosphere in oven-dried glassware with magnetic stirring. Reagents were purchased at the highest commercial quality and used without further purification, unless otherwise stated. Reaction solvents were dried using an Innovative Technology, Inc. solvent purification system by passage through activated alumina columns. Tetrahydrofuran (THF) was freshly distilled from sodium benzophenone ketyl. Analytical thin-layer chromatography (TLC) was performed on pre-coated 0.2 mm thick silica gel 60-F_{254} plates (*Merck*); visualized using UV light and/or $KMnO_4$ solution followed by heating. All compounds were purified by flash chromatography using silica gel 60 (40-63 µm, *Silicycle*) and provided spectroscopic data consistent with ≥95% of the assigned structure. Melting points (uncorrected) were obtained from a Cole-Parmer MP-250 D-F melting point instrument. ^{1}H NMR and ^{13}C NMR spectra were recorded on a Bruker Avance DRX-500 spectrometer in $CDCl_3$ at ambient temperature; chemical shifts (δ) are given in ppm and calibrated using the signal of residual undeuterated solvent as internal reference for ^{1}H NMR (δ_H = 7.26 ppm for $CDCl_3$) and using the signal of the deuterated solvent for ^{13}C NMR (δ_C = 77.16 ppm for $CDCl_3$). ^{1}H NMR data are reported as follows: chemical shift (multiplicity, 1st order spin system if available, coupling constant, integration). Coupling constants (*J*) are reported in Hz, and apparent splitting patterns are designated using the following abbreviations: s (singlet), d (doublet), t (triplet), q (quartet), m (multiplet), br (broad), app. (apparent) and the appropriate combinations. ^{13}C NMR spectra with proton decoupling were described with the aid of an APT sequence, separating methylene and quaternary carbons (e, even) from methyl and methine carbons (o, odd). IR spectra were recorded on an Agilent Technologies Cary 630 FT-IR (ATR) spectrometer; wavenumbers (ν) are given in cm^{-1}; and the abbreviations w (weak, <33%), m (medium, 33-66%), s (strong, 66-94%), vs (very strong, ≥95%) and br (broad) are used to describe the relative intensities of the IR absorbance bands. Mass spectra were obtained through the AIMS Mass Spectrometry Laboratory at the University of Toronto. All HPLC analyses were performed on an Agilent 1260 Infinity series instrument equipped with a variable-wavelength UV detector. The instrument was fitted with a CHIRALPAK™ AD-H, CHIRALPAK™ AS-H, CHIRALPAK™ IA-H and CHIRALCEL™ OJ-H column (Diacel, 4.6 mm × 25 cm). Optical rotations were measured in a 1-dm cell with an Anton Paar MCP 200 polarimeter.

2. Unsuccessful Substrates

Substrates Unable to Prepare:

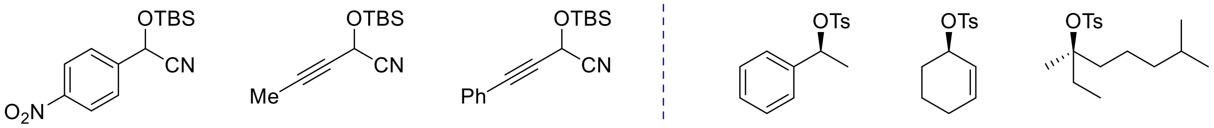

Substrates that Failed:

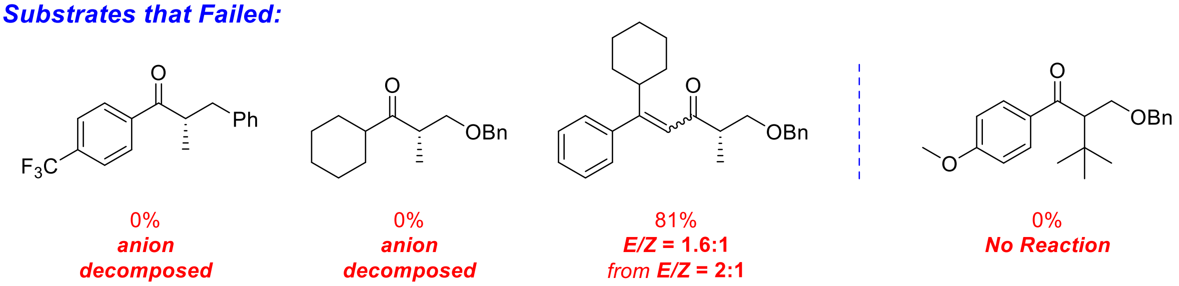

The following cyanohydrins could not be obtained because the corresponding aldehydes decomposed under the standard reaction conditions. Likewise, the benzylic and allylic tosylates decomposed under all conditions evaluated, including the general experimental procedure for the synthesis of chiral non-racemic secondary tosylates (Page S17, Part 5). The additional conditions examined for the preparation of these tosylates from the corresponding alcohols were: (1). NMI (1.5 equiv.), TMDEA (1.5 equiv.), TsCl (1.5 equiv.), DCM, RT; (2). DMAP (1.1 equiv.), TEA (1.5 equiv.), TsCl (1.5 equiv.), DCM, RT; (3). *n*-BuLi (1.2 equiv.), TsCl (1.4 equiv.), THF, −78 °C to RT. Several attempted alkylation reactions with more challenging cyanohydrins also failed, further illustrating the limitations of the system.

3. Proof of Absolute Configuration and Inversion of Stereochemistry

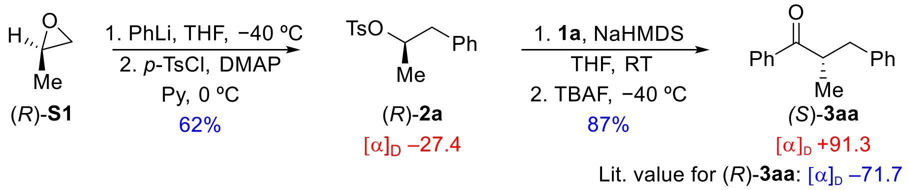

The absolute configuration of alkylated product (*S*)-**3aa** was determined by analogy to the work of Oppolzer and co-workers.[34] Although the optical rotation of (*S*)-**3aa** obtained in the present study is opposite in sign to the reported value, this observation indicates the formation of the opposite enantiomer and thereby corroborates the originally assigned absolute configuration. Notably, the original absolute-configuration assignment was made by inference, based on analogy

to a structurally related compound prepared using the same bornanesultam-mediated alkylation methodology, whose configuration was independently established through total synthesis.[34,35] Accordingly, the stereochemical outcome of the reaction of aryl cyanohydrin **1a** with chiral secondary tosylate (*R*)-**2a** is consistent with inversion of configuration, as expected for an S_N2 pathway. In addition, the stereospecific synthesis of (*R*)-cyclamen aldehyde (Supporting Information, p. S52) supports the configuration determination by analogy for (*S*)-**3xe**.[41] Collectively, these data indicate that alkylation of aryl cyanohydrin **1a** with chiral secondary tosylate (*R*)-**2a**, prepared from commercially available epoxide (*R*)-**S1**, proceeds stereospecifically with inversion of configuration.

4. General Experimental Procedures for the Synthesis of Cyanohydrins

Cyanohydrins were prepared from the corresponding aldehydes using known procedures:[20,22]

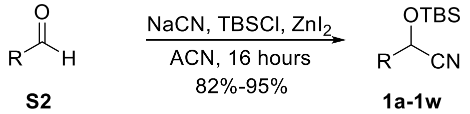

To a solution of aldehyde (**S2**, 3 mmol) in anhydrous MeCN (6 mL) was added anhydrous ZnI_2 (95.7 mg, 0.3 mmol, 0.1 equiv.), NaCN (1.18 g, 24 mmol, 8 equiv.) and *tert*-butyldimethylsilyl chloride (633 mg, 4.2 mmol, 1.4 equiv.). The resulting mixture was stirred for *ca.* 16 h (monitored by TLC). The reaction was quenched with distilled water and extracted with ethyl acetate (3x). The combined organic phases were dried (anhyd. $MgSO_4$), filtered, and concentrated *in vacuo* to afford the crude product. Purification by flash column chromatography (silica gel, eluting with 2-5% diethyl ether/hexane) afforded the cyanohydrin **1a-1w** in 82–95% yield as colorless oil or white solid.

CAUTION: *This procedure involves the use of highly toxic sodium cyanide. All operations must be carried out in a well-ventilated fume hood, and all cyanide-containing waste should be carefully quenched in accordance with established safety protocols.*

5. Spectral Data for the Cyanohydrins 1a-1w

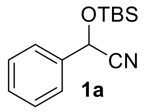

2-((*tert*-Butyldimethylsilyl)oxy)-2-phenylacetonitrile (1a)

Color and State: colorless oil

The characterization data are identical to those reported.[20]

¹H NMR (500 MHz, $CDCl_3$) δ 7.50-7.47 (m, 2H), 7.44-7.37 (m, 3H), 5.52 (s, 1H), 0.94 (s, 9H), 0.23 (s, 3H), 0.15 (s, 3H).

IR (Neat) 3068 (w), 3034 (w), 2956 (w), 2930 (w), 2885 (w), 2859 (w), 1603 (w), 1584 (w), 1495 (w), 1469 (w), 1454 (w), 1256 (m), 1092 (m), 1070 (m), 835 (s), 779 (s), 734 (m), 693 (s), 675 (m) cm^{-1}.

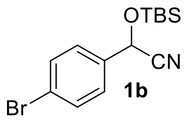

2-(4-Bromophenyl)-2-((*tert*-butyldimethylsilyl)oxy)acetonitrile (1b)

Color and State: yellow oil

The characterization data are identical to those reported.[22]

¹H NMR (500 MHz, $CDCl_3$) δ 7.57-7.54 (m, 2H), 7.36-7.33 (m, 2H), 5.46 (s, 1H), 0.94 (s, 9H), 0.23 (s, 3H), 0.16 (s, 3H).

IR (Neat) 2952 (w), 2930 (w), 2885 (w), 2859 (w), 1592 (w), 1487 (w), 1469 (w), 1401 (w), 1256 (m), 1111 (m), 1088 (m), 1070 (m), 1010 (m), 835 (s), 779 (s), 678 (m) cm^{-1}.

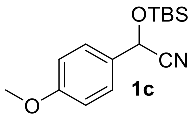

2-((*tert*-Butyldimethylsilyl)oxy)-2-(4-methoxyphenyl)acetonitrile (1c)

Color and State: colorless oil

The characterization data are identical with those reported.[22]

¹H NMR (500 MHz, $CDCl_3$) δ 7.40-7.37 (m, 2H), 6.94-6.91 (m, 2H), 5.46 (s, 1H), 3.83 (s, 3H), 0.93 (s, 9H), 0.21 (s, 3H), 0.13 (s, 3H).

IR (Neat) 3001 (w), 2956 (w), 2930 (w), 2889 (w), 2859 (w), 1610 (w), 1588 (w), 1510 (m), 1465 (w), 1249 (s), 1174 (m), 1081 (m), 1032 (m), 835 (s), 779 (s), 671 (m) cm^{-1}.

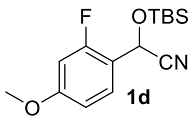

2-((*tert*-Butyldimethylsilyl)oxy)-2-(2-fluoro-4-methoxyphenyl)acetonitrile (1d)

Color and State: colorless oil

¹H NMR (500 MHz, $CDCl_3$) δ 7.52 (t, J = 8.6 Hz, 1H), 6.76 (dd, J = 8.6, 1.7 Hz, 1H), 6.63 (dd, J = 11.9, 2.3 Hz, 1H), 5.69 (s, 1H), 3.82 (s, 3H), 0.91 (s, 9H), 0.22 (s, 3H), 0.12 (s, 3H).

^{13}C NMR (125 MHz, $CDCl_3$) δ 162.05 (e, d, $^{3}J_{C\text{-}F}$ = 11.0 Hz), 160.26 (e, d, $^{1}J_{C\text{-}F}$ = 248.2 Hz), 129.19 (o, d, $^{3}J_{C\text{-}F}$ = 5.5 Hz), 118.79 (e), 116.26 (e, d, $^{2}J_{C\text{-}F}$ = 14.7 Hz), 110.70 (o, d, $^{4}J_{C\text{-}F}$ = 3.7 Hz), 101.83 (o, d, $^{2}J_{C\text{-}F}$ = 24.4 Hz), 58.04 (o, d, $^{3}J_{C\text{-}F}$ = 3.7 Hz), 55.83 (o), 25.62 (o), 18.25 (e), –5.11 (o), –5.18 (o).

^{19}F NMR (471 MHz, $CDCl_3$) δ –116.81.

IR (Neat) 3012 (w), 2956 (w), 2933 (w), 2889 (w), 2859 (w), 1625 (m), 1588 (w), 1506 (m), 1465 (w), 1252 (m), 1155 (m), 1107 (m), 1077 (s), 1029 (m), 950 (m), 835 (vs), 779 (s), 675 (m) cm^{-1}.

HRMS (DART) calcd. for $C_{15}H_{26}FN_2O_2Si$ $[M+NH_4]^+$: 313.1742, found: 313.1749.

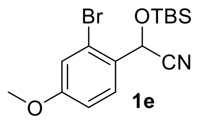

1e

2-((*tert*-Butyldimethylsilyl)oxy)-2-(2-bromo-4-methoxyphenyl)-acetonitrile (1e)

Color and State: colorless oil

^{1}H NMR (500 MHz, $CDCl_3$) δ 7.61 (d, J = 8.8 Hz, 1H), 7.11 (d, J = 2.4 Hz, 1H), 6.94 (dd, J = 8.6, 2.4 Hz, 1H), 5.73 (s, 1H), 3.82 (s,3H), 0.93 (s, 9H), 0.24 (s, 3H), 0.15 (s, 3H).

^{13}C NMR (125 MHz, $CDCl_3$) δ 160.89 (e), 129.39 (o), 127.99 (e), 122.14 (e), 118.77 (e), 118.22 (o), 114.33 (o), 63.36 (o), 55.79 (o), 25.67 (o), 18.28 (e), –4.98 (o), –5.00 (o).

IR (Neat) 3008 (w), 2952 (w), 2930 (w), 2892 (w), 2859 (w), 1603 (m), 1569 (w), 1491 (m), 1465 (m), 1282 (m), 1256 (m), 1234 (s), 1092 (m), 1029 (m), 835 (s), 779 (s), 675 (m) cm^{-1}.

HRMS (DART) calcd. for $C_{15}H_{26}{}^{79}BrN_2O_2Si$ $[M+NH_4]^+$: 373.0941, found: 373.0951.

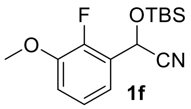

1f

2-((*tert*-Butyldimethylsilyl)oxy)-2-(2-fluoro-3-methoxyphenyl)acetonitrile (1f)

Color and State: colorless oil

^{1}H NMR (500 MHz, $CDCl_3$) δ 7.22-7.19 (m, 1H), 7.15 (td, J = 8.0, 1.1 Hz, 1H), 7.01 (td, J = 8.1, 1.3 Hz, 1H), 5.77 (s, 1H), 3.90 (s, 3H), 0.92 (s, 9H), 0.24 (s, 3H), 0.14 (s, 3H).

^{13}C NMR (125 MHz, $CDCl_3$) δ 149.42 (e, d, $^{1}J_{C\text{-}F}$ = 249.1 Hz), 147.84 (e, d, $^{2}J_{C\text{-}F}$ = 10.1 Hz), 125.08 (e, d, $^{2}J_{C\text{-}F}$ = 11.1 Hz), 124.72 (o, d, $^{4}J_{C\text{-}F}$ = 5.5 Hz), 119.25 (o, d, $^{3}J_{C\text{-}F}$ = 1.8 Hz), 118.50 (e), 114.51 (o, d, $^{3}J_{C\text{-}F}$ = 1.8 Hz), 58.17 (o, d, $^{3}J_{C\text{-}F}$ = 6.4 Hz), 56.59 (o), 25.63 (o), 18.28 (e), –5.11 (o), –5.19 (o).

^{19}F NMR (471 MHz, $CDCl_3$) δ –140.91.

IR (Neat) 3008 (w), 2952 (w), 2933 (w), 2889 (w), 2859 (w), 1621 (w), 1588 (w), 1491 (s), 1465 (m), 1278 (m), 1256 (m), 1208 (m), 1062 (s), 835 (s), 779 (s), 742 (m), 719 (m), 675 (m) cm^{-1}.

HRMS (DART) calcd. for $C_{15}H_{26}FN_2O_2Si$ $[M+NH_4]^+$: 313.1742, found: 313.1753.

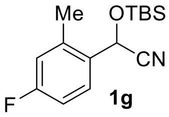

1g

2-((*tert*-Butyldimethylsilyl)oxy)-2-(4-fluoro-2-methylphenyl)acetonitrile (1g)

Color and State: colorless oil

^{1}H NMR (500 MHz, $CDCl_3$) δ 7.47 (dd, J = 8.2, 5.8 Hz, 1H), 6.96-6.91 (m, 2H), 5.52 (s, 1H), 2.44 (s, 3H), 0.93 (s, 9H), 0.22 (s, 3H), 0.13 (s, 3H).

^{13}C NMR (125 MHz, $CDCl_3$) δ 163.14 (e, d, $^1J_{C\text{-}F}$ = 248.2 Hz), 138.37 (e, d, $^3J_{C\text{-}F}$ = 8.3 Hz), 130.54 (e, d, $^4J_{C\text{-}F}$ = 2.7 Hz), 128.95 (o, d, $^3J_{C\text{-}F}$ = 9.2 Hz), 118.76 (e), 118.05 (o, d, $^2J_{C\text{-}F}$ = 22.1 Hz), 113.34 (o, d, $^2J_{C\text{-}F}$ = 21.2 Hz), 62.03 (o), 25.63 (o), 19.00 (o), 18.27 (e), –4.98 (o), –5.06 (o).

^{19}F NMR (471 MHz, $CDCl_3$) δ –116.35.

IR (Neat) 2956 (w), 2930 (w), 2889 (w), 2859 (w), 1614 (w), 1592 (w), 1495 (m), 1469 (w), 1256 (m), 1245 (m), 1114 (m), 1077 (s), 835 (s), 779 (s) 675 (m) cm^{-1}.

HRMS (DART) calcd. for $C_{15}H_{26}FN_2OSi$ $[M+NH_4]^+$: 297.1793, found: 297.1801.

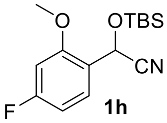

1h

2-((*tert*-Butyldimethylsilyl)oxy)-2-(4-fluoro-2-methoxyphenyl)acetonitrile (1h)

Color and State: colorless oil

^{1}H NMR (500 MHz, $CDCl_3$) δ 7.54 (dd, J = 8.2, 6.8 Hz, 1H), 6.72 (td, J = 8.3, 2.2 Hz, 1H), 6.63 (dd, J = 10.4, 2.2 Hz, 1H), 5.71 (s, 1H), 3.87 (s, 3H), 0.93 (s, 9H), 0.23 (s, 3H), 0.13 (s, 3H).

^{13}C NMR (125 MHz, $CDCl_3$) δ 164.30 (e, d, $^1J_{C\text{-}F}$ = 248.2 Hz), 157.12 (e, d, $^3J_{C\text{-}F}$ = 11.1 Hz), 128.46 (o, d, $^3J_{C\text{-}F}$ = 10.1 Hz), 120.97 (e, d, $^4J_{C\text{-}F}$ = 2.8 Hz), 119.27 (e), 107.56 (o, d, $^2J_{C\text{-}F}$ = 21.2 Hz), 99.21 (o, d, $^2J_{C\text{-}F}$ = 25.8 Hz), 58.54 (o), 55.94 (o), 25.69 (o), 18.33 (e), –5.07 (o), –5.16 (o).

^{19}F NMR (471 MHz, $CDCl_3$) δ –109.60.

IR (Neat) 2956 (w), 2933 (w), 2885 (w), 2859 (w), 1610 (m), 1502 (m), 1465 (m), 1416 (w), 1282 (m), 1256 (m), 1193 (m), 1152 (m), 1111 (m), 1085 (s), 1032 (m), 954 (m), 831 (vs), 779 (s), 675 (m) cm^{-1}.

HRMS (DART) calcd. for $C_{15}H_{26}FN_2O_2Si$ $[M+NH_4]^+$: 313.1742, found: 313.1746.

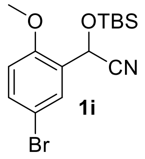

2-(5-Bromo-2-methoxyphenyl)-2-((*tert*-butyldimethylsilyl)oxy)acetonitrile (1i)

Color and State: colorless oil

^{1}H NMR (500 MHz, $CDCl_3$) δ 7.68 (d, *J* = 1.8 Hz, 1H), 7.45 (dd, *J* = 8.6, 2.2 Hz, 1 H), 6.78 (d, *J* = 8.8 Hz, 1H), 5.71 (s, 1H), 3.87 (s, 3H), 0.95 (s, 9H), 0.25 (s, 3H), 0.15 (s, 3H).

^{13}C NMR (125 MHz, $CDCl_3$) δ 154.94 (e), 133.09 (o), 130.01 (o), 126.99 (e), 118.86 (e), 113.38 (e), 112.43 (o), 58.41 (o), 55.94 (o), 25.70 (o), 18.35 (e), –5.07 (o), –5.16 (o).

IR (Neat) 3004 (w), 2952 (w), 2930 (w), 2889 (w), 2859 (w), 2855 (w), 1595 (w), 1577 (w), 1472 (m), 1405 (w), 1282 (m), 1265 (m), 1230 (m), 1163 (m), 1129 (m), 1096 (s), 1055 (m), 1014 (m), 835 (s), 779 (s), 675 (m) cm^{-1}.

HRMS (DART) calcd. for $C_{15}H_{26}{}^{79}BrN_2O_2Si$ $[M+NH_4]^+$: 373.0941, found: 373.0934.

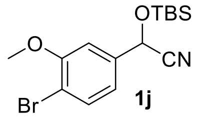

2-(4-Bromo-3-methoxyphenyl)-2-((*tert*-butyldimethylsilyl)oxy)-acetonitrile (1j)

Color and State: colorless oil

^{1}H NMR (500 MHz, $CDCl_3$) δ 7.56 (d, *J* = 8.1 Hz, 1H), 7.03 (s, 1H), 6.91 (dd, *J* = 8.1, 1.0 Hz, 1H), 5.47 (s, 1H), 3.92 (s, 3H), 0.95 (s, 9H), 0.24 (s, 3H), 0.16 (s, 3H).

^{13}C NMR (125 MHz, $CDCl_3$) δ 156.49 (e), 137.40 (e), 133.81 (o), 119.32 (o), 118.95 (e), 112.82 (e), 109.54 (o), 63.60 (o), 56.41 (o), 25.65 (o), 18.31 (e), –4.92 (o), –5.08 (o).

IR (Neat) 3008 (w), 2952 (w), 2930 (w), 2885 (w), 2859 (w), 1592 (w), 1483 (m), 1461 (m), 1405 (m), 1282 (m), 1252 (m), 1167 (m), 1092 (m), 1047 (m), 1025 (m), 835 (s), 779 (s), 678 (m) cm^{-1}.

HRMS (DART) calcd. for $C_{15}H_{26}{}^{79}BrN_2O_2Si$ $[M+NH_4]^+$: 373.0941, found: 373.0945.

2-((*tert*-Butyldimethylsilyl)oxy)-2-(3-chloro-5-methylphenyl)acetonitrile (1k)

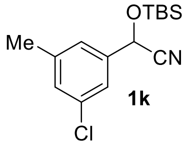

Color and State: white solid, m.p. = 68-69.5 °C.

^{1}H NMR (500 MHz, $CDCl_3$) δ 7.26 (s, 1H), 7.19 (s, 1H), 7.15 (s, 1H), 5.44 (s, 1H), 2.37 (s, 3H), 0.95 (s, 9H), 0.24 (s, 3H), 0.17 (s, 3H).

^{13}C NMR (125 MHz, $CDCl_3$) δ 140.80 (e), 138.26 (e), 134.74 (e), 130.17 (o), 125.06 (o), 123.48 (o), 118.99 (e), 63.49 (o), 25.66 (o), 21.33 (o), 18.31 (e), –4.96 (o), –5.06 (o).

IR (Neat) 2960 (w), 2933 (w), 2900 (w), 2863 (w), 1606 (w), 1584 (w), 1454 (w), 1346 (w), 1256 (m), 1167 (w), 1055 (m), 999 (m), 857 (m), 835 (s), 779 (s), 690 (m), 667 (m) cm^{-1}.

HRMS (DART) calcd. for $C_{15}H_{26}{}^{35}ClN_2OSi$ $[M+NH_4]^+$: 313.1497, found: 313.1494.

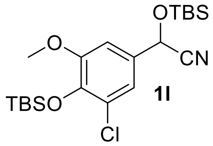

2-((*tert*-Butyldimethylsilyl)oxy)-2-(4-((*tert*-butyldimethylsilyl)oxy)-3-chloro-5-methoxyphenyl)acetonitrile (1l)

Color and State: colorless oil

^{1}H NMR (500 MHz, $CDCl_3$) δ 7.03 (d, *J* = 1.3 Hz, 1H), 6.87 (d, *J* = 1.4 Hz, 1H), 5.40 (s, 1H), 3.82 (s, 3H), 1.04 (s, 9H), 0.94 (s, 9H), 0.22 (s, 3H), 0.21 (s, 3H), 0.21 (s, 3H), 0.15 (s, 3H).

^{13}C NMR (125 MHz, $CDCl_3$) δ 151.94 (e), 142.64 (e), 129.55 (e), 126.16 (e), 119.92 (o), 119.15 (e), 107.86 (o), 63.54 (o), 55.56 (o), 25.98 (o), 25.67 (o), 18.98 (e), 18.31 (e), –3.92 (o), –3.94 (o), –4.92 (o), –5.01 (o).

IR (Neat) 2952 (w), 2930 (w), 2885 (w), 2859 (w), 1577 (w), 1495 (m), 1465 (m), 1319 (m), 1290 (m), 1252 (m), 1148 (m), 1096 (m), 1051 (m), 898 (m), 835 (s), 805 (s), 779 (s), 712 (m), 675 (m) cm^{-1}.

HRMS (DART) calcd. for $C_{21}H_{40}{}^{35}ClN_2O_3Si_2$ $[M+NH_4]^+$: 459.2261, found: 459.2266.

2-((*tert*-Butyldimethylsilyl)oxy)-2-(3,4,5-trimethoxyphenyl)acetonitrile (1m)

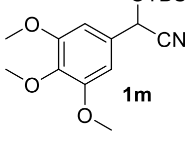

Color and State: colorless oil

^{1}H NMR (500 MHz, $CDCl_3$) δ 6.68 (s, 2H), 5.45 (s, 1H), 3.87 (s, 6H), 3.85 (s, 3H), 0.95 (s, 9H), 0.23 (s, 3H), 0.16 (s, 3H).

^{13}C NMR (125 MHz, $CDCl_3$) δ 153.73 (e), 138.74 (e), 132.06 (e), 119.32 (e), 103.17 (o), 63.98 (o), 61.00 (o), 56.31 (o), 25.66 (o), 18.32 (e), –4.92 (o), –5.06 (o).

IR (Neat) 3001 (w), 2952 (w), 2933 (w), 2885 (w), 2852 (w), 1595 (m), 1506 (m), 1461 (m), 1420 (m), 1331 (m), 1234 (m), 1148 (m), 1126 (s), 1100 (s), 1006 (m), 835 (s), 805 (s), 779 (s), 704 (m), 675 (m) cm^{-1}.

HRMS (DART) calcd. for $C_{17}H_{28}NO_4Si$ $[M+H]^+$: 338.1782, found: 338.1775.

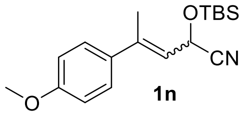

1n

2-((*tert*-Butyldimethylsilyl)oxy)-4-(4-methoxyphenyl)pent-3-enenitrile (1n)

Color and State: colorless oil, ***E/Z*** = 3:1

^{1}H NMR (500 MHz, $CDCl_3$) δ 7.37-7.34 (m, 2H for *E* isomer), 7.13-7.10 (m, 2H for *Z* isomer), 6.94-6.88 (m, 2H for *E* isomer and 2H for *Z* isomer), 5.80 (dq, *J* = 8.1, 1.3 Hz, 1H for *E* isomer), 5.60 (dq, *J* = 9.2, 1.5 Hz, 1H for *Z* isomer), 5.29 (d, *J* = 8.1 Hz, 1H for *E* isomer), 4.88 (d, *J* = 9.1 Hz, 1H for *Z* isomer), 3.83 (s, 3H for *Z* isomer), 3.82 (s, 3H for *E* isomer), 2.12 (d, *J* = 1.4 Hz, 3H for *E* isomer), 2.09 (d, *J* = 1.4 Hz, 3H for *Z* isomer), 0.94 (s, 9H for *E* isomer), 0.86 (s, 9H for *Z* isomer), 0.21 (s, 3H for *E* isomer), 0.18 (s, 3H for *E* isomer), 0.00 (s, 3H for *Z* isomer), –0.01 (s, 3H for *Z* isomer).

^{13}C NMR (125 MHz, $CDCl_3$) δ 159.80 (e), 159.49 (e), 143.05 (e), 140.20 (e), 133.85 (e), 131.78 (e), 128.82 (o), 127.20 (o), 122.63 (o), 121.60 (o), 119.83 (e), 119.24 (e), 114.06 (o), 113.91 (o), 60.08 (o), 59.52 (o), 55.39 (o), 55.38 (o), 25.63 (o), 25.59 (o), 25.33 (o), 18.19 (e), 18.09 (e), 16.67 (o), –4.78 (o), –4.80 (o), –4.88 (o), –5.03 (o).

IR (Neat) 2997 (w), 2952 (w), 2930 (w), 2900 (w), 2859 (w), 1640 (w), 1606 (m), 1573 (w), 1510 (m), 1461 (m), 1442 (w), 1290 (w), 1249 (s), 1182 (m), 1114 (m), 1085 (m), 1051 (m), 1032 (s), 835 (s), 779 (s), 708 (m), 671 (m) cm^{-1}.

HRMS (DART) calcd. for $C_{18}H_{28}NO_2Si$ $[M+H]^+$: 318.1884, found: 318.1882.

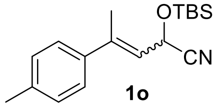

1o

2-((*tert*-Butyldimethylsilyl)oxy)-4-(*p*-tolyl)pent-3-enenitrile (1o)

Color and State: colorless oil, ***E/Z*** = 1.6:1

^{1}H NMR (500 MHz, $CDCl_3$) δ 7.31-7.29 (m, 2H for *E* isomer), 7.20-7.16 (m, 2H for *E* isomer and 2H for *Z* isomer), 7.07-7.05 (m, 2H for *Z* isomer), 5.82 (d, *J* = 8.0 Hz, 1H for *E* isomer), 5.61 (d, *J* = 9.1 Hz, 1H for *Z* isomer), 5.28 (d, *J* = 8.0 Hz, 1H for *E* isomer), 4.84 (d, *J* = 9.2 Hz, 1H for *Z* isomer), 2.37 (s, 3H for *Z* isomer), 2.36 (s, 3H for *E* isomer), 2.12 (s, 3H for *E* isomer), 2.09 (s, 3H for *Z* isomer), 0.93 (s, 9H for *E* isomer), 0.84 (s, 9H for *Z* isomer), 0.21 (s, 3H for *E* isomer), 0.17 (s, 3H for *E* isomer), –0.01 (s, 3H for *Z* isomer), –0.02 (s, 3H for Z isomer).

^{13}C NMR (125 MHz, $CDCl_3$) δ 143.52 (e), 140.72 (e), 138.68 (e), 138.27 (e), 138.06 (e), 136.66 (e), 129.39 (o), 129.30 (o), 127.51 (o), 125.97 (o), 122.80 (o), 122.49 (o), 119.87 (e), 119.24 (e), 60.10 (o), 59.54 (o), 25.68 (o), 25.64 (o), 25.40 (o), 21.33 (o), 21.25 (o), 18.26 (e), 18.14 (e), 16.77 (o), –4.75 (o), –4.84 (o), –4.99 (o).

IR (Neat) 3027 (w), 2952 (w), 2930 (w), 2885 (w), 2859 (w), 1644 (w),1610 (w), 1569 (w), 1513 (w), 1469 (w), 1442 (w), 1252 (m), 1114 (m), 1081 (m), 1051 (m), 835 (s), 809 (s), 779 (s), 671 (m) cm^{-1}.

HRMS (DART) calcd. for $C_{18}H_{31}N_2OSi$ $[M+NH_4]^+$: 319.2200, found: 319.2199.

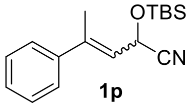

1p

2-((*tert*-Butyldimethylsilyl)oxy)-4-phenylpent-3-enenitrile (1p)

Color and State: colorless oil, ***E/Z*** = 3.3:1

^{1}H NMR (500 MHz, $CDCl_3$) δ 7.43-7.40 (m, 2H for *E* isomer and 1H for *Z* isomer), 7.39-7.32 (m, 3H for *E* isomer and 2H for *Z* isomer), 7.20-7.17 (m, 2H for *Z* isomer), 5.86 (dq, *J* = 8.0, 1.3 Hz, 1H for *E* isomer), 5.66 (dq, *J* = 9.1, 1.5 Hz, 1H for Z isomer), 5.31 (d, *J* = 8.1 Hz, 1H for *E* isomer), 4.84 (d, *J* = 9.2 Hz, 1H for *Z* isomer), 2.16 (d, *J* = 1.4 Hz, 3H for *E* isomer), 2.13 (d, *J* = 1.5 Hz, 3H for *Z* isomer), 0.95 (s, 9H for *E* isomer), 0.86 (s, 9H for *Z* isomer), 0.23 (s, 3H for *E* isomer), 0.20 (s, 3H for *E* isomer), 0.00 (s, 3H for *Z* isomer), –0.01 (s, 3H for *Z* isomer).

^{13}C NMR (125 MHz, $CDCl_3$) δ143.55 (e), 141.57 (e), 140.89 (e), 139.60 (e), 128.72 (o), 128.58 (o), 128.29 (o), 128.20 (o), 127.53 (o), 126.06 (o), 123.28 (o), 123.04 (o), 119.72 (e), 119.08 (e), 59.99 (o), 59.46 (o), 25.64 (o), 25.59 (o), 25.32 (o), 18.20 (e), 18.09 (e), 16.78 (o), –4.79 (o), –4.95 (o), –5.07 (o).

IR (Neat) 3083 (w), 3060 (w), 3030 (w), 2952 (w), 2930 (w), 2889 (w), 2859 (w), 1644 (w), 1599 (w), 1577 (w), 1495 (w), 1469 (w), 1442 (w), 1360 (w), 1252 (m), 1114 (m), 1088 (m), 1051 (m), 831 (s), 779 (s), 757 (s), 697 (s), 671 (m) cm^{-1}.

HRMS (DART) calcd. for $C_{17}H_{29}N_2OSi$ $[M+NH_4]^+$: 305.2044, found: 305.2040.

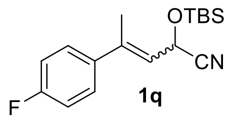

1q

2-((*tert*-Butyldimethylsilyl)oxy)-4-(4-fluorophenyl)pent-3-enenitrile (1q)

Color and State: colorless oil, ***E/Z*** = 2.5:1

^{1}H NMR (500 MHz, $CDCl_3$) δ 7.39-7.35 (m, 2H for *E* isomer), 7.18-7.14 (m, 2H for *Z* isomer), 7.11-7.02 (m, 2H for *E* isomer and 2H for *Z* isomer), 5.80 (dq, *J* = 8.0, 1.3 Hz, 1H for *E* isomer), 5.65 (dq, *J* = 9.1, 1.5 Hz, 1H for *Z* isomer), 5.27 (d, *J* = 8.1 Hz, 1H for *E* isomer), 4.78 (d, *J* = 9.1 Hz, 1H for *Z* isomer), 2.13 (d, *J* = 1.4 Hz, 3H for *E* isomer), 2.10 (d, *J* = 1.5 Hz, 3H for *Z* isomer), 0.93 (s, 9H for *E* isomer), 0.85 (s, 9H for *Z* isomer), 0.22 (s, 3H for *E* isomer), 0.18 (s, 3H for *E* isomer), 0.01 (s, 3H for *Z* isomer), –0.02 (s, 3H for *Z* isomer). .

^{13}C NMR (125 MHz, $CDCl_3$) δ 162.83 (e, d, $^1J_{C\text{-}F}$ = 248.1 Hz), 162.52 (e, d, $^1J_{C\text{-}F}$ = 248.2 Hz), 142.44 (e), 139.87 (e), 137.63 (e, d, $^4J_{C\text{-}F}$ = 2.8 Hz), 135.52 (e, d, $^4J_{C\text{-}F}$ = 3.7 Hz), 129.32 (o, d, $^3J_{C\text{-}F}$ = 8.3 Hz), 127.77 (o, d, $^3J_{C\text{-}F}$ = 8.3 Hz), 123.52 (o), 123.28 (o), 119.57 (e), 119.02 (e), 115.77 (o, d, $^2J_{C\text{-}F}$ = 21.2 Hz), 115.47 (o, d, $^2J_{C\text{-}F}$ = 22.0 Hz), 59.91 (o), 59.42 (o), 25.64 (o), 25.59 (o), 25.42 (o), 18.22 (e), 18.11 (e), 16.91 (o), –4.81 (o), –4.94 (o), –5.01 (o).

^{19}F NMR (471 MHz, $CDCl_3$) δ –113.37 (*E* isomer), –113.88 (*Z* isomer).

IR (Neat) 2952 (w), 2930 (w), 2885 (w), 2859 (w), 1644 (w), 1588 (w), 1483 (w), 1469 (w), 1398 (w), 1252 (m), 1114 (m), 1088 (m), 1073 (s), 1051 (m), 1006 (m), 835 (s), 813 (s), 779 (s), 671 (m) cm^{-1}.

HRMS (DART) calcd. for $C_{17}H_{28}FN_2OSi$ $[M+NH_4]^+$: 323.1949, found: 323.1957.

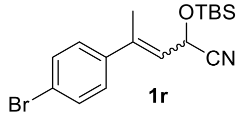

1r

4-(4-Bromophenyl)-2-((*tert*-butyldimethylsilyl)oxy)pent-3-enenitrile (1r)

Color and State: colorless oil, ***E/Z*** = 4:1

^{1}H NMR (500 MHz, $CDCl_3$) δ 7.54-7.51 (m, 2H for *Z* isomer), 7.49-7.47 (m, 2H for *E* isomer), 7.28-7.25 (m, 2H for *E* isomer), 7.07-7.04 (m, 2H for *Z* isomer), 5.83 (dq, *J* = 7.8, 0.7 Hz, 1H for *E* isomer), 5.66 (d, *J* = 9.0 Hz, 1H for *Z* isomer), 5.26 (d, *J* = 7.9 Hz, 1H for *E*

isomer), 4.76 (d, J = 9.1 Hz, 1H for Z isomer), 2.12 (s, 3H for E isomer), 2.09 (s, 3H for Z isomer), 0.93 (s, 9H for E isomer), 0.85 (s, 9H for Z isomer), 0.21 (s, 3H for E isomer), 0.18 (s, 3H for E isomer), 0.02 (s, 3H for Z isomer), –0.01 (s, 3H for Z isomer).

^{13}C NMR (125 MHz, $CDCl_3$) δ 142.26 (e), 140.49 (e), 139.87 (e), 138.50 (e), 132.02 (o), 131.77 (o), 129.27 (o), 127.74 (o), 123.89 (o), 123.77 (o), 122.44 (e), 122.37 (e), 119.49 (e), 118.92 (e), 59.89 (o), 59.43 (o), 25.67 (o), 25.62 (o), 25.26 (o), 18.26 (e), 18.14 (e), 16.75 (o), –4.76 (o), –4.77 (o), –4.86 (o), –4.90 (o).

IR (Neat) 2952 (w), 2930 (w), 2885 (w), 2859 (w), 1644 (w), 1588 (w), 1483 (w), 1469 (w), 1442 (w), 1398 (w), 1252 (m), 1114 (m), 1088 (m), 1073 (s), 1051 (m), 1006 (m), 835 (s), 813 (s), 779 (s), 671 (m) cm^{-1}.

HRMS (DART) calcd. for $C_{17}H_{28}{}^{79}BrN_2OSi$ $[M+NH_4]^+$: 383.1149, found: 383.1158.

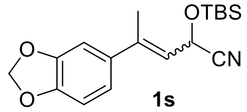

1s

4-(Benzo[d][1,3]dioxol-5-yl)-2-((*tert*-butyldimethylsilyl)oxy)pent-3-enenitrile (1s)

Color and State: colorless oil, ***E/Z*** = 4:1

^{1}H NMR (500 MHz, $CDCl_3$) δ 6.90-6.88 (m, 2H for E isomer), 6.82 (d, J = 7.9 Hz, 1H for Z isomer), 6.79 (d, J = 8.5 Hz, 1H for E isomer), 6.67 (d, J = 1.5 Hz, 1H for Z isomer), 6.63 (dd, J = 7.9, 1.5 Hz, 1H for Z isomer), 5.99 (s, 2H for Z isomer), 5.97 (s, 2H for E isomer), 5.75 (dq, J = 8.0, 1.2 Hz, 1H for E isomer), 5.59 (dq, J = 9.2, 1.4 Hz, 1H for Z isomer), 5.25 (d, J = 8.1 Hz, 1H for E isomer), 4.87 (d, J = 9.1 Hz, 1H for Z isomer), 2.09 (d, J = 0.8 Hz, 3H for E isomer), 2.07 (d, J = 1.2 Hz, 3H for Z isomer), 0.92 (s, 9H for E isomer), 0.86 (s, 9H for Z isomer), 0.20 (s, 3H for E isomer), 0.17 (s, 3H for E isomer), 0.03 (s, 3H for Z isomer), 0.01 (s, 3H for Z isomer).

^{13}C NMR (125 MHz, $CDCl_3$) δ 147.99 (e), 147.80 (e), 147.53 (e), 143.04 (e), 140.38 (e), 135.85 (e), 133.36 (e), 123.04 (o), 122.32 (o), 121.26 (o), 119.87 (o), 119.76 (e), 119.18 (e), 108.49 (o), 108.27 (o), 107.98 (o), 106.59 (o), 101.41 (e), 101.38 (e), 60.03 (o), 59.51 (o), 25.67 (o), 25.65 (o), 25.47 (o), 18.25 (e), 18.15 (e), 16.99 (o), –4.75 (o), –4.82 (o), –4.91 (o).

IR (Neat) 2952 (w), 2930 (w), 2889 (w), 2859 (w), 2777 (w), 1644 (w), 1606 (w), 1506 (m), 1487 (m), 1435 (m), 1241 (s), 1103 (m), 1073 (m), 1036 (s), 1006 (m), 936 (m), 831 (s), 805 (s), 779 (s), 671 (m) cm^{-1}.

HRMS (DART) calcd. for $C_{18}H_{26}NO_3Si$ $[M+H]^+$: 332.1677, found: 332.1680.

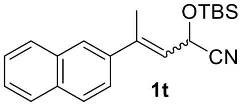

1t

2-((*tert*-Butyldimethylsilyl)oxy)-4-(naphthalen-2-yl)pent-3-enenitrile (1t)

Color and State: colorless oil, ***E/Z*** = 3.2:1

^{1}H NMR (500 MHz, $CDCl_3$) δ 7.88-7.82 (m, 4H for *E* isomer and 3H for *Z* isomer), 7.64 (s, 1H for *Z* isomer), 7.57-7.47 (m, 3H for *E* isomer and 2H for *Z* isomer), 7.30 (d, *J* = 8.4 Hz, 1H for *Z* isomer), 6.00 (d, *J* = 7.9 Hz, 1H for *E* isomer), 5.74 (d, *J* = 9.2 Hz, 1H for *Z* isomer), 5.35 (d, *J* = 7.9 Hz, 1H for *E* isomer), 4.89 (d, *J* = 9.3 Hz, 1H for *Z* isomer), 2.26 (s, 3H for *E* isomer), 2.20 (s, 3H for *Z* isomer), 0.96 (s, 9H for *E* isomer), 0.85 (s, 9H for *Z* isomer), 0.24 (s, 3H for *E* isomer), 0.21 (s, 3H for *E* isomer), –0.02 (s, 3H for *Z* isomer), –0.03 (s, 3H for *Z* isomer).

^{13}C NMR (125 MHz, $CDCl_3$) δ 143.52 (e), 140.76 (e), 138.77 (e), 137.10 (e), 133.39 (e), 133.24 (e), 132.98 (e), 128.63 (o), 128.40 (o), 128.27 (o), 128.19 (o), 127.89 (o), 127.72 (o), 126.78 (o), 126.64 (o), 126.61 (o), 126.58 (o), 126.47 (o), 125.37 (o), 125.25 (o), 124.06 (o), 123.81 (o), 123.53 (o), 119.78 (e), 119.17 (e), 60.12 (o), 59.62 (o), 25.70 (o), 25.64 (o), 25.45 (o), 18.29 (e), 18.13 (e), 16.87 (o), –4.71 (o), –4.84 (o), –4.93 (o).

IR (Neat) 3056 (w), 2952 (w), 2930 (w), 2885 (w), 2855 (w), 1640 (w), 1595 (w), 1502 (w), 1469 (w), 1442 (w), 1387 (w), 1360 (w), 1252 (m), 1111 (m), 1081 (m), 1051 (m), 1006 (w), 831 (vs), 813 (s), 779 (s), 745 (s), 671 (m) cm^{-1}.

HRMS (DART) calcd. for $C_{21}H_{28}NOSi$ $[M+H]^+$: 338.1935, found: 338.1937.

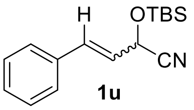

1u

2-((*tert*-Butyldimethylsilyl)oxy)-4-phenylbut-3-enenitrile (1u)

Color and State: slightly yellow oil, ***E/Z*** = 1:1

^{1}H NMR (500 MHz, $CDCl_3$) δ 7.42-7.37 (m, 3H for *E* isomer and 2H for *Z* isomer), 7.37-7.34 (m, 1H for *E* isomer and 1H for *Z* isomer), 7.33-7.30 (m, 1H for *E* isomer), 7.24-7.22 (m, 2H for *Z* isomer), 6.83-6.78 (m, 1H for *E* isomer and 1H for *Z* isomer), 6.19 (dd, *J* = 15.7, 5.8 Hz, 1H for *E* isomer), 5.81 (dd, *J* = 11.2, 8.9 Hz, 1H for *Z* isomer), 5.24 (dd, *J* = 8.9, 1.1 Hz, 1H for *Z* isomer), 5.14 (dd, *J* = 5.8, 1.5 Hz, 1H for *E* isomer), 0.95 (s, 9H for *E* isomer), 0.86 (s, 9H for *Z* isomer), 0.23 (s, 3H for *E* isomer), 0.20 (s, 3H for *E* isomer), 0.04 (s, 3H for *Z* isomer), 0.03 (s, 3H for *Z* isomer).

^{13}C NMR (125 MHz, $CDCl_3$) 135.26 (e), 135.11 (e), 134.10 (o), 133.81 (o), 128.91 (o), 128.81 (o), 128.74 (o), 128.46 (o), 127.27 (o), 127.11 (o), 123.87 (o), 119.26 (e), 118.61 (e), 62.81 (o), 58.87 (o), 25.70 (o), 25.59 (o), 18.34 (e), 18.17 (e), –4.79 (o), –4.85 (o), –4.90 (o), –4.94 (o).

IR (Neat) 3083 (w), 3060 (w), 3030 (w), 2956 (w), 2930 (w), 2885 (w), 2859 (w), 1655 (w), 1599 (w), 1577 (w), 1495 (w), 1469 (w), 1450 (w), 1256 (m), 1133 (m), 1100 (m), 1077 (m), 965 (m), 865 (m), 831 (s), 809 (m), 779 (s), 738 (m), 690 (s) cm^{-1}.

HRMS (DART) calcd. for $C_{16}H_{27}N_2OSi$ $[M+NH_4]^+$: 291.1887, found: 291.1891.

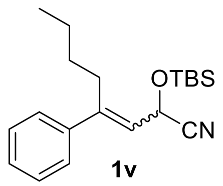

1v

2-((*tert*-Butyldimethylsilyl)oxy)-4-phenyloct-3-enenitrile (1v)

Color and State: colorless oil, ***E/Z*** **=** 1.2:1

^{1}H NMR (500 MHz, $CDCl_3$) δ 7.43-7.30 (m, 5H for *E* isomer and 3H for Z isomer), 7.14-7.12 (m, 2H for Z isomer), 5.71 (dt, *J* = 8.3, 0.8 Hz, 1H for *E* isomer), 5.61 (dt, *J* = 9.1, 1.2 Hz, 1H for *Z* isomer), 5.29 (d, *J* = 8.3 Hz, 1H for *E* isomer), 4.78 (d, *J* = 9.1 Hz, 1H for *Z* isomer), 2.54-2.51 (m, 2H for *E* isomer), 2.44-2.34 (m, 2H for *Z* isomer), 1.38-1.28 (m, 4H for *E* isomer and 4H for *Z* isomer), 0.93 (s, 9H for *E* isomer), 0.87 (t, *J* = 6.9 Hz, 3H for *E* isomer and 3H for *Z* isomer), 0.83 (s, 9H for *Z* isomer), 0.22 (s, 3H for *E* isomer), 0.18 (s, 3H for *E* isomer), –0.03 (s, 3H for *Z* isomer), –0.05 (s, 3H for *Z* isomer).

^{13}C NMR (125 MHz, $CDCl_3$) δ148.09 (e), 146.33 (e), 140.99 (e), 139.00 (e), 128.69 (o), 128.62 (o), 128.18 (o), 128.09 (o), 127.95 (o), 126.72 (o), 123.54 (o), 122.52 (o), 119.88 (e), 119.41 (e), 60.01 (o), 59.20 (o), 38.59 (e), 30.71 (e), 30.64 (e), 29.79 (e), 25.69 (o), 25.63 (o), 22.83 (e), 22.34 (e), 18.24 (e), 18.14 (e), 13.97 (o), –4.69 (o), –4.71 (o), –4.89 (o), –5.01 (o).

IR (Neat) 3083 (w), 3060 (w), 3027 (w), 2956 (w), 2930 (m), 2889 (w), 2859 (w), 1644 (w), 1599 (w), 1573 (w), 1495 (w), 1465 (w), 1442 (w), 1256 (m), 1092 (m), 1070 (m), 835 (s), 779 (s), 701 (m), 671 (w) cm^{-1}.

HRMS (DART) calcd. for $C_{20}H_{35}N_2OSi$ $[M+NH_4]^+$: 347.2513, found: 347.2521.

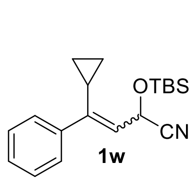

1w

2-((*tert*-Butyldimethylsilyl)oxy)-4-phenyloct-3-enenitrile (1w)

Color and State: colorless oil, ***E/Z*** **=** 1:5.5

^{1}H NMR (500 MHz, $CDCl_3$) δ 7.39-7.27 (m, 3H for *E* isomer and 3H for *Z* isomer), 7.14-7.12 (m, 2H for *E* isomer and 2H for *Z* isomer), 5.75 (d, *J* = 8.2 Hz, 1H for *E* isomer), 5.66 (d, *J* = 8.2 Hz, 1H for *E* isomer), 5.57 (d, *J* = 9.2 Hz, 1H for *Z* isomer), 4.69 (d, *J* = 9.1 Hz, 1H for *Z* isomer), 1.79-1.74 (m, 1H for *E* isomer), 1.64 (ddt, *J* = 13.3, 8.2, 5.2 Hz, 1H for *Z* isomer), 0.96-0.89 (m, 2H for *Z* isomer), 0.94 (s, 9H for *Z* isomer), 0.83 (s, 9H for *E* isomer), 0.82-0.70 (m, 2H for *E* isomer), 0.55 (app. sextet, *J* = 4.9 Hz, 1H for *Z* isomer), 0.51-0.44 (m, 1H for *E* isomer and 1H for *Z* isomer), 0.40-0.35 (m, 1H for *E* isomer), 0.23 (s, 3H for *E* isomer), 0.19 (s, 3H for *E* isomer), –0.03 (s, 3H for *Z* isomer), –0.05 (s, 3H for *Z* isomer).

^{13}C NMR (125 MHz, $CDCl_3$) δ149.12 (e), 146.17 (e), 139.95 (e), 137.42 (e), 128.54 (o), 128.49 (o), 128.19 (o), 128.16 (o), 127.87 (o), 127.58 (o), 126.04 (o), 120.70 (o), 119.77 (e), 119.32 (e), 60.02 (o), 59.20 (o), 25.70 (o), 25.63 (o), 18.23 (o), 18.11 (e), 11.89 (o), 7.07 (e), 6.55 (e), 6.45 (e), 5.54 (e), –4.72 (o), –4.89 (o), –5.02 (o).

IR (Neat) 3083 (w), 3060 (w), 3012 (w), 2952 (w), 2930 (w), 2889 (w), 2859 (w), 1644 (w), 1599 (w), 1573 (w), 1491 (w), 1469 (w), 1442 (w), 1252 (m), 1100 (m), 1070 (s), 924 (m), 835 (s), 779 (s), 701 (s), 671 (m) cm^{-1}.

HRMS (DART) calcd. for $C_{19}H_{31}N_2OSi$ $[M+NH_4]^+$: 331.2200, found: 331.2205.

6. General Experimental Procedure for the Synthesis of Chiral Non-Racemic Secondary Tosylates

The enantioenriched secondary tosylates were prepared from the corresponding secondary alcohols, most of which were either obtained from commercial sources (for **2k**, **2l, 2m**) or synthesized from the corresponding enantioenriched epoxides using organometal reagents (for **2b-2e**, **2f-2j**).[42] Tosylates **2a** and **2g** were prepared from the corresponding enantioenriched 1,2-diols using known procedures.[43]

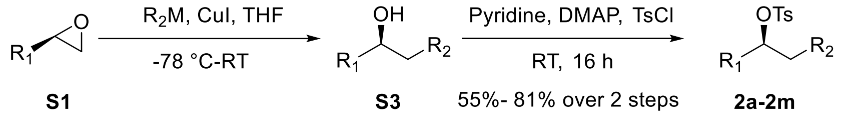

A freshly prepared solution of the requisite organometallic reagent R_2M (4.9 mmol, 1.4 equiv., 0.33 M in anhyd. THF) was cooled to –78 °C with stirring. Copper(I) iodide (0.6 mmol, 114 mg, 0.2 equiv.) was then added to form a brown suspension. The mixture was stirred for 15 min and

the epoxide **S1** (3.5 mmol, 1.0 equiv.) was added. The resulting mixture was allowed to warm to room temperature and stirred for another *ca*. 16 h (monitored by TLC). The reaction was quenched with saturated aqueous ammonium chloride solution and diluted with ether (20 mL) and water (20 mL). The biphasic mixture was stirred for 30 min and then separated, and the aqueous phase was extracted with diethyl ether (2x). The combined organic phases were dried (anhyd. $MgSO_4$), filtered, and concentrated *in vacuo* to afford the crude product **S3**, which was used for the next step without further purification.

To a stirred solution of crude alcohol **S3** (3.5 mmol) in anhydrous pyridine (5 mL) were added dimethylaminopyridine (DMAP, 42.7 mg, 0.35 mmol, 0.1 equiv.) and *p*-toluenesulfonyl chloride (934 mg, 4.9 mmol, 1.4 equiv.) at 0 °C. The resulting reaction mixture was allowed to warm to room temperature and stirred for *ca*. 16 h (monitored by TLC). The reaction was quenched with water (30 mL) and extracted with ethyl acetate (3x). The combined organic phases were dried (anhyd. $MgSO_4$), filtered, and concentrated *in vacuo* to afford the crude product. Purification by flash column chromatography (silica gel, eluting with 10-20% diethyl ether/hexane) afforded the tosylates **2a-2m** in 55–85% yield.

7. Spectral Data for the Secondary Tosylates 2a-2m

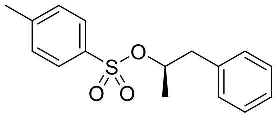
2a

(*R*)-1-Phenylpropan-2-yl-4-methylbenzenesulfonate (2a)

Color and State: white solid, m.p. = 63.6-65.1 °C.

The characterization data are identical to those reported.[44]

$[\alpha]_D^{25}$ –27.4 (*c* = 3.10, EtOH), lit. $[\alpha]_D$ –24.8 (*c* = 3.10, EtOH); (CHIRAL AD-H column) 99:0.7:0.3 hexane/isopropanol/methanol at 0.6 mL/min flow rate; tR (*S*)-enantiomer (minor) = 25.3 min., tR (*R*)-enantiomer (major) = 27.6 min.; ≥99% *ee*.

^{1}H NMR (500 MHz, $CDCl_3$) δ 7.63-7.61 (m, 2H), 7.22-7.19 (m, 5H), 7.05-7.03 (m, 2H), 4.75 (sextet, *J* = 6.4 Hz, 1H), 2.92 (dd, *J* = 13.9, 6.5 Hz, 1H), 2.78 (dd, *J* = 13.8, 6.4 Hz, 1H), 2.41 (s, 3H), 1.30 (d, *J* = 6.2 Hz, 3H).

IR (Neat) 3060 (w), 3034 (w), 2989 (w), 2937 (w), 2874 (w), 1595 (w), 1495 (w), 1454 (w), 1375 (w), 1338 (s), 1167 (s), 1126 (m), 1096 (m), 1070 (m), 1018 (m), 913 (s), 891 (s), 813 (m), 768 (s), 742 (s), 697 (s), 663 (s) cm^{-1}.

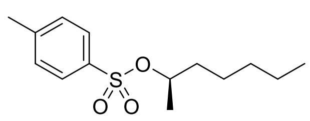

2b

(*R*)-Heptan-2-yl 4-methylbenzenesulfonate (2b)

Color and State: colorless oil

The characterization data are identical to those reported.[45]

$[\alpha]_D^{25}$ –3.9 (*c* = 1.30, $CHCl_3$), lit. for (*S*)-enantiomer $[\alpha]_D^{23}$ +6.6 (*c* = 1.30, $CHCl_3$); (CHIRALPAK AD-H column) 98:1:1 hexane/isopropanol/methanol at 0.6 mL/min flow rate; tR (*S*)-enantiomer (minor) = 13.8 min., tR (*R*)-enantiomer (major) = 14.6 min.; ≥98.5% *ee*.

^{1}H NMR (500 MHz, $CDCl_3$) δ 7.80-7.78 (m, 2H), 7.34-7.31 (m, 2H), 4.60 (sextet, *J* = 6.3 Hz, 1H), 2.44 (s, 3H), 1.63-1.56 (m, 1H), 1.50-1.43 (m, 1H), 1.26 (d, *J* = 6.3 Hz, 3H), 1.23-1.10 (m, 6H), 0.82 (t, *J* = 7.1 Hz, 3H).

IR (Neat) 2956 (w), 2930 (w), 2863 (w), 1599 (w), 1495 (w), 1457 (w), 1353 (m), 1174 (s), 1096 (m), 891 (s), 813 (m), 779 (m), 754 (m), 723 (m), 686 (m), 660 (s) cm^{-1}.

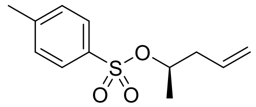

2c

(*R*)-Pent-4-en-2-yl-4-methylbenzenesulfonate (2c)

Color and State: yellow oil

The characterization data are identical with those reported.[46]

$[\alpha]_D^{20}$ +10.0 (*c* = 1.35, $CHCl_3$), lit. $[\alpha]_D^{20}$ +11.8 (*c* = 1.35, $CHCl_3$); (CHIRALPAK AD-H column) 99.5:0.5 hexane/methanol at 0.4 mL/min flow rate; tR (*S*)-enantiomer (minor) = 23.6 min., tR (*R*)-enantiomer (major) = 28.3 min.; ≥99.5% *ee*.

^{1}H NMR (500 MHz, $CDCl_3$) δ 7.80-7.78 (m, 2H), 7.34-7.32 (m, 2H), 5.60 (ddt, *J* = 17.0, 10.1, 7.0 Hz, 1H), 5.06-5.02 (m, 2H), 4.65 (sextet, *J* = 6.3 Hz, 1H), 2.45 (s, 3H), 2.38-2.26 (m, 2H), 1.26 (d, *J* = 6.2 Hz, 3H).

IR (Neat) 3079 (w), 3068 (w), 2982 (w), 2930 (w), 2878 (w), 2851 (w), 1644 (w), 1599 (w), 1495 (w), 1450 (w), 1353 (m), 1174 (s), 1096 (m), 902 (s), 857 (w), 813 (m), 783 (m), 760 (m), 690 (m), 663 (s) cm^{-1}.

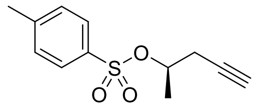

2d

(*R*)-Pent-4-yn-2-yl 4-methylbenzenesulfonate (2d)

Color and State: colorless oil

$[\alpha]_D^{20}$ +20.7 (c = 1.11, $CHCl_3$); (CHIRALCEL OJ-H column) 98:1:1 hexane/isopropanol/methanol at 0.6 mL/min flow rate; tR (*S*)-enantiomer (minor) = 37.4 min., tR (*R*)-enantiomer (major) = 40.0 min.; ≥98.5% *ee*.

^{1}H NMR (500 MHz, $CDCl_3$) δ 7.81-7.79 (m, 2H), 7.35-7.32 (m, 2H), 4.67 (sextet, J = 6.2 Hz, 1H), 2.52 (ddd, J = 16.8, 5.0, 2.7 Hz, 1H), 2.45 (ddd, J = 16.8, 7.2, 2.6 Hz, 1H), 2.44 (s, 3H), 1.96 (t, J = 2.7 Hz, 1H), 1.37 (d, J = 6.3 Hz, 3H).

^{13}C NMR (125 MHz, $CDCl_3$) δ 144.87 (e), 134.18 (e), 129.94 (o). 127.94 (o), 78.53 (e), 76.98 (o), 71.42 (o), 26.59 (e), 21.77 (o), 20.07 (o).

IR (Neat): 3288 (w), 2986 (w), 2933 (w), 2878 (w), 2125 (w), 1599 (w), 1495 (w), 1454 (w), 1349 (m), 1189 (m), 1174 (s), 1096 (m), 1036 (m), 906 (s), 850 (m), 813 (m), 757 (m), 663 (s) cm^{-1}.

HRMS (DART) calcd. for $C_{12}H_{15}O_3S$ $[M+H]^+$: 239.0736, found: 239.0736.

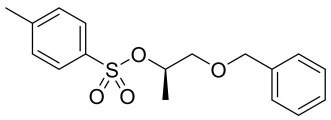

2e

(*R*)-1-(Benzyloxy)propan-2-yl-4-methylbenzenesulfonate (2e)

Recrystallized at -20 °C in hexane/ether after column chromatograph.

Color and State: colorless crystalline solid, m.p. = 33.0-33.6 °C.

The characterization data are identical to those reported.[47]

$[\alpha]_D^{25}$ +12.8 (c = 0.19, $CHCl_3$), lit. for (*S*)-enantiomer $[\alpha]_D^{25}$ –7.0 (c = 0.19, $CHCl_3$); (CHIRALPAK AD-H column) 98:1:1 hexane/isopropanol/methanol at 0.6 mL/min flow rate; tR (*S*)-enantiomer (minor) = 40.1 min., tR (*R*)-enantiomer (major) = 44.9 min.; ≥99.5% *ee*

^{1}H NMR (500 MHz, $CDCl_3$) δ 7.80-7.77 (m, 2H), 7.34-7.25 (m, 5H), 7.22-7.21 (m, 2H), 4.74 (pentd, J = 6.3, 4.6 Hz, 1H), 4.44 (d, A of an AB quartet, J_{AB} = 12.0 Hz, 1H), 4.41 (d, B of an AB quartet, J_{AB} = 12.0 Hz, 1H), 3.51 (dd, J = 10.7, 6.0 Hz, 1H), 3.44 (dd, J = 10.7, 4.4 Hz, 1H), 2.41 (s, 3H), 1.32 (d, J = 6.4 Hz, 3H).

IR (Neat) 3086 (w), 3064 (w), 3030 (w), 2982 (w), 2933 (w), 2866 (w), 2803 (w), 1599 (w), 1495 (w), 1454 (w), 1349 (m), 1174 (s), 1096 (m), 917 (s), 898 (s), 813 (m), 768 (m), 738 (s), 697 (m), 663 (s) cm^{-1}.

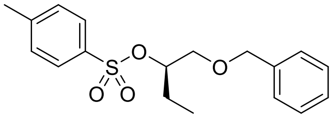

2f

(*R*)-1-(Benzyloxy)butan-2-yl-4-methylbenzenesulfonate (2f)

Color and State: colorless oil

$[\alpha]_D^{20}$ +14.7 (c = 0.85, $CHCl_3$); (CHIRALPAK AD-H column) 98:1:1 hexane/isopropanol/methanol at 0.6 mL/min flow rate; tR (*R*)-enantiomer (major) = 20.0 min., tR (*S*)-enantiomer (minor) = 21.5 min.; ≥99% *ee*.

^{1}H NMR (500 MHz, $CDCl_3$) δ 7.80-7.77 (m, 2H), 7.34-7.27 (m, 3H), 7.25-7.21 (m, 4H), 4.61 (tt, J = 6.1, 5.2 Hz, 1H), 4.44 (d, A of an AB quartet, J_{AB} = 12.0 Hz, 1H), 4.39 (d, B of an AB quartet, J_{AB} = 12.0 Hz, 1H), 3.54 (dd, J = 10.7, 5.3 Hz, 1H), 3.51 (dd, J = 10.7, 4.7 Hz, 1H), 2.41(s, 3H), 1.80-1.65 (m, 2H), 0.85 (t, J = 7.4 Hz, 3H).

^{13}C NMR (125 MHz, $CDCl_3$) δ 144.49 (e), 137.89 (e), 134.53 (e), 129.70 (o), 128.47 (o), 127.97 (o), 127.81 (o), 127.72 (o), 83.13 (o), 73.39 (e), 70.66 (e), 24.83 (e), 21.75 (o), 9.34 (o).

IR (Neat) 3064 (w), 3030 (w), 2971 (w), 2937 (w), 2922 (w), 2878 (w), 2866 (w), 2807 (w), 1599 (w), 1495 (w), 1454 (w), 1357 (m), 1174 (s), 1092 (m), 898 (s), 850 (m), 813 (s), 734 (s), 697 (s), 663 (s) cm^{-1}.

HRMS (DART) calcd. for $C_{18}H_{23}O_4S$ $[M+H]^+$: 335.1312, found: 335.1316.

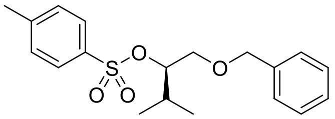

2g

(*R*)-1-(Benzyloxy)-3-methylbutan-2-yl-4-methylbenzenesulfonate (2g)

Color and State: colorless oil

$[\alpha]_D^{20}$ +7.0 (c = 1.07, $CHCl_3$); (CHIRALPAK AD-H column) 98:1:1 hexane/isopropanol/methanol at 0.6 mL/min flow rate; tR (*S*)-enantiomer (minor) = 29.7 min., tR (*R*)-enantiomer (major) = 33.5 min.; ≥99% *ee*.

^{1}H NMR (500 MHz, $CDCl_3$) δ 7.79-7.77 (m, 2H), 7.34-7.27 (m, 3H), 7.25-7.21 (m, 4H), 4.53 (q, J = 5.1 Hz, 1H), 4.41 (d, A of an AB quartet, J_{AB} = 11.9 Hz, 1H), 4.36 (d, B of an AB quartet, J_{AB} = 12.0 Hz, 1H), 3.56 (d, J = 4.9 Hz, 2H), 2.40 (s, 3H), 2.10 (octet, J = 6.7 Hz, 1H), 0.89 (d, J = 6.6 Hz, 3H), 0.88 (d, J = 6.7 Hz, 3H).

^{13}C NMR (125 MHz, $CDCl_3$) δ 144.39 (e), 137.90 (e), 134.68 (e), 129.62 (o), 128.45 (o), 127.96 (o), 127.78 (o), 127.72 (o), 86.54 (o), 73.36 (e), 69.31 (e), 29.66 (o), 21.73 (o), 18.46 (o), 17.62 (o)

IR (Neat) 3086 (w), 3064 (w), 3030 (w), 2967 (w), 2930 (w), 2900 (w), 2874 (w), 2814 (w), 1599 (w), 1495 (w), 1454 (w), 1357 (m), 1189 (m), 1174 (s), 1111 (m), 1092 (m), 902 (s), 842 (m), 813 (m), 779 (m), 734 (m), 697 (m), 667 (s) cm^{-1}.

HRMS (DART) calcd. for $C_{19}H_{25}O_4S$ $[M+H]^+$: 349.1468, found: 349.1477.

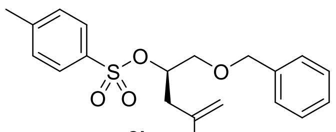
2h

(*R*)-1-(Benzyloxy)-4-methylpent-4-en-2-yl-4-methylbenzenesulfonate (2h)

Color and State: colorless oil

$[\alpha]_D^{20}$ +10.1 (*c* = 0.95, $CHCl_3$); (CHIRALPAK AD-H column) 98:1:1 hexane/isopropanol/methanol at 0.6 mL/min flow rate; tR (*S*)-enantiomer (minor) = 18.2 min., tR (*R*)-enantiomer (major) = 19.6 min.; ≥99.5% *ee*.

^{1}H NMR (500 MHz, $CDCl_3$) δ 7.79-7.77 (m, 2H), 7.34-7.22 (m, 7H), 4.80-4.77 (m, 1H), 4.76 (t, *J* = 1.6 Hz, 1H), 4.71 (q, *J* = 1.0 Hz, 1H), 4.45 (d, A of an AB quartet, J_{AB} = 12.0 Hz, 1H), 4.40 (d, B of an AB quartet, J_{AB} = 11.8 Hz, 1H), 3.55 (d, *J* = 4.7 Hz, 2H), 2.42-2.40 (m, 5H), 1.64 (s, 3H).

^{13}C NMR (125 MHz, $CDCl_3$) δ 144.52 (e), 140.09 (e), 137.87 (e), 134.42 (e), 129.63 (o), 128.45 (o), 128.05 (o), 127.79 (o), 127.72 (o), 114.77 (e), 79.99 (o), 73.39 (e), 70.74 (e), 40.08 (e), 22.49 (o), 21.73 (o).

IR (Neat) 3064 (w), 3030 (w), 2967 (w), 2919 (w), 2863 (w), 2803 (w), 1651 (w), 1599 (w), 1495 (w), 1454 (w), 1357 (m), 1174 (s), 1122 (m), 1096 (m), 895 (s), 813 (m), 772 (m), 738 (s), 697 (m), 663 (s) cm^{-1}.

HRMS (DART) calcd. for $C_{20}H_{25}O_4S$ $[M+H]^+$: 361.1468, found: 361.1478.

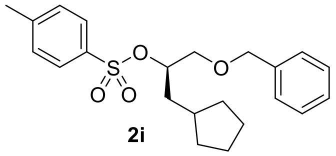
2i

(*R*)-1-(Benzyloxy)-3-cyclopentylpropan-2-yl-4-methylbenzenesulfonate (2i)

Color and State: colorless oil

$[\alpha]_D^{20}$ +19.4 (*c* = 1.23, $CHCl_3$); (CHIRALPAK AD-H column) 98:1:1 hexane/isopropanol/methanol at 0.6 mL/min flow rate; tR (*R*)-enantiomer (major) = 18.8 min., tR (*S*)-enantiomer (minor) = 20.2 min.; ≥99.5% *ee*.

^{1}H NMR (500 MHz, $CDCl_3$) δ 7.80-7.78 (m, 2H), 7.34-7.25 (m, 5H), 7.23-7.22 (m, 2H), 4.69-4.64 (m, 1H), 4.45 (d, A of an AB quartet, J_{AB} = 12.0 Hz), 4.39 (d, B of an AB quartet, J_{AB} = 12.0 Hz), 3.53 (d, J = 4.7 Hz, 2H), 2.41 (s, 3H), 1.79-1.67 (m, 3H), 1.65-1.51 (m, 4H), 1.49-1.37 (m, 2H), 1.08-0.93 (m, 2H).

^{13}C NMR (125 MHz, $CDCl_3$) δ 144.48 (e), 137.93 (e), 134.57 (e), 129.68 (o), 128.46 (o), 127.99 (o), 127.80 (o), 127.74 (o), 81.77 (o), 73.39 (e), 71.38 (e), 37.95 (e), 36.03 (o), 32.88 (e), 32.55 (e), 25.07 (e), 25.05 (e), 21.73 (o).

IR (Neat) 3086 (w), 3060 (w), 3030 (w), 2945 (w), 2863 (w), 1651 (w), 1599 (w), 1495 (w), 1450 (w), 1360 (m), 1174 (s), 1096 (m), 906 (s), 813 (m), 738 (m), 697 (m), 663 (s) cm^{-1}.

HRMS (DART) calcd. for $C_{22}H_{29}O_4S$ $[M+H]^+$: 389.1781, found: 389.1778.

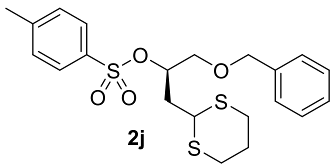
2j

(*R*)-1-(Benzyloxy)-3-(1,3-dithian-2-yl)propan-2-yl-4-methylbenzenesulfonate (2j)

Color and State: crystalline white solid, m.p.: 89.5-90.8 °C.

$[\alpha]_D^{20}$ +17.8 (c = 0.93, $CHCl_3$); (CHIRALPAK AD-H column) 86:7:7 hexane/isopropanol/methanol at 1 mL/min flow rate; tR (*R*)-enantiomer (major) = 15.7 min., tR (*S*)-enantiomer (minor) = 17.6 min.; ≥99.5% *ee*.

^{1}H NMR (500 MHz, $CDCl_3$) δ 7.82-7.80 (m, 2H), 7.35-7.25 (m, 7H), 4.91 (app. sextet, J = 4.3 Hz, 1H), 4.49 (d, A of an AB quartet, J_{AB} = 12.1 Hz, 1H), 4.42 (d, B of an AB quartet, J_{AB} = 12.1 Hz, 1H), 3.76 (dd, J = 9.2, 5.5 Hz, 1H), 3.58 (d, J = 4.6 Hz, 2H), 2.81-2.69 (m, 3H), 2.59 (ddd, J = 13.9, 11.2, 2.6 Hz, 1H), 2.43 (s, 3H), 2.16 (ddd, J = 14.7, 8.5, 6.0 Hz, 1H), 2.11 (ddd, J = 14.7, 9.6, 5.0 Hz, 1H), 2.03 (dtt, J = 14.1, 2.8 Hz, 1H), 1.83 (dtt, J = 13.9, 10.8, 3.0 Hz, 1H).

^{13}C NMR (125 MHz, $CDCl_3$) δ 144.78 (e), 137.74 (e), 134.15 (e), 129.85 (o), 128.54 (o), 128.16 (o), 127.91 (o), 127.86 (o), 78.22 (o), 73.46 (e), 70.81 (e), 42.58 (o), 37.35 (e), 29.85 (e), 29.48 (e), 25.82 (e), 21.80 (o).

IR (Neat) 3090 (w), 3056 (w), 3042 (w), 2978 (w), 2937 (w), 2889 (w), 2814 (w), 1595 (w), 1495 (w), 1450 (w), 1420 (w), 1357 (s), 1178 (s), 1118 (m), 1088 (s), 969 (s), 895 (s), 816 (s), 801 (m), 783 (m), 760 (s), 754 (s), 697 (s), 663 (s) cm^{-1}.

HRMS (DART) calcd. for $C_{21}H_{27}O_4S_3$ $[M+H]^+$: 439.1066, found: 439.1076.

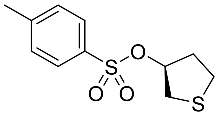

2k

(*S*)-Tetrahydrothiophen-3-yl-4-methylbenzenesulfonate (2k)

Color and State: colorless oil

$[\alpha]_D^{20}$ −12.7 (*c* = 0.92, $CHCl_3$); (CHIRALPAK AD-H column) 96:2:2 hexane/isopropanol/methanol at 1.0 mL/min flow rate; tR (*R*)-enantiomer (minor) = 30.9 min., tR (*S*)-enantiomer (major) = 40.4 min.; 99.5% *ee*.

^{1}H NMR (500 MHz, $CDCl_3$) δ 7.81-7.78 (m, 2H), 7.36-7.33 (m, 2H), 5.20 (pent, *J* = 3.8 Hz, 1H), 2.99 (dd, *J* = 12.4, 4.8 Hz, 1H), 2.96-2.90 (m, 2H), 2.85 (ddd, *J* = 10.6, 7.4, 3.4 Hz, 1H), 2.45 (s, 3H), 2.33-2.27 (m, 1H), 1.94 (dddd, *J* = 13.5, 9.6, 7.7, 3.8 Hz, 1H).

^{13}C NMR (125 MHz, $CDCl_3$) δ 145.06 (e), 134.10 (e), 130.06 (o), 127.83 (o), 83.77 (o), 36.67 (e), 36.61 (e), 28.15 (e), 21.77 (o).

IR (Neat) 3045 (w), 2937 (w), 2866 (w), 1595 (w), 1495 (w), 1450 (w), 1428 (w), 1353 (s), 1193 (m), 1170 (s), 1096 (m), 969 (m), 924 (s), 880 (s), 857 (s), 813 (s), 716 (s), 686 (m), 663 (s) cm^{-1}.

HRMS (DART) calcd. for $C_{11}H_{15}O_3S$ $[M+H]^+$: 259.0457, found: 259.0462.

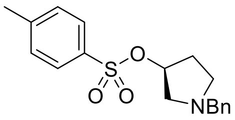

2l

(*S*)-Tetrahydrofuran-3-yl-4-methylbenzenesulfonate (2l)

Color and State: brown oil

The characterization data are identical with those reported.[48]

$[\alpha]_D^{20}$ –45.0 (*c* = 5.00, MeOH), lit. $[\alpha]_D$ –30.0 (*c* = 5.00, MeOH); (CHIRALPAK IA-H column) 96:2:2 hexane/isopropanol/methanol at 1.0 mL/min flow rate; tR (*R*)-enantiomer (major) = 17.7 min., tR (*S*)-enantiomer (minor) = 19.9 min.; ≥95.5% *ee*.

^{1}H NMR (500 MHz, $CDCl_3$) δ 7.78-7.75 (m, 2H), 7.32-7.23 (m, 7H), 4.98 (dq, *J* = 10.6, 3.0 Hz, 1H), 3.61 (d, A of an AB quartet, J_{AB} = 12.9 Hz, 1H), 3.55 (d, B of an AB quartet, J_{AB} = 12.8 Hz, 1H), 2.76 (dd, *J* = 11.2, 6.2 Hz, 1H), 2.68 (q, *J* = 7.8 Hz, 1H), 2.64 (dd, *J* = 11.3, 3.0 Hz, 1H), 2.49-2.44 (m, 1H), 2.44 (s, 3H), 2.13 (app. sextet, *J* = 7.3 Hz, 1H), 1.94 (dddd, *J* = 13.9, 8.0, 5.6, 2.4 Hz, 1H).

IR (Neat) 3060 (w), 3027 (w), 2956 (w), 2919 (w), 2866 (w), 2796 (w), 2743 (w), 1595 (w), 1495 (w), 1454 (w), 1357 (m), 1189 (m), 1170 (s), 1126 (m), 1096 (m), 1073 (m), 954 (m), 932 (m), 887 (s), 876 (s), 846 (m), 813 (s), 772 (m), 742 (m), 697 (s), 660 (s) cm^{-1}.

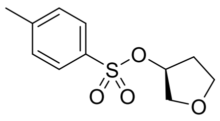
2m

(*S*)-Tetrahydrofuran-3-yl-4-methylbenzenesulfonate (2m)

Color and State: colorless oil

The characterization data are identical to those reported.[49]

$[\alpha]_D^{20}$ +0.9 (c = 1.13, $CHCl_3$); (CHIRALPAK AD-H column) 90:5:5 hexane/isopropanol/methanol at 1.0 mL/min flow rate; tR (*S*)-enantiomer (minor) = 40.5 min., tR (*R*)-enantiomer (major) = 56.6 min.; ≥99.5% *ee*.

^{1}H NMR (500 MHz, $CDCl_3$) δ 7.80-7.78 (m, 2H), 7.36-7.34 (m, 2H), 5.11 (app. septet, J = 2.4 Hz, 1H), 3.91-3.78 (m, 4H), 2.45 (s, 3H), 2.12-2.04 (m, 2H).

IR (Neat) 3060 (w), 3045 (w), 3027 (w), 2982 (w), 2956 (w), 2926 (w), 2870 (w), 1595 (w), 1495 (w), 1439 (w), 1357 (m), 1170 (s), 1092 (m), 1077 (m), 954 (s), 883 (s), 813 (s), 727 (m), 660 (s) cm^{-1}.

8. General Experimental Procedures for the Alkylation of Chiral Non-Racemic Secondary Tosylates with Cyanohydrins

Method A: Sodium bis(trimethylsilyl)amide (0.5 mL, 1.0 M in THF, 2.0 equiv.) was added dropwise to a stirred solution of the cyanohydrin (0.375 mmol, 1.5 equiv.) in anhydrous THF (2 mL) at room temperature. The resulting mixture was stirred for *ca*. 10 min to generate the corresponding anion solution. The requisite tosylate (0.25 mmol) was then added, and the reaction was stirred at room temperature for *ca*. 16 h (monitored by TLC). The reaction suspension was cooled to –40 °C, and tetrabutylammonium fluoride (1.0 mL, 1.0 M in THF, 4 equiv.) was added, giving a dark red, clear solution. The reaction was stirred at –40 °C for *ca.* 1 h and then quenched with saturated aqueous ammonium chloride solution. ***CAUTION:*** *This procedure involves the generation of highly toxic cyanides. All operations must be performed in a well-ventilated fume hood, and all cyanide-containing waste must be quenched correctly in accordance with established safety protocols.* The biphasic mixture was extracted with ethyl acetate, and the combined organic phases were dried (anhyd. $MgSO_4$), filtered, and concentrated *in vacuo* to afford the crude product.

Purification by flash column chromatography (silica gel, eluting with 5-10% diethyl ether/hexane) afforded the chiral ketones in 66–95% yield.

Method B: Sodium bis(trimethylsilyl)amide (0.5 mL, 1.0 M in THF, 2.0 equiv.) was added dropwise to a stirred solution of the cyanohydrin (0.375 mmol, 1.5 equiv.) in anhydrous THF (2 mL) at room temperature. The resulting mixture was stirred for *ca*. 10 min to generate the corresponding anion solution. The requisite tosylate (0.25 mmol) was then added, and the reaction was stirred at room temperature for *ca*. 16 h (monitored by TLC). The reaction suspension was cooled to –40 °C, quenched with HCl (0.2 mL, 2.5 M in diethyl ether, 2.0 equiv.) and stirred for *ca.* 10 min. ***CAUTION:*** *This procedure involves the generation of highly toxic hydrogen cyanide. All operations must be performed in a well-ventilated fume hood, and all cyanide-containing waste must be quenched correctly in accordance with established safety protocols.* Tetrabutylammonium fluoride solution (1.0 mL, 1.0 M in THF, 4 equiv.) was then added, giving a dark red, clear solution. The reaction was stirred at –40 °C for *ca*. 1 h and quenched with saturated aqueous ammonium chloride solution. The biphasic mixture was extracted with ethyl acetate, and the combined organic layers were dried (anhyd. $MgSO_4$), filtered, and concentrated *in vacuo* to afford the crude product. Purification by flash column chromatography (silica gel, eluting with 5-10% diethyl ether/hexane) afforded the chiral ketones.

Method C: Sodium bis(trimethylsilyl)amide (0.5 mL, 1.0 M in THF, 2.0 equiv.) was added to a stirred solution of the cyanohydrin (0.375 mmol, 1.5 equiv.) in anhydrous THF (2 mL) at room temperature. The mixture was stirred for *ca*. 10 min to generate the corresponding anion solution. The requisite tosylate (0.25 mmol) was then added, and the reaction was stirred at room temperature for *ca*. 16 h (monitored by TLC). The reaction was quenched with saturated aqueous ammonium chloride solution and extracted with ethyl acetate. The combined organic layers were dried (anhyd. $MgSO_4$), filtered, and concentrated *in vacuo* to afford the crude product. Purification by flash column chromatography afforded the cyanohydrin intermediate as colorless oil.

The cyanohydrin intermediate was dissolved in anhydrous THF (2 mL), and the resulting solution was cooled to –40 °C. Tetrabutylammonium fluoride (1.0 mL, 1.0 M in THF, 4 equiv.) was added, generating a clear reddish-yellow solution that was stirred at –40 °C for *ca*. 1 h before being quenched with saturated ammonium chloride solution. ***CAUTION:*** *This procedure involves the generation of highly toxic hydrogen cyanide. All operations must be performed in a well-ventilated*

fume hood, and all cyanide-containing waste must be quenched correctly in accordance with established safety protocols. The biphasic mixture was extracted with ethyl acetate, and the combined organic phases were dried (anhyd. $MgSO_4$), filtered, and concentrated *in vacuo* to afford the crude product. Purification by flash column chromatography (silica gel, eluting with 5-10% diethyl ether/hexane) afforded the chiral ketones.

9. Spectral Data for the Enantioenriched *α*-Tertiary Ketones 3aa-3cm

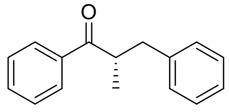
3aa

(*S*)-2-Methyl-1,3-diphenylpropan-1-one (3aa)

Color and State: colorless oil

The characterization data are identical to those reported.[34]

$[\alpha]_D^{20}$ +91.3 (c = 0.84, $CHCl_3$), lit. for (*R*)-enantiomer $[\alpha]_D^{20}$ -71.7 (c = 0.84, $CHCl_3$); (CHIRALPAK AD-H column) 99:1hexane/isopropanol at 0.6 mL/min flow rate; tR (*R*)-enantiomer (minor) = 11.8 min., tR = (*S*)-enantiomer (major) = 12.6 min.; ≥99.5% *ee* (100% *cee*) using Method A.

H NMR (500 MHz, $CDCl_3$) δ 7.94-7.91 (m, 2H), 7.56-7.53 (m, 1H), 7.47-7.43 (m, 2H), 7.28-7.25 (m, 2H), 7.21-7.16 (m. 3H), 3.75 (sextet, *J* = 7.0 Hz, 1H), 3.17 (dd, *J* = 13.8, 6.3 Hz, 1H), 2.70 (dd, *J* = 13.7, 7.9 Hz, 1H), 1.21 (d, *J* = 6.9 Hz, 3H).

IR (Neat) 3082 (w), 3061 (w), 3026 (w), 2969 (w), 2927 (w), 2870 (w), 1679 (s), 1595 (m), 1578 (w), 1493 (w), 1448 (m), 1370 (w), 1228 (m), 1192 (m), 1178 (m), 1078 (m), 972 (s), 744 (m), 731 (m), 697 (vs) cm^{-1}.

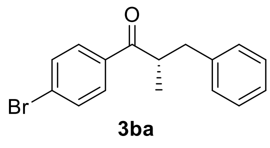
3ba

(*S*)-1-(4-Bromophenyl)-2-methyl-3-phenylpropan-1-one (3ba)

Color and State: yellow oil

$[\alpha]_D^{20}$ +46.3 (*c* = 1.26, $CHCl_3$); (CHIRALPAK IA-H column) 99:1 hexane/isopropanol at 0.6 mL/min flow rate; tR (*S*)-enantiomer (major) = 14.9 min., tR (*R*)-enantiomer (minor) = 16.7 min.; 87% *ee* (88% *cee*) using Method A.

^{1}H NMR (500 MHz, $CDCl_3$) δ 7.78-7.75 (m, 2H), 7.59-7.56 (m, 2H), 7.27-7.24 (m, 2H), 7.20-7.17 (m, 3H), 3.68 (sextet, *J* = 7.0 Hz, 1H), 3.14 (dd, *J* = 13.8, 6.6 Hz, 1H), 2.70 (dd, *J* = 13.7, 7.6 Hz, 1H), 1.20 (d, *J* = 6.8 Hz, 3H).

^{13}C NMR (125 MHz, $CDCl_3$) δ 202.88 (e), 139.86 (e), 135.40 (e), 132.08 (o), 129.95 (o), 129.18 (o), 128.58 (o), 128.20 (e), 126.45 (o), 42.98 (o), 39.57 (e), 17.58 (o).

IR (Neat) 3083 (w), 3060 (w), 3027 (w), 2963 (w), 2930 (w), 2874 (w), 2855 (w), 1681 (s), 1580 (s), 1565 (m), 1483 (w), 1454 (m), 1394 (m), 1223 (m), 1189 (m), 1174 (m), 1070 (s), 1010 (m), 969 (s), 835 (s), 779 (m), 745 (s), 697 (s) cm^{-1}.

HRMS (DART) calcd. for $C_{16}H_{16}{}^{79}BrO$ $[M+H]^+$: 303.0379, found: 303.0379.

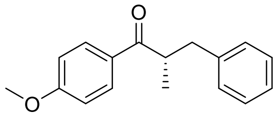

3ca

(*S*)-1-(4-Methoxyphenyl)-2-methyl-3-phenylpropan-1-one (3ca)

Color and State: colorless oil

$[\alpha]_D^{20}$ +47.4 (*c* = 0.72, $CHCl_3$); (CHIRALPAK AS-H column) 99:0.5:0.5 hexane/isopropanol/methanol at 0.4 mL/min flow rate; tR (*S*)-enantiomer (major) = 31.3 min., tR (*R*)-enantiomer (minor) = 35.8min.; ≥99% *ee* (100% *cee*) using Method A.

^{1}H NMR (500 MHz, $CDCl_3$) δ 7.93-7.91 (m, 2H), 7.29-7.24 (m, 2H), 7.21-7.16 (m, 3H), 6.93-6.91 (2H), 3.86 (s, 3H), 3.70 (sextet, *J* = 6.9 Hz, 1H), 3.15 (dd, *J* = 13.7, 6.2 Hz, 1H), 2.68 (dd, *J* = 13.7, 7.9 Hz, 1H), 1.19 (d, *J* = 6.9 Hz, 3H).

^{13}C NMR (125 MHz, $CDCl_3$) δ 202.39 (e), 163.52 (e), 140.29 (e), 130.70 (o), 129.58 (e), 129.23 (o), 128.48 (o), 126.27 (o), 113.92 (o), 55.59 (o), 42.49 (o), 39.66 (e), 17.70 (o).

IR (Neat) 3060 (w), 3027 (w), 3004 (w), 2967 (w), 2933 (w), 2874 (w), 2840 (w), 1670 (s), 1595 (s), 1573 (m), 1510 (m), 1454 (m), 1416 (m), 1308 (m), 1256 (s), 1230 (m), 1167 (s), 1114 (m), 1029 (m), 973 (s), 839 (m), 760 (m), 745 (m), 727 (m), 697 (s) cm^{-1}.

HRMS (DART) calcd. for $C_{17}H_{19}O_2$ $[M+H]^+$: 255.1380, found: 255.1379.

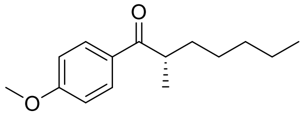

3cb

(*S*)-1-(4-Methoxyphenyl)-2-methylheptan-1-one (3cb)

Color and State: colorless oil

$[\alpha]_D^{20}$ +24.9 (*c* = 0.82, $CHCl_3$); (CHIRALPAK AS-H column) 99:0.5:0.5 hexane/isopropanol/methanol at 0.4 mL/min flow rate; tR (*S*)-enantiomer (major) = 20.0 min., tR (*R*)-enantiomer (minor) = 21.5 min.; ≥99% *ee* (100% *cee*) using Method A.

^{1}H NMR (500 MHz, $CDCl_3$) δ 7.96-7.93 (m, 2H), 6.95-6.92 (m, 2H), 3.87 (s, 3H), 3.42 (sextet, *J* = 6.7 Hz, 1H), 1.81-1.74 (m, 1H), 1.45-1.37 (m, 1H), 1.33-1.22 (m, 6H), 1.17 (d, *J* = 6.8 Hz, 3H), 0.86 (t, *J* = 6.7 Hz, 3H).

^{13}C NMR (125 MHz, $CDCl_3$) δ 203.28 (e). 163.43 (e), 130.63 (o), 129.94 (e), 113.87 (o), 55.58 (o), 40.32 (o), 34.02 (e), 32.09 (e), 27.27 (e), 22.67 (e), 17.54 (o), 14.15 (o).

IR (Neat) 2956 (m), 2930 (m), 2855 (w), 1674 (m), 1599 (s), 1573 (m), 1510 (m), 1461 (m), 1308 (m), 1256 (s), 1234 (s), 1170 (s), 1114 (m), 1029 (m), 973 (m), 839 (s), 809 (m) 764 (m), 693 (w) cm^{-1}.

HRMS (DART) calcd. for $C_{15}H_{23}O_2$ $[M+H]^+$: 235.1693, found: 235.2692.

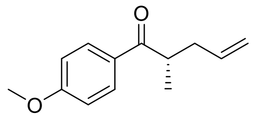

3cc

(*S*)-1-(4-Methoxyphenyl)-2-methylpent-4-en-1-one (3cc)

Color and State: colorless oil

$[\alpha]_D^{20}$ +37.1 (*c* = 0.25, $CHCl_3$); (CHIRALPAK AS-H column) 99:0.5:0.5 hexane/isopropanol/methanol at 0.4 mL/min flow rate; tR (*S*)-enantiomer (major) = 23.7 min., tR (*R*)-enantiomer (minor) = 27.2 min.; ≥96% *ee* (97% *cee*) using method A.

^{1}H NMR (500 MHz, $CDCl_3$) δ 7.96-7.93 (m, 2H), 6.96-6.93 (m, 2H), 5.78 (ddt, *J* = 17.0, 10.1, 7.0 Hz, 1H), 5.05 (app. dq, *J* = 17.1, 1.6 Hz, 1H), 5.00 (ddt, *J* = 10.2, 1.9, 1.0 Hz, 1H), 3.87 (s, 3H), 3.49 (sextet, *J* = 6.9 Hz, 1H), 2.55 (app. dtt, *J* = 14.1, 6.4, 1.3 Hz, 1H), 2.19 (app. dt, *J* = 14.4, 7.3 Hz, 1H), 1.20 (d, *J* = 6.9 Hz, 3H).

^{13}C NMR (125 MHz, $CDCl_3$) δ 202.31 (e), 163.54 (e), 136.20 (o), 130.72 (o), 129.62 (e), 116.71 (e), 113.95 (o), 55.61 (o), 40.20 (o), 37.95 (e), 17.34 (o).

IR (Neat) 3075 (w), 3004 (w), 2971 (w), 2933 (w), 2878 (w), 2840 (w), 1674 (s), 1640 (w), 1599 (s), 1573 (w), 1510 (m), 1457 (w), 1420 (w), 1260 (m), 1241 (s), 1211 (m), 1170 (s), 1032 (m), 977 (m), 842 (m) cm^{-1}.

HRMS (DART) calcd. for $C_{13}H_{17}O_2$ $[M+H]^+$: 205.1223, found: 205.1222.

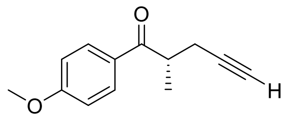

3cd

(*S*)-1-(4-Methoxyphenyl)-2-methylpent-4-yn-1-one (3cd)

Color and State: colorless oil

$[\alpha]_D^{20}$ +39.2 (c = 0.88, $CHCl_3$); (CHIRALPAK AD-H column) 99:0.5:0.5 hexane/isopropanol/methanol at 0.4 mL/min flow rate; tR (*S*)-enantiomer (major) = 56.2 min., tR (*R*)-enantiomer (minor) = 61.6 min.; ≥99.5% *ee* (100% *cee*) using Method A.

^{1}H NMR (500 MHz, $CDCl_3$) δ 7.97-7.94 (m, 2H), 6.96-6.94 (m, 2H), 3.87 (s, 3H), 3.63 (sextet, J = 7.0 Hz, 1H), 2.60 (ddd, J = 16.9, 5.9, 2.6 Hz, 1H), 2.39 (ddd, J = 16.9, 8.1, 2.6 Hz, 1H), 1.97 (t, J = 2.7 Hz, 1H), 1.30 (d, J = 7.0 Hz, 3H).

^{13}C NMR (125 MHz, $CDCl_3$) δ 202.86 (e), 163.77 (e), 130.84 (o), 129.07 (e), 114.04 (o), 82.70 (e), 69.69 (o), 55.63 (o), 40.08 (o), 22.49 (e), 17.77 (o).

IR (Neat) 3291 (w), 3068 (w), 3001 (w), 2971 (w), 2933 (w), 2878 (w), 2840 (w), 2117 (w), 1670 (m), 1595 (s), 1573 (m), 1510 (m), 1457 (m), 1420 (m), 1357 (w), 1308 (m), 1256 (m), 1234 (s), 1200 (m), 1167 (s), 1118 (m), 1025 (m), 977 (s), 839 (s), 801 (m), 760 (m), 690 (m) cm^{-1}.

HRMS (DART) calcd. for $C_{13}H_{15}O_2$ $[M+H]^+$: 203.1067, found: 203.1071.

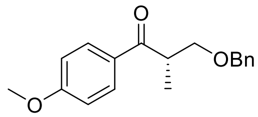

3ce

(*S*)-3-(Benzyloxy)-1-(4-methoxyphenyl)-2-methylpropan-1-one (3ce)

Color and State: colorless oil

$[\alpha]_D^{20}$ +65 (c = 1.40, $CHCl_3$); (CHIRALPAK AD-H column) 99:0.7:0.3 hexane/isopropanol/methanol at 1.0 mL/min flow rate; tR (*S*)-enantiomer (major) = 31.4 min., tR (*R*)-enantiomer (minor) = 36.1 min.; 99.5% *ee* (≥99.5% *cee*) using Method A.

^{1}H NMR (500 MHz, $CDCl_3$) δ 7.99-7.96 (m, 2H), 7.33-7.24 (m, 5H), 6.95-6.92 (m, 2H), 4.53 (d, A of an AB quartet, J_{AB} = 12.1 Hz, 1H), 4.60 (d, B of an AB quartet, J_{AB} = 12.1 Hz, 1H), 3.87 (s, 3H), 3.84 (dd, J = 8.6, 7.1 Hz, 1H), 3.78 (sextet, J = 6.6 Hz, 1H), 3.54 (dd, J = 8.7, 5.9 Hz, 1H), 1.21 (d, J = 6.9 Hz, 3H).

^{13}C NMR (125 MHz, $CDCl_3$) δ 201.38 (e), 163.61 (e), 138.43 (e), 130.85 (o), 129.85 (e), 128.45 (o), 127.69 (o), 127.65 (o), 113.88 (o), 73.47 (e), 72.86 (e), 55.60 (o), 41.18 (o), 15.21 (o).

IR (Neat) 3060 (w), 3030 (w), 3004 (w), 2960 (w), 2933 (w), 2900 (w), 2855 (w), 1670 (m), 1595 (s), 1573 (m), 1510 (m), 1454 (m), 1420 (m), 1364 (m), 1308 (m), 1252 (s), 1215 (s), 1170 (s), 1096 (s), 1029 (s), 973 (s), 839 (s), 809 (m), 734 (s), 697 (s) cm^{-1}.

HRMS (DART) calcd. for $C_{18}H_{21}O_3$ $[M+H]^+$: 285.1485, found: 285.1488.

(*S*)-2-((Benzyloxy)methyl)-1-(4-methoxyphenyl)butan-1-one (3cf)

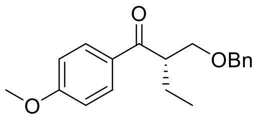

3cf

Color and State: colorless oil

$[\alpha]_D^{20}$ −17.2 (*c* = 1.09, $CHCl_3$); (CHIRALCEL OJ-H column) 98:1:1 hexane/isopropanol/methanol at 1.0 mL/min flow rate; tR (*R*)-enantiomer (minor) = 40.5 min., tR (*S*)-enantiomer (major) = 44.7 min.; 99% *ee* (≥99.5% *cee*) using Method A.

^{1}H NMR (500 MHz, $CDCl_3$) δ 8.00-7.97 (m, 2H), 7.31-7.28 (m, 2H), 7.26-7.23 (m, 3H), 6.96-6.93 (m, 2H), 4.50 (d, A of an AB quartet, J_{AB} = 12.1 Hz, 1H), 4.46 (d, B of an AB quartet, J_{AB} = 12.1 Hz, 1H), 3.87 (s, 3H), 3.79 (app. t, *J* = 8.1 Hz, 1 H), 3.69 (tt, *J* = 7.1, 5.9 Hz, 1H), 3.60 (dd, *J* = 8.6, 5.8 Hz, 1H), 1.78 (app. septet, *J* = 7.3 Hz, 1H), 1.67-1.59 (m, 1H), 0.87 (t, *J* = 7.4 Hz, 3H). **^{13}C NMR** (125 MHz, $CDCl_3$) δ 201.51 (e), 163.60 (e), 138.45 (e), 130.99 (e), 130.81 (o), 128.43 (o), 127.64 (o), 127.62 (o), 113.85 (o), 73.46 (e), 71.74 (e), 55.60 (o), 48.04 (o), 23.14 (e), 11.86 (o).

IR (Neat) 3060 (w), 3030 (w), 3004 (w), 2963 (w), 2930 (w), 2859 (m), 1670 (m), 1595 (s), 1573 (m), 1510 (m), 1454 (m), 1420 (m), 1364 (m), 1308 (m), 1256 (s), 1215 (s), 1170 (s), 1092 (s), 1025 (s), 962 (m), 839 (m), 813 (m), 734 (s), 697 (s) cm^{-1}.

HRMS (DART) calcd. for $C_{19}H_{23}O_3$ $[M+H]^+$: 299.1642, found: 299.1634.

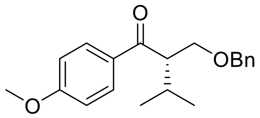

3cg

(*S*)-2-((Benzyloxy)methyl)-1-(4-methoxyphenyl)-3-methylbutan-1-one (3cg)

Color and State: colorless oil

$[\alpha]_D^{20}$ −39.4 (*c* = 1.13, $CHCl_3$); (CHIRALPAK AS-H column) 99:0.7:0.3 hexane/isopropanol/methanol at 0.6 mL/min flow rate; tR (*S*)-enantiomer (major) = 19.2 min., tR (*R*)-enantiomer (minor) = 22.9 min.; ≥99.5% *ee* (100% *cee*) using Method A.

^{1}H NMR (500 MHz, $CDCl_3$) δ 8.00-7.97 (m, 2H), 7.30-7.23 (m, 3H), 7.21-7.20 (m, 2H), 6.95-6.93 (m, 2H), 4.48 (d, A of an AB quartet, J_{AB} = 12.1 Hz, 1H), 4.44 (d, B of an AB quartet, J_{AB} = 12.1 Hz, 1H), 3.87 (s, 3H), 3.86 (app. t, *J* = 8.8 Hz, 1H), 3.71 (dd, *J* = 8.9, 4.6 Hz, 1H), 3.57 (ddd,

J = 8.4, 7.3, 4.6 Hz, 1H), 2.07 (octet, J = 6.9 Hz, 1H), 0.93 (d, J = 6.8 Hz, 3H), 0.92 (d, J = 6.7 Hz, 3H).

^{13}C NMR (125 MHz, $CDCl_3$) δ 201.93 (e), 163.49 (e), 138.52 (e), 131.79 (e), 130.81 (o), 128.39 (o), 127.53 (o), 113.81 (o), 73.46 (e), 70.64 (e), 55.59 (o), 52.65 (o), 29.37 (o), 21.43 (o), 20.30 (o).

IR (Neat) 3060 (w), 3030 (w), 3004 (w), 2960 (m), 2930 (w), 2870 (w), 2840 (w), 1670 (m), 1599 (s), 1573 (m), 1510 (w), 1454 (w), 1256 (s), 1215 (m), 1170 (s), 1100 (m), 1029 (m), 734 (m), 697 (m) cm^{-1}.

HRMS (DART) calcd. for $C_{20}H_{25}O_3$ $[M+H]^+$: 313.1798, found: 313.1807.

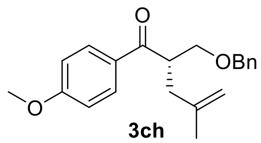

3ch

(*S*)-2-((Benzyloxy)methyl)-1-(4-methoxyphenyl)-4-methylpent-4-en-1-one (3ch)

Color and State: colorless oil

$[\alpha]_D^{20}$ −10.7 (c = 0.72, $CHCl_3$); (CHIRALPAK AD-H column) 98:1:1 hexane/isopropanol/methanol at 1.0 mL/min flow rate; tR (*S*)-enantiomer (major) = 8.6 min., tR (*R*)-enantiomer (minor) = 10.0 min.; 97% *ee* (≥97% *cee*) using Method A.

^{1}H NMR (500 MHz, $CDCl_3$) δ 8.01-7.98 (m, 2H), 7.31-7.24 (m, 3H), 7.22-7.21 (m, 2H), 6.96-6.93 (m, 2H), 4.71 (t, J = 1.7 Hz, 1H), 4.67 (q, J = 1.0 Hz, 1H), 4.48 (d, A of an AB quartet, J_{AB} = 12.1 Hz, 1H), 4.45 (d, B of an AB quartet, J_{AB} = 12.1 Hz, 1H), 3.95 (tdd, J = 7.6, 6.5, 5.4 Hz, 1H), 3.87 (s, 3H), 3.78 (dd, J = 8.9, 8.0 Hz, 1H), 3.59 (dd, J = 9.0, 5.2 Hz, 1H), 2.47 (dd, J = 14.6, 7.5 Hz, 1H), 2.26 (dd, J = 14.5, 6.5 Hz, 1H), 1.72 (s, 3H).

^{13}C NMR (125 MHz, $CDCl_3$) δ 200.84 (e), 163.60 (e), 142.86 (e), 138.36 (e), 130.82 (o), 130.78 (e), 128.40 (o), 127.60 (o), 127.56 (o), 113.86 (o), 112.37 (e), 73.42 (e), 71.94 (e), 55.58 (o), 44.88 (o), 37.65 (e), 22.85 (o).

IR (Neat) (s) 3064 (w), 3027 (w), 3004 (w), 2963 (w), 2933 (w), 2904 (w), 2855 (w), 2840 (w), 1670 (m), 1599 (s), 1573 (m), 1510 (m), 1454 (m), 1420 (m), 1364 (m), 1308 (m), 1252 (s), 1215 (m), 1167 (s), 1092 (s), 1029 (s), 950 (m), 891 (m), 839 (m), 805 (m), 734 (s), 697 (s) cm^{-1}.

HRMS (DART) calcd. for $C_{21}H_{25}O_3$ $[M+H]^+$: 325.1798, found: 325.1800.

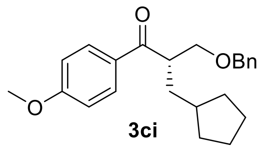

(*S*)-3-(Benzyloxy)-2-(cyclopentylmethyl)-1-(4-methoxyphenyl)propan-1-one (3ci)

Color and State: colorless oil

$[\alpha]_D^{20}$ −5.2 (*c* = 1.01, $CHCl_3$); (CHIRALPAK AD-H column) 99:0.7:0.3 hexane/isopropanol/methanol at 1.0 mL/min flow rate; tR (*R*)-enantiomer (minor) = 34.1 min., tR (*S*)-enantiomer (major) = 48.7 min.; ≥99% *ee* (99.5% *cee*) using Method A.

^{1}H NMR (500 MHz, $CDCl_3$) δ 8.01-7.98 (m, 2H), 7.30-7.20 (m, 5H), 6.96-6.93 (m, 2H), 4.48 (d, A of an AB quartet, J_{AB} = 12.2 Hz, 1H), 4.44 (d, B of an AB quartet, J_{AB} = 12.2 Hz, 1H), 3.88 (s, 3H), 3.84-3.79 (m, 1H), 3.76 (t, *J* = 8.4 Hz, 1H), 3.58 (dd, *J* = 8.6, 5.1Hz, 1H), 1.83-1.76 (m, 1H), 1.75-1.66 (m, 3H), 1.60-1.39 (m, 5H), 1.12-1.01 (m, 2H).

^{13}C NMR (125 MHz, $CDCl_3$) δ 202.01 (e), 163.60 (e), 138.45 (e), 131.12 (e), 130.83 (o), 128.41 (o), 127.57 (o), 113.87 (o), 73.41 (e), 72.74 (e), 55.60 (o), 45.92 (o), 38.28 (o), 36.41 (e), 33.35 (e), 32.92 (e), 25.28 (e), 25.24 (e).

IR (Neat) 3064 (w), 3030 (w), 3004 (w), 2945 (m), 2859 (w), 1718 (w), 1703 (w), 1670 (m), 1599 (s), 1573 (w), 1510 (m), 1454 (m), 1360 (m), 1308 (m), 1252 (s), 1215 (m), 1170 (s), 1096 (s), 1029 (m), 985 (m), 906 (m), 906 (m), 839 (m), 813 (m), 734 (m), 697 (s), 663 (m) cm^{-1}.

HRMS (DART) calcd. for $C_{23}H_{29}O_3$ $[M+H]^+$: 353.2111, found: 353.2120.

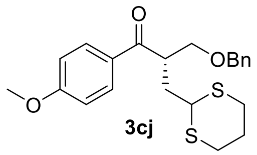

(*S*)-2-((1,3-Dithian-2-yl)methyl)-3-(benzyloxy)-1-(4-methoxyphenyl)propan-1-one (3cj)

Color and State: yellow oil

$[\alpha]_D^{20}$ +9.3 (*c* = 1.09, $CHCl_3$); (CHIRALPAK AD-H column) 86:7:7 hexane/isopropanol/methanol at 1.0 mL/min flow rate; tR (*S*)-enantiomer (major) = 17.4 min., tR (*R*)-enantiomer (minor) = 37.3 min.; ≥99% *ee* (≥99% *cee*) using Method A.

^{1}H NMR (500 MHz, $CDCl_3$) δ 8.04-8.01 (m, 2H), 7.31-7.24 (m, 3H), 7.23-7.21 (m, 2H), 6.95-6.92 (m, 2H), 4.52 (d, A of an AB quartet, J_{AB} = 12.7 Hz, 1H), 4.45 (d, B of an AB quartet, J_{AB} = 13.0 Hz, 1H), 4.11 (app. ddt, *J* = 11.7, 8.2, 6.3 Hz, 1H), 3.96 (dd, *J* = 8.0, 7.0 Hz, 1H), 3.87 (s, 3H), 3.72 (dd, *J* = 9.4, 6.6 Hz, 1H), 3.58 (dd, *J* = 9.2, 6.3 Hz, 1H), 2.82-2.75 (m, 3H), 2.71 (ddd, *J* =

13.9, 11.2, 2.6 Hz, 1H), 2.38 (ddd, J = 14.4, 7.8, 6.8 Hz, 1H), 2.08-2.00 (m, 2H), 1.85 (dtt, J = 13.9, 10.5, 3.5 Hz, 1H).

^{13}C NMR (125 MHz, $CDCl_3$) δ 199.76 (e), 163.75 (e), 138.13 (e), 131.08 (o), 130.31 (e), 128.43 (o), 127.67 (o), 127.60 (o), 113.90 (o), 73.30 (e), 71.93 (e), 55.60 (o), 45.26 (o), 43.78 (o), 35.00 (e), 29.93 (e), 29.80 (e), 25.97 (e).

IR (Neat) 3056 (w), 3030 (w), 3004 (w), 2933 (w), 2896 (w), 2855 (w), 2840 (w), 1666 (m), 1595 (s), 1573 (m), 1510 (m), 1454 (w), 1420 (m), 1360 (m), 1256 (s), 1167 (s), 1092 (s), 1025 (m), 954 (m), 906 (m), 839 (m), 816 (m), 734 (s), 697 (s), 663 (w) cm^{-1}.

HRMS (DART) calcd. for $C_{22}H_{27}O_3S_2$ $[M+H]^+$: 403.1396, found: 403.1402.

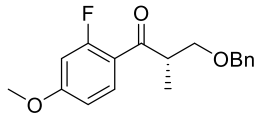

3de

(*S*)-3-(Benzyloxy)-1-(2-fluoro-4-methoxyphenyl)-2-methylpropan-1-one (3de)

Color and State: colorless oil

$[\alpha]_D^{20}$ −4.4 (c = 1.15, $CHCl_3$); (CHIRALPAK AD-H column) 99:0.5:0.5 hexane/isopropanol/methanol at 0.4 mL/min flow rate; tR (*S*)-enantiomer (major) = 35.6 min., tR (*R*)-enantiomer (minor) = 37.9 min.; 97% *ee* (≥97% *cee*) using Method A.

^{1}H NMR (500 MHz, $CDCl_3$) δ 7.84 (t, J = 8.7 Hz, 1H), 7.32-7.24 (m, 5H), 6.75 (dd, J = 8.8, 1.7 Hz, 1H), 6.60 (dd, J = 12.3, 1.4 Hz, 1H), 4.53 (d, A of an AB quartet, J_{AB} = 12.1 Hz, 1H), 4.47 (d, B of an AB quartet, J_{AB} = 12.1 Hz, 1H), 3.86 (s, 3H), 3.85-3.83 (m, 1H), 3.68 (sextet, J = 6.7 Hz, 1H), 3.53 (dd, J = 8.6, 6.5 Hz, 1H),1.22 (d, J = 6.9 Hz, 3H).

^{13}C NMR (125 MHz, $CDCl_3$) δ 199.77 (e, d, $^3J_{C\text{-}F}$ = 4.6 Hz), 164.74 (e, d, $^3J_{C\text{-}F}$ = 12.0 Hz), 163.21 (e, d, $^1J_{C\text{-}F}$ = 254.6 Hz), 138.50 (e), 132.67 (o, d, $^3J_{C\text{-}F}$ = 4.6 Hz), 128.43 (o), 127.67 (o), 127.61 (o), 118.74 (e, d, $^2J_{C\text{-}F}$ = 6.5 Hz), 110.91 (o, d, $^4J_{C\text{-}F}$ = 2.8 Hz), 101.88 (o, d, $^2J_{C\text{-}F}$ = 28.5 Hz), 73.37 (e), 72.43 (e), 55.97 (o), 45.71 (o, d, $^4J_{C\text{-}F}$ = 7.3 Hz), 14.42 (o).

^{19}F NMR (471 MHz, $CDCl_3$) δ –107.40.

IR (Neat) 3086 (w), 3060 (w), 3027 (w), 2971 (w), 2933 (w), 2855 (w), 2796 (w), 1674 (m), 1610 (s), 1573 (m), 1498 (m), 1454 (m), 1442 (m), 1364 (m), 1338 (m), 1290 (m), 1275 (m), 1234 (s), 1208 (m), 1155 (m), 1092 (s), 1029 (m), 973 (m), 950 (m), 839 (m), 734 (s), 697 (s) cm^{-1}.

HRMS (DART) calcd. for $C_{18}H_{20}FO_3$ $[M+H]^+$: 303.1391, found: 303.1392.

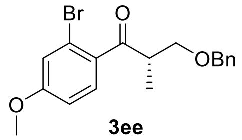

(*S*)-3-(Benzyloxy)-1-(2-bromo-4-methoxyphenyl)-2-methylpropan-1-one (3ee)

Color and State: colorless oil

$[\alpha]_D^{20}$ +4.4 (c = 0.80, $CHCl_3$); (CHIRALPAK AD-H column) 98:1:1 hexane/isopropanol/methanol at 1.0 mL/min flow rate; tR (*S*)-enantiomer (major) = 16.8 min., tR (*R*)-enantiomer (minor) 19.2 min.; ≥98% *ee* (≥98% *cee*) using Method B.

^{1}H NMR (500 MHz, $CDCl_3$) δ 7.45 (d, J = 8.6 Hz, 1H), 7.33-7.25 (m, 5H), 7.14 (d, J = 2.4 Hz, 1H), 6.85 (dd, J = 8.6, 2.4 Hz, 1H), 4.50 (d, A of an AB quartet, J_{AB} = 12.1 Hz, 1H), 4.46 (d, B of an AB quartet, J_{AB} = 12.0 Hz, 1H), 3.83 (s, 3H), 3.77 (dd, J = 8.9, 7.1 Hz, 1H), 3.68-3.62 (m, 1H), 3.53 (dd, J = 9.0, 5.6 Hz, 1H), 1.21 (d, J = 7.0 Hz, 3H).

^{13}C NMR (125 MHz, $CDCl_3$) δ 205.05 (e), 161.59 (e) 138.30 (e), 133.74 (e), 130.88 (o), 128.44 (o), 127.67 (o), 120.75 (e), 119.26 (o), 113.19 (o), 73.44 (e), 72.71 (e), 55.81 (o), 45.39 (o), 14.10 (o).

IR (Neat) 3086 (w), 3064 (w), 3030 (w), 3004 (w), 2971 (w), 2933 (w), 2896 (w), 2855 (w), 2796 (w), 1688 (m), 1595 (s), 1558 (m), 1487 (m), 1454 (m), 1360 (w), 1297 (m), 1230 (s), 1211 (m), 1182 (m), 1096 (m), 1029 (s), 969 (m), 846 (m), 816 (m), 734 (s), 697 (s), 671 (m) cm^{-1}.

HRMS (DART) calcd. for $C_{18}H_{20}{}^{79}BrO_3$ $[M+H]^+$: 363.0590, found: 363.0579.

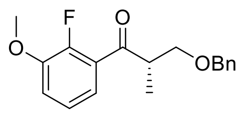

(*S*)-3-(Benzyloxy)-1-(2-fluoro-3-methoxyphenyl)-2-methylpropan-1-one (3fe)

Color and State: colorless oil

$[\alpha]_D^{20}$ −5.0 (c = 1.05, $CHCl_3$); (CHIRALPAK AD-H column) 98:1:1 hexane/isopropanol/methanol at 0.6 mL/min flow rate; tR (*S*)-enantiomer (major) = 17.9 min., tR (*R*)-enantiomer (minor) = 21.7 min.; ≥87% *ee* (≥87% *cee*) using Method A.

^{1}H NMR (500 MHz, $CDCl_3$) δ 7.32-7.24 (m, 6H), 7.14-7.09 (m, 2H), 4.51 (d, A of an AB quartet, J_{AB} = 12.2 Hz, 1H), 4.45 (d, A of an AB quartet, J_{AB} = 12.1 Hz, 1H), 3.91 (s, 3H), 3.82 (ddd, J =

9.0, 6.7, 1.1 Hz, 1H), 3.66 (dqd, J = 12.6, 6.8, 1.1 Hz, 1H), 3.55 (dd, J = 8.8, 5.8 Hz, 1H), 1.23 (d, J = 6.8 Hz, 3H).

^{13}C NMR (125 MHz, $CDCl_3$) δ 201.81 (e, d, $^3J_{C-F}$ = 3.7 Hz), 151.48 (e, d, $^1J_{C-F}$ = 253.7 Hz), 148.32 (e, d, $^2J_{C-F}$ = 11.9 Hz), 138.39 (e), 128.43 (o), 127.63 (o, d, $^3J_{C-F}$ = 2.7 Hz), 127.40 (e, d, $^2J_{C-F}$ = 11.1 Hz), 124.26 (o, d, $^3J_{C-F}$ = 4.6 Hz), 121.47 (o, d, $^4J_{C-F}$ = 1.8 Hz), 116.97 (o), 116.95 (o), 73.35 (e), 72.27 (e), 56.73 (o), 46.37 (o, d, $^4J_{C-F}$ = 5.5 Hz), 14.02 (o).

^{19}F NMR (471 MHz, $CDCl_3$) δ –134.82.

IR (Neat) 3086 (w), 3064 (w), 3027 (w), 3004 (w), 2971 (w), 2933 (w), 2855 (w), 2796 (w), 1685 (m), 1610 (w), 1580 (m), 1476 (s), 1454 (m), 1439 (m), 1364 (m), 1319 (m), 1271 (s), 1249 (m), 1211 (m), 1185 (m), 1092 (m), 1070 (s), 991 (s), 820 (m), 794 (m), 738 (s), 697 (s) cm^{-1}.

HRMS (DART) calcd. for $C_{18}H_{20}FO_3$ $[M+H]^+$: 303.1391, found: 303.1392.

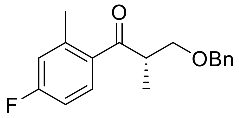

3ge

(*S*)-3-(Benzyloxy)-1-(4-fluoro-2-methylphenyl)-2-methylpropan-1-one (3ge)

Color and State: colorless oil

$[\alpha]_D^{20}$ +5.5 (c = 0.88, $CHCl_3$); (CHIRALPAK AD-H column) 98:1:1 hexane/isopropanol/methanol at 0.6 mL/min flow rate; tR (*S*)-enantiomer (major) = 9.4 min., tR (*R*)-enantiomer (minor) 10.8 min.; 88.5% *ee* (89% *cee*) using Method A.

^{1}H NMR (500 MHz, $CDCl_3$) δ 7.65 (dd, J = 8.3, 5.9 Hz, 1H), 7.33-7.24 (m, 5H), 6.95-6.90 (m, 2H), 4.51 (d, A of an AB quartet, J_{AB} = 12.2 Hz, 1H), 4.46 (d, B of an AB quartet, J_{AB} = 12.0 Hz, 1H), 3.78 (dd, J = 8.6, 7.3 Hz, 1H), 3.62-3.55 (m, 1H), 3.53 (dd, J = 8.7, 5.4 Hz, 1H), 2.46 (s, 3H), 1.17 (d, J = 6.9 Hz, 3H).

^{13}C NMR (125 MHz, $CDCl_3$) δ 205.83 (e), 163.91 (e, d, $^1J_{C-F}$ = 251.9 Hz), 141.82 (e, d, $^3J_{C-F}$ = 9.1 Hz), 138.28 (e), 134.80 (e, d, $^4J_{C-F}$ = 2.7 Hz), 130.72 (o, d, $^3J_{C-F}$ = 9.2 Hz), 128.46 (o), 127.71 (o), 127.64 (o), 118.62 (o, d, $^2J_{C-F}$ = 21.1 Hz), 112.53 (o, d, $^2J_{C-F}$ = 21.1 Hz), 73.50 (e), 72.86 (e), 44.73 (o), 21.20 (o), 14.40 (o).

^{19}F NMR (471 MHz, $CDCl_3$) δ –109.03.

IR (Neat) 3064 (w), 3030 (w), 2971 (w), 2930 (w), 2859 (w), 2792 (w), 1685 (s), 1603 (m), 1580 (s), 1495 (m), 1450 (m), 1360 (m), 1234 (s), 1208 (m), 1155 (m), 1096 (s), 1029 (m), 977 (s), 865 (m), 820 (m), 734 (s), 697 (s) cm^{-1}.

HRMS (DART) calcd. for $C_{18}H_{20}FO_2$ $[M+H]^+$: 287.1442, found: 287.1435.

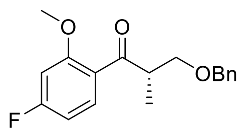

3he

(*S*)-3-(Benzyloxy)-1-(4-fluoro-2-methoxyphenyl)-2-methylpropan-1-one (3he)

Color and State: colorless oil

$[\alpha]_D^{20}$ −11.9 (*c* = 1.42, $CHCl_3$); (CHIRALPAK AD-H column) 98:1:1 hexane/isopropanol/methanol at 0.6 mL/min flow rate; tR (*S*)-enantiomer (major) = 14.6 min., tR (*R*)-enantiomer (minor) = 16.1 min.; ≥95.5% *ee* (96% *cee*) using Method A.

^{1}H NMR (500 MHz, $CDCl_3$) δ 7.65 (dd, *J* = 8.3, 7.0 Hz, 1H), 7.33-7.30 (m, 2H), 7.27-7.24 (m, 3H), 6.69 (ddd, *J* = 8.5, 7.9, 2.3 Hz, 1H), 6.64 (dd, *J* = 10.9, 2.3 Hz, 1H), 4.50 (d, A of an AB quartet, J_{AB} = 12.1 Hz, 1H), 4.45 (d, B of an AB quartet, J_{AB} = 12.0 Hz, 1H), 3.85 (s, 3H), 3.80 (dd, *J* = 8.5, 6.5 Hz, 1H), 3.80-3.73 (m, 1H), 3.50 (dd, *J* = 8.4, 5.7 Hz, 1H), 1.19 (d, *J* = 6.9 Hz, 3H).

^{13}C NMR (125 MHz, $CDCl_3$) δ 203.84 (e), 166.01 (e, d, $^1J_{C\text{-}F}$ = 252.8 Hz), 159.95 (e, d, $^3J_{C\text{-}F}$ = 11.0 Hz), 138.48 (e), 132.64 (o, d, $^3J_{C\text{-}F}$ = 11.0 Hz), 128.40 (o), 127.64 (o), 127.61 (o), 125.15 (e, d, $^4J_{C\text{-}F}$ = 2.8 Hz), 107.80 (o, d, $^2J_{C\text{-}F}$ = 21.2 Hz), 99.61 (o, d, $^2J_{C\text{-}F}$ = 26.6 Hz), 73.36 (e), 72.68 (e), 55.94 (o), 46.02 (o), 14.21 (o).

^{19}F NMR (471 MHz, $CDCl_3$) δ –104.79.

IR (Neat) 3086 (w), 3064 (w), 3030 (w), 3008 (w), 2971 (w), 2933 (w), 2859 (w), 1674 (m), 1603 (s), 1588 (s), 1495 (m), 1454 (m), 1409 (s), 1364 (m), 1278 (s), 1249 (s), 1196 (s), 1152 (s), 1100 (s), 1029 (s), 977 (s), 954 (s), 835 (s), 816 (m), 734 (s), 697 (s) cm^{-1}.

HRMS (DART) calcd. for $C_{18}H_{20}FO_3$ $[M+H]^+$: 303.1391, found: 303.1389.

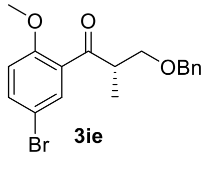

3ie

(*S*)-3-(Benzyloxy)-1-(5-bromo-2-methoxyphenyl)-2-methylpropan-1-one (3ie)

Color and State: colorless oil

$[\alpha]_D^{20}$ +2.3 (*c* = 1.14, $CHCl_3$); (CHIRALPAK AD-H column) 98:1:1 hexane/isopropanol/methanol at 0.6 mL/min flow rate; tR (*S*)-enantiomer (major) = 16.2 min., tR (*R*)-enantiomer (minor) = 23.6 min.; ≥98.5% *ee* (99% *cee*) using Method A.

^{1}H NMR (500 MHz, $CDCl_3$) δ 7.65 (d, *J* = 2.3 Hz, 1H), 7.51 (dd, *J* = 8.8, 2.4 Hz, 1H), 7.33-7.24 (m, 5H), 6.82 (d, *J* = 8.7 Hz, 1H), 4.49 (d, A of an AB quartet, J_{AB} = 12.1 Hz, 1H), 4.44, B of an AB quartet, J_{AB} = 12.0 Hz, 1H), 3.84 (s, 3H), 3.78-3.75 (m, 1H), 3.71 (sextet, *J* = 6.5 Hz, 1H), 3.50 (dd, *J* = 8.5, 5.7 Hz, 1H), 1.19 (d, *J* = 6.7 Hz, 3H).

^{13}C NMR (125 MHz, $CDCl_3$) δ 204.41 (e), 156.98 (e), 138.37 (e), 135.41 (o), 132.89 (o), 130.90 (e), 128.47 (o), 127.69 (o), 127.67 (o), 113.40 (o), 73.43 (e), 72.60 (e), 56.00 (o), 46.25 (o), 14.01 (o).

IR (Neat) 3064 (w), 3030 (w), 3004 (w), 2971 (w), 2933 (w), 2896 (w), 2855 (w), 1700 (m), 1592 (m), 1569 (m), 1461 (s), 1390 (m), 1286 (m), 1271 (s), 1237 (s), 1196 (m), 1174 (m), 1092 (s), 1070 (m), 1021 (s), 980 (s), 813 (m), 734 (s), 697 (s) cm^{-1}.

HRMS (DART) calcd. for $C_{18}H_{20}{}^{79}BrO_3$ $[M+H]^+$: 363.0590, found: 363.0589.

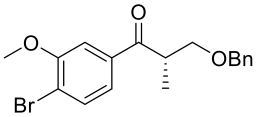
3je

(*S*)-3-(Benzyloxy)-1-(4-bromo-3-methoxyphenyl)-2-methylpropan-1-one (3je)

Color and State: colorless oil

$[\alpha]_D^{20}$ +14.1 (*c* = 1.26, $CHCl_3$); (CHIRALPAK AD-H column) 98:1:1 hexane/isopropanol/methanol at 1.0 mL/min flow rate; tR (*S*)-enantiomer (major) = 15.7 min., tR (*R*)-enantiomer (minor) = 17.6 min.; ≥97% *ee* (≥97.5% *cee*) using Method A.

^{1}H NMR (500 MHz, $CDCl_3$) δ 7.62 (d, *J* = 8.1 Hz, 1H), 7.51 (d, *J* = 1.4 Hz, 1H), 7.42, (dd, *J* = 8.2, 1.5 Hz, 1H), 7.33-7.24 (m, 5H), 4.51 (d, A of an AB quartet, J_{AB} = 12.2 Hz, 1H), 4.47 (d, B of an AB quartet, J_{AB} = 12.0 Hz, 1H), 3.93 (s, 3H), 3.82 (dd, *J* = 8.4, 7.6 Hz, 1H), 3.76 (sextet, *J* = 6.7 Hz, 1H), 3.56 (dd, *J* = 8.5, 5.4 Hz, 1H), 1.21 (d, *J* = 6.7 Hz, 3H).

^{13}C NMR (125 MHz, $CDCl_3$) δ 202.06 (e), 156.38 (e), 138.20 (e), 137.40 (e), 133.44 (o), 128.46 (o), 127.74 (o), 127.67 (o), 122.01 (o), 117.84 (e), 111.12 (o), 73.51 (e), 72.78 (e), 56.45 (o), 41.69 (o), 14.95 (o).

IR (Neat) 3064 (w), 3030 (w), 2971 (w), 2933 (w), 2855 (w), 2796 (w), 1677 (s), 1573 (m), 1480 (m), 1454 (m), 1401 (s), 1364 (m), 1275 (s), 1252 (s), 1196 (m), 1178 (m), 1096 (s), 1047 (s), 1025 (s), 1010 (s), 988 (m), 876 (m), 839 (m), 816 (m), 734 (s), 697 (s) cm^{-1}.

HRMS (DART) calcd. for $C_{18}H_{20}{}^{79}BrO_3$ $[M+H]^+$: 363.0590, found: 363.0590.

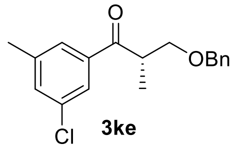

3ke

(*S*)-3-(Benzyloxy)-1-(3-chloro-5-methylphenyl)-2-methylpropan-1-one (3ke)

Color and State: colorless oil

$[\alpha]_D^{20}$ +12.3 (*c* = 1.17, $CHCl_3$); (CHIRALPAK AD-H column) 98:1:1 hexane/isopropanol/methanol at 0.6 mL/min flow rate; tR (*S*)-enantiomer (major) = 9.2 min., tR (*R*)-enantiomer (minor) = 10.9 min.; ≥93.5% *ee* (94% *cee*) using Method A.

^{1}H NMR (500 MHz, $CDCl_3$) δ 7.74 (s, 1H), 7.64 (s, 1H), 7.33 (s,1H), 7.33-7.25 (m, 5H), 4.52 (d, A of an AB quartet, J_{AB} = 12.1 Hz, 1H), 4.47 (d, B of an AB quartet, J_{AB} = 12.0 Hz, 1H), 3.81 (dd, *J* = 8.5, 7.7 Hz, 1H), 3.74 (sextet, *J* = 6.7 Hz, 1H), 3.54 (dd, *J* = 8.6, 5.6 Hz, 1H), 2.39 (s, 3H), 1.20 (d, *J* = 6.9 Hz, 3H).

^{13}C NMR (125 MHz, $CDCl_3$) δ 202.04 (e), 140.38 (e), 138.47 (e), 138.27 (e), 134.72 (e), 133.57 (o), 128.48 (o), 127.72 (o), 127.65 (o), 127.36 (o), 125.84 (o), 73.50 (e), 72.68 (e), 41.83 (o), 21.31 (o), 14.86 (o).

IR (Neat) 3064 (w), 3030 (w), 2971 (w), 2930 (w), 2855 (w), 1681 (s), 1595 (w), 1577 (m), 1495 (w), 1454 (m), 1435 (m), 1364 (m), 1260 (m), 1178 (m), 1096 (s), 1029 (m), 991 (m), 861 (m), 786 (m), 734 (s), 697 (s), 671 (s) cm^{-1}.

HRMS (DART) calcd. for $C_{18}H_{20}{}^{35}ClO_2$ $[M+H]^+$: 303.1146, found: 303.1154.

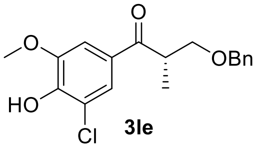

3le

(*S*)-3-(Benzyloxy)-1-(3-chloro-4-hydroxy-5-methoxyphenyl)-2-methylpropan-1-one (3le)

Color and State: yellow oil

$[\alpha]_D^{20}$ +3.2 (*c* = 1.20, $CHCl_3$); (CHIRALPAK AD-H column) 94:3:3 hexane/isopropanol/methanol at 0.6 mL/min flow rate; tR (*R*)-enantiomer (minor) = 13.1 min., tR (*S*)-enantiomer (major) = 14.3 min.; 94% *ee* (≥94% *cee*) using Method A.

^{1}H NMR (500 MHz, $CDCl_3$) δ 7.65 (d, J = 1.4 Hz, 1H), 7.45 (d, J = 1.3 Hz, 1H), 7.33-7.31 (m, 2H), 7.30-7.25 (m, 3H), 6.31 (brs, 1H), 4.52 (d, A of an AB quartet, J_{AB} = 12.1 Hz, 1H), 4.48 (d, A of an AB quartet, J_{AB} = 12.0 Hz, 1H), 3.93 (s, 3H), 3.81 (app. t, J = 8.2 Hz, 1H), 3.73 (sextet, J = 6.7 Hz, 1H), 3.54 (dd, J = 8.7, 5.6 Hz, 1H), 1.20 (d, J = 6.9 Hz, 3H).

^{13}C NMR (125 MHz, $CDCl_3$) δ 200.65 (e), 147.54 (e), 146.64 (e), 138.26 (e), 129.49 (e), 128.48 (o), 127.73 (o), 127.67 (o), 124.03 (o), 119.40 (e), 109.06 (o), 73.51 (e), 72.87 (e), 56.64 (o), 41.13 (o), 15.11 (o),

IR (Neat)) 3310 (br, w), 3064 (w), 3030 (w), 2971 (w), 2933 (w), 2859 (w), 2799 (w), 1670 (m), 1592 (m), 1502 (m), 1454 (m), 1413 (s), 1360 (m), 1278 (s), 1193 (s), 1174 (s), 1137 (m), 1092 (s), 1051 (s), 1021 (s), 906 (m), 876 (m), 854 (m), 734 (s), 697 (s), 667 (m) cm^{-1}.

HRMS (DART) calcd. for $C_{18}H_{20}{}^{35}ClO_4$ $[M+H]^+$: 335.1045, found: 335.1051.

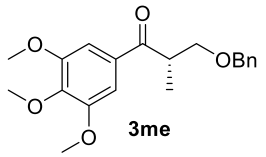

(*S*)-3-(Benzyloxy)-2-methyl-1-(3,4,5-trimethoxyphenyl)propan-1-one (3me)

Color and State: colorless oil

$[\alpha]_D^{20}$ +6.9 (c = 1.09, $CHCl_3$); (CHIRALPAK AD-H column) 98:1:1 hexane/isopropanol/methanol at 0.6 mL/min flow rate; tR (*S*)-enantiomer (major) = 23.5 min., tR (*R*)-enantiomer (minor) = 25.9 min.; ≥97.5% *ee* (98% *cee*) using Method A.

^{1}H NMR (500 MHz, $CDCl_3$) δ 7.32-7.29 (m, 2H), 7.27-7.25 (m, 5H), 4.53 (d, A of an AB quartet, J_{AB} = 11.9 Hz, 1H), 4.49 (d, B of an AB quartet, J_{AB} = 12.0 Hz, 1H), 3.92 (s, 3H), 3.88 (s, 6H), 3.83 (app. t, J = 8.0 Hz, 1H), 3.77 (sextet, J = 6.7 Hz, 1H), 3.56 (dd, J = 8.5, 5.8 Hz, 1H), 1.22 (d, J = 6.8 Hz, 3H).

^{13}C NMR (125 MHz, $CDCl_3$) δ 201.90 (e), 153.20 (e), 142.72 (e), 138.31 (e), 132.21 (e), 128.47 (o), 127.74 (o), 106.14 (o), 73.58 (e), 73.03 (e), 61.06 (o), 56.40 (o), 41.47 (o), 15.25 (o)

IR (Neat) 3060 (w), 3023 (w), 2933 (w), 2851 (w), 1674 (m), 1580 (m), 1502 (m), 1454 (m), 1413 (m), 1364 (m), 1316 (m), 1230 (m), 1193 (m), 1167 (m), 1122 (s), 1100 (m), 1051 (m), 1025 (m), 999 (m), 861 (m), 734 (m), 697 (m) cm^{-1}.

HRMS (DART) calcd. for $C_{20}H_{25}O_5$ $[M+H]^+$: 345.1697, found: 345.1701.

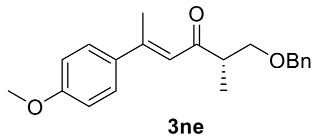

3ne

(*S,E*)-1-(Benzyloxy)-5-(4-methoxyphenyl)-2-methylhex-4-en-3-one (3ne)

Color and State: slightly yellow oil

$[\alpha]_D^{20}$ −20.4 (*c* = 0.60, $CHCl_3$); (CHIRALPAK IA-H column) 98:1:1 hexane/isopropanol/methanol at 0.6 mL/min flow rate; tR (*S*)-enantiomer (major) = 15.9 min., tR (*R*)-enantiomer (minor) = 17.2 min.; ≥99% *ee* (99.5% *cee*) using Method A; ***E/Z*** ≥19:1.

^{1}H NMR (500 MHz, $CDCl_3$) δ 7.47-7.44 (m, 2H), 7.32-7.26 (m, 5H), 6.91-6.89 (m, 2H), 6.57 (s, 1H), 4.55 (d, A of an AB quartet, J_{AB} = 12.1 Hz, 1H), 4.51 (d, B of an AB quartet= 12.0 Hz, 1H), 3.84 (s, 3H), 3.77-3.73 (m, 1H), 3.51 (dd, *J* = 8.4, 6.4 Hz, 1H), 2.99 (sextet, *J* = 6.8 Hz, 1H), 2.55 (s, 3H), 1.16 (d, *J* = 6.9 Hz, 3H).

^{13}C NMR (125 MHz, $CDCl_3$) δ 202.92 (e), 160.69 (e), 154.12 (e), 138.47 (e), 134.87 (e), 128.46 (o), 128.07 (o), 127.74 (o), 127.66 (o), 122.43 (o), 114.01 (o), 73.40 (e), 72.60 (e), 55.49 (o), 48.08 (o), 18.41 (o), 14.05 (o).

IR (Neat) 3056 (w), 3030 (w), 3001 (w), 2963 (w), 2933 (w), 2855 (w), 2840 (w), 1674 (m), 1588 (s), 1596 (s), 1510 (s), 1454 (m), 1439 (m), 1375 (m), 1360 (m), 1290 (m), 1249 (s), 1178 (s), 1155 (m), 1062 (s), 1029 (s), 965 (m), 827 (s), 805 (m), 734 (s), 697 (s) cm^{-1}.

HRMS (DART) calcd. for $C_{21}H_{25}O_3$ $[M+H]^+$: 325.1798, found: 325.1803.

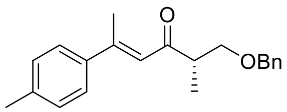

3oe

(*S,E*)-1-(Benzyloxy)-2-methyl-5-(p-tolyl)hex-4-en-3-one (3oe)

Color and State: slightly yellow oil

$[\alpha]_D^{20}$ −16.3 (*c* = 0.98, $CHCl_3$); (CHIRALPAK IA-H column) 99:0.5:0.5 hexane/isopropanol/methanol at 0.4 mL/min flow rate; tR (*S*)-enantiomer (major) = 20.6 min., tR (*R*)-enantiomer (minor) = 22.1 min.; 99% *ee* (99% *cee*) using Method A; ***E/Z*** ≥19:1.

^{1}H NMR (500 MHz, $CDCl_3$) δ 7.40-7.37 (m, 2H), 7.34-7.25 (m, 5H), 7.20-7.17 (m, 2H), 6.58 (q, *J* = 0.6 Hz, 1H), 4.55 (d, A of an AB quartet, J_{AB} = 12.1 Hz, 1H), 4.51 (d, B of an AB quartet, J_{AB} = 12.2 Hz, 1H), 3.75 (dd, *J* = 8.7, 7.7 Hz, 1H), 3.51 (dd, *J* = 9.0, 6.0 Hz, 1H), 2.99 (sextet, *J* = 6.8 Hz, 1H), 2.55 (s, 3H), 2.38 (s, 3H), 1.16 (d, *J* = 7.0 Hz, 3H).

^{13}C NMR (125 MHz, $CDCl_3$) δ 203.04 (e), 154.61 (e), 139.85 (e), 139.39 (e), 138.45 (e), 129.35 (o), 128.48 (o), 127.74 (o), 127.68 (o), 126.59 (o), 123.31 (o), 73.41 (e), 72.53 (o), 48.07 (o), 21.35 (o), 18.57 (o), 14.01 (o).

IR (Neat) 3086 (w), 3060 (w), 3027 (w), 2967 (w), 2922 (w), 2855 (w), 1677 (m), 1592 (s), 1565 (m), 1510 (w), 1495 (w), 1450 (m), 1360 (m), 1096 (m), 1062 (s), 1029 (m), 962 (m), 865 (w), 813 (s), 734 (s), 697 (s) cm^{-1}.

HRMS (DART) calcd. for $C_{21}H_{25}O_2$ $[M+H]^+$: 309.1849, found: 309.1850.

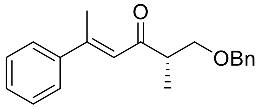

3pe

(*S*,*E*)-1-(Benzyloxy)-2-methyl-5-phenylhex-4-en-3-one (3pe)

Color and State: slightly yellow oil

$[\alpha]_D^{20}$ –0.5 (*c* = 1.01, $CHCl_3$); (CHIRALPAK AD-H column) 99:0.7:0.3 hexane/isopropanol/methanol at 0.6 mL/min flow rate; tR (*S*)-enantiomer (major) = 15.8 min., tR (*R*)-enantiomer (minor) = 17.4 min.; ≥99% *ee* (≥99.5% *cee*) using Method A; ***E/Z*** ≥19:1.

^{1}H NMR (500 MHz, $CDCl_3$) δ 7.49-7.45 (m, 2H), 7.40-7.36 (m, 3H), 7.33-7.25 (m, 5H), 6.58 (q, *J* = 1.2 Hz, 1H), 4.54 (d, A of an AB quartet, J_{AB} = 12.0 Hz, 1H), 4.51 (d, B of an AB quartet, J_{AB} = 12.1 Hz, 1H), 3.74 (dd, *J* = 9.3, 7.3 Hz, 1H), 3.52 (dd, *J* = 9.3, 5.8 Hz, 1H), 2.99 (sextet, *J* = 7.0 Hz, 1H), 2.55 (d, *J* = 0.9 Hz, 3H), 1.16 (d, *J* = 7.1 Hz, 3H).

^{13}C NMR (125 MHz, $CDCl_3$) δ 203.09 (e), 154.59 (e), 142.87 (e), 138.44 (e), 129.18 (o), 128.66 (o), 128.49 (o), 127.76 (o), 127.70 (o), 126.67 (o), 124.11 (o), 73.44 (e), 72.53 (e), 48.10 (o), 18.69 (o), 13.95 (o).

IR (Neat) 3060 (w), 3030 (w), 2967 (w), 2930 (w), 2855 (w), 2796 (w), 1677 (m), 1595 (s), 1573 (m), 1495 (w), 1446 (m), 1375 (m), 1360 (m), 1085 (m), 1059 (m), 1025 (m), 982 (m), 734 (m), 693 (s) cm^{-1}.

HRMS (DART) calcd. for $C_{20}H_{23}O_2$ $[M+H]^+$: 295.1693, found: 295.1688.

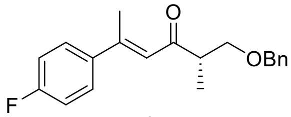

3qe

(*S*,*E*)-1-(Benzyloxy)-5-(4-fluorophenyl)-2-methylhex-4-en-3-one (3qe)

Color and State: slightly yellow oil

$[\alpha]_D^{20}$ −3.1 (*c* = 0.54, $CHCl_3$); (CHIRALPAK AD-H column) 99:1 hexane/isopropanol at 0.6 mL/min flow rate; tR (*S*)-enantiomer (major) = 14.2 min., tR (*R*)-enantiomer (minor) = 15.7 min.; ≥94.5% *ee* (95% *cee*) using Method A; ***E/Z*** ≥19:1.

^{1}H NMR (500 MHz, $CDCl_3$) δ 7.45-7.42 (m, 2H), 7.33-7.25 (m, 5H), 7.07-7.03 (m, 2H), 6.54 (q, *J* = 0.8 Hz, 1H), 4.54 (d, A of an AB quartet, J_{AB} = 12.0 Hz, 1H), 4.50 (d, B of an AB quartet, J_{AB} = 12.1 Hz, 1H), 3.73 (dd, *J* = 8.6, 7.9 Hz, 1H), 3.52 (dd, *J* = 9.1, 5.8 Hz, 1H), 2.99 (sextet, *J* = 6.8 Hz, 1H), 2.53 (s, 3H), 1.16 (d, *J* = 7.1 Hz, 3H).

^{13}C NMR (125 MHz, $CDCl_3$) δ 202.99 (e), 163.42 (e, d, $^1J_{C\text{-}F}$ = 249.1 Hz), 153.23 (e), 138.81 (e, d. $^4J_{C\text{-}F}$ = 2.8 Hz), 138.40 (e), 128.50 (o), 128.49 (o, d, $^3J_{C\text{-}F}$ = 8.3 Hz), 127.77 (o), 123.74 (o), 124.02 (o), 115.60 (o, d, $^2J_{C\text{-}F}$ = 22.0 Hz), 73.45 (e), 72.55 (e), 48.07 (o), 18.69 (o), 13.91 (o).

^{19}F NMR (471 MHz, $CDCl_3$) δ –112.35.

IR (Neat) 3064 (w), 3030 (w), 2967 (w), 2930 (w), 2855 (w), 2796 (w), 1677 (m), 1599 (s), 1584 (s), 1506 (s), 1454 (m), 1375 (m), 1360 (m), 1234 (m), 1159 (m), 1100 (m), 1062 (s), 1029 (m), 1014 (w), 965 (m), 831 (s), 734 (m), 697 (s) cm^{-1}.

HRMS (DART) calcd. for $C_{20}H_{22}FO_2$ $[M+H]^+$: 313.1598, found: 313.1601.

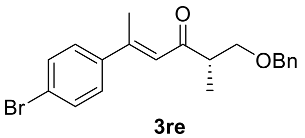

3re

(*S,E*)-1-(Benzyloxy)-5-(4-bromophenyl)-2-methylhex-4-en-3-one (3re)

Color and State: yellow oil

$[\alpha]_D^{20}$ −13.5 (*c* = 1.19, $CHCl_3$); (CHIRALPAK AD-H column) 99:0.7:0.3 hexane/isopropanol/methanol at 0.6 mL/min flow rate; tR (*S*)-enantiomer (major) = 19.6 min., tR (*R*)-enantiomer (minor) = 21.7 min.; ≥95.5% *ee* (96% *cee*) using Method A; ***E/Z*** ≥19:1.

^{1}H NMR (500 MHz, $CDCl_3$) δ 7.50-7.47 (m, 2H), 7.36-7.26 (m, 7H), 6.55 (q, *J* = 1.4 Hz, 1H), 4.53 (d, A of an AB quartet, J_{AB} = 12.2 Hz, 1H), 4.50 (d, B of an AB quartet, J_{AB} = 12.1 Hz, 1H), 3.72 (dd, *J* = 9.0, 7.6 Hz, 1H), 3.52 (dd, *J* = 9.1, 5.7 Hz, 1H), 2.99 (sextet, *J* = 6.7 Hz, 1H), 2.51 (s, 3H), 1.15 (d, *J* = 6.8 Hz, 3H).

^{13}C NMR (125 MHz, $CDCl_3$) δ 202.99 (e), 152.96 (e), 141.65 (e), 138.34 (e), 131.79 (o), 128.48 (o), 128.24 (o), 127.75 (o), 127.73 (o), 124.37 (o), 123.38 (e), 73.43 (e), 72.49 (e), 48.05 (o), 18.46 (o), 13.83 (o).

IR (Neat) 3086 (w), 3064 (w), 3027 (w), 2967 (w), 2930 (w), 2855 (w), 2796 (w), 1677 (m), 1595 (s), 1588 (m), 1483 (m), 1454 (m), 1435 (m), 1401 (m), 1360 (m), 1264 (w), 1096 (m), 1062 (s), 1029 (m), 1006 (s), 962 (m), 906 (m), 876 (m), 816 (s), 734 (s), 697 (s) cm^{-1}.

HRMS (DART) calcd. for $C_{20}H_{22}{}^{79}BrO_2$ $[M+H]^+$: 373.0798, found: 373.0804.

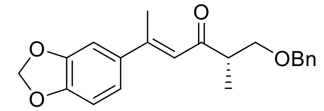

3se

(*S,E*)-5-(Benzo[*d*][*1,3*]dioxol-5-yl)-1-(benzyloxy)-2-methylhex-4-en-3-one (3se)

Color and State: yellow oil

$[\alpha]_D^{20}$ −18.6 (*c* = 0.56, $CHCl_3$); (CHIRALPAK AD-H column) 98:1:1 hexane/isopropanol/methanol at 0.6 mL/min flow rate; tR (*S*)-enantiomer (major) = 20.5 min., tR (*R*)-enantiomer (minor) = 25.5 min.; 99% *ee* (99.5% *cee*) using Method A; ***E/Z*** ≥19:1.

^{1}H NMR (500 MHz, $CDCl_3$) δ 7.34-7.25 (m, 5H), 7.01 (dd, J = 8.2, 1.5 Hz, 1H), 6.97 (d, J = 1.5 Hz, 1H), 6.80 (d, J = 8.1 Hz, 1H), 6.52 (q, J = 1.0 Hz, 1H), 6.00 (s, 2H), 4.54 (d, A of an AB quartet, J_{AB} = 12.2 Hz, 1H), 4.50 (d, B of an AB quartet, J_{AB} = 12.1 Hz, 1H), 3.73 (dd, J = 9.0, 7.5 Hz, 1H), 3.50 (dd, J = 9.3, 5.8 Hz, 1H), 2.97 (sextet, J = 6.8 Hz, 1H), 2.51 (s, 3H), 1.15 (d, J = 7.1 Hz, 3H).

^{13}C NMR (125 MHz, $CDCl_3$) δ 202.93 (e), 154.04 (e), 148.67 (e), 148.08 (e), 138.44 (e), 136.90 (e), 128.49 (o), 127.75 (o), 127.70 (o), 122.96 (o), 120.97 (o), 108.34 (o), 106.98 (o), 101.55 (e), 73.42 (e), 72.55 (e), 48.09 (o), 18.68 (o), 14.02 (o).

IR (Neat) 3064 (w), 3030 (w), 2967 (w), 2922 (w), 2896 (w), 2874 (w), 2859 (w), 2781 (w), 1674 (m), 1584 (s), 1502 (m), 1487 (s), 1439 (s), 1379 (m), 1360 (m), 1252 (s), 1223 (s), 1159 (m), 1107 (m), 1066 (s), 1036 (s), 969 (m), 932 (m), 913 (m), 854 (m), 809 (s), 734 (s), 697 (s) cm^{-1}.

HRMS (DART) calcd. for $C_{21}H_{23}O_4$ $[M+H]^+$: 339.1591, found: 339.1593.

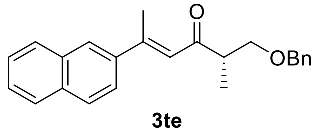

3te

(*S,E*)-1-(Benzyloxy)-2-methyl-5-(naphthalen-2-yl)hex-4-en-3-one (3te)

Color and State: slightly yellow oil

$[\alpha]_D^{20}$ −6.0 (*c* = 0.92, $CHCl_3$); (CHIRALPAK AD-H column) 98:1:1 hexane/isopropanol/methanol at 0.6 mL/min flow rate; tR (*S*)-enantiomer (major) = 27.0 min., tR (*R*)-enantiomer (minor) = 28.9 min.; 99% *ee* (99% *cee*) using Method A; ***E/Z*** ≥19:1.

^{1}H NMR (500 MHz, $CDCl_3$) δ 7.94 (d, *J* = 0.6 Hz, 1H), 7.87-7.82 (m, 3H), 7.59 (dd, *J* = 8.6, 1.6 Hz, 1H), 7.53-7.49 (m, 2H), 7.34-7.24 (m, 5H), 6.73 (q, *J* = 0.9 Hz, 1H), 4.56 (d, A of an AB quartet, J_{AB} = 12.1 Hz, 1H), 4.52 (d, B of an AB quartet, J_{AB} = 12.0 Hz, 1H), 3.77 (dd, *J* = 9.1, 7.6 Hz, 1H), 3.55 (dd, *J* = 9.2, 5.8 Hz, 1H), 3.04 (sextet, *J* = 7.0 Hz, 1H), 2.66 (s, 3H), 1.19 (d, *J* = 7.1 Hz, 3H).

^{13}C NMR (125 MHz, $CDCl_3$) δ 203.09 (e), 154.30 (e), 139.97 (e), 138.43 (e), 133.68 (e), 133.30 (e), 128.65 (o), 128.49 (o), 128.29 (o), 127.75 (o), 127.73 (o), 126.70 (o), 126.86 (o), 126.65 (o), 126.36 (o), 124.47 (o), 124.25 (o), 73.44 (e), 72.57 (e), 48.13 (o), 18.68 (o), 13.98 (o).

IR (Neat) 3056 (w), 3027 (w), 2967 (w), 2930 (w), 2855 (m), 2796 (w), 1677 (m), 1588 (s), 1495 (w), 1450 (m), 1435 (m), 1379 (m), 1360 (m), 1155 (m), 1059 (s), 1029 (m), 965 (m), 895 (m), 876 (m), 854 (m), 813 (s), 734 (s), 697 (s), 656 (m) cm^{-1}.

HRMS (DART) calcd. for $C_{24}H_{25}O_2$ $[M+H]^+$: 345.1849, found: 345.1857.

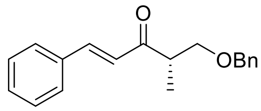

3ue

(*S,E*)-5-(Benzyloxy)-4-methyl-1-phenylpent-1-en-3-one (3ue)

Color and State: yellow oil

$[\alpha]_D^{20}$ −1.3 (*c* = 0.59, $CHCl_3$); (CHIRALPAK AD-H column) 99:0.7:0.3 hexane/isopropanol/methanol at 0.6 mL/min flow rate; tR (*S*)-enantiomer (major) = 29.0 min., tR (*R*)-enantiomer (minor) = 37.7 min.; ≥99% *ee* (99.5% *cee*) using Method A; ***E/Z*** ≥19:1.

^{1}H NMR (500 MHz, $CDCl_3$) δ 7.63 (d, *J* = 16.1 Hz, 1H), 7.57-7.53 (m, 2H), 7.41-7.38 (m, 3H), 7.31-7.24 (m, 5H), 6.84 (d, *J* = 16.1 Hz, 1H), 4.54 (d, A of an AB quartet, J_{AB} = 12.1 Hz, 1H), 4.51 (d, B of an AB quartet, J_{AB} = 12.1 Hz, 1H), 3.76 (dd, *J* = 9.2, 7.5 Hz, 1H), 3.54 (dd, *J* = 9.2, 5.8 Hz, 1H), 3.27-3.20 (m, 1H), 1.19 (d, *J* = 7.0 Hz, 1H)..

^{13}C NMR (125 MHz, $CDCl_3$) δ 202.13 (e), 142.91 (o), 138.34 (e), 134.82 (e), 130.55 (o), 129.03 (o), 128.51 (o), 128.49 (o), 127.75 (o), 127.72 (o), 125.47 (o), 73.47 (e), 72.45 (e), 45.13 (o), 14.17 (o).

IR (Neat) 3083 (w), 3060 (w), 3027 (w), 2971 (w), 2930 (w), 2855 (w), 2796 (w), 1685 (m), 1655 (m), 1606 (s), 1577 (m), 1495 (m), 1450 (m), 1364 (m), 1331 (m), 1189 (m), 1096 (m), 1070 (m), 1051 (s), 977 (m), 906 (w), 760 (m), 734 (s), 697 (s) cm^{-1}.

HRMS (DART) calcd. for $C_{19}H_{21}O_2$ $[M+H]^+$: 281.1536, found: 281.1536.

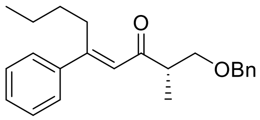

3ve

(*S*,*E*)-1-(Benzyloxy)-2-methyl-5-phenylnon-4-en-3-one (3ve)

Color and State: slight yellow oil

$[\alpha]_D^{20}$ −2.0 (*c* = 0.27, $CHCl_3$); (CHIRALPAK AD-H column) 99.5:0.5 hexane/methanol at 0.4 mL/min flow rate; tR (*S*)-enantiomer (major) = 45.2 min., tR = (S)-enantiomer (minor) = 49.9 min.; 99.5% *ee* (≥99.5% *cee*) using Method A; ***E/Z*** ≥19:1.

^{1}H NMR (500 MHz, $CDCl_3$) δ 7.45-7.38 (m, 2H), 7.38-7.36 (m, 3H), 7.32-7.25 (m, 5H), 6.47 (s, 1H), 4.54 (d, A of an AB quartet, J_{AB} = 12.1 Hz, 1H), 4.51 (d, B of an AB quartet, J_{AB} = 12.1 Hz, 1H), 3.74 (dd, *J* = 9.2, 7.2 Hz, 1H), 3.52 (dd, *J* = 9.2, 6.1 Hz, 1H), 3.09-2.95 (m, 3H), 1.43-1.33 (m, 4H), 1.16 (d, *J* = 7.0 Hz, 3H), 0.88 (t, *J* = 7.0 Hz, 3H).

^{13}C NMR (125 MHz, $CDCl_3$) δ 202.66 (e), 159.95 (e), 142.07 (e), 138.44 (e), 129.00 (o), 128.64 (o), 128.46 (o), 127.74 (o), 127.67 (o), 126.96 (o), 124.27 (o), 73.40 (e), 72.54 (e), 48.07 (o), 31.37 (e), 31.32 (e), 23.06 (e), 14.02 (o), 13.97 (o).

IR (Neat) 3083 (w), 3060 (w), 3027 (w), 2956 (w), 2930 (w), 2859 (w), 2796 (w), 1677 (m), 1592 (m), 1573 (m), 1495 (w), 1450 (m), 1364 (m), 1096 (m), 1062 (m), 1029 (m), 995 (m), 764 (m), 734 (m), 693 (s) cm^{-1}.

HRMS (DART) calcd. for $C_{23}H_{29}O_3$ $[M+H]^+$: 337.2162, found: 337.2164.

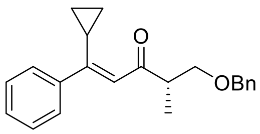

3we

(*S*,*E*)-5-(Benzyloxy)-1-cyclopropyl-4-methyl-1-phenylpent-1-en-3-one (3we)

Color and State: slightly yellow oil

$[\alpha]_D^{20}$ +7.3 (*c* = 1.31, $CHCl_3$); (CHIRALPAK AD-H column) 99:0.5:0.5 hexane/isopropanol/methanol at 0.4 mL/min flow rate; tR (*S*)-enantiomer (major) = 19.6 min., tR (*R*)-enantiomer (minor) = 21.1 min.; ≥99.5% *ee* (100% *cee*) using Method A; ***E/Z*** ≥19:1.

^{1}**H NMR** (500 MHz, $CDCl_3$) δ 7.34-7.26 (m, 8H), 7.14-7.13 (m, 2H), 6.24 (s, 1H), 4.55 (d, A of an AB quartet, J_{AB} = 12.1 Hz, 1H), 4.50 (d, B of an AB quartet, J_{AB} = 12.0 Hz, 1H), 3.75 (dd, *J* = 9.1, 7.4 Hz, 1H), 3.50 (dd, *J* = 9.2, 5.9 Hz, 1H), 3.19 (ddt, *J* = 13.6, 8.4, 5.3 Hz, 1H), 2.95 (sextet, *J* = 6.9 Hz, 1H), 1.16 (d, *J* = 7.1 Hz, 3H), 0.93-0.89 (m, 2H), 0.51-0.46 (m, 2H).

^{13}**C NMR** (125 MHz, $CDCl_3$) δ 203.01 (e), 162.73 (e), 139.21 (e), 138.49 (e), 128.46 (o), 128.22 (o), 127.91 (o), 127.88 (o), 127.73 (o), 127.66 (o), 125.83 (o), 73.40 (e), 72.54 (e), 47.96 (o), 14.18 (o), 14.05 (o), 7.58 (e), 7.57 (e).

IR (Neat) 3083 (w), 3060 (w), 3027 (w), 3012 (w), 2967 (w), 2930 (w), 2855 (w), 2792 (w), 1674 (m), 1580 (m), 1487 (w), 1454 (m), 1360 (w), 1096 (m), 1055 (m), 1029 (m), 969 (m), 917 (m), 857 (m), 764 (m), 734 (m), 697 (s) cm^{-1}.

HRMS (DART) calcd. for $C_{22}H_{25}O_2$ $[M+H]^+$: 321.1849, found: 321.1850.

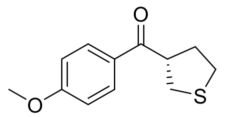
3ck

(*S*)-(4-Methoxyphenyl)(tetrahydrothiophen-3-yl)methanone (3ck)

Color and State: yellow oil

$[\alpha]_D^{20}$ +16.8 (*c* = 0.42, $CHCl_3$); (CHIRALPAK IA-H column) 96:2:2 hexane/isopropanol/methanol at 0.6 mL/min flow rate; tR (*S*)-enantiomer (major) = 32.6 min., tR (*R*)-enantiomer (minor) = 43.4 min.; 88% *ee* (≥88% *cee*) using Method A.

^{1}**H NMR** (500 MHz, $CDCl_3$) δ 7.98-7.95 (m, 2H), 6.97-6.94 (m, 2H), 3.93 (pentet, *J* = 7.8 Hz, 1H), 3.88 (s, 3H), 3.14-3.07 (m, 2H), 3.02 (ddd, *J* = 10.5, 7.2, 4.2 Hz, 1H), 2.96 (ddd, *J* = 10.3, 9.0, 6.4 Hz, 1H), 2.34 (pentd, *J* = 6.2, 4.4 Hz, 1H), 2.30-2.22 (m, 1H).

^{13}**C NMR** (125 MHz, $CDCl_3$) δ 198.11 (e), 163.85 (e), 130.84 (o), 129.52 (e), 114.06 (o), 55.66 (o), 50.38 (o), 34.83 (e), 34.32 (e), 31.33 (e).

IR (Neat) 3004 (w), 2930 (w), 2855 (w), 2840 (w), 1666 (s), 1595 (s), 1578 (m), 1510 (m), 1457 (w), 1439 (w), 1420 (m), 1357 (m), 1308 (m), 1256 (s), 1234 (s), 1167 (s), 1114 (m), 1018 (s), 995 (m), 980 (m), 865 (m), 835 (s), 805 (m), 783 (m), 753 (m), 671 (m) cm^{-1}.

HRMS (DART) calcd. for $C_{12}H_{15}O_2S$ $[M+H]^+$: 223.0787, found: 223.0790.

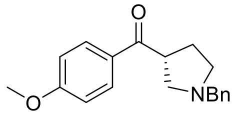

3cl

(*R*)-(1-Benzylpyrrolidin-3-yl)(4-methoxyphenyl)methanone (3cl)

Color and State: yellow oil

$[\alpha]_D^{20}$ −5.1 (*c* = 0.85, $CHCl_3$); (CHIRALPAK AD-H column) 88.9:5:5:0.1 hexane/isopropanol/methanol/diethyl amine at 1.0 mL/min flow rate; tR (*R*)-enantiomer (major) = 22.2 min., tR = (*S*)-enantiomer (minor) = 24.4 min.; ≥94.5% *ee* (99% *cee*) using Method B.

^{1}H NMR (500 MHz, $CDCl_3$) δ 7.94-7.91 (m, 2H), 7.35-7.29 (m, 4H), 7.26-7.23 (m, 1H), 6.94-6.91 (m, 2H), 3.93 (dtd, *J* = 9.9, 7.9, 6.2 Hz, 1H), 3.86 (s, 3H), 3.66 (s, 2H), 3.05 (app. t, *J* = 8.8 Hz, 1H), 2.85 (td, *J* = 8.2, 4.5 Hz, 1H), 2.68 (dd, *J* = 9.2, 7.8 Hz, 1H), 2.50 (app. q, *J* = 8.3 Hz, 1H), 2.23-2.11 (m, 2H).

^{13}C NMR (125 MHz, $CDCl_3$) δ 199.20 (e), 163.56 (e), 138.97 (e), 130.86 (o), 129.67 (e), 128.99 (o), 128.41 (o), 127.14 (o), 113.92 (o), 60.38 (e), 56.94 (e), 55.58 (o), 54.21 (e), 44.82 (o), 28.12 (e).

IR (Neat) 3060 (w), 3027 (w), 3004 (w), 2956 (w), 2933 (w), 2915 (w), 2837 (w), 2788 (w), 2736 (w), 1670 (m), 1595 (s), 1573 (m), 1510 (m), 1454 (m), 1420 (w), 1353 (m), 1308 (m), 1260 (s), 1223 (s), 1167 (s), 1114 (m), 1025 (m), 995 (m), 839 (m), 749 (s), 697 (s) cm^{-1}

HRMS (DART) calcd. for $C_{19}H_{22}NO_2$ $[M+H]^+$: 296.1645, found: 296.1652.

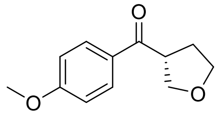

3cm

(*R*)-(4-Methoxyphenyl)(tetrahydrofuran-3-yl)methanone (3cm)

Color and State: colorless oil

$[\alpha]_D^{20}$ +13.8 (*c* = 1.26, $CHCl_3$); (CHIRALPAK AD-H column) 95:5 hexane/isopropanol at 1.0 mL/min flow rate; tR (*R*)-enantiomer (major) = 42.2 min., tR (*S*)-enantiomer (minor) = 50.1 min.; ≥98 % *ee* (≥98.5% *cee*) using Method C.

^{1}H NMR (500 MHz, $CDCl_3$) δ 7.95-7.92 (m, 2H), 6.96-6.93 (m, 2H), 4.12-4.06 (m, 1H), 3.99-3.88 (m, 4H), 3.87 (s, 3H), 2.33-2.27 (m, 1H), 2.21-2.12 (m, 1H).

^{13}C NMR (125 MHz, $CDCl_3$) δ 198.04 (e), 163.77 (e), 130.82 (o), 129.58 (e), 114.02 (o), 70.57 (e), 68.69 (e), 55.62 (o), 46.15 (o), 29.92 (e).

IR (Neat) 3071 (w), 3053 (w), 2952 (w), 2937 (w), 2859 (w), 2844 (w), 1670 (m), 1595 (s), 1573 (m), 1510 (m), 1457 (w), 1420 (m), 1308 (m), 1256 (s), 1223 (s), 1167 (s), 1114 (m), 1059 (m), 1025 (m), 955 (m), 917 (m), 898 (m), 839 (s), 764 (m) cm^{-1}.

HRMS (DART) calcd. for $C_{12}H_{15}O_3$ $[M+H]^+$: 207.1016, found: 207.1015.

10. Experimental Procedures and Characterization Data for Ester 4, Homologated Ketone 5, Ether 6, and Benzylic Alcohol 7

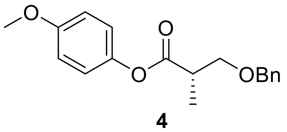

4

4-Methoxyphenyl (*S*)-3-(Benzyloxy)-2-methylpropanoate (4).[36] To a solution of (*S*)-3-(benzyloxy)-1-(4-methoxyphenyl)-2-methylpropan-1-one (**3ce,** 56.9 mg, 0.2 mmol) in DCM (2 mL) were added *meta*-chloroperoxybenzoic acid (68.8 mg, 0.4 mmol, 2.0 equiv.) and trifluoroacetic acid (0.1 mL). The resulting mixture was stirred at room temperature for *ca.* 48 h (monitored by TLC). The reaction was then diluted with DCM (10 mL) and quenched with saturated aqueous $NaHCO_3$ solution. The biphasic mixture was extracted with DCM, and the combined organic layers were dried (anhyd. $MgSO_4$), filtered, and concentrated *in vacuo* to afford the crude product. Purification by flash column chromatography (silica gel, eluting with 5-10% diethyl ether/hexane) afforded the chiral ester (*S*)-**4** (47.8 mg, 80%) as colorless oil: $[\alpha]_D^{20}$ +27.8 (c = 0.59, $CHCl_3$); (CHIRALPAK AS-H column) 99:0.5:0.5 hexane/isopropanol/methanol at 0.4 mL/min flow rate; tR (*S*)-enantiomer (major) = 20.3 min., tR (*R*)-enantiomer (minor) = 23.6 min.; 99% *ee* (99.5% *cee*).

^{1}H NMR (500 MHz, $CDCl_3$) δ 7.35-7.26 (m, 5H), 6.99-6.96 (m, 2H), 6.89-6.86 (m, 2H), 4.58 (s, 2H), 3.80 (s, 3H), 3.78 (dd, J = 8.9, 7.5 Hz, 1H), 3.65 (dd, J = 9.1, 5.7 Hz, 1H), 3.05-2.98 (m, 1H), 1.32 (d, J = 7.1 Hz, 3H).

^{13}C NMR (125 MHz, $CDCl_3$) δ 173.87 (e), 157.37 (e), 144.48 (e), 138.30 (e), 128.55 (o), 127.80 (o), 127.76 (o), 122.44 (o), 114.57 (o), 73.42 (e), 72.22 (e), 55.75 (o), 40.60 (o), 14.10 (o).

IR (Neat) 3060 (w), 3030 (w), 2971 (w), 2937 (w), 2907 (w), 2859 (w), 2837 (w), 2803 (w), 1752 (s), 1674 (w), 1595 (w), 1502 (s), 1454 (m), 1360 (w), 1249 (m), 1223 (m), 1193 (s), 1163 (s), 1129 (m), 1100 (s), 1029 (s), 872 (m), 816 (m), 738 (m), 697 (m), 652 (w) cm^{-1}.

HRMS (DART) calcd. for $C_{18}H_{21}O_4$ $[M+H]^+$: 301.1434, found: 301.1430.

(*S*)-4-(Benzyloxy)-1-(4-methoxyphenyl)-3-methylbutan-2-one (5).[37] To a solution of (*S*)-3-

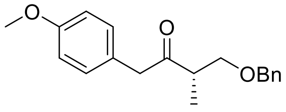

5

(benzyloxy)-1-(4-methoxyphenyl)-2-methylpropan-1-one (**3ce,** 56.9 mg, 0.2 mmol) in DCM (2mL) at –40 °C was added a solution of diethylaluminum chloride (0.16 mL, 25% wt. in toluene, *ca.* 1.5 equiv.). The mixture was stirred for *ca.* 10 min, and then trimethylsilyldiazomethane (0.25 mL, 1.6 M in hexane, 2.0 equiv.) was added and the reaction was stirred for *ca.* 1 h. Tetrabutylammonium fluoride (1.0 mL, 1.0 M in THF, 5.0 equiv.) was added, and the reaction was diluted with DCM (10 mL) and quenched by the addition of saturated aqueous $NaHCO_3$ solution. The biphasic mixture was extracted with DCM, and the combined organic layers were dried (anhyd. $MgSO_4$), filtered, and concentrated *in vacuo* to afford the crude product. Purification by flash column chromatography (silica gel, eluting with 20-40% diethyl ether/hexane) afforded the chiral ketone (*S*)-**5** (38.1 mg, 64%) as colorless oil: $[\alpha]_D^{20}$ +34.0 (*c* = 0.47, $CHCl_3$); (CHIRALCEL OJ-H column) 98:5:5 hexane/isopropanol/methanol at 0.6 mL/min flow rate; tR (*R*)-enantiomer (minor) = 24.2 min., tR (*S*)-enantiomer (major) = 29.4 min.; ≥99% *ee* (≥99.5% *cee*).

^{1}H NMR (500 MHz, $CDCl_3$) δ 7.36-7.26 (m, 5H), 7.11-7.08 (m, 2H), 6.86-6.83 (m, 2H), 4.49 (d, A of an AB quartet, J_{AB} = 12.3 Hz, 1H), 4.46 (d, B of an AB quartet, J_{AB} = 12.1 Hz, 1H), 3.79 (s, 3H), 3.75 (d, A of an AB quartet, J_{AB} = 15.9 Hz, 1H), 3.71 (d, A of an AB quartet, J_{AB} = 16.0 Hz, 1H), 3.63 (app. t, *J* = 8.6 Hz, 1H), 3.48 (dd, *J* = 9.0, 5.6 Hz, 1H), 3.01 (sextet, *J* = 6.9 Hz, 1H), 1.05 (d, *J* = 7.0 Hz, 3H).

^{13}C NMR (125 MHz, $CDCl_3$) δ 210.94 (e), 158.70 (e), 138.25 (e), 130.74 (o), 128.50 (o), 127.75 (o), 127.72 (o), 126.26 (e), 114.18 (o), 73.41 (e), 72.67 (e), 55.37 (o), 48.82 (e), 45.55 (o), 13.80 (o).

IR (Neat) 3064 (w), 3030 (w), 2956 (w), 2930 (w), 2855 (w), 2796 (w), 1711 (m), 1674 (w), 1610 (w), 1584 (w), 1510 (s), 1454 (m), 1301 (m), 1245 (s), 1178 (m), 1096 (m), 1073 (m), 1029 (s), 820 (m), 794 (m), 734 (s), 697 (s) cm^{-1}.

HRMS (DART) calcd. for $C_{19}H_{23}O_3$ $[M+H]^+$: 299.1642, found: 299.1647.

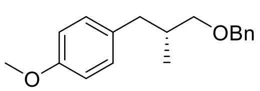

6

(*R*)-1-(3-(Benzyloxy)-2-methylpropyl)-4-methoxybenzene (6).[38] To a solution of (*S*)-3-(benzyloxy)-1-(4-methoxyphenyl)-2-methylpropan-1-one (**3ce,** 56.9 mg, 0.2 mmol) in trifluoroacetic acid (0.2 mL) was added

triethylsilane (46.5 mg, 0.4 mmol, 2.0 equiv.) at room temperature. The mixture was stirred for *ca.* 24 h (monitored by TLC), diluted with DCM (10 mL) and quenched with saturated aqueous $NaHCO_3$ solution. The biphasic mixture was extracted with DCM, and the combined organic layers were dried (anhyd. $MgSO_4$), filtered, and concentrated *in vacuo* to afford the crude product. Purification by flash column chromatography (silica gel, eluting with 2-5% diethyl ether/hexane) afforded the chiral ketone (*R*)-**6** (47.8 mg, 88%) as a colorless oil: $[\alpha]_D^{20}$ −6.2 (*c* = 0.72, $CHCl_3$); (CHIRALPAK AS-H column) 99:0.7:0.3 hexane/isopropanol/methanol at 0.6 mL/min flow rate; tR (*R*)-enantiomer (major) = 8.6 min., tR (*S*)-enantiomer (minor) = 12.4 min.; ≥99% *ee* (≥99.5% *cee*).

^{1}H NMR (500 MHz, $CDCl_3$) δ 7.36-7.26 (m, 5H), 7.08-7.06 (m, 2H), 6.83-6.80 (m, 2H), 4.52 (d, A of an AB quartet, J_{AB} =. 12.7 Hz, 1H), 4.50 (d, B of an AB quartet, J_{AB} =. 12.4 Hz, 1H), 3.79 (s, 3H), 3.33 (dd, *J* = 9.0, 6.2 Hz, 1H), 3.29 (dd, *J* = 8.9, 6.2 Hz, 1H), 2.75 (dd, *J* = 13.6, 5.9 Hz, 1H), 2.38 (dd, *J* = 13.5, 8.1 Hz, 1H), 2.04 (octet, *J* = 6.7 Hz, 1H), 0.91 (d, *J* = 6.7 Hz, 3H).

^{13}C NMR (125 MHz, $CDCl_3$) δ 157.91 (e), 138.94 (e), 132.92 (e), 130.25 (o), 128.47 (o), 127.70 (o), 127.59 (o), 113.72 (o), 75.17 (e), 73.15 (e), 55.38 (o), 39.12 (e), 35.79 (o), 16.99 (o).

IR (Neat) 3086 (w), 3064 (w), 3030 (w), 3001 (w), 2952 (w), 2930 (w), 2907 (w), 2851 (w), 2792 (w), 1610 (w), 1584 (w), 1510 (s), 1454 (m), 1360 (w), 1241 (s), 1174 (m), 1092 (m), 1073 (m), 1032 (s), 839 (m), 801 (m), 734 (s), 697 (s) cm^{-1}.

HRMS (DART) calcd. for $C_{18}H_{23}O_2$ $[M+H]^+$: 271.1693, found: 271.1704.

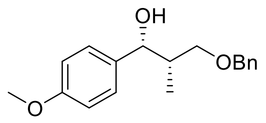

7

(1*R*,2*S*)-3-(Benzyloxy)-1-(4-methoxyphenyl)-2-methylpropan-1-ol (7).[39] Titanium tetrachloride (0.24 mL, 1M in DCM, 1.2 equiv.) was added to a stirred solution of (*S*)-3-(benzyloxy)-1-(4-methoxyphenyl)-2-methylpropan-1-one (**3ce,** 56.9 mg, 0.2 mmol) in anhydrous DCM (2 mL) at −78 °C followed by the dropwise addition of diisobutylaluminium hydride (DIBAL-H, 0.25 mL, 25 wt% in toluene, *ca.* 2 equiv.). The mixture was stirred for *ca.* 1 h (monitored by TLC) and then carefully quenched by the addition of HCl (1 M aqueous solution, 5 mL), followed by dilution with DCM (5 mL). The mixture was stirred at room temperature until two clear layers formed. The biphasic mixture was extracted with DCM, and the combined organic layers were dried (anhyd. $MgSO_4$), filtered, and concentrated *in vacuo* to afford the crude product. Purification by flash

column chromatography (silica gel, eluting with 20-40% diethyl ether/hexane) afforded the chiral ketone (1*R*,2*S*)-**7** (48.0 mg, 84%) as a colorless oil: $[\alpha]_D^{20}$ +9.0 (*c* = 0.74, $CHCl_3$); (CHIRALPAK AD-H column) 95:5 hexane/isopropanol at 1.0 mL/min flow rate; tR (1*S*,2*R*)-enantiomer (minor) = 12.7 min., tR (1*R*,2*S*)-enantiomer (minor) = 14.2 min.; 99% *ee* (99.5% *cee*).

^{1}H NMR (500 MHz, $CDCl_3$) δ 7.38-7.29 (m, 5H), 7.25-7.21 (m, 2H), 6.87-6.85 (m, 2H), 4.85 (app. t, *J* = 3.7 Hz, 1H), 4.51 (s, 2H), 3.81 (s, 3H), 3.50-3.44 (m, 2H), 3.04 (d, *J* = 4.0 Hz, 1H), 2.18-2.11 (m, 1H), 0.89 (d, *J* = 7.0 Hz, 3H).

^{13}C NMR (125 MHz, $CDCl_3$) δ 158.79 (e), 138.20 (e), 135.18 (e), 128.61 (o), 127.89 (o), 127.83 (o), 127.42 (o), 113.57 (o), 76.19 (o), 74.17 (e), 73.61 (e), 55.40 (o), 40.45 (o), 11.62 (o).

IR (Neat) 3452 (br, w), 3064 (w), 3030 (w), 2963 (w), 2930 (w), 2904 (w), 2859 (w), 1610 (m), 1584 (w), 1510 (s), 1454 (m), 1360 (w), 1301 (w), 1245 (s), 1174 (m), 1092 (m), 1032 (m), 839 (m), 738 (m), 697 (m) cm^{-1}.

HRMS (DART) calcd. for $C_{18}H_{23}O_3$ $[M+H]^+$: 287.1642, found: 287.1648.

11. Experimental Procedures for the Preparation and Characterization Data for (*R*)-Cyclamen Aldehyde (10)

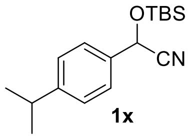

1x

2-((*tert*-Butyldimethylsilyl)oxy)-2-(4-isopropylphenyl)acetonitrile (1x). Cyanohydrin **1x** was prepared (255.1 mg, 88%) as a colorless oil from cuminaldehyde **8** on a 1.0 mmol scale using the general experimental procedure for cyanohydrin synthesis.

^{1}H NMR (500 MHz, $CDCl_3$) δ 7.40-7.38 (m, 2H), 7.28-7.26 (m, 2H), 5.50 (s, 1H), 2.93 (app. septet, *J* = 6.8 Hz, 1H), 1.26 (d, *J* = 6.9 Hz, 6H), 0.95 (s, 9H), 0.23 (s, 3H), 0.15 (s, 3H).

^{13}C NMR (125 MHz, $CDCl_3$) δ 150.28 (e), 134.04 (e), 127.11 (o), 126.28 (o), 119.58 (e), 64.05 (o), 34.05 (o), 25.71 (o), 24.04 (o), 18.34 (e), –4.92 (o), –5.01 (o).

IR (Neat) 2960 (w), 2930 (w), 2885 (w), 2859 (w), 1610 (w), 1513 (w), 1465 (w), 1416 (w), 1265 (m), 1088 (m), 835 (s), 779 (s), 675 (m) cm^{-1}.

HRMS (DART) calcd. for $C_{17}H_{31}N_2OSi$ $[M+NH_4]^+$: 307.2200, found: 307.2202.

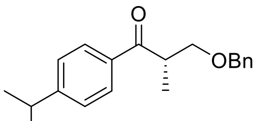

3xe

(*S*)-3-(Benzyloxy)-1-(4-isopropylphenyl)-2-methylpropan-1-one (3xe). Chiral ketone **3xe** was prepared (210.5 mg, 81% yield) as colorless oil from cyanohydrin **1x** and (*R*)-**2e** using the general experimental procedure (Method A) for the alkylation of chiral non-racemic secondary tosylates: $[\alpha]_D^{20}$ +12.4 (*c* = 1.03, $CHCl_3$); (CHIRALPAK AD-H column) 99:0.7:0.3 hexane/isopropanol/methanol at 0.6 mL/min flow rate; tR (*S*)-enantiomer (major) = 19.1 min., tR = (*R*)-enantiomer (minor) = 22.4 min.; ≥97.5% *ee* (98% *cee*).

^{1}H NMR (500 MHz, $CDCl_3$) δ 7.94-7.91 (m, 2H), 7.33-7.24 (m, 7H), 4.53 (d, A of an AB quartet, J_{AB} = 12.2 Hz, 1H), 4.49 (d, B of an AB quartet, J_{AB} = 12.1 Hz, 1H), 3.86-3.78 (m, 2H), 3.56-3.54 (m, 1H), 2.97 (septet, J = 6.9 Hz, 1H), 1.28 (d, J = 6.8 Hz, 6H), 1.23 (d, J = 6.2 Hz, 3H).

^{13}C NMR (125 MHz, $CDCl_3$) δ 202.49 (e), 154.62 (e), 138.45 (e), 134.72 (e), 128.82 (o), 128.45 (o), 127.69 (o), 127.65 (o), 126.82 (o), 73.47 (e), 72.82 (e), 41.48 (o), 34.38 (o), 23.81 (o), 15.12 (o).

IR (Neat) 3086 (w), 3060 (w), 3030 (w), 2960 (m), 2930 (w), 2866 (w), 2796 (w), 1677 (s), 1603 (m), 1569 (w), 1495 (w), 1454 (m), 1416 (m), 1364 (m), 1249 (m), 1219 (m), 1185 (m), 1096 (s), 1055 (m), 973 (s), 846 (m), 734 (s), 697 (s) cm^{-1}.

HRMS (DART) calcd. for $C_{20}H_{25}O_2$ $[M+H]^+$: 297.1849, found: 297.1848.

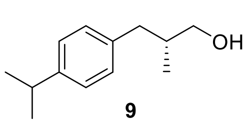

9

(*R*)-3-(4-Isopropylphenyl)-2-methylpropan-1-ol (9). To a solution of (*S*)-3-(benzyloxy)-1-(4-isopropylphenyl)-2-methylpropan-1-one (**3xe**, 150 mg, 0.51 mmol) in methanol (5 mL) was added palladium on carbon (Pd/C, 30 mg, 10 wt% Pd, wet). The suspension was stirred at room temperature under hydrogen (balloon) for *ca.* 16 h. Aqueous perchloric acid (70 wt%, 0.1 mL) was then added, and the mixture was stirred for an additional 2 days (monitored by TLC). The reaction was quenched with solid K_2CO_3 (*ca.* 200 mg), and the suspension was filtered through Celite. The filtrate was concentrated *in vacuo* to afford the crude product. Purification by flash column chromatography (silica gel, eluting with 20-40% diethyl ether/hexane) afforded the chiral alcohol (*R*)-**9** (73.5 mg, 75%) as a colorless oil. The characterization data are identical to those reported:[41] $[\alpha]_D^{20}$ +10.2 (*c* = 0.66, $CHCl_3$); (CHIRALPAK AD-H column) 98:1:1 hexane/isopropanol/methanol at 0.6 mL/min flow rate; tR (*R*)-enantiomer (major) = 11.8 min., tR = (*S*)-enantiomer (minor) = 12.8 min.; 98% *ee* (100% *cee*).

^{1}**H NMR** (500 MHz, $CDCl_3$) δ 7.16-7.13 (m, 2H), 7.11-7.09 (m, 2H), 3.54 (dd, *J* = 10.5, 5.8 Hz, 1H), 3.47 (dd, *J* = 10.5, 6.2 Hz, 1H), 2.88 (septet, *J* = 6.9 Hz, 1H), 2.71 (dd, *J* = 13.5, 6.6 Hz, 1H), 2.41 (dd, *J* = 13.5, 7.9 Hz, 1H), 1.94 (app. octet, *J* = 6.6 Hz, 1H), 1.24 (d, *J* = 7.0 Hz, 6H), 0.93 (d, *J* = 6.7 Hz, 3H).

IR (Neat) 3351 (br, w), 3090 (w), 3049 (w), 3008 (w), 2960 (m), 2926 (m), 2870 (m), 1510 (m), 1457 (m), 1416 (w), 1383 (w), 1364 (w), 1282 (w), 1178 (w), 1100 (w), 1066 (w), 1032 (s), 984 (w), 846 (m), 798 (m), 667 (w), 652 (w) cm^{-1}.

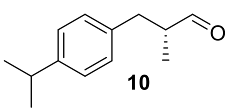

10

(*R*)-3-(4-Isopropylphenyl)-2-methylpropanal ((*R*)-Cyclamen Aldehyde) (10). Dess-Martin periodinane (DMP, 229.9 mg, 0.54 mmol, 1.4 equiv.) was added to a stirred solution of (*R*)-3-(4-isopropylphenyl)-2-methylpropan-1-ol (**9**, 73.5 mg, 0.38 mmol) in DCM (3.8 mL) at 0 °C. The reaction mixture was allowed to warm to room temperature and stirred for *ca.* 15 min (monitored by TLC). The reaction was quenched with saturated aqueous $NaHCO_3$ solution, and the biphasic mixture was extracted with dichloromethane. The combined organic layers were washed with saturated aqueous $Na_2S_2O_3$ solution, dried (anhyd. $MgSO_4$, filtered, and concentrated *in vacuo* to afford the crude product. Purification by flash column chromatography (silica gel, eluting with 5-15% diethyl ether/hexane) afforded the chiral (*R*)-cyclamen aldehyde **10** in (62.1 mg, 86%) as a colorless oil. The characterization data are identical to those reported:[41,50] $[\alpha]_D^{20}$ –5.8 (*c* = 2.1, $CHCl_3$), lit. $[\alpha]_D^{21}$ –7.0 (*c* = 2.1, $CHCl_3$).

^{1}**H NMR** (500 MHz, $CDCl_3$) δ 9.72 (d, *J* = 1.0 Hz, 1H), 7.17-7.14 (m, 2H), 7.10-7.08 (m, 2H), 3.05 (dd, *J* = 13.8, 5.9 Hz, 1H), 2.88 (septet, *J* = 6.8 Hz, 1H), 2.66 (sextet, *J* = 6.9 Hz, 1H), 2.58 (dd, *J* = 13.6, 8.2 Hz, 1H), 1.24 (d, *J* = 6.9 Hz, 6H), 1.09 (d, *J* = 7.0 Hz, 3H).

^{13}**C NMR** (125 MHz, $CDCl_3$) δ 204.71 (o), 147.14 (e), 136.21 (e), 129.07 (o), 126.71 (o), 48.23 (o), 36.46 (e), 33.85 (o), 24.16 (o), 13.44 (o).

IR (Neat) 3049 (w), 3012 (w), 2960 (m), 2926 (m), 2870 (w), 2807 (w), 2710 (w), 1726 (s), 1513 (m), 1457 (m), 1420 (w), 1364 (w), 1260 (w), 1114 (w), 1051 (w), 1018 (w), 924 (w), 839 (m), 798 (m) cm^{-1}.

HRMS (DART) calcd. for $C_{13}H_{22}NO$ $[M+NH_4]^+$: 208.1696, found: 208.1691.

12. Copies of Proton and Carbon NMR Spectra

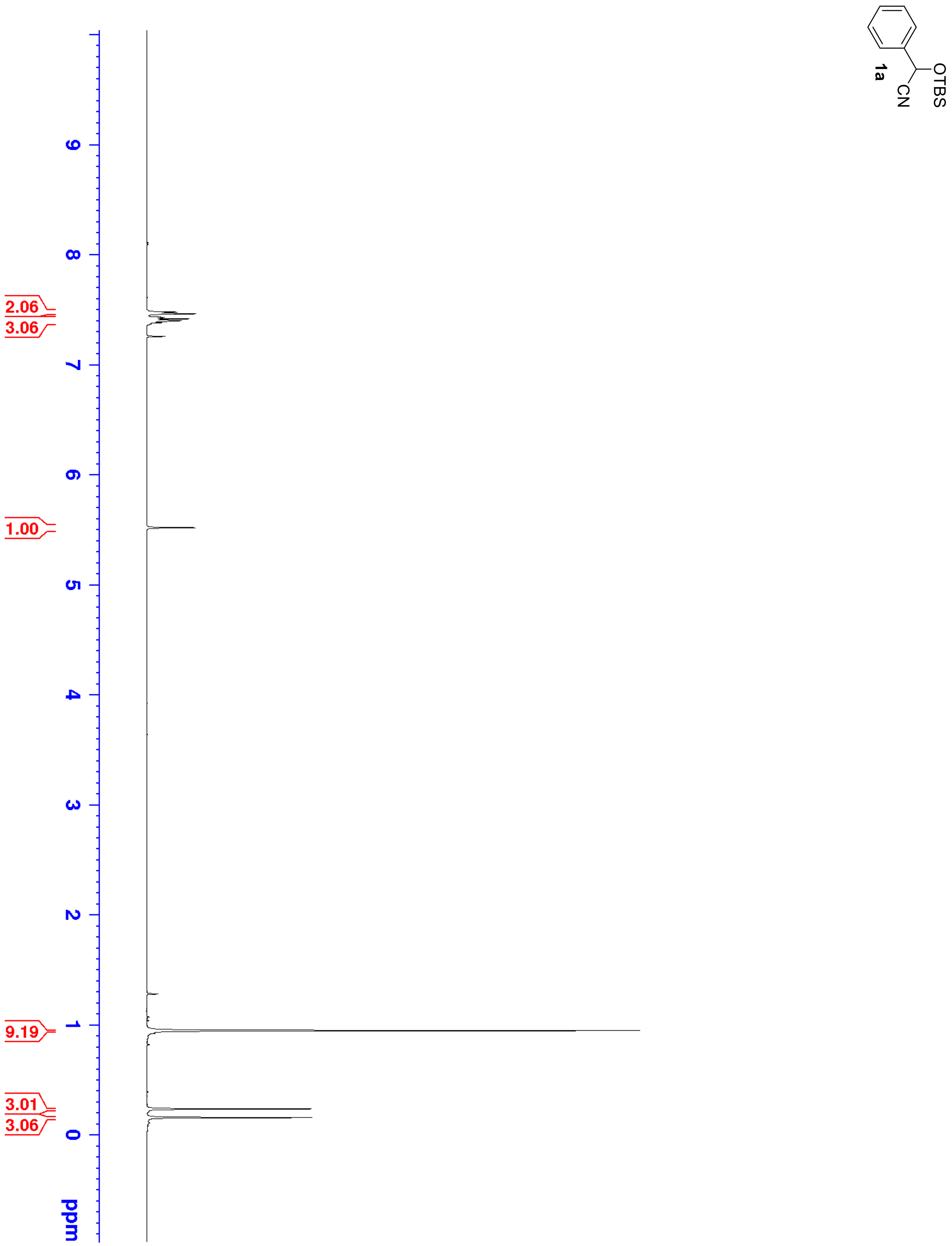

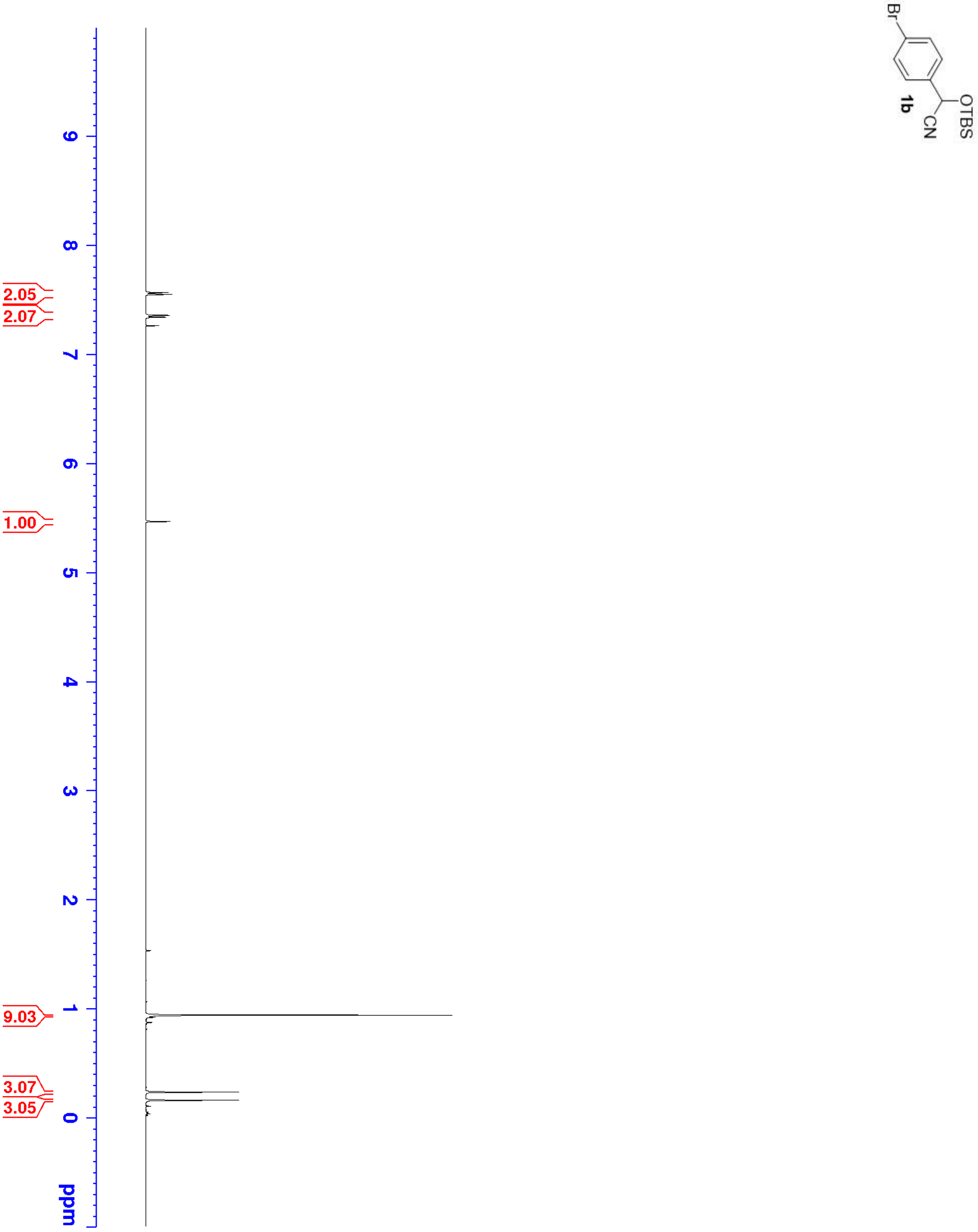

Br
OTBS
CN
1b
ppm
2.05
2.07
1.00
9.03
3.07
3.05

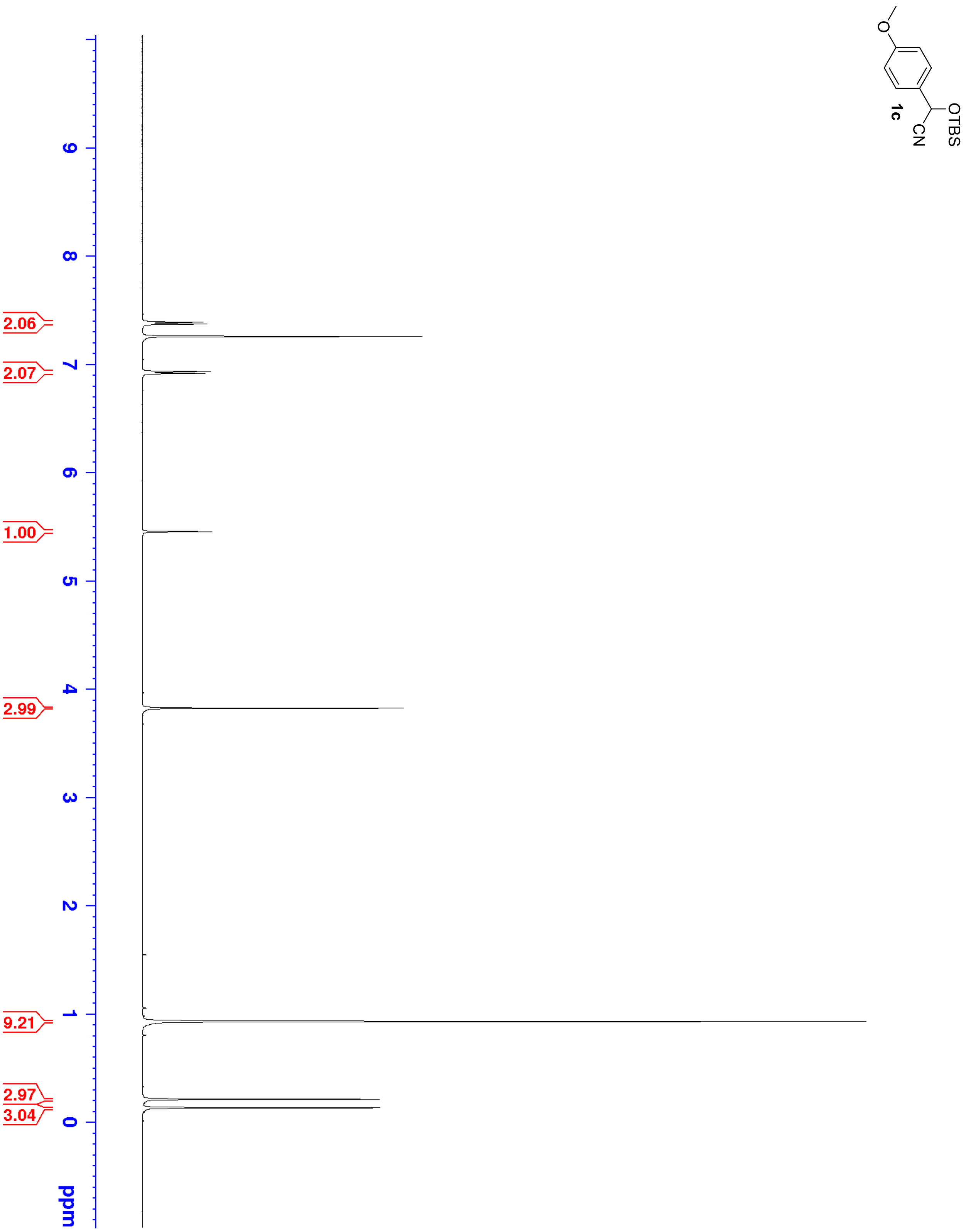

OTBS
CN
1c
2.06
2.07
1.00
2.99
9.21
2.97
3.04
9
8
7
6
5
4
3
2
1
0
ppm

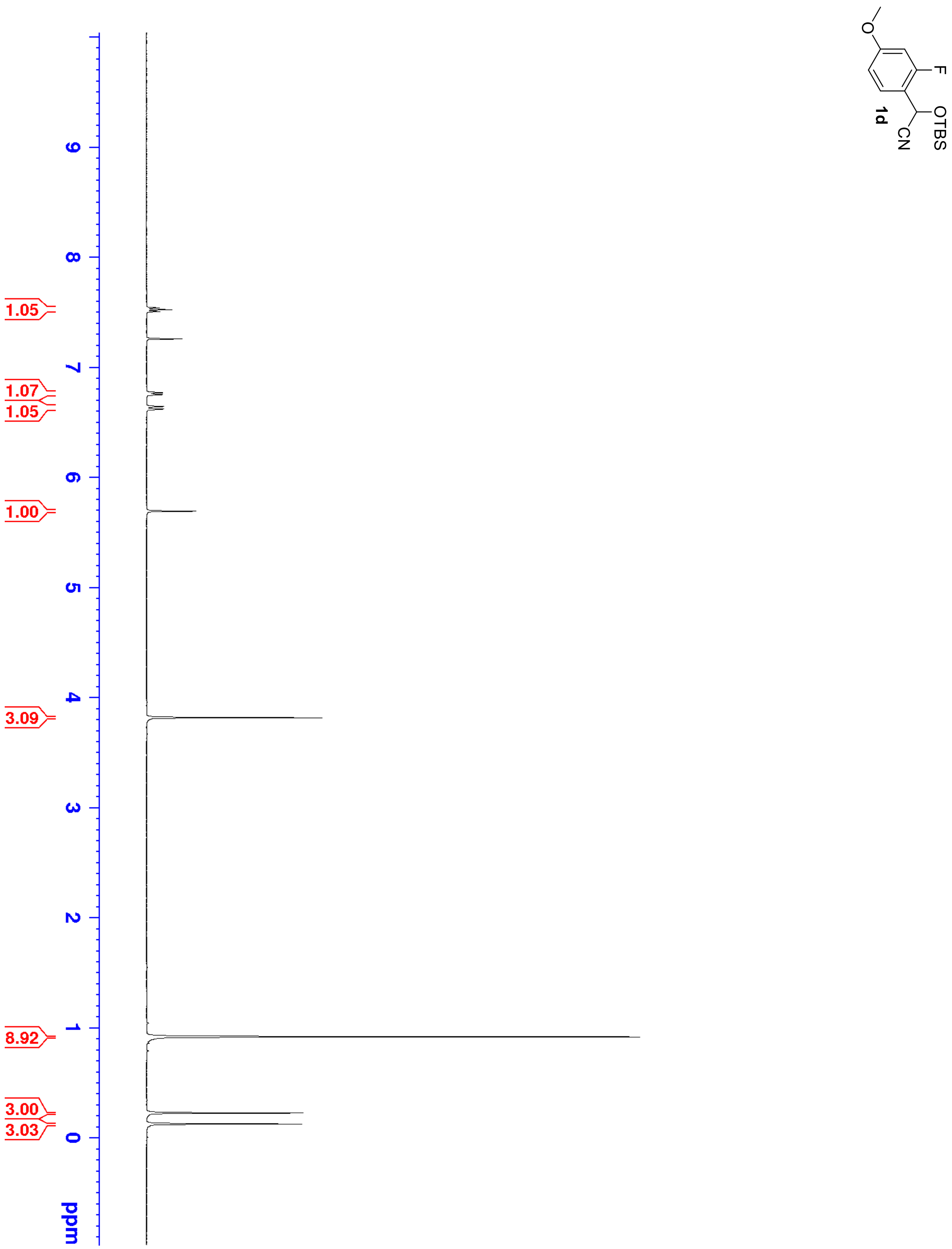
O
F
OTBS
CN
1d
9
8
7
6
5
4
3
2
1
0
ppm
1.05
1.07
1.05
1.00
3.09
8.92
3.00
3.03

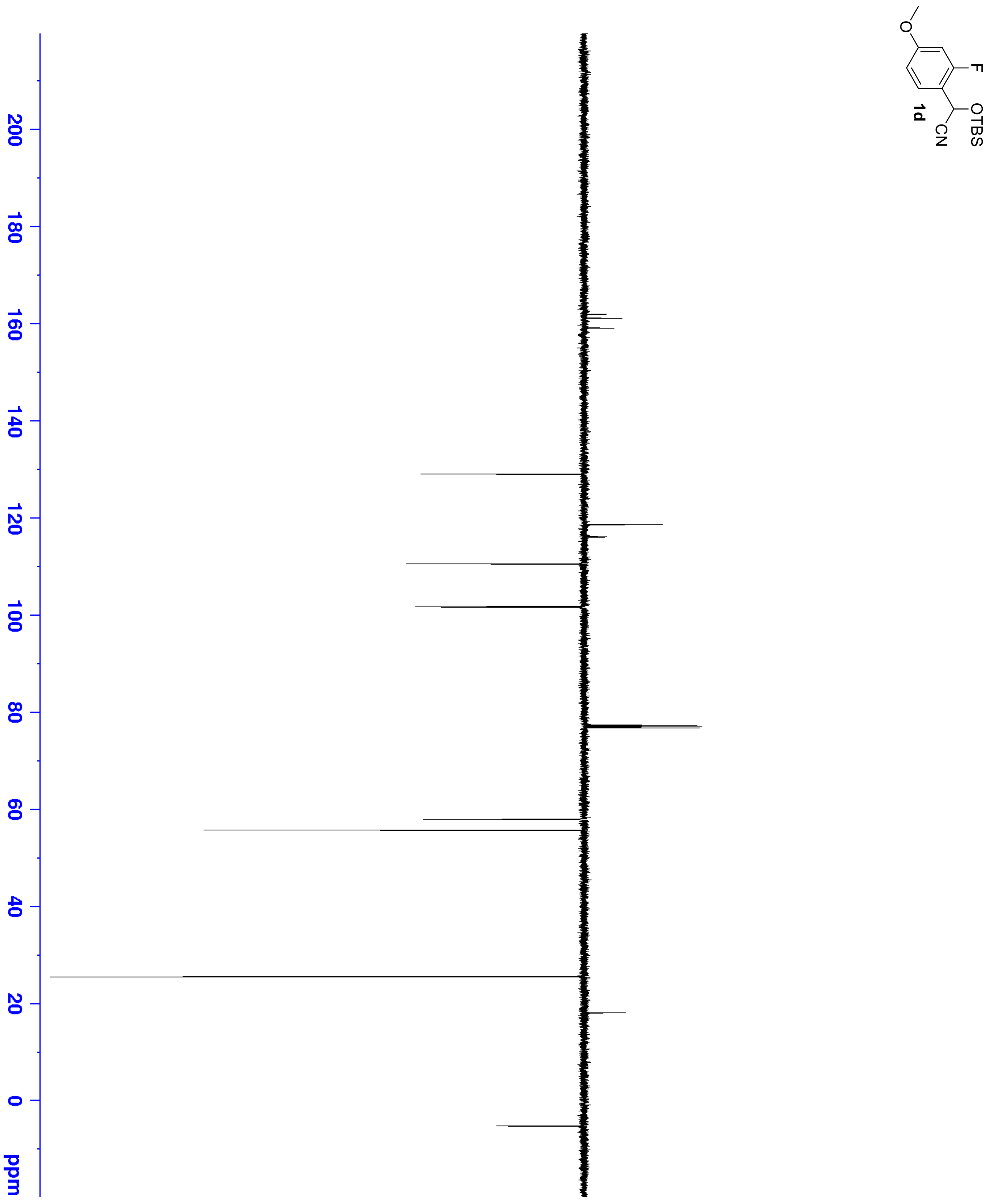
F
OTBS
CN
O
1d
200
180
160
140
120
100
80
60
40
20
0
ppm

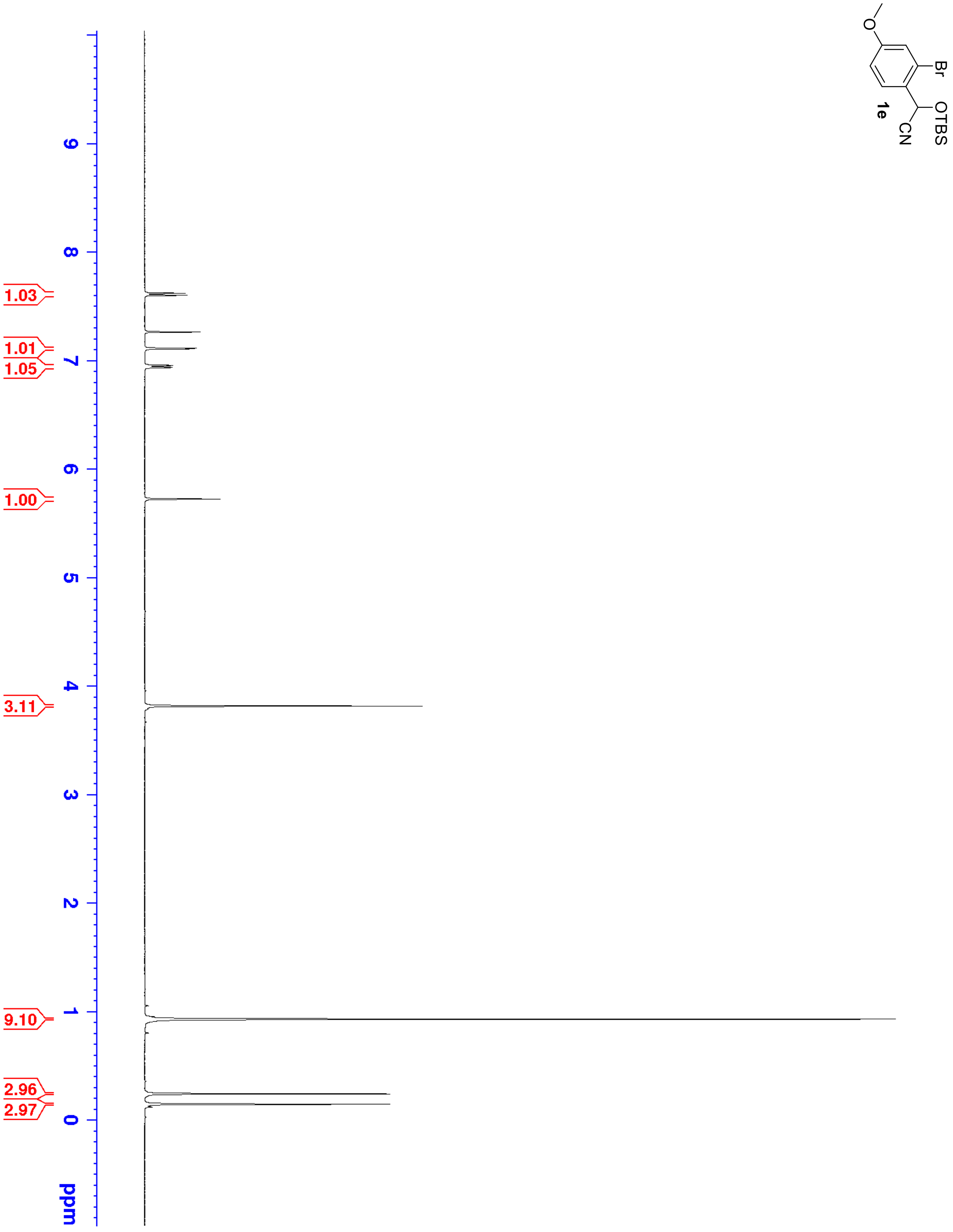

OTBS
CN
Br
O
1e
1.03
1.01
1.05
1.00
3.11
9.10
2.96
2.97
9
8
7
6
5
4
3
2
1
0
ppm

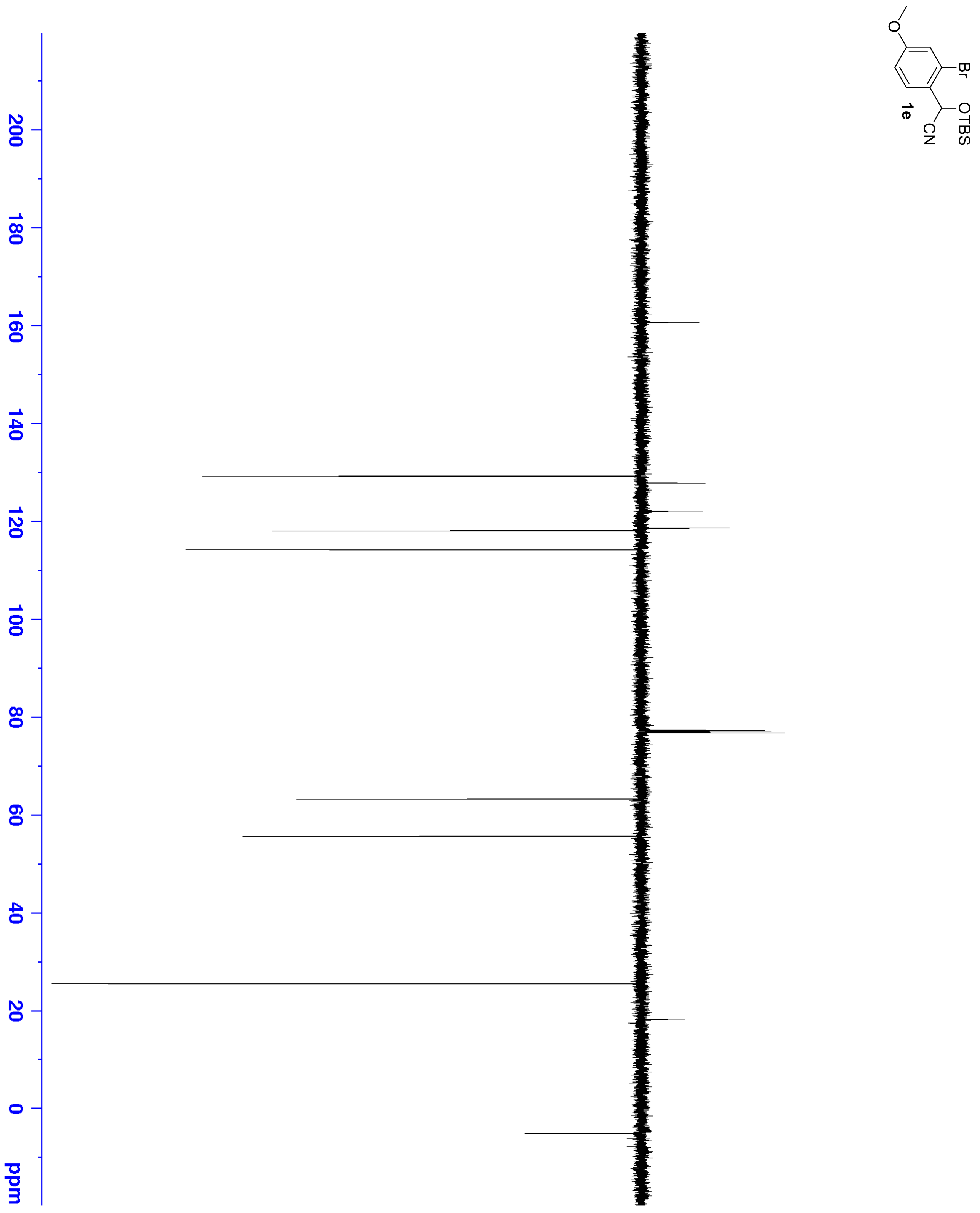
Br
OTBS
CN
1e
200
180
160
140
120
100
80
60
40
20
0
ppm

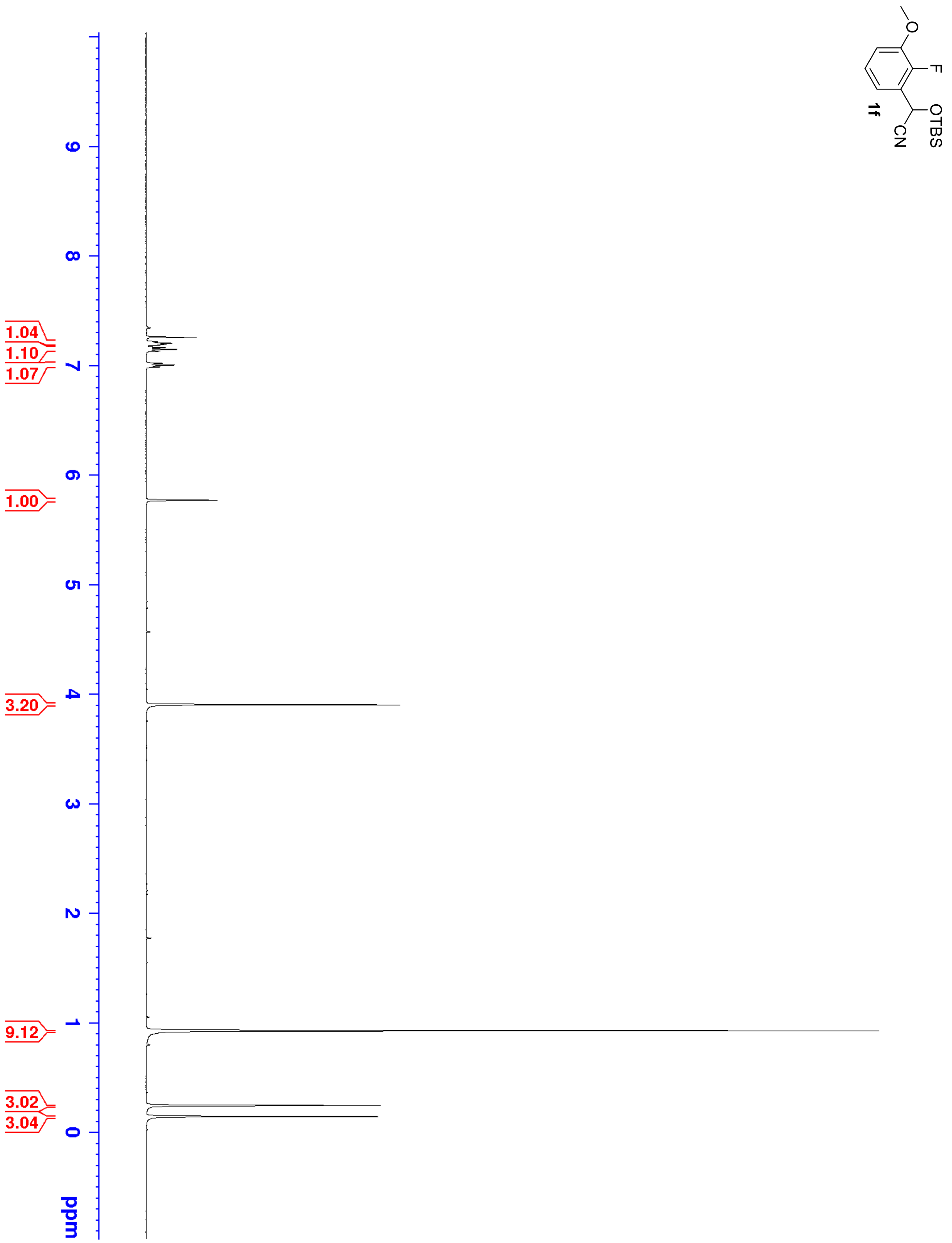
OTBS
CN
F
1f
1.04
1.10
1.07
1.00
3.20
9.12
3.02
3.04
9
8
7
6
5
4
3
2
1
0
ppm

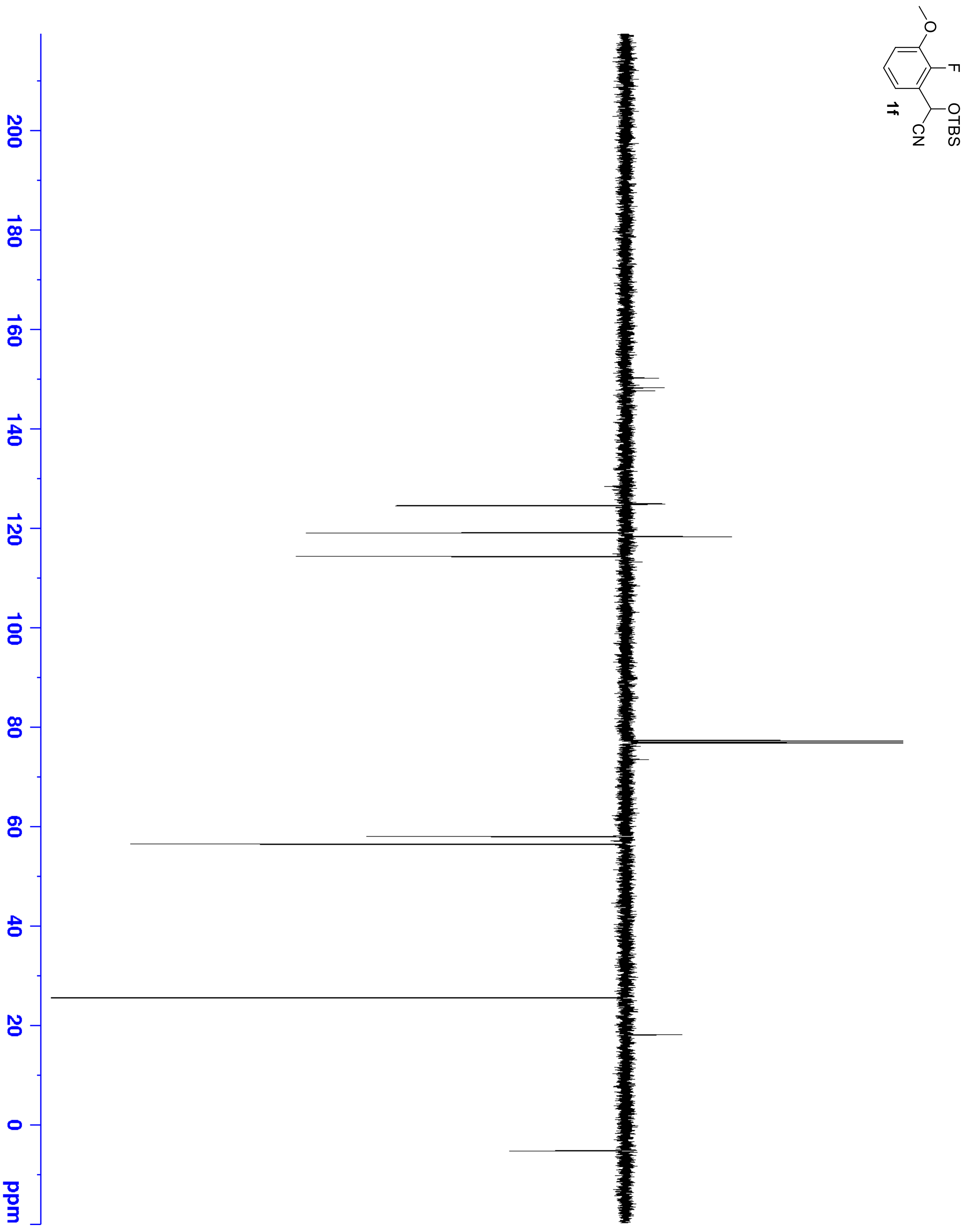
OTBS
CN
F
O
1f
200
180
160
140
120
100
80
60
40
20
0
ppm

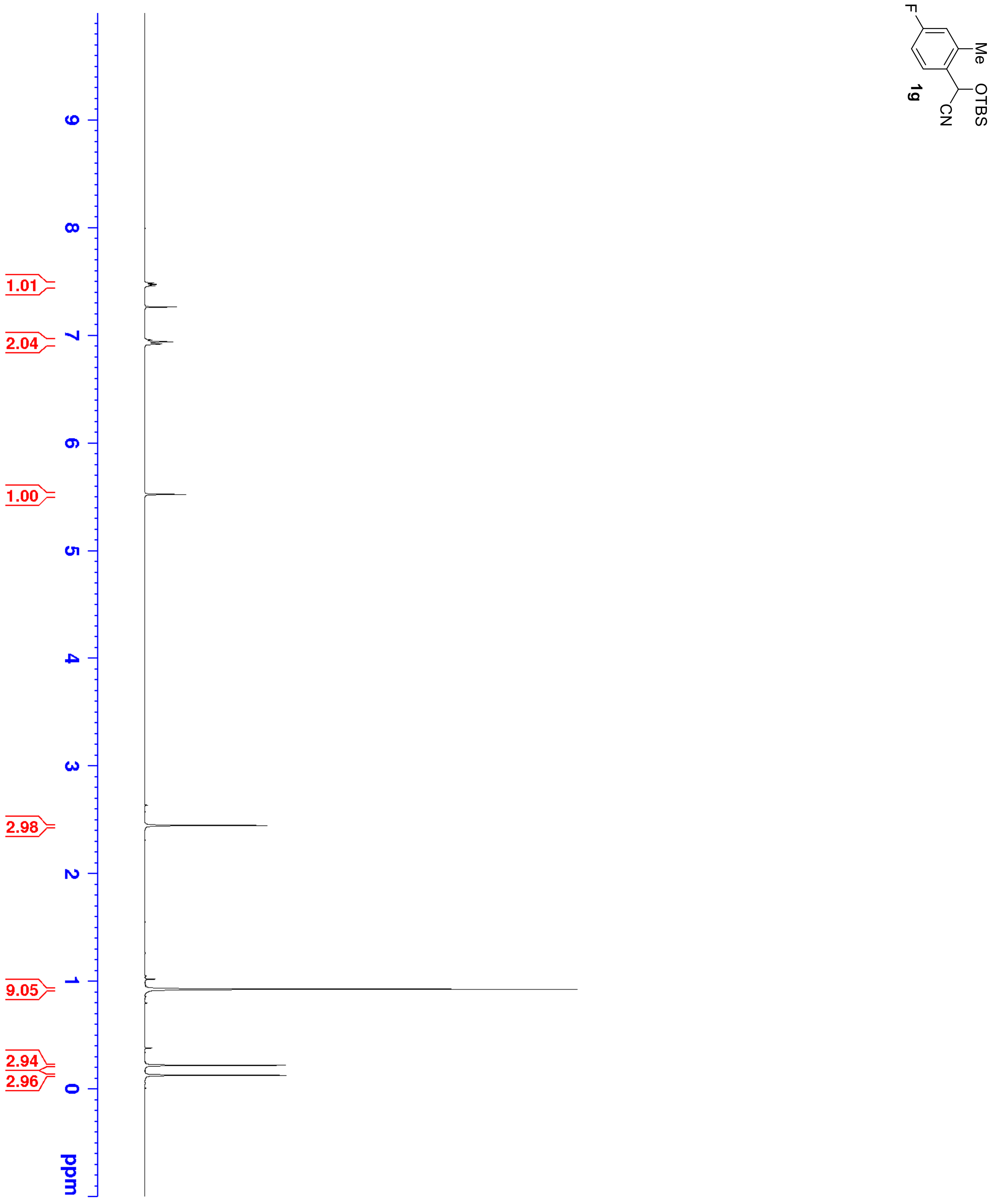
F
Me
OTBS
CN
1g
1.01
2.04
1.00
2.98
9.05
2.94
2.96
9
8
7
6
5
4
3
2
1
0
ppm

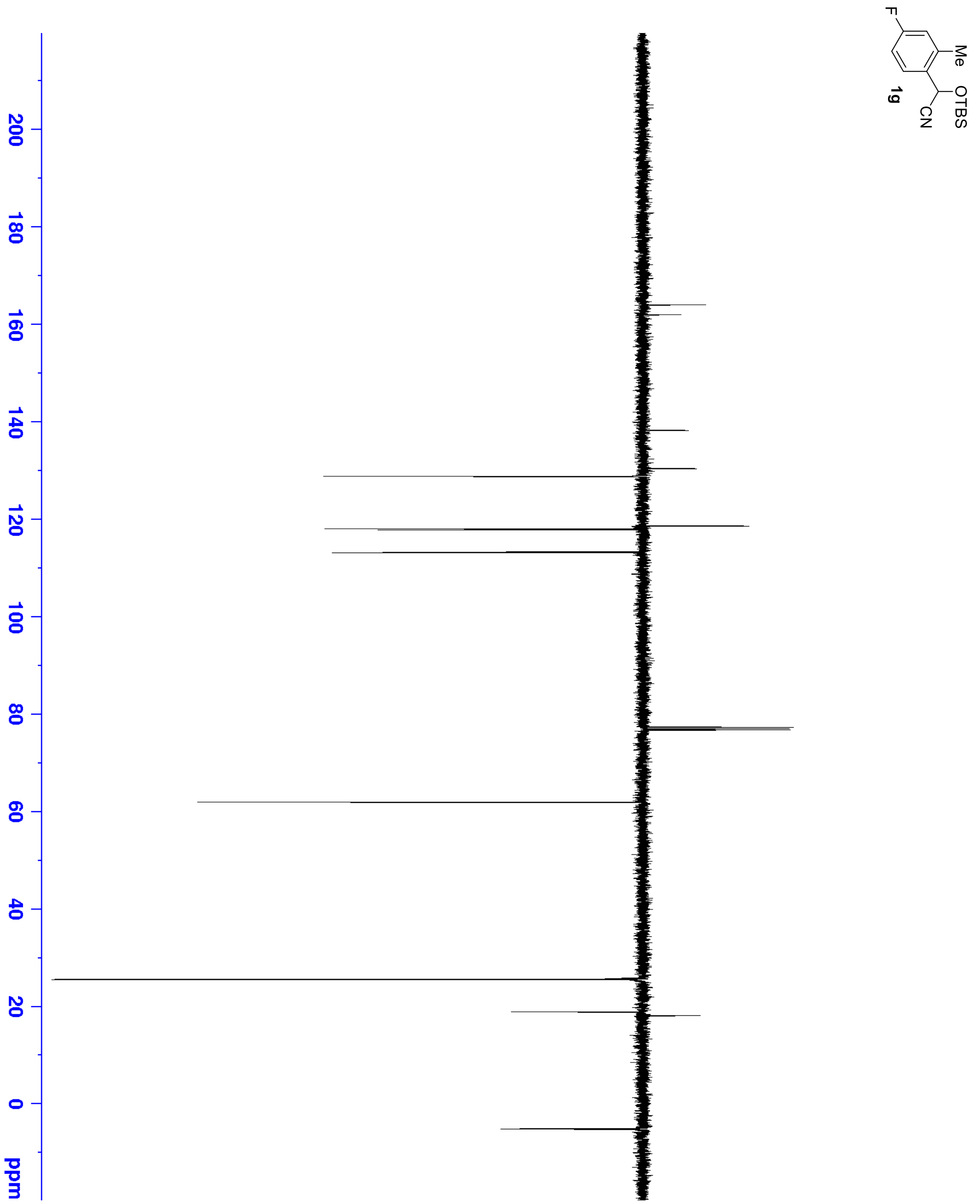
F
Me
OTBS
CN
1g
200
180
160
140
120
100
80
60
40
20
0
ppm

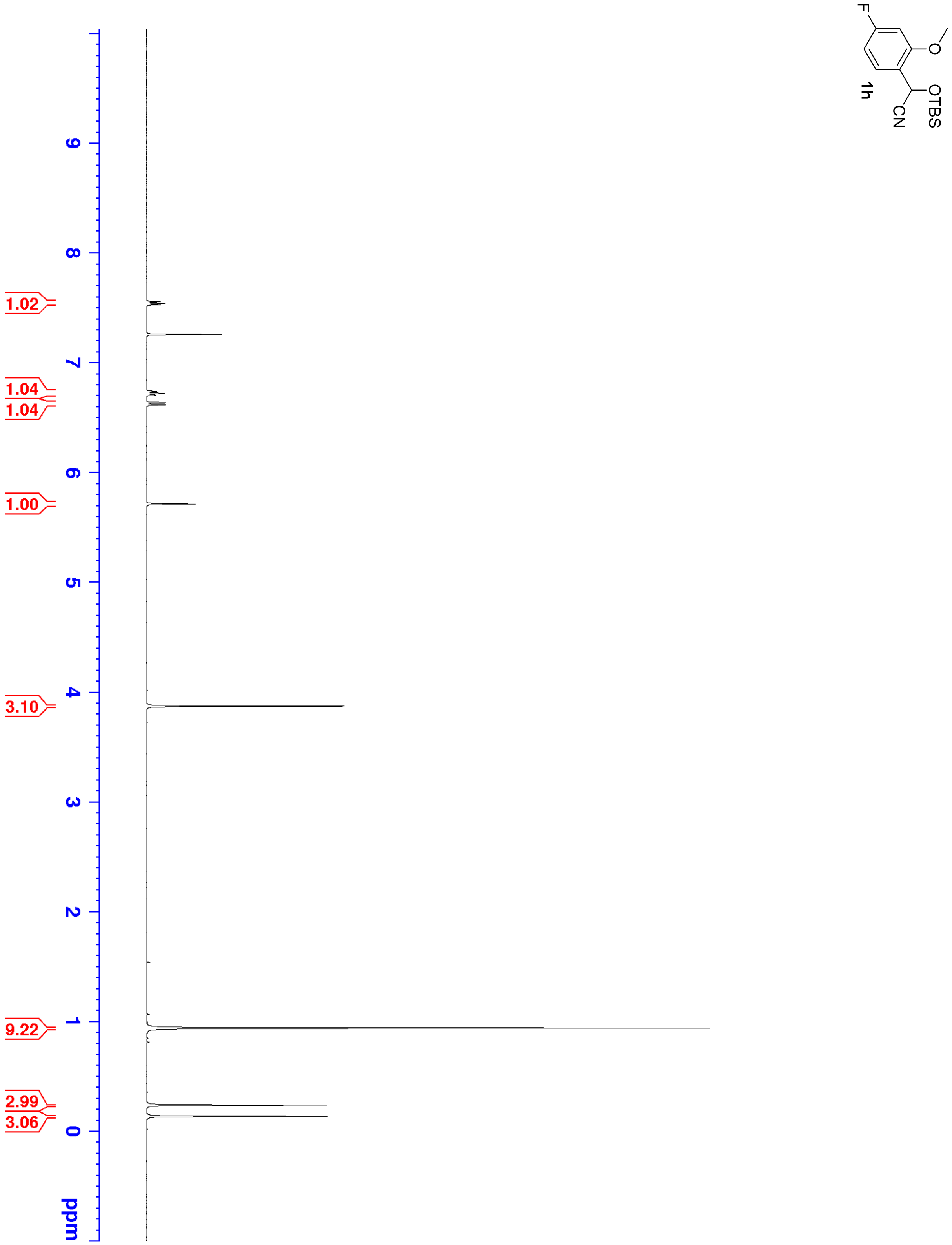
F
O
OTBS
CN
1h
9
8
7
6
5
4
3
2
1
0
ppm
1.02
1.04
1.04
1.00
3.10
9.22
2.99
3.06

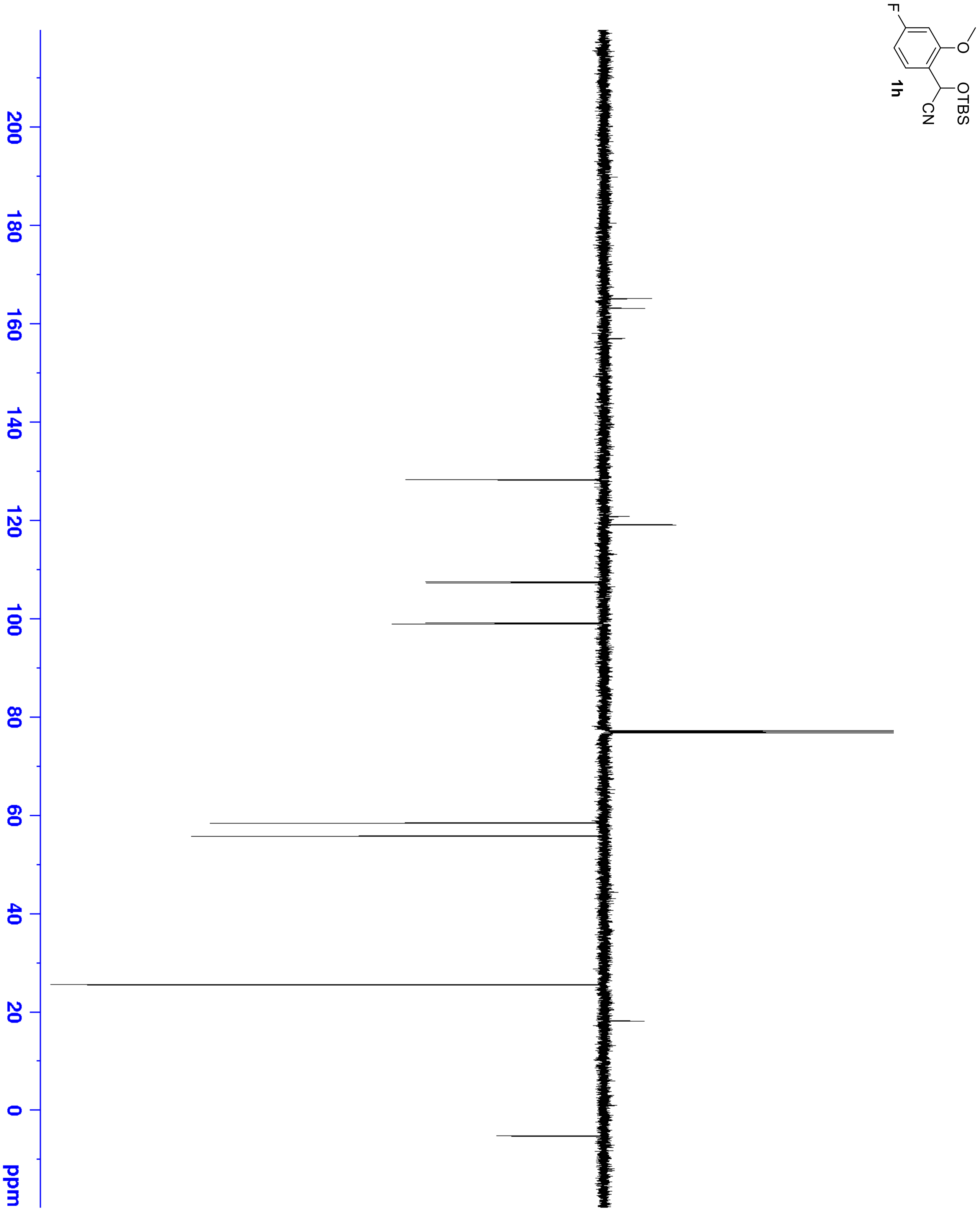

OTBS
CN
O
F
1h
200
180
160
140
120
100
80
60
40
20
0
ppm

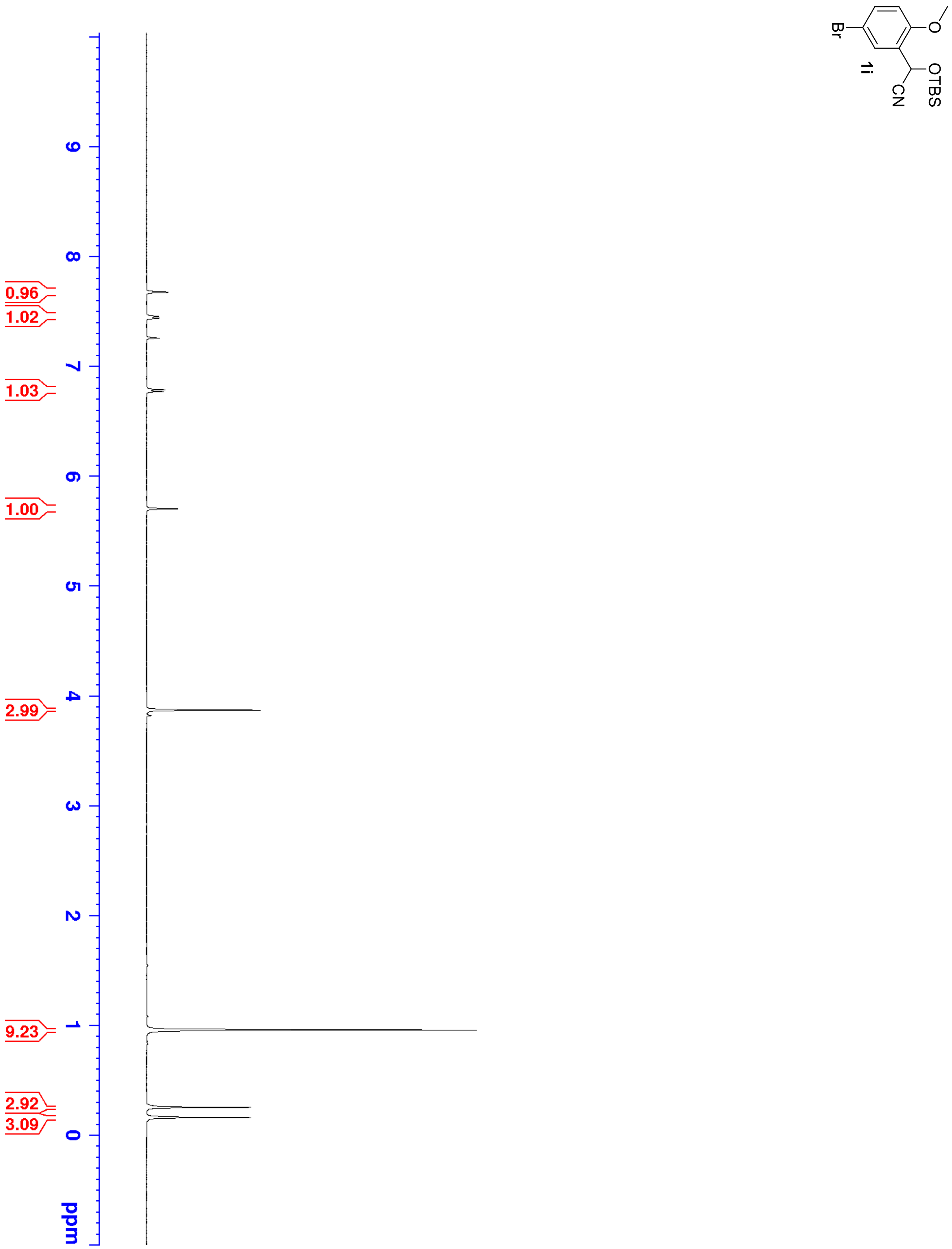
OTBS
CN
O
Br
1i
0.96
1.02
1.03
1.00
2.99
9.23
2.92
3.09
9
8
7
6
5
4
3
2
1
0
ppm

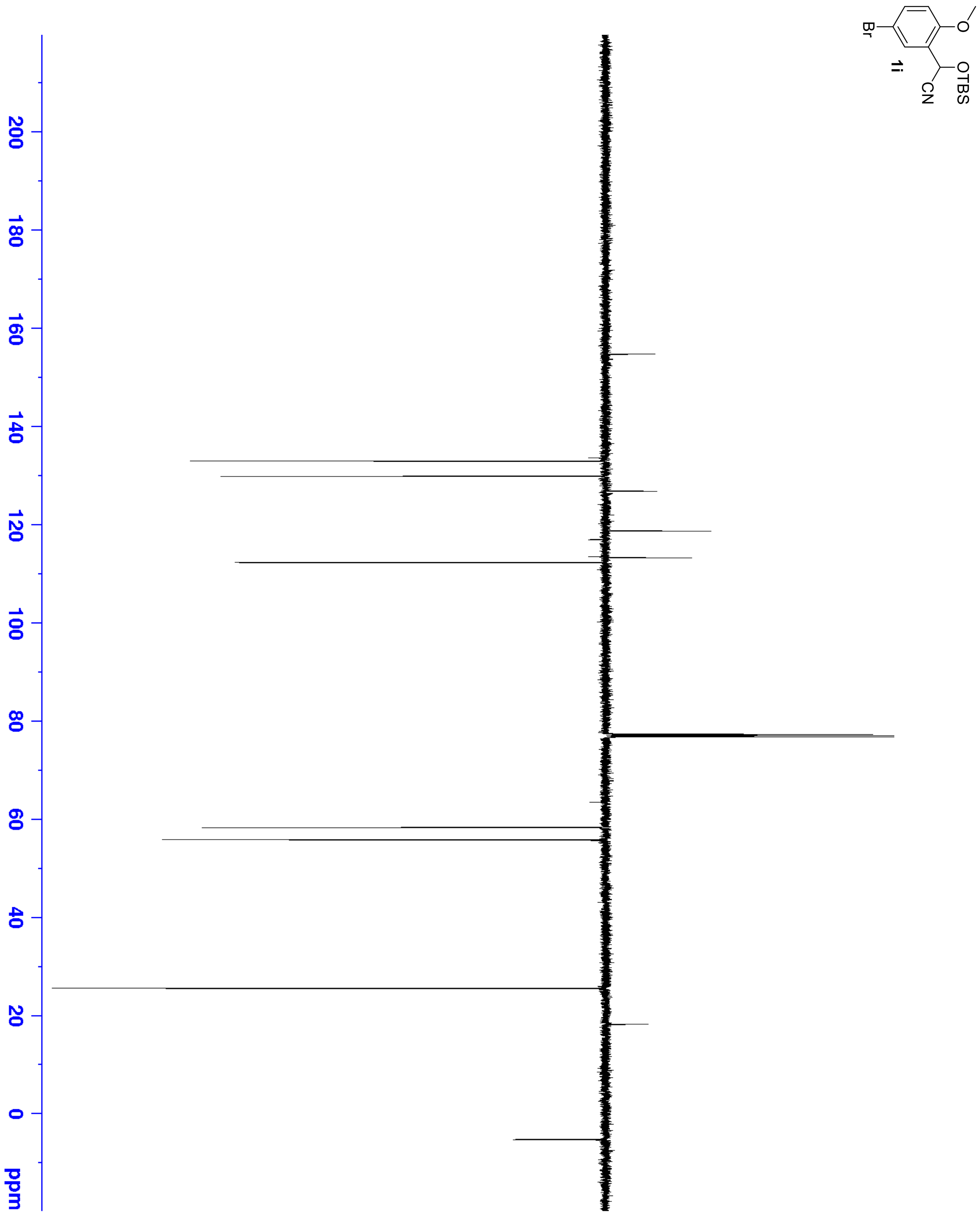
OTBS
CN
Br
O
1i
200
180
160
140
120
100
80
60
40
20
0
ppm

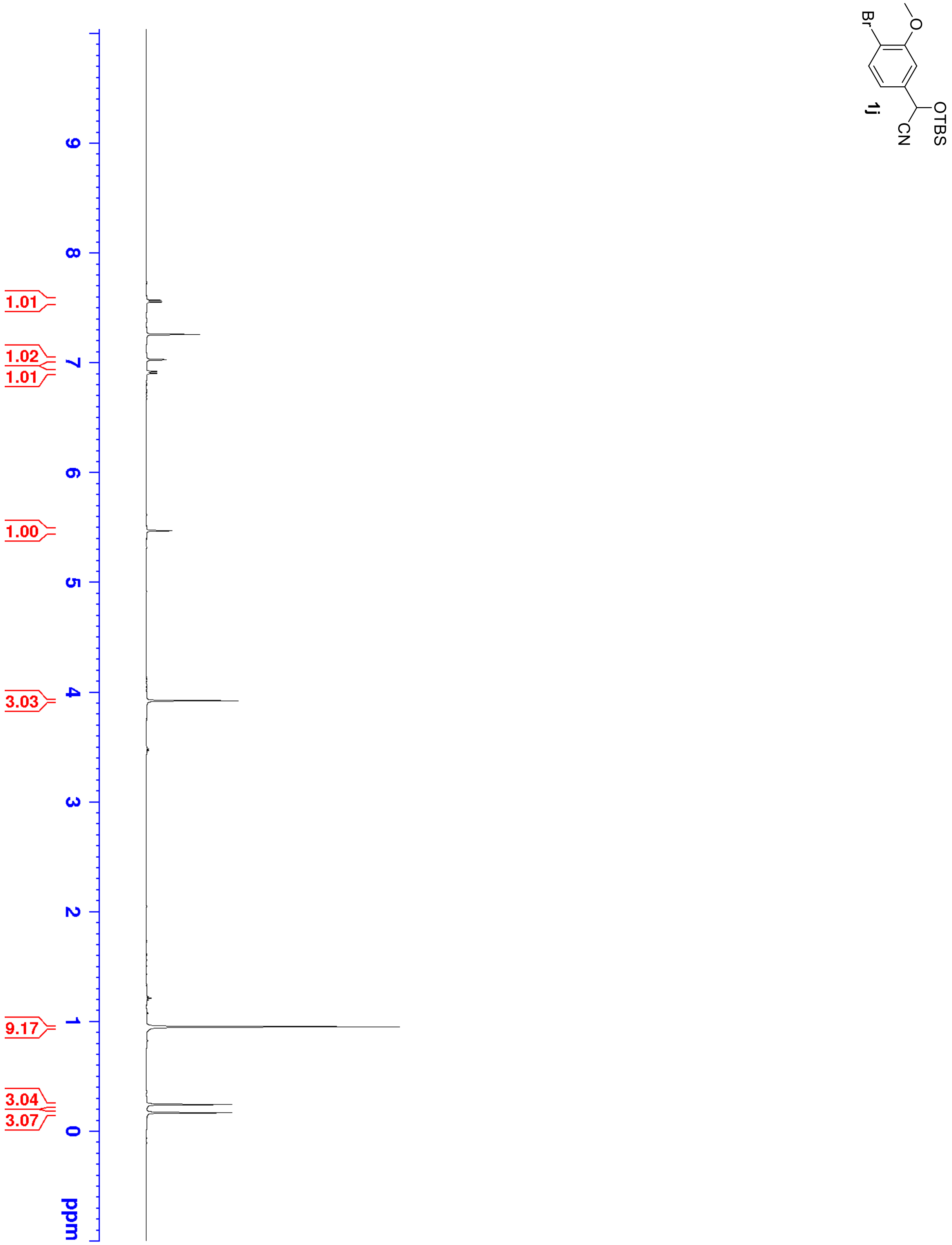

OTBS
CN
Br
O
1j
1.01
1.02
1.01
1.00
3.03
9.17
3.04
3.07
9
8
7
6
5
4
3
2
1
0
ppm

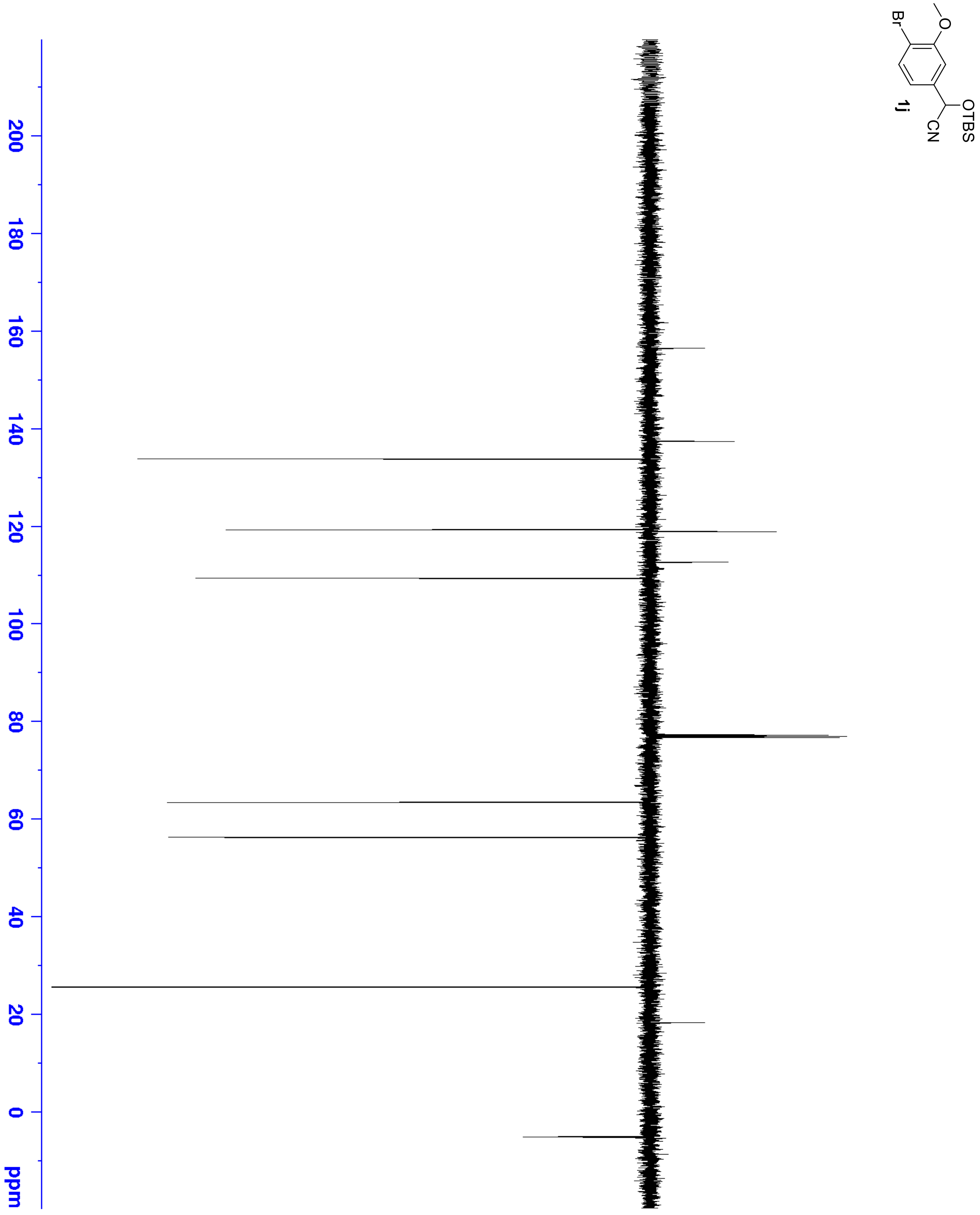

OTBS
CN
Br
O
1j
200
180
160
140
120
100
80
60
40
20
0
ppm

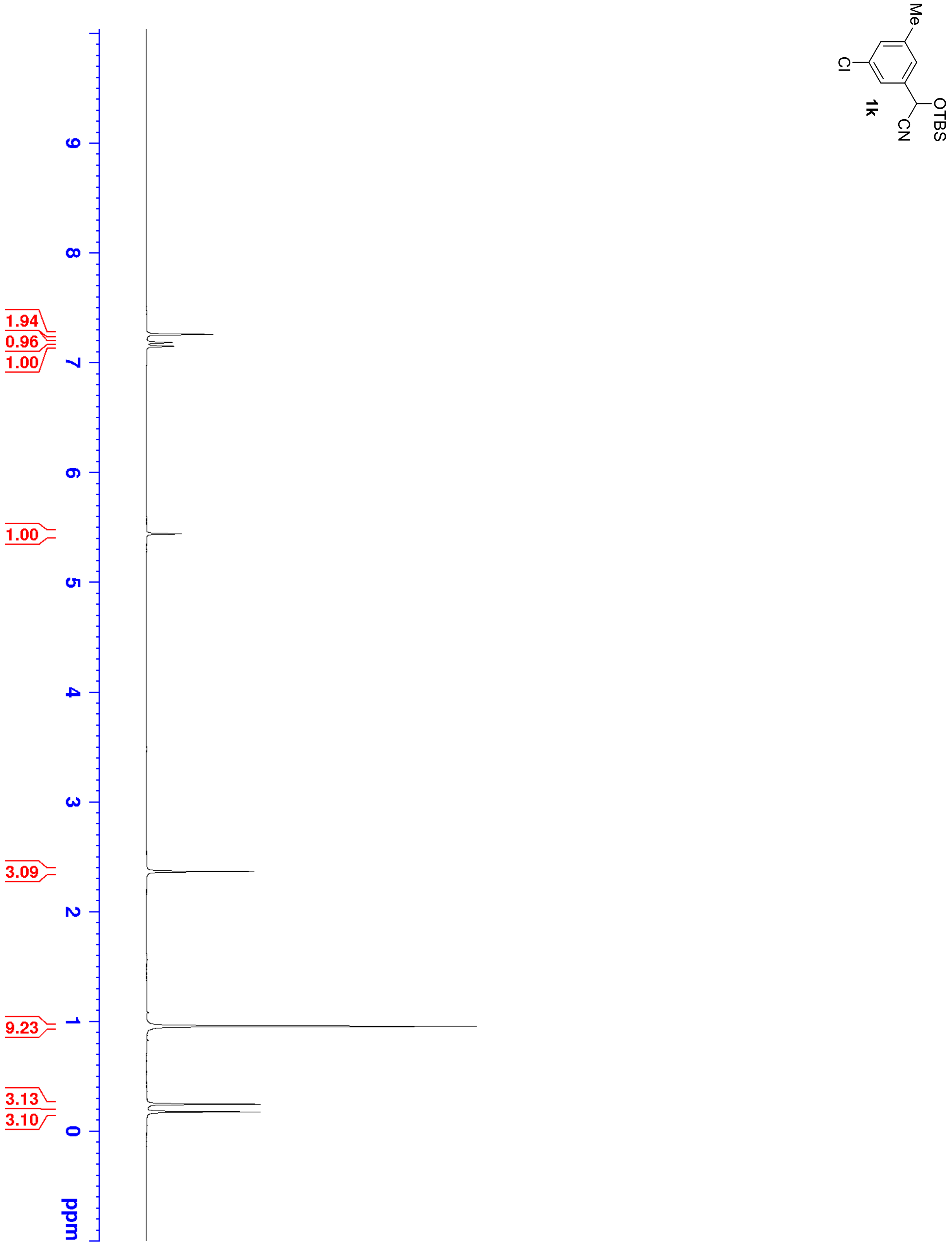

Me
Cl
OTBS
CN
1k
9
8
7
6
5
4
3
2
1
0
ppm
1.94
0.96
1.00
1.00
3.09
9.23
3.13
3.10

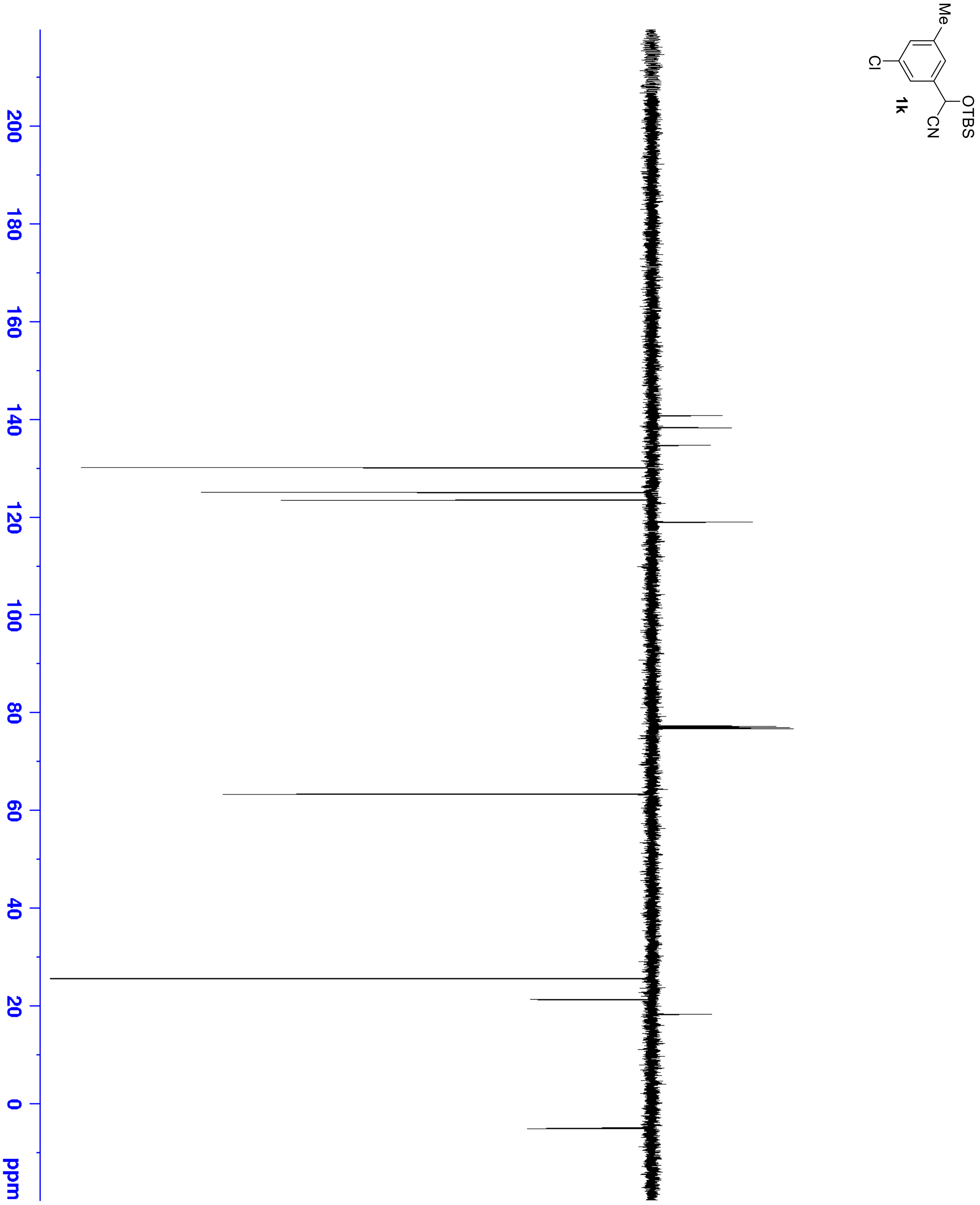

Me
Cl
OTBS
CN
1k
200
180
160
140
120
100
80
60
40
20
0
ppm

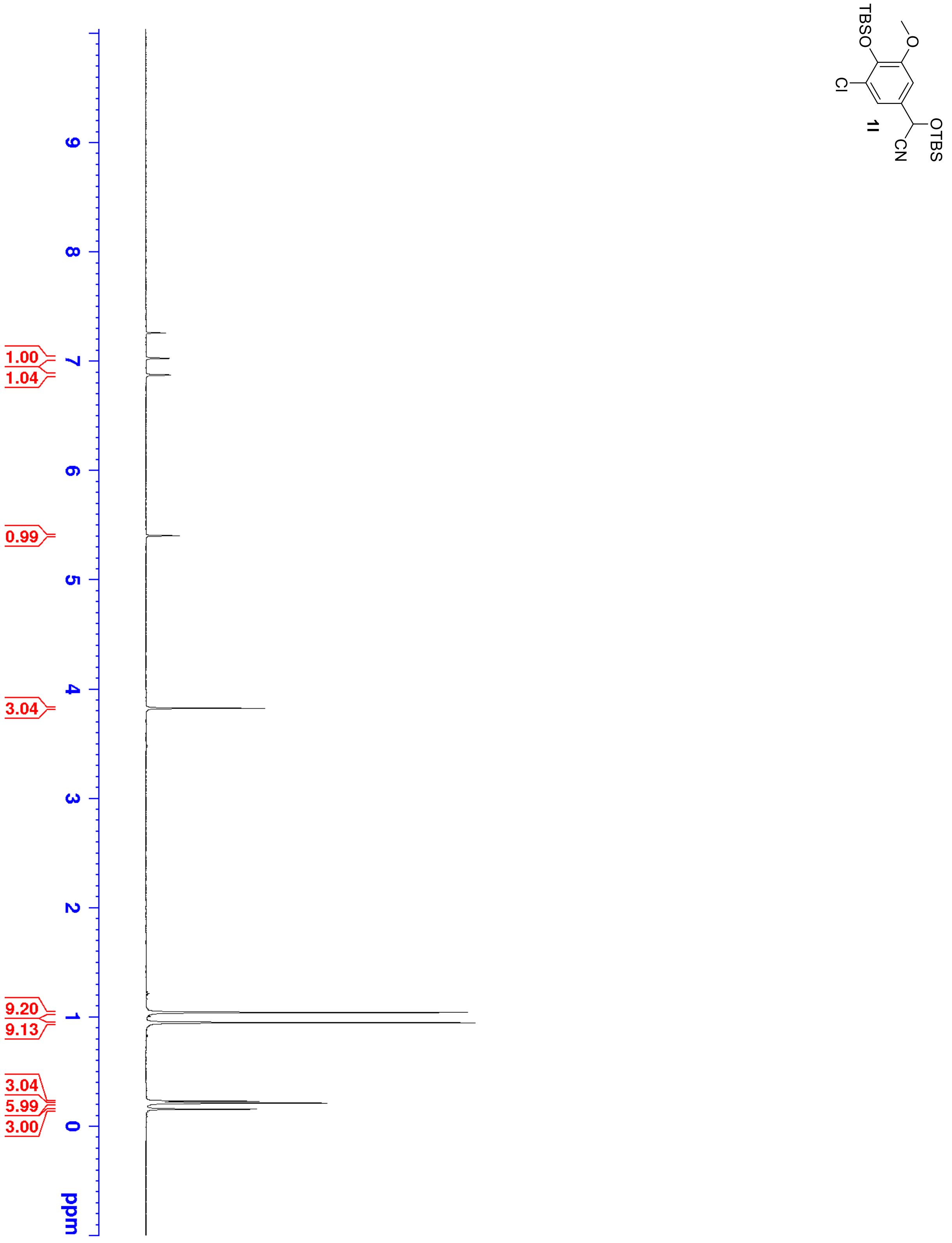
TBSO
O
Cl
OTBS
CN
1l
1.00
1.04
0.99
3.04
9.20
9.13
3.04
5.99
3.00
9
8
7
6
5
4
3
2
1
0
ppm

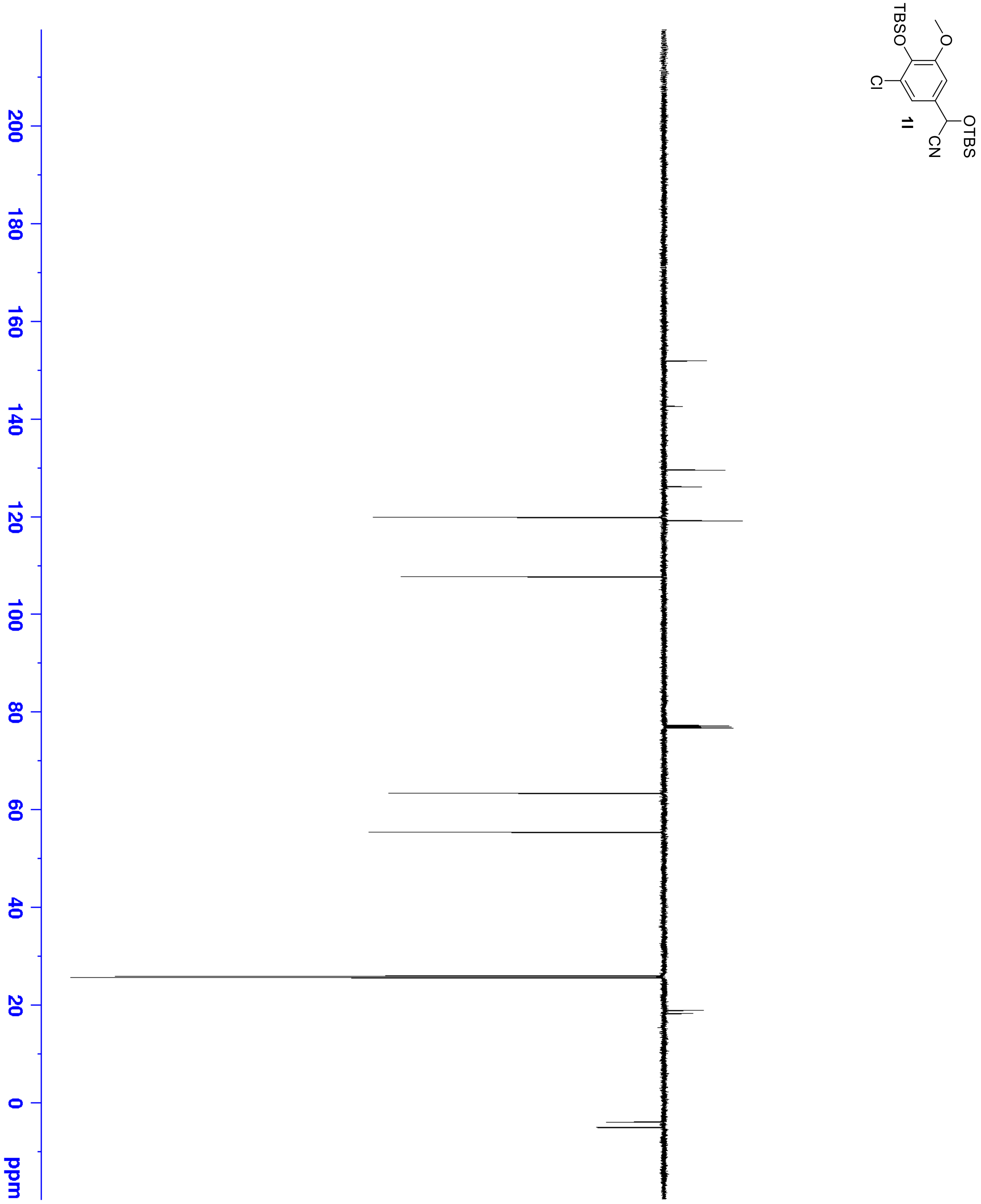

TBSO
Cl
OTBS
CN
1l
200
180
160
140
120
100
80
60
40
20
0
ppm

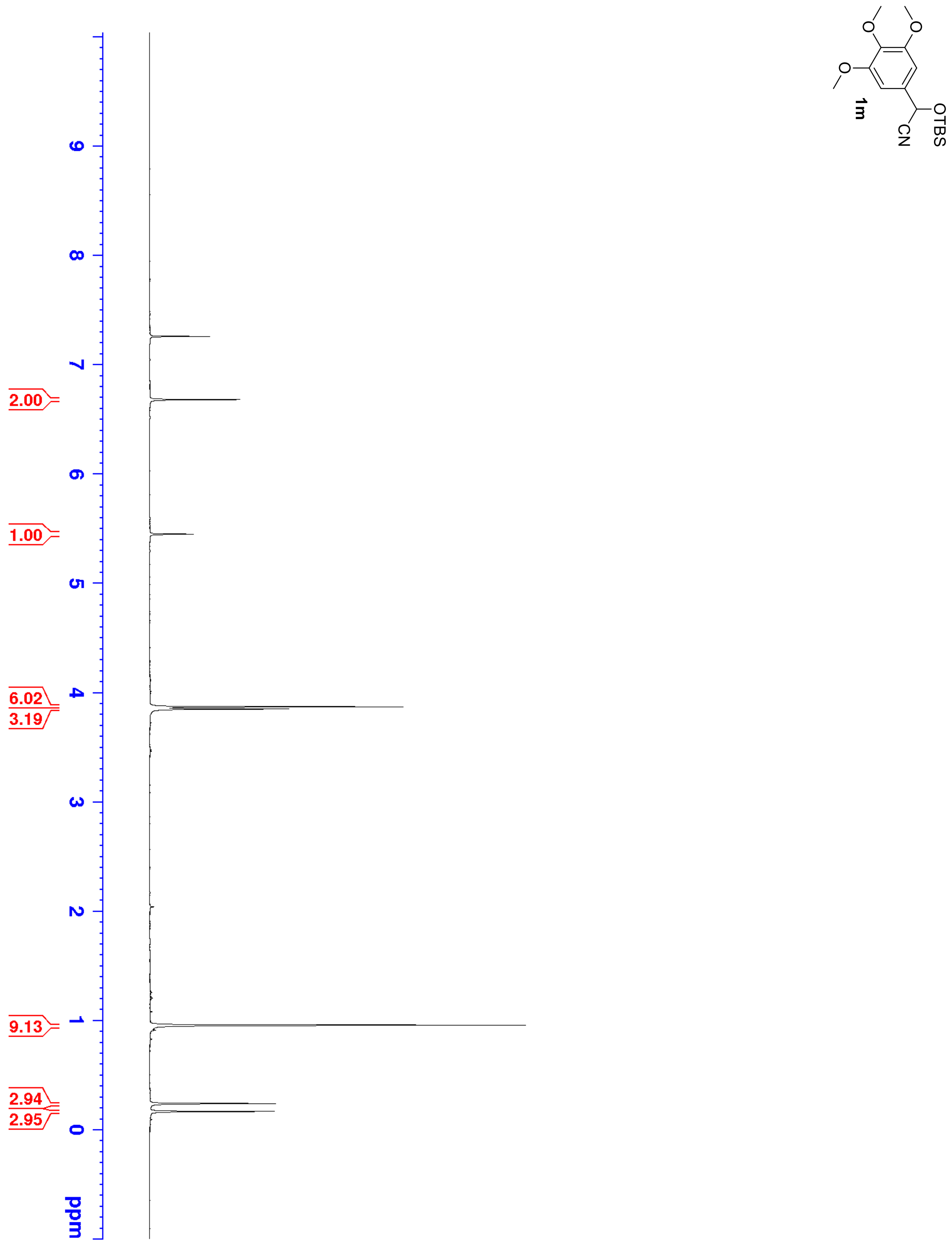
OTBS
CN
1m
2.00
1.00
6.02
3.19
9.13
2.94
2.95
ppm

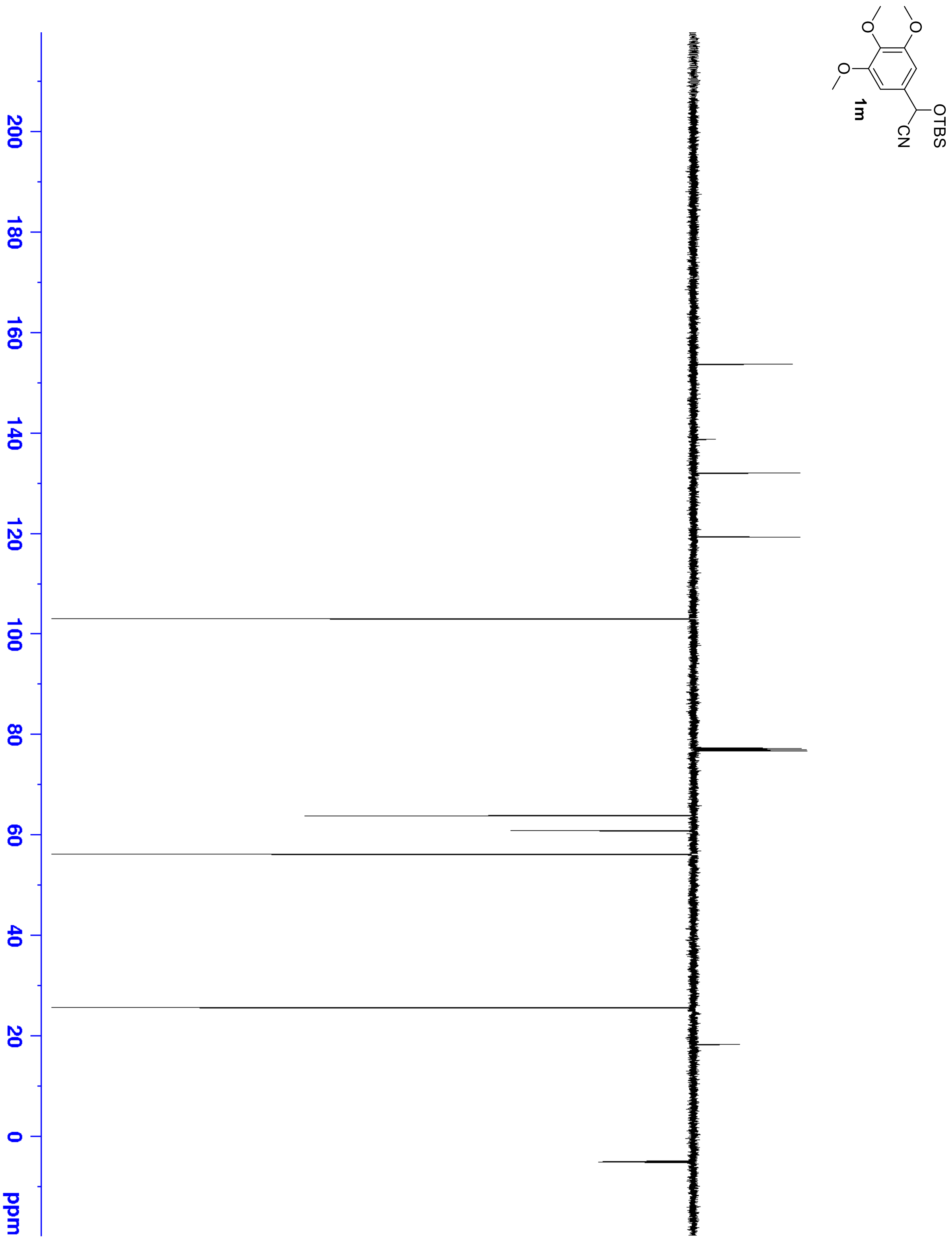
OTBS
CN
1m
200
180
160
140
120
100
80
60
40
20
0
ppm

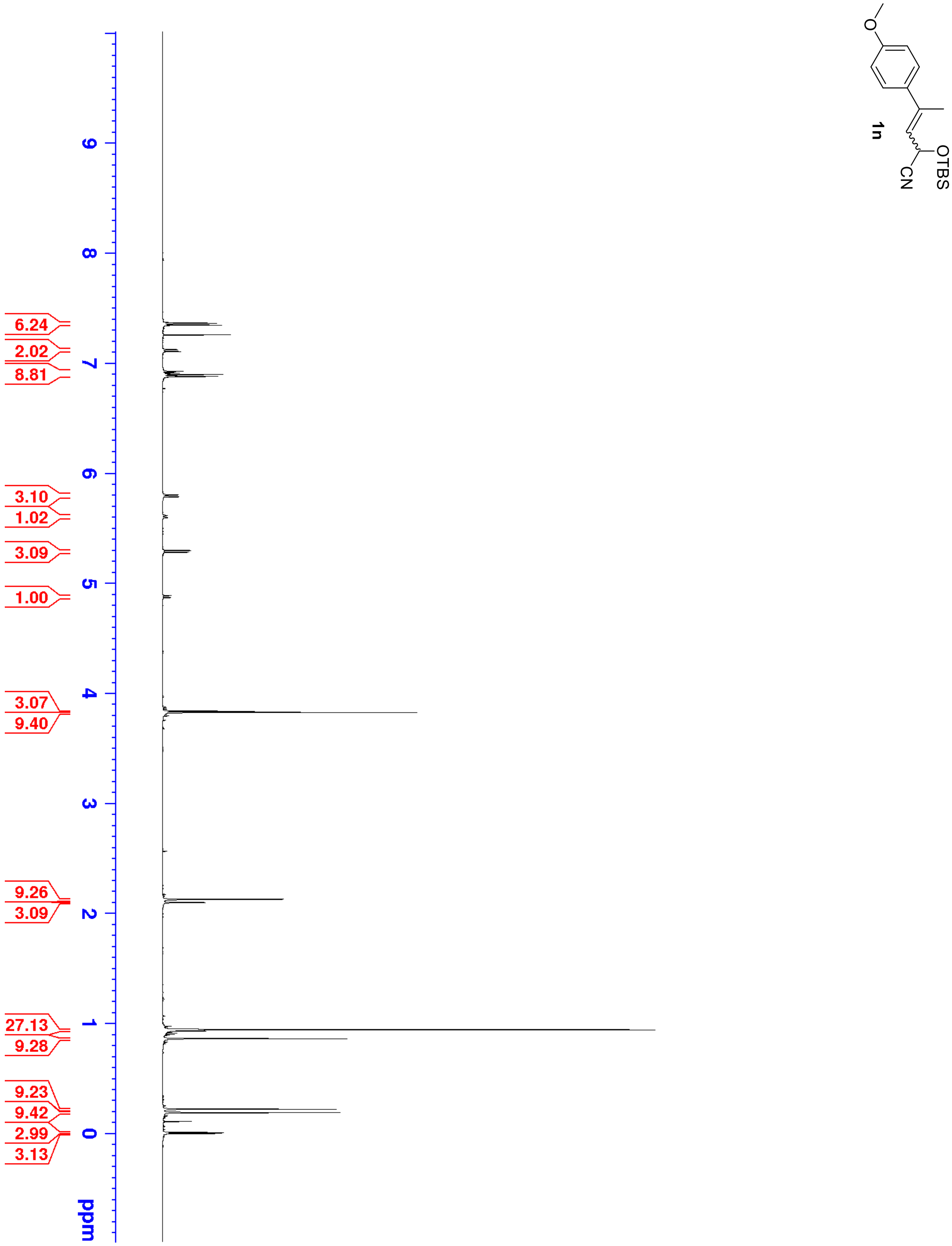

OTBS
CN
1n
6.24
2.02
8.81
3.10
1.02
3.09
1.00
3.07
9.40
9.26
3.09
27.13
9.28
9.23
9.42
2.99
3.13
ppm

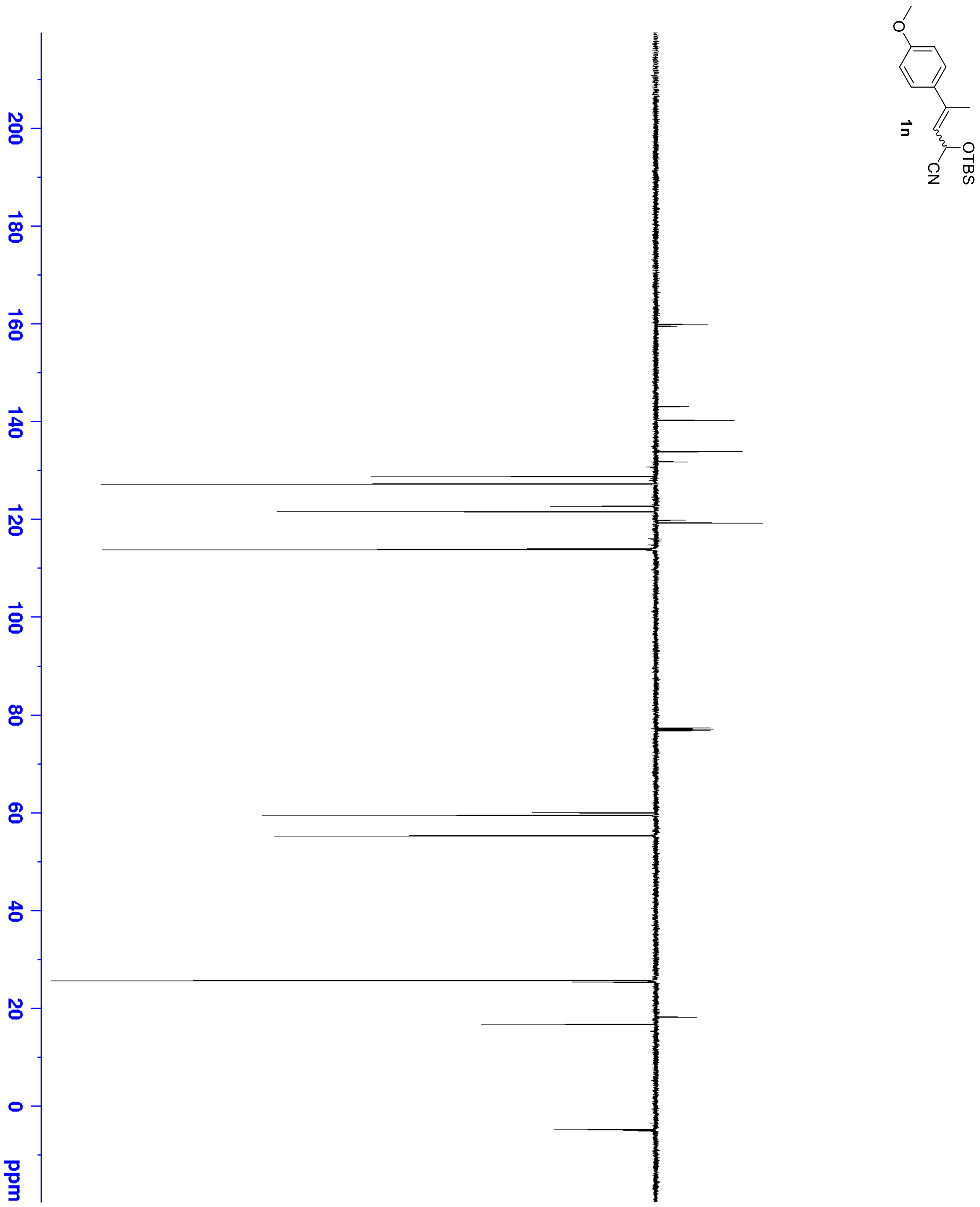

1n
OTBS
CN
O
200
180
160
140
120
100
80
60
40
20
0
ppm

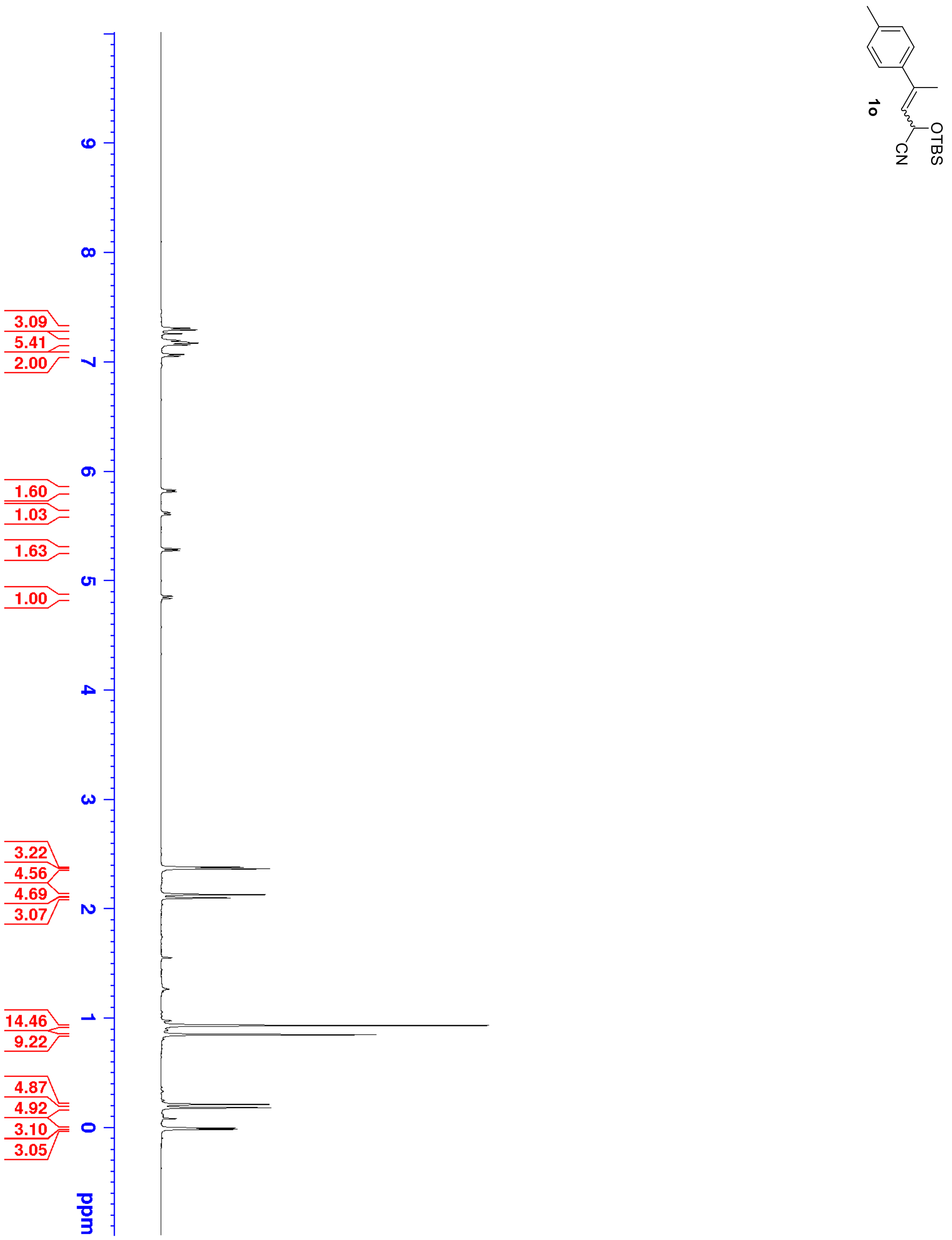

OTBS
CN
1o
3.09
5.41
2.00
1.60
1.03
1.63
1.00
3.22
4.56
4.69
3.07
14.46
9.22
4.87
4.92
3.10
3.05
9
8
7
6
5
4
3
2
1
0
ppm

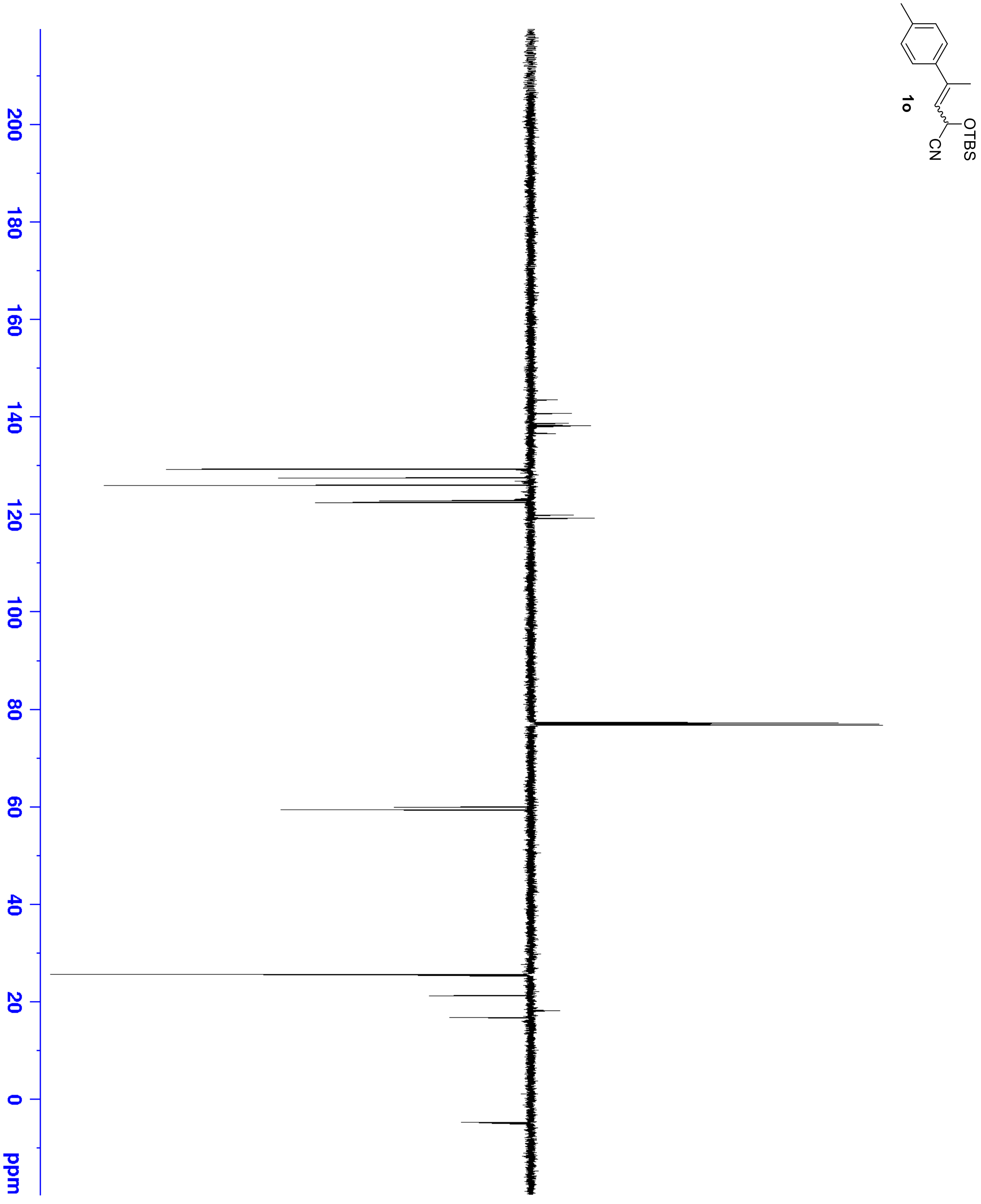
OTBS
CN
1o
200
180
160
140
120
100
80
60
40
20
0
ppm

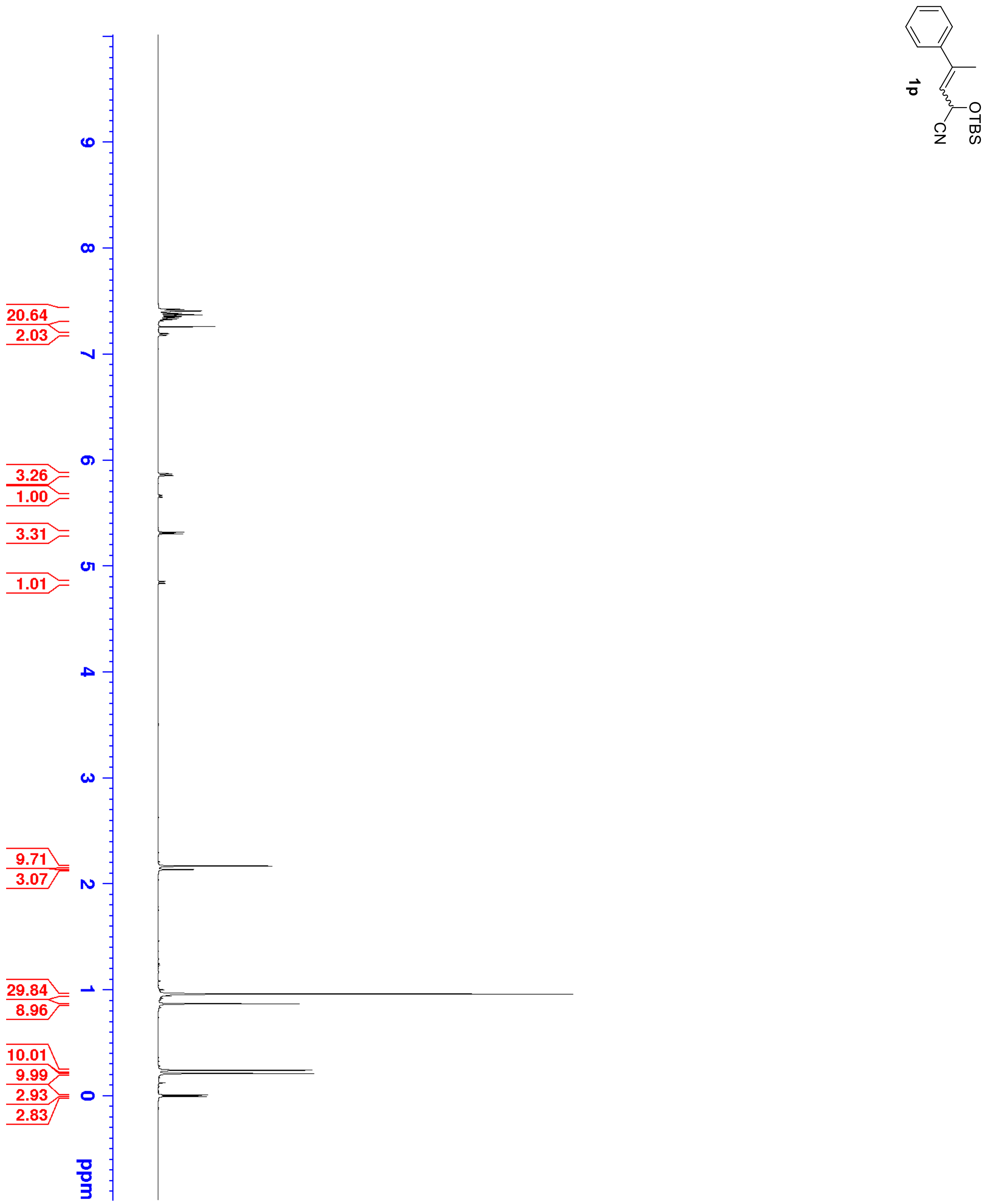
OTBS
CN
1p
20.64
2.03
3.26
1.00
3.31
1.01
9.71
3.07
29.84
8.96
10.01
9.99
2.93
2.83
9
8
7
6
5
4
3
2
1
0
ppm

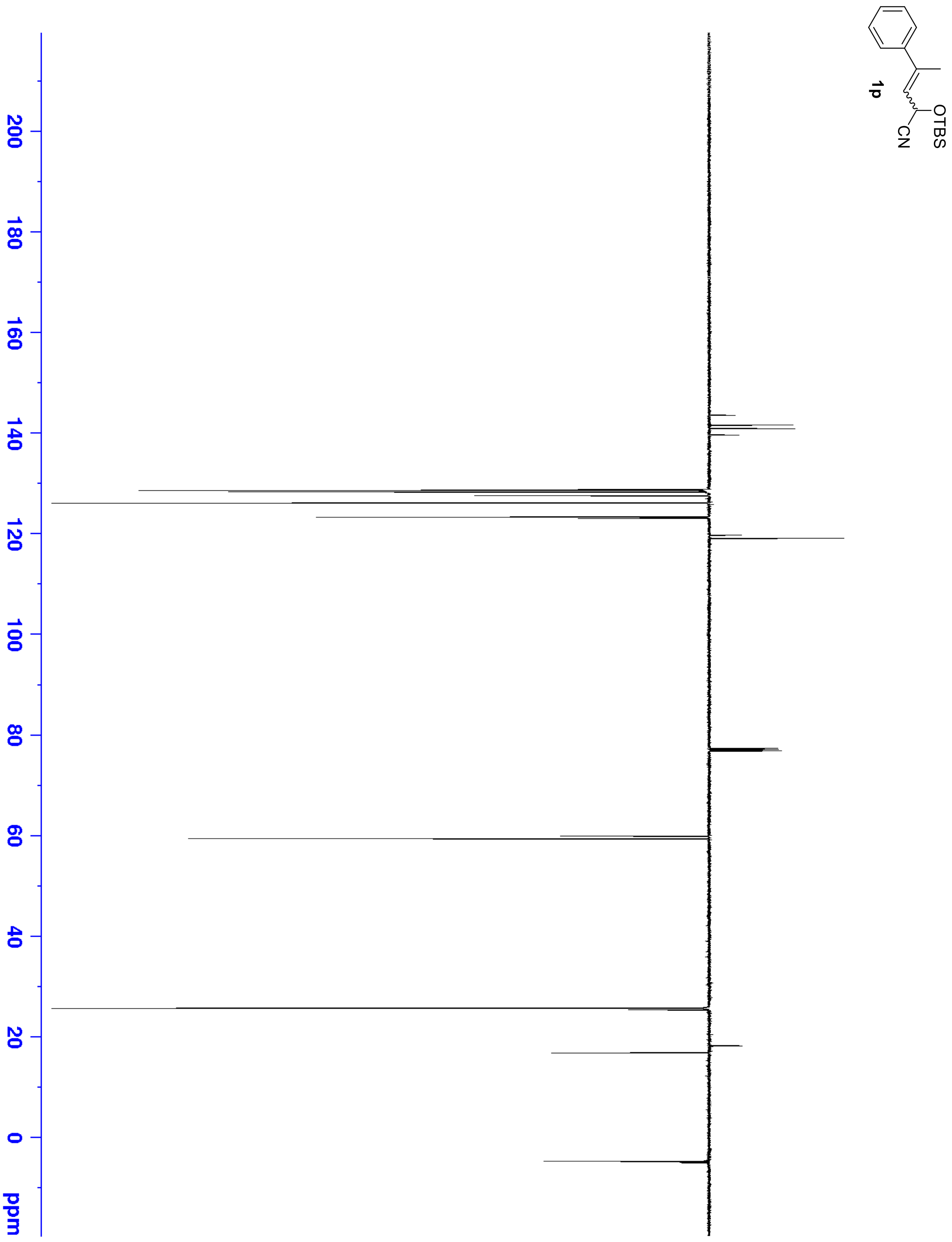

OTBS
CN
1p
200
180
160
140
120
100
80
60
40
20
0
ppm

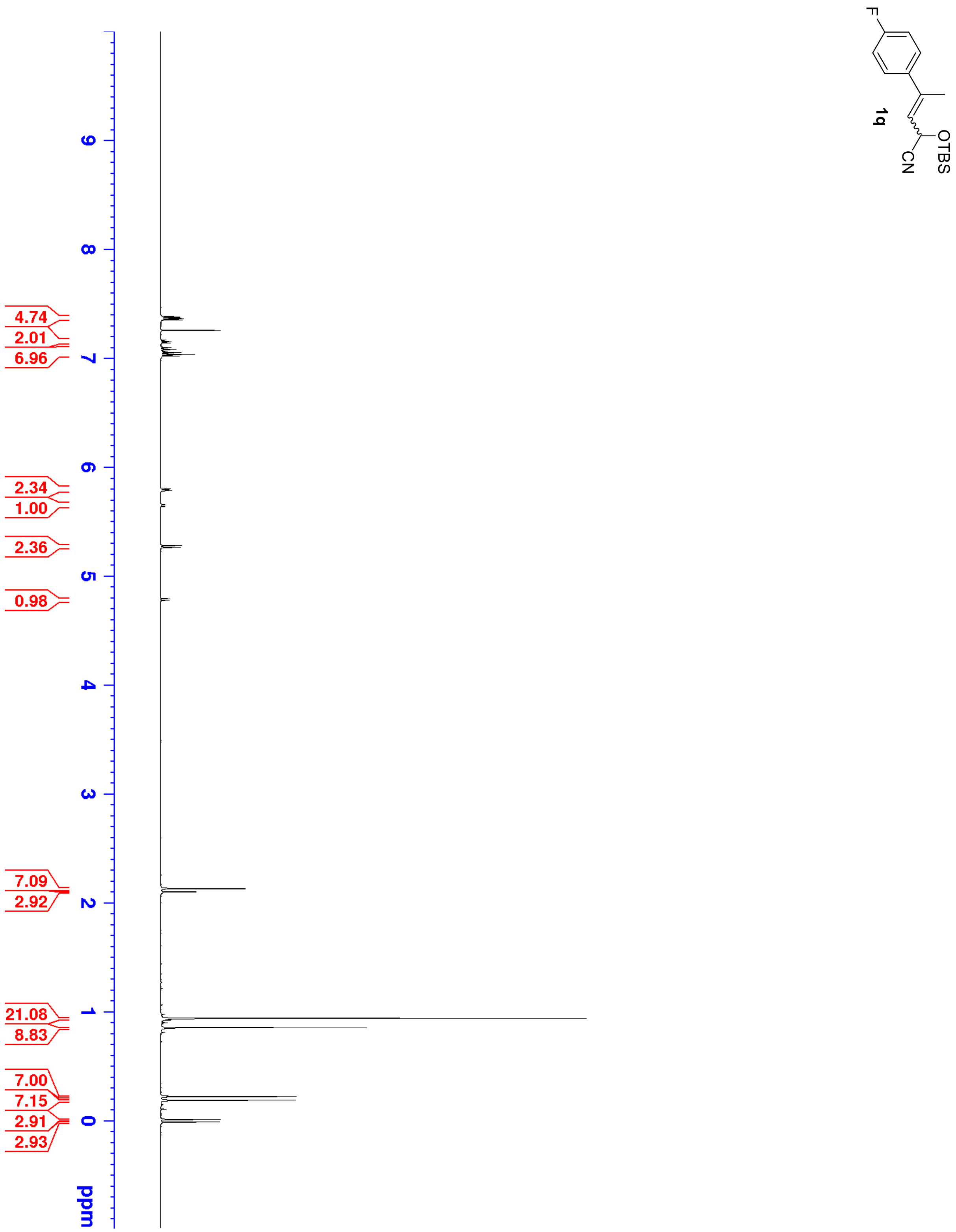
F
OTBS
CN
1q
4.74
2.01
6.96
2.34
1.00
2.36
0.98
7.09
2.92
21.08
8.83
7.00
7.15
2.91
2.93
9
8
7
6
5
4
3
2
1
0
ppm

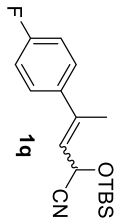

1q

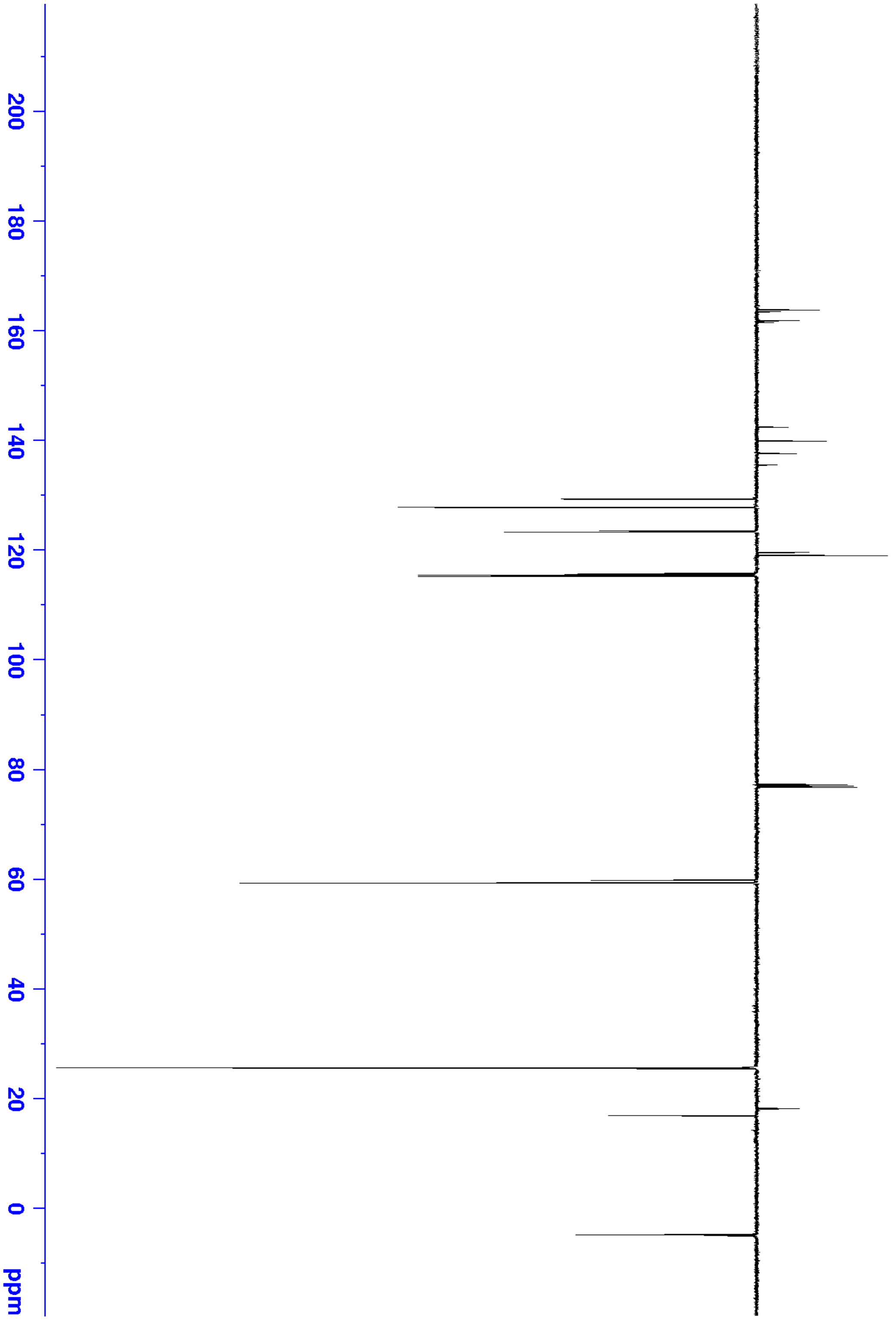

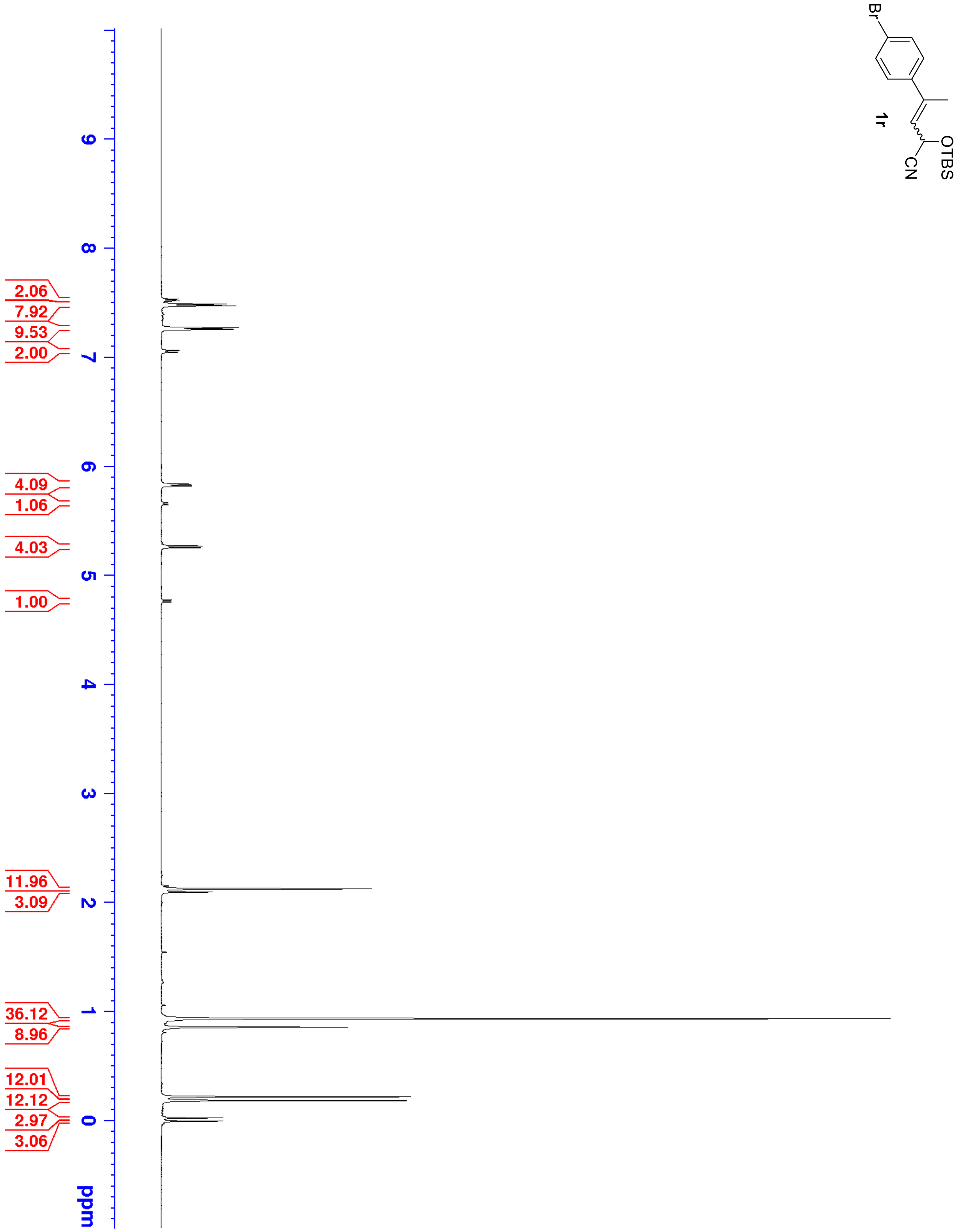

Br
OTBS
CN
1r
2.06
7.92
9.53
2.00
4.09
1.06
4.03
1.00
11.96
3.09
36.12
8.96
12.01
12.12
2.97
3.06
9
8
7
6
5
4
3
2
1
0
ppm

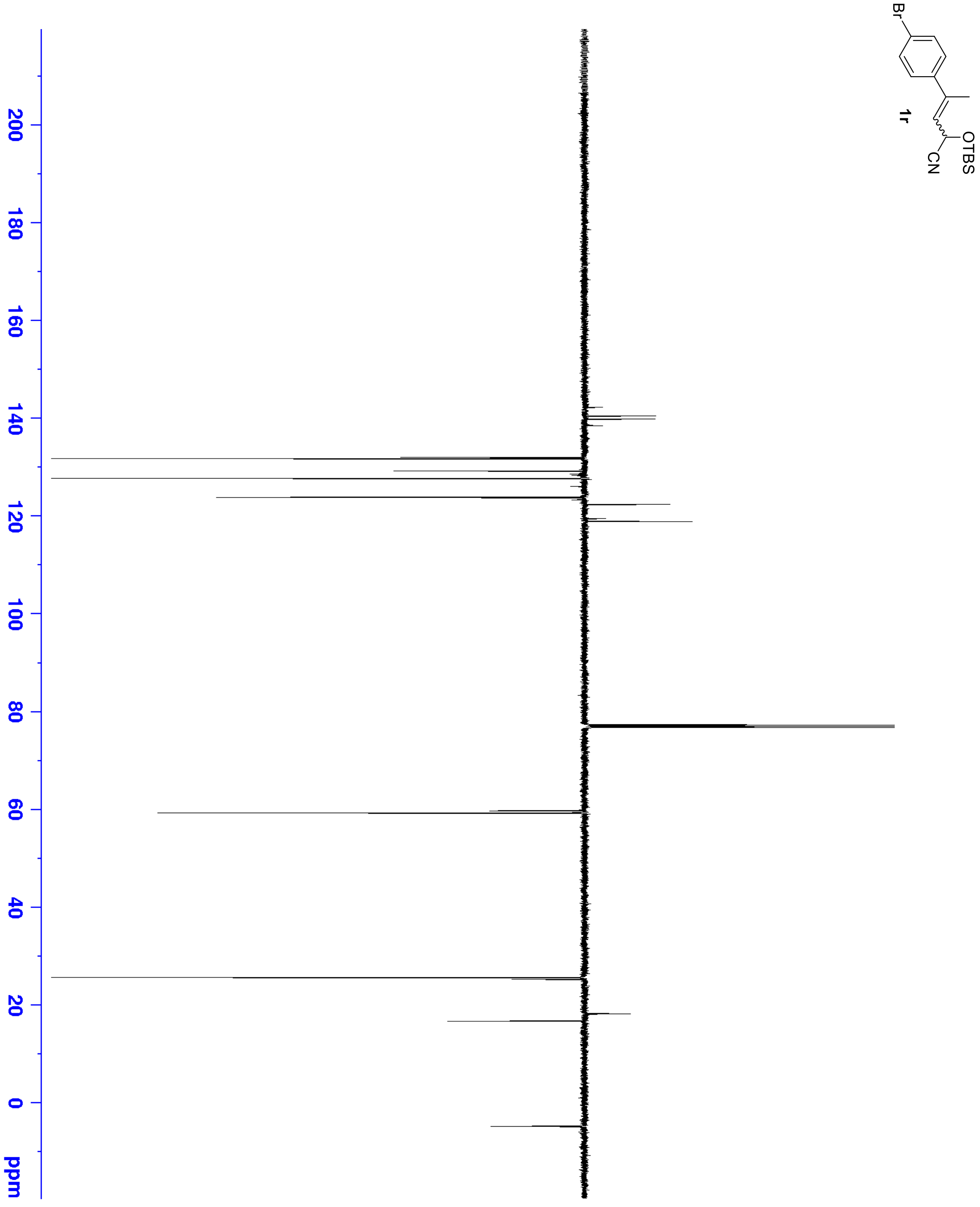

Br
OTBS
CN
1r
200
180
160
140
120
100
80
60
40
20
0
ppm

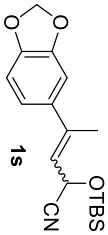
OTBS
CN

1s

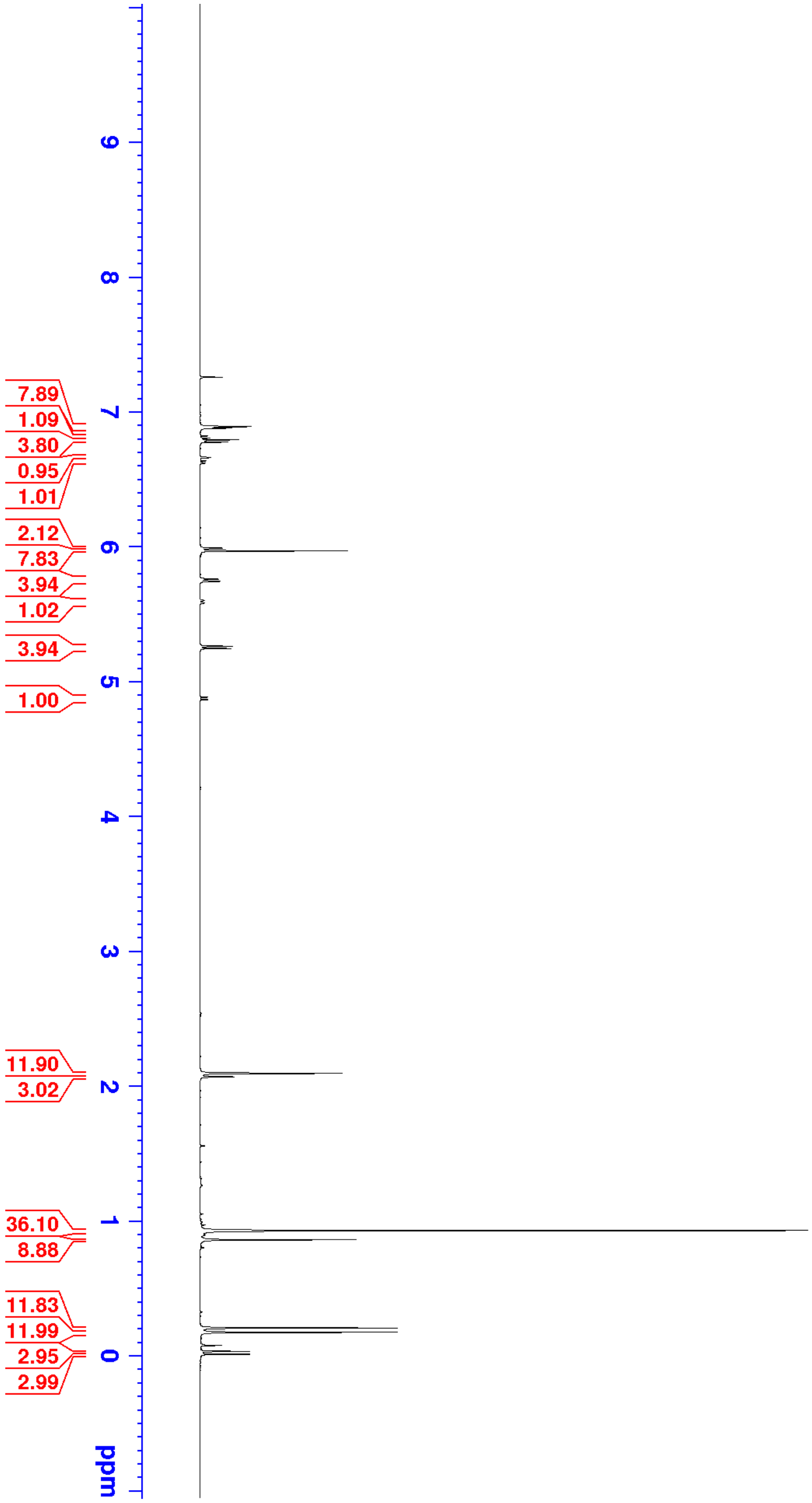
9
8
7
6
5
4
3
2
1
0
ppm
7.89
1.09
3.80
0.95
1.01
2.12
7.83
3.94
1.02
3.94
1.00
11.90
3.02
36.10
8.88
11.83
11.99
2.95
2.99

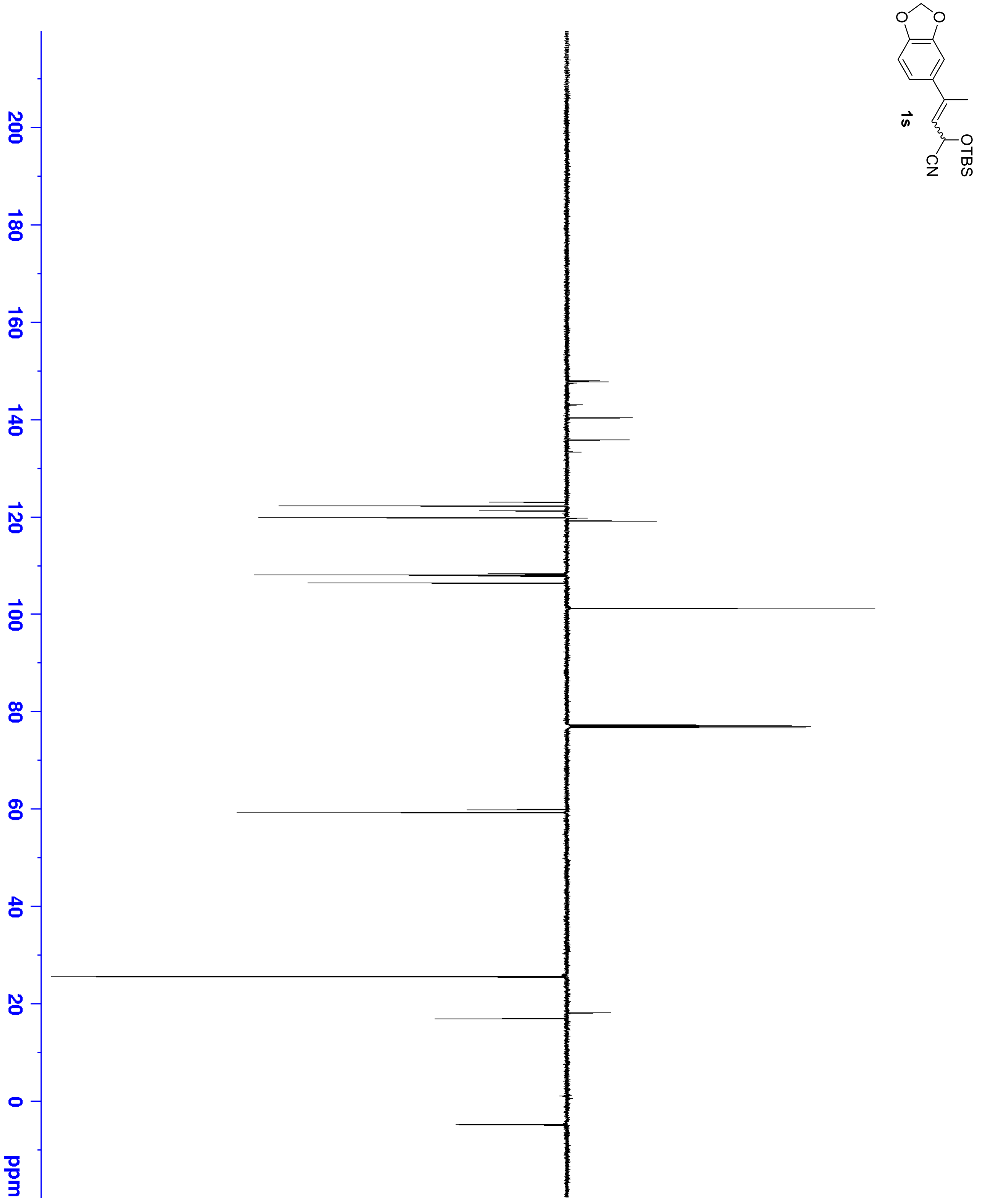
OTBS
CN
1s
200
180
160
140
120
100
80
60
40
20
0
ppm

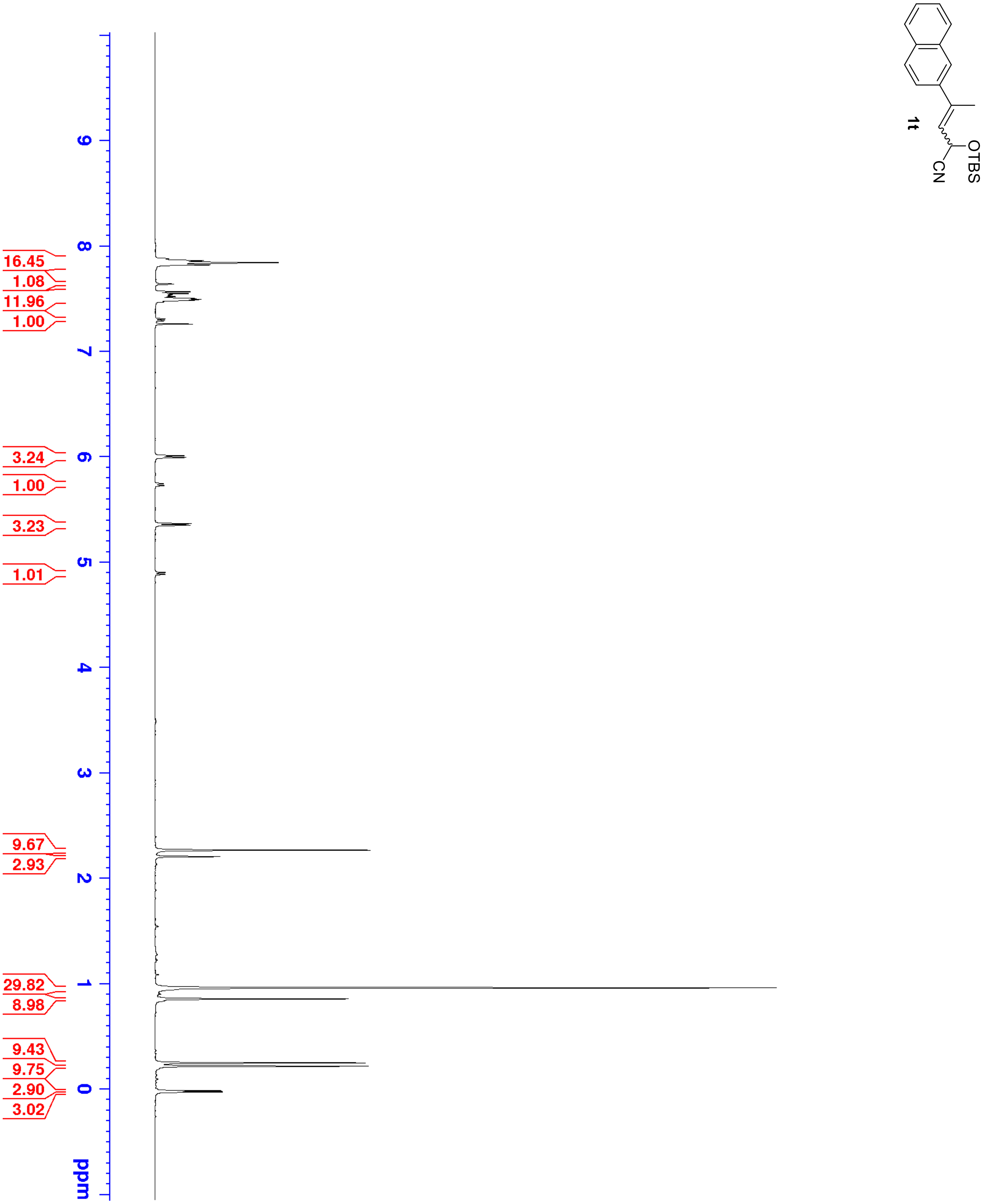
OTBS
CN
1t
ppm
16.45
1.08
11.96
1.00
3.24
1.00
3.23
1.01
9.67
2.93
29.82
8.98
9.43
9.75
2.90
3.02

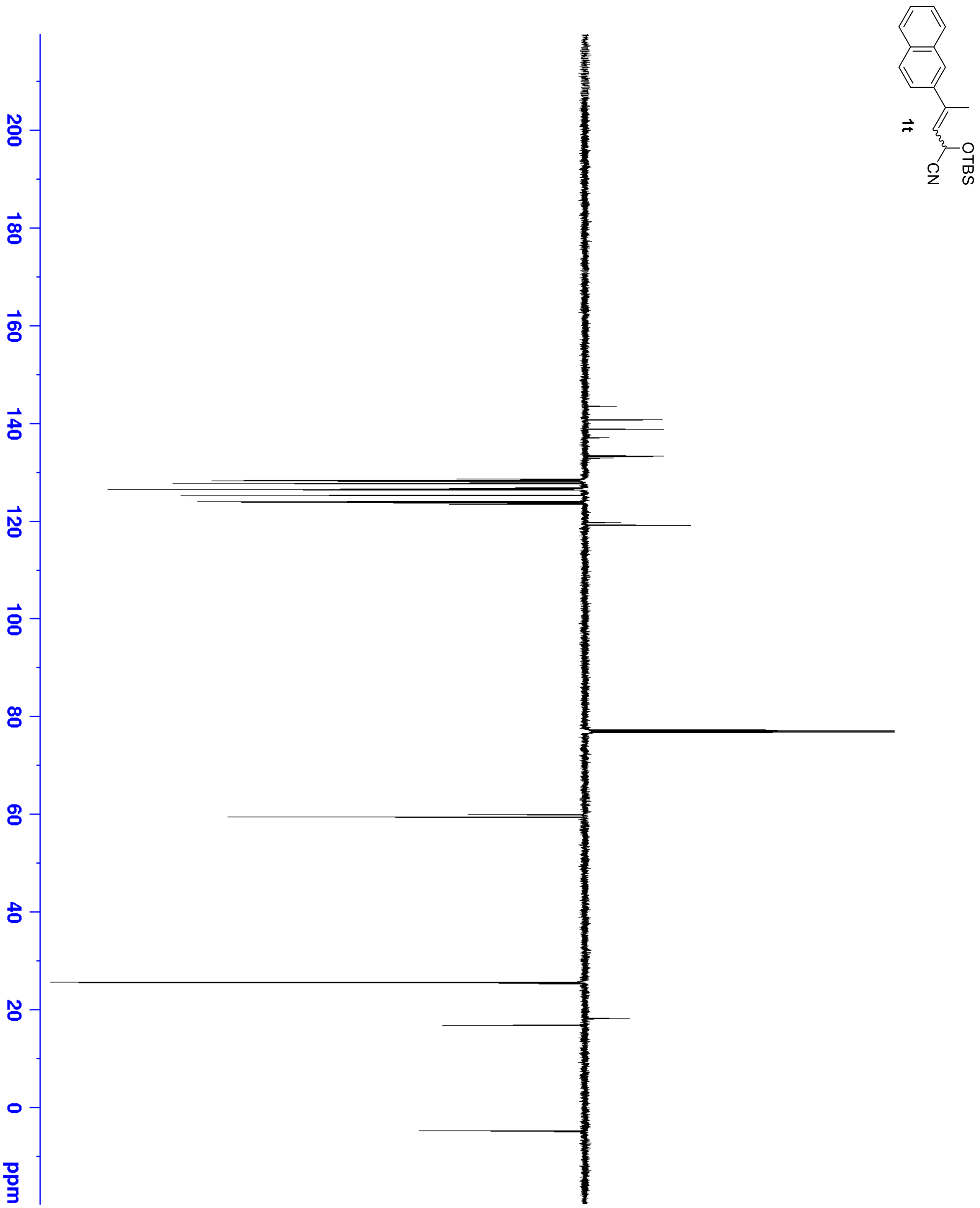

OTBS
CN
1t
200
180
160
140
120
100
80
60
40
20
0
ppm

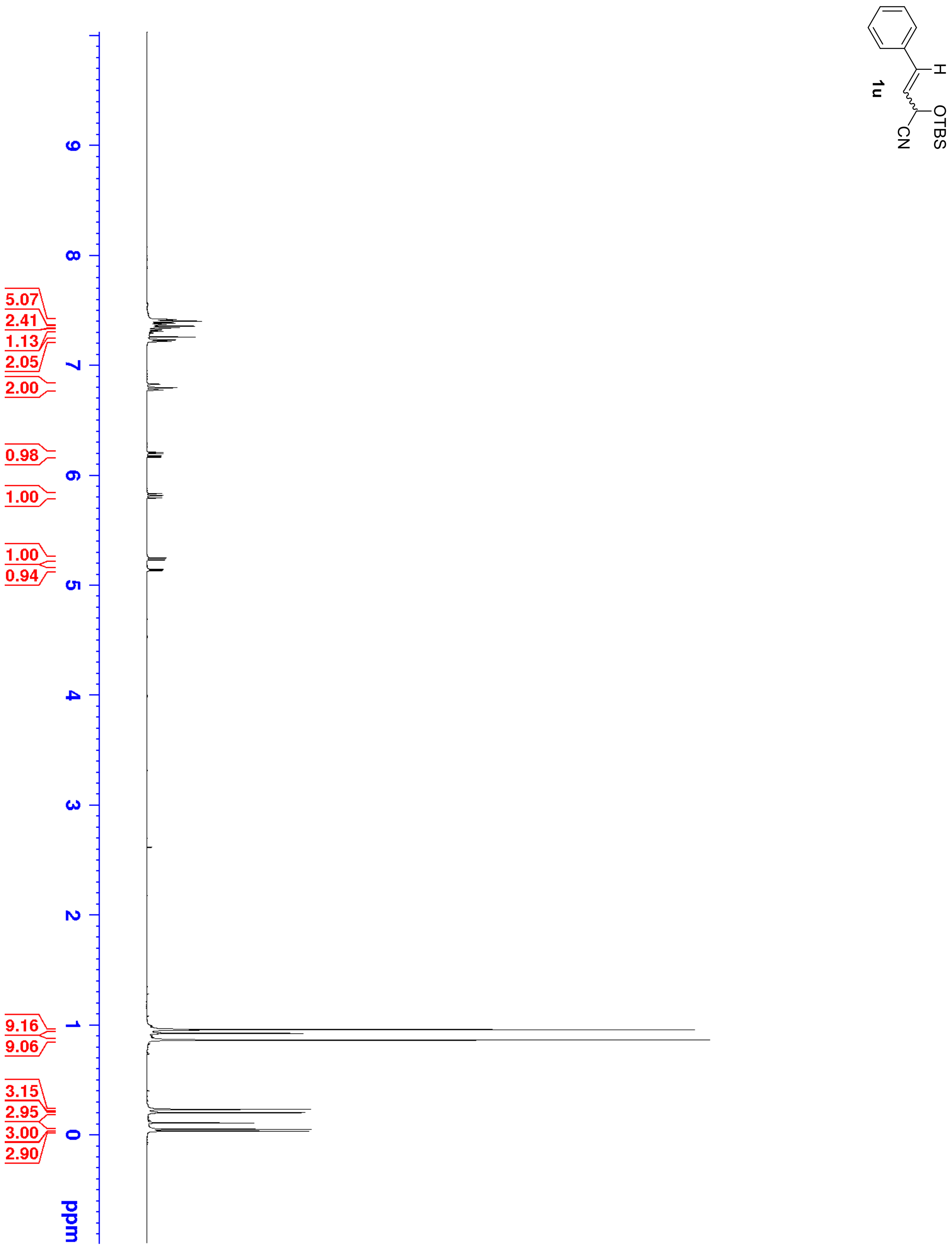
H
OTBS
CN
1u
ppm
5.07
2.41
1.13
2.05
2.00
0.98
1.00
1.00
0.94
9.16
9.06
3.15
2.95
3.00
2.90

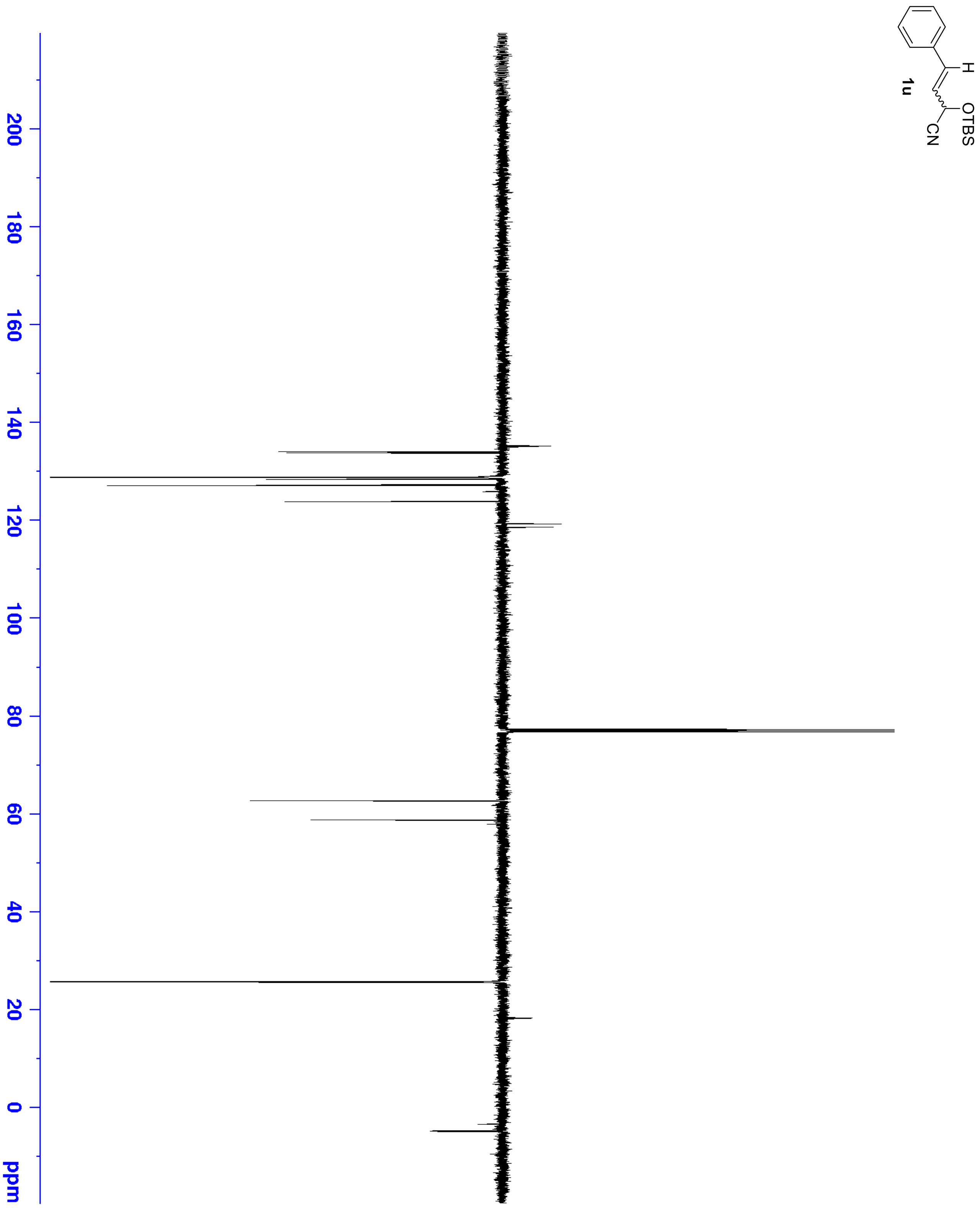
H
OTBS
CN
1u
200
180
160
140
120
100
80
60
40
20
0
ppm

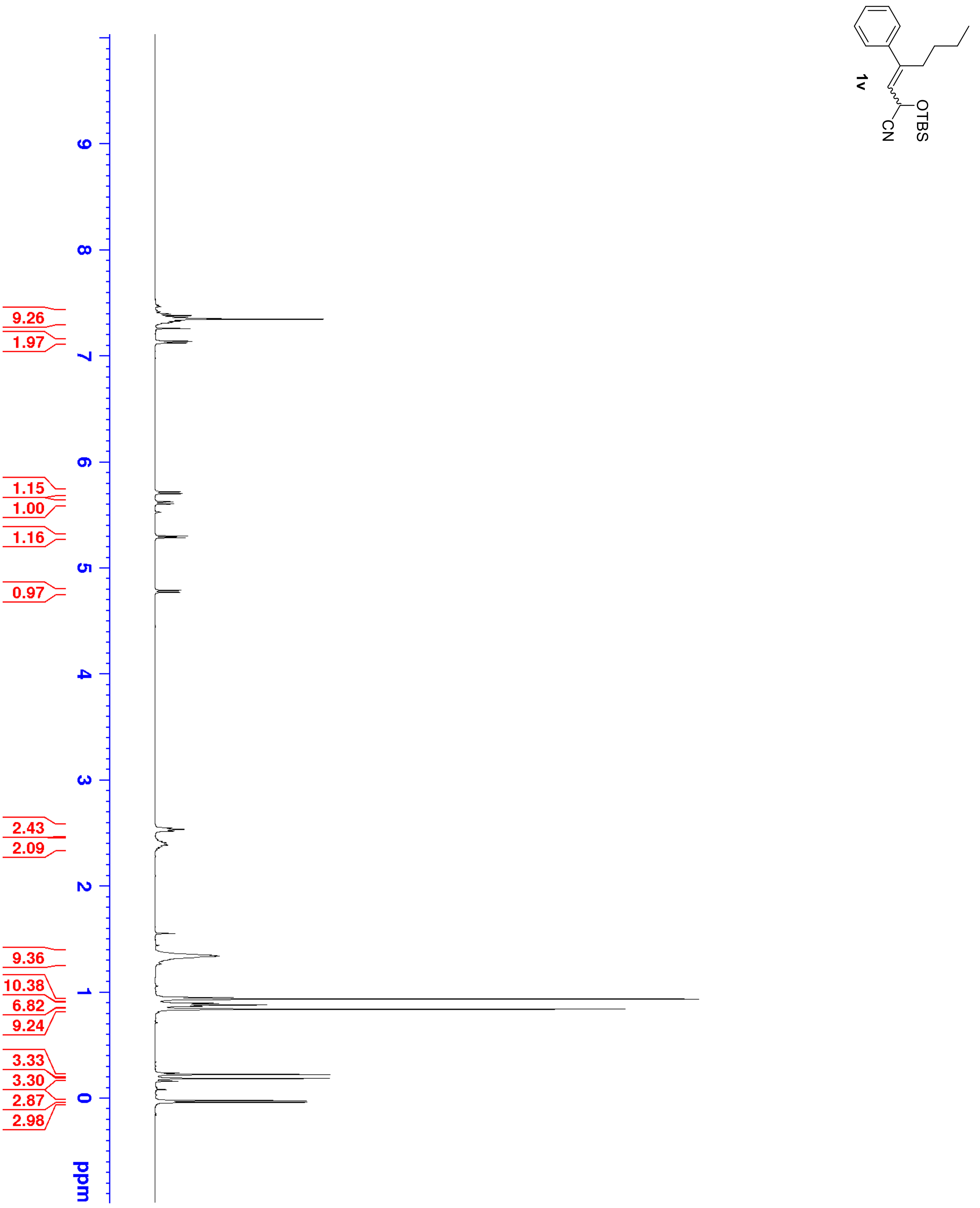
OTBS
CN
1v
9.26
1.97
1.15
1.00
1.16
0.97
2.43
2.09
9.36
10.38
6.82
9.24
3.33
3.30
2.87
2.98
ppm

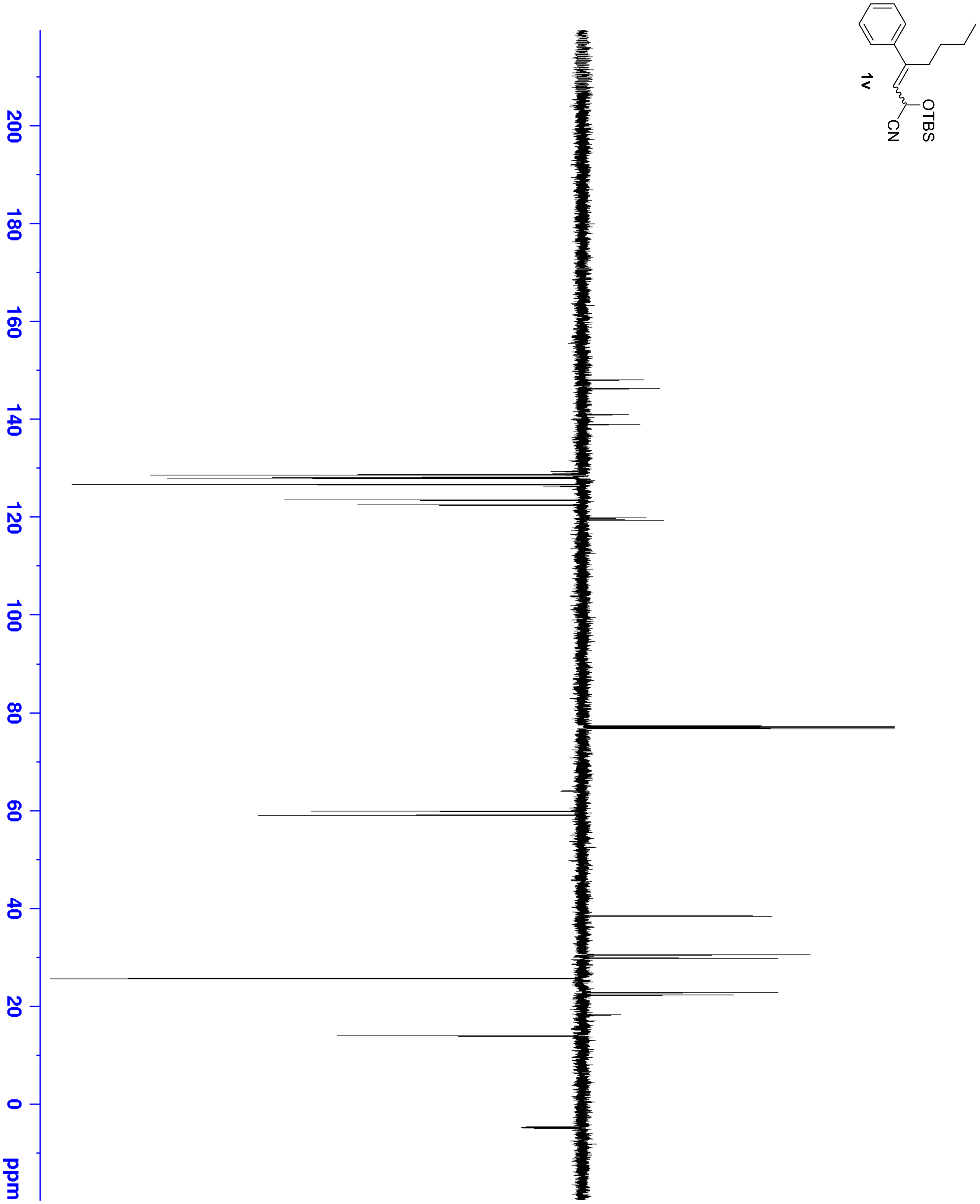

OTBS
CN
1v
200
180
160
140
120
100
80
60
40
20
0
ppm

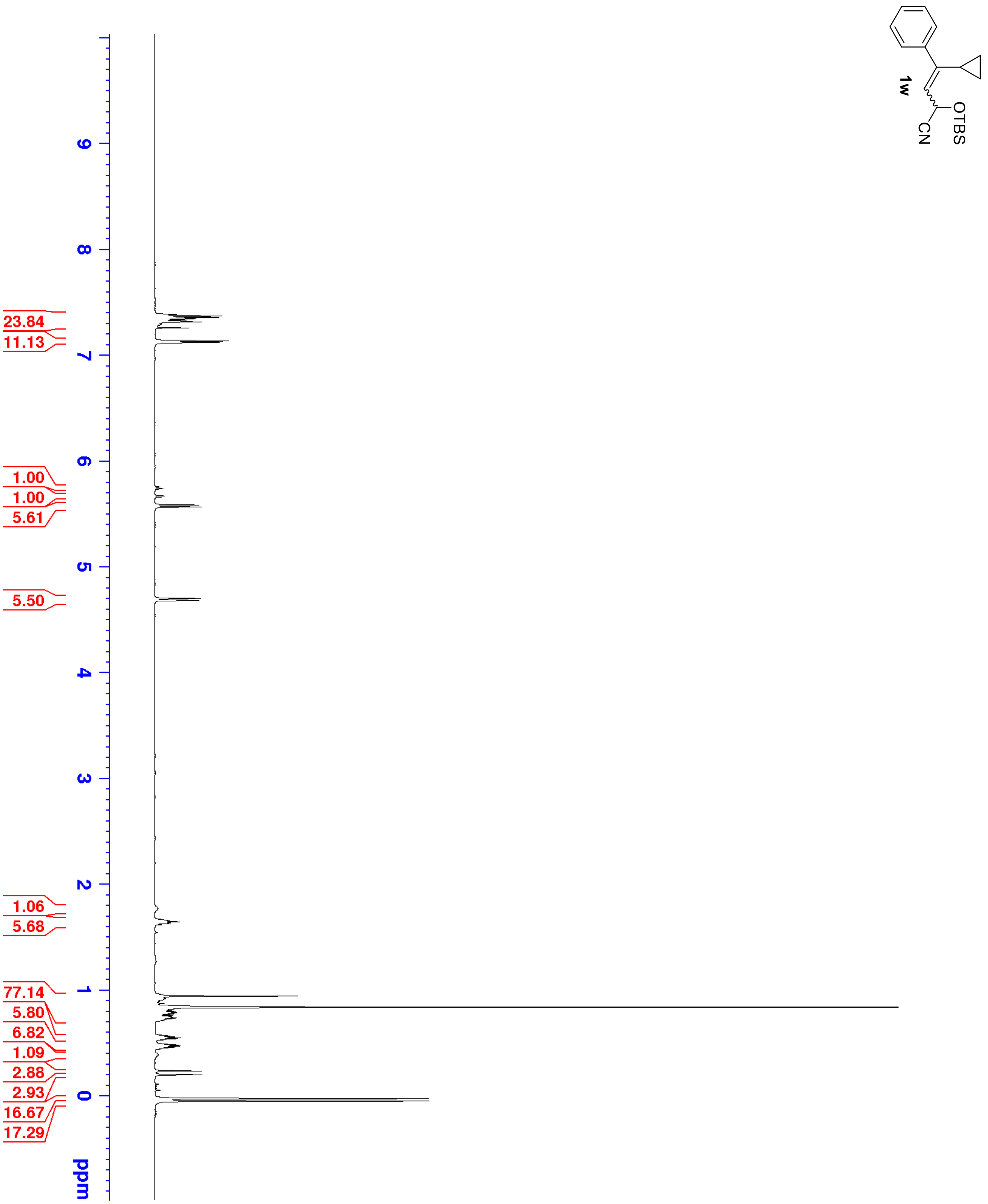
OTBS
CN
1w
23.84
11.13
1.00
1.00
5.61
5.50
1.06
5.68
77.14
5.80
6.82
1.09
2.88
2.93
16.67
17.29
9
8
7
6
5
4
3
2
1
0
ppm

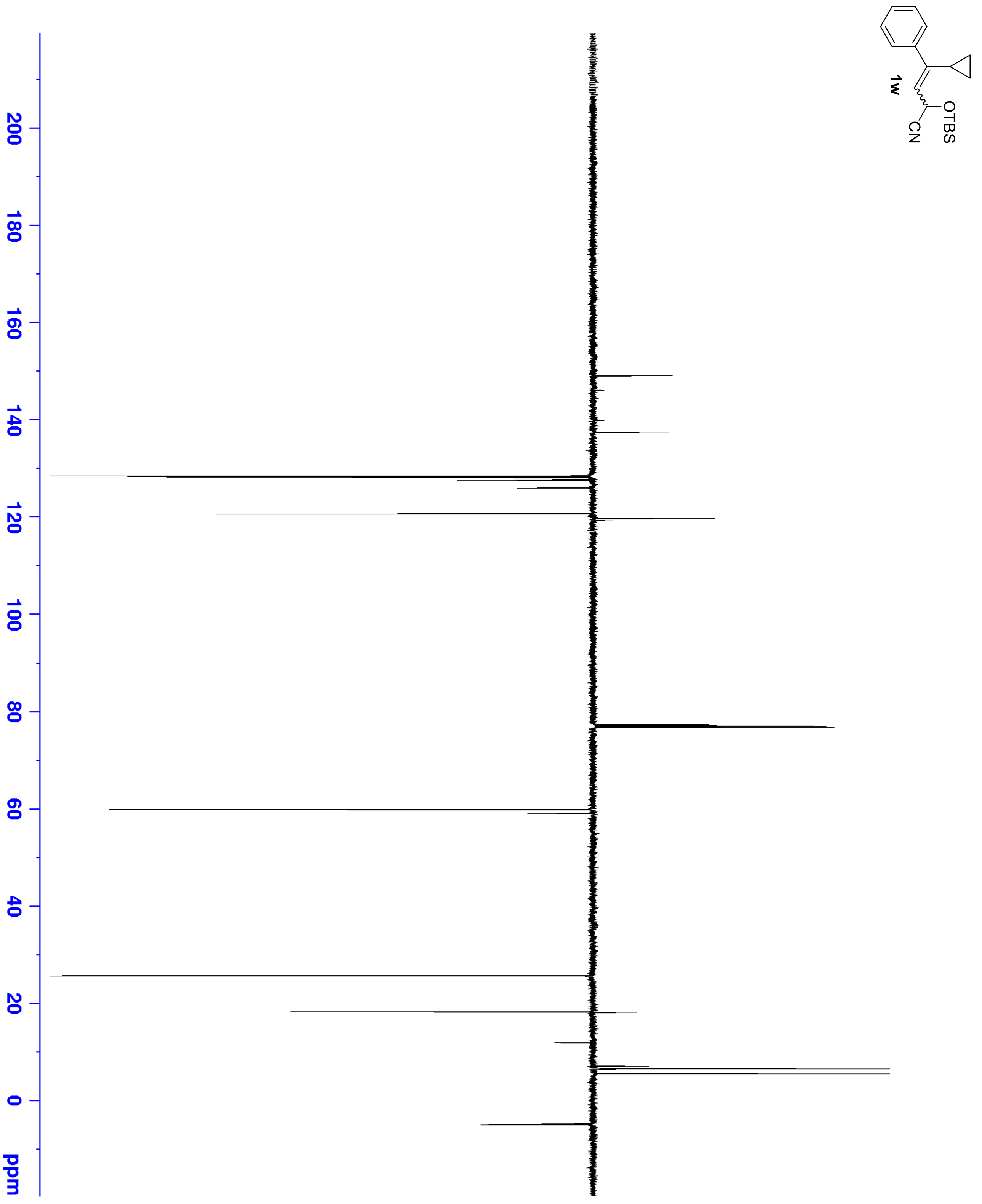
OTBS
CN
1w
200
180
160
140
120
100
80
60
40
20
0
ppm

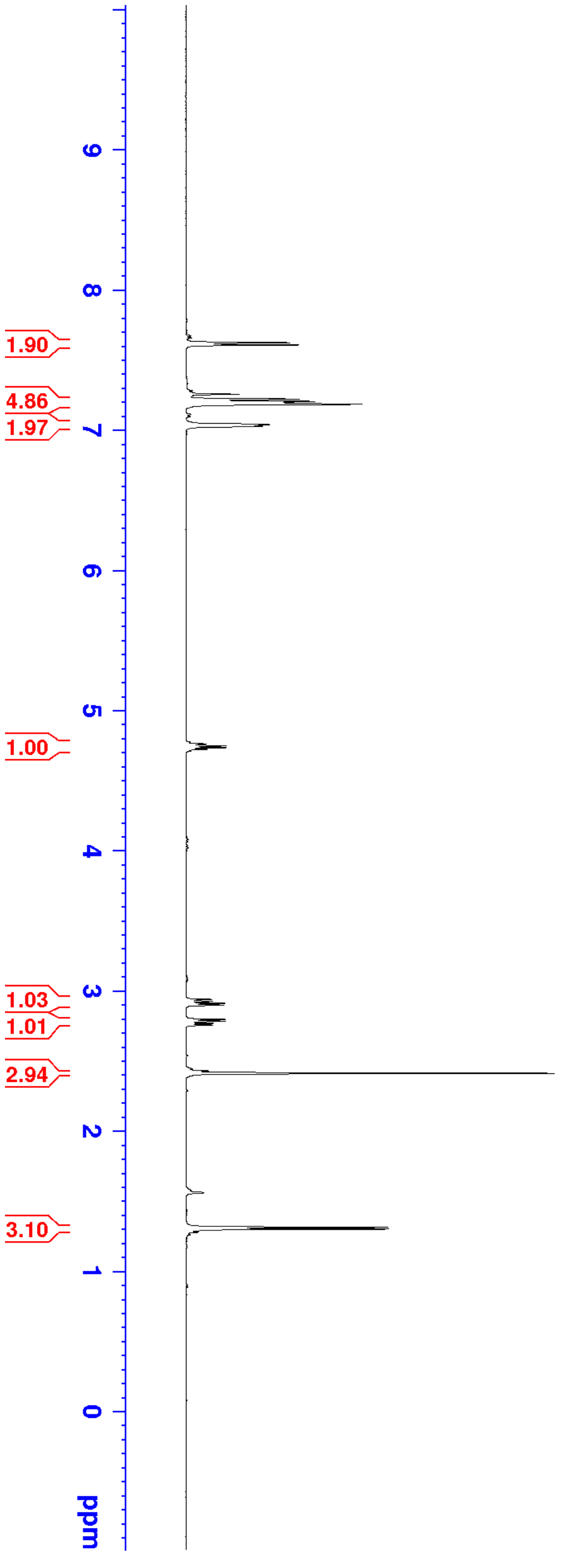

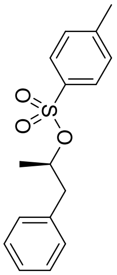

2a

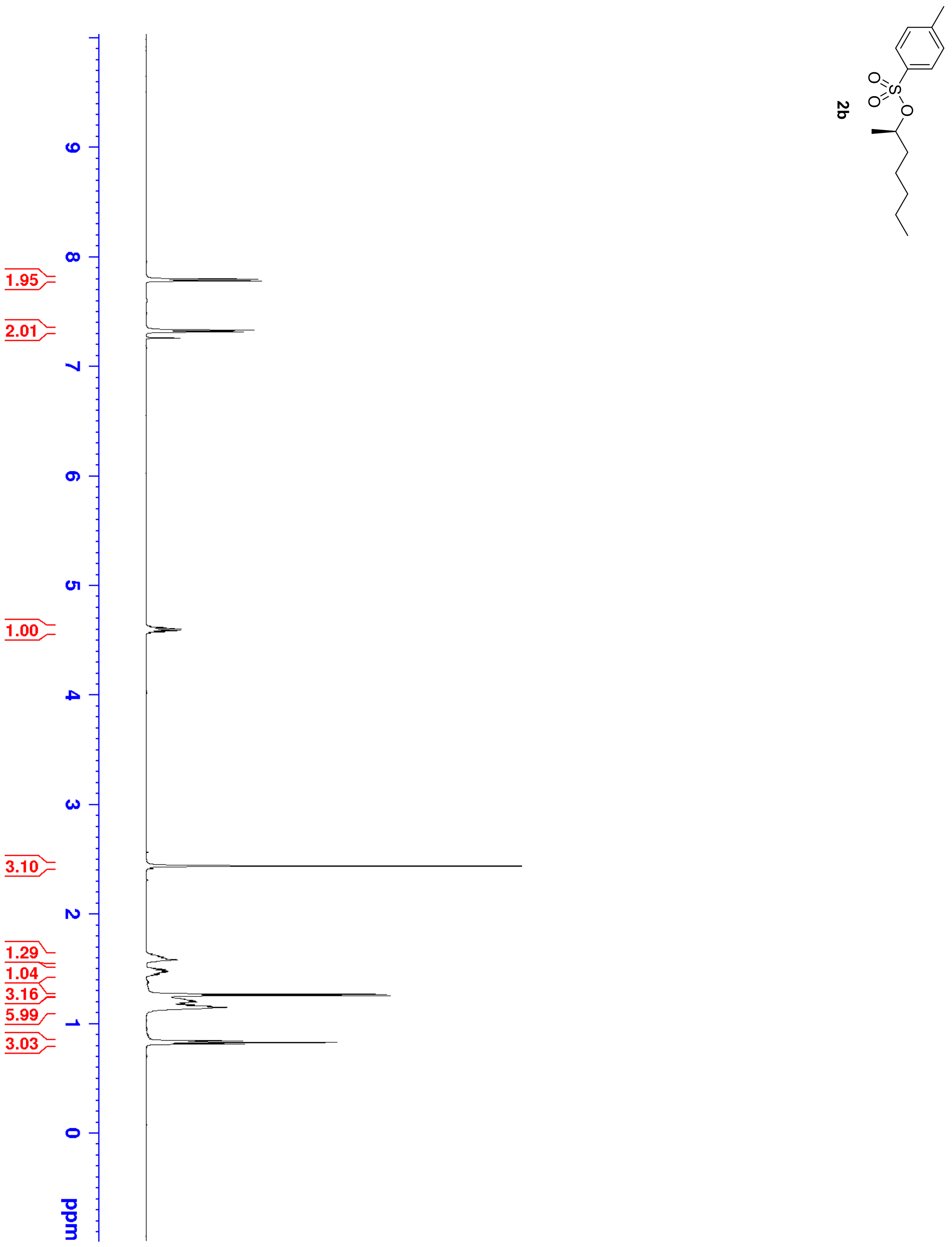
2b
9
8
7
6
5
4
3
2
1
0
ppm
1.95
2.01
1.00
3.10
1.29
1.04
3.16
5.99
3.03

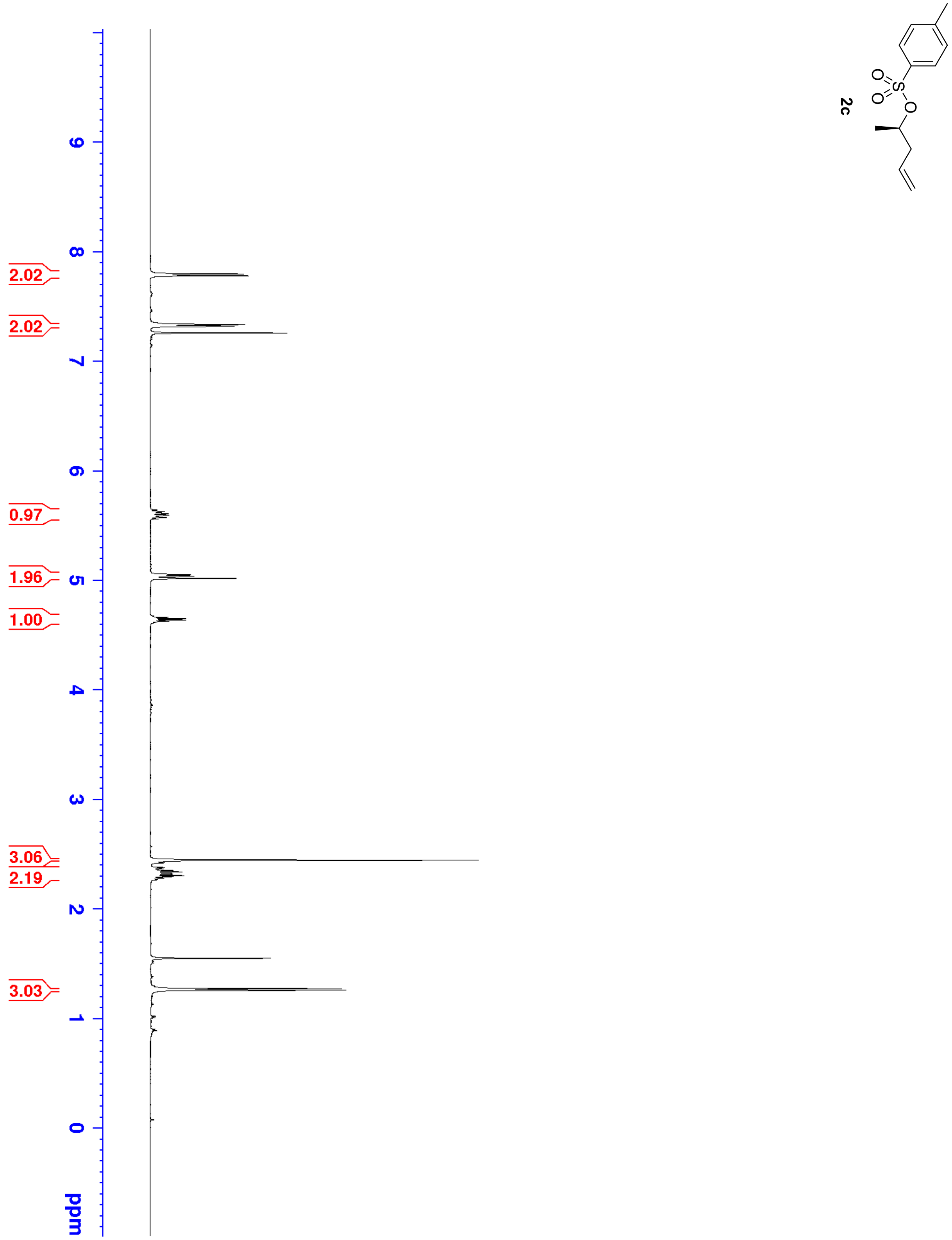
2c
9
8
7
6
5
4
3
2
1
0
ppm
2.02
2.02
0.97
1.96
1.00
3.06
2.19
3.03

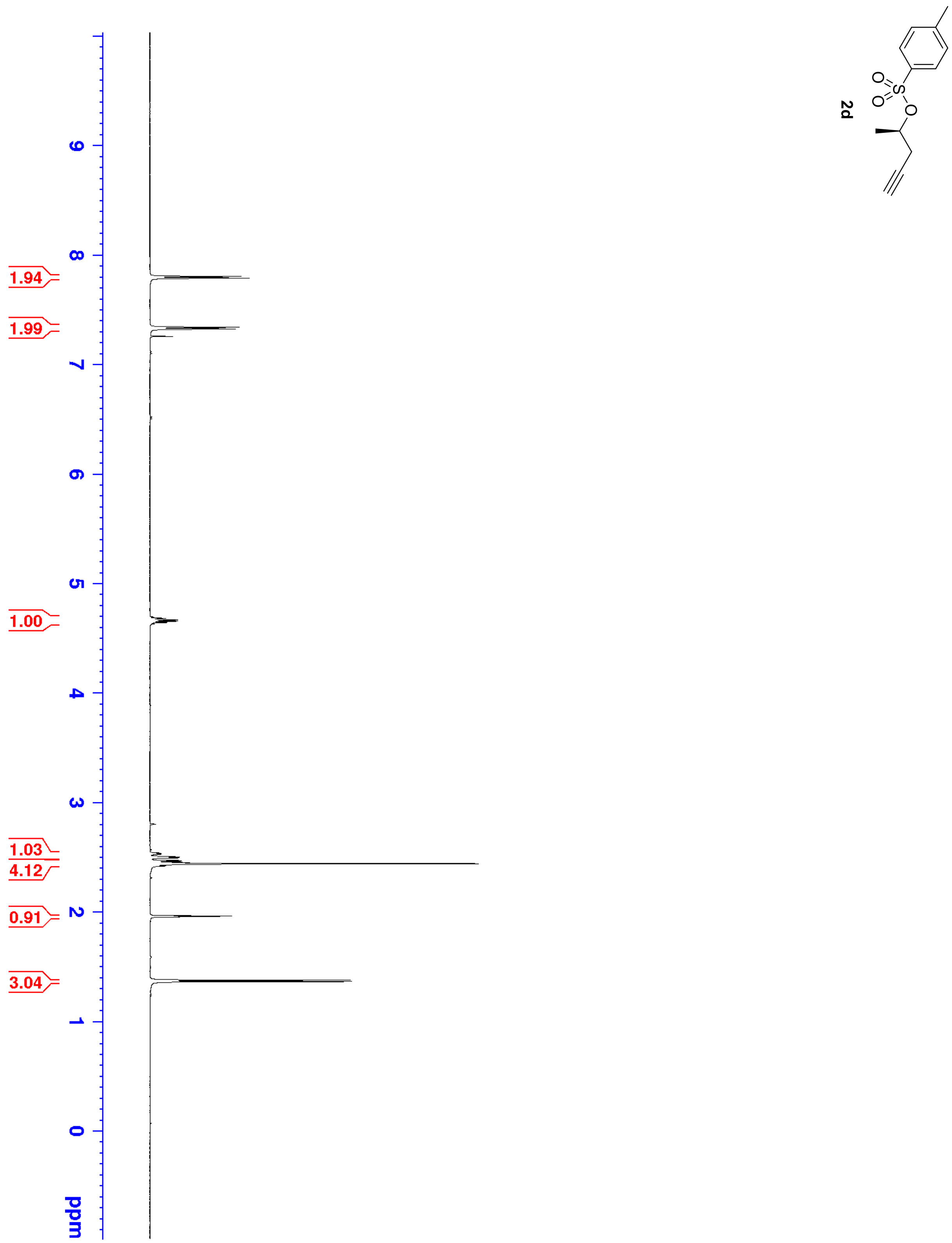
2d
1.94
1.99
1.00
1.03
4.12
0.91
3.04
9
8
7
6
5
4
3
2
1
0
ppm

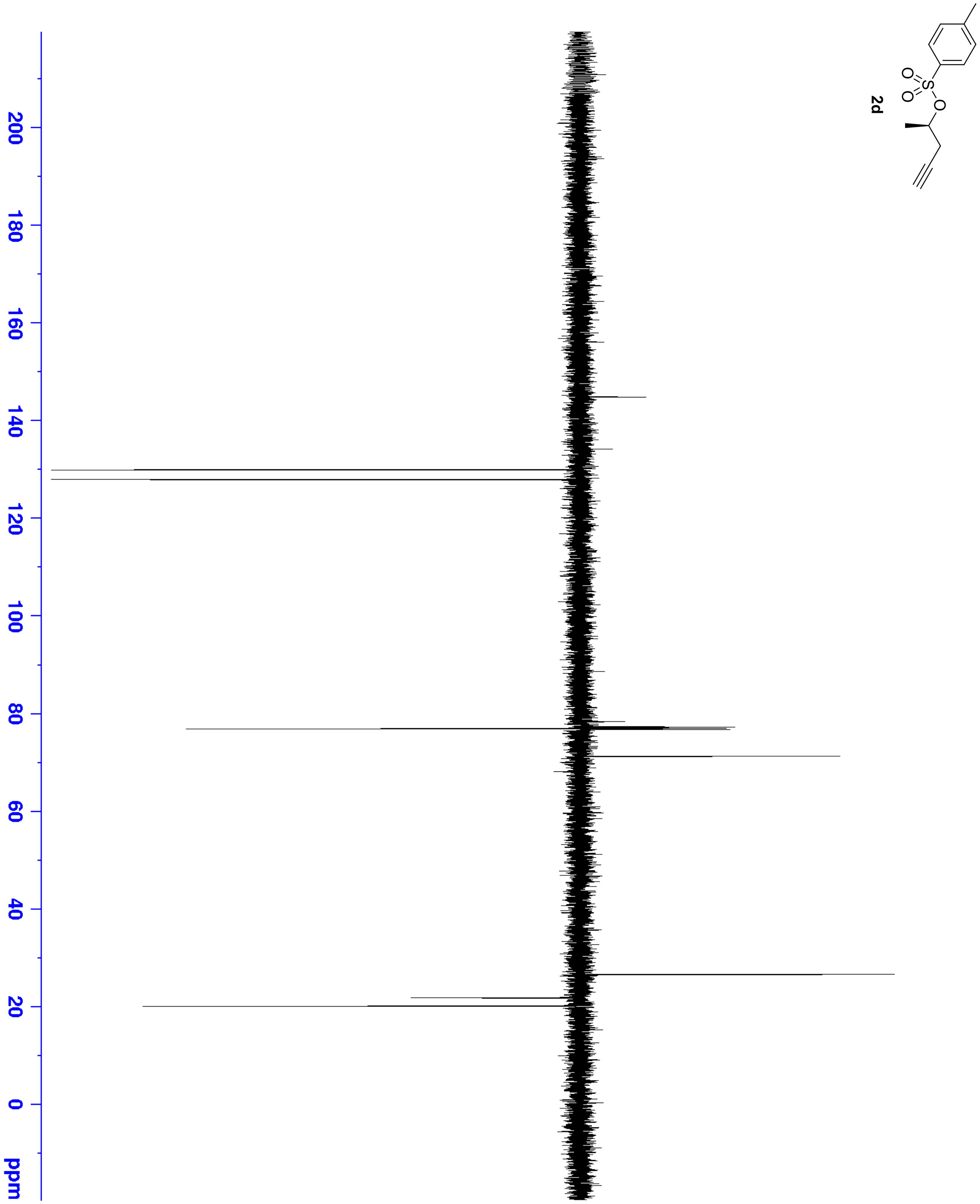
200
180
160
140
120
100
80
60
40
20
0
ppm
2d

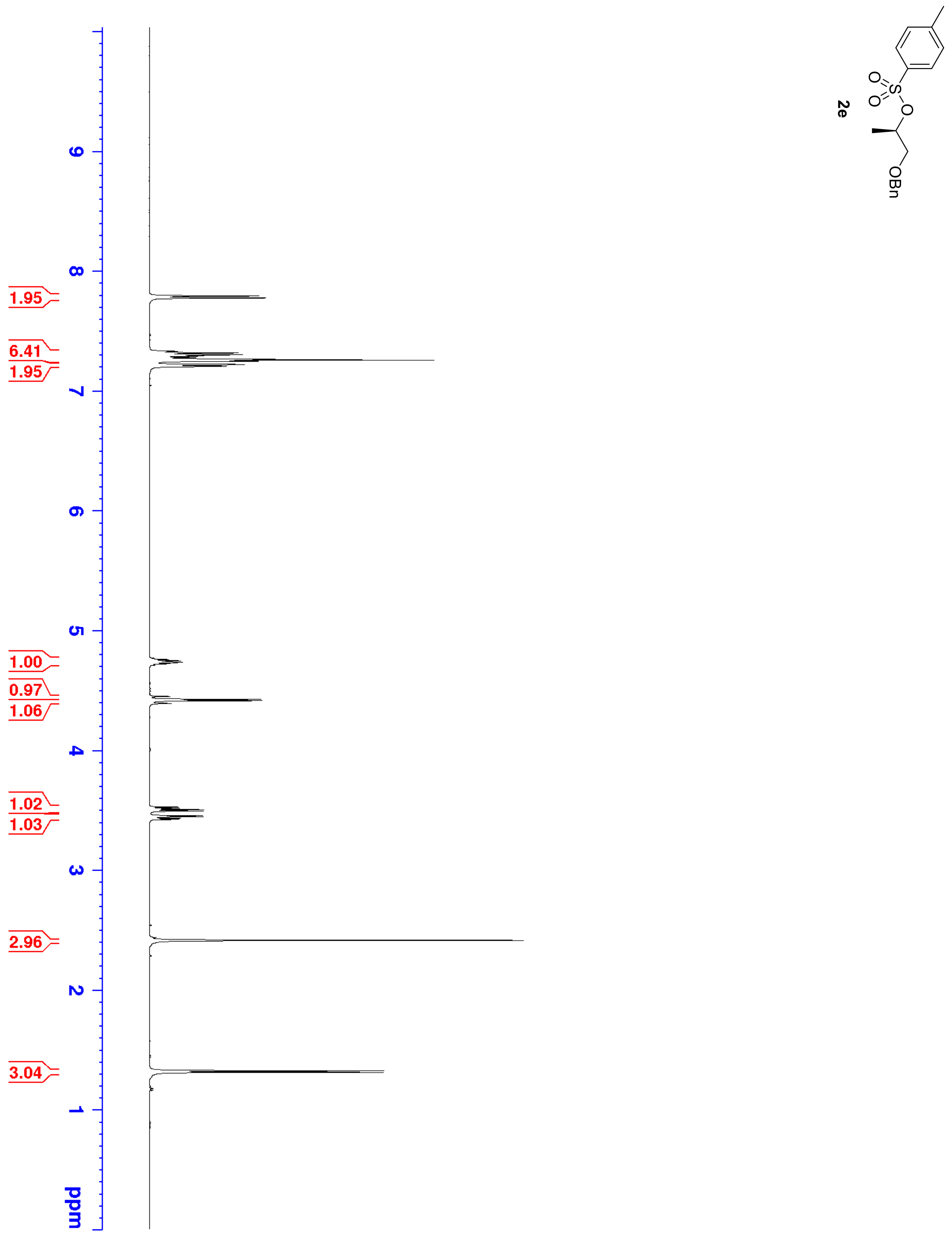

2e
OBn
1.95
6.41
1.95
1.00
0.97
1.06
1.02
1.03
2.96
3.04
ppm

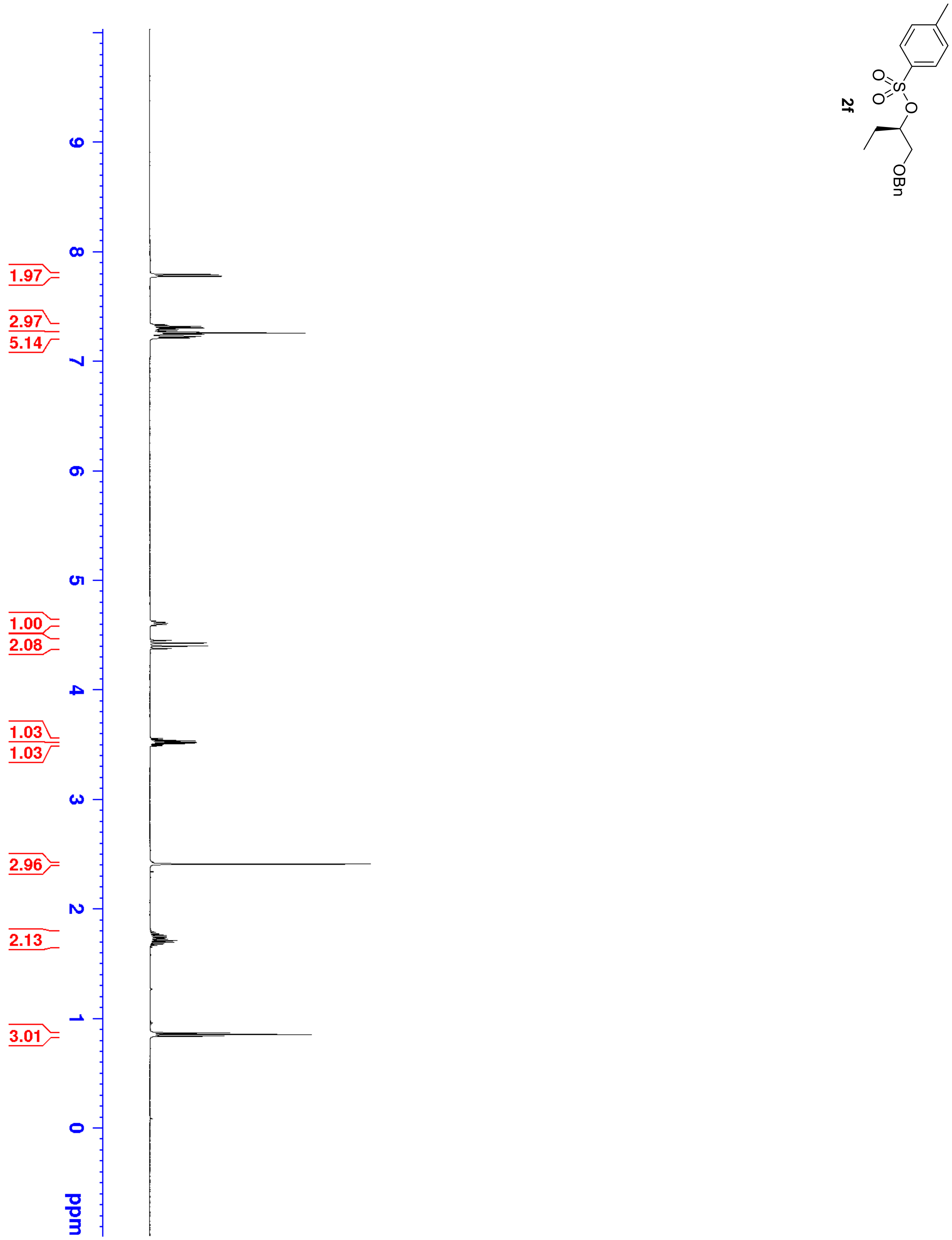
2f
OBn
ppm
1.97
2.97
5.14
1.00
2.08
1.03
1.03
2.96
2.13
3.01

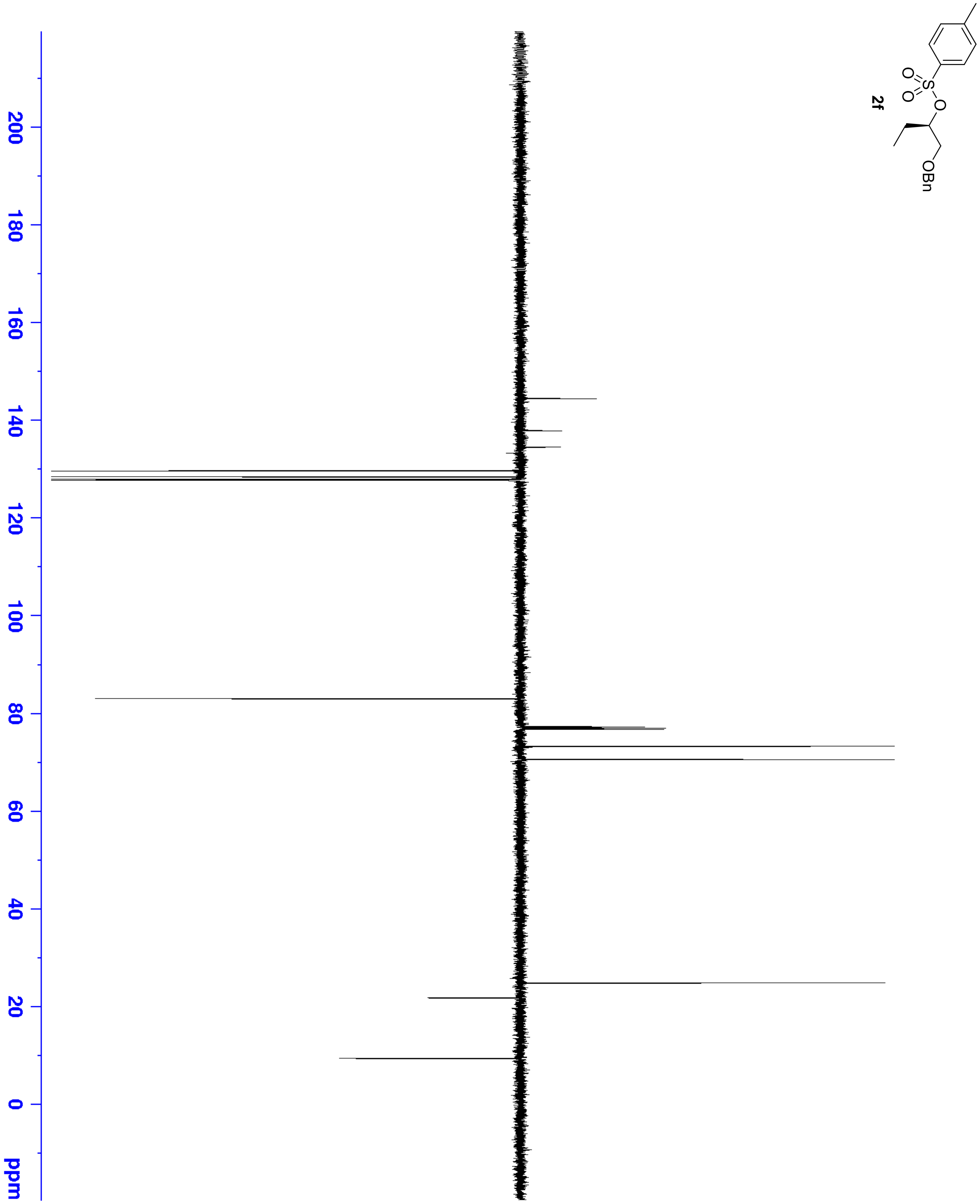

200
180
160
140
120
100
80
60
40
20
0
ppm
OBn
2f

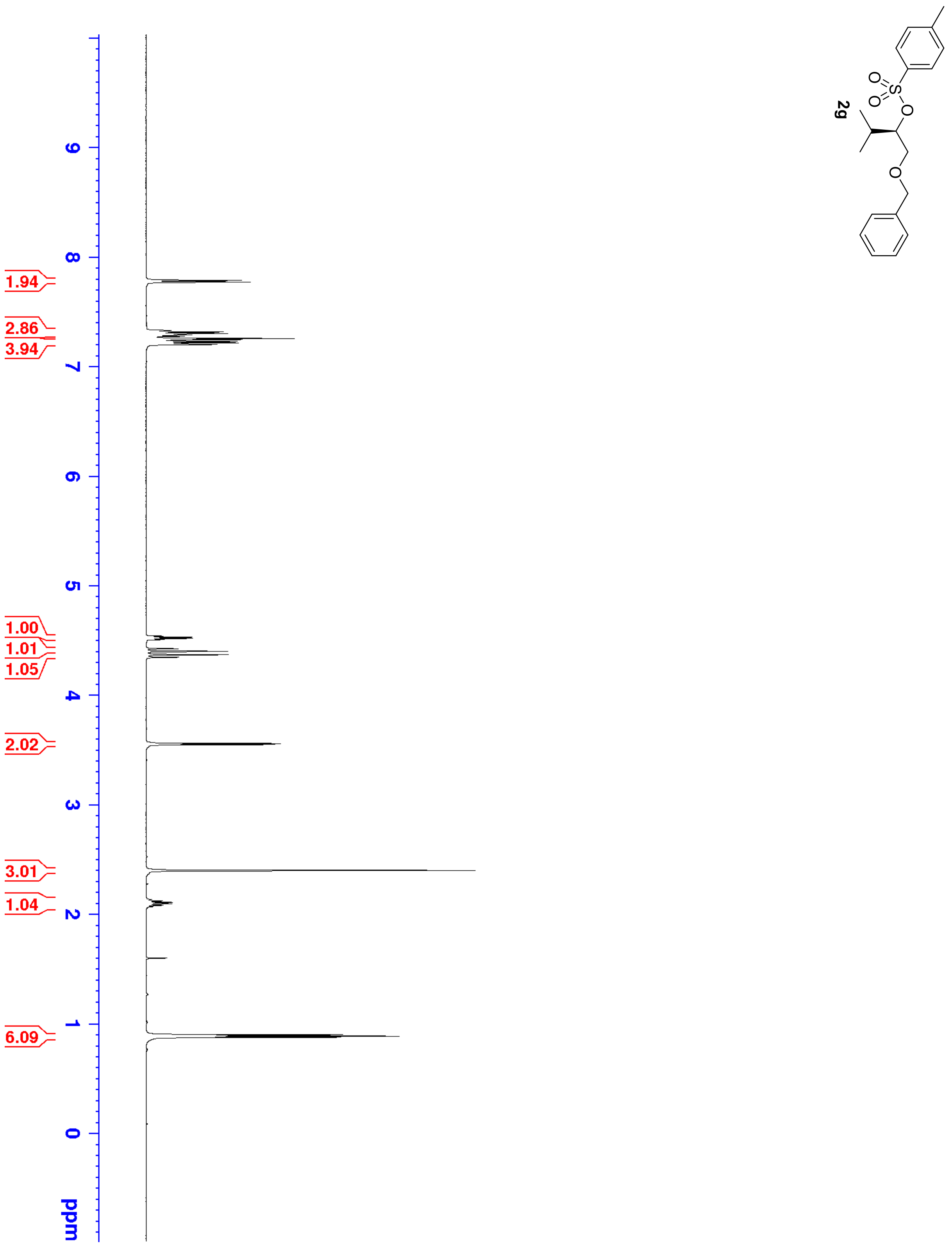
2g
1.94
2.86
3.94
1.00
1.01
1.05
2.02
3.01
1.04
6.09
9
8
7
6
5
4
3
2
1
0
ppm

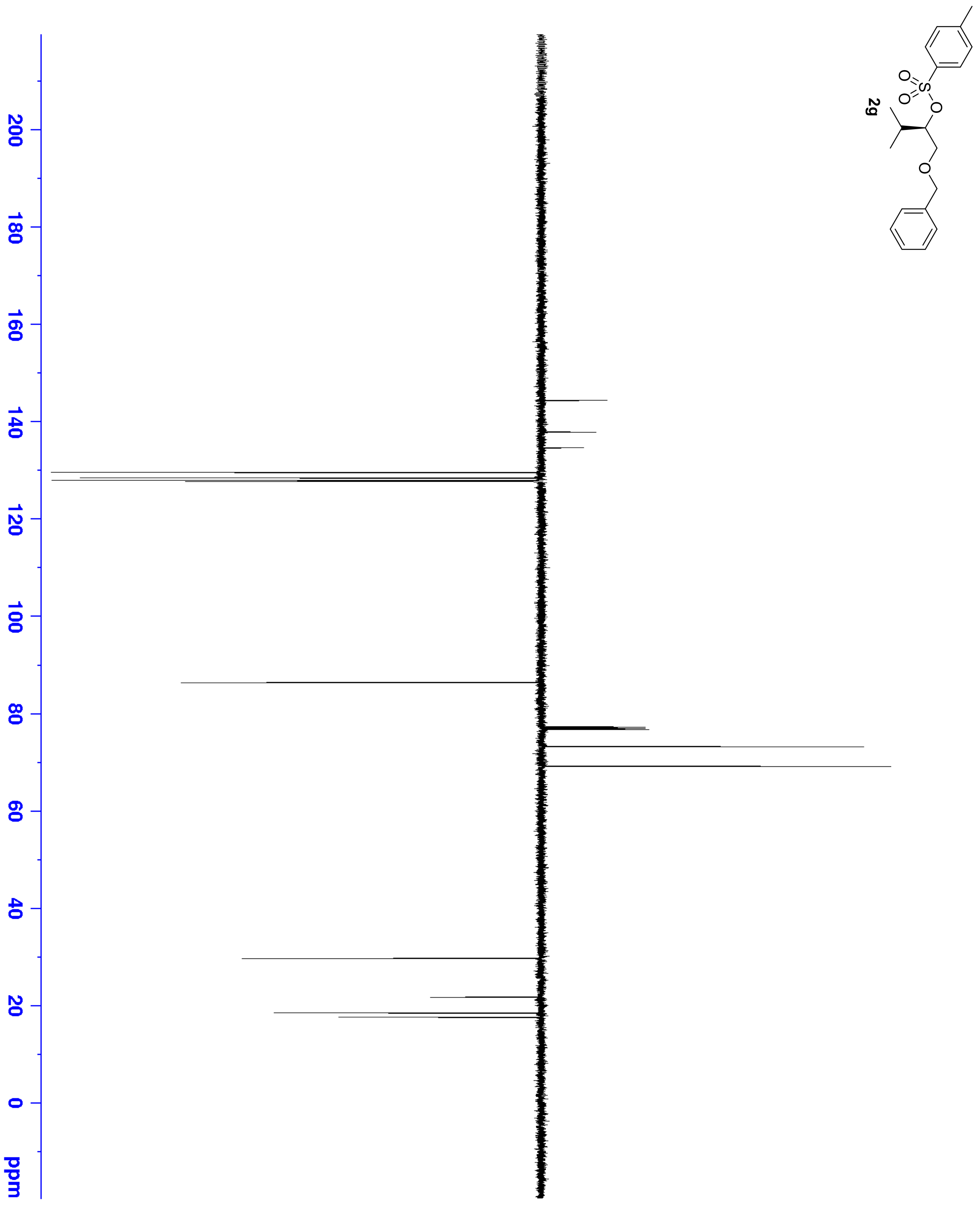
2g
200
180
160
140
120
100
80
60
40
20
0
ppm

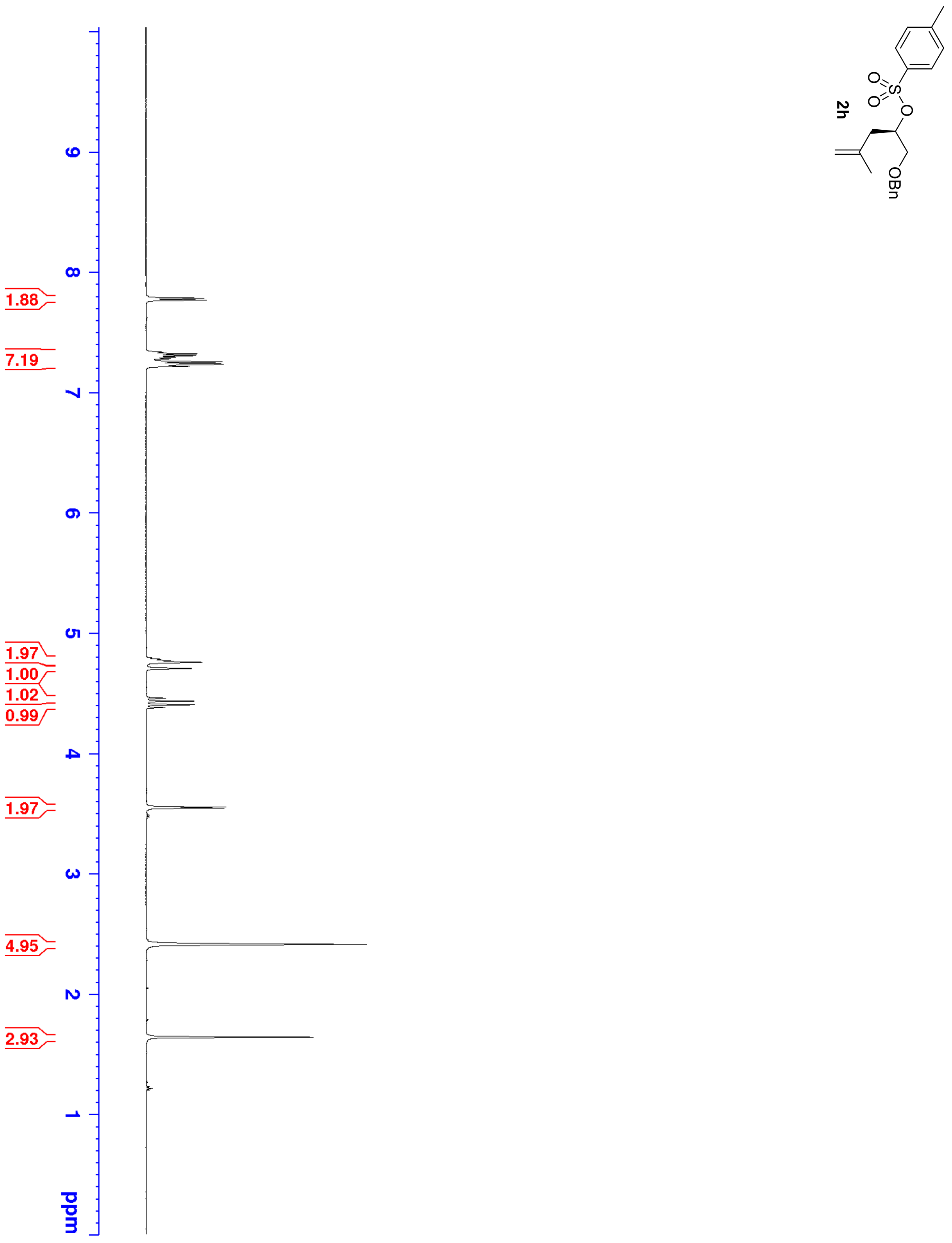
2h
OBn
1.88
7.19
1.97
1.00
1.02
0.99
1.97
4.95
2.93
9
8
7
6
5
4
3
2
1
ppm

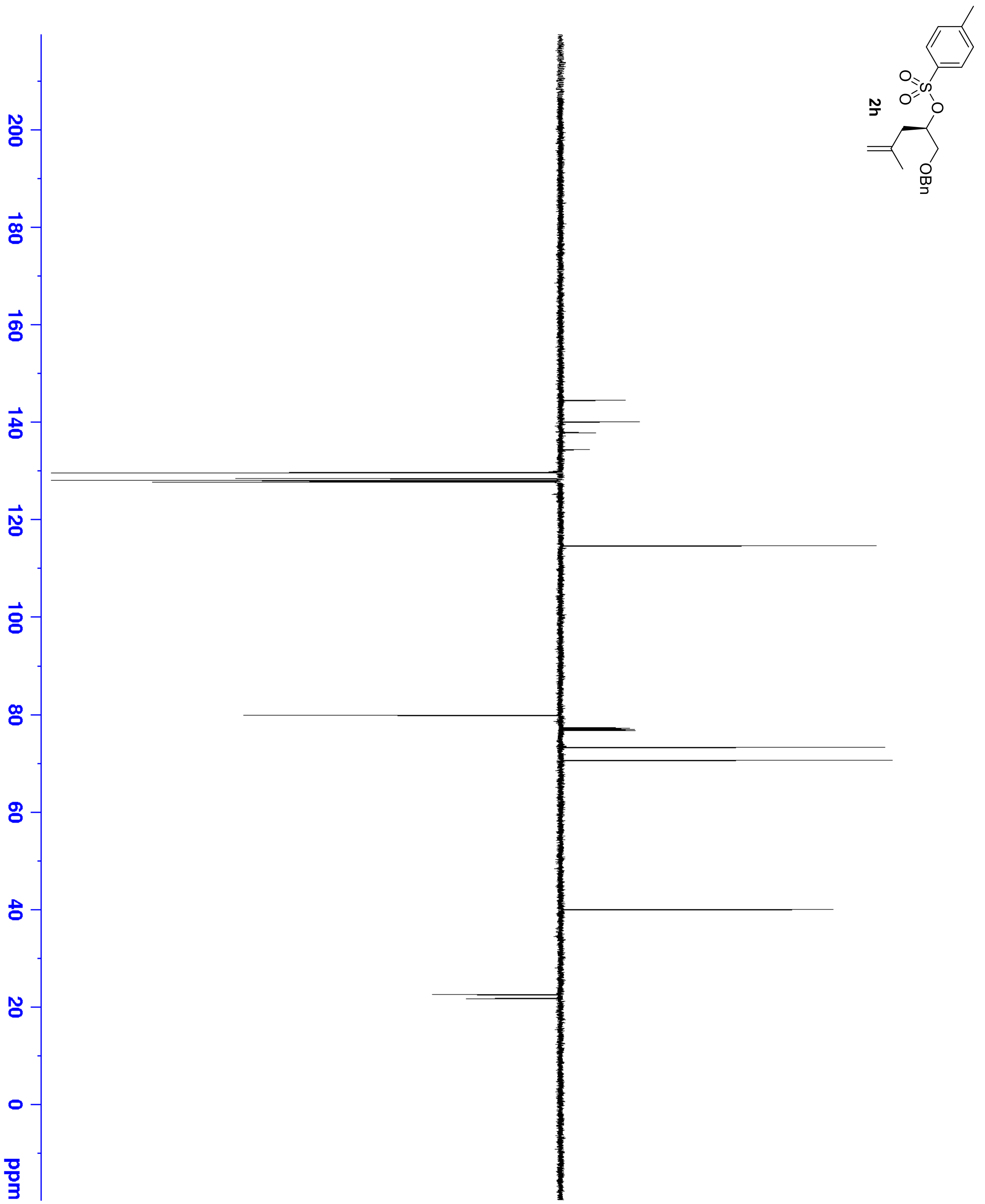
2h
OBn
ppm
200
180
160
140
120
100
80
60
40
20
0

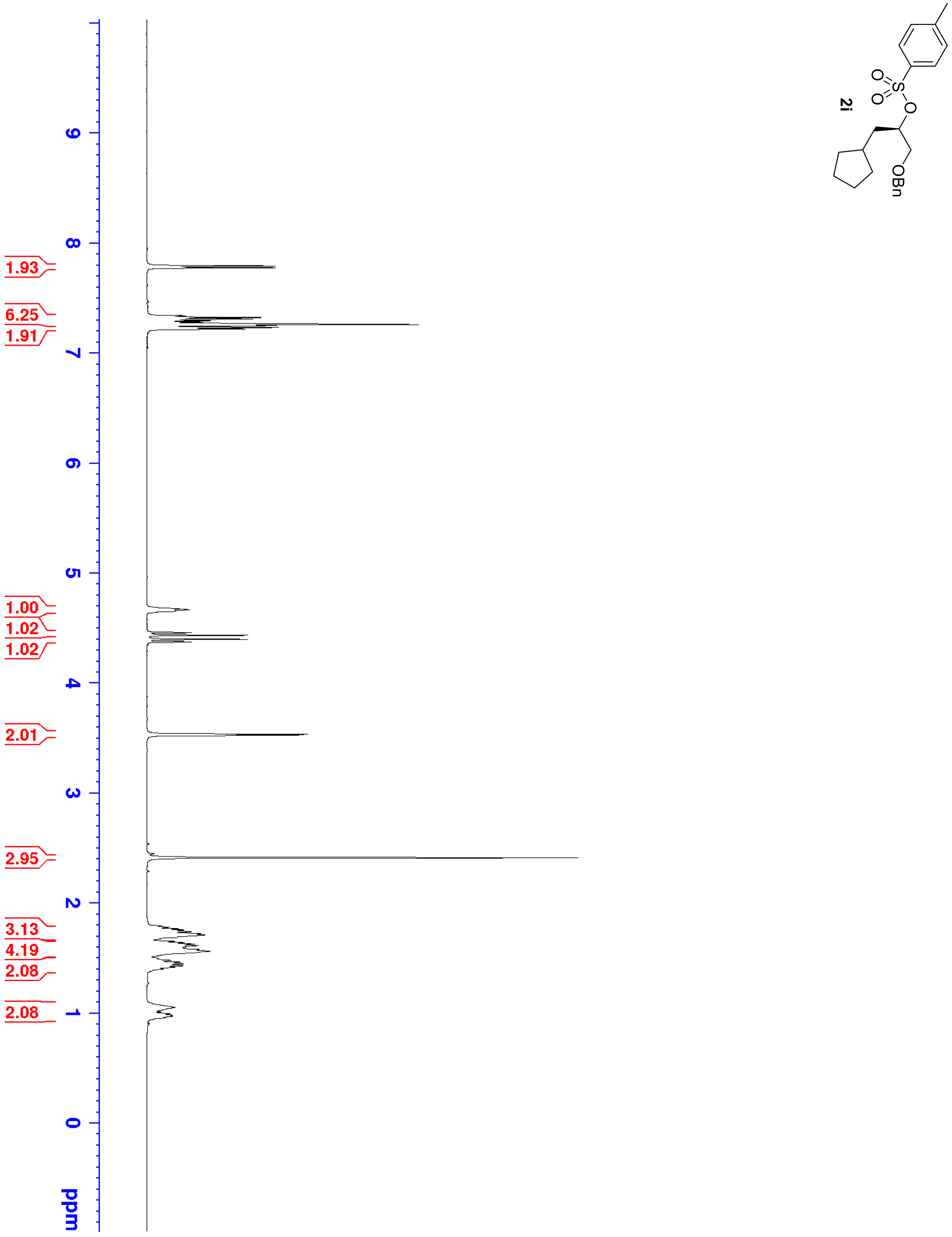
2i
OBn
1.93
6.25
1.91
1.00
1.02
1.02
2.01
2.95
3.13
4.19
2.08
2.08
ppm

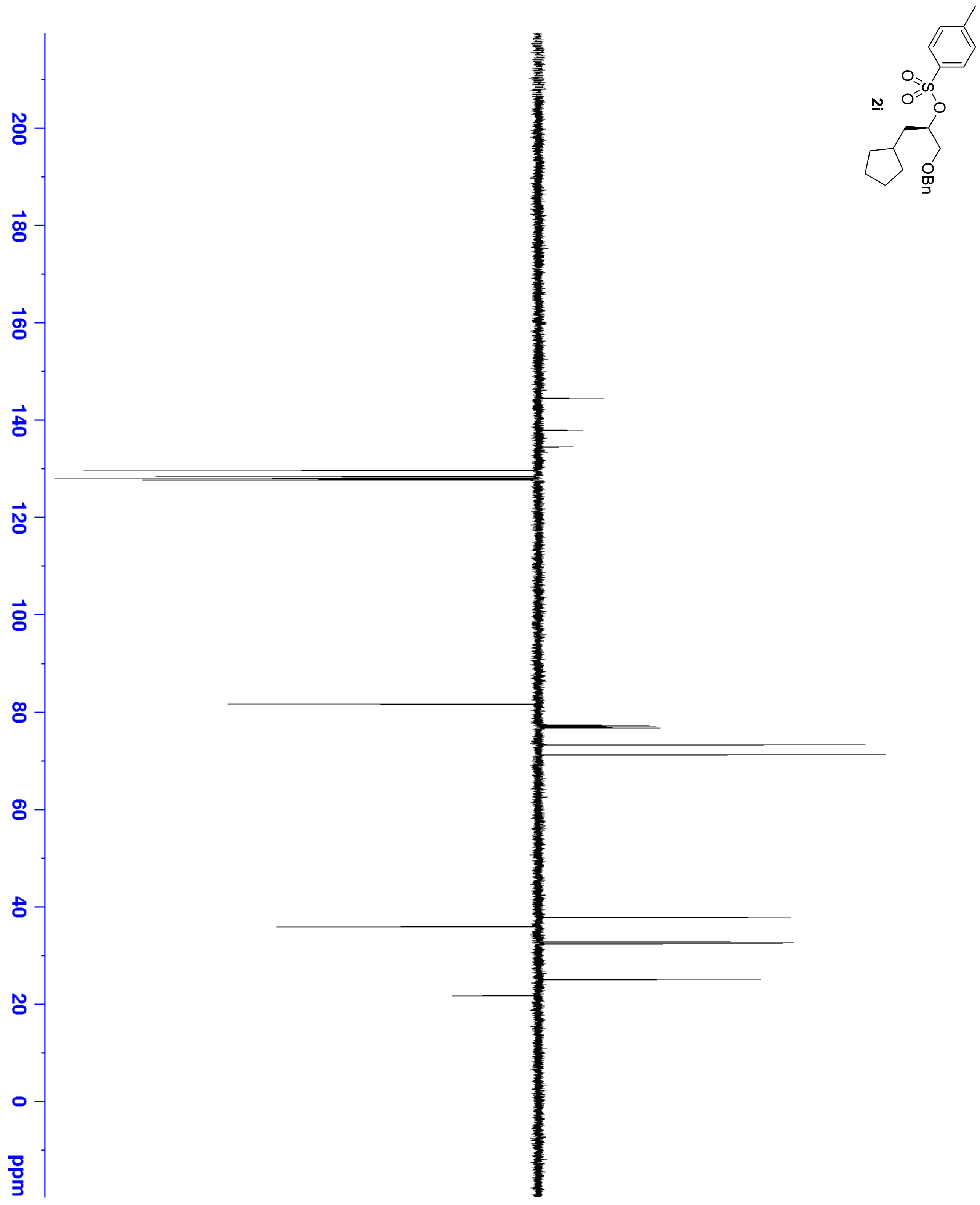
200
180
160
140
120
100
80
60
40
20
0
ppm
2i
OBn

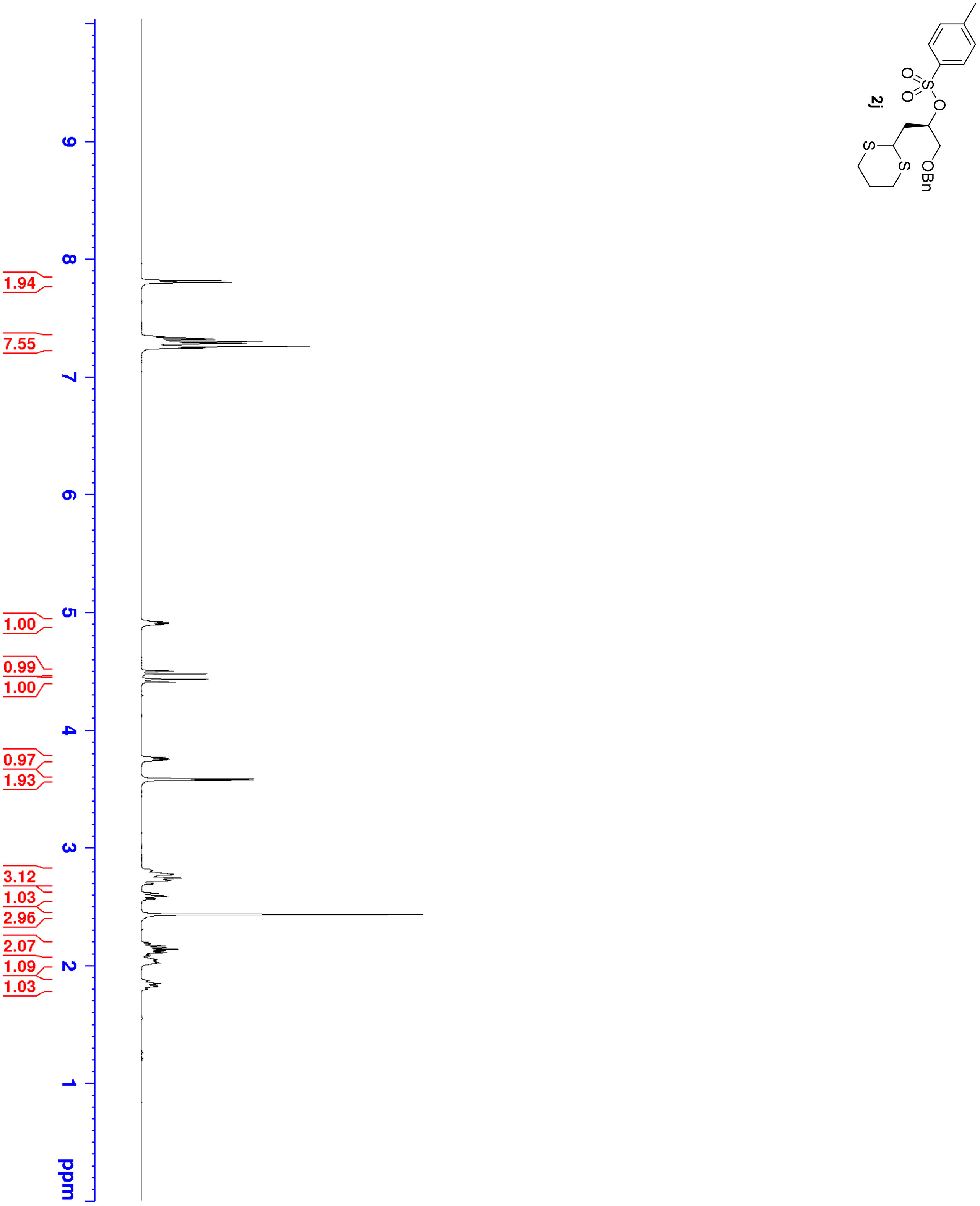
2j
OBn
S
S
O
O
O
1.94
7.55
1.00
0.99
1.00
0.97
1.93
3.12
1.03
2.96
2.07
1.09
1.03
9
8
7
6
5
4
3
2
1
ppm

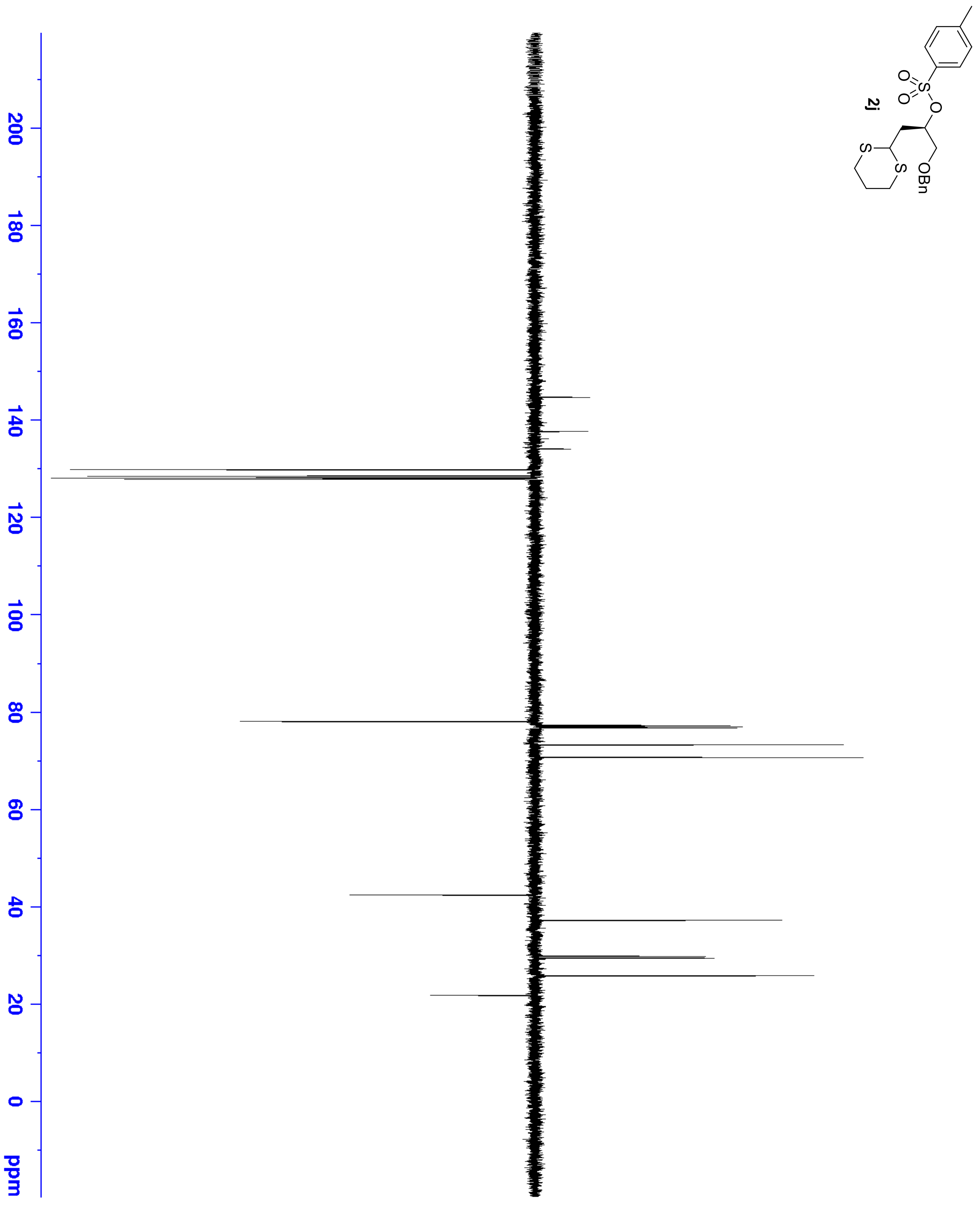
2j
OBn
S
S
O
ppm
200
180
160
140
120
100
80
60
40
20
0

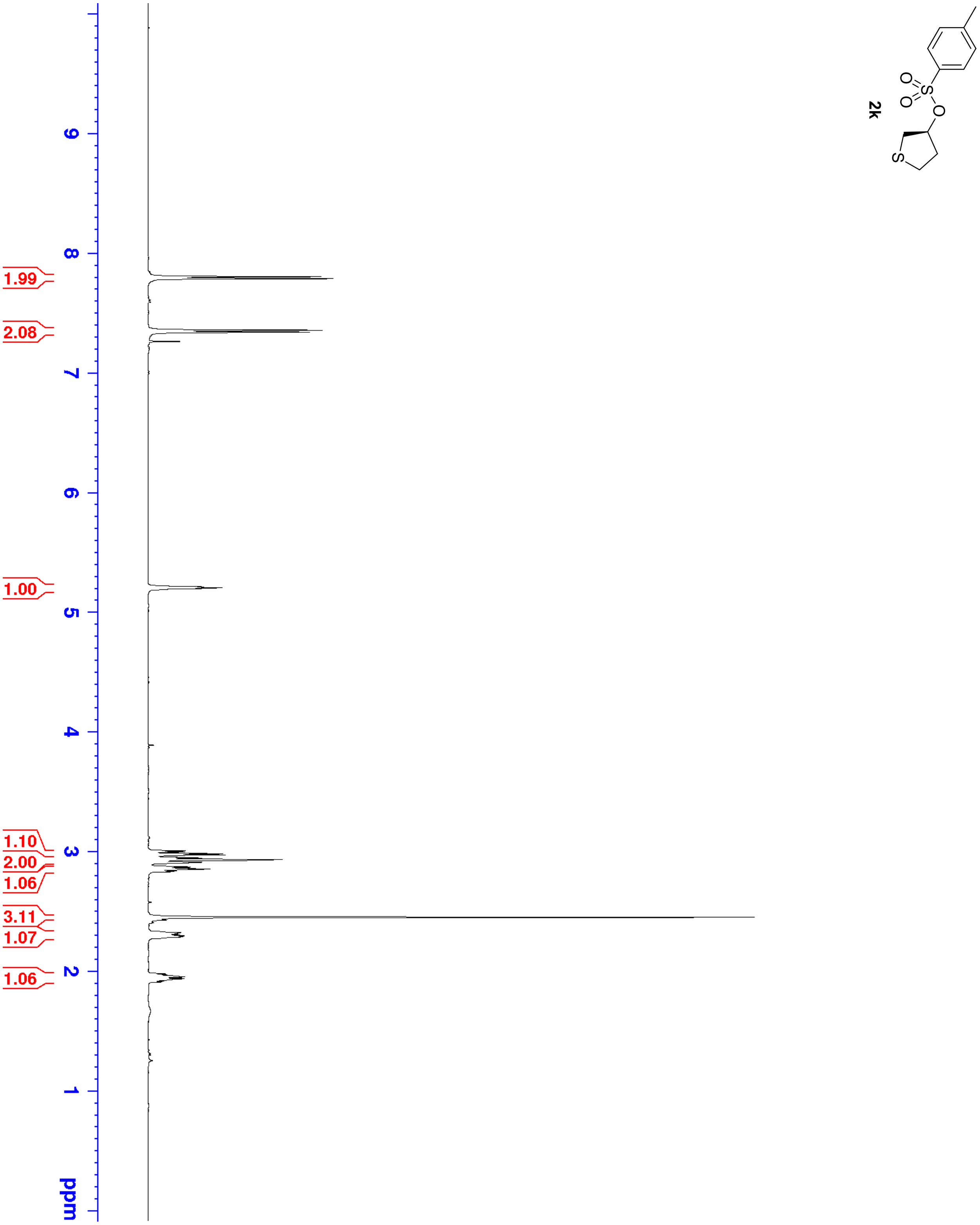

2k
1.99
2.08
1.00
1.10
2.00
1.06
3.11
1.07
1.06
9
8
7
6
5
4
3
2
1
ppm

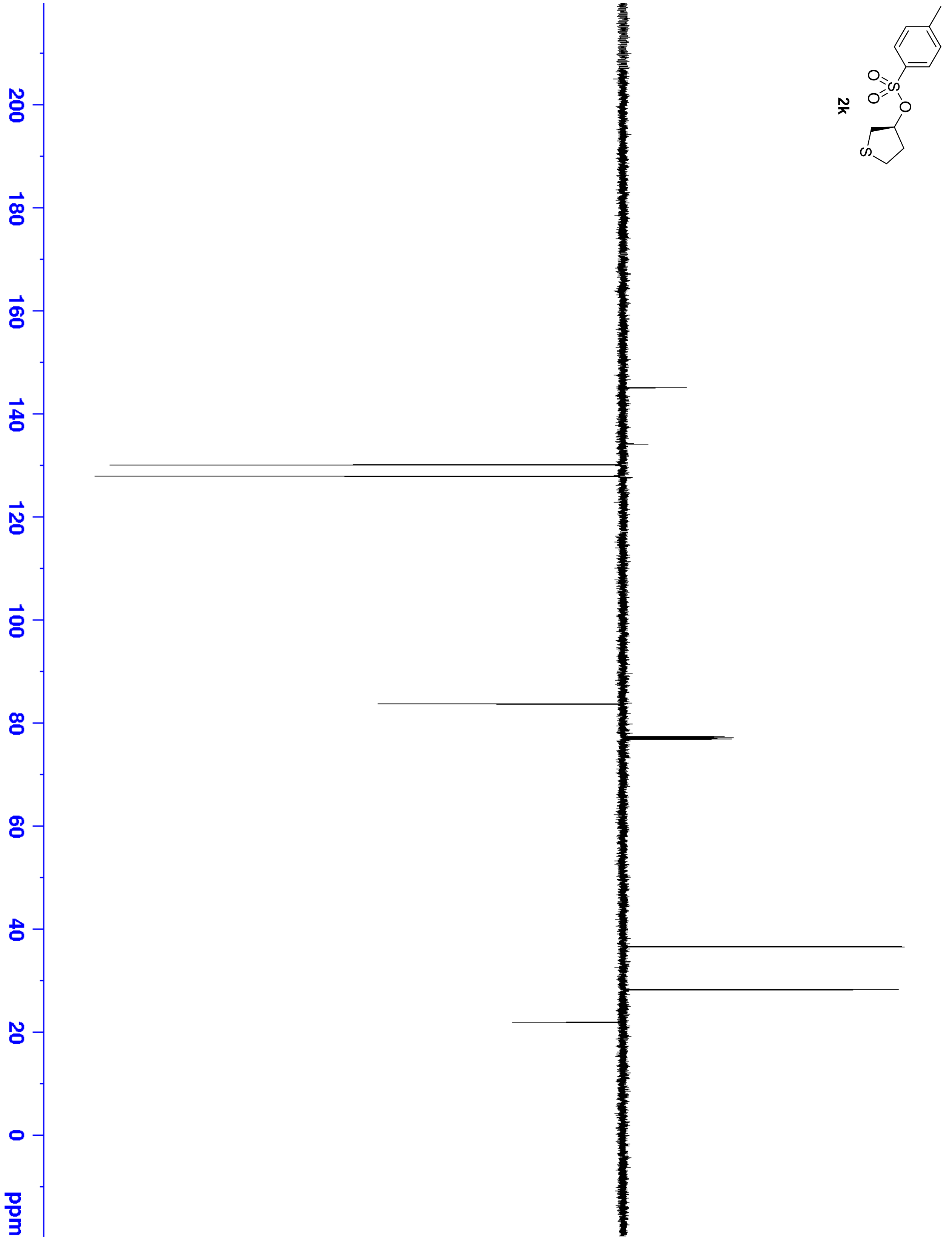
2k
200
180
160
140
120
100
80
60
40
20
0
ppm

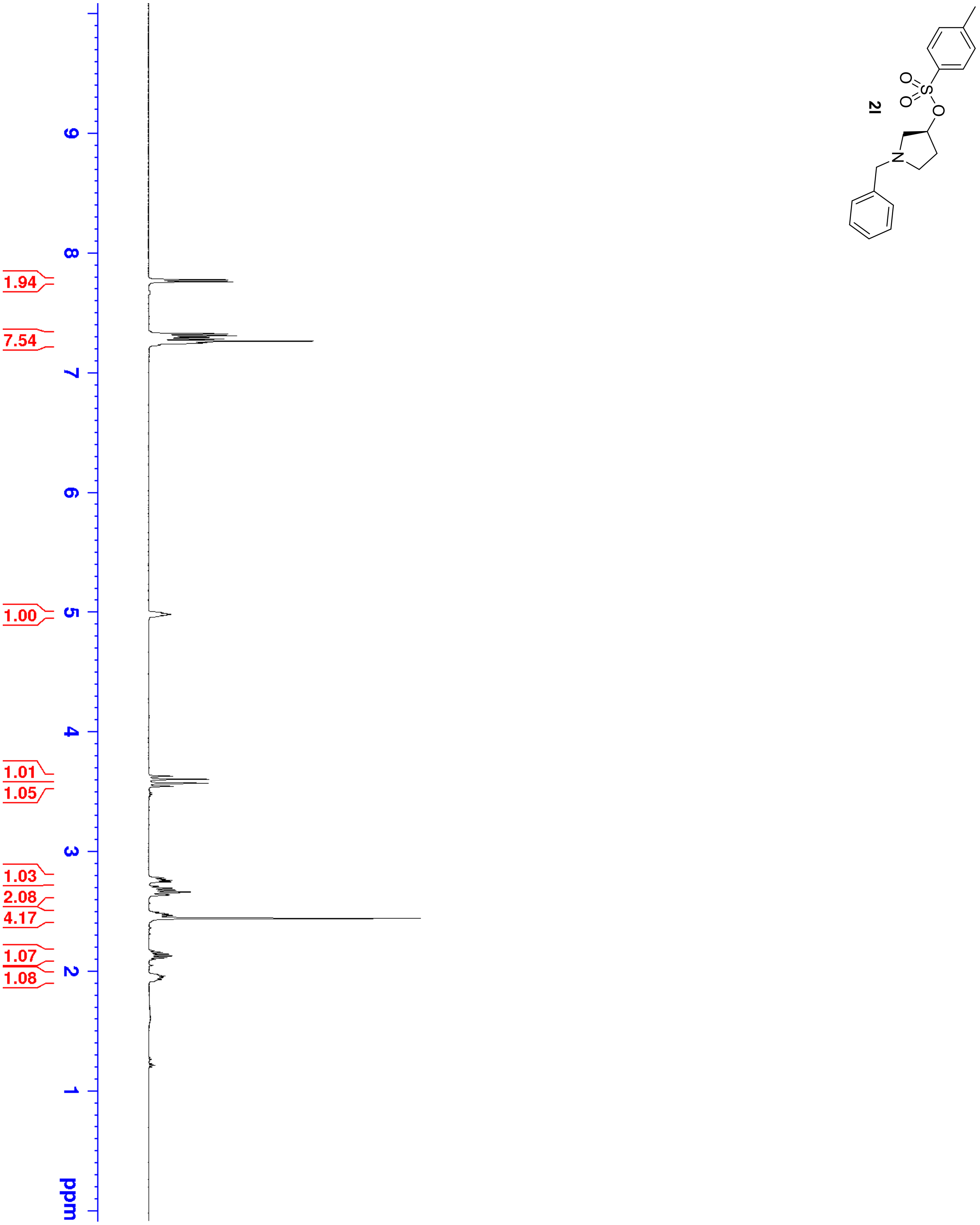
2l
ppm
1
2
3
4
5
6
7
8
9
1.94
7.54
1.00
1.01
1.05
1.03
2.08
4.17
1.07
1.08
N
O
S
O
O

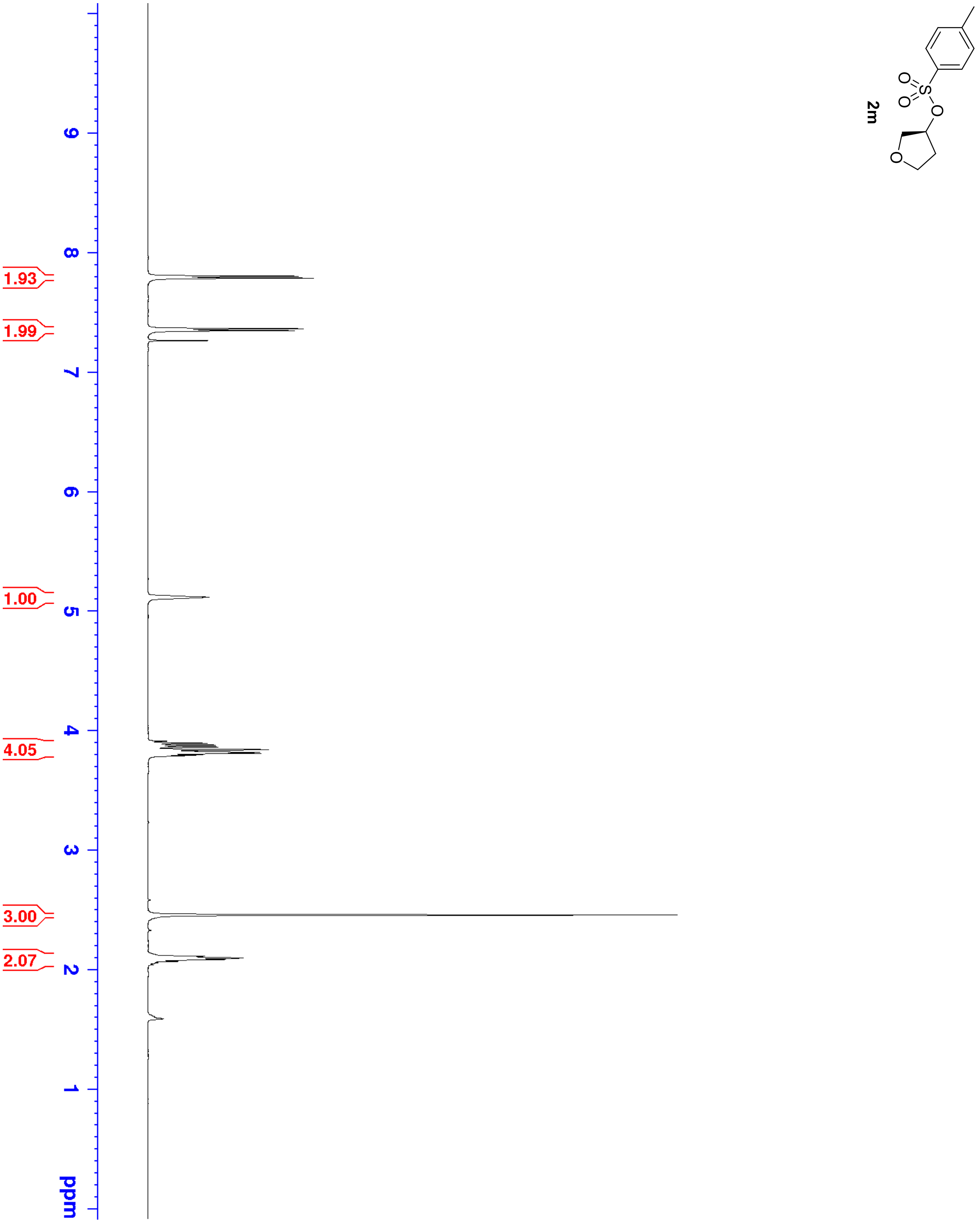
2m
1.93
1.99
1.00
4.05
3.00
2.07
9
8
7
6
5
4
3
2
1
ppm

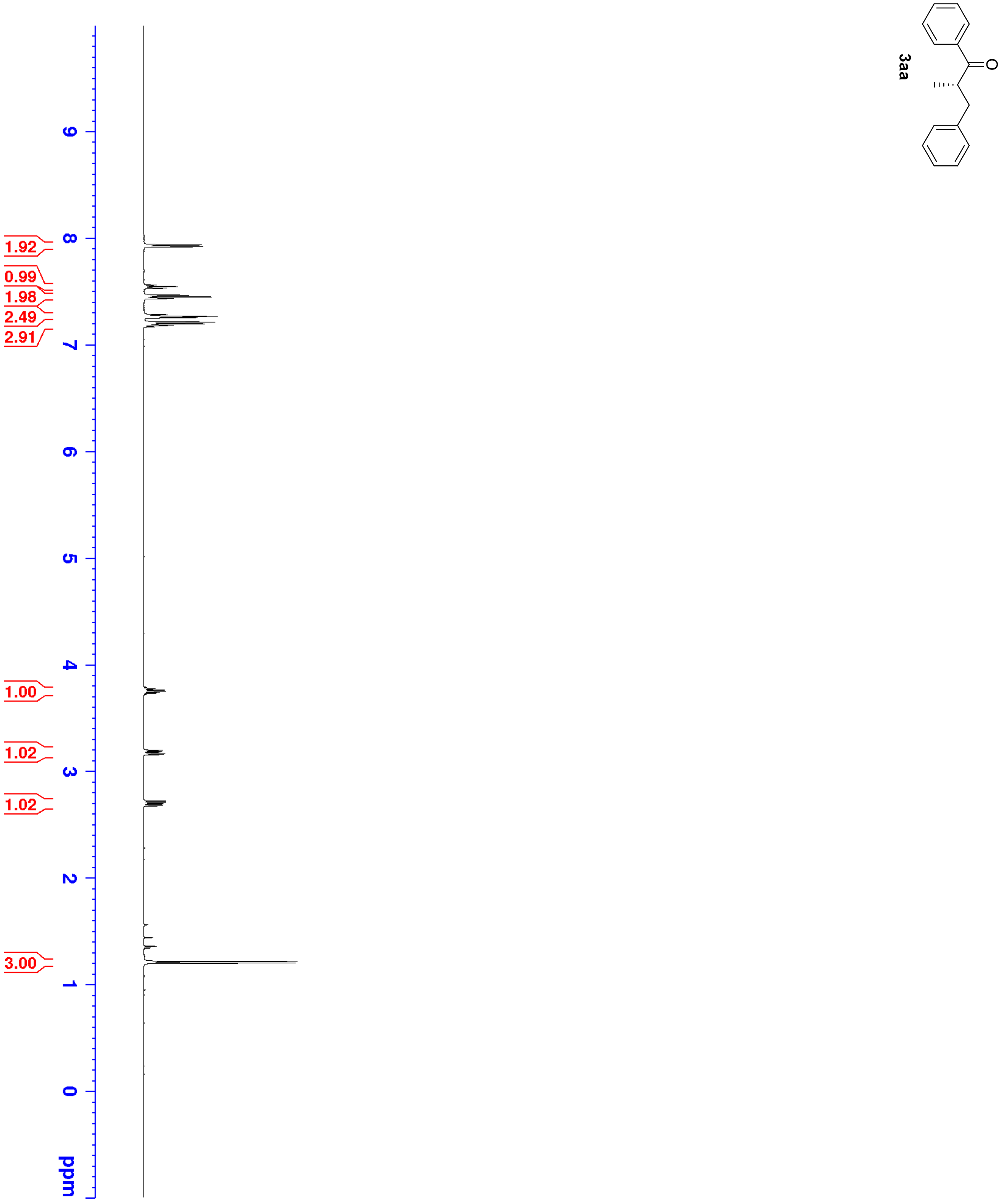
3aa
1.92
0.99
1.98
2.49
2.91
1.00
1.02
1.02
3.00
9
8
7
6
5
4
3
2
1
0
ppm

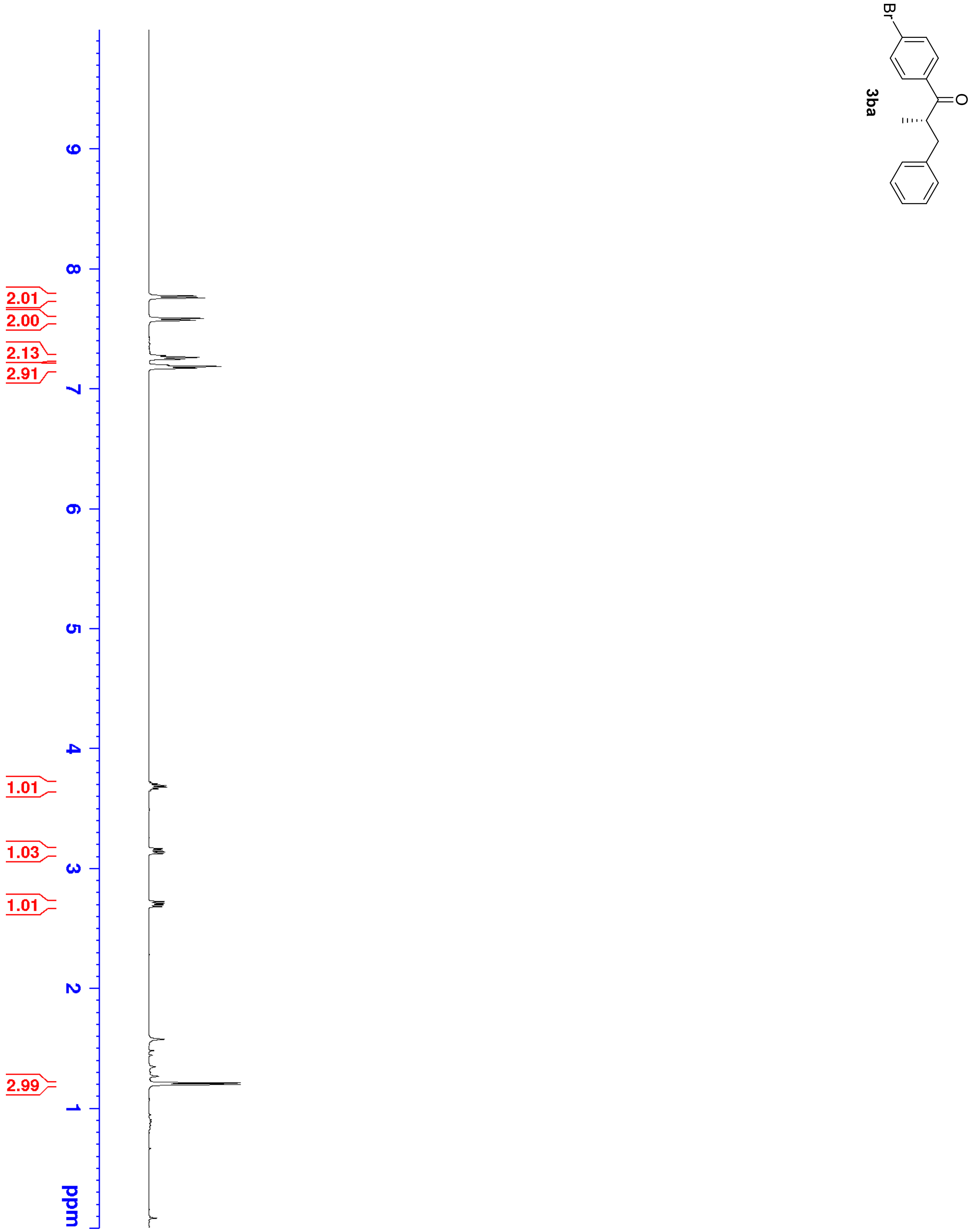
3ba
ppm

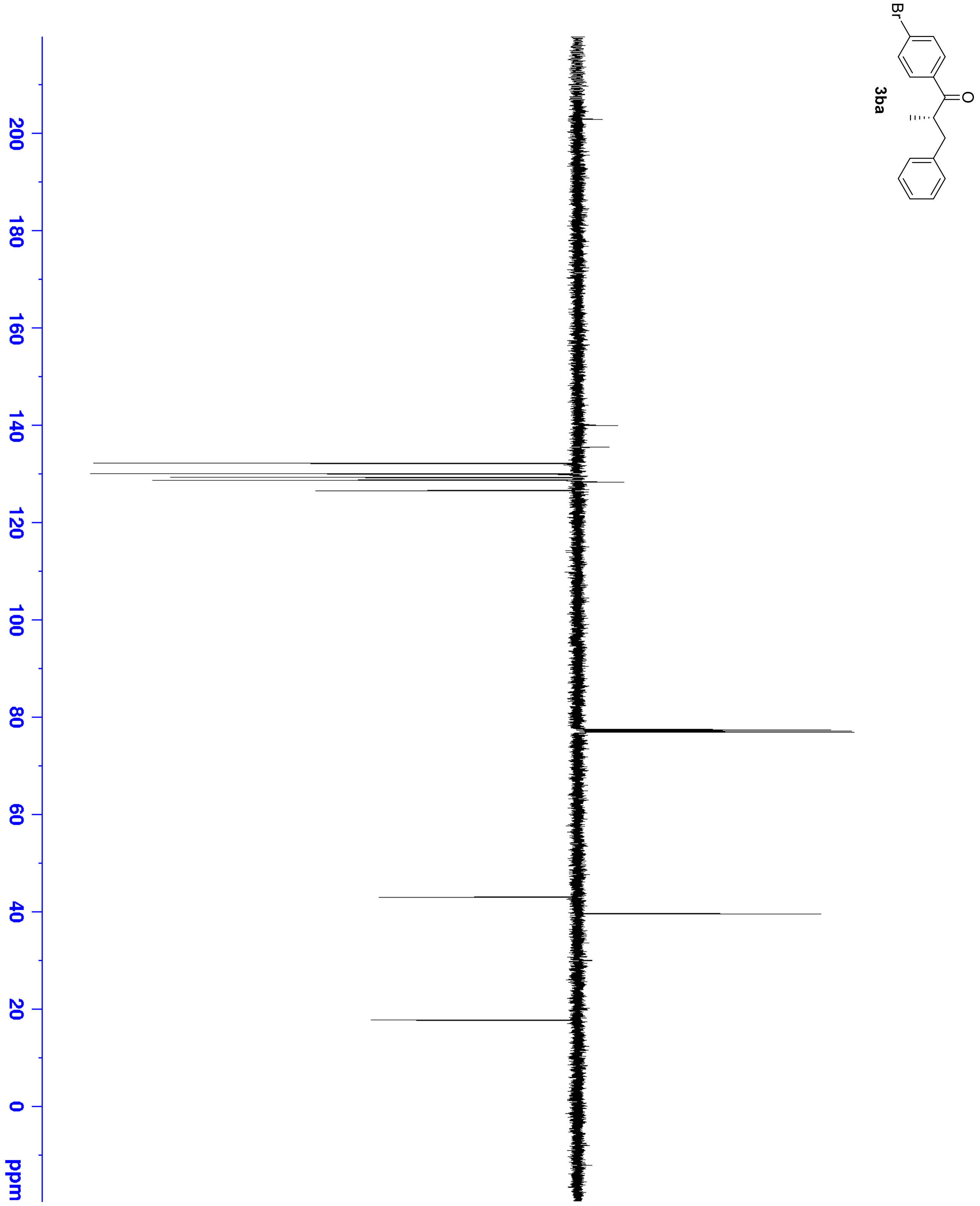
Br
O
3ba
200
180
160
140
120
100
80
60
40
20
0
ppm

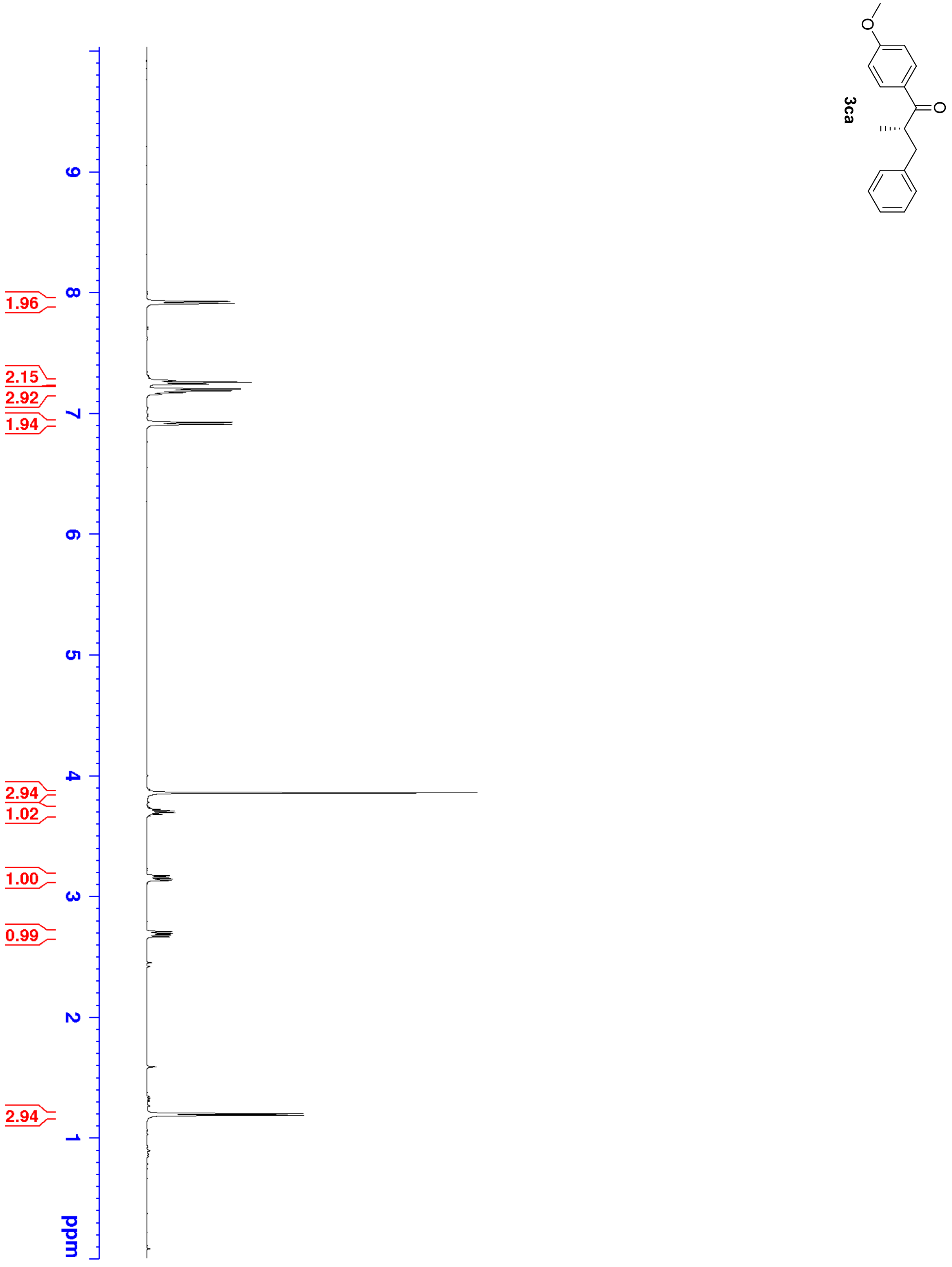
3ca
1.96
2.15
2.92
1.94
2.94
1.02
1.00
0.99
2.94
9
8
7
6
5
4
3
2
1
ppm

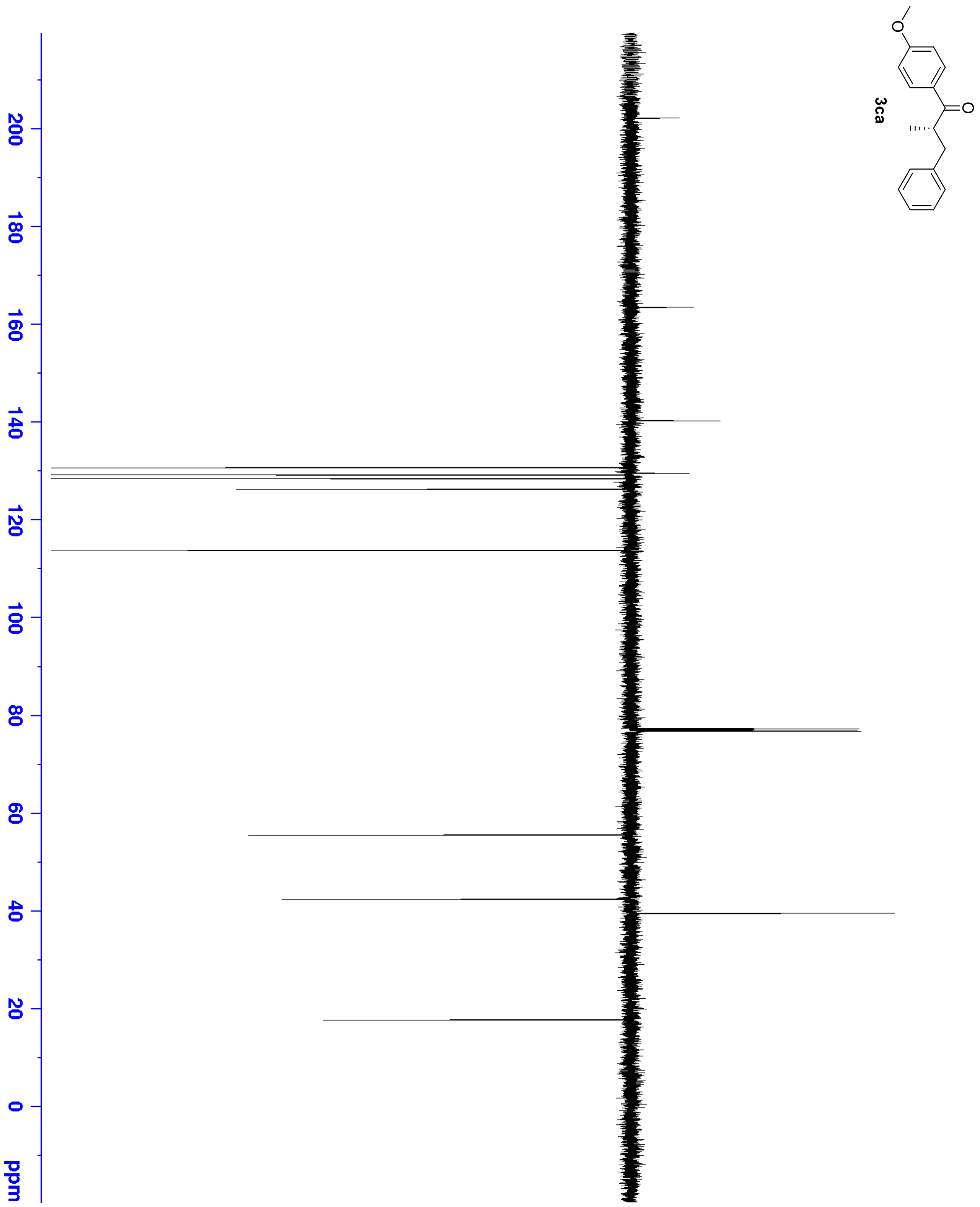
3ca
200
180
160
140
120
100
80
60
40
20
0
ppm

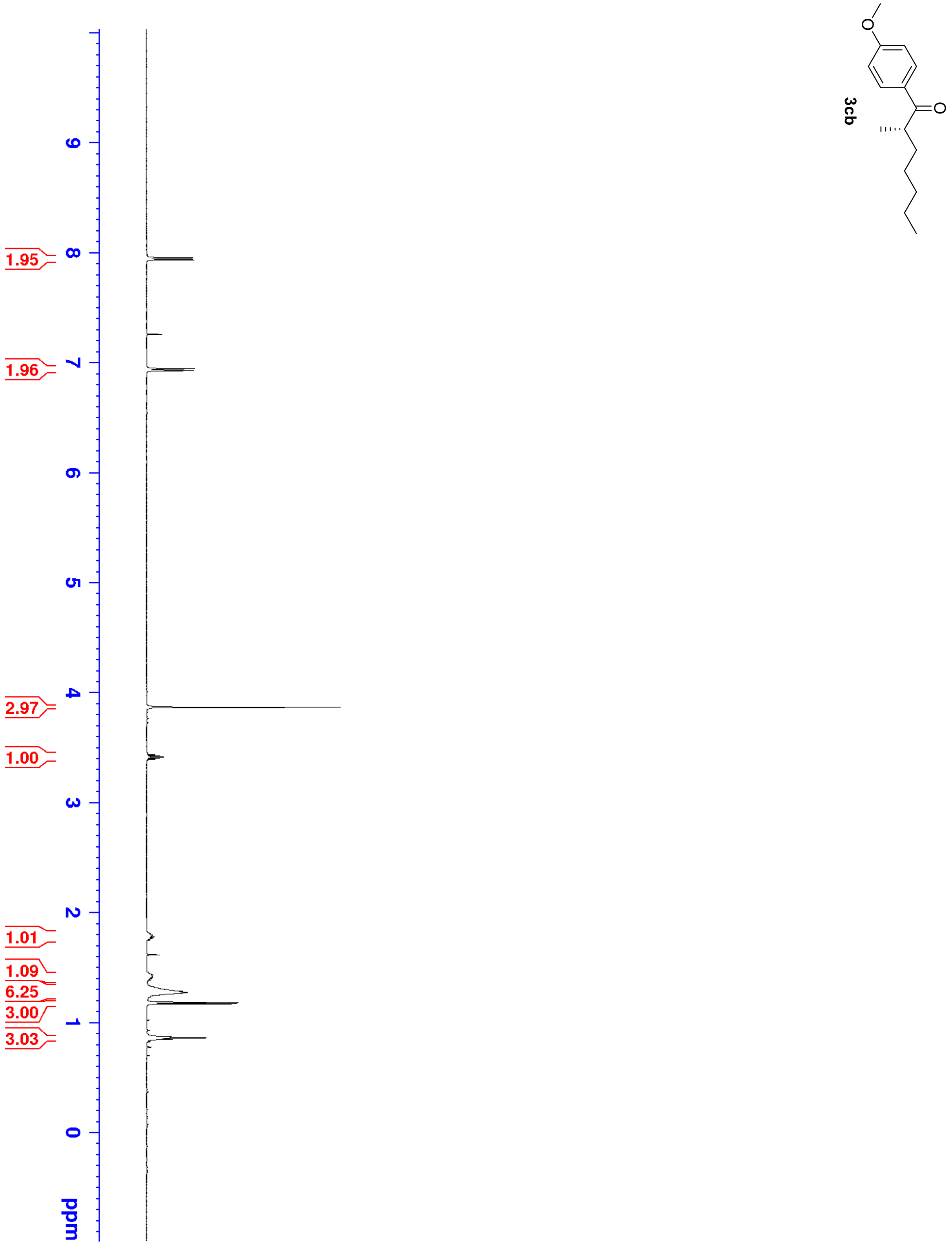
3cb
1.95
1.96
2.97
1.00
1.01
1.09
6.25
3.00
3.03
9
8
7
6
5
4
3
2
1
0
ppm

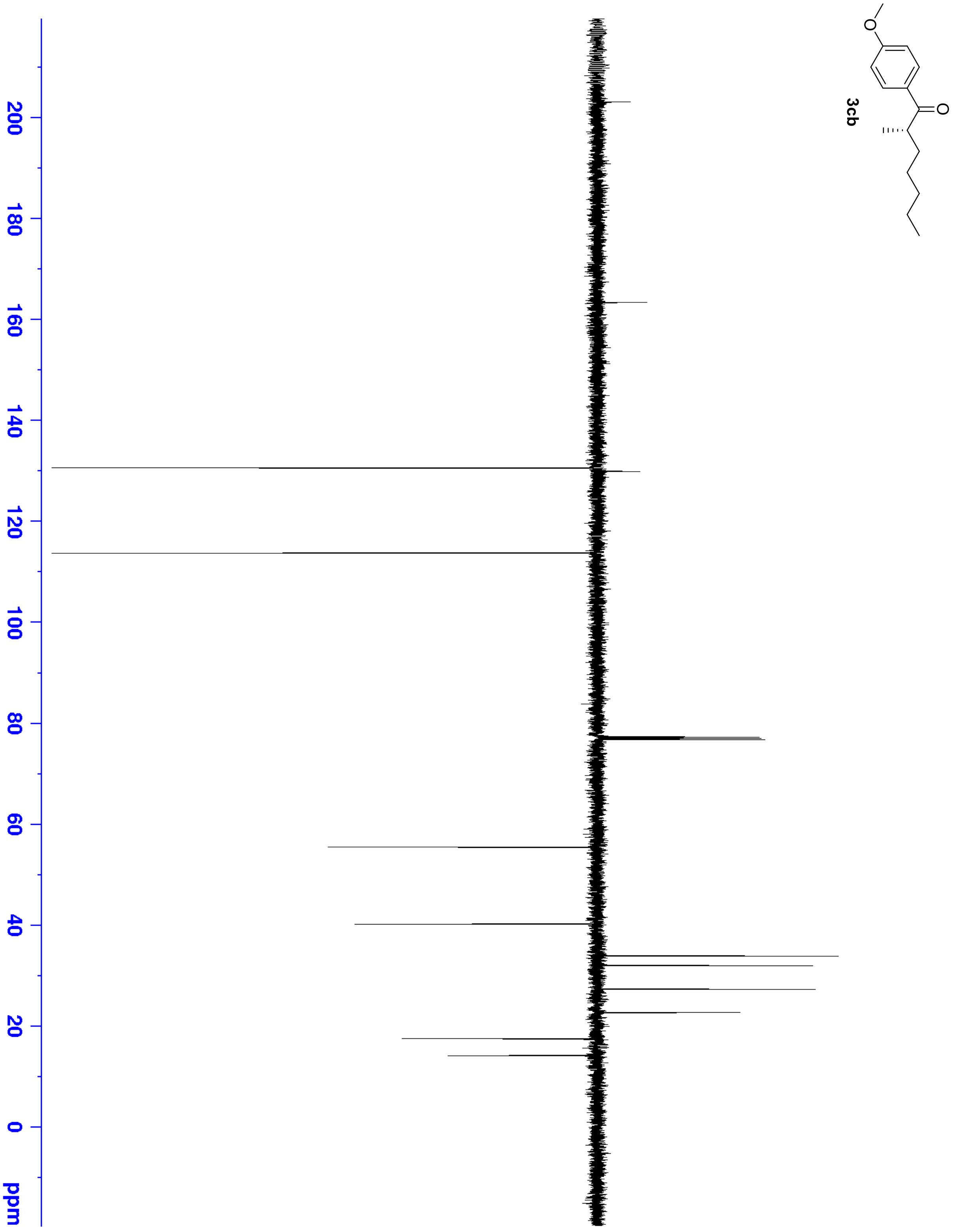
3cb
200
180
160
140
120
100
80
60
40
20
0
ppm

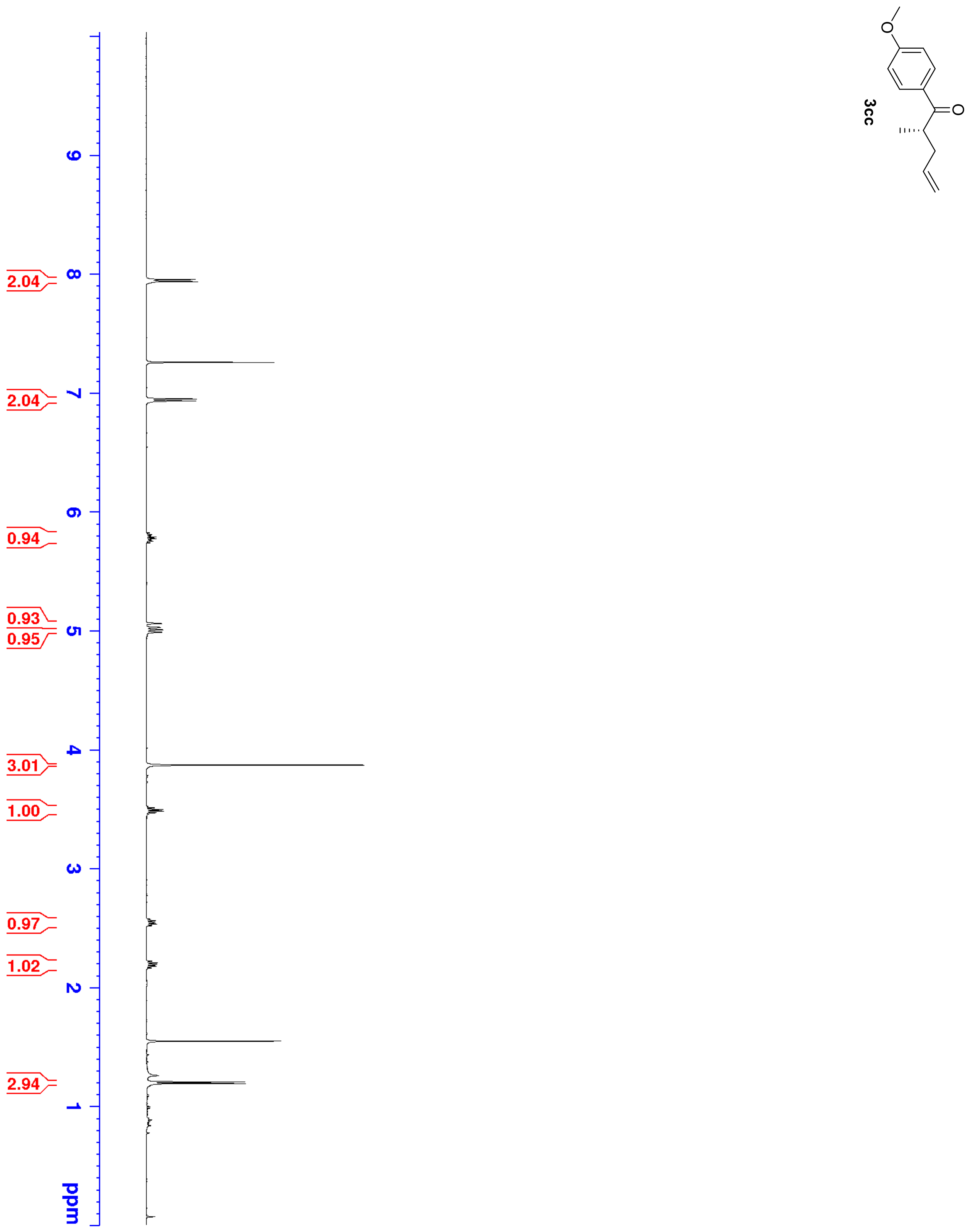
3cc
9
8
7
6
5
4
3
2
1
ppm
2.04
2.04
0.94
0.93
0.95
3.01
1.00
0.97
1.02
2.94

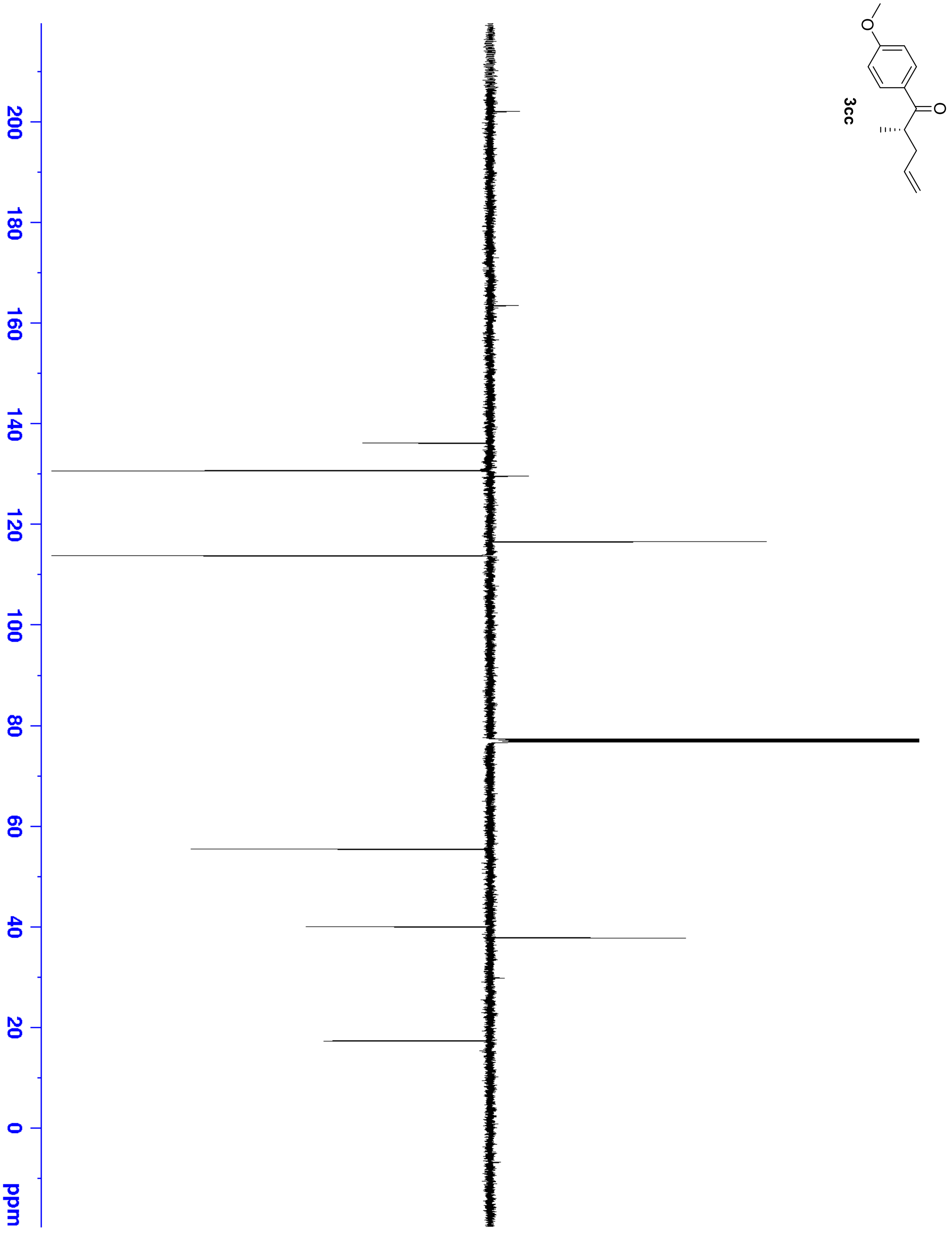
3cc
200
180
160
140
120
100
80
60
40
20
0
ppm

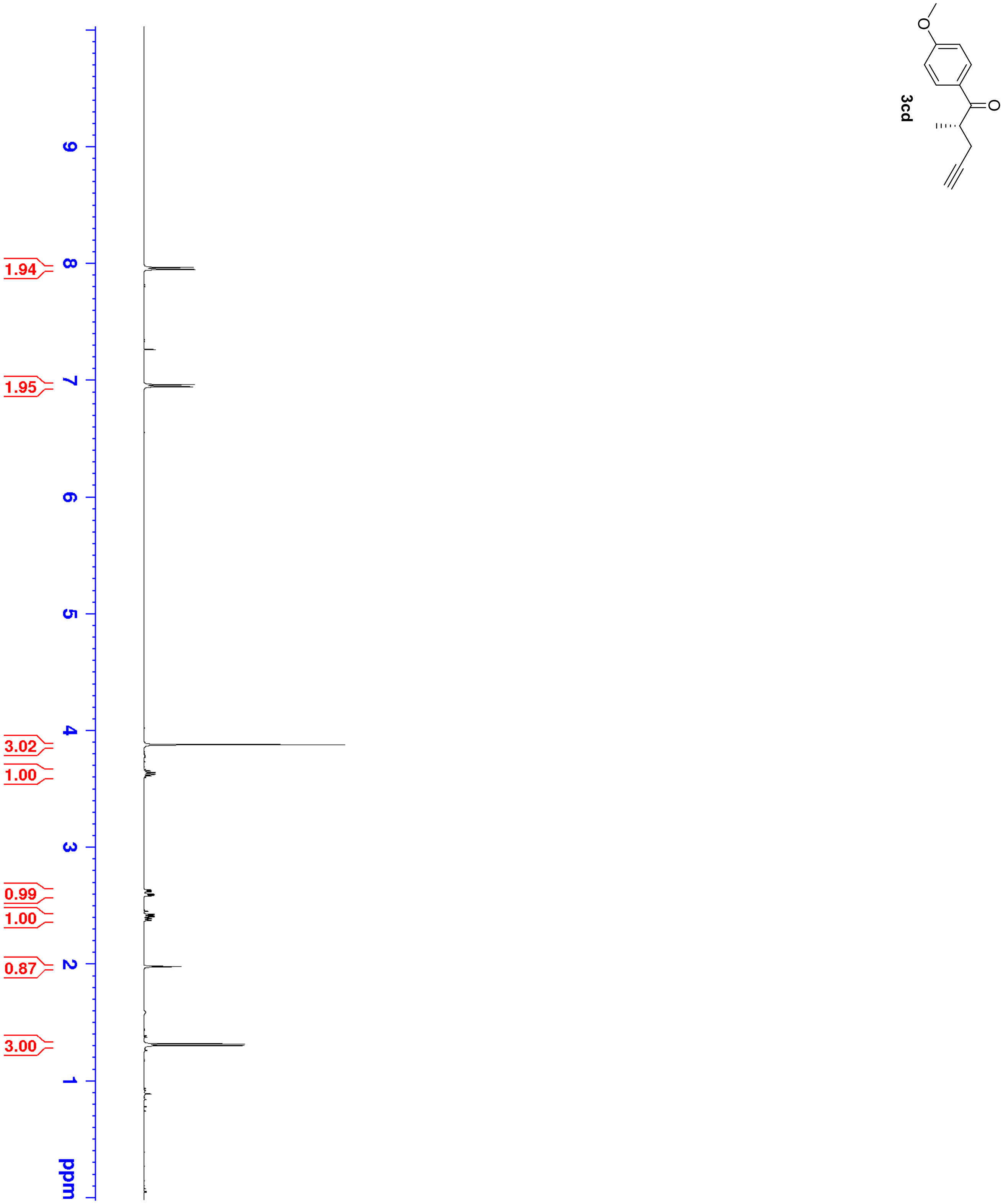
3cd
1.94
1.95
3.02
1.00
0.99
1.00
0.87
3.00
9
8
7
6
5
4
3
2
1
ppm

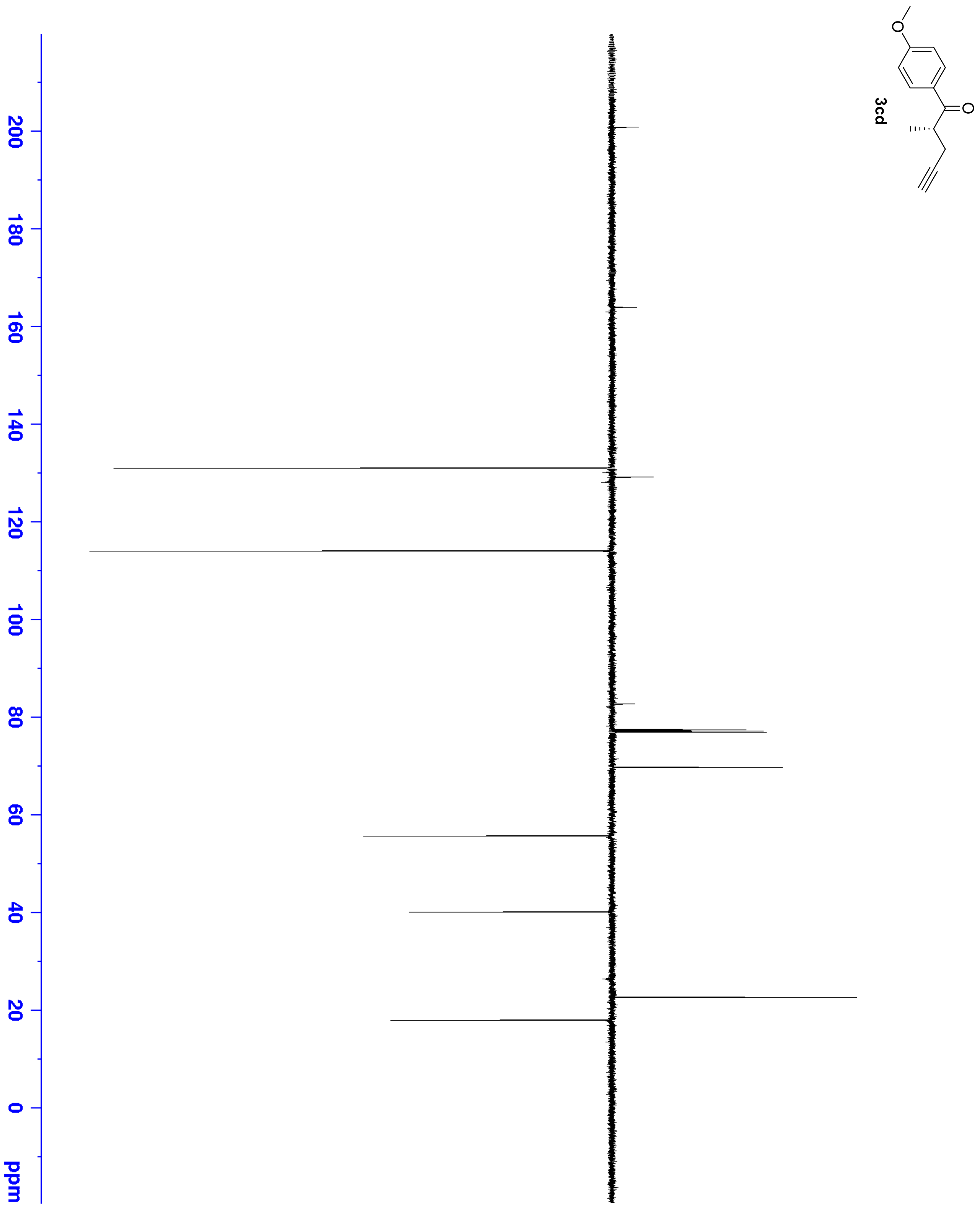
3cd
200
180
160
140
120
100
80
60
40
20
0
ppm

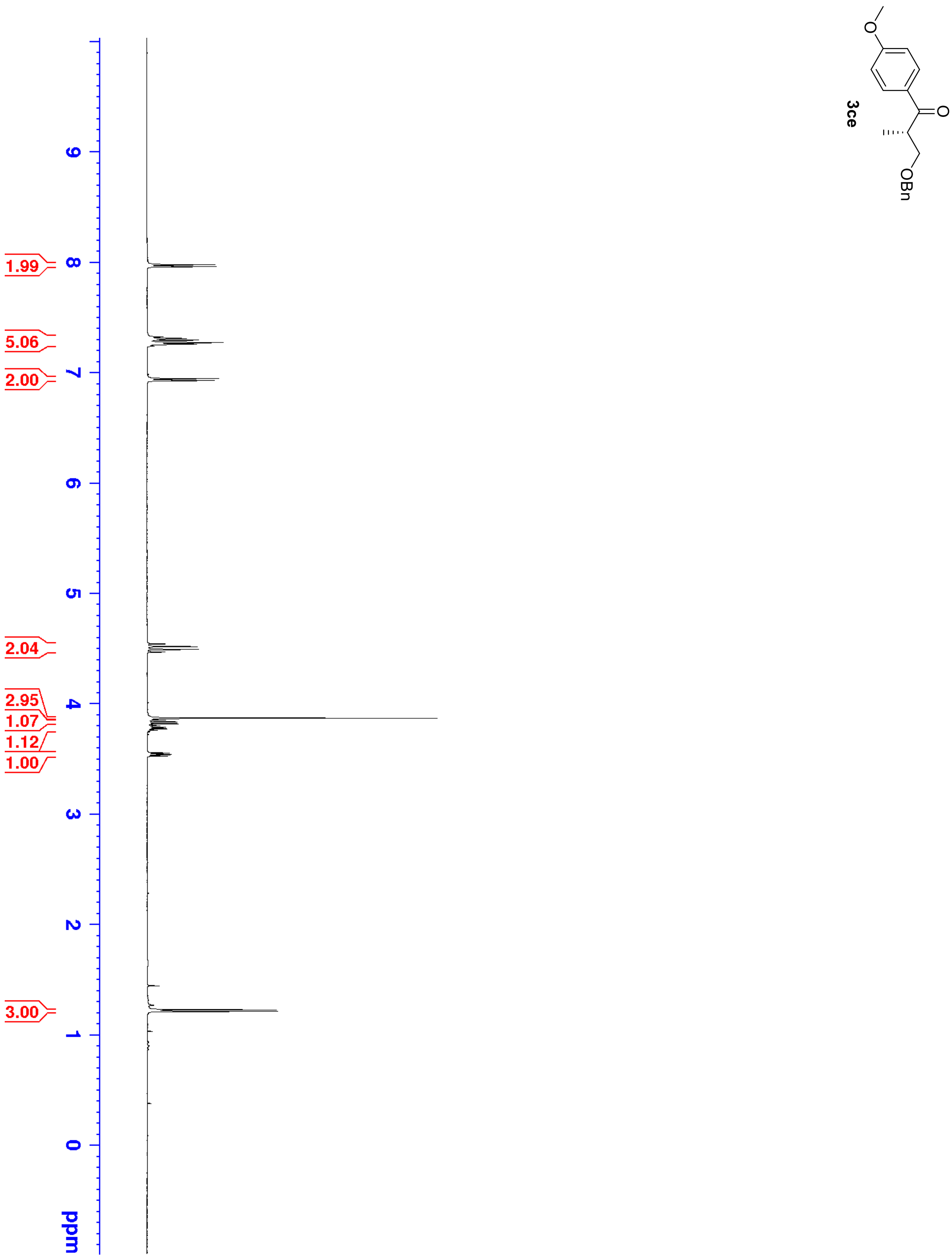
3ce
OBn
O
1.99
5.06
2.00
2.04
2.95
1.07
1.12
1.00
3.00
9
8
7
6
5
4
3
2
1
0
ppm

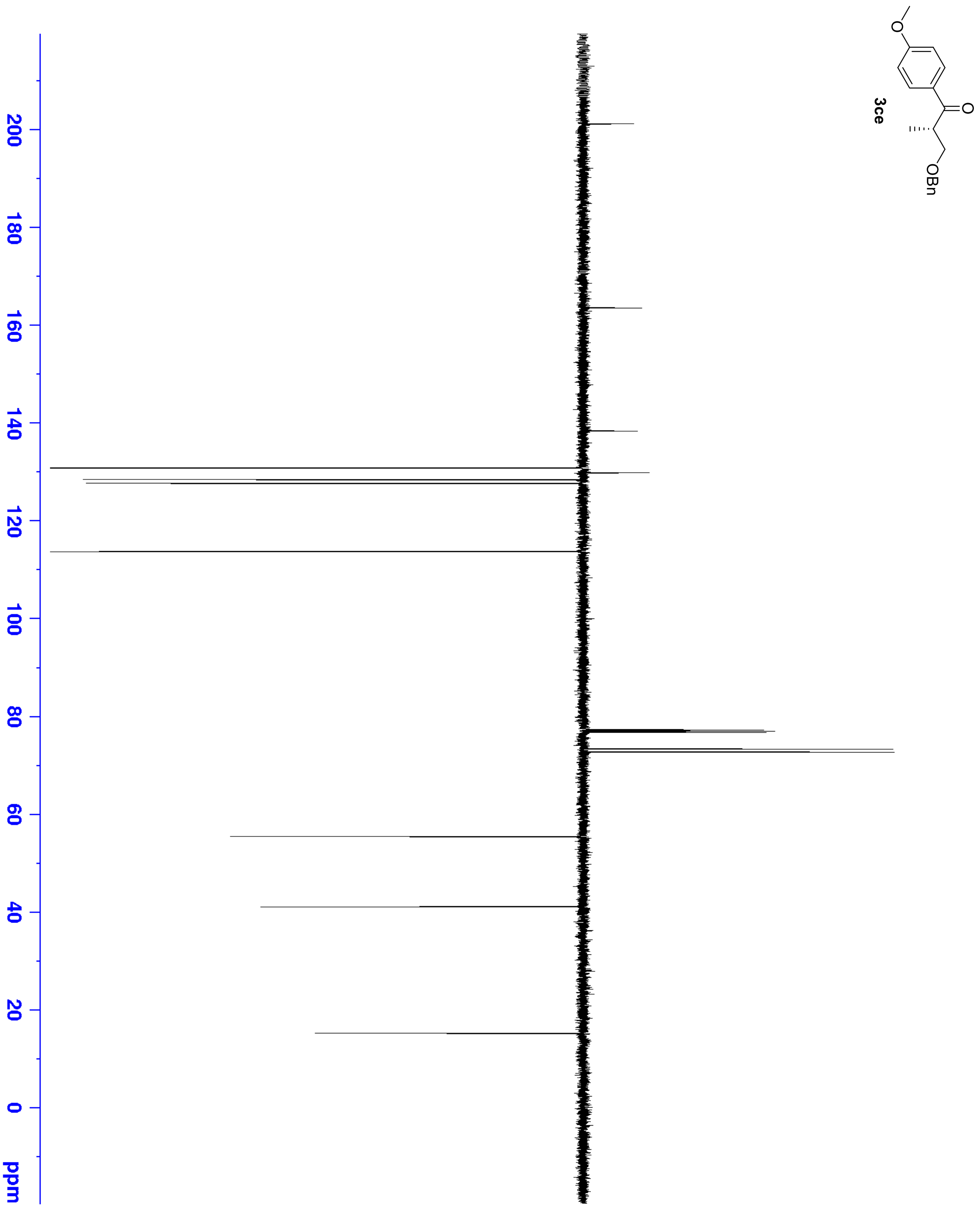
3ce
OBn
O
200
180
160
140
120
100
80
60
40
20
0
ppm

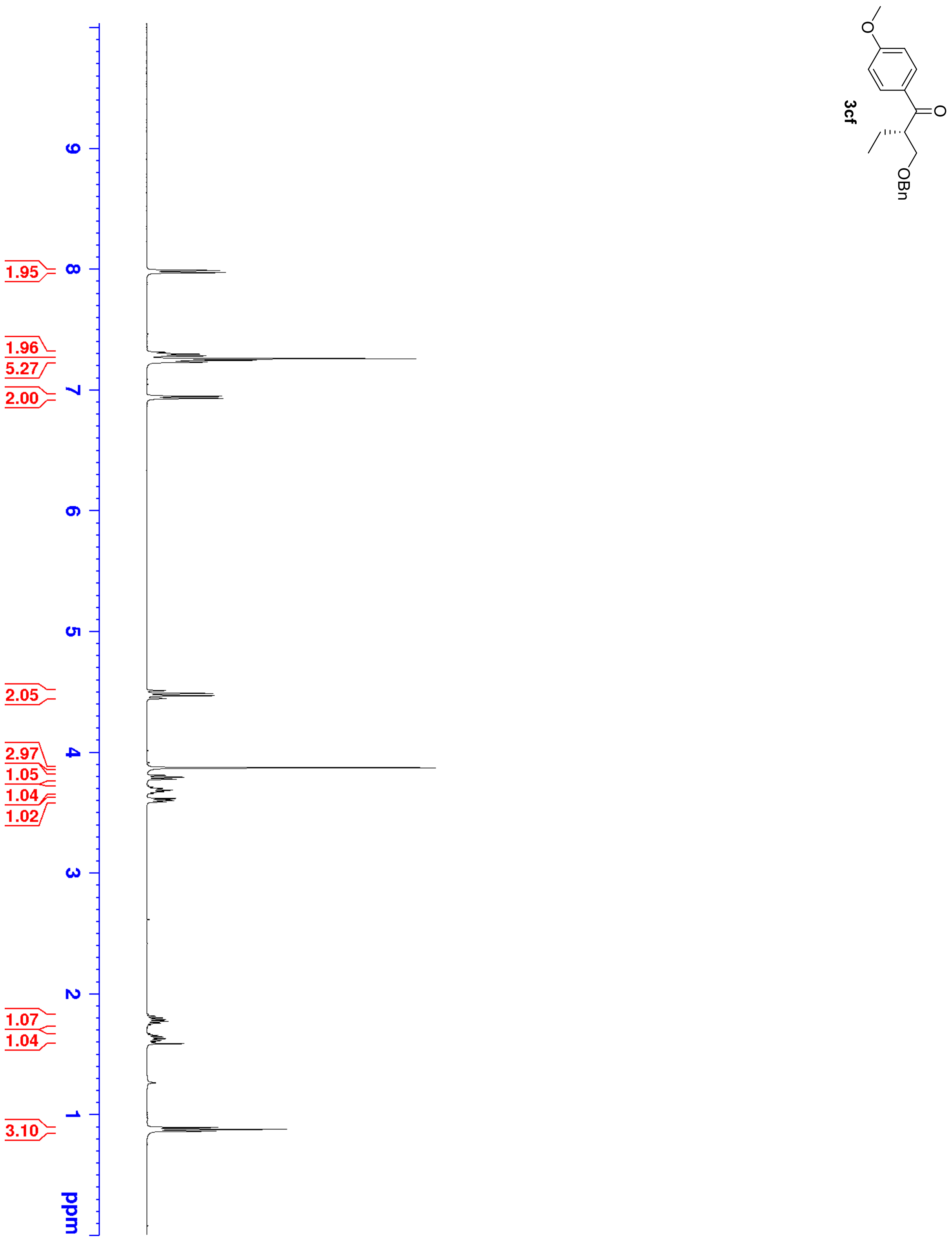

3cf
O
OBn
1.95
1.96
5.27
2.00
2.05
2.97
1.05
1.04
1.02
1.07
1.04
3.10
9
8
7
6
5
4
3
2
1
ppm

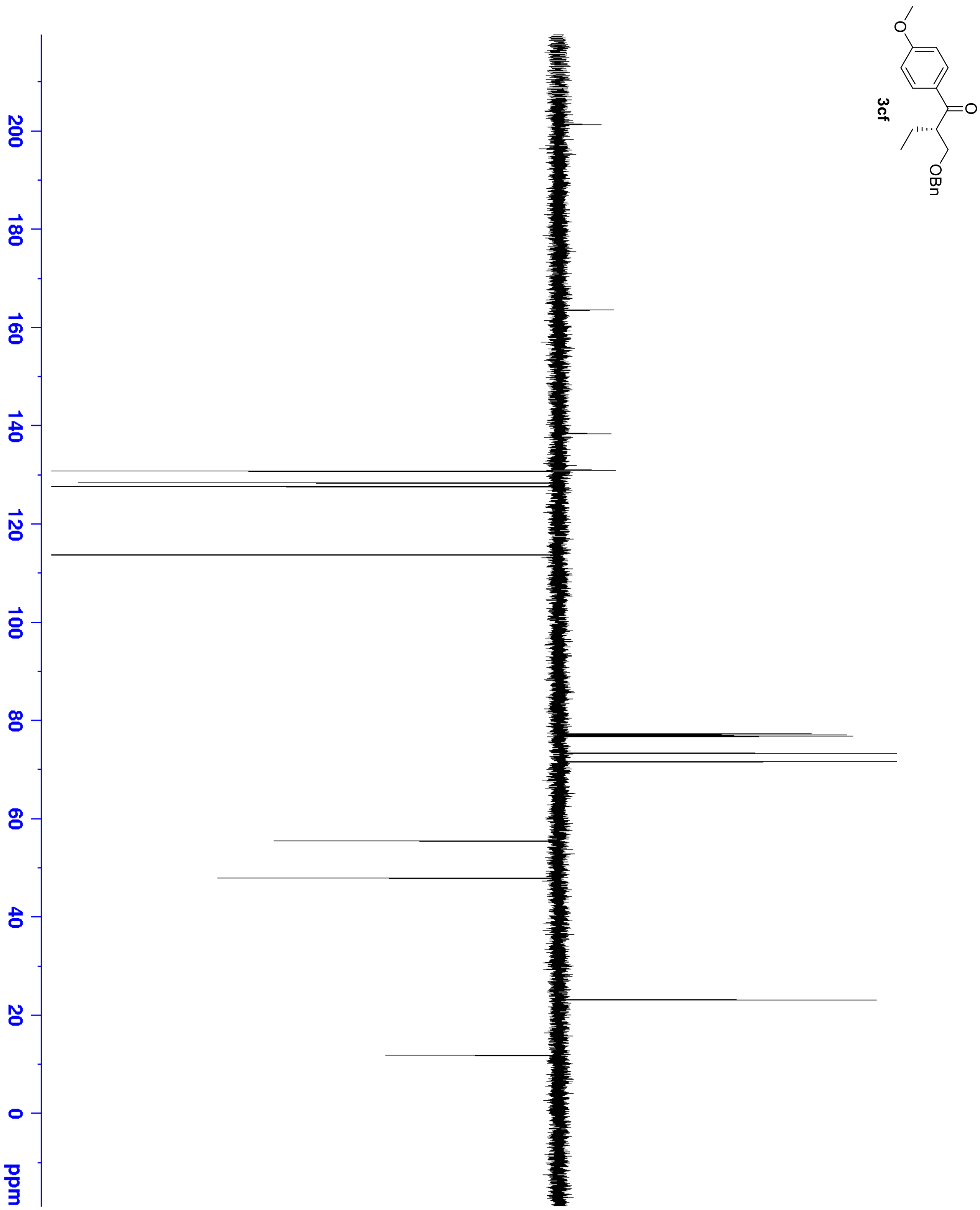
3cf
OBn
O
200
180
160
140
120
100
80
60
40
20
0
ppm

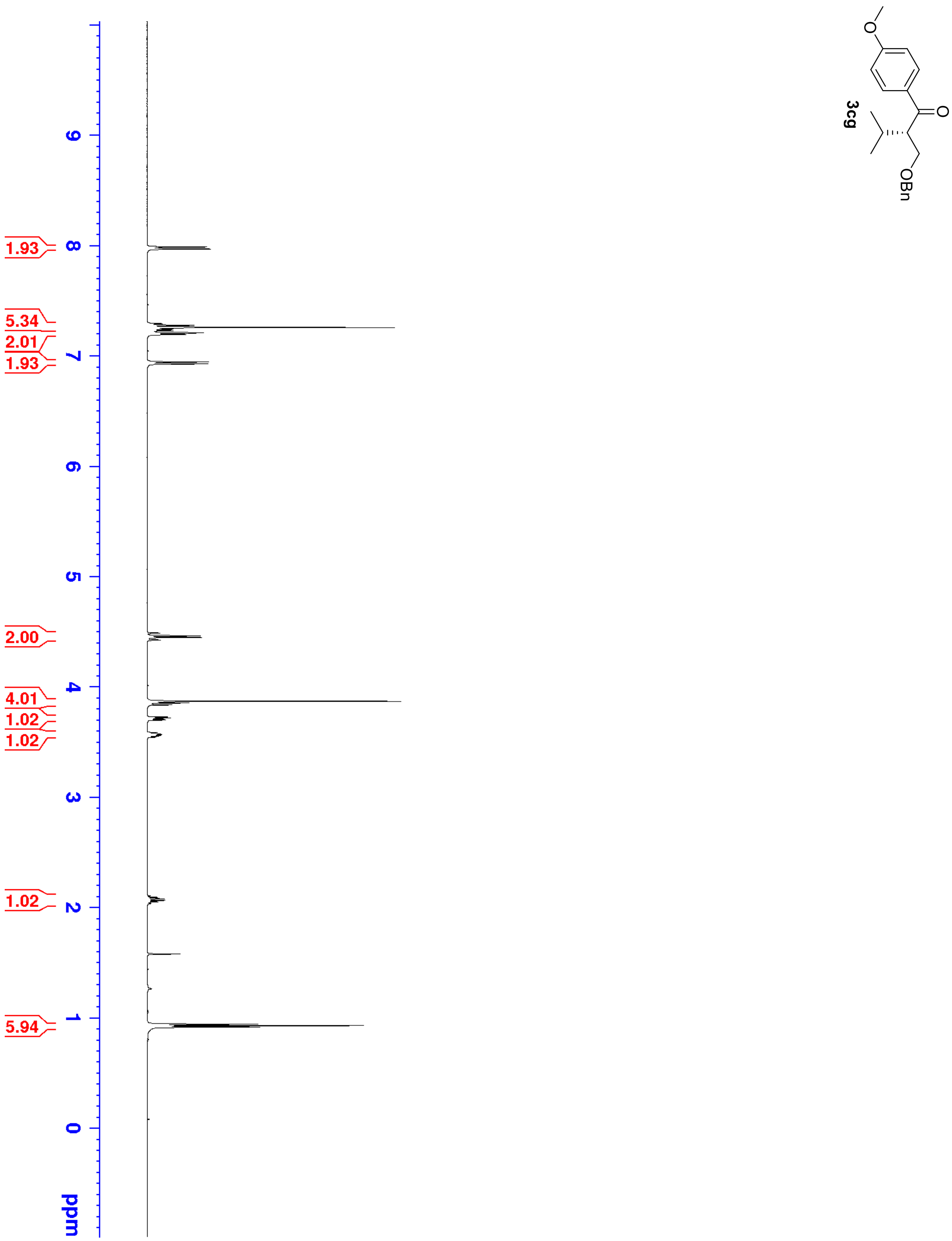
3cg
OBn
O
1.93
5.34
2.01
1.93
2.00
4.01
1.02
1.02
1.02
5.94
9
8
7
6
5
4
3
2
1
0
ppm

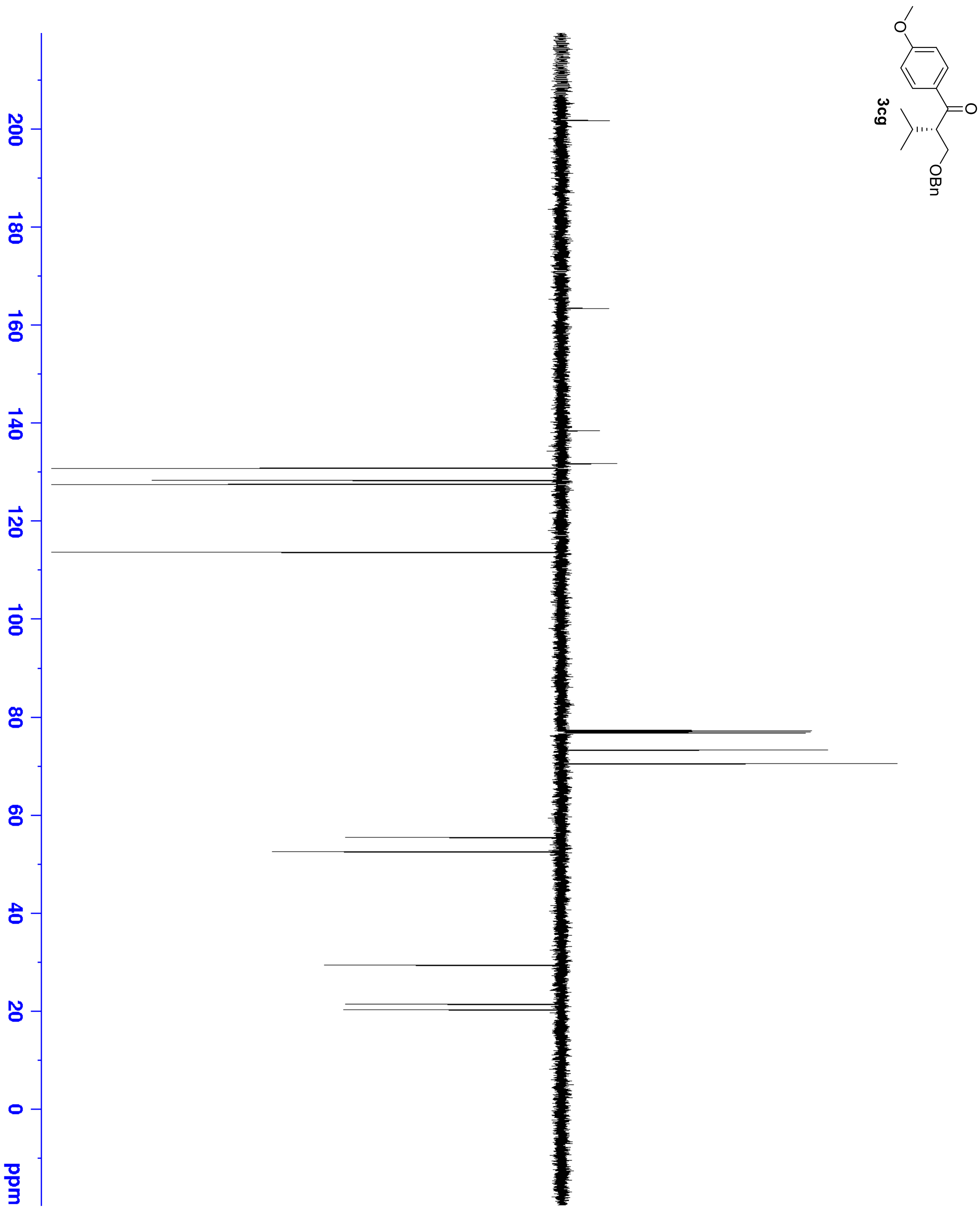
3cg
OBn
O
200
180
160
140
120
100
80
60
40
20
0
ppm

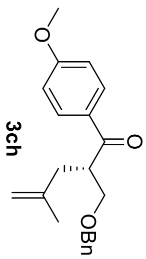
3ch
O
OBn

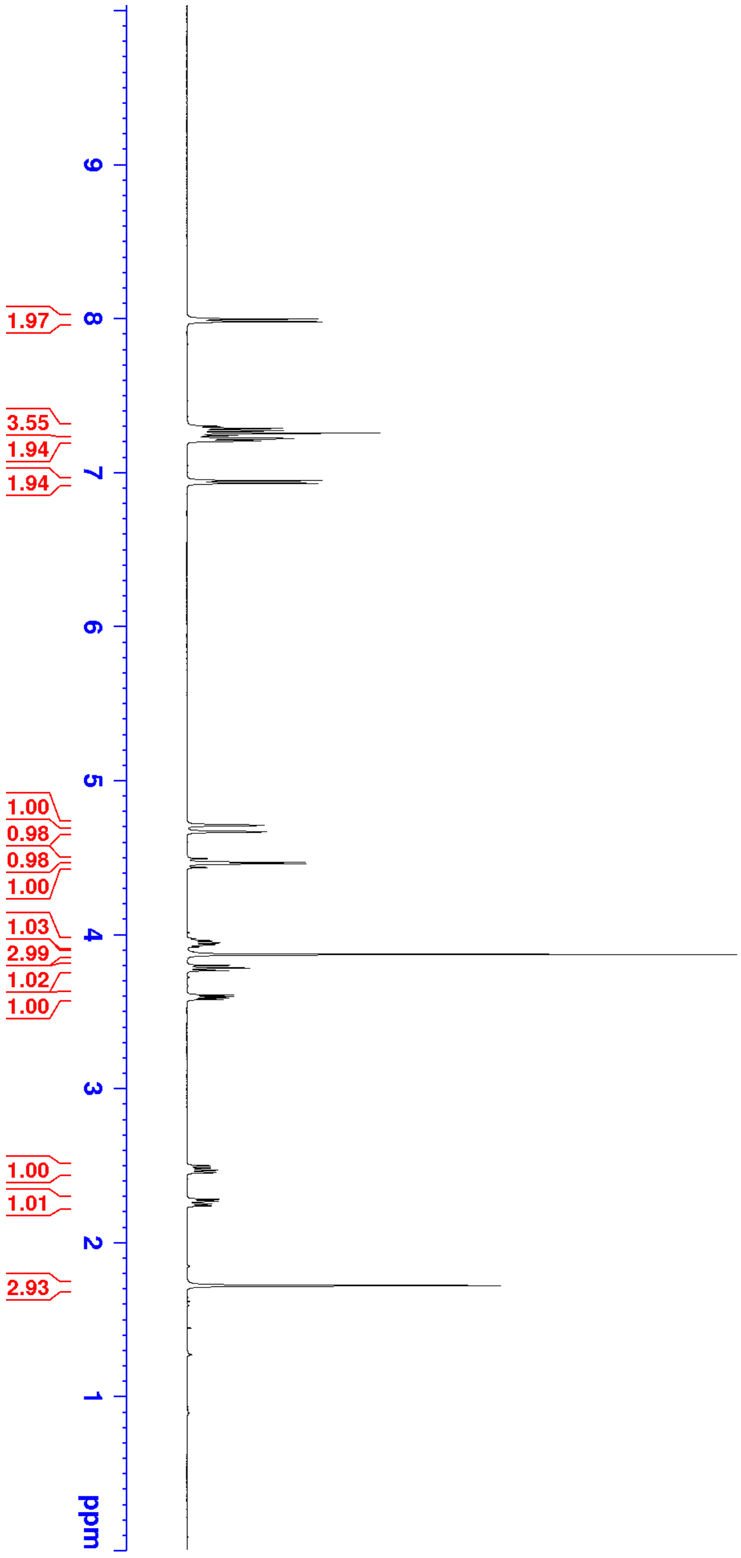
9
8
7
6
5
4
3
2
1
ppm
1.97
3.55
1.94
1.94
1.00
0.98
0.98
1.00
1.03
2.99
1.02
1.00
1.00
1.01
2.93

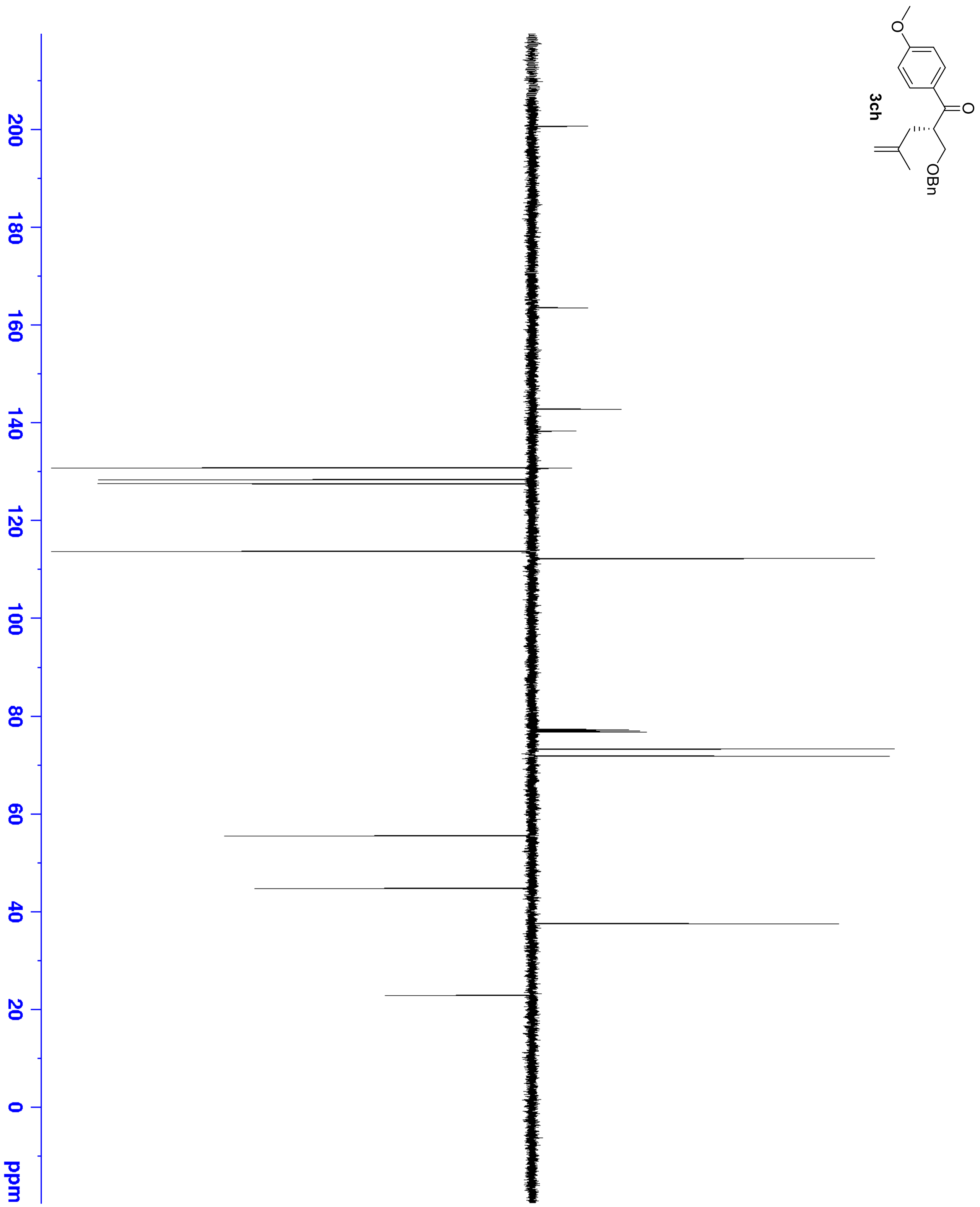

3ch
O
OBn
200
180
160
140
120
100
80
60
40
20
0
ppm

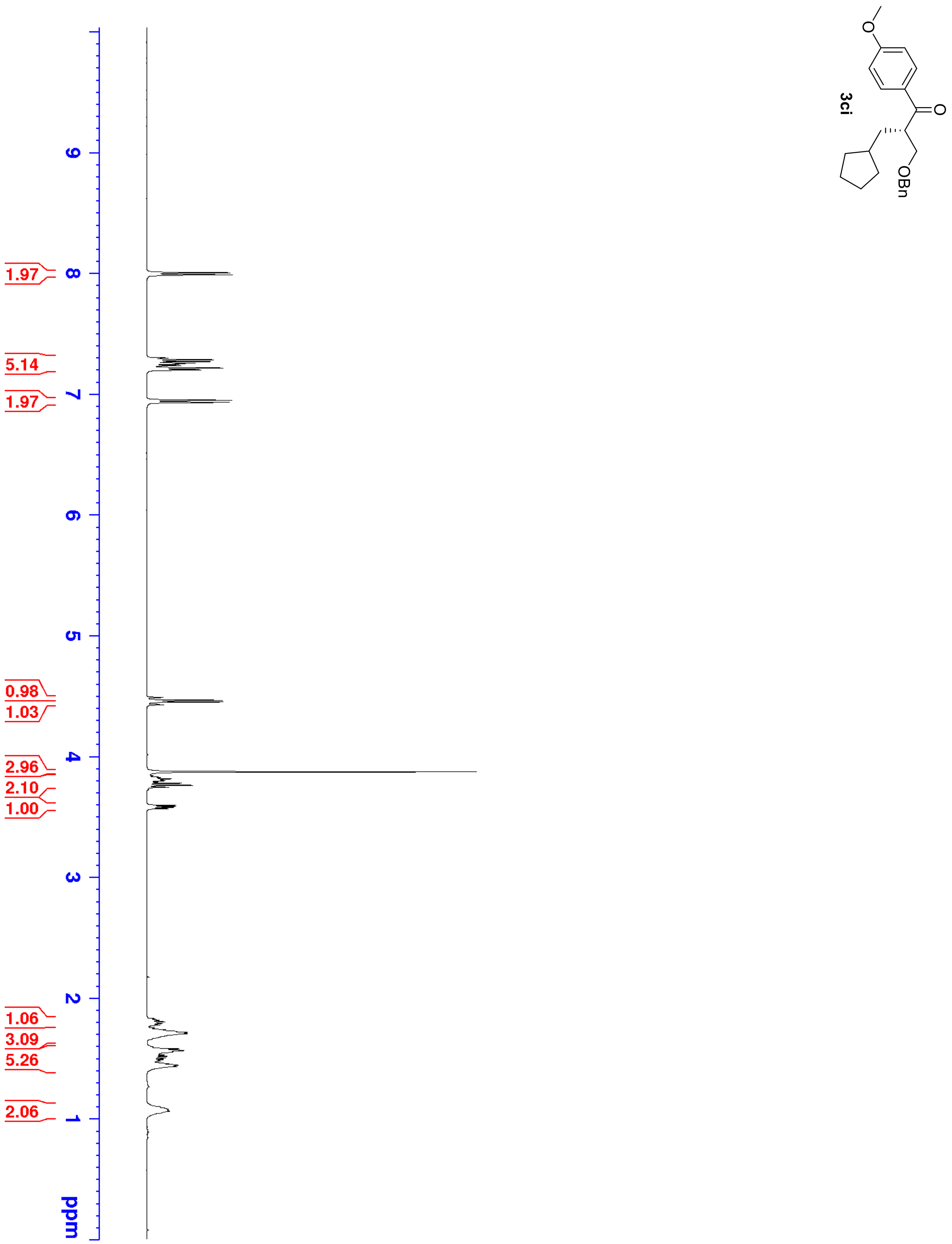
3ci
OBn
O
1.97
5.14
1.97
0.98
1.03
2.96
2.10
1.00
1.06
3.09
5.26
2.06
9
8
7
6
5
4
3
2
1
ppm

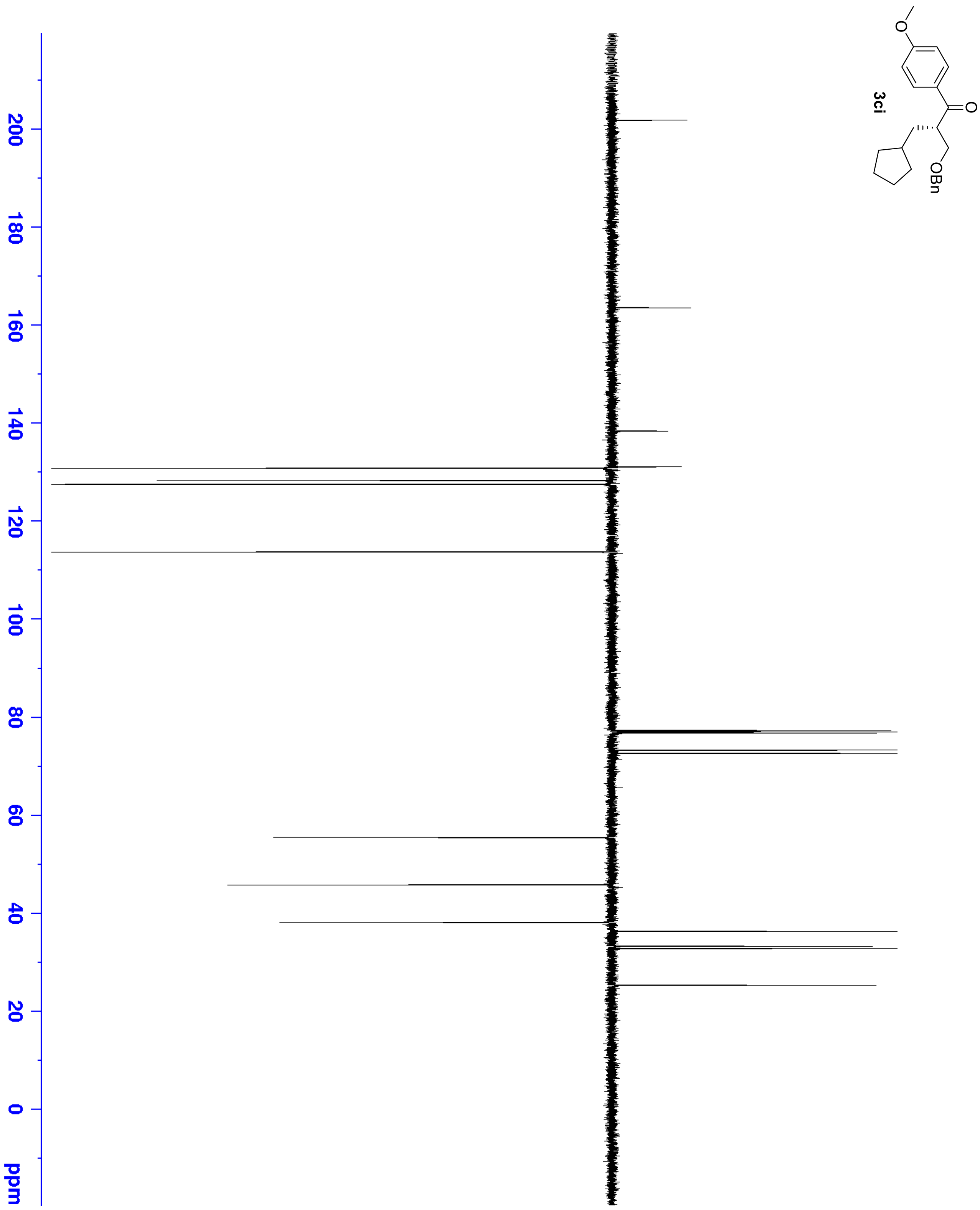
3ci
O
OBn
200
180
160
140
120
100
80
60
40
20
0
ppm

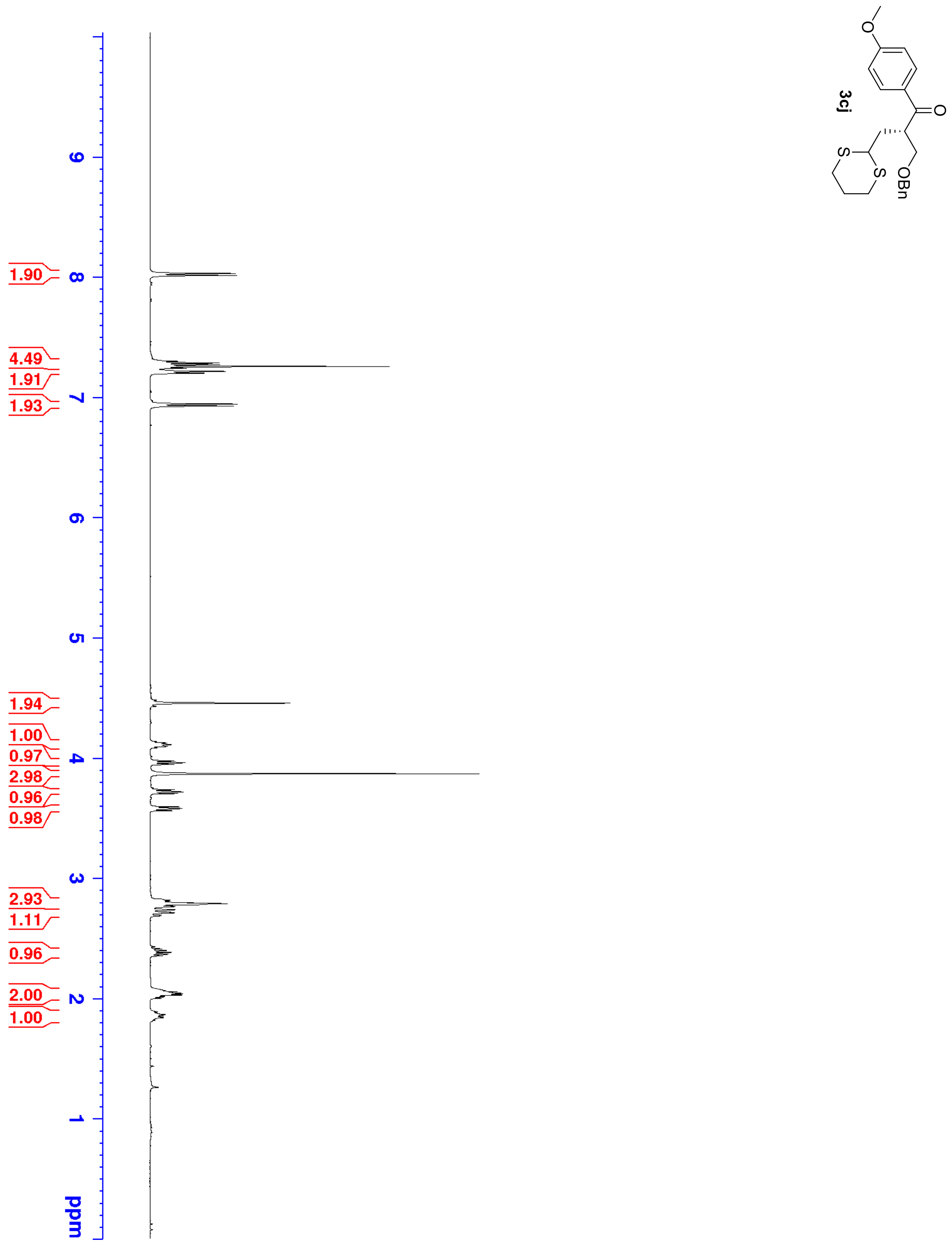
3cj
O
OBn
S
S
1.90
4.49
1.91
1.93
1.94
1.00
0.97
2.98
0.96
0.98
2.93
1.11
0.96
2.00
1.00
9
8
7
6
5
4
3
2
1
ppm

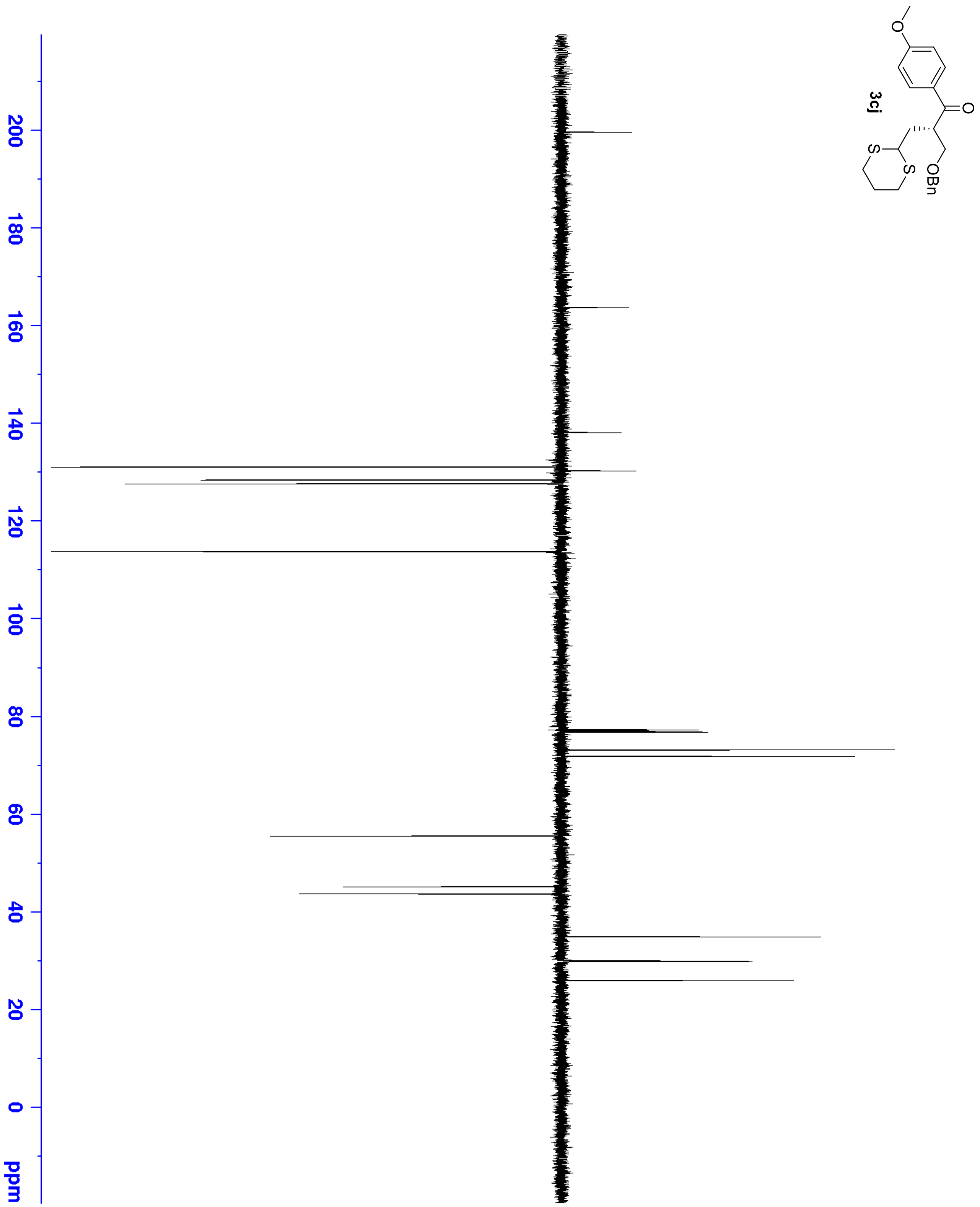
3cj
OBn
O
S
S
200
180
160
140
120
100
80
60
40
20
0
ppm

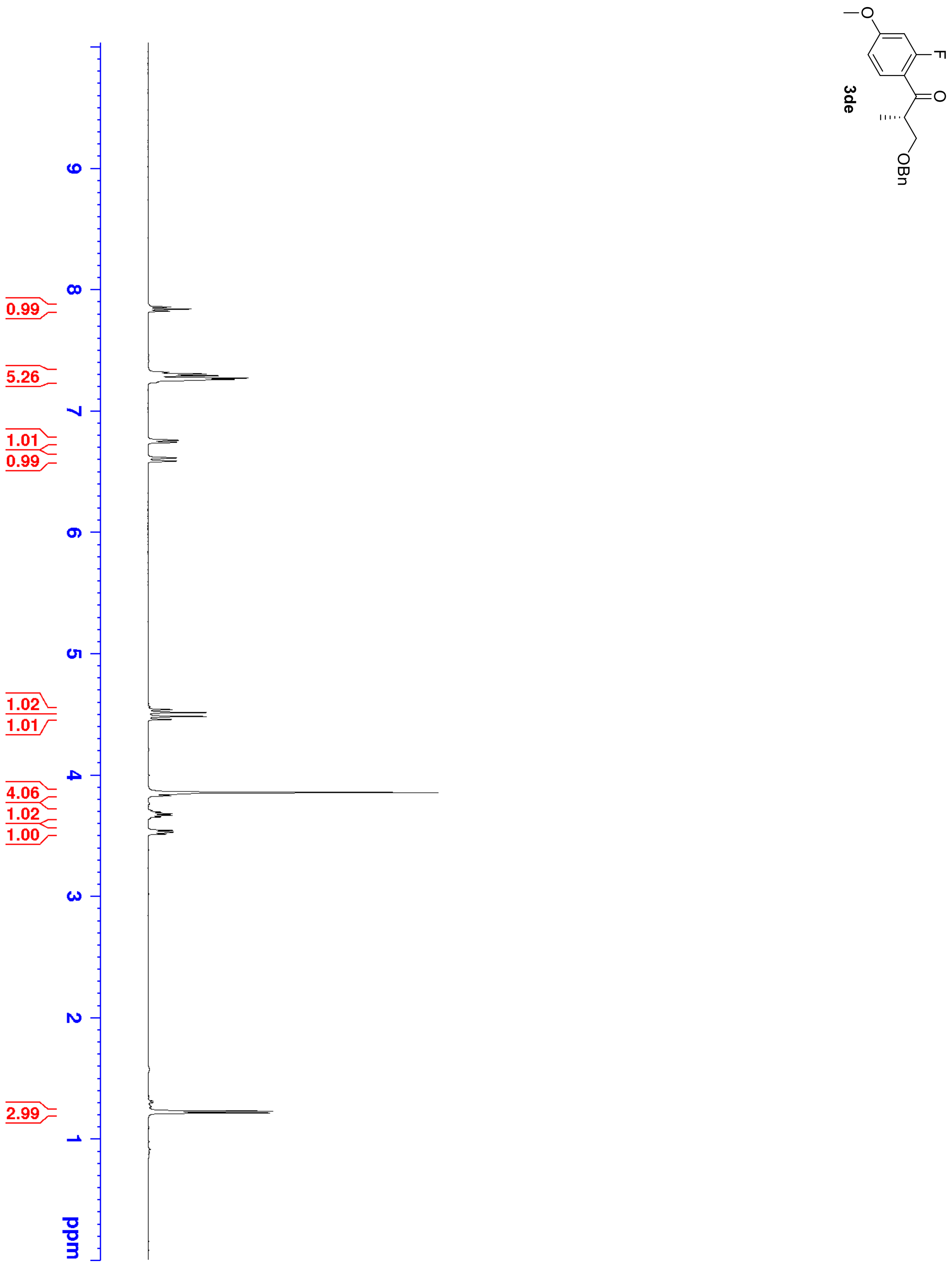
3de
OBn
F
O
ppm
9
8
7
6
5
4
3
2
1
0.99
5.26
1.01
0.99
1.02
1.01
4.06
1.02
1.00
2.99

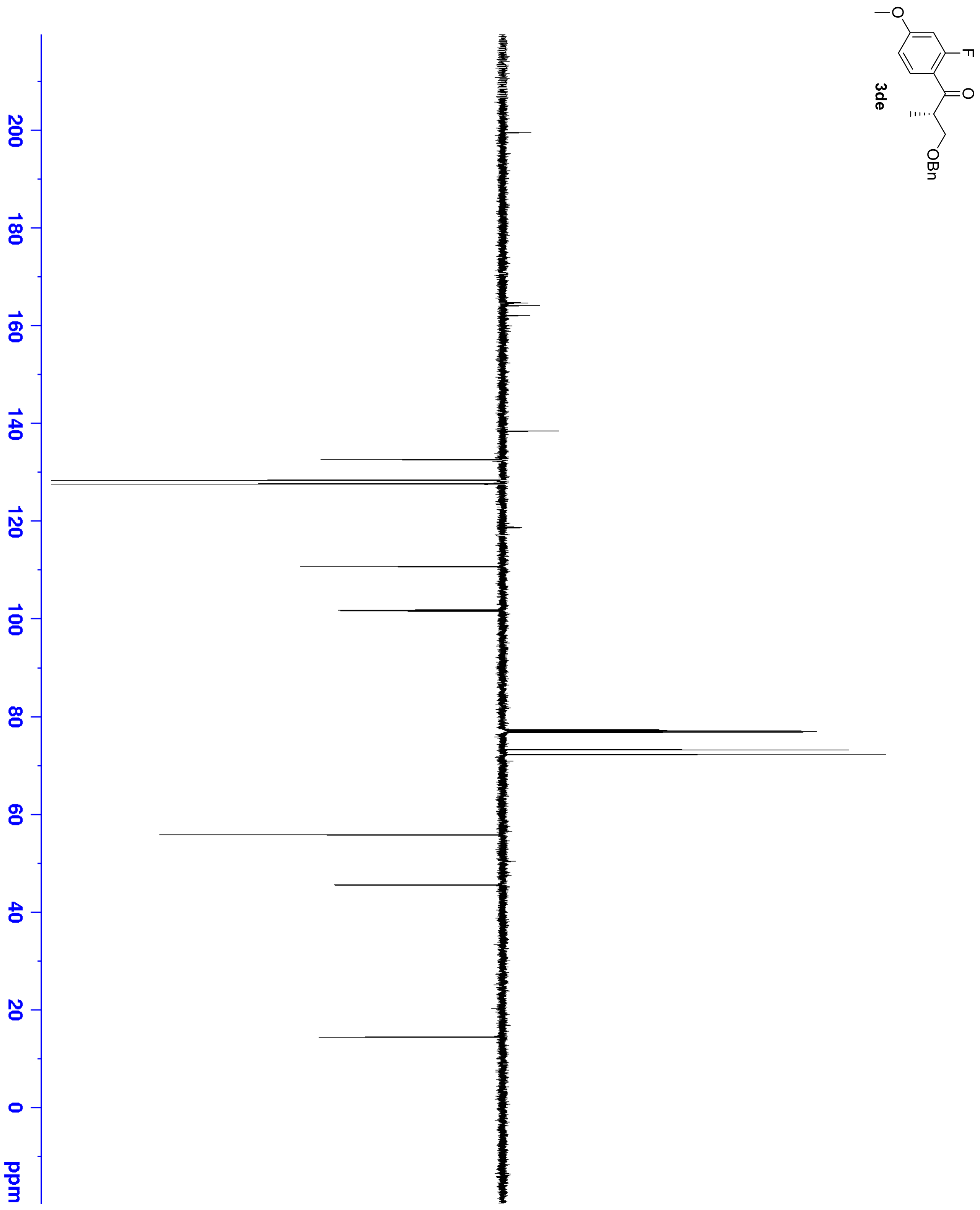
3de
OBn
F
O
200
180
160
140
120
100
80
60
40
20
0
ppm

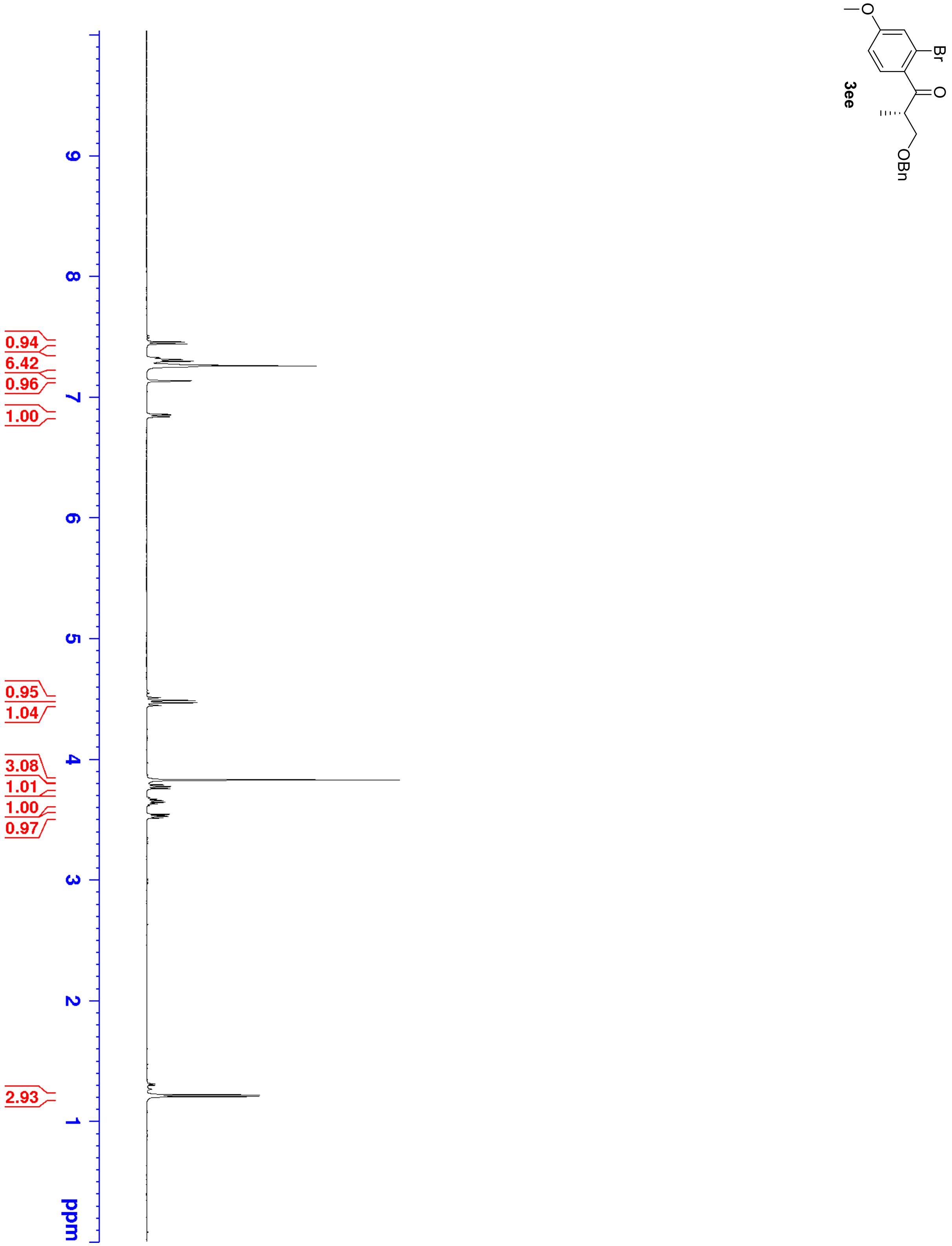

0.94
6.42
0.96
1.00
0.95
1.04
3.08
1.01
1.00
0.97
2.93
9
8
7
6
5
4
3
2
1
ppm
Br
O
OBn
3ee

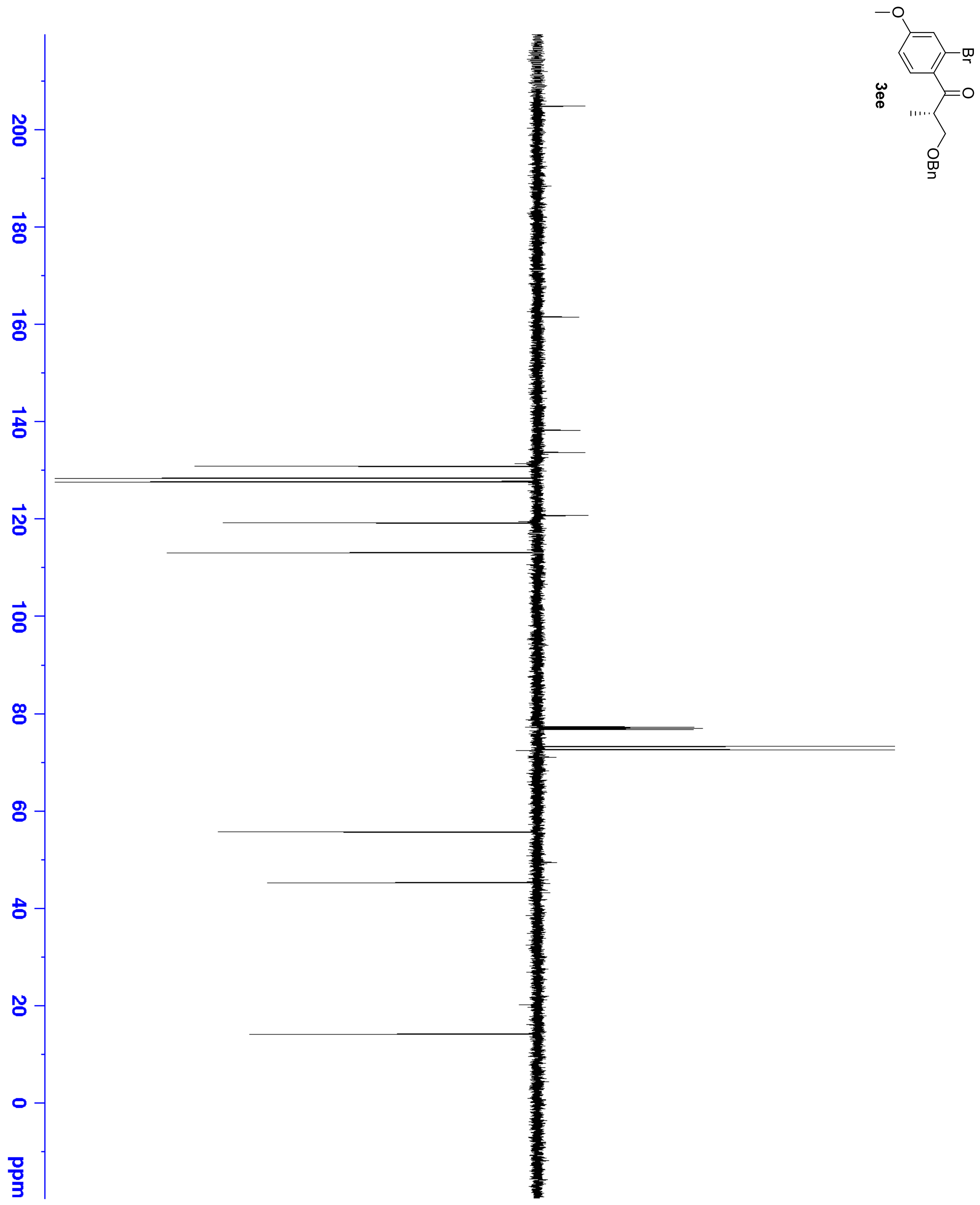

Br
O
OBn
3ee
200
180
160
140
120
100
80
60
40
20
0
ppm

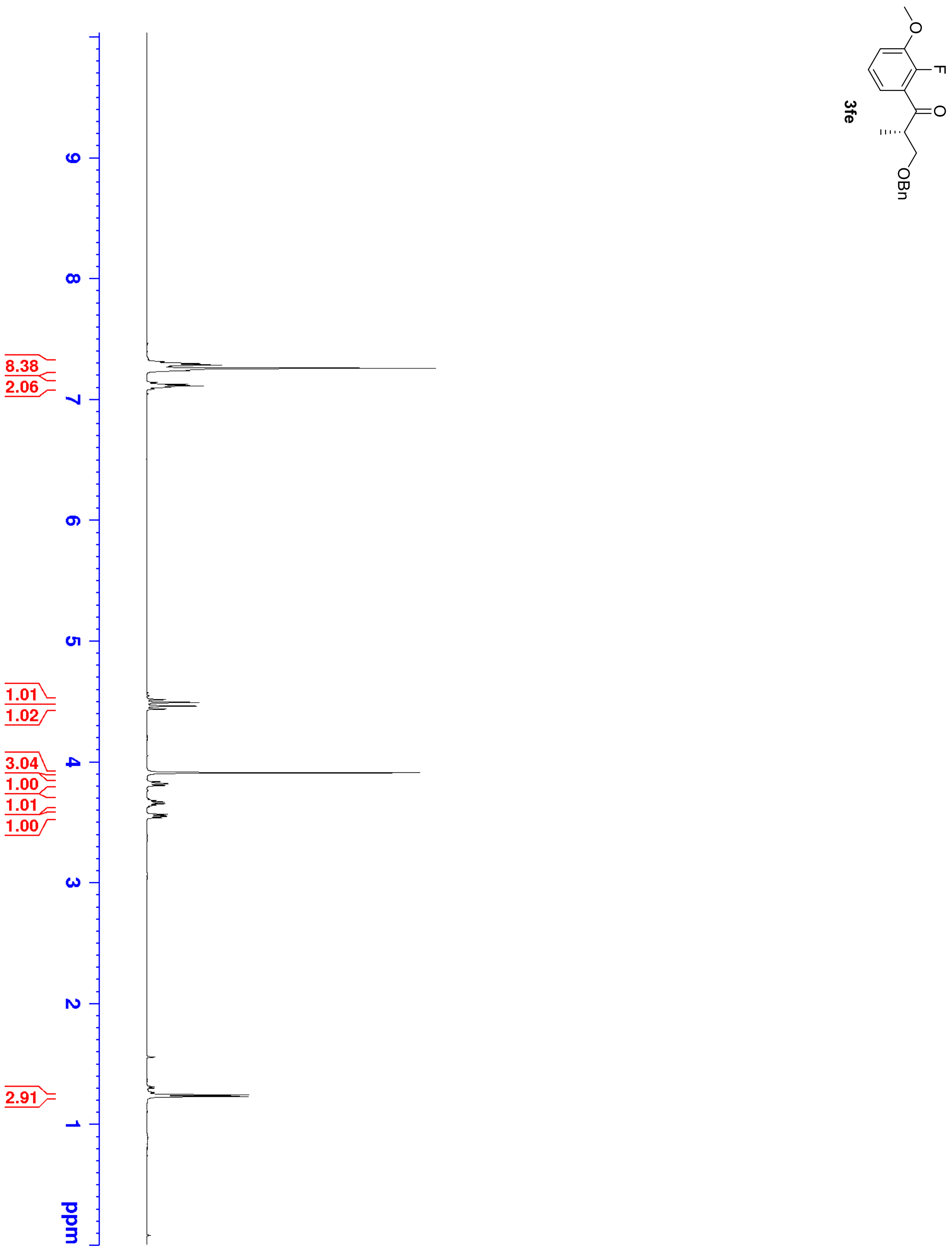

3fe
F
O
OBn
9
8
7
6
5
4
3
2
1
ppm
8.38
2.06
1.01
1.02
3.04
1.00
1.01
1.00
2.91

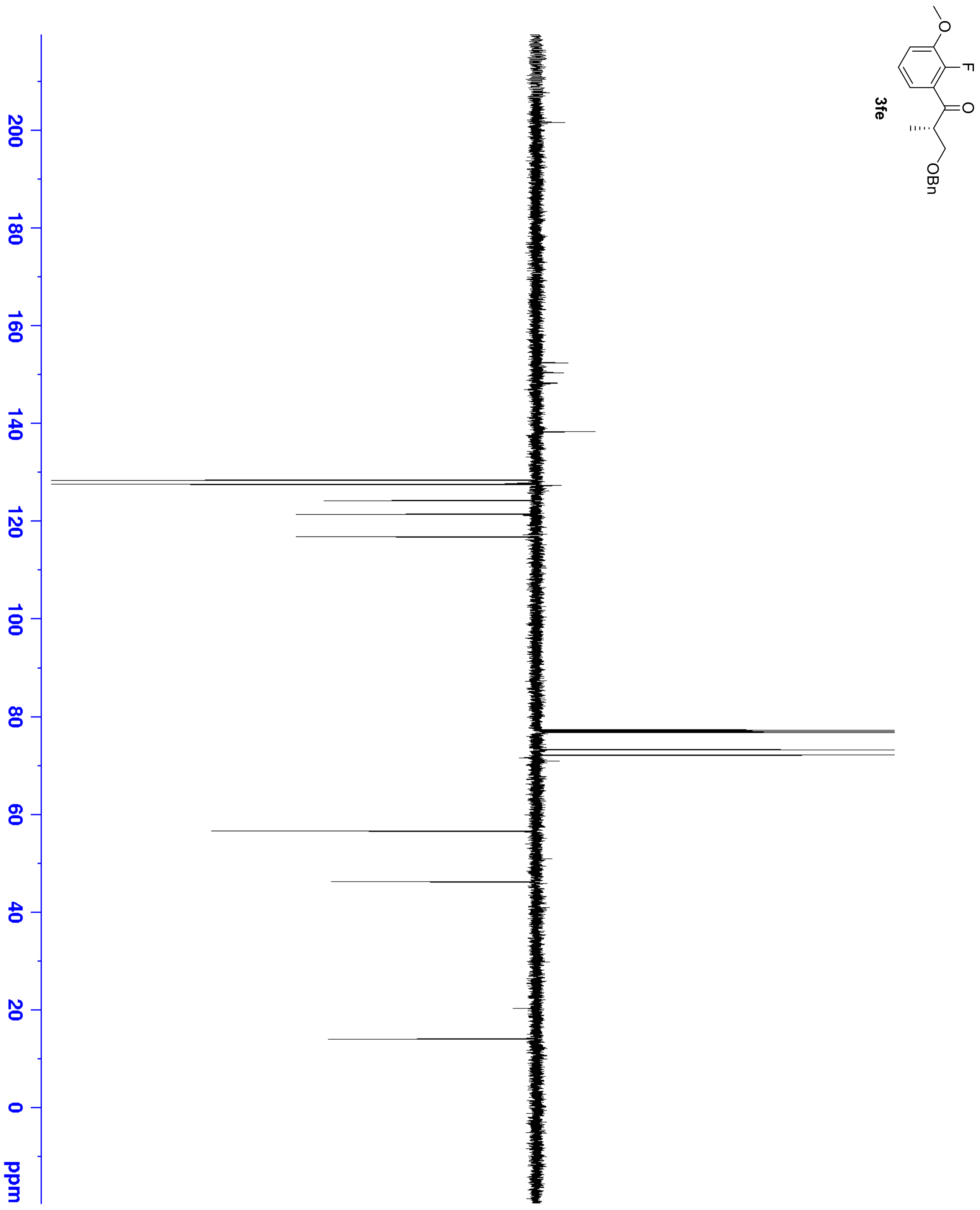

F
O
OBn
3fe
200
180
160
140
120
100
80
60
40
20
0
ppm

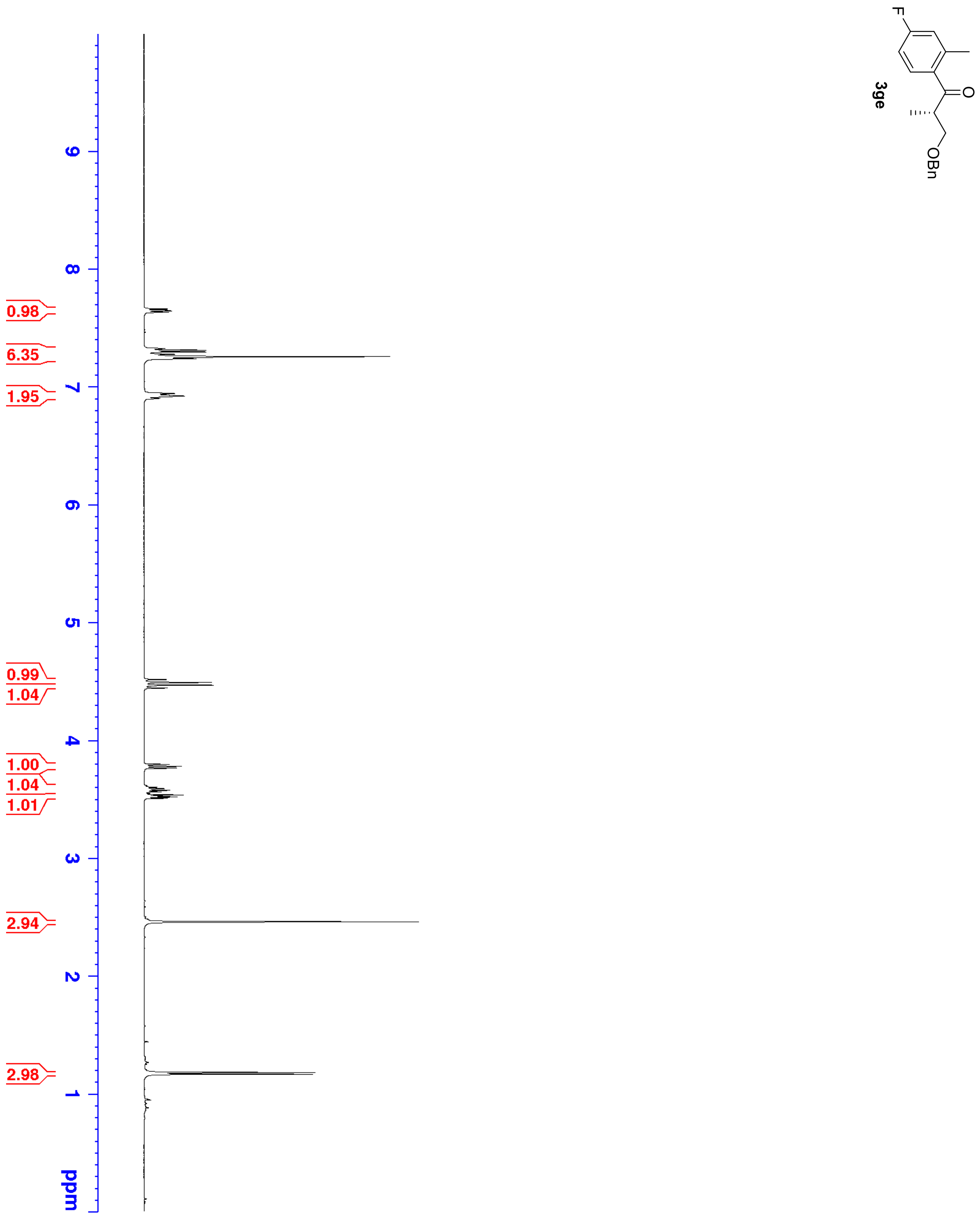
3ge
F
O
OBn
0.98
6.35
1.95
0.99
1.04
1.00
1.04
1.01
2.94
2.98
9
8
7
6
5
4
3
2
1
ppm

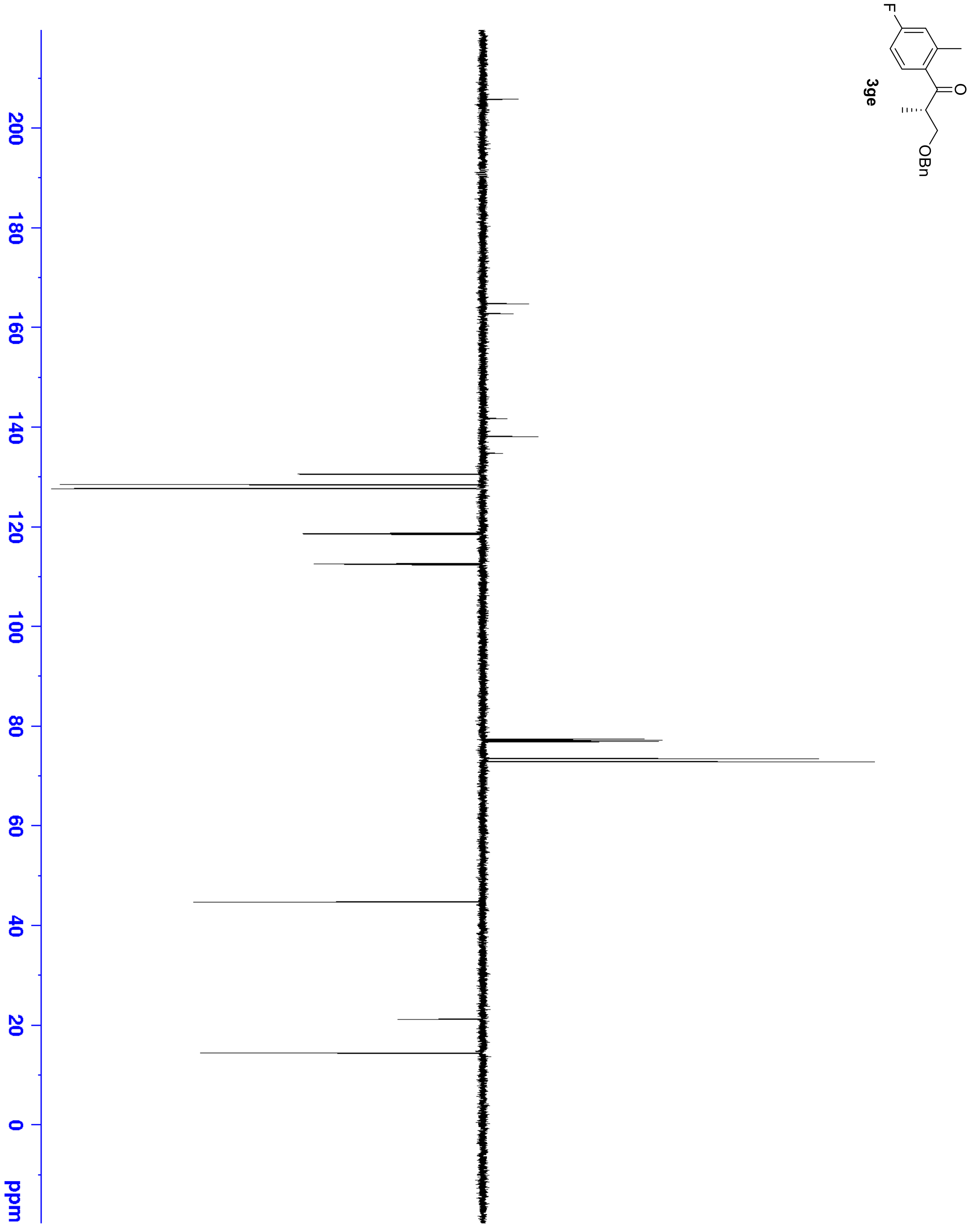

F
O
OBn
3ge
200
180
160
140
120
100
80
60
40
20
0
ppm

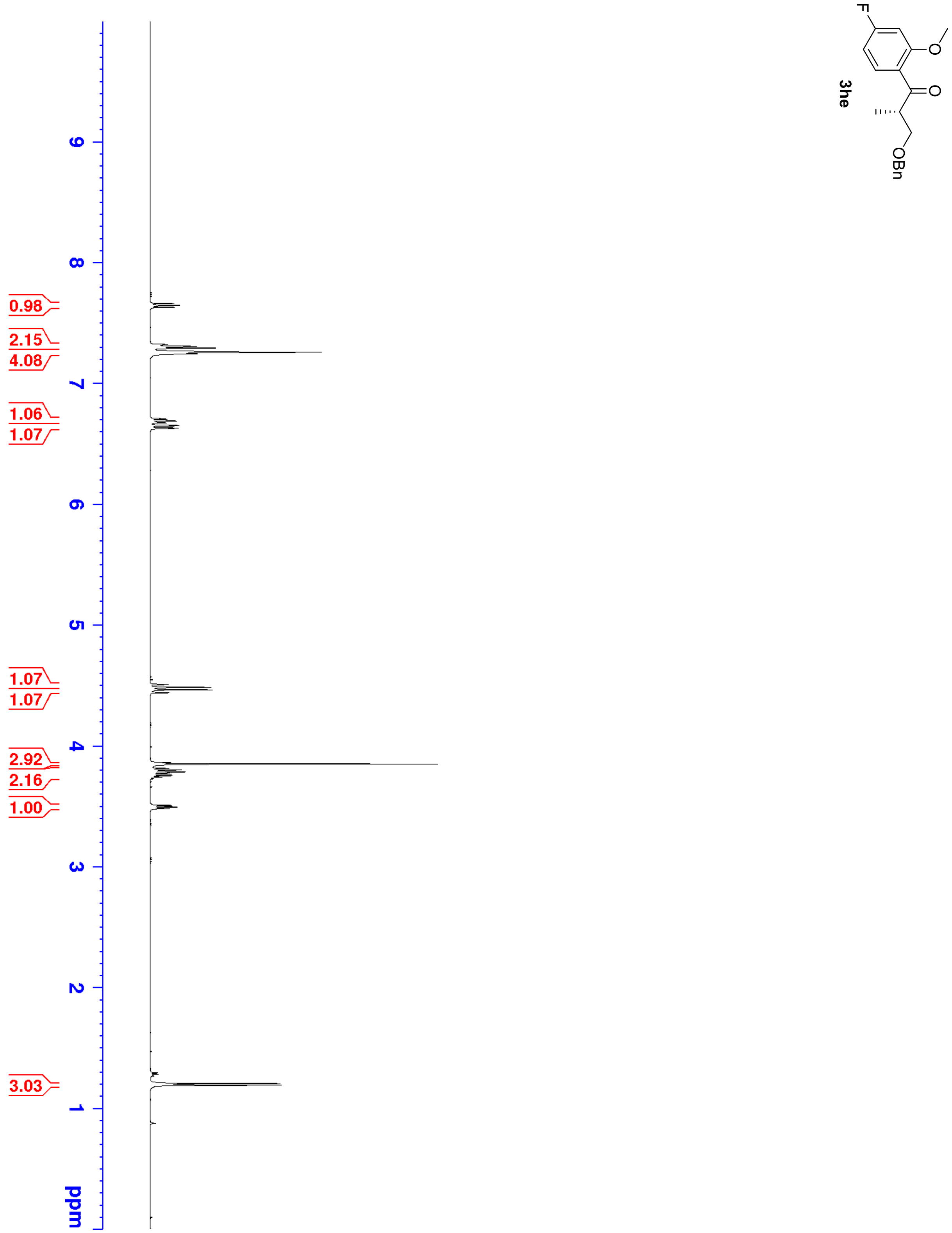
3he
OBn
F
O
0.98
2.15
4.08
1.06
1.07
1.07
1.07
2.92
2.16
1.00
3.03
9
8
7
6
5
4
3
2
1
ppm

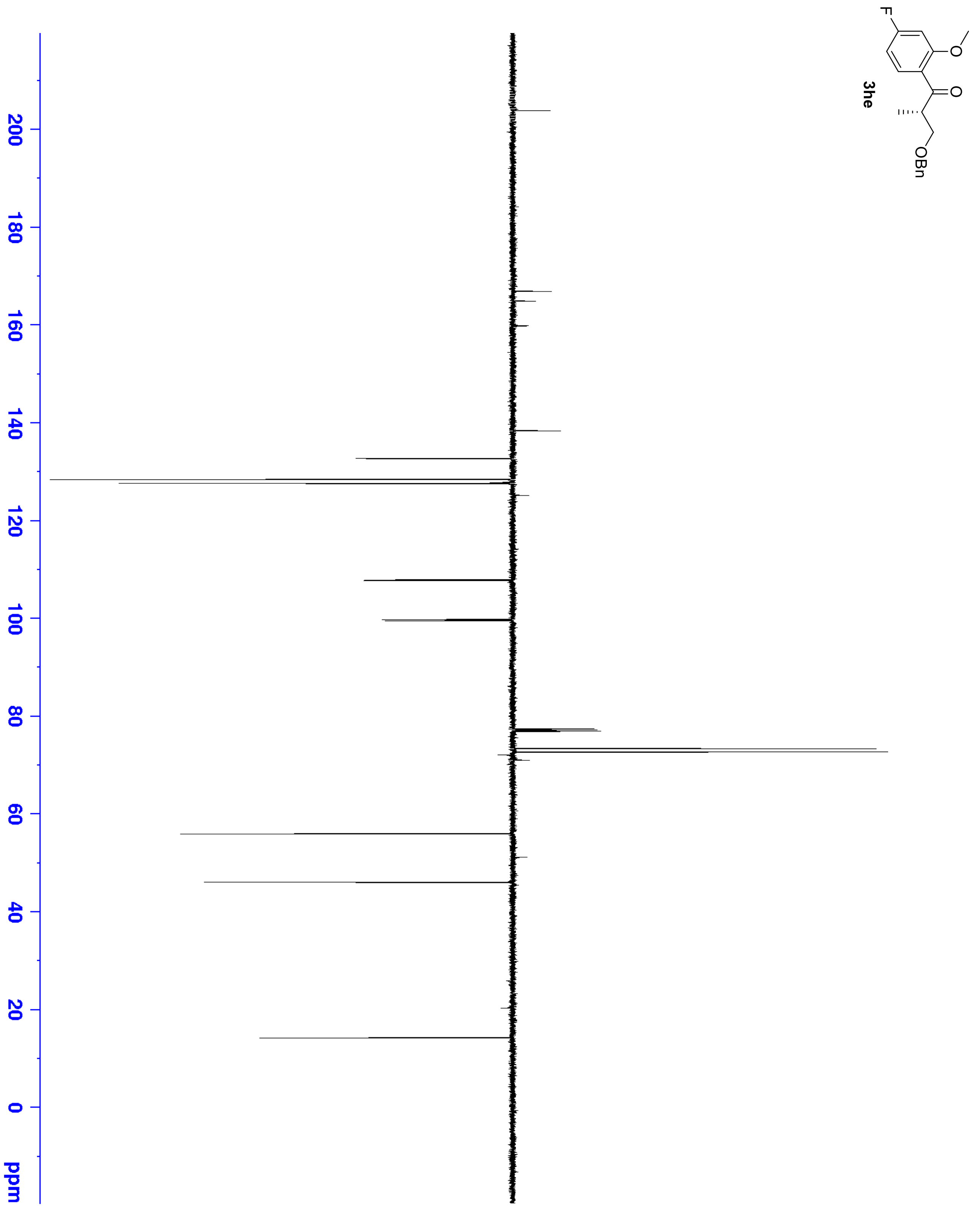
3he
OBn
200
180
160
140
120
100
80
60
40
20
0
ppm

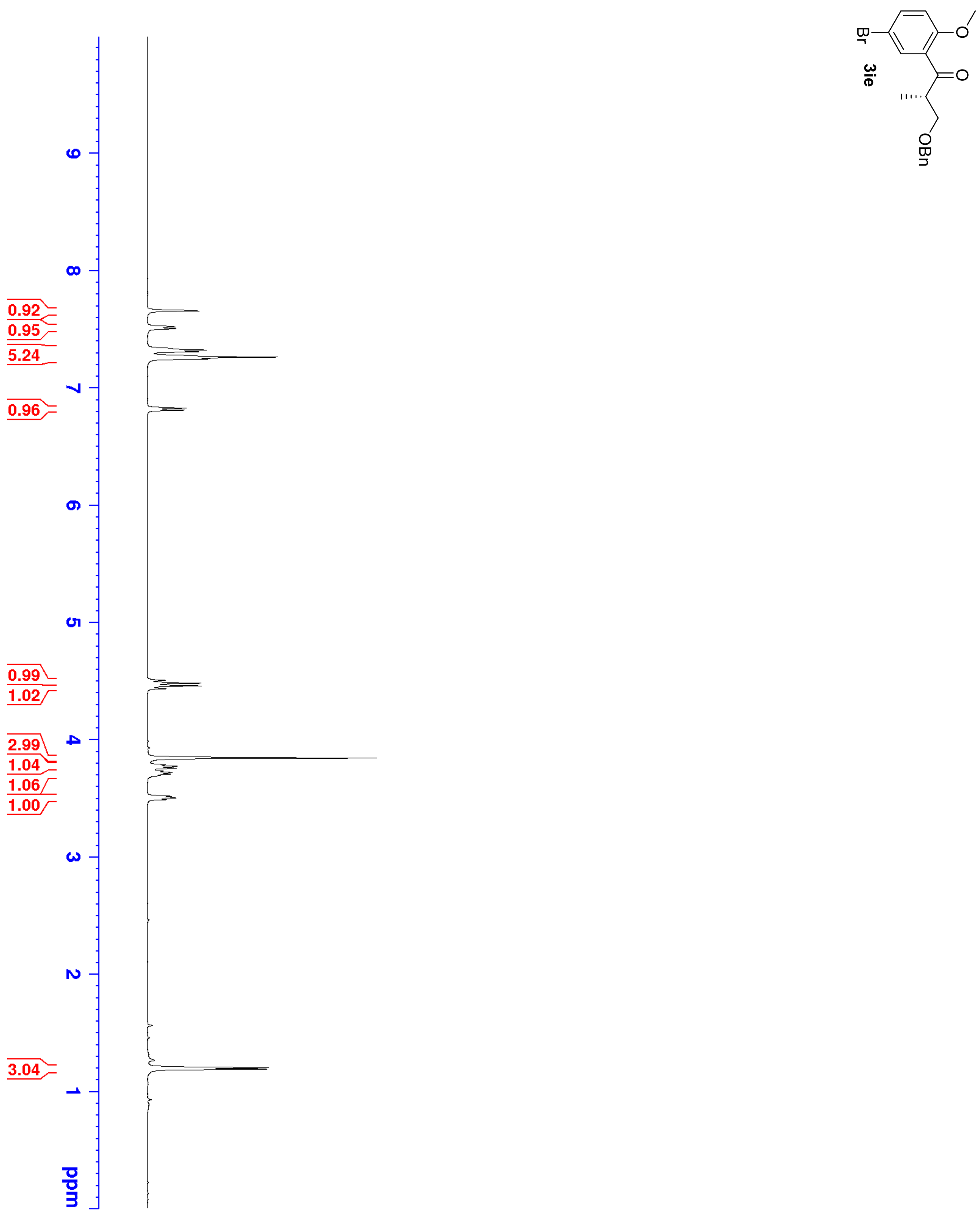
3ie
Br
O
OBn
0.92
0.95
5.24
0.96
0.99
1.02
2.99
1.04
1.06
1.00
3.04
9
8
7
6
5
4
3
2
1
ppm

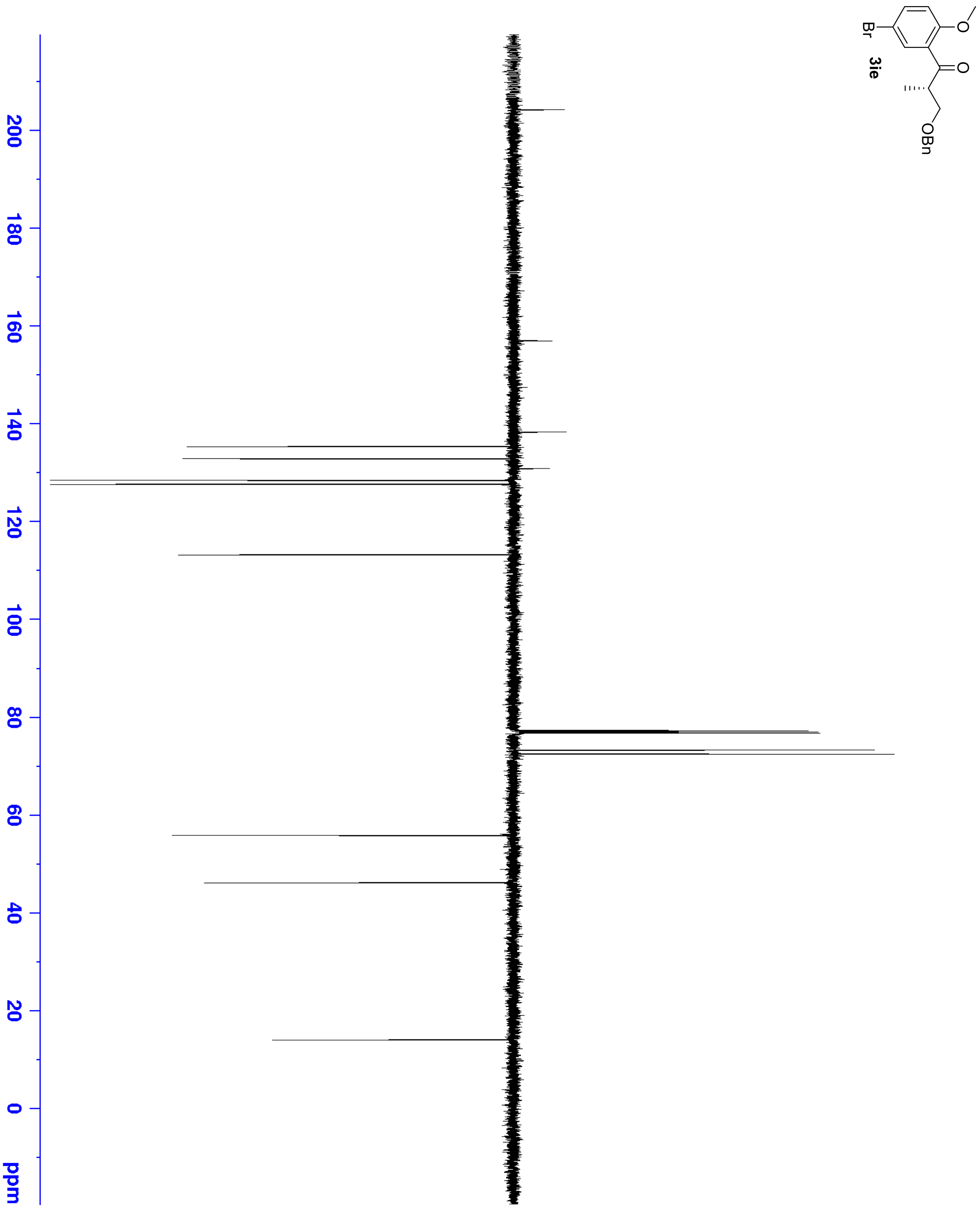

Br
O
O
OBn
3ie
200
180
160
140
120
100
80
60
40
20
0
ppm

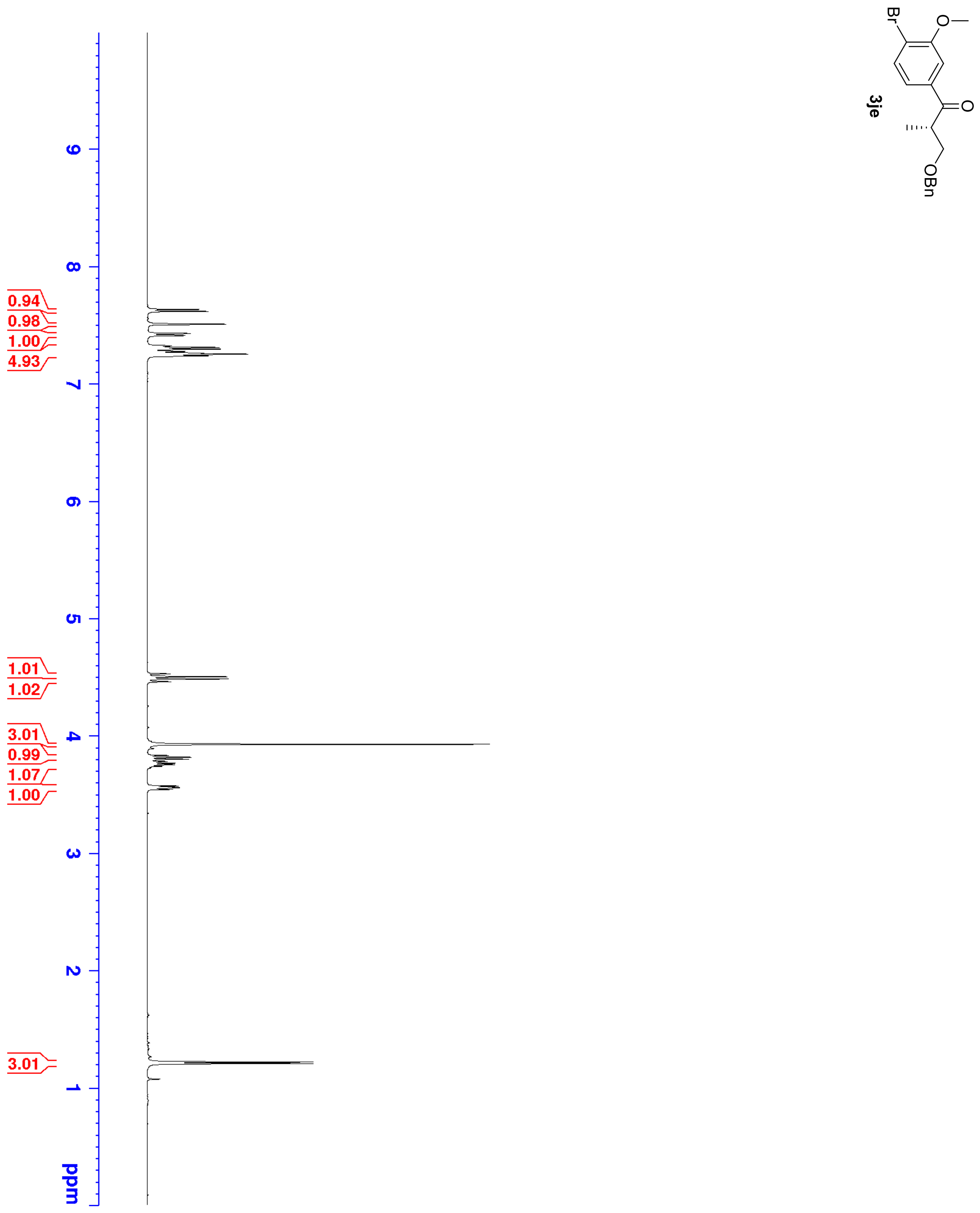
3je
Br
OBn
0.94
0.98
1.00
4.93
1.01
1.02
3.01
0.99
1.07
1.00
3.01
9
8
7
6
5
4
3
2
1
ppm

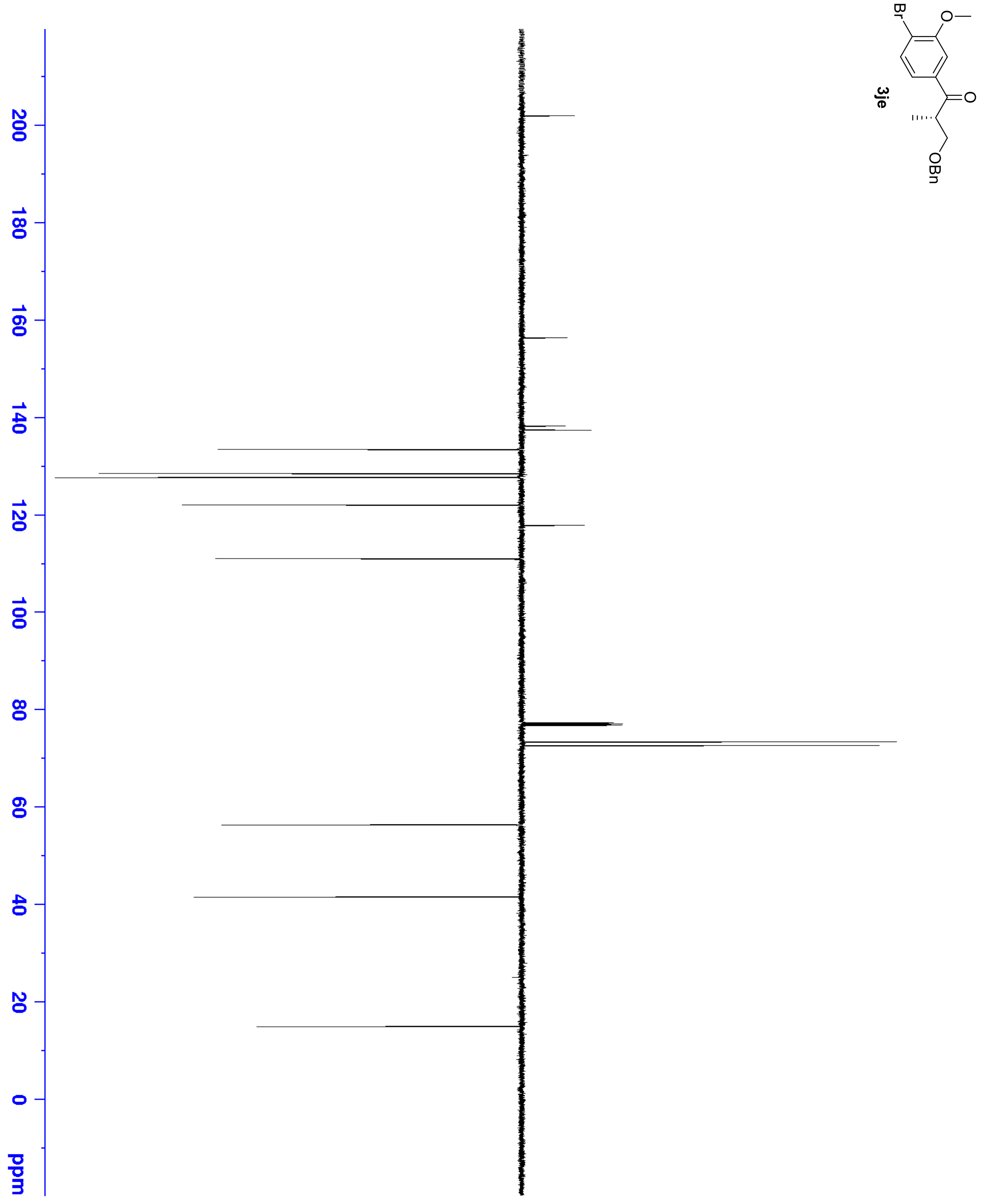
3je
Br
OBn
200
180
160
140
120
100
80
60
40
20
0
ppm

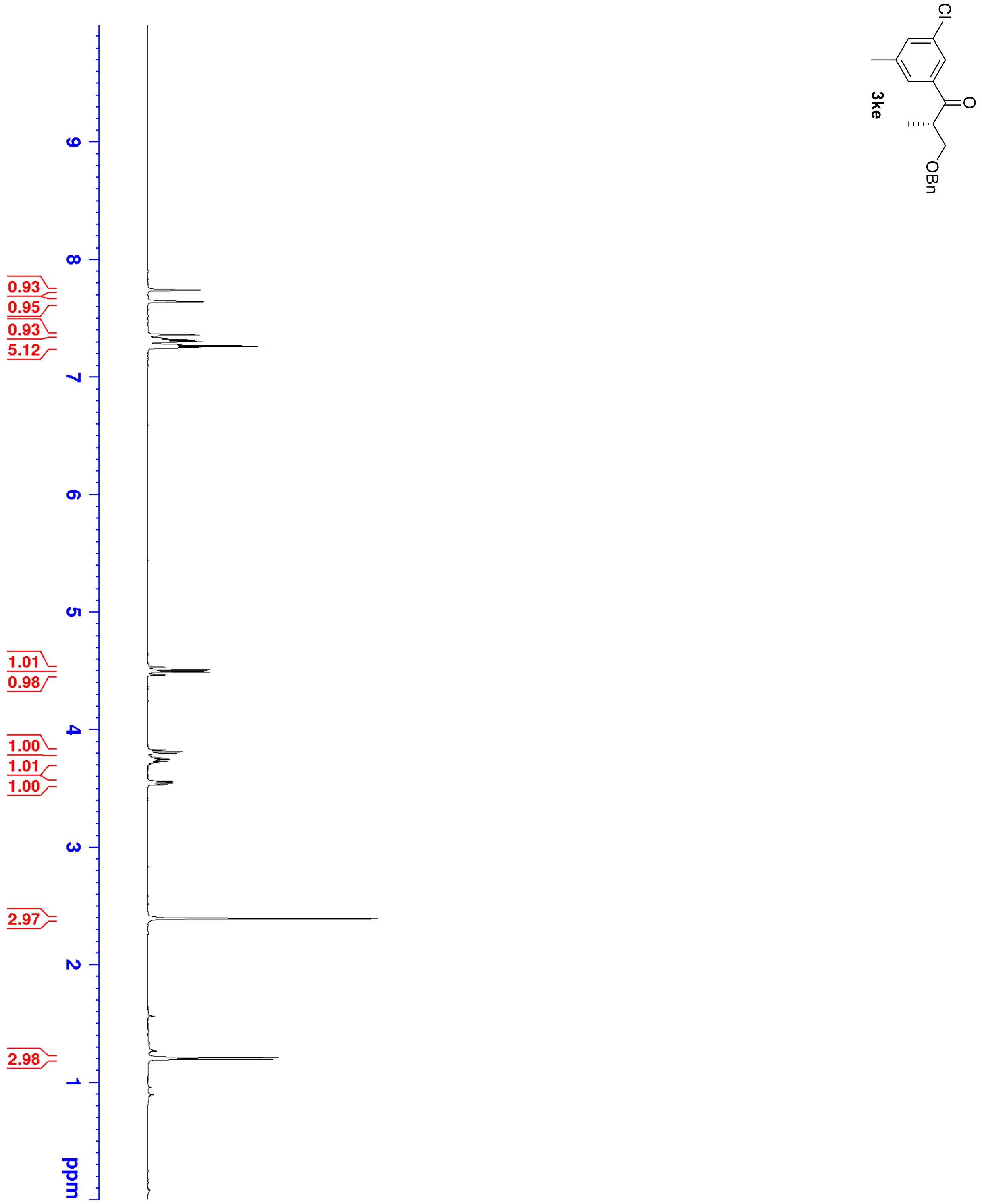
Cl
O
OBn
3ke
0.93
0.95
0.93
5.12
1.01
0.98
1.00
1.01
1.00
2.97
2.98
9
8
7
6
5
4
3
2
1
ppm

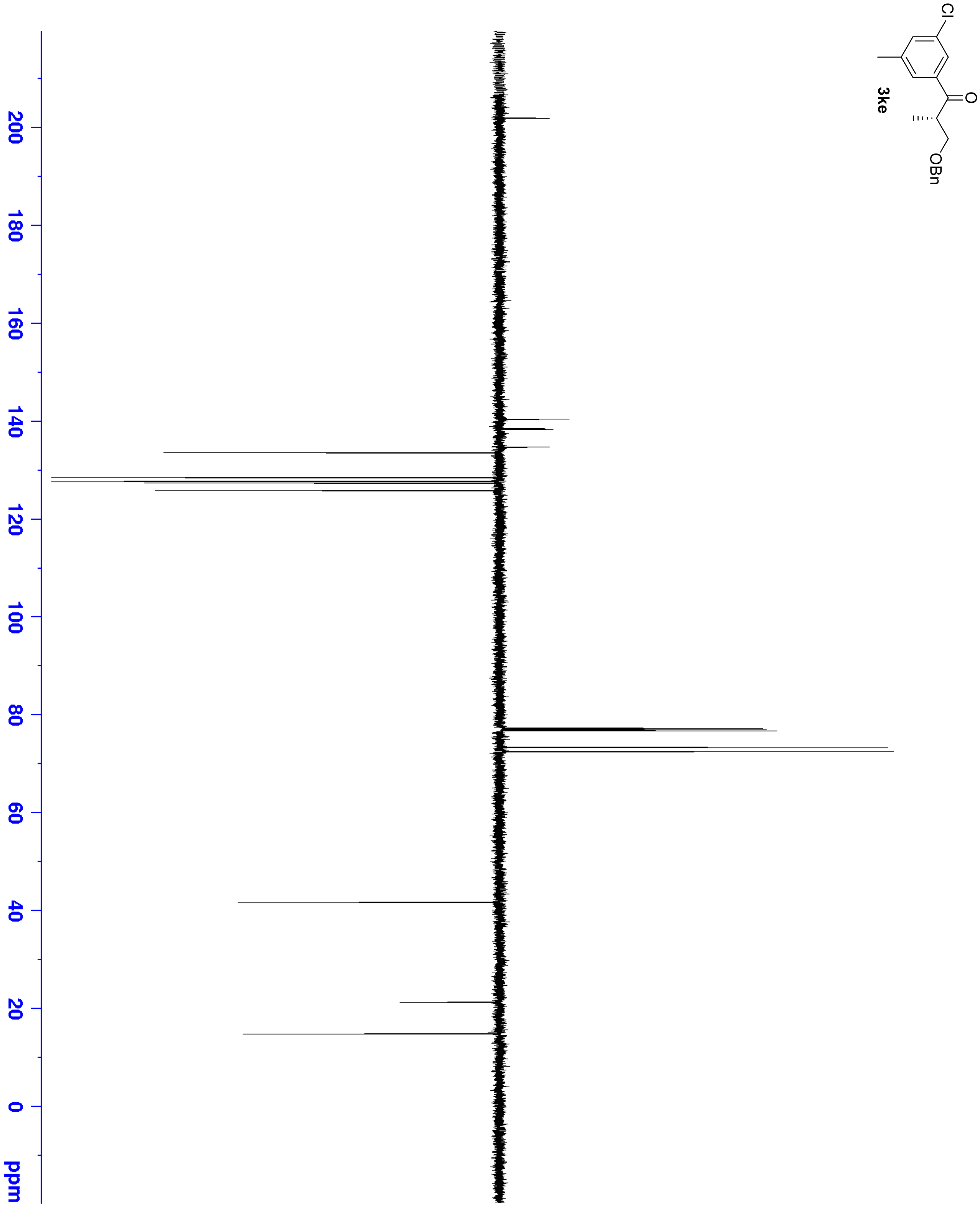
Cl
O
OBn
3ke
200
180
160
140
120
100
80
60
40
20
0
ppm

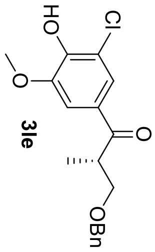

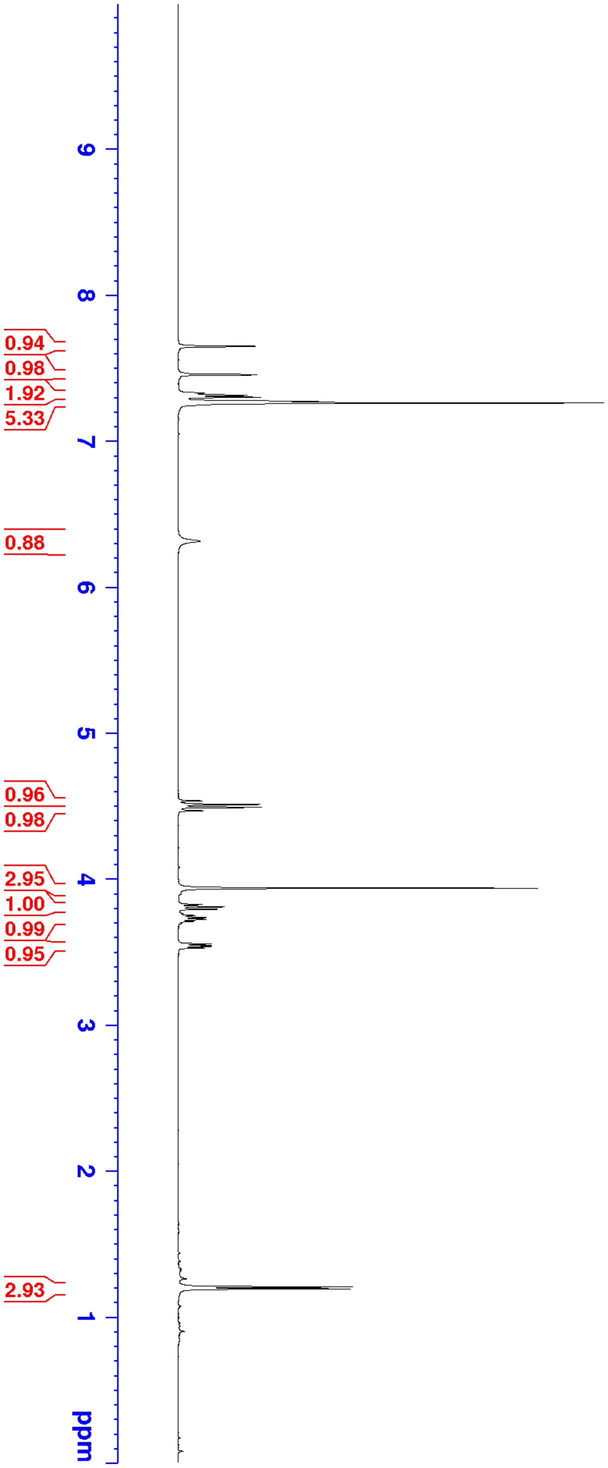

0.94
0.98
1.92
5.33
0.88
0.96
0.98
2.95
1.00
0.99
0.95
2.93
9
8
7
6
5
4
3
2
1
ppm

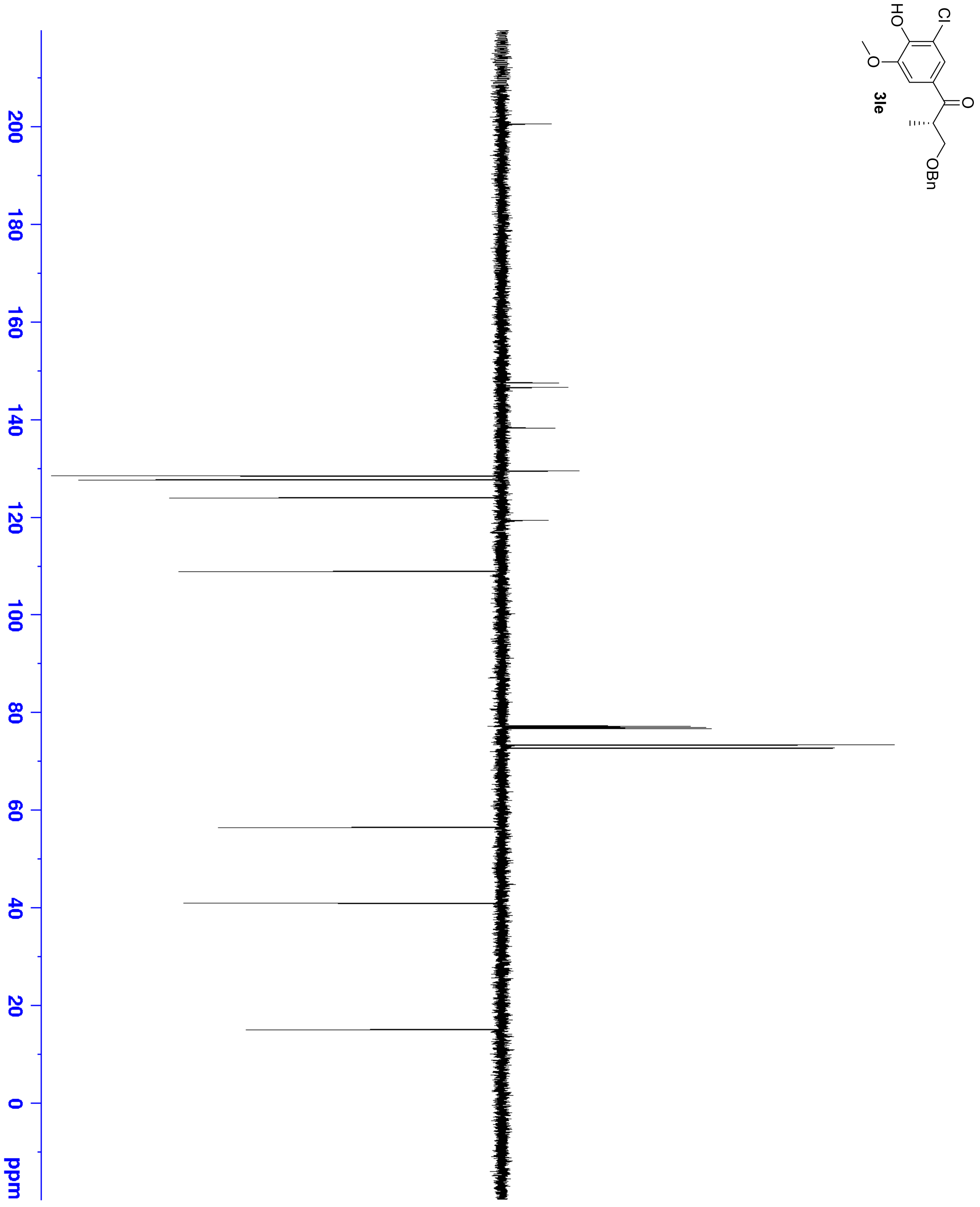
3le
HO
Cl
O
OBn
200
180
160
140
120
100
80
60
40
20
0
ppm

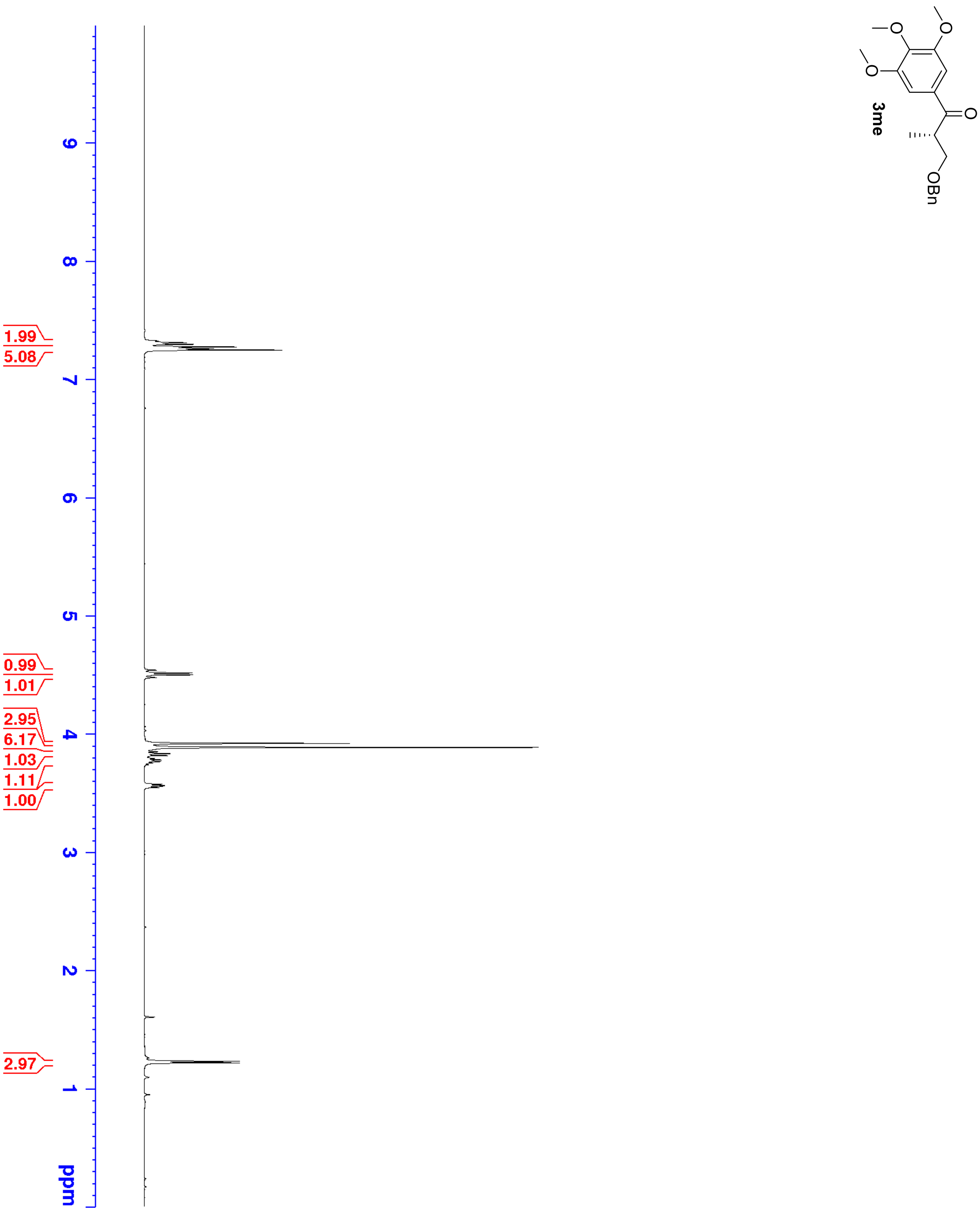
3me
OBn
O
9
8
7
6
5
4
3
2
1
ppm
1.99
5.08
0.99
1.01
2.95
6.17
1.03
1.11
1.00
2.97

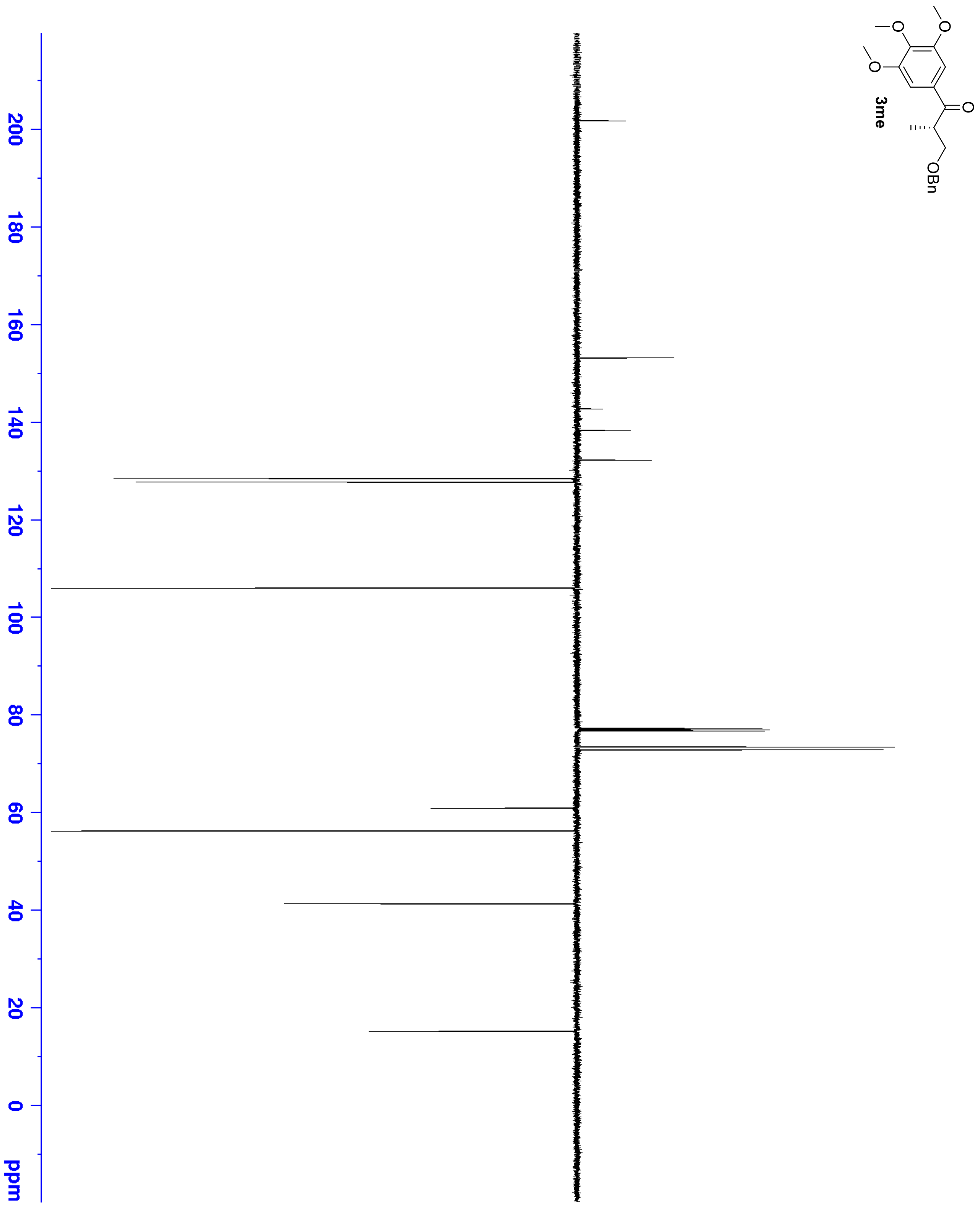

3me
OBn
O
200
180
160
140
120
100
80
60
40
20
0
ppm

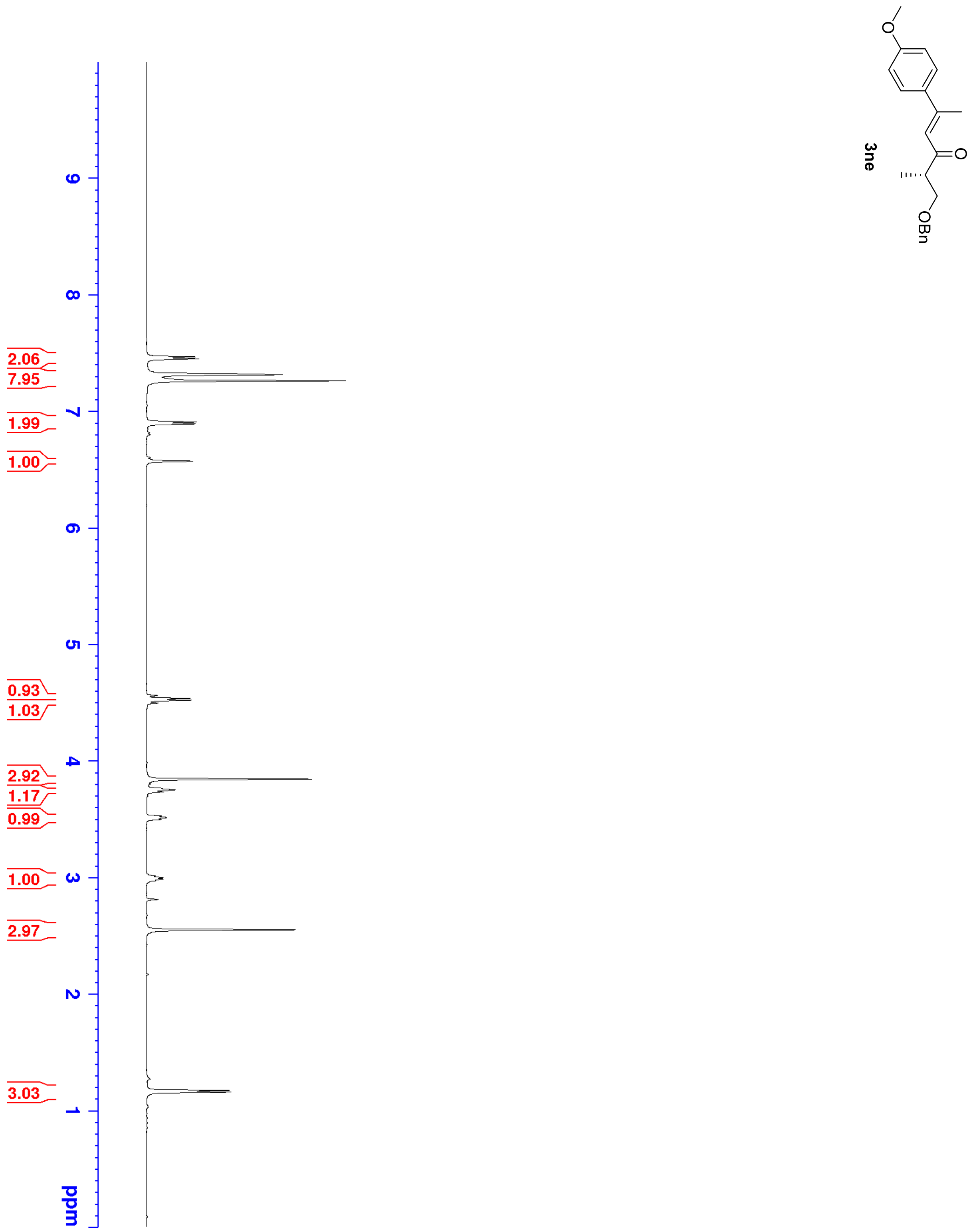
OBn
3ne
2.06
7.95
1.99
1.00
0.93
1.03
2.92
1.17
0.99
1.00
2.97
3.03
9
8
7
6
5
4
3
2
1
ppm

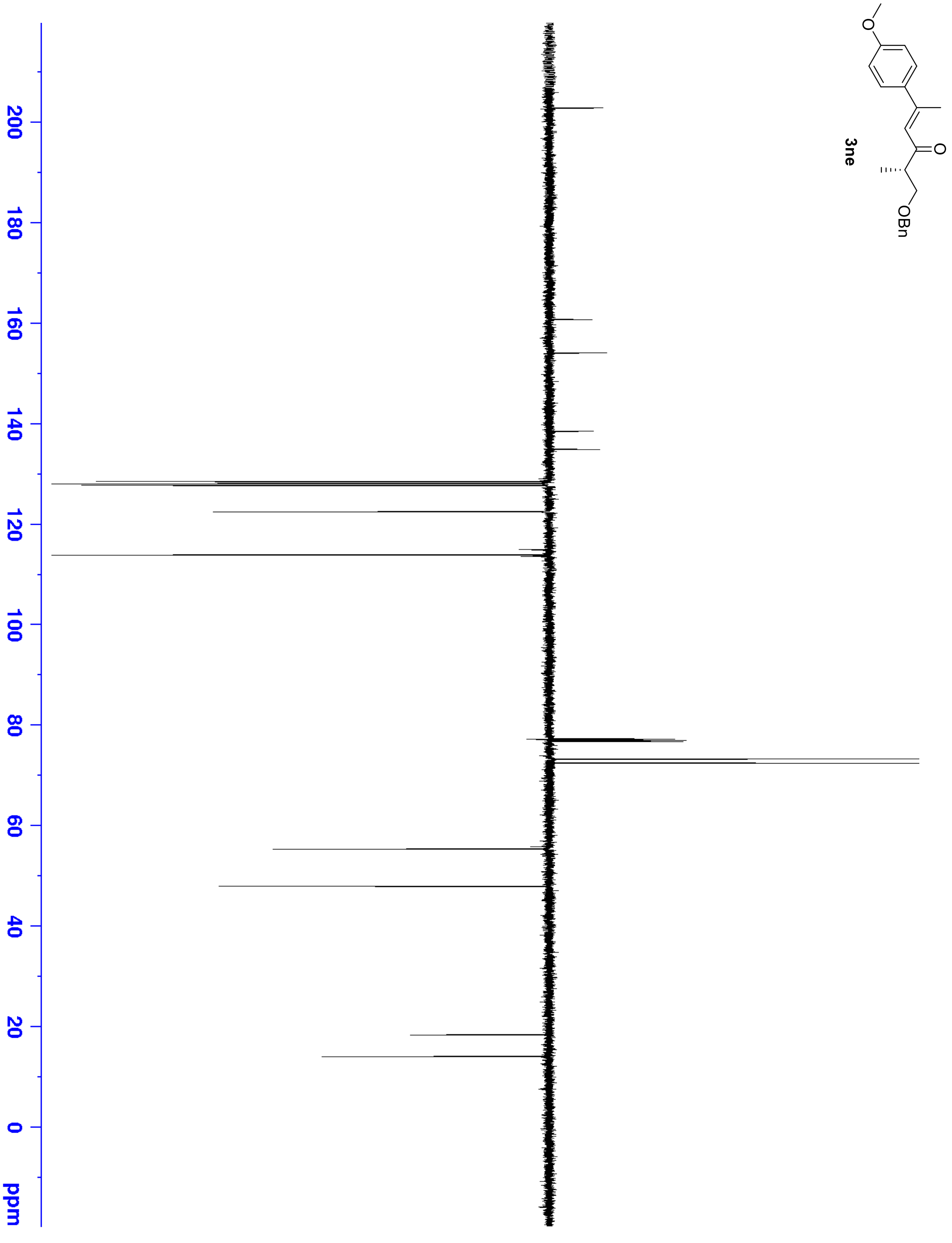

OBn
O
3ne
200
180
160
140
120
100
80
60
40
20
0
ppm

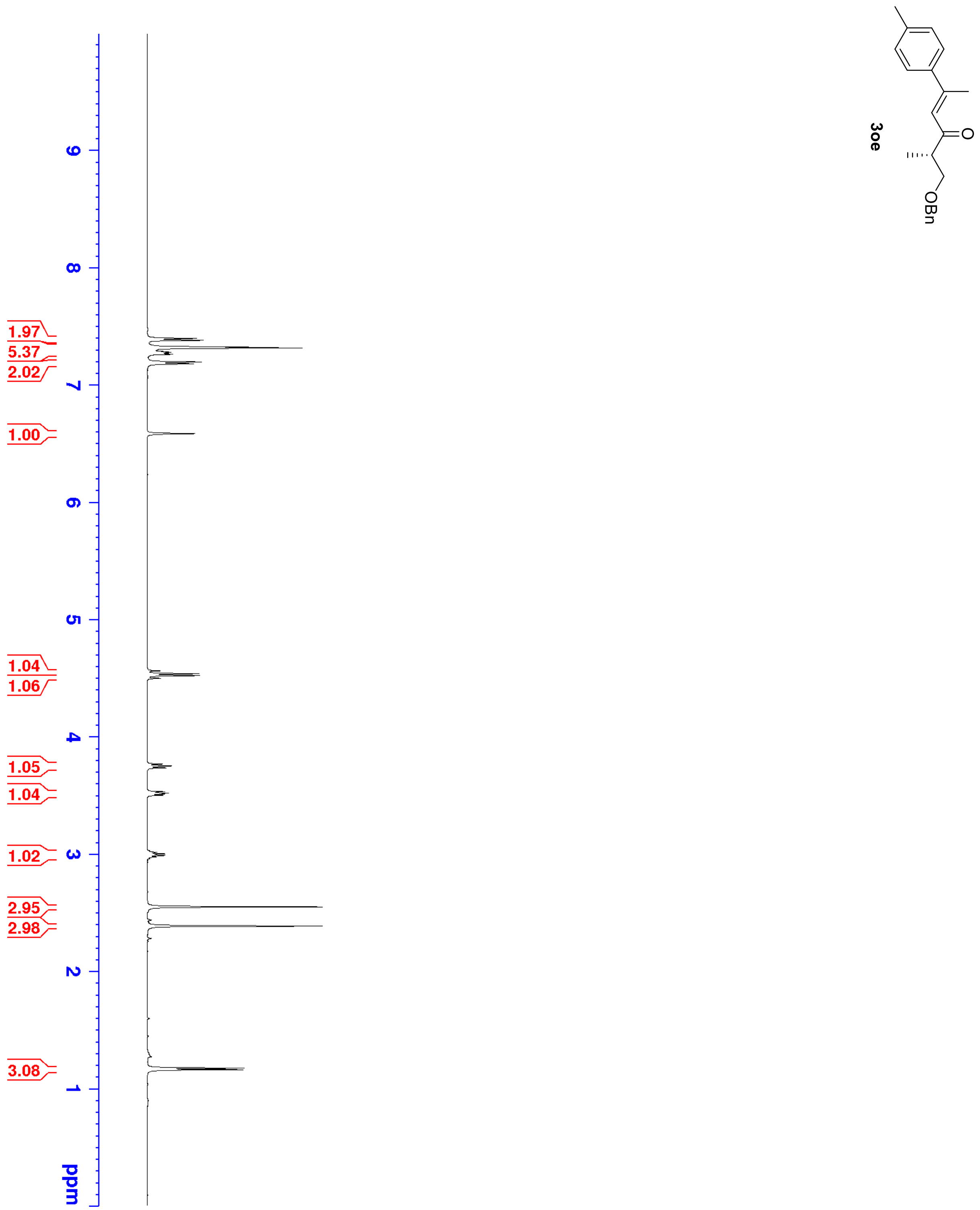
3oe
OBn
O
ppm
9
8
7
6
5
4
3
2
1
1.97
5.37
2.02
1.00
1.04
1.06
1.05
1.04
1.02
2.95
2.98
3.08

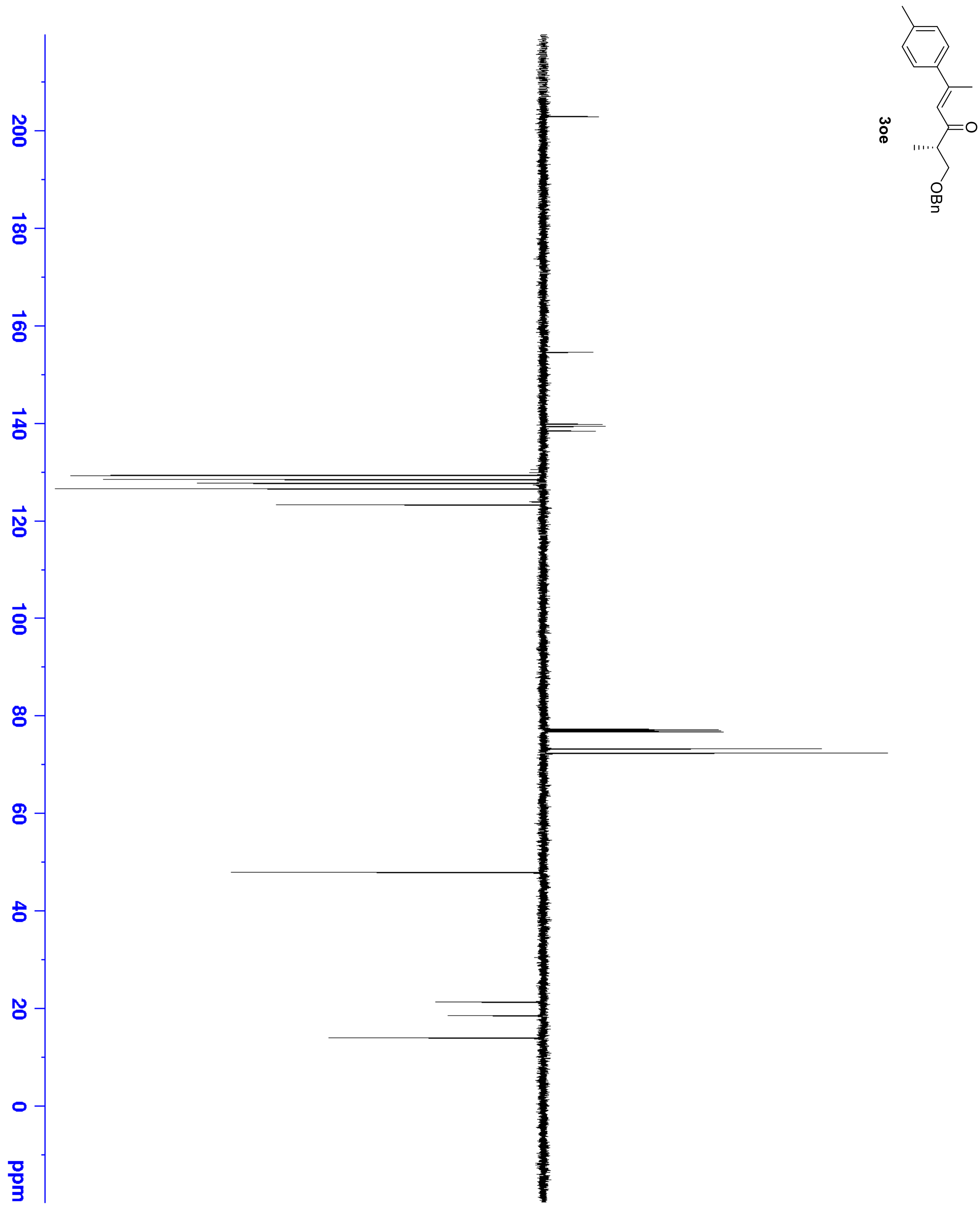
3oe
OBn
O
200
180
160
140
120
100
80
60
40
20
0
ppm

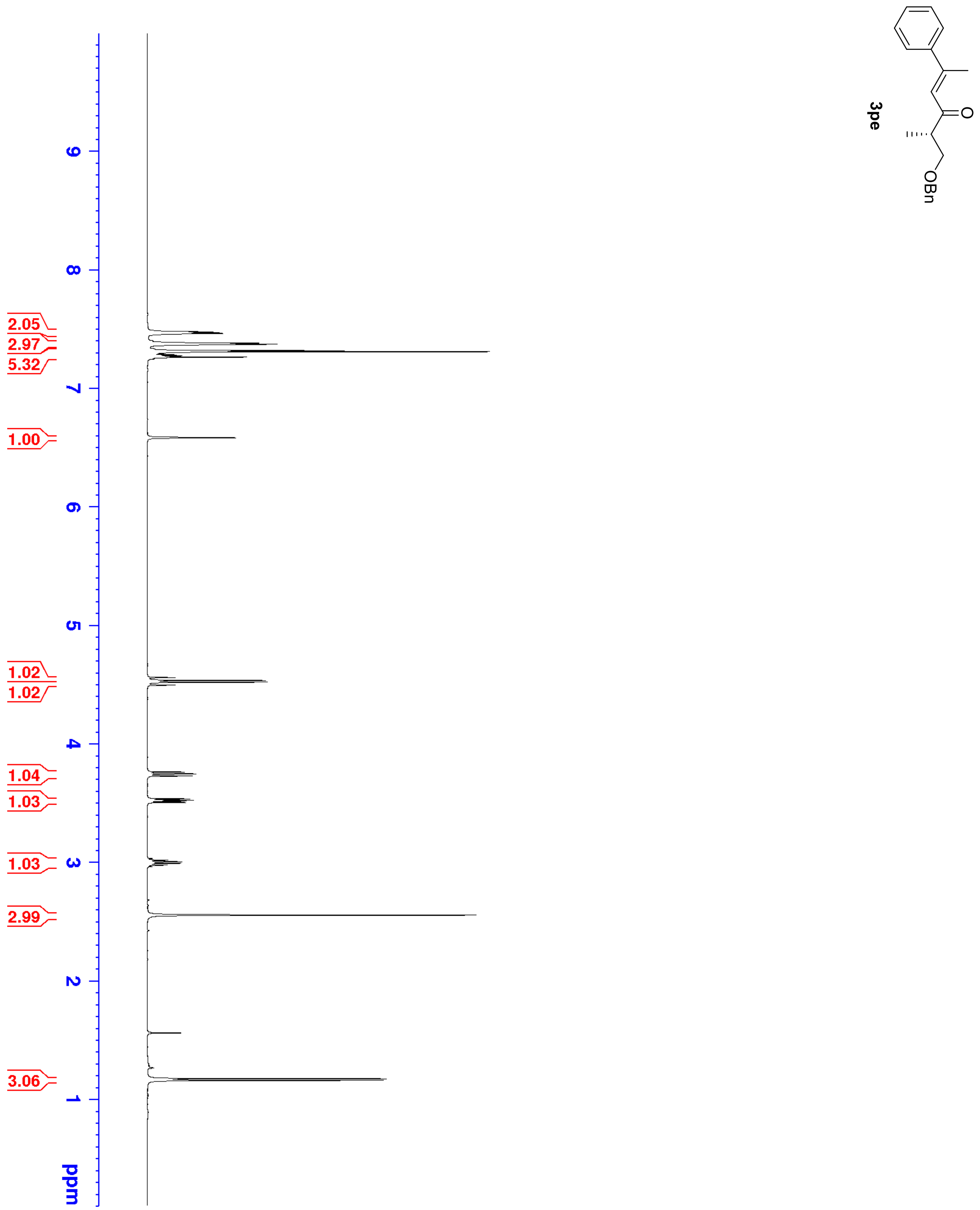
3pe
OBn
O
2.05
2.97
5.32
1.00
1.02
1.02
1.04
1.03
1.03
2.99
3.06
ppm

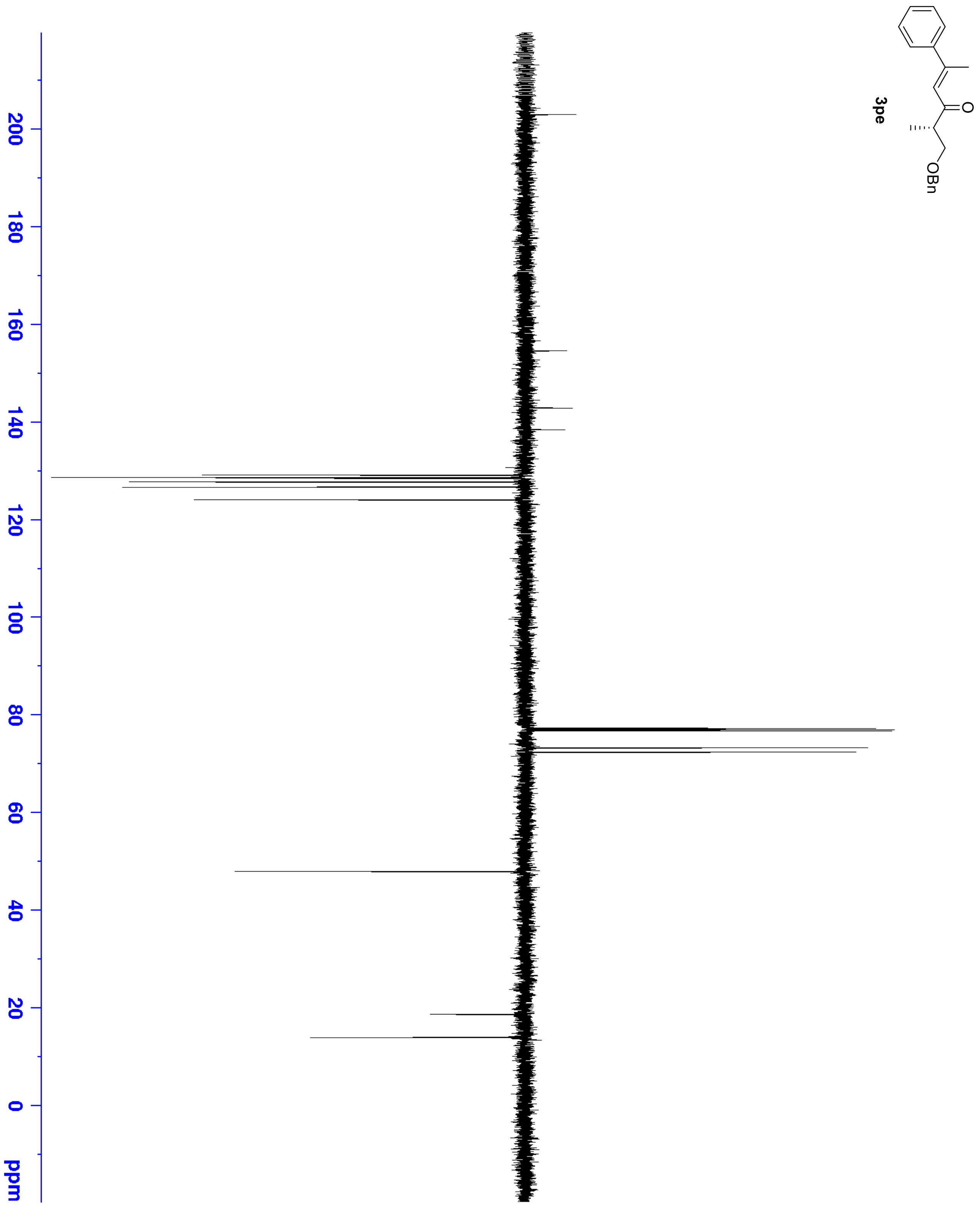

O
OBn
3pe
200
180
160
140
120
100
80
60
40
20
0
ppm

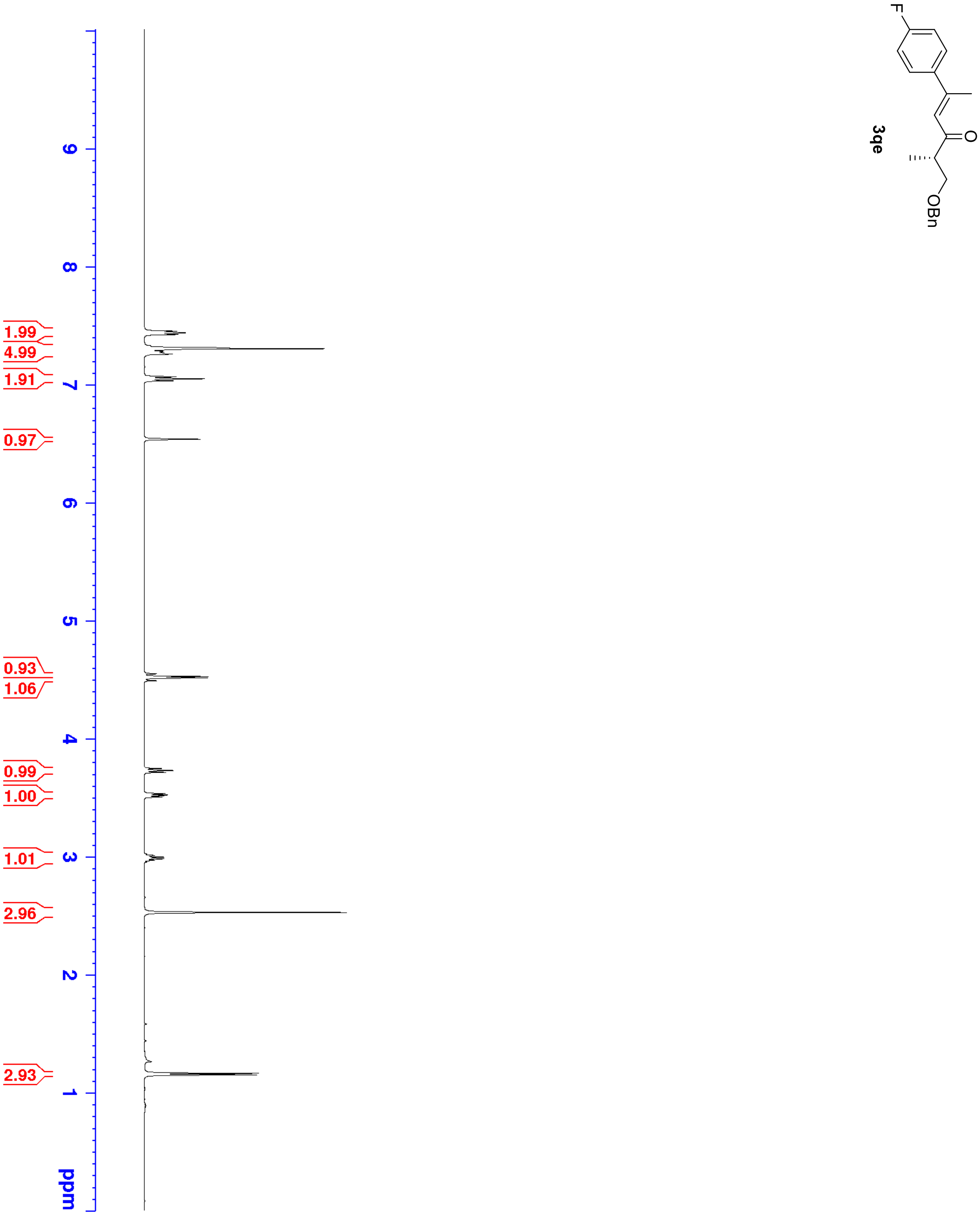

F
O
OBn
3qe
1.99
4.99
1.91
0.97
0.93
1.06
0.99
1.00
1.01
2.96
2.93
9
8
7
6
5
4
3
2
1
ppm

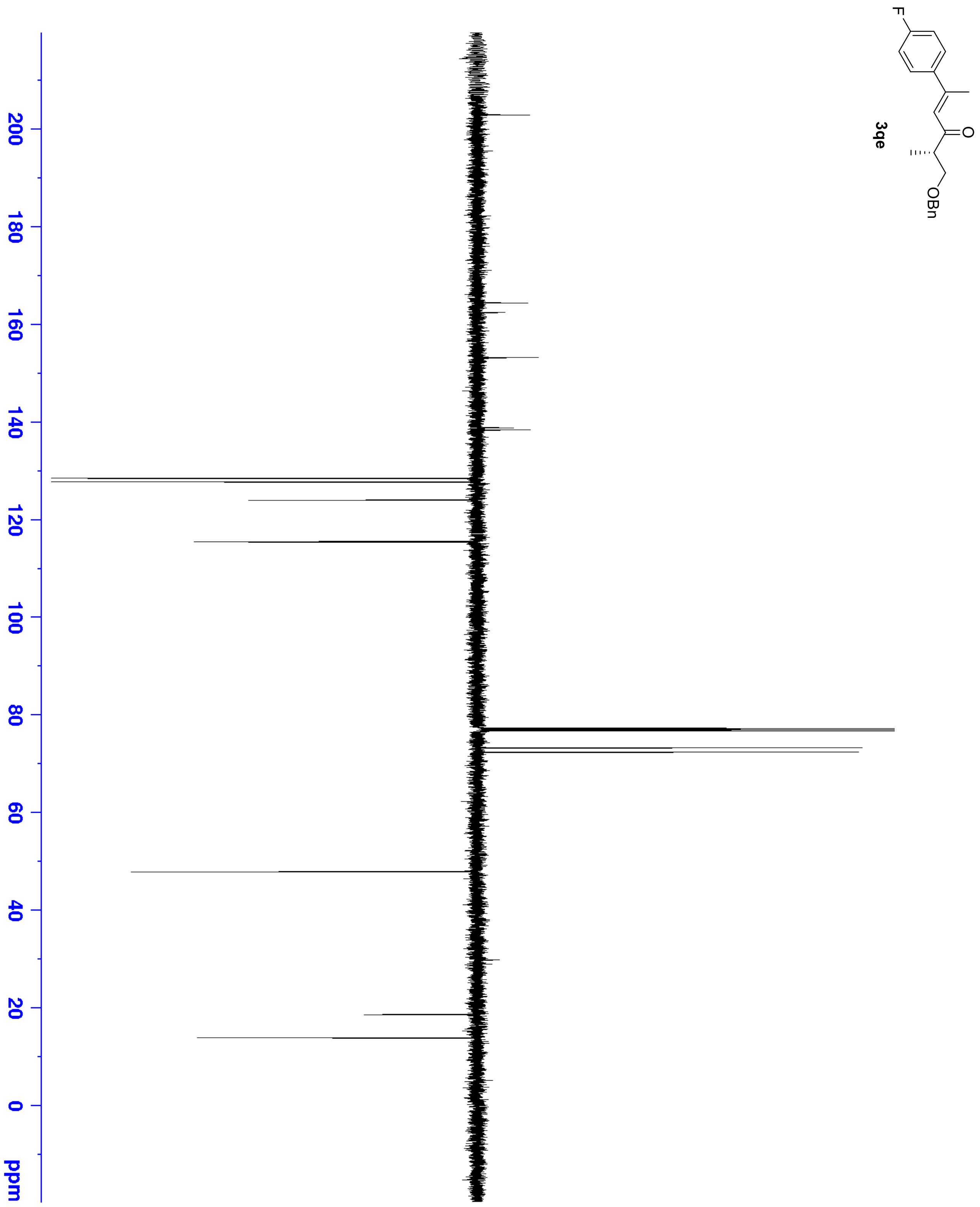
3qe
F
O
OBn
200
180
160
140
120
100
80
60
40
20
0
ppm

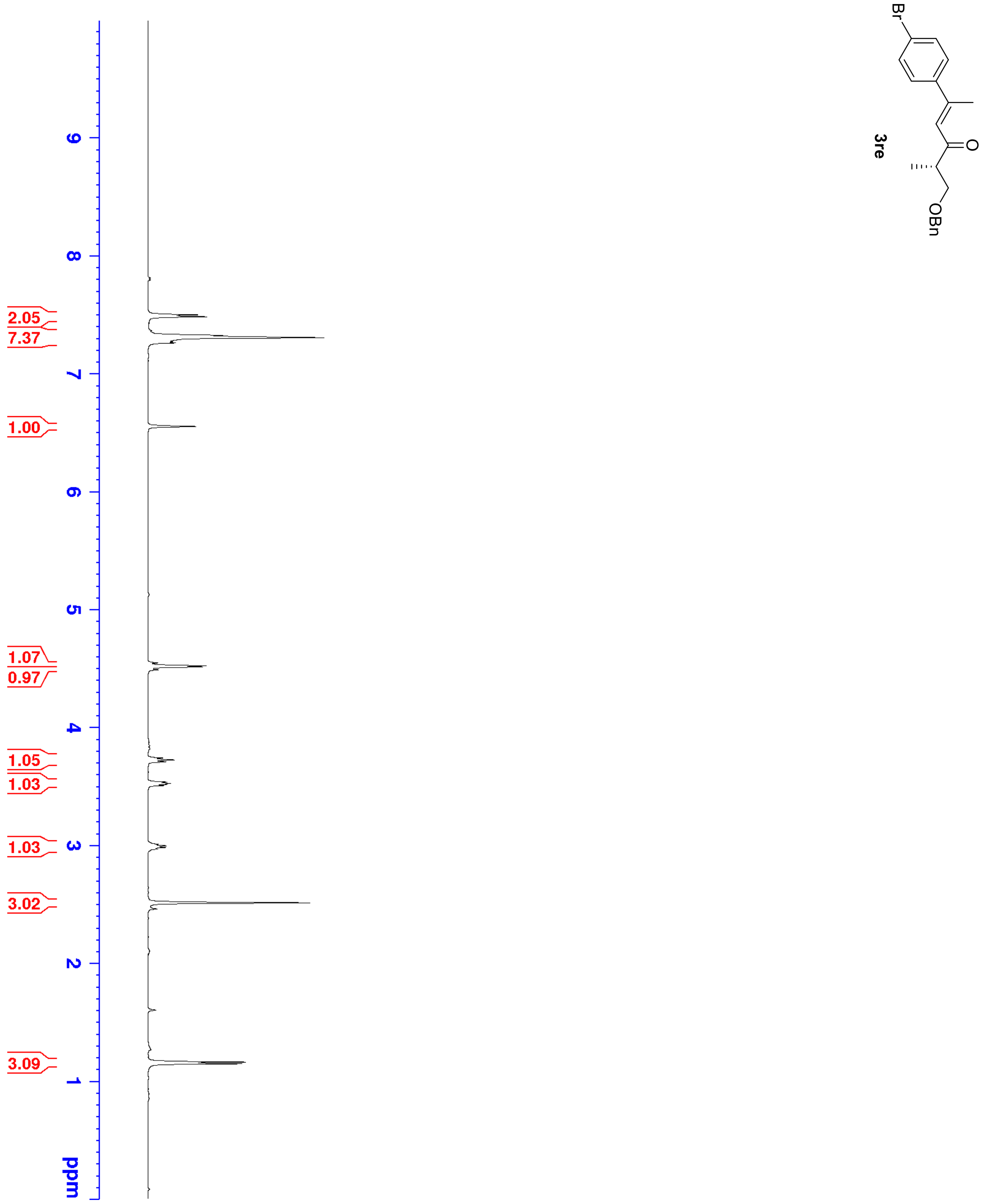
Br
OBn
3re
ppm
9
8
7
6
5
4
3
2
1
2.05
7.37
1.00
1.07
0.97
1.05
1.03
1.03
3.02
3.09

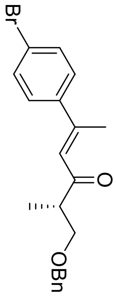

3re

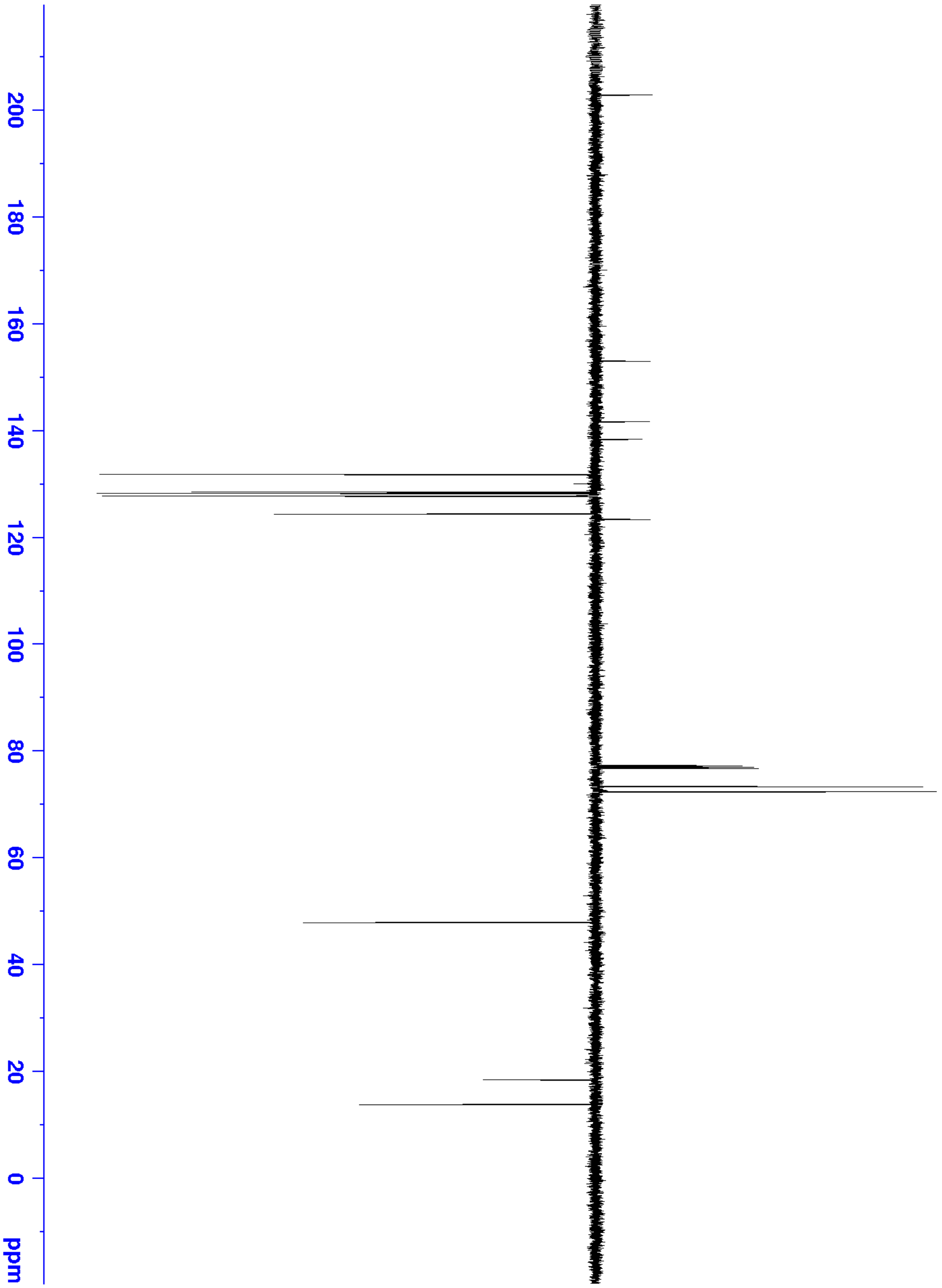

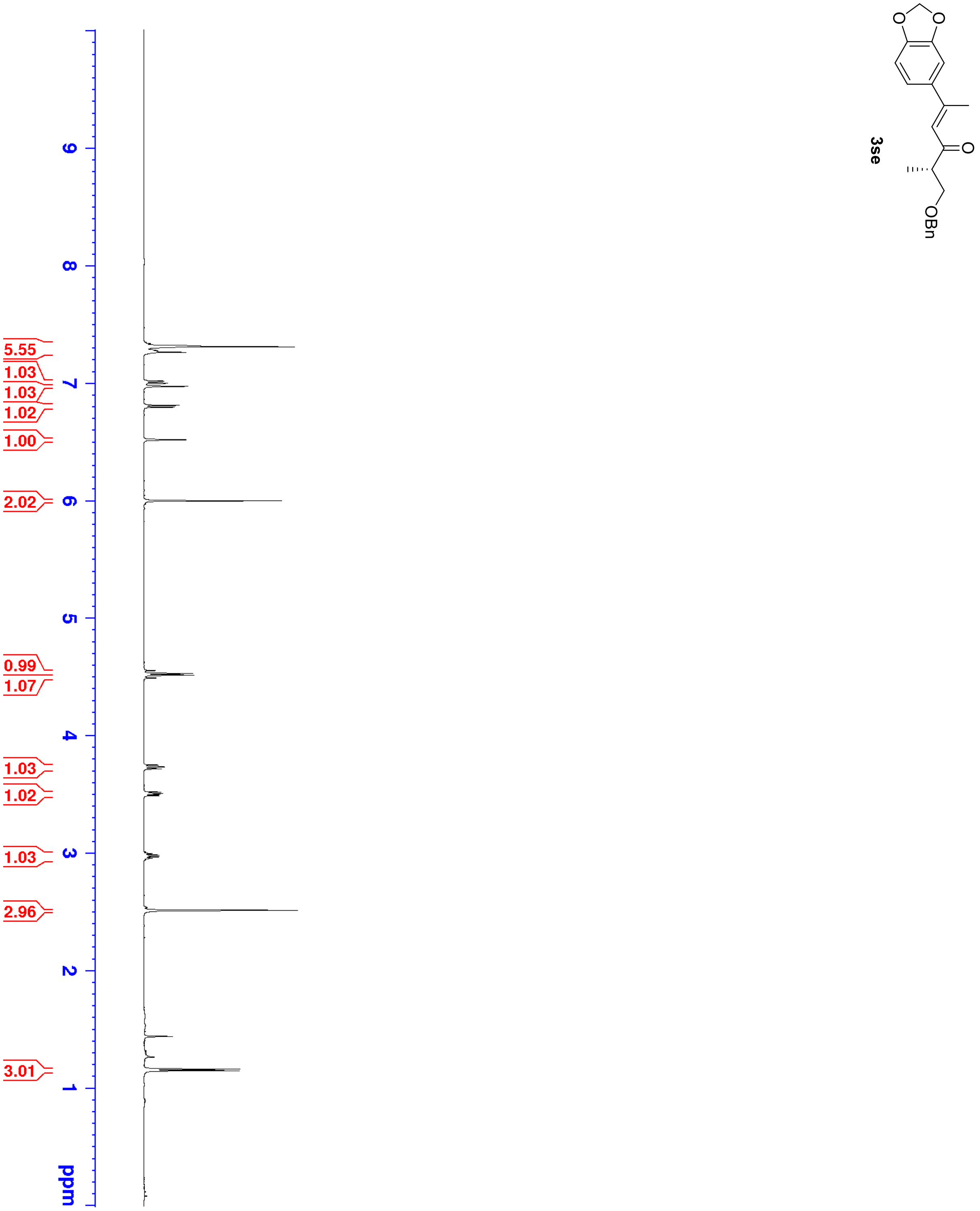
3se
OBn
5.55
1.03
1.03
1.02
1.00
2.02
0.99
1.07
1.03
1.02
1.03
2.96
3.01
9
8
7
6
5
4
3
2
1
ppm

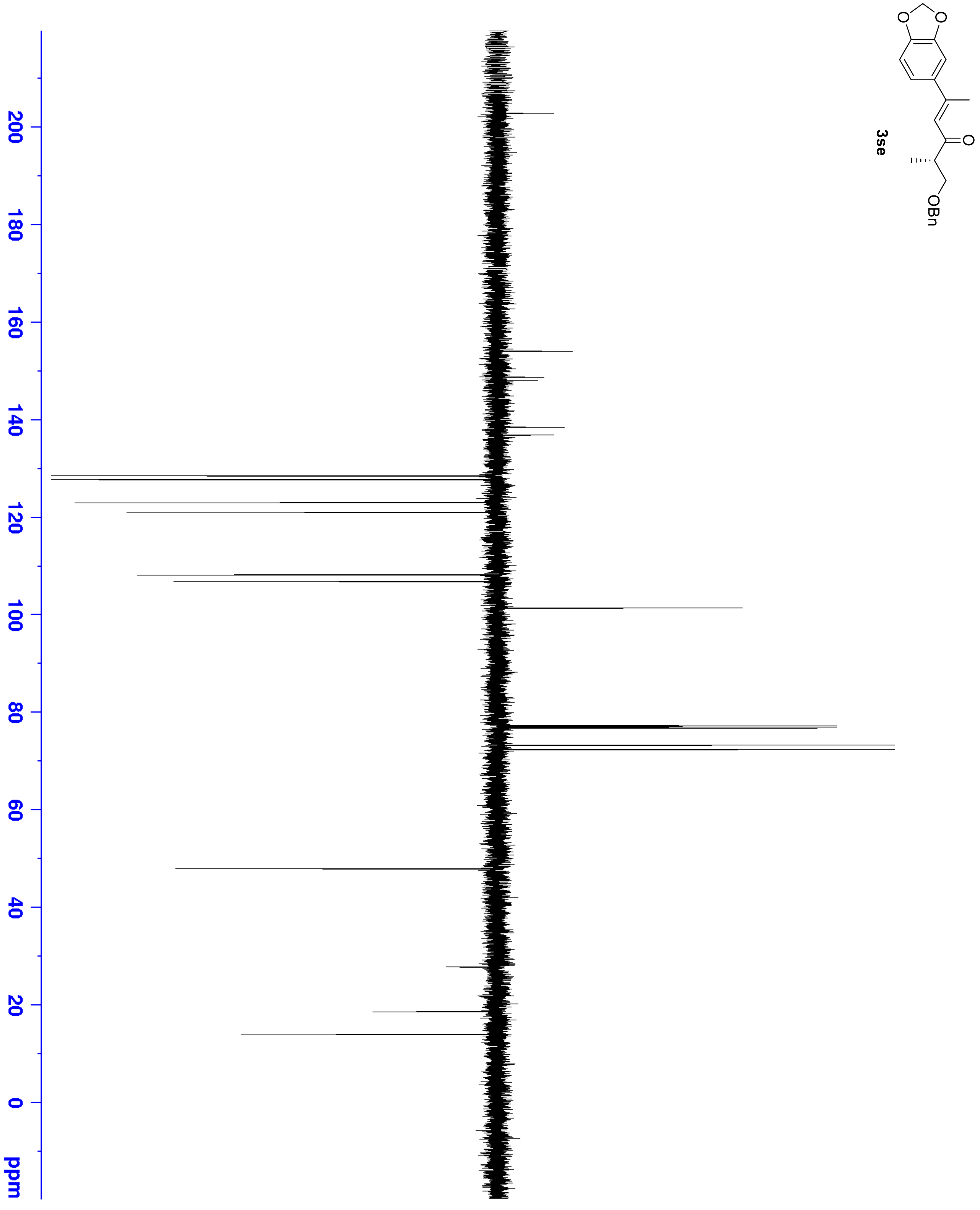
3se
OBn
O
ppm
200
180
160
140
120
100
80
60
40
20
0

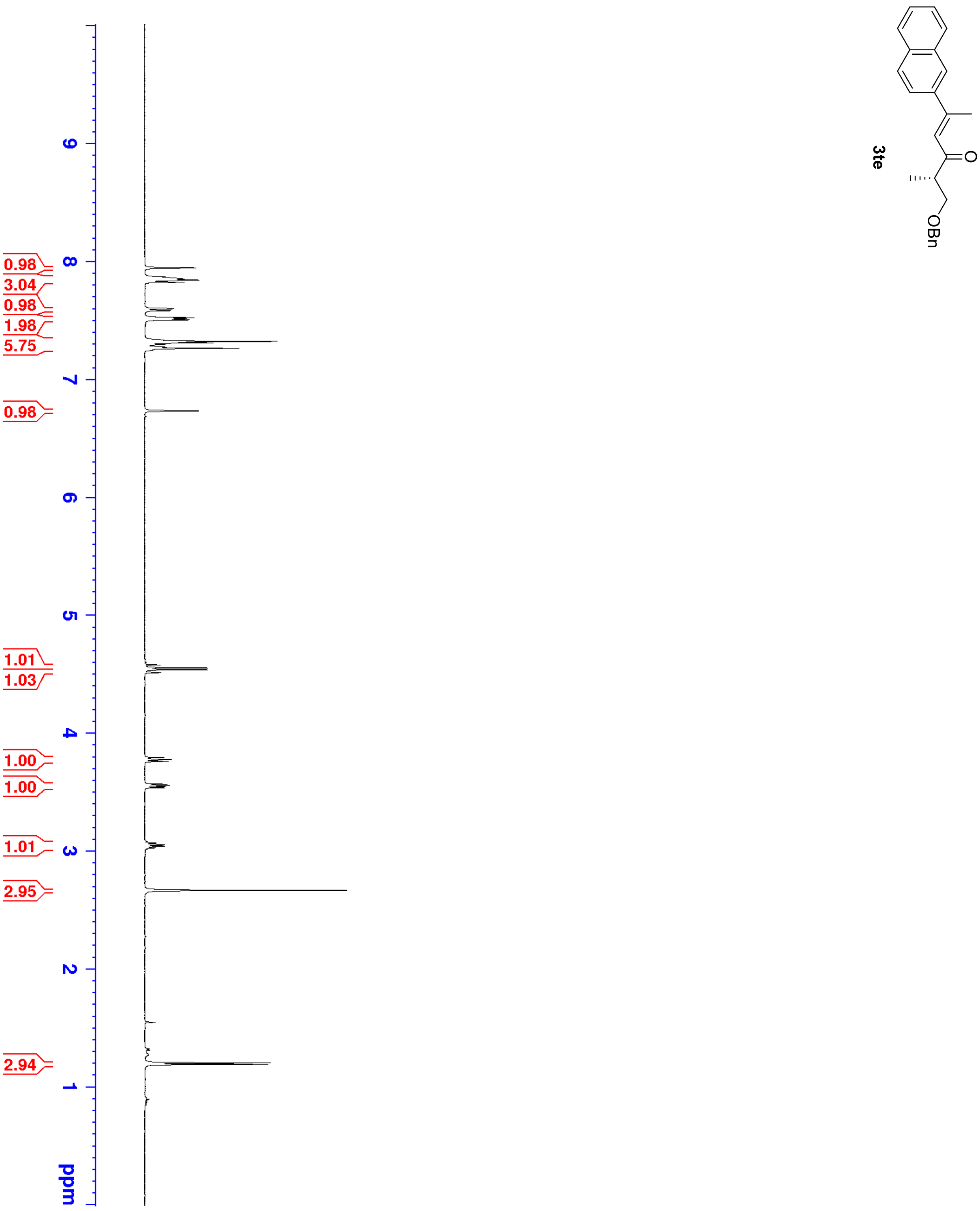

3te
OBn
O
0.98
3.04
0.98
1.98
5.75
0.98
1.01
1.03
1.00
1.00
1.01
2.95
2.94
9
8
7
6
5
4
3
2
1
ppm

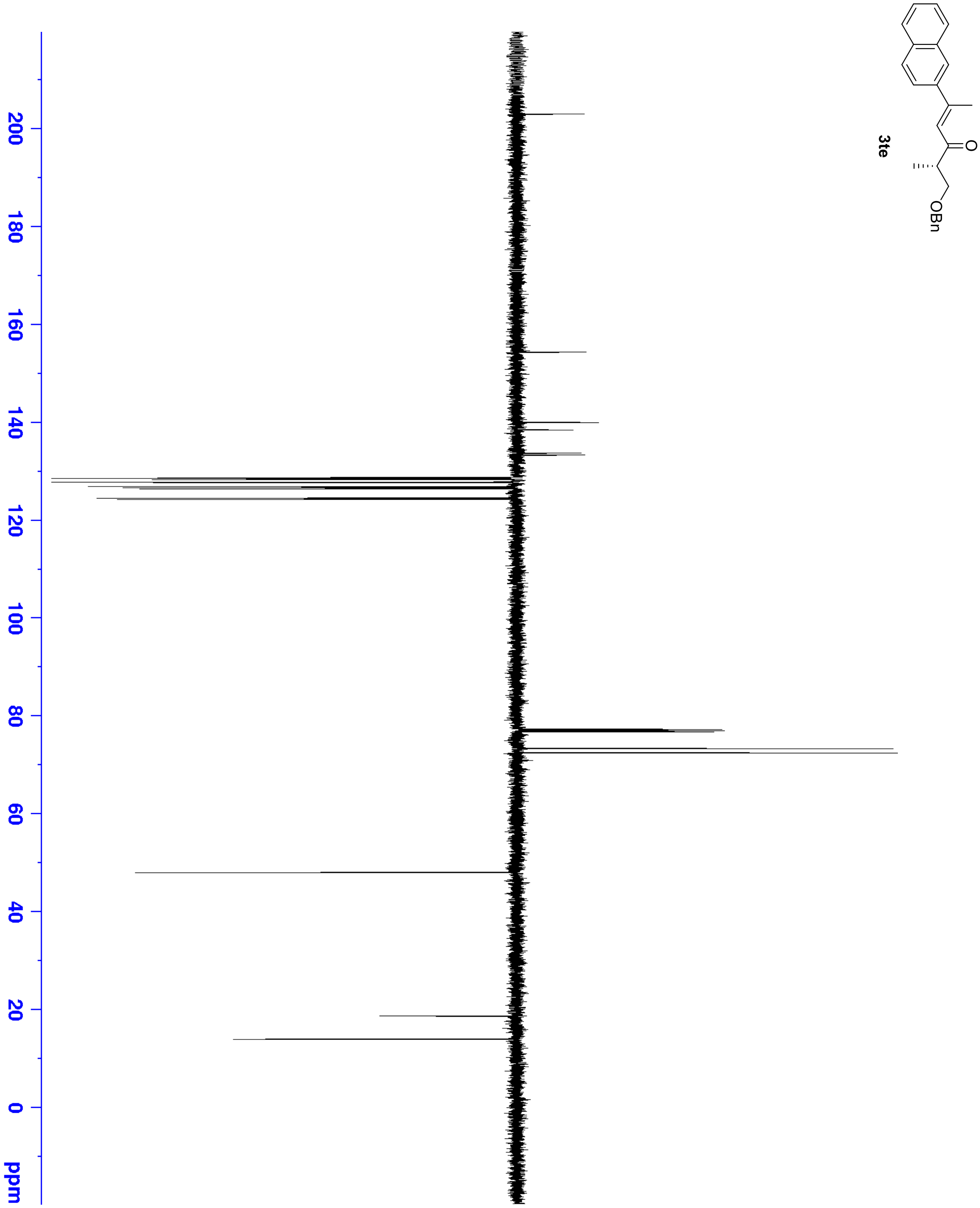

3te
OBn
O
200
180
160
140
120
100
80
60
40
20
0
ppm

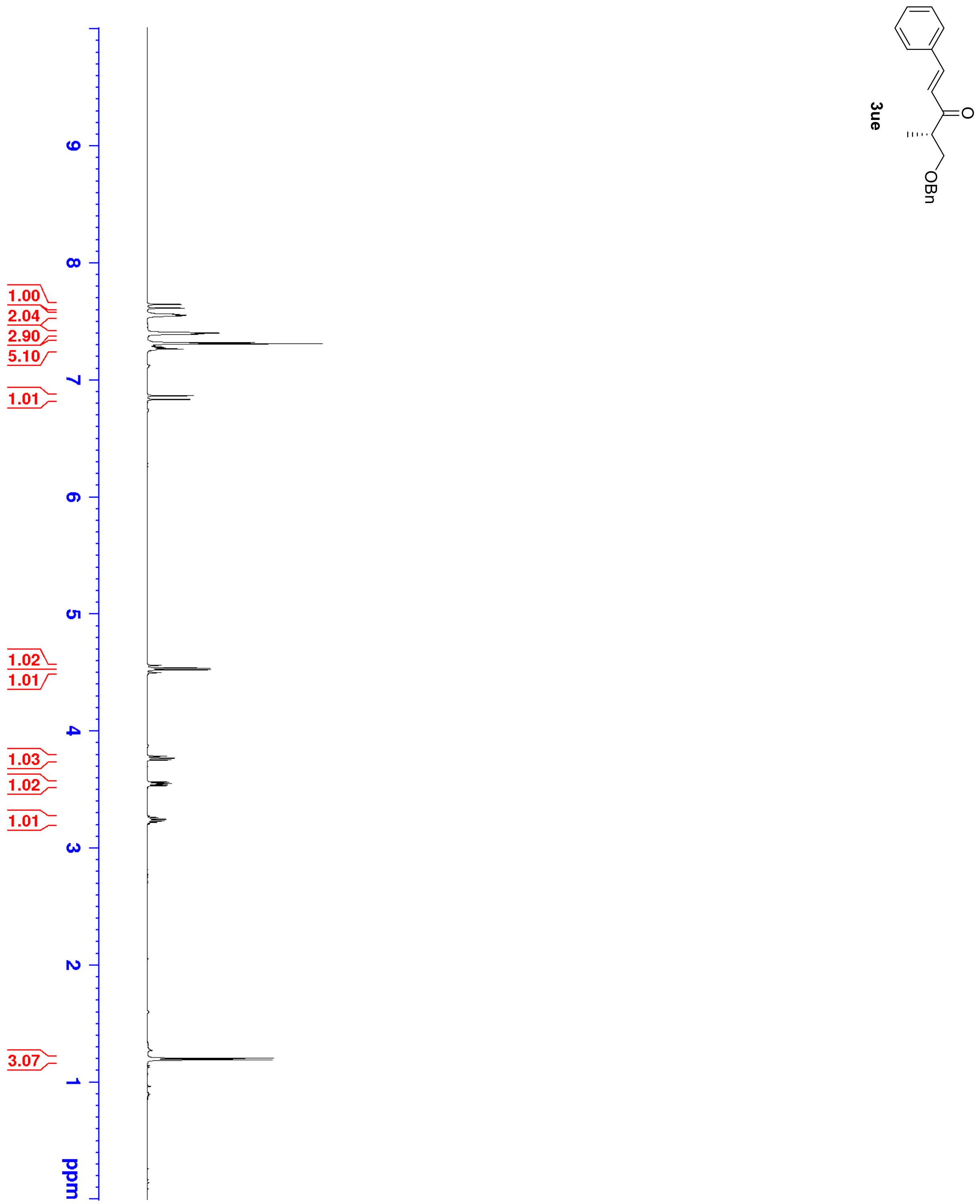
3ue
OBn
O
1.00
2.04
2.90
5.10
1.01
1.02
1.01
1.03
1.02
1.01
3.07
9
8
7
6
5
4
3
2
1
ppm

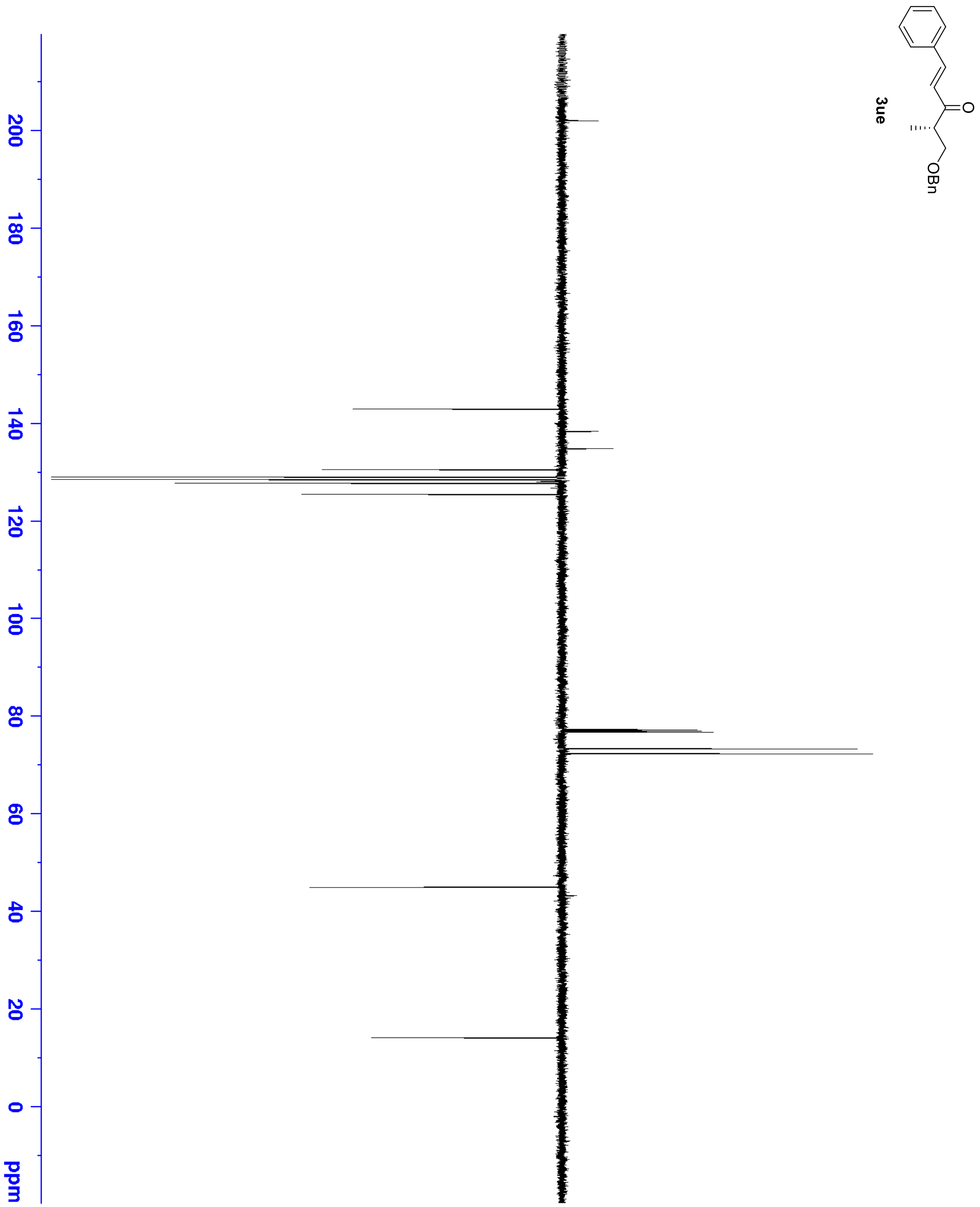

3ue
O
OBn
200
180
160
140
120
100
80
60
40
20
0
ppm

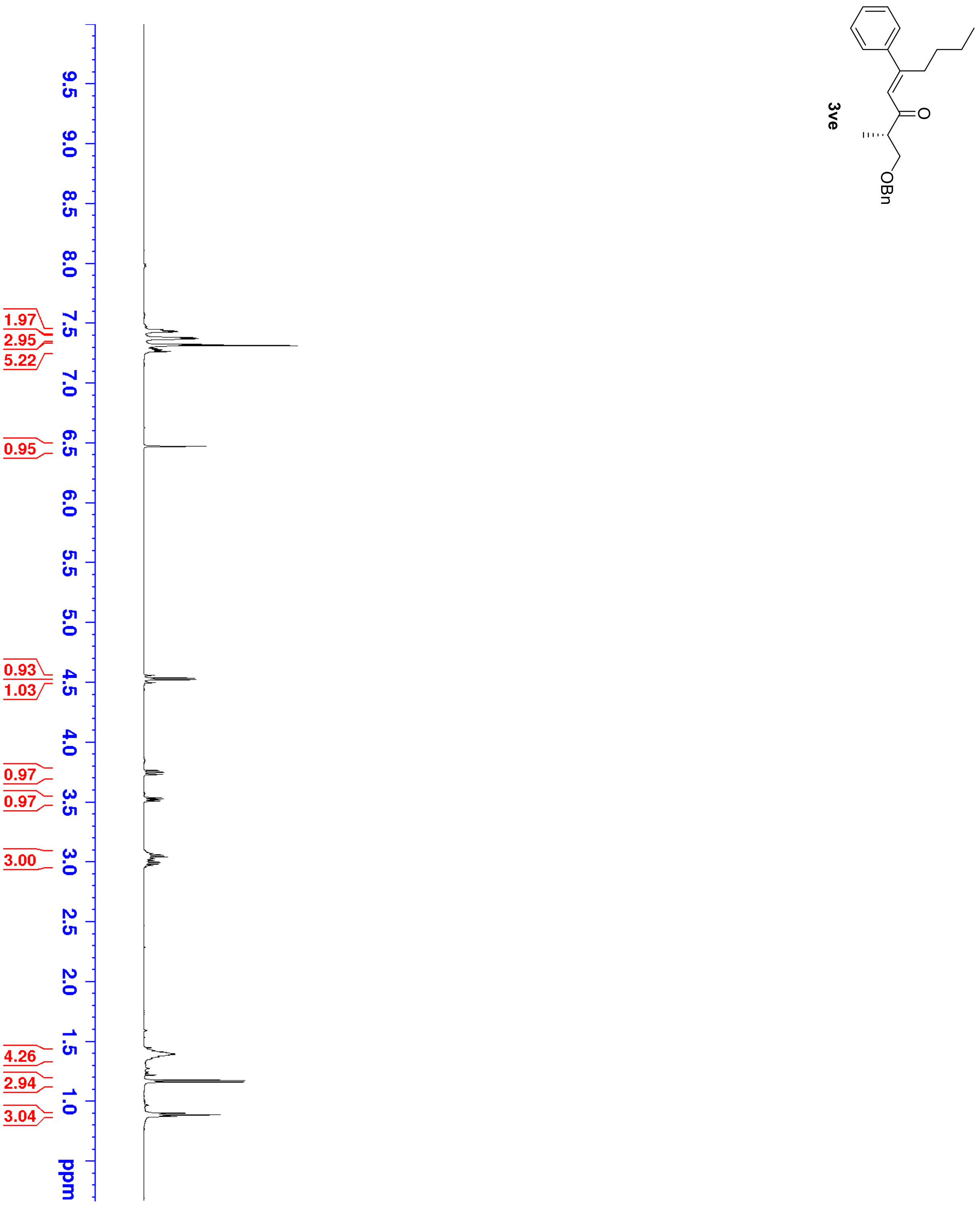
3ve
O
OBn
9.5
9.0
8.5
8.0
7.5
7.0
6.5
6.0
5.5
5.0
4.5
4.0
3.5
3.0
2.5
2.0
1.5
1.0
ppm
1.97
2.95
5.22
0.95
0.93
1.03
0.97
0.97
3.00
4.26
2.94
3.04

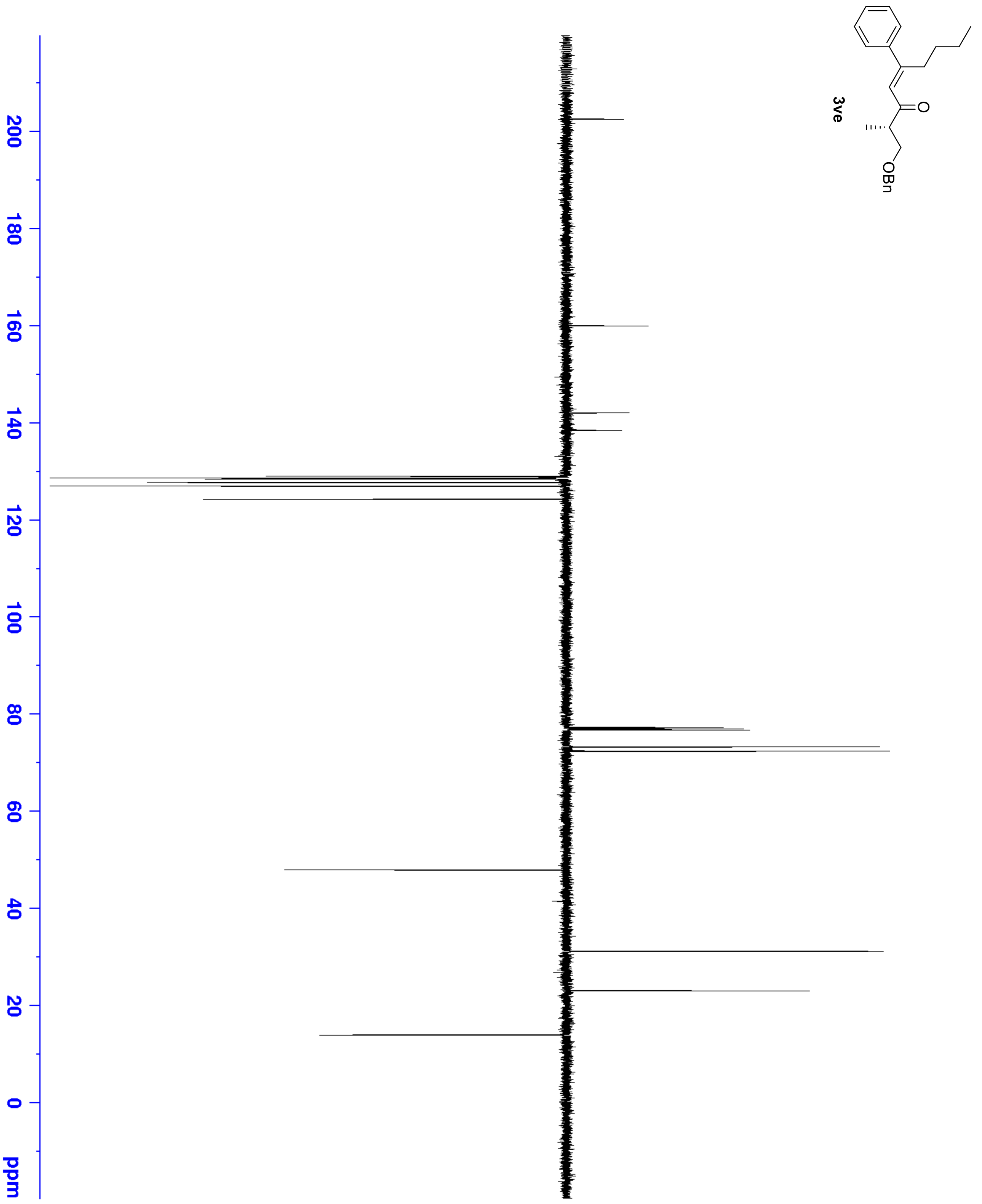
3ve
OBn
O
200
180
160
140
120
100
80
60
40
20
0
ppm

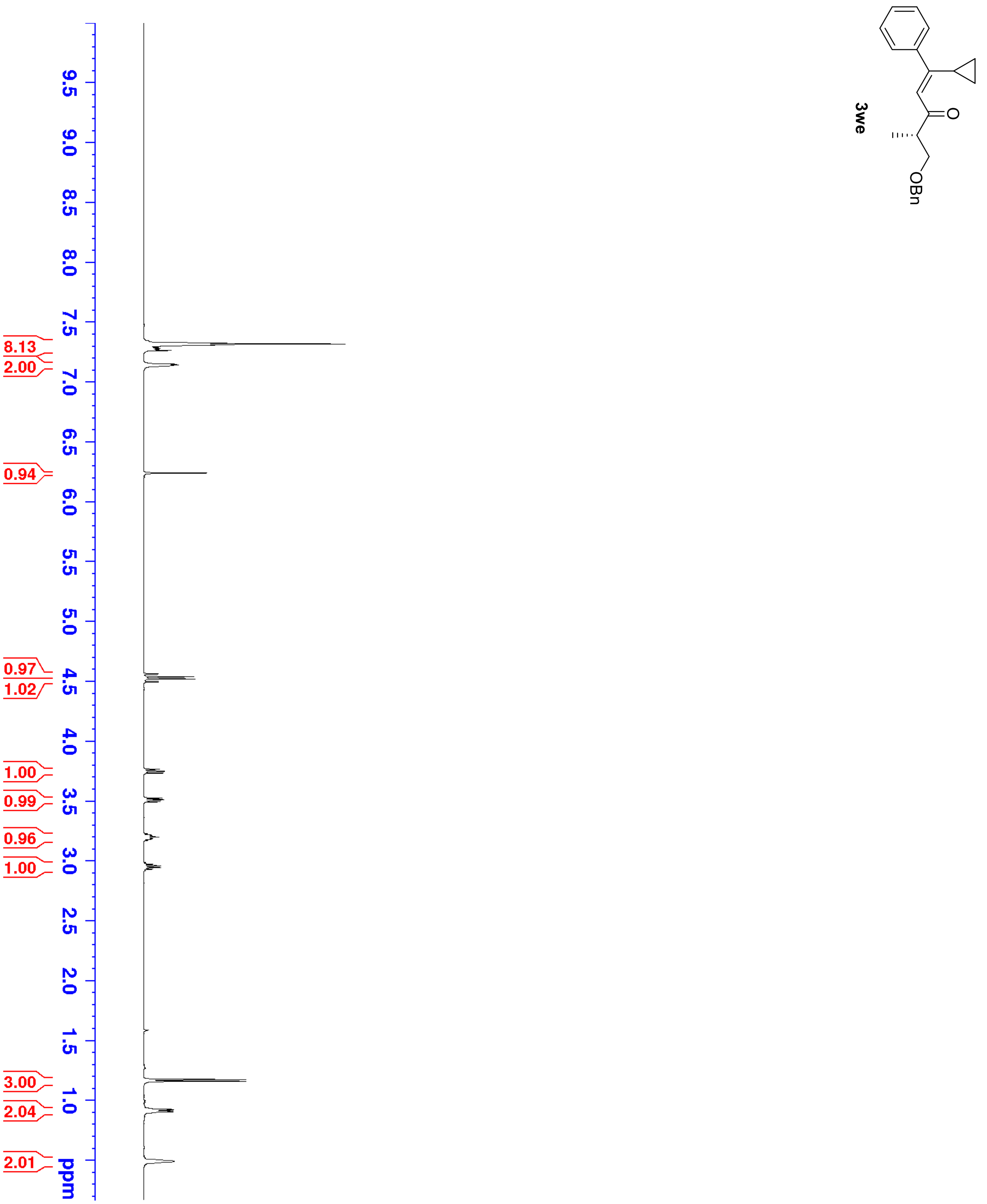
3we
OBn
O
9.5
9.0
8.5
8.0
7.5
7.0
6.5
6.0
5.5
5.0
4.5
4.0
3.5
3.0
2.5
2.0
1.5
1.0
ppm
8.13
2.00
0.94
0.97
1.02
1.00
0.99
0.96
1.00
3.00
2.04
2.01

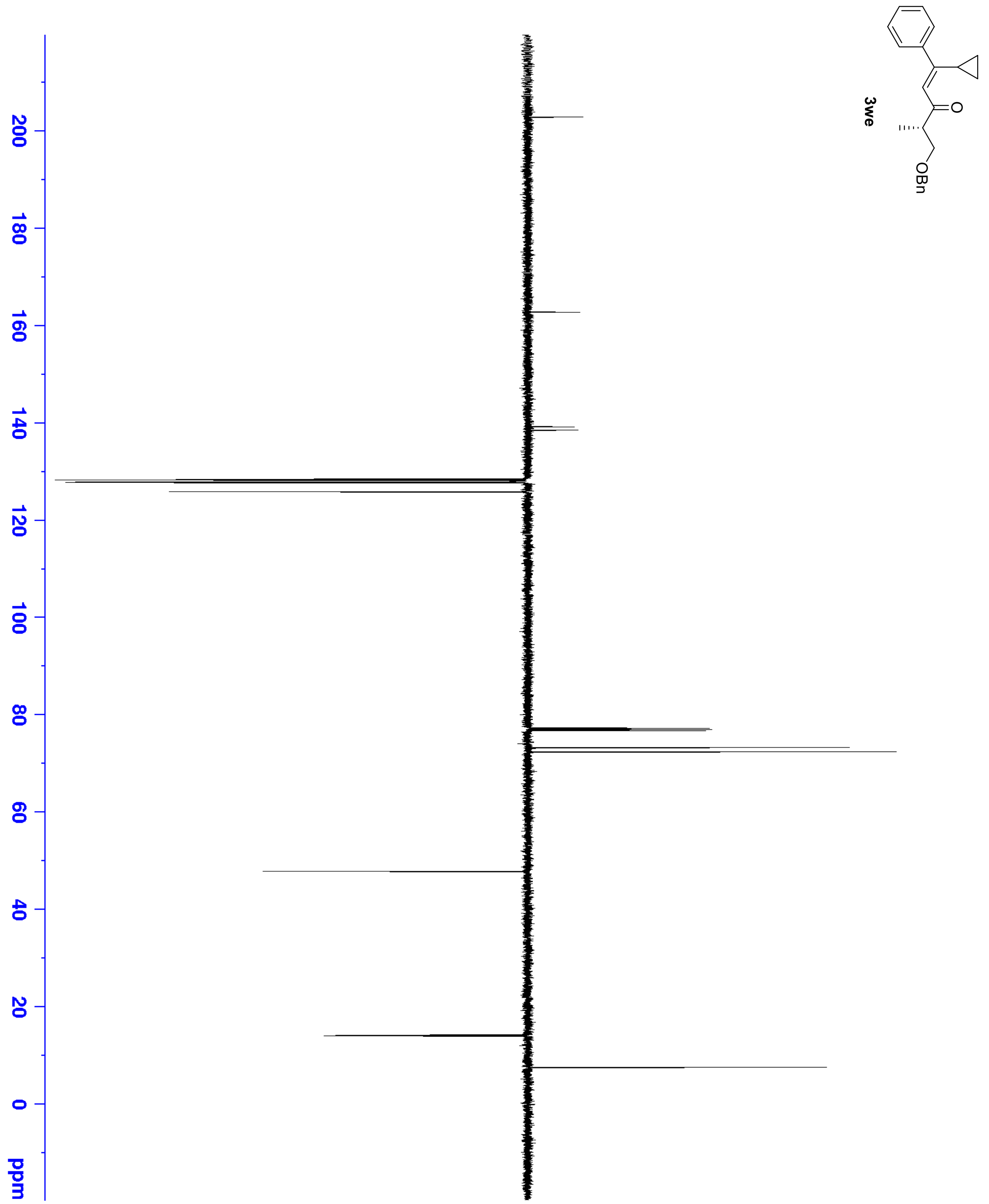

3we
O
OBn
200
180
160
140
120
100
80
60
40
20
0
ppm

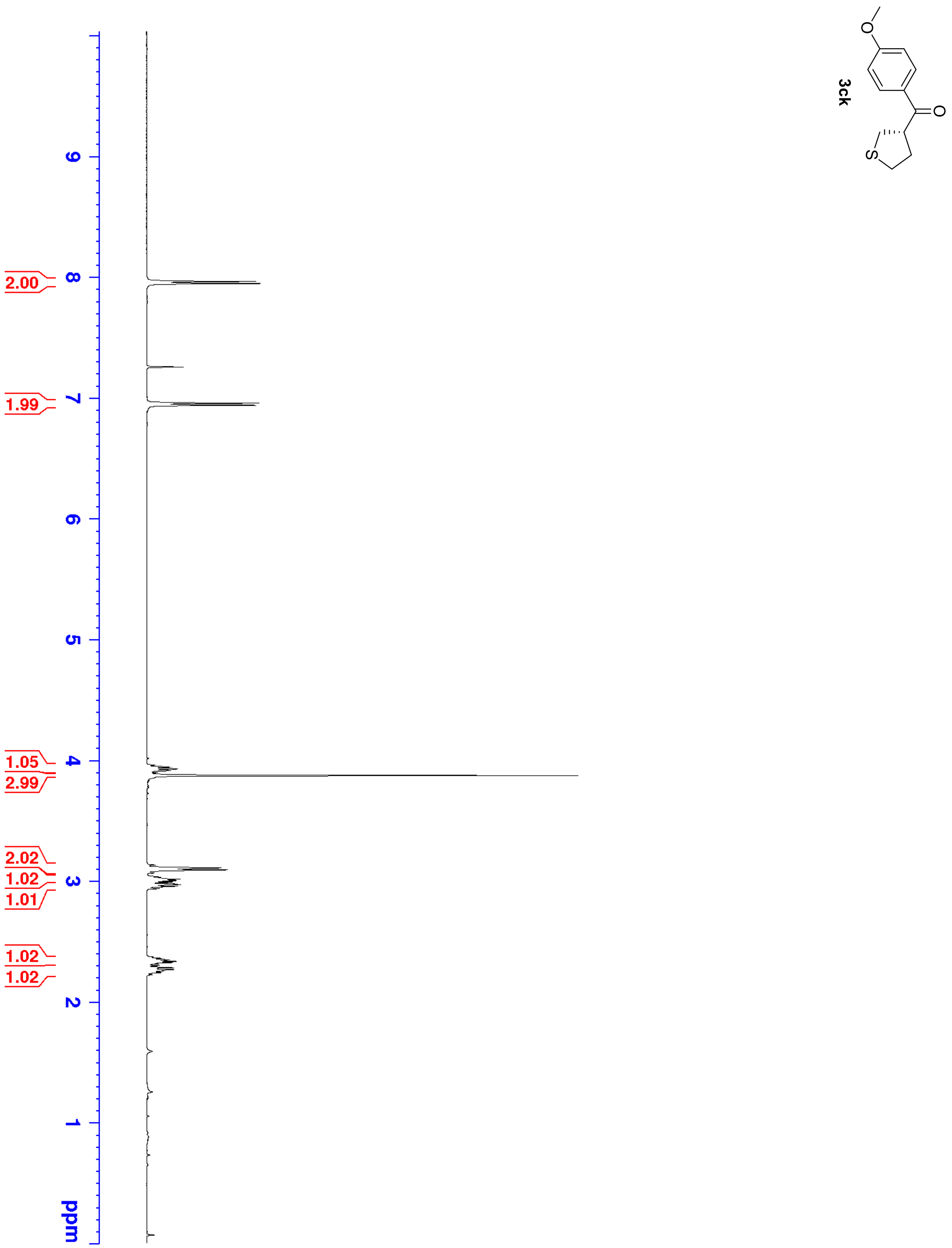
3ck
2.00
1.99
1.05
2.99
2.02
1.02
1.01
1.02
1.02
9
8
7
6
5
4
3
2
1
ppm

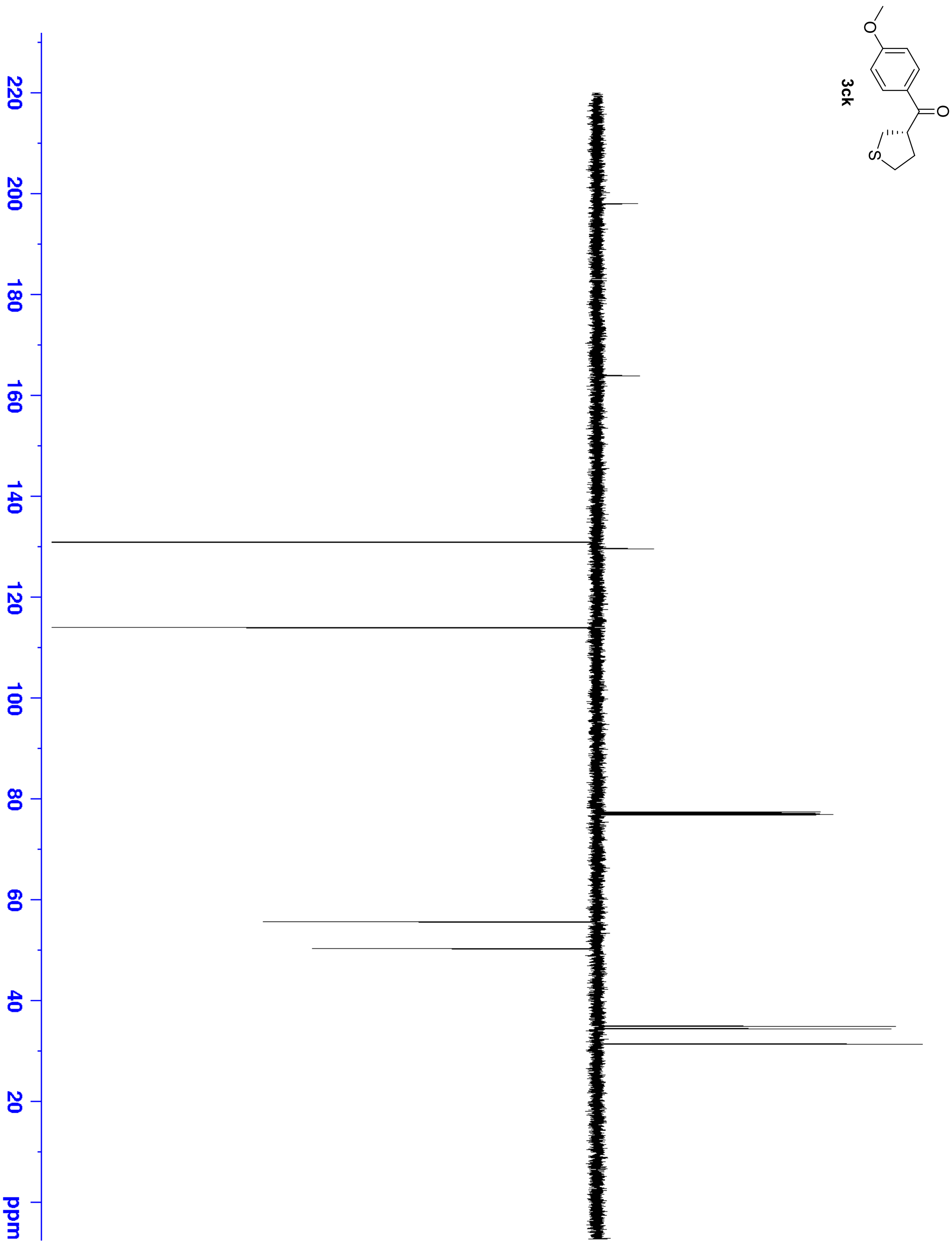
3ck
220
200
180
160
140
120
100
80
60
40
20
ppm

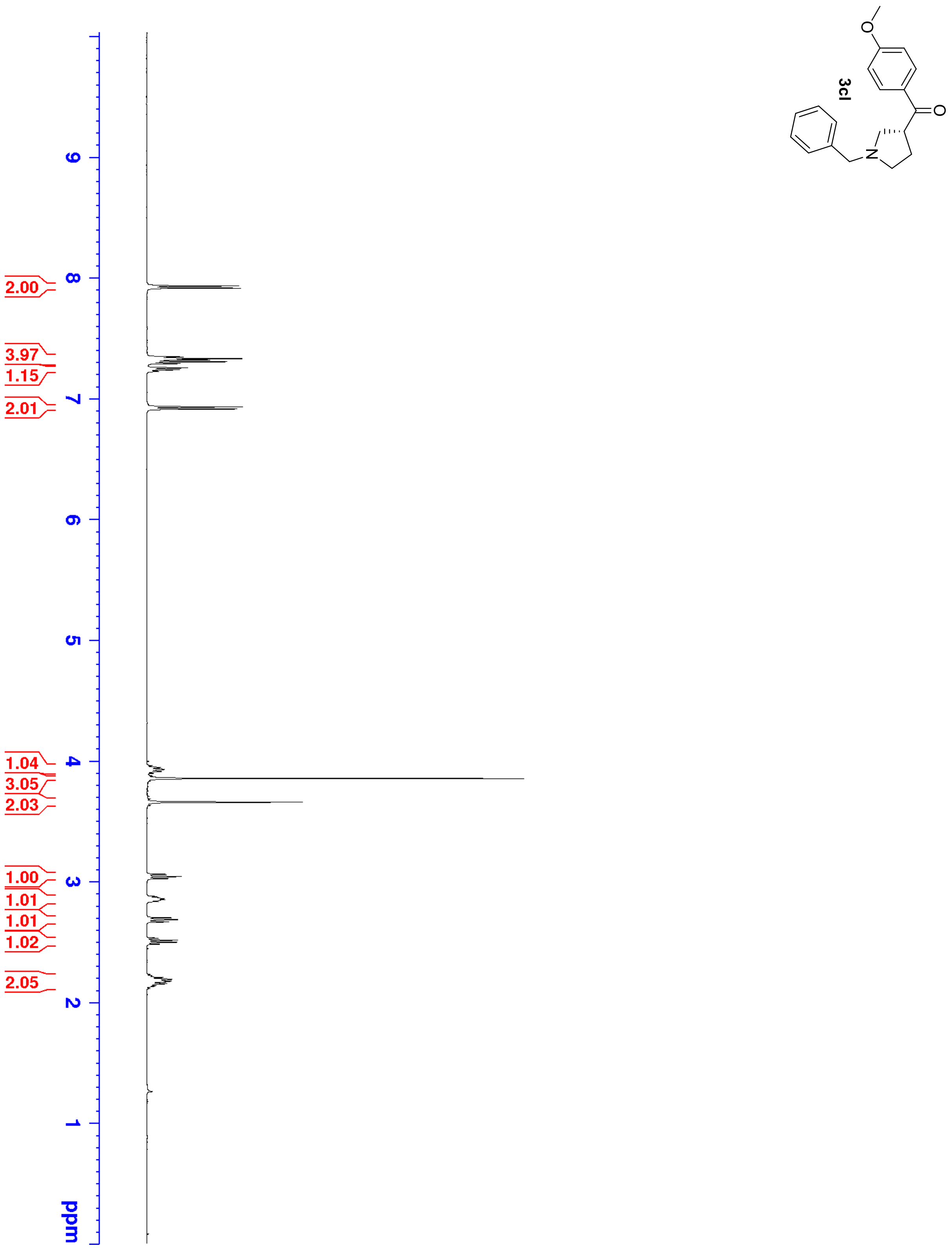
3cl
ppm

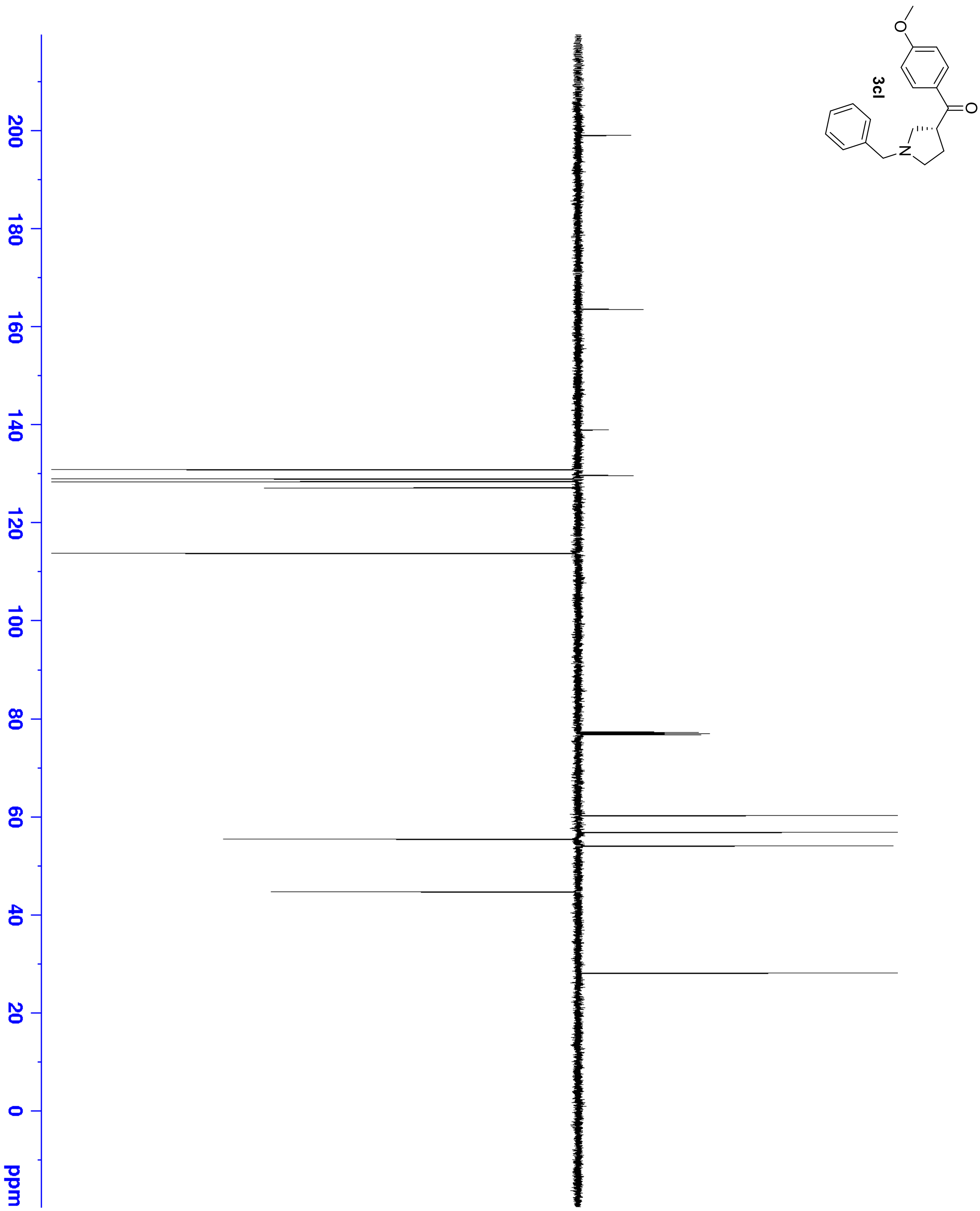
3cl
200
180
160
140
120
100
80
60
40
20
0
ppm

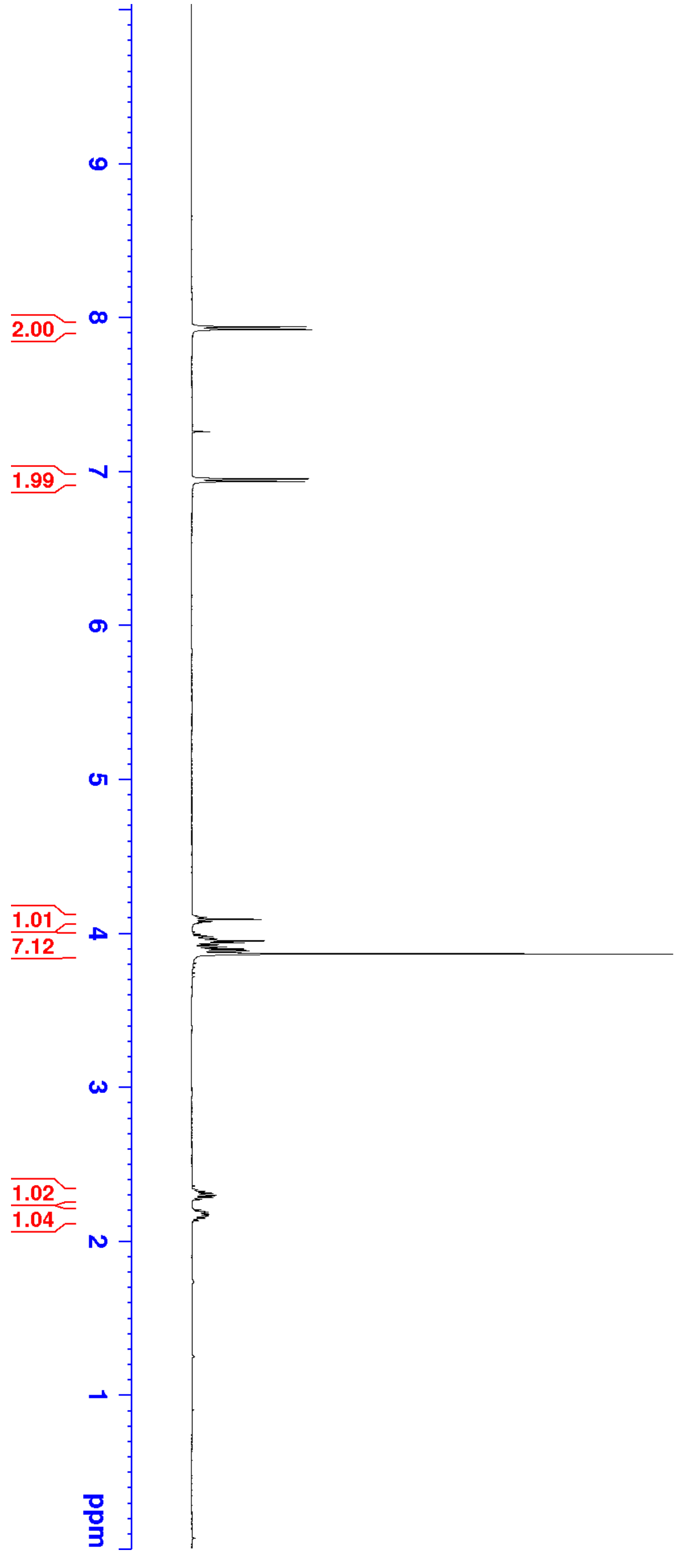

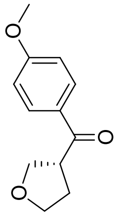

3cm

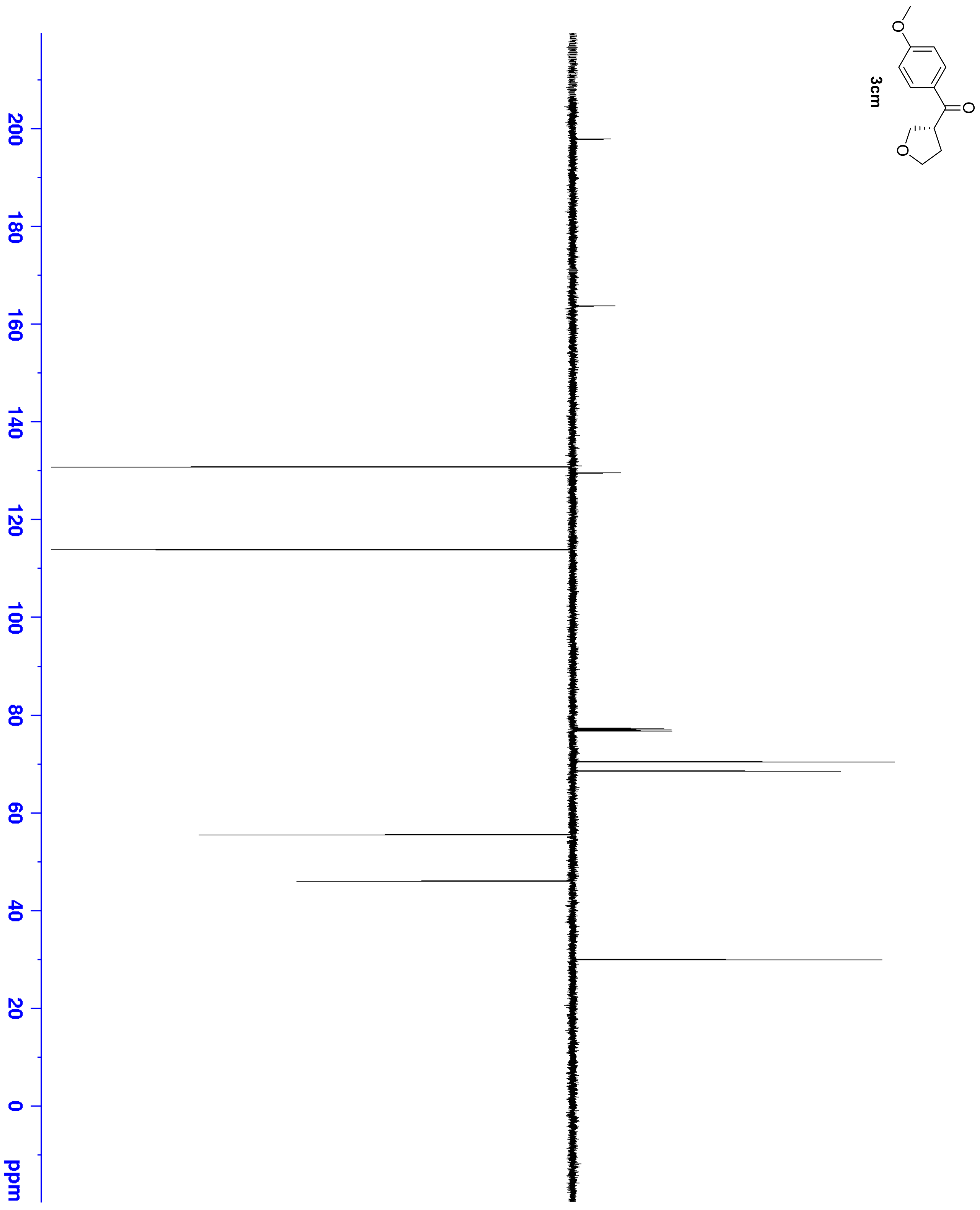
3cm
200
180
160
140
120
100
80
60
40
20
0
ppm

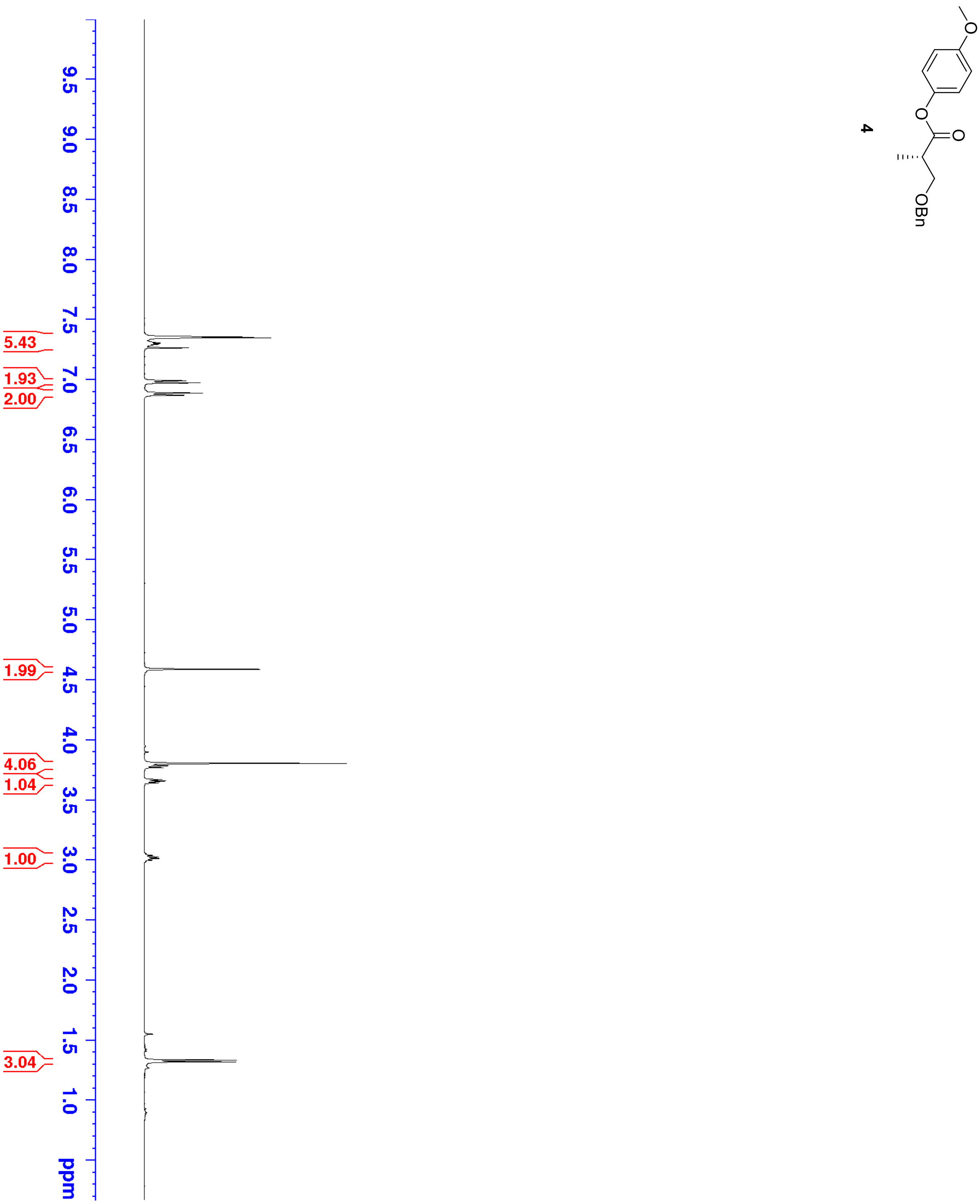
OBn
4
5.43
1.93
2.00
1.99
4.06
1.04
1.00
3.04
ppm

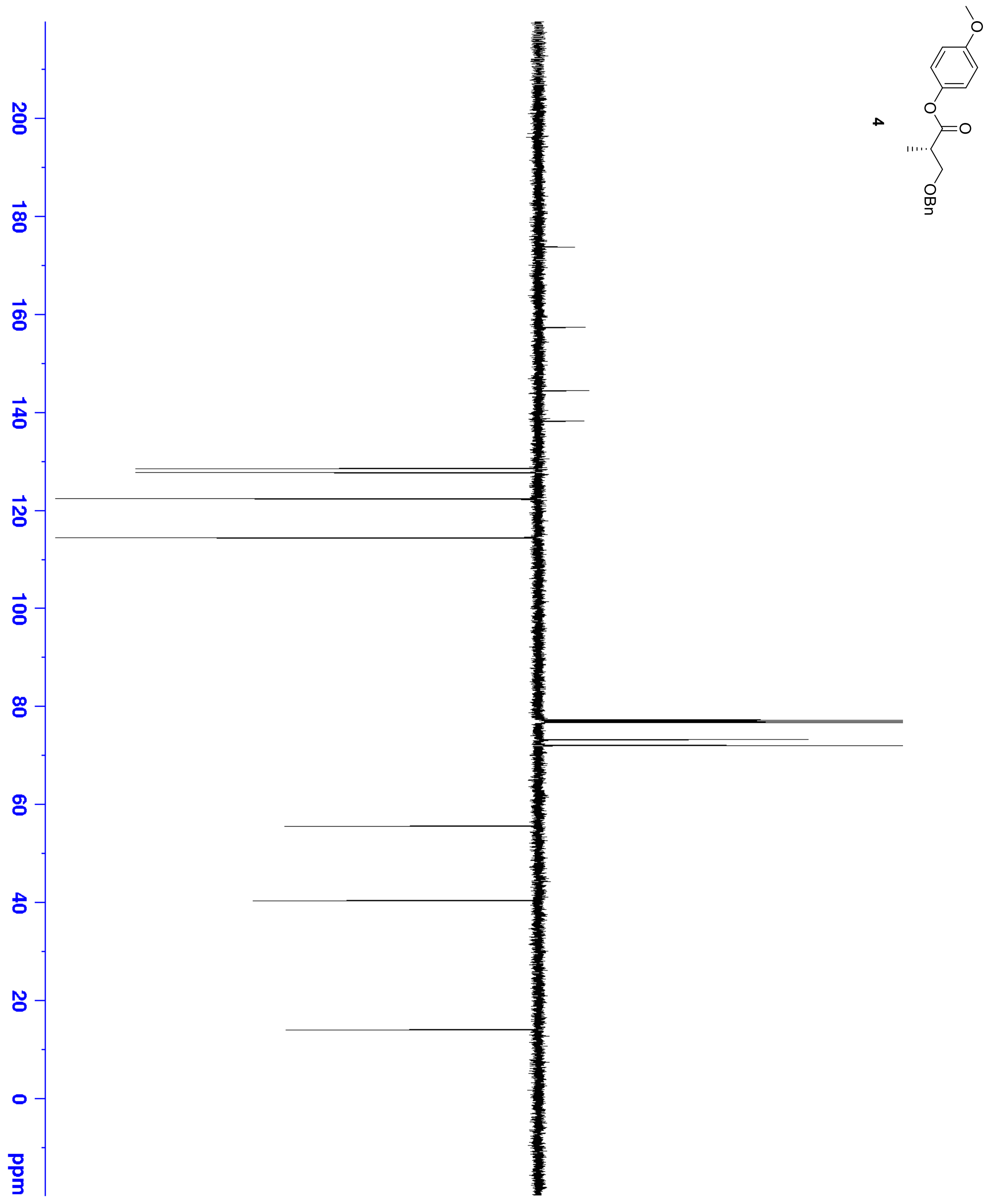

200
180
160
140
120
100
80
60
40
20
0
ppm
4
OBn

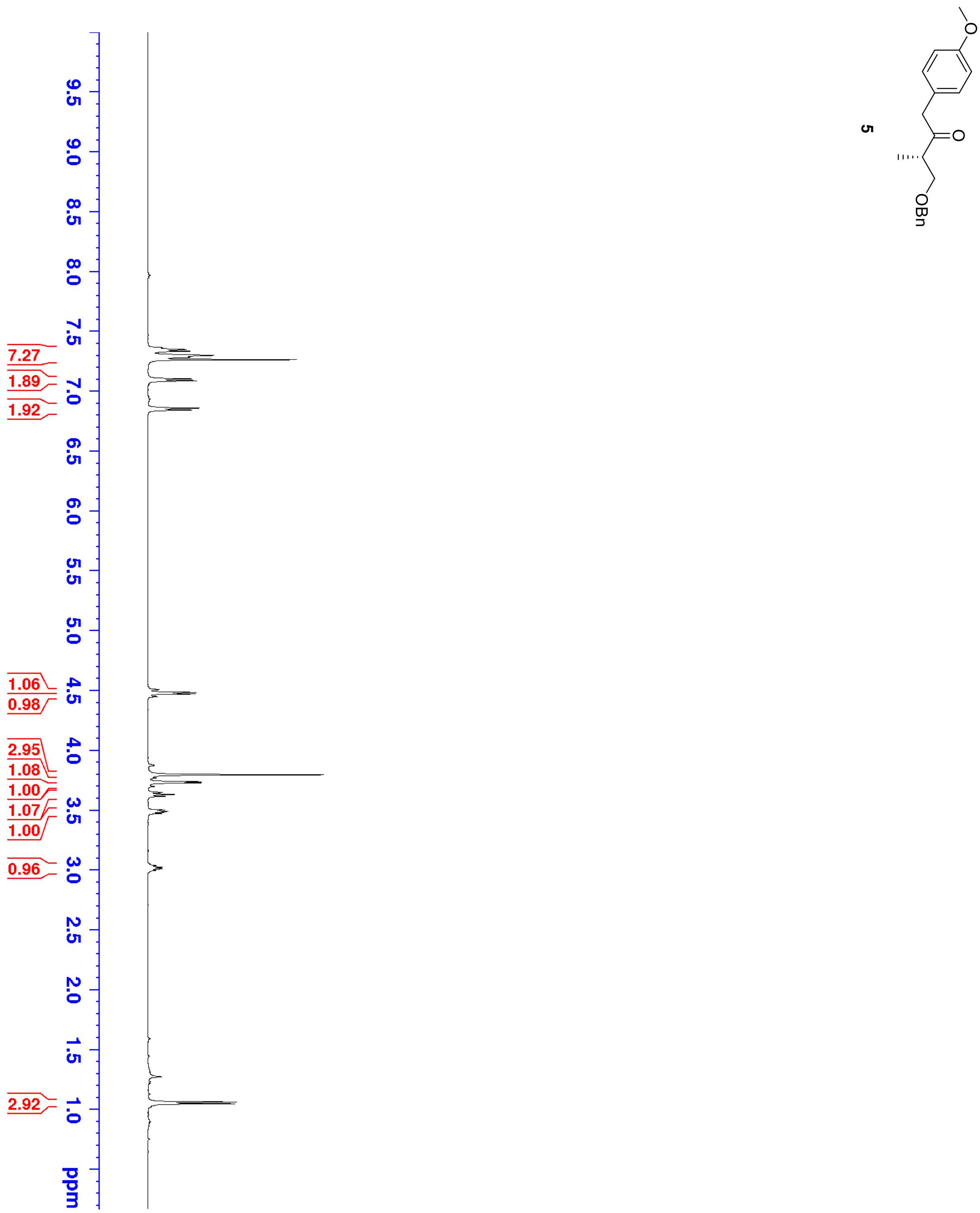

5
OBn
O
7.27
1.89
1.92
1.06
0.98
2.95
1.08
1.00
1.07
1.00
0.96
2.92
9.5
9.0
8.5
8.0
7.5
7.0
6.5
6.0
5.5
5.0
4.5
4.0
3.5
3.0
2.5
2.0
1.5
1.0
ppm

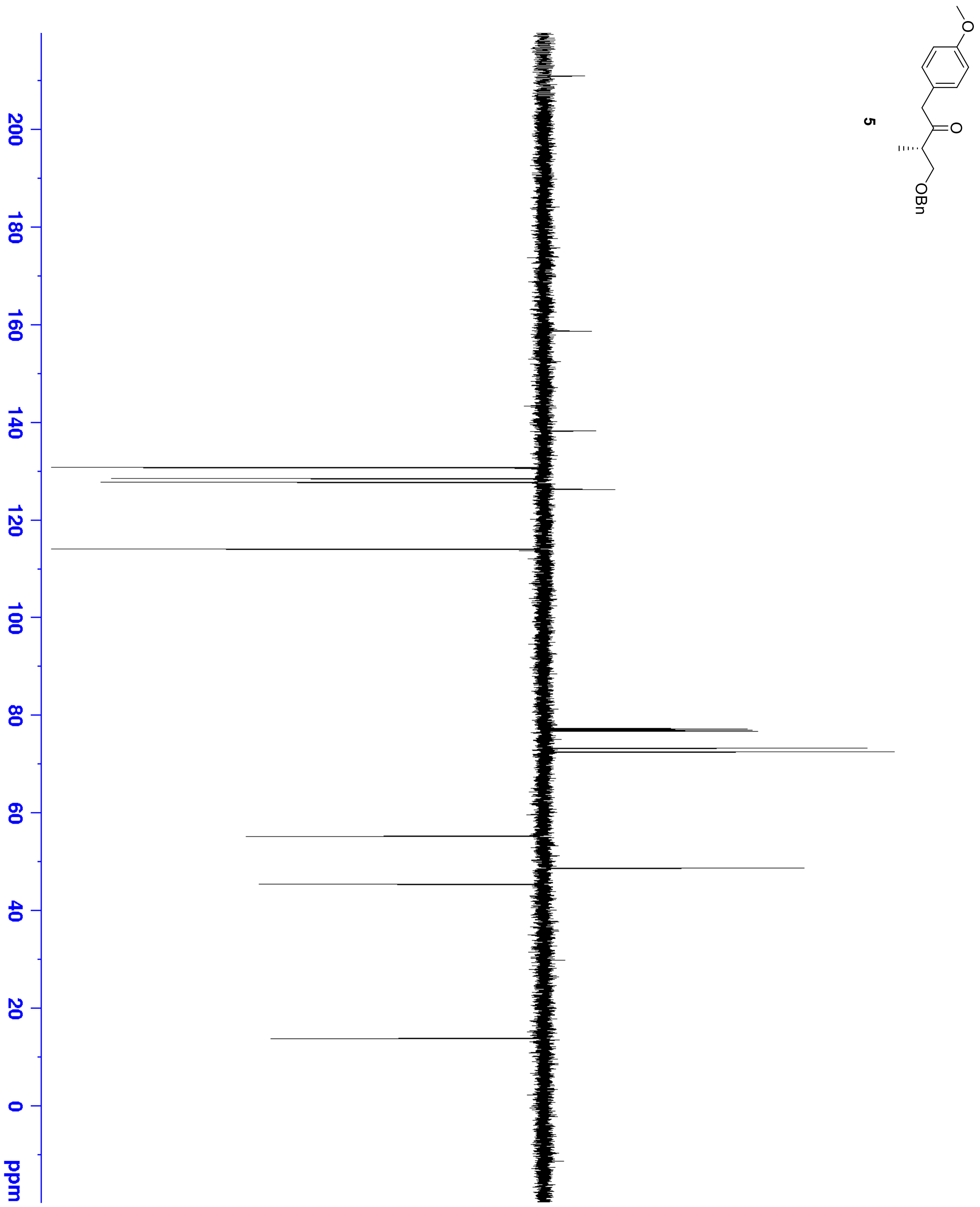
200
180
160
140
120
100
80
60
40
20
0
ppm
5
O
OBn

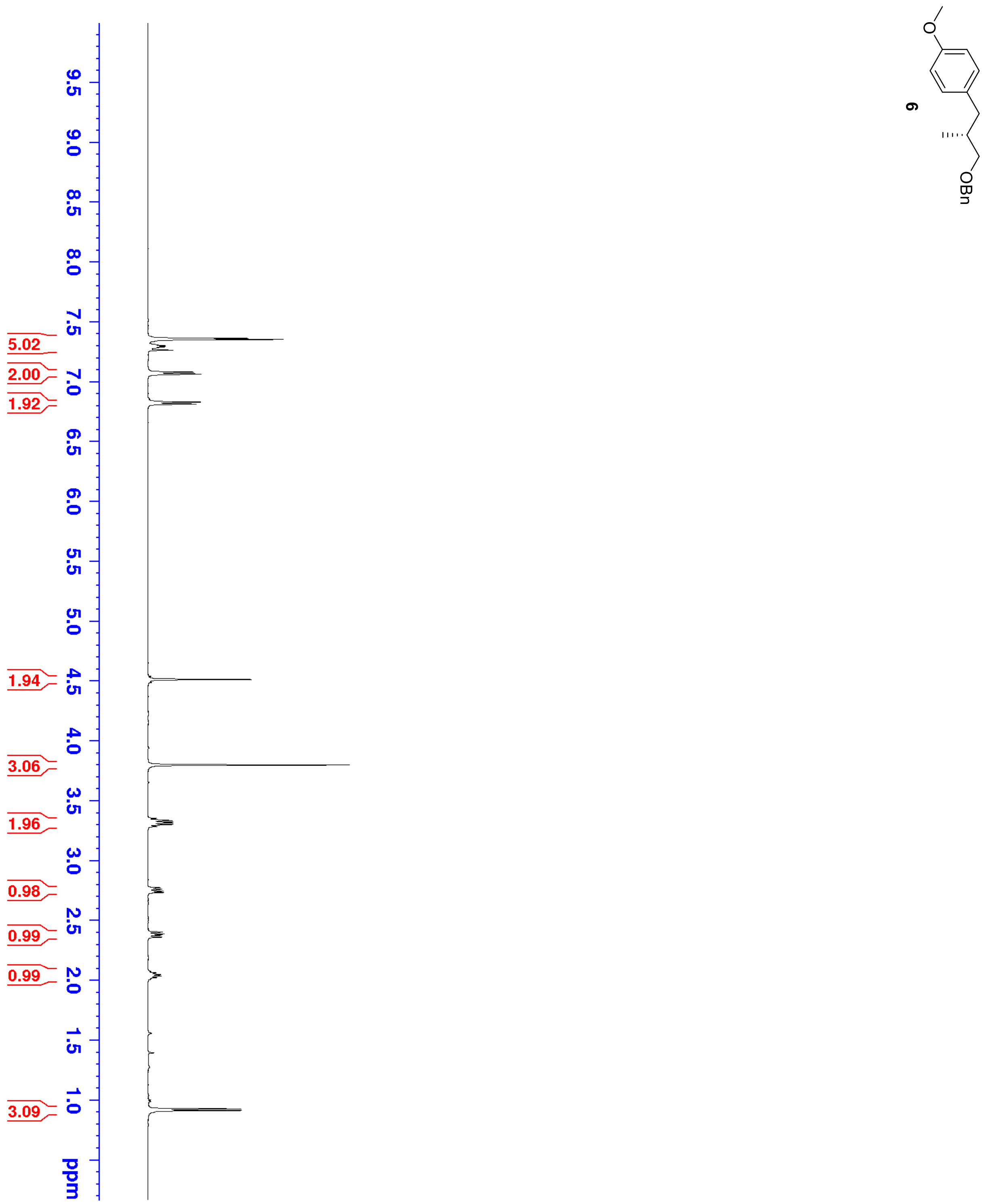
OBn
O
6
9.5
9.0
8.5
8.0
7.5
7.0
6.5
6.0
5.5
5.0
4.5
4.0
3.5
3.0
2.5
2.0
1.5
1.0
ppm
5.02
2.00
1.92
1.94
3.06
1.96
0.98
0.99
0.99
3.09

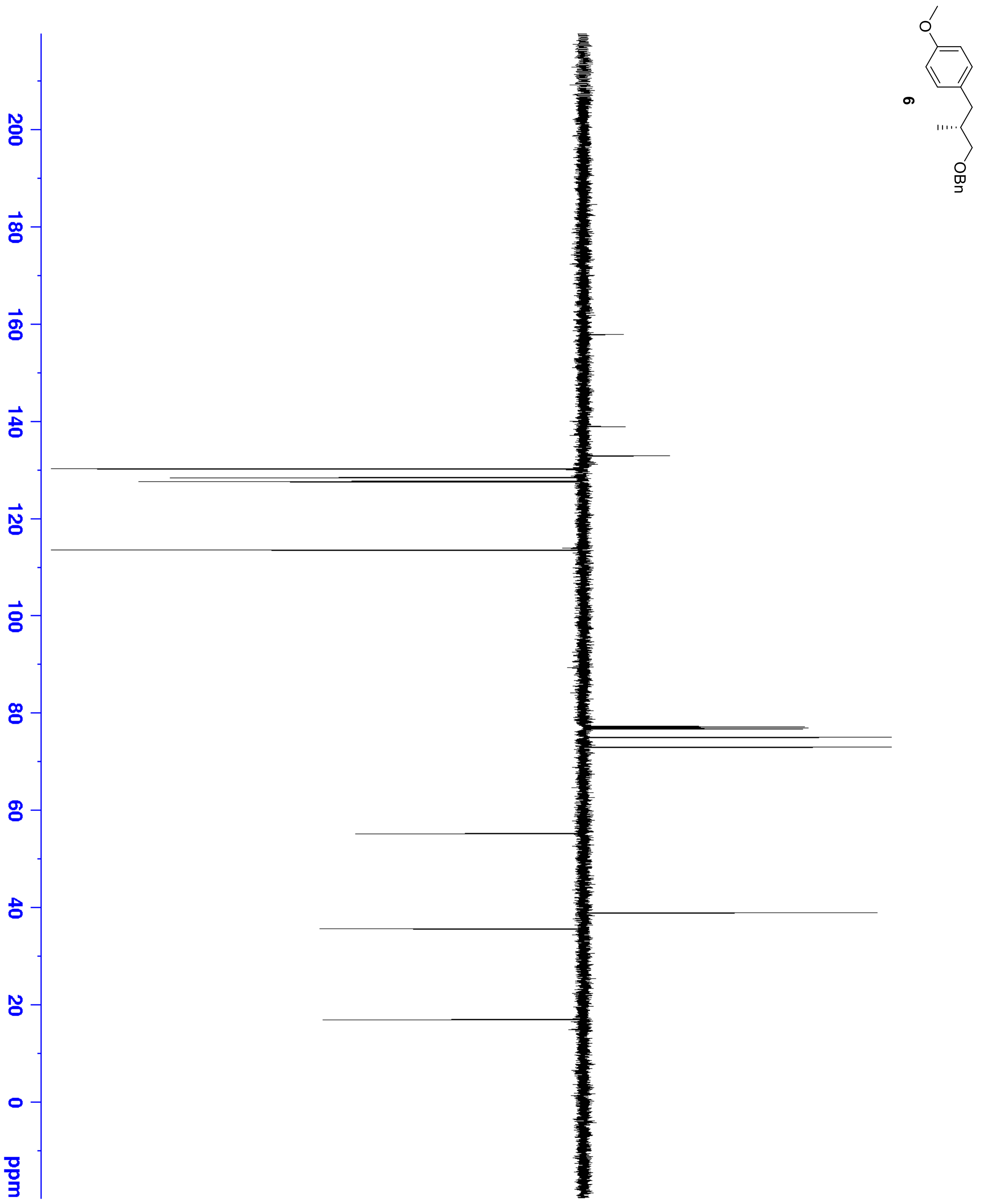

OBn
6
200
180
160
140
120
100
80
60
40
20
0
ppm

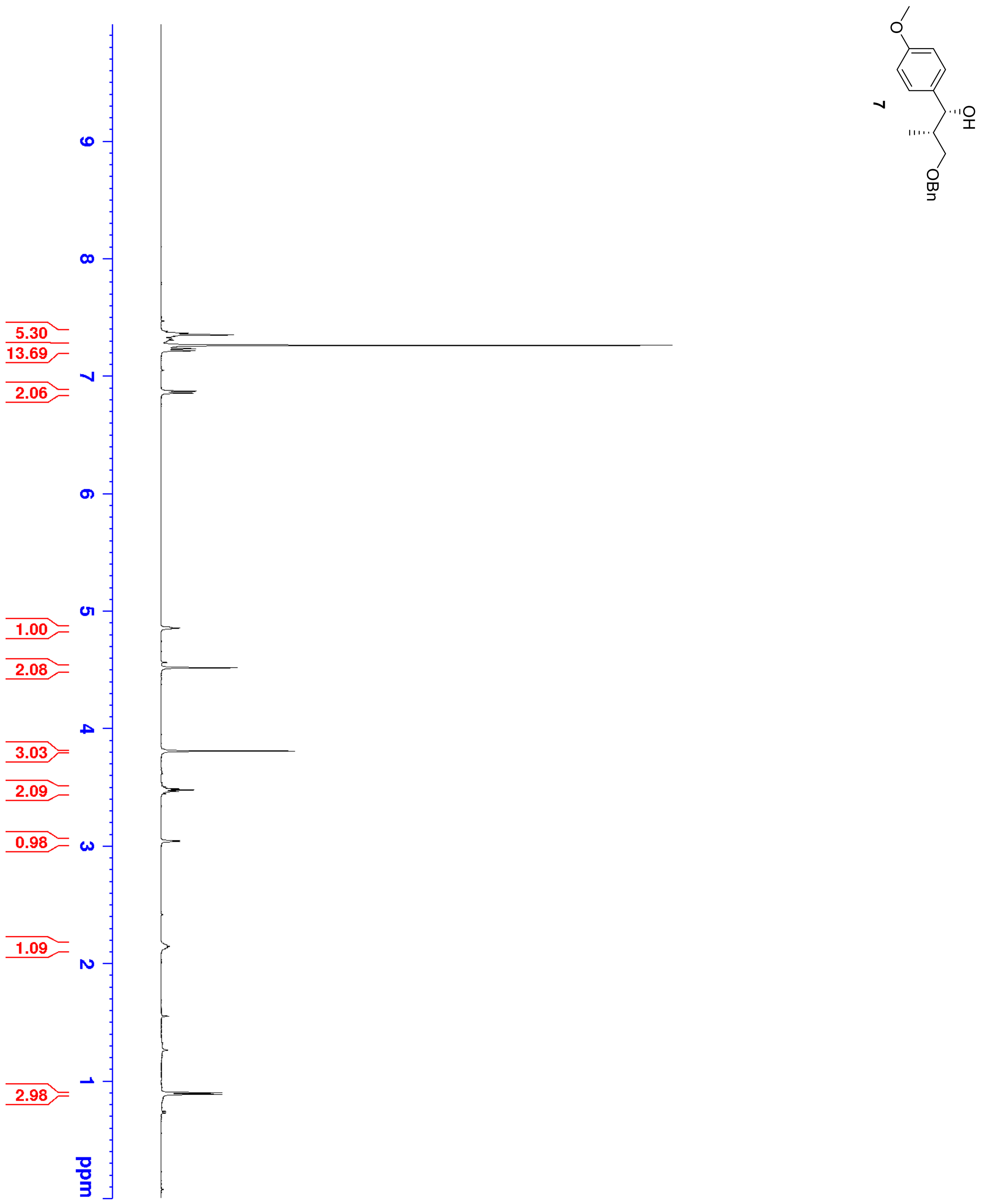

OH
OBn
7
5.30
13.69
2.06
1.00
2.08
3.03
2.09
0.98
1.09
2.98
9
8
7
6
5
4
3
2
1
ppm

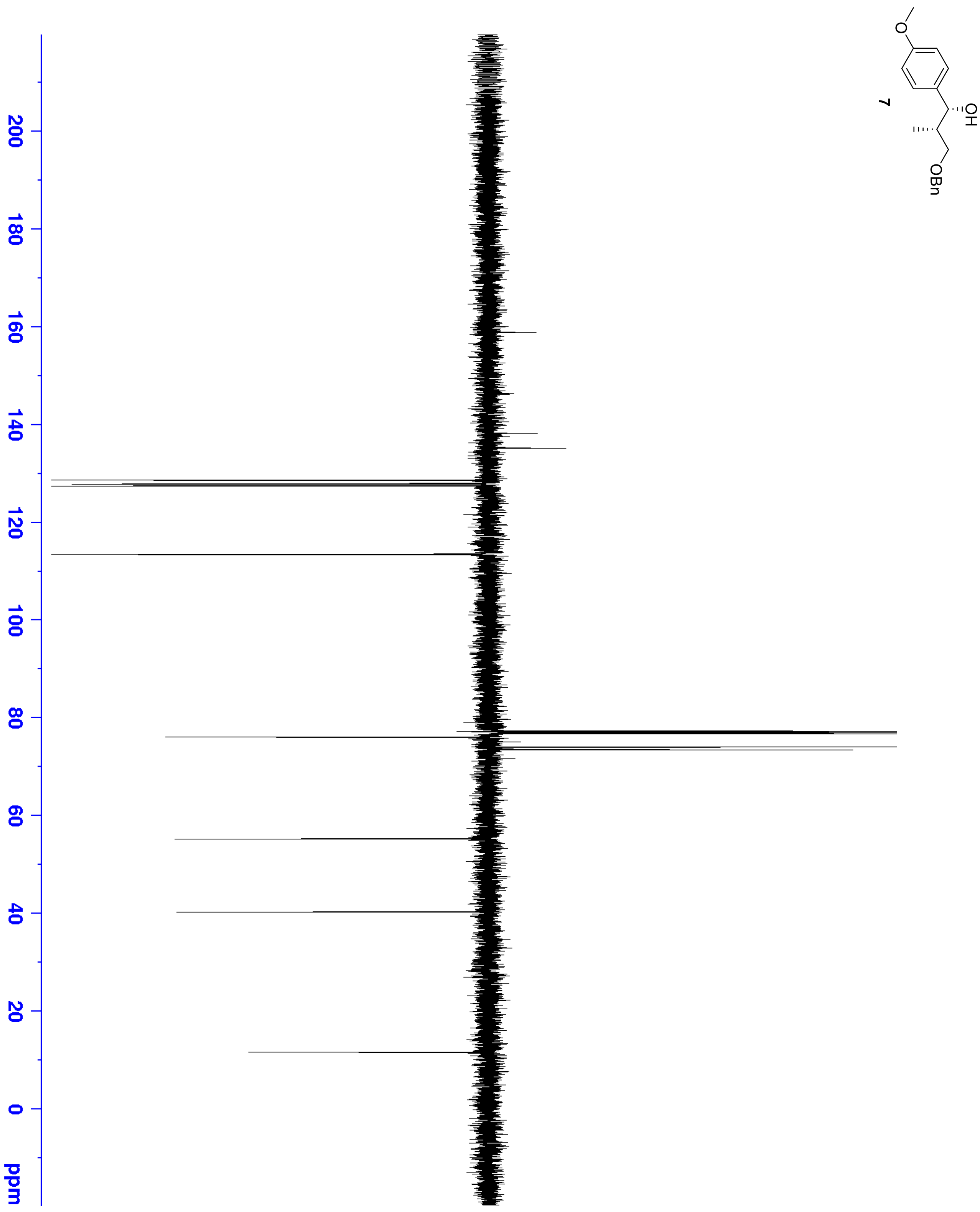
200
180
160
140
120
100
80
60
40
20
0
ppm
OH
OBn
7

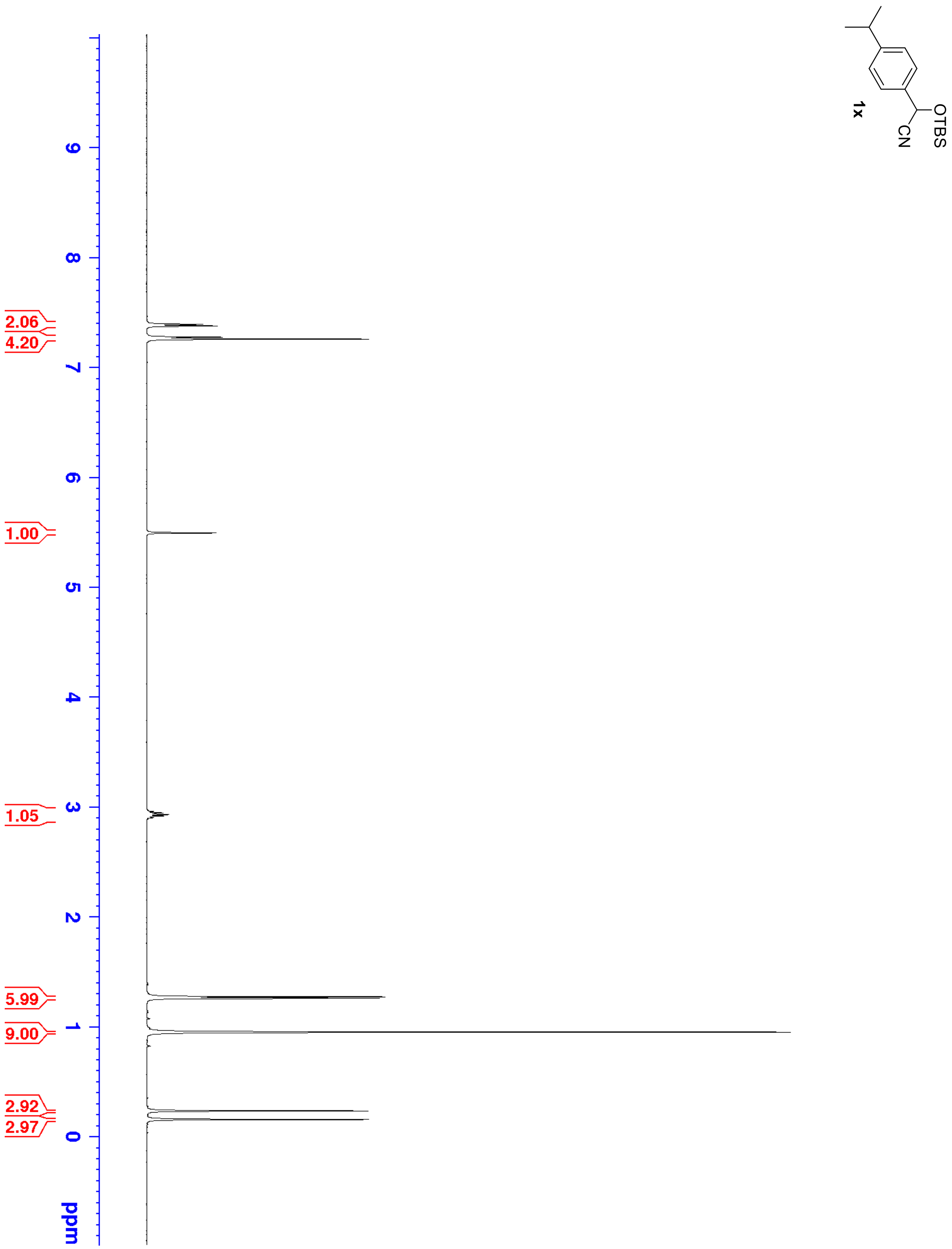
OTBS
CN
1x
2.06
4.20
1.00
1.05
5.99
9.00
2.92
2.97
9
8
7
6
5
4
3
2
1
0
ppm

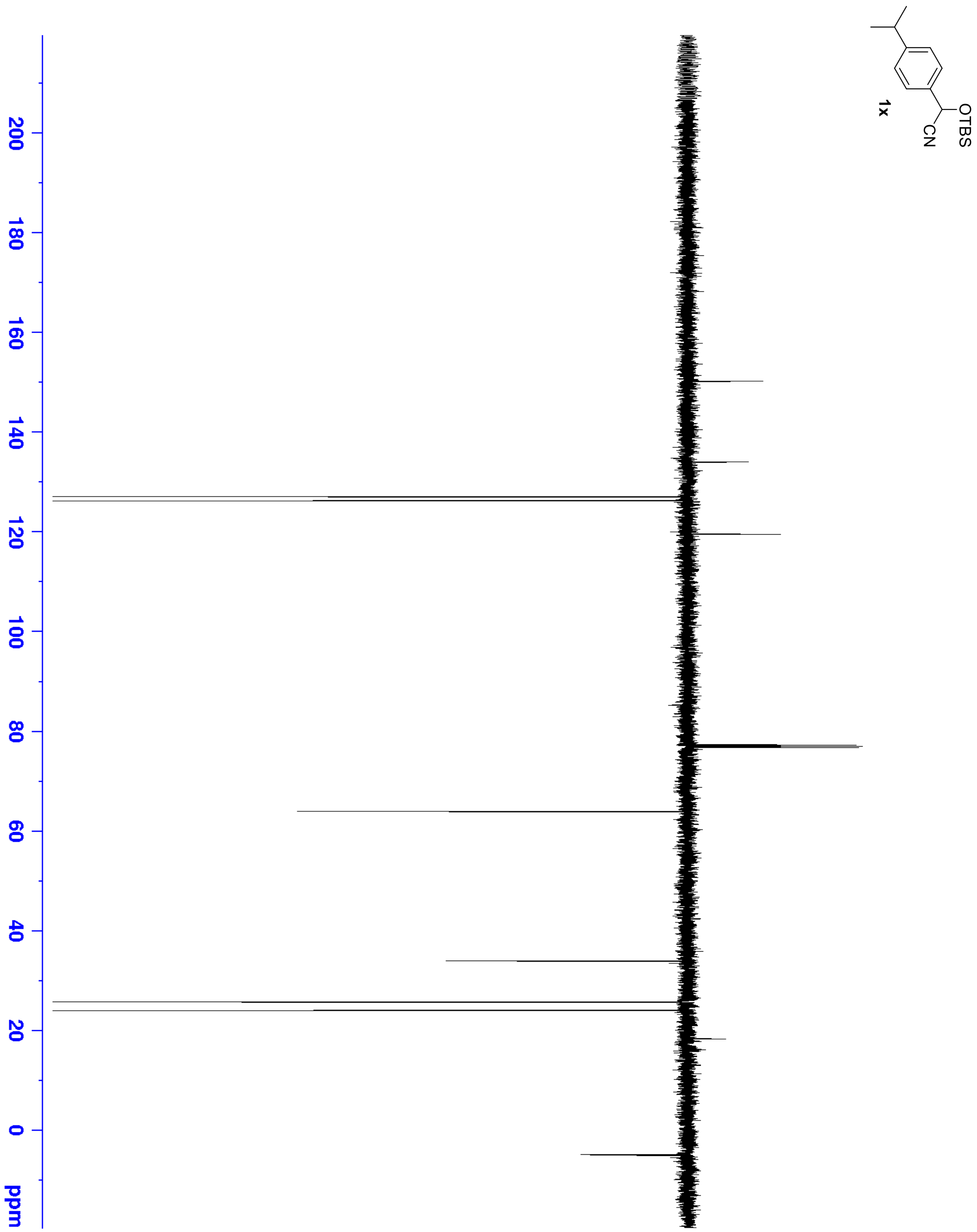
OTBS
CN
1x
200
180
160
140
120
100
80
60
40
20
0
ppm

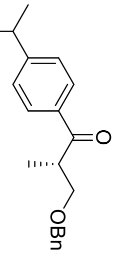
OBn

3xe

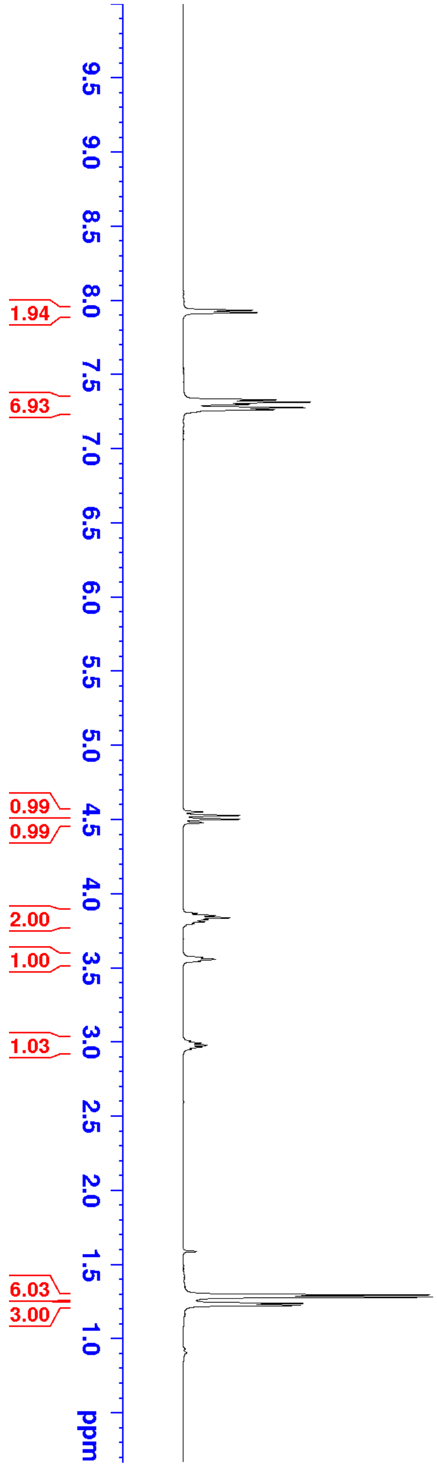
9.5
9.0
8.5
8.0
7.5
7.0
6.5
6.0
5.5
5.0
4.5
4.0
3.5
3.0
2.5
2.0
1.5
1.0
ppm
1.94
6.93
0.99
0.99
2.00
1.00
1.03
6.03
3.00

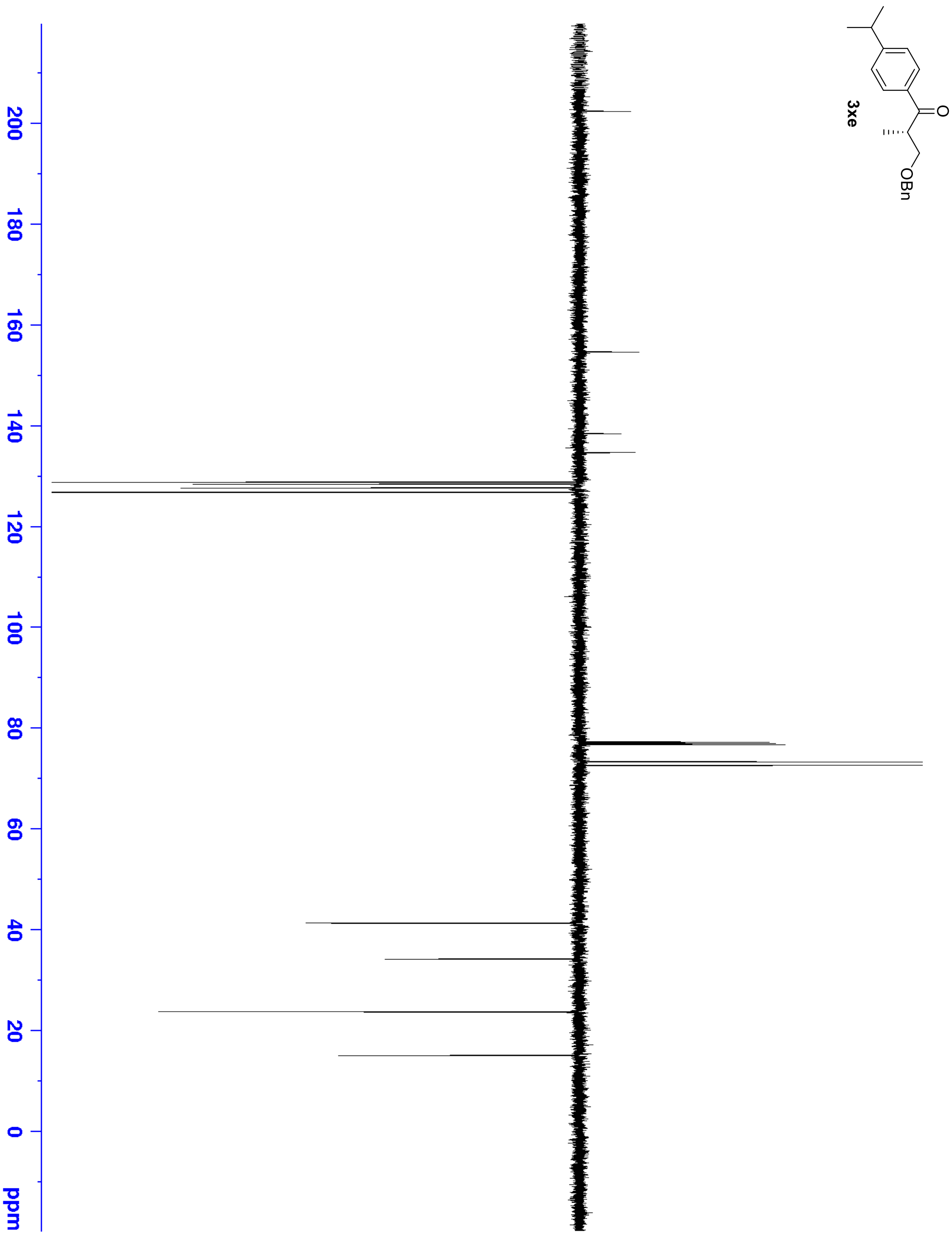

3xe
O
OBn
200
180
160
140
120
100
80
60
40
20
0
ppm

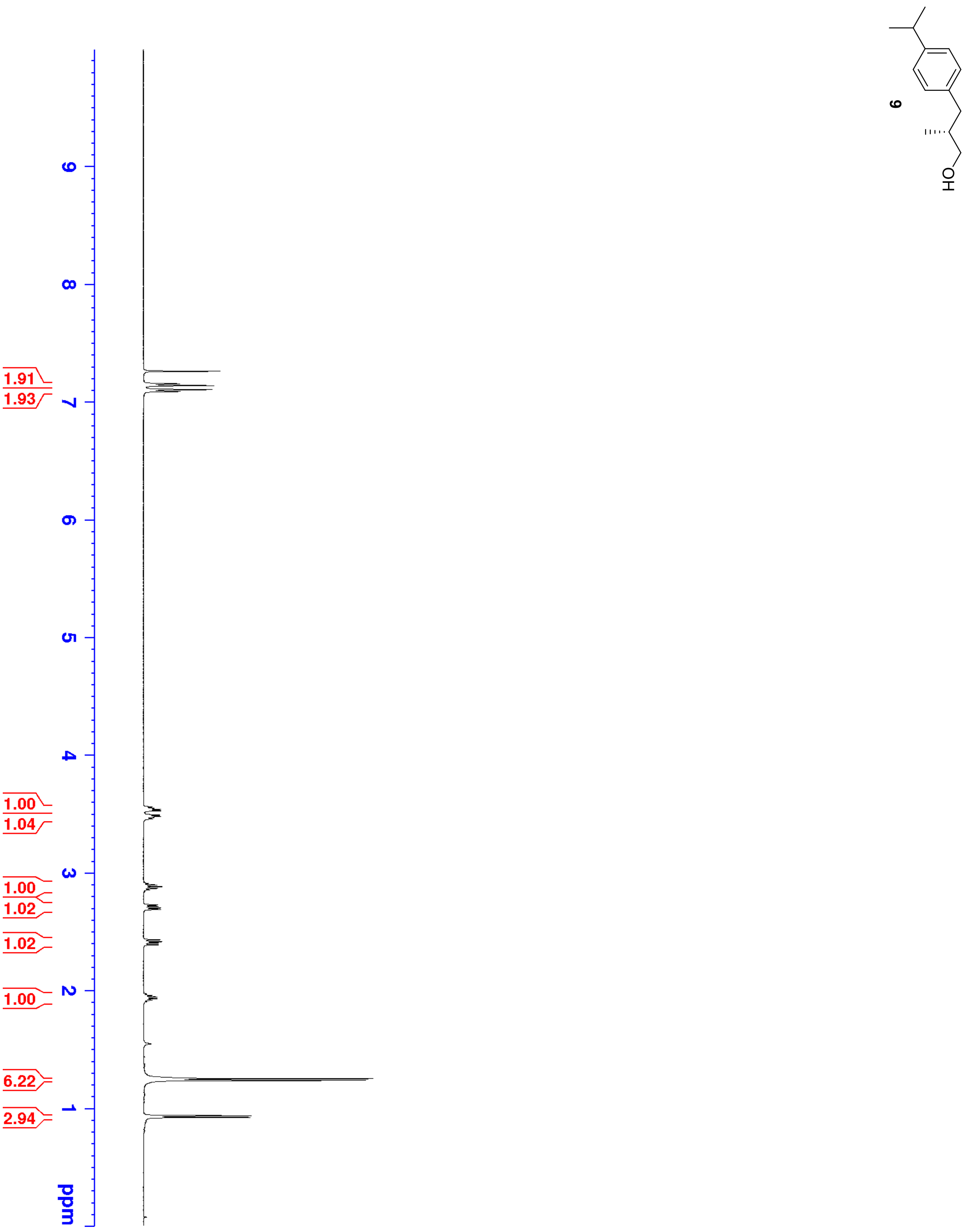
9
OH
1.91
1.93
1.00
1.04
1.00
1.02
1.02
1.00
6.22
2.94
9
8
7
6
5
4
3
2
1
ppm

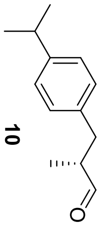

10

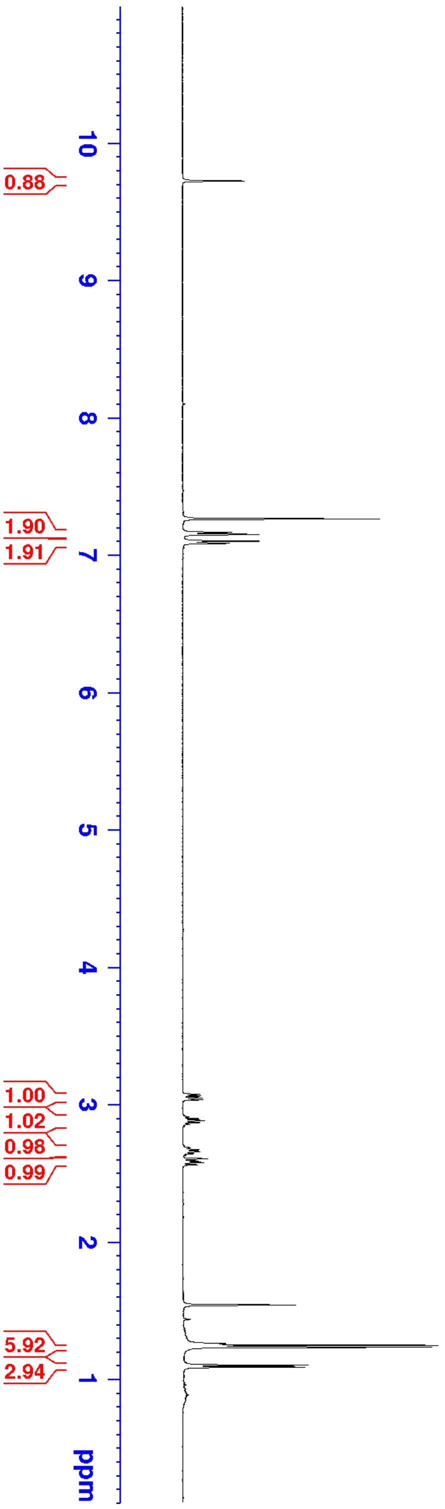

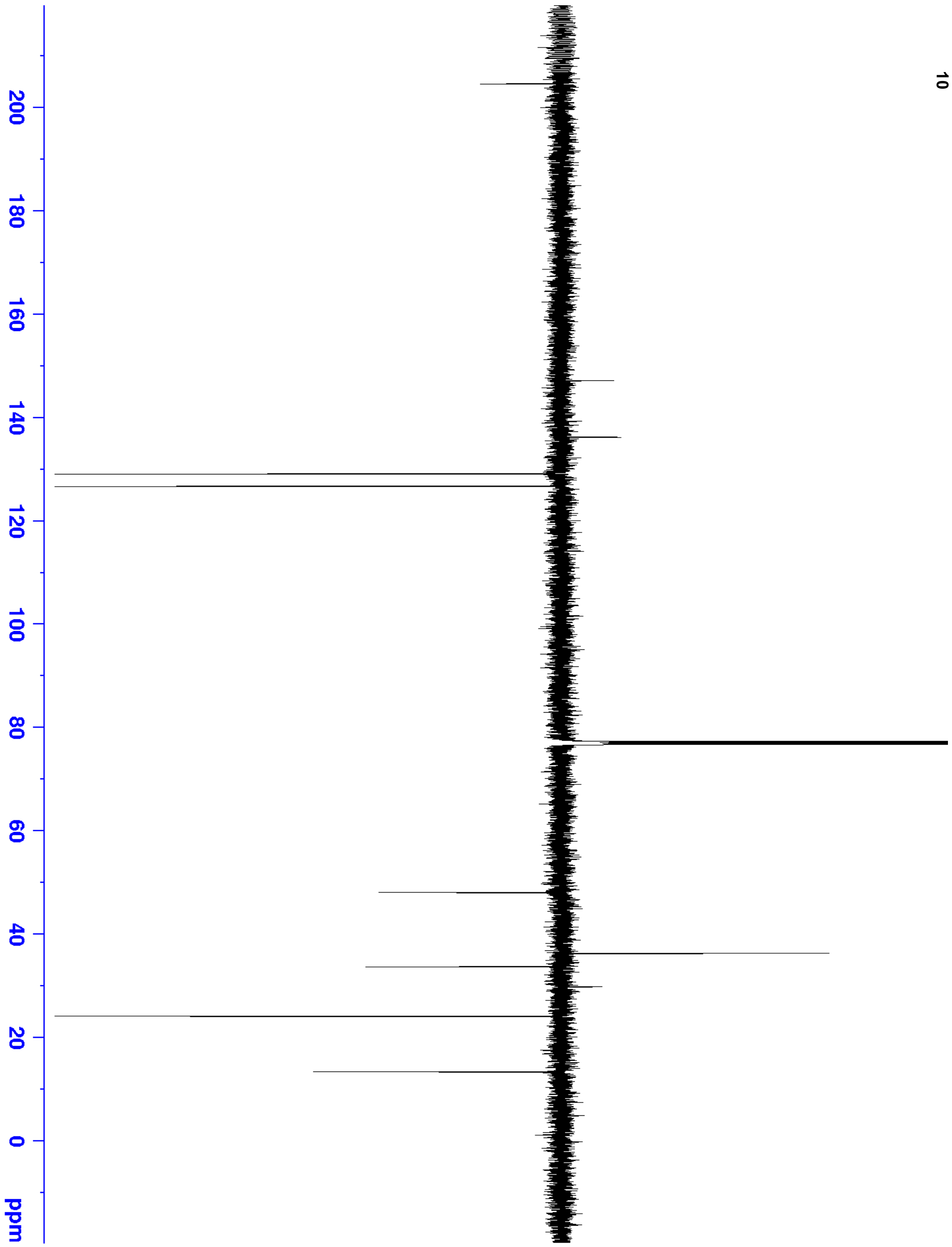

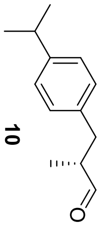

10

13. HPLC Chromatograms

2-Methyl-1,3-diphenylpropan-1-one (*rac*-3aa)

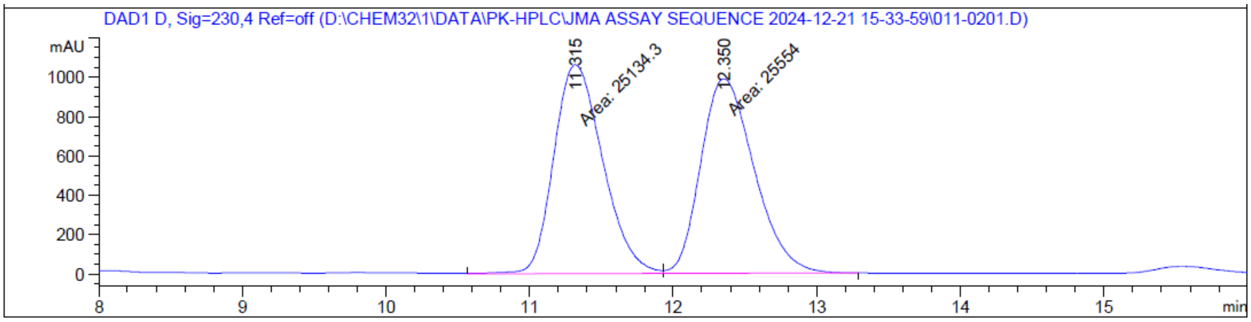

Peak #	RetTime [min]	Type	Width [min]	Area [mAU*s]	Height [mAU]	Area %
1	11.315	MF	0.3946	2.51343e4	1061.68982	49.5860
2	12.350	FM	0.4306	2.55540e4	989.03101	50.4140

(*S*)-2-Methyl-1,3-diphenylpropan-1-one (3aa)

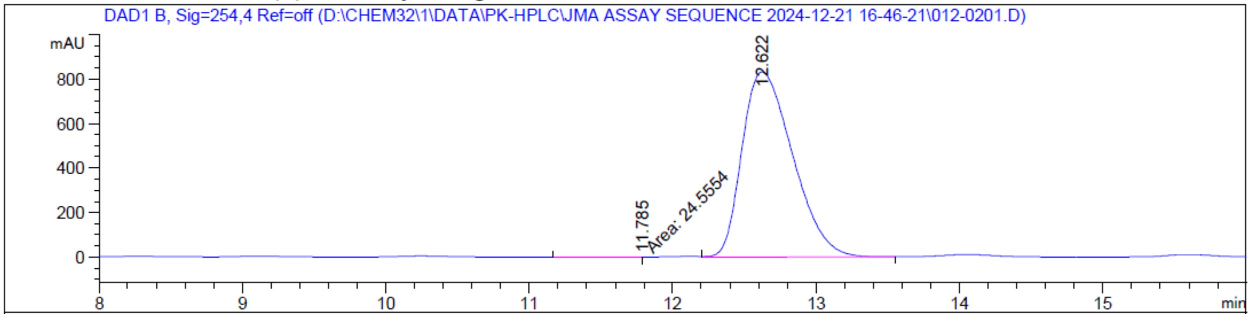

Peak #	RetTime [min]	Type	Width [min]	Area [mAU*s]	Height [mAU]	Area %
1	11.785	MM	0.5414	24.55535	7.55917e-1	0.1202
2	12.622	VB	0.3845	2.03972e4	830.70276	99.8798

1-(4-Bromophenyl)-2-methyl-3-phenylpropan-1-one (*rac*-3ba)

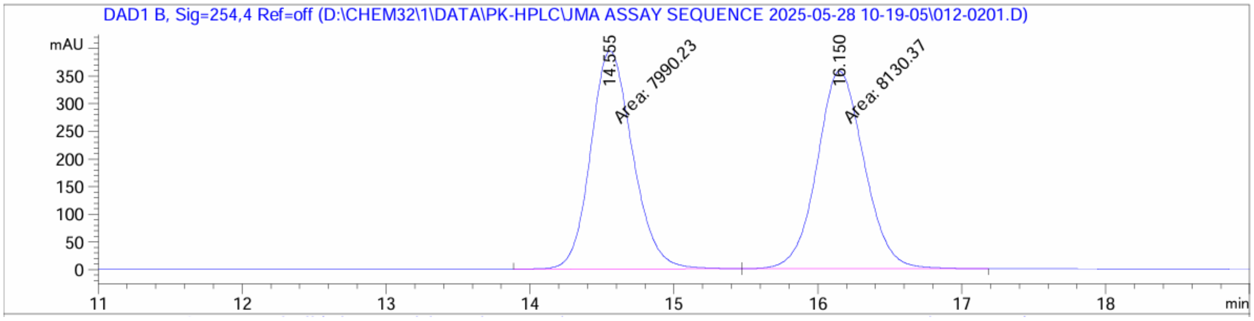

Peak #	RetTime [min]	Type	Width [min]	Area [mAU*s]	Height [mAU]	Area %
1	14.555	MM	0.3377	7990.23242	394.39737	49.5653
2	16.150	MM	0.3793	8130.37012	357.24023	50.4347

(*S*)-1-(4-Bromophenyl)-2-methyl-3-phenylpropan-1-one (3ba)

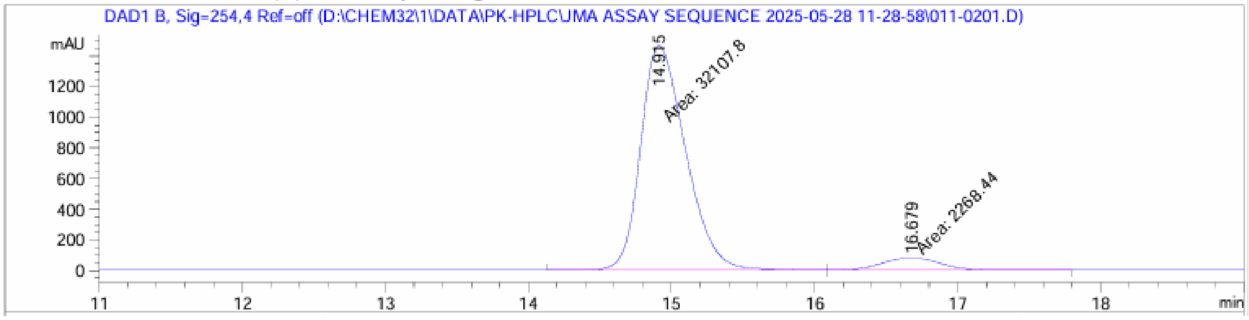

Peak #	RetTime [min]	Type	Width [min]	Area [mAU*s]	Height [mAU]	Area %
1	14.915	MM	0.3648	3.21078e4	1466.95398	93.4011
2	16.679	MM	0.4685	2268.43896	80.69793	6.5989

1-(4-Methoxyphenyl)-2-methyl-3-phenylpropan-1-one (*rac*-**3ca**)

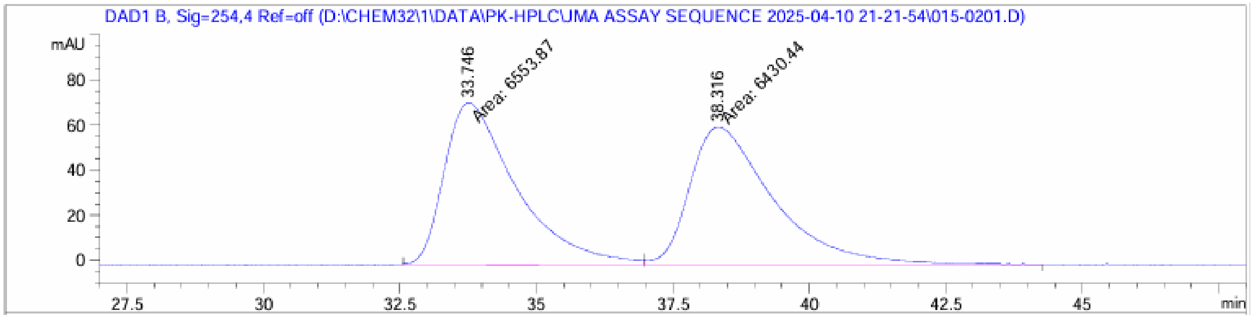

Peak #	RetTime [min]	Type	Width [min]	Area [mAU*s]	Height [mAU]	Area %
1	33.746	MF	1.5257	6553.86816	71.59261	50.4753
2	38.316	FM	1.7591	6430.44385	60.92545	49.5247

(*S*)-1-(4-Methoxyphenyl)-2-methyl-3-phenylpropan-1-one (**3ca**)

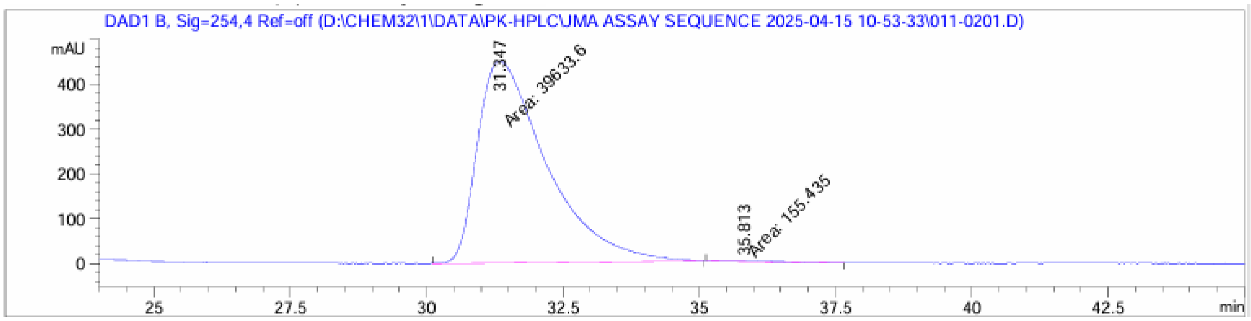

Peak #	RetTime [min]	Type	Width [min]	Area [mAU*s]	Height [mAU]	Area %
1	31.347	MM	1.4678	3.96336e4	450.02551	99.6094
2	35.813	MM	1.0756	155.43451	2.40858	0.3906

1-(4-Methoxyphenyl)-2-methylheptan-1-one (*rac*-**3cb**)

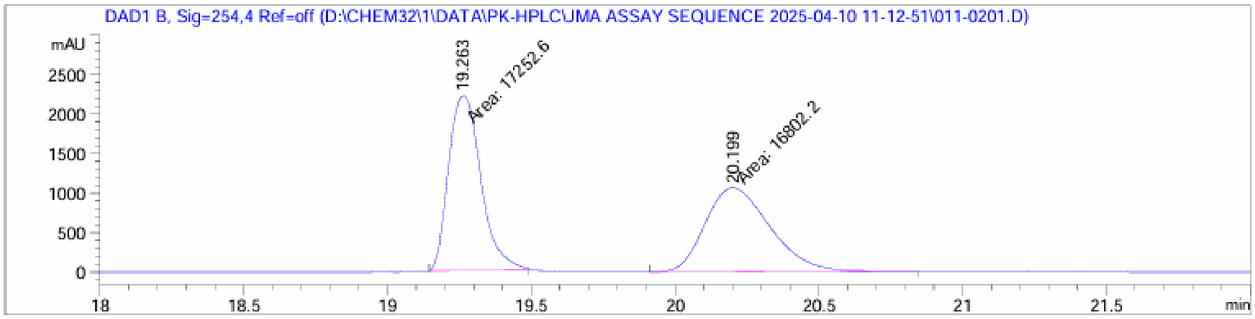

Peak #	RetTime [min]	Type	Width [min]	Area [mAU*s]	Height [mAU]	Area %
1	19.263	MM	0.1303	1.72526e4	2207.40503	50.6614
2	20.199	MM	0.2629	1.68022e4	1065.29602	49.3386

(*S*)- 1-(4-Methoxyphenyl)-2-methylheptan-1-one (**3cb**)

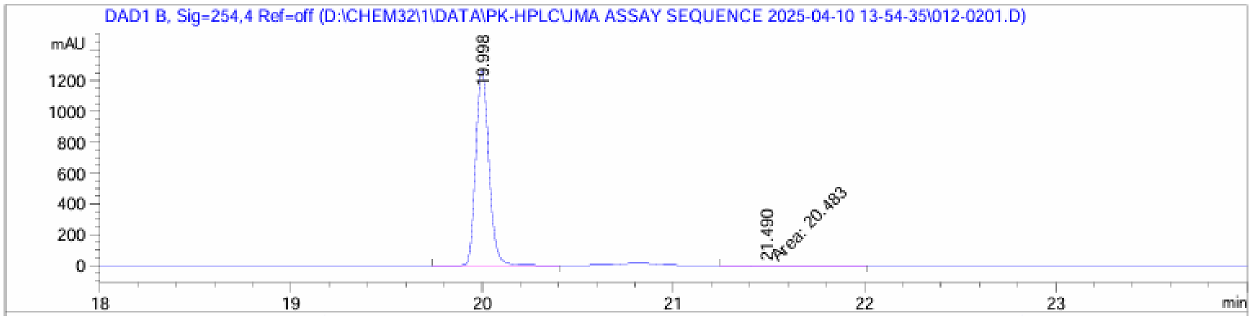

Peak #	RetTime [min]	Type	Width [min]	Area [mAU*s]	Height [mAU]	Area %
1	19.998	BB	0.0758	6064.15723	1268.68372	99.6634
2	21.490	MM	0.3741	20.48300	9.12508e-1	0.3366

1-(4-Methoxyphenyl)-2-methylpent-4-en-1-one (*rac*-3cc)

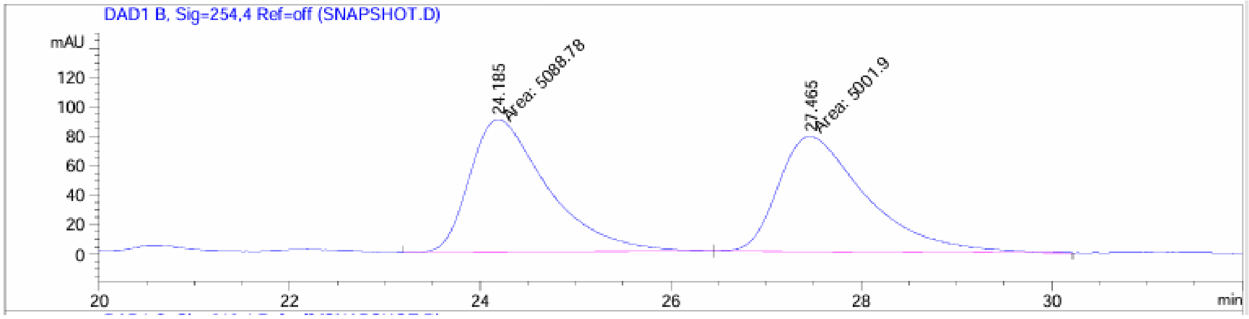

Peak #	RetTime [min]	Type	Width [min]	Area [mAU*s]	Height [mAU]	Area %
1	24.185	MM	0.9423	5088.77539	90.00413	50.4305
2	27.465	MM	1.0667	5001.89697	78.15236	49.5695

(*S*)-1-(4-Methoxyphenyl)-2-methylpent-4-en-1-one (3cc)

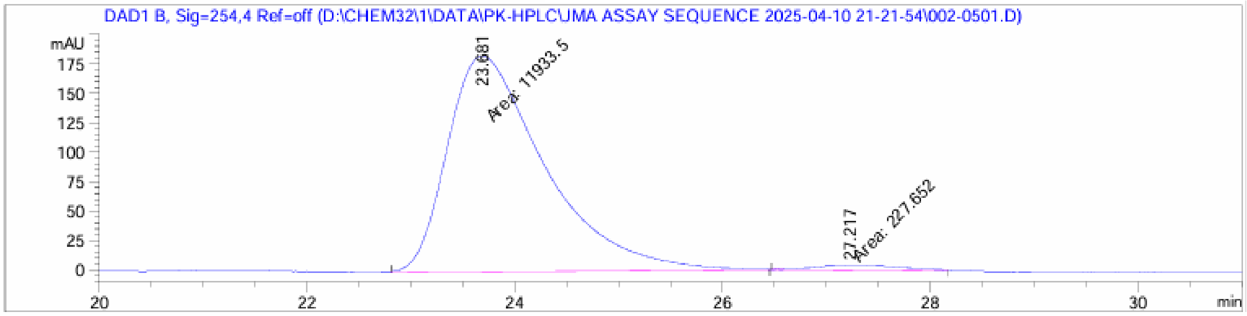

Peak #	RetTime [min]	Type	Width [min]	Area [mAU*s]	Height [mAU]	Area %
1	23.681	MM	1.0881	1.19335e4	182.79303	98.1280
2	27.217	MM	0.8935	227.65224	4.24668	1.8720

1-(4-Methoxyphenyl)-2-methylpent-4-yn-1-one (*rac*-**3cd**)

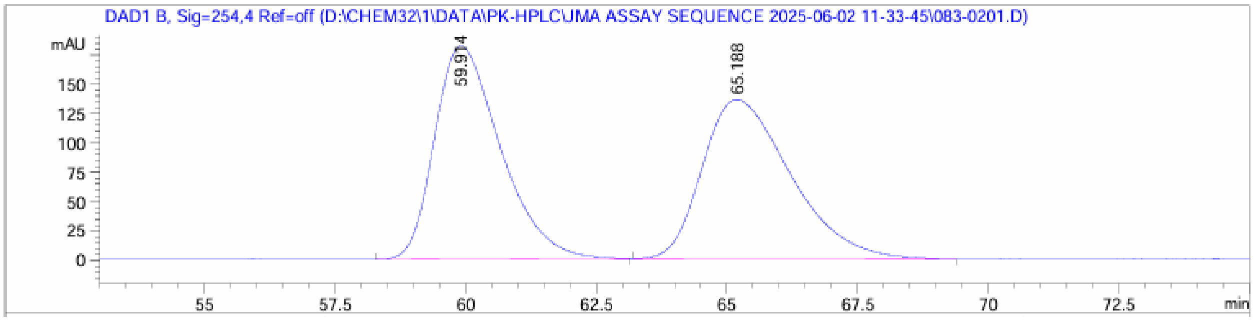

Peak #	RetTime [min]	Type	Width [min]	Area [mAU*s]	Height [mAU]	Area %
1	59.914	BB	1.2876	1.59250e4	181.51114	50.0196
2	65.188	BB	1.6443	1.59126e4	135.67683	49.9804

(*S*)-1-(4-Methoxyphenyl)-2-methylpent-4-yn-1-one (**3cd**)

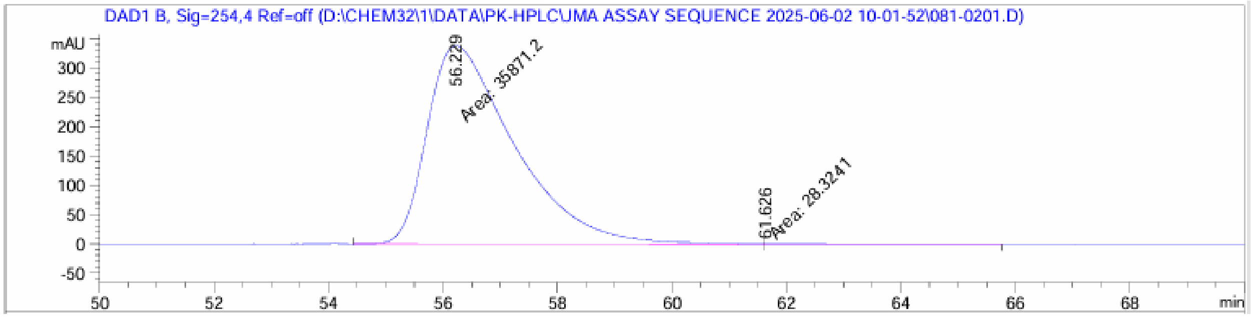

Peak #	RetTime [min]	Type	Width [min]	Area [mAU*s]	Height [mAU]	Area %
1	56.229	MF	1.7630	3.58712e4	339.11398	99.9211
2	61.626	FM	0.8200	28.32407	5.75665e-1	0.0789

3-(Benzyloxy)-1-(4-methoxyphenyl)-2-methylpropan-1-one (*rac*-3ce)

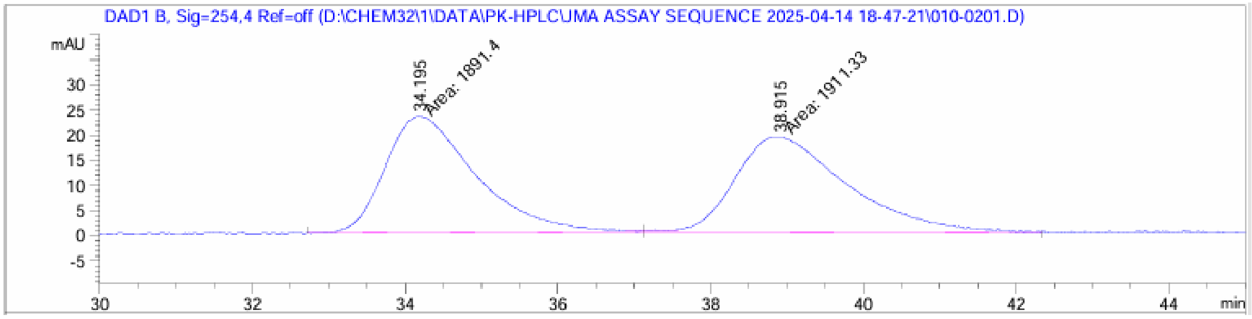

Peak #	RetTime [min]	Type	Width [min]	Area [mAU*s]	Height [mAU]	Area %
1	34.195	MM	1.3607	1891.39832	23.16702	49.7380
2	38.915	MM	1.6882	1911.32715	18.86940	50.2620

(*S*)-3-(Benzyloxy)-1-(4-methoxyphenyl)-2-methylpropan-1-one (3ce)

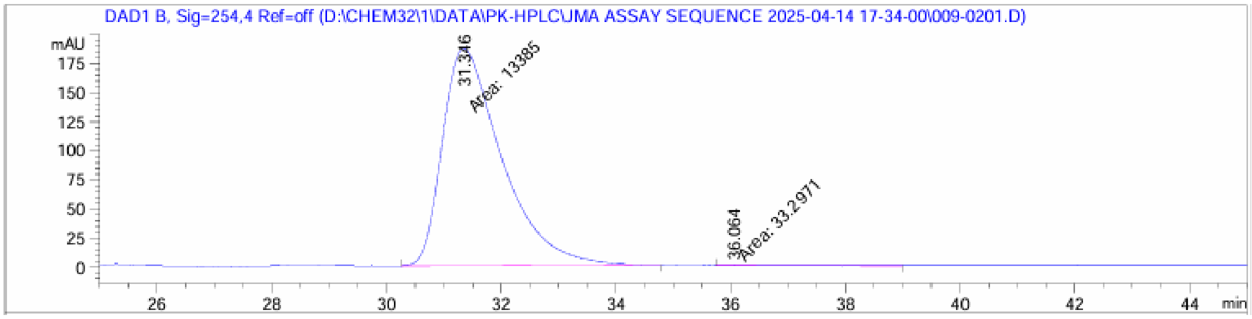

Peak #	RetTime [min]	Type	Width [min]	Area [mAU*s]	Height [mAU]	Area %
1	31.346	MM	1.1955	1.33850e4	186.60411	99.7519
2	36.064	MM	1.3174	33.29705	4.21260e-1	0.2481

2-((Benzyloxy)methyl)-1-(4-methoxyphenyl)butan-1-one (*rac*-3cf)

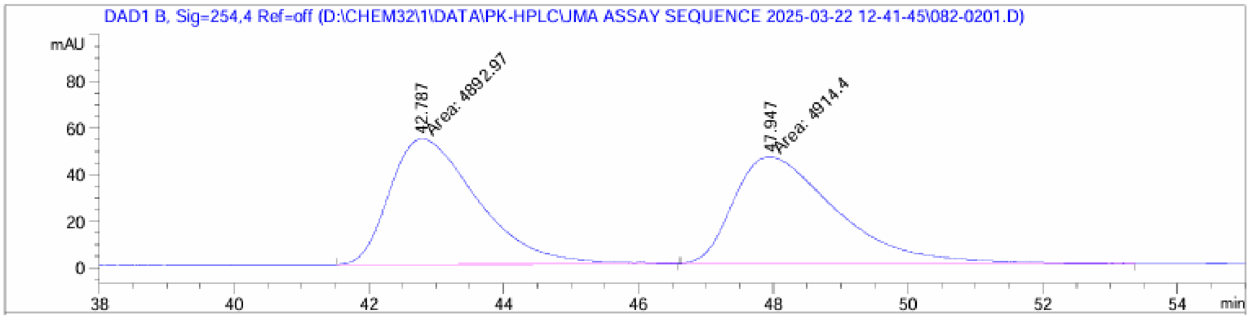

Peak #	RetTime [min]	Type	Width [min]	Area [mAU*s]	Height [mAU]	Area %
1	42.787	MM	1.5135	4892.96680	53.87965	49.8907
2	47.947	MM	1.7856	4914.39746	45.87109	50.1093

(*S*)-2-((Benzyloxy)methyl)-1-(4-methoxyphenyl)butan-1-one (3cf)

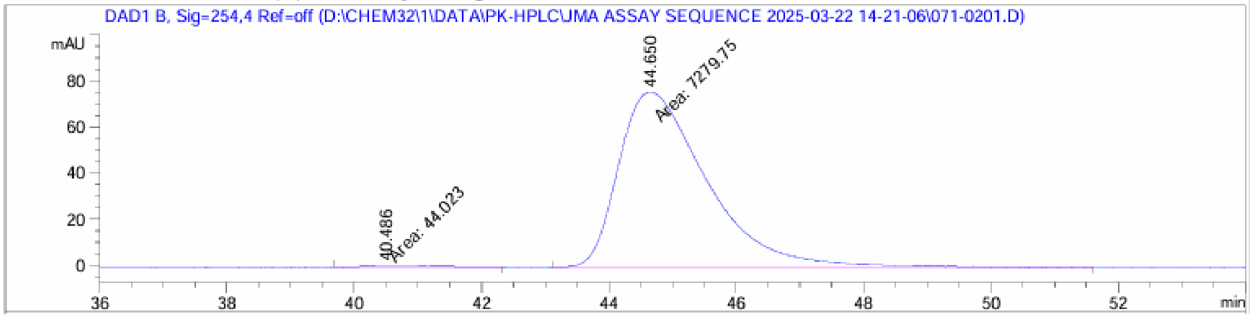

Peak #	RetTime [min]	Type	Width [min]	Area [mAU*s]	Height [mAU]	Area %
1	40.486	MM	1.1394	44.02296	6.43960e-1	0.6011
2	44.650	MM	1.5983	7279.74707	75.90912	99.3989

2-((Benzyloxy)methyl)-1-(4-methoxyphenyl)-3-methylbutan-1-one (*rac*-3cg)

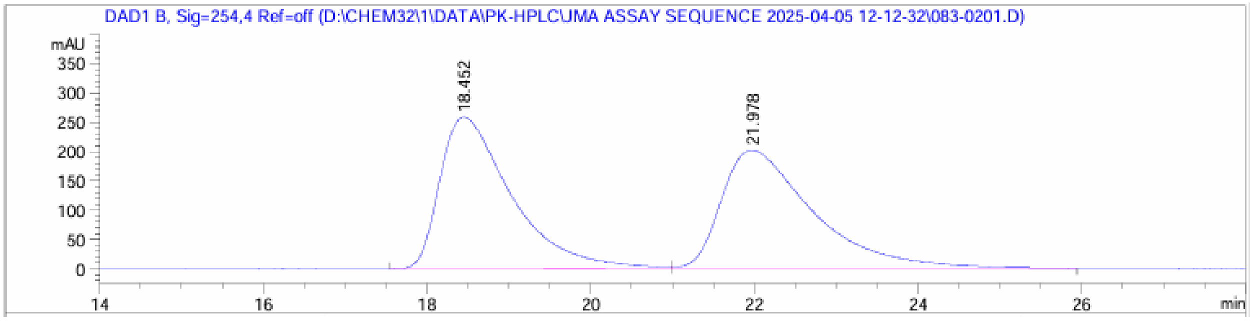

Peak #	RetTime [min]	Type	Width [min]	Area [mAU*s]	Height [mAU]	Area %
1	18.452	BV	0.8692	1.57682e4	259.38458	50.0258
2	21.978	VB	1.0393	1.57519e4	202.55374	49.9742

(*S*)-2-((Benzyloxy)methyl)-1-(4-methoxyphenyl)-3-methylbutan-1-one (3cg)

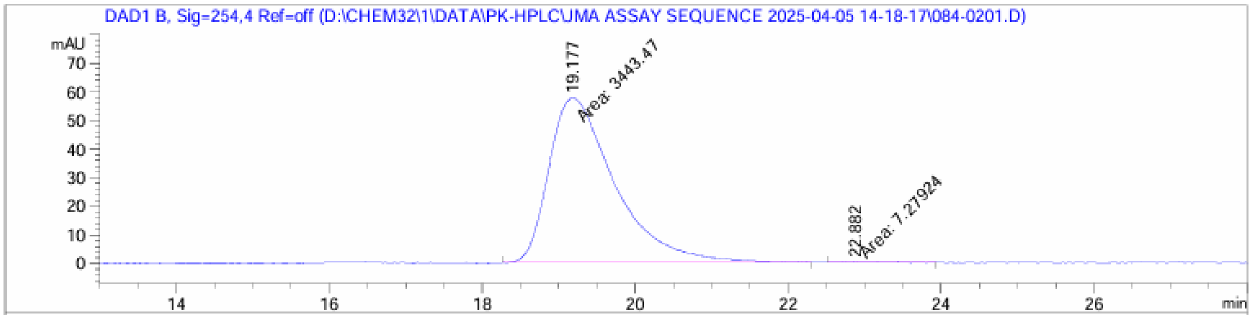

Peak #	RetTime [min]	Type	Width [min]	Area [mAU*s]	Height [mAU]	Area %
1	19.177	MM	0.9981	3443.47266	57.49857	99.7891
2	22.882	MM	0.5085	7.27924	2.38564e-1	0.2109

3-(Benzyloxy)-2-(cyclopentylmethyl)-1-(4-methoxyphenyl)propan-1-one (*rac*-3ch)

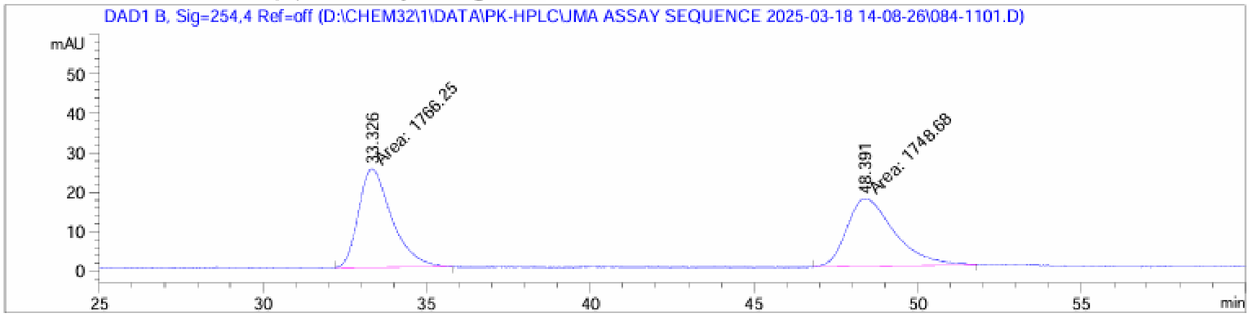

Peak #	RetTime [min]	Type	Width [min]	Area [mAU*s]	Height [mAU]	Area %
1	33.326	MM	1.1797	1766.24658	24.95271	50.2498
2	48.391	MM	1.6995	1748.68396	17.14904	49.7502

(*S*)-3-(Benzyloxy)-2-(cyclopentylmethyl)-1-(4-methoxyphenyl)propan-1-one (3ch)

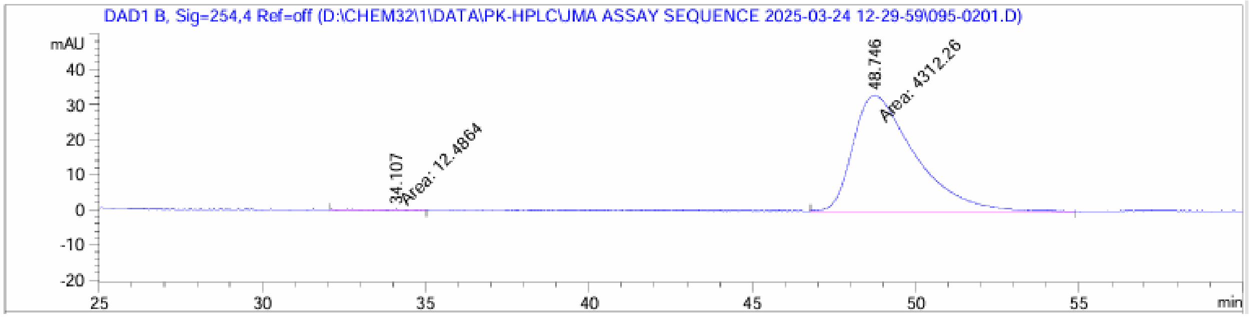

Peak #	RetTime [min]	Type	Width [min]	Area [mAU*s]	Height [mAU]	Area %
1	34.107	MM	0.8097	12.48642	2.57025e-1	0.2887
2	48.746	MM	2.1784	4312.25635	32.99320	99.7113

2-((Benzyloxy)methyl)-1-(4-methoxyphenyl)-4-methylpent-4-en-1-one (*rac*-**3ci**)

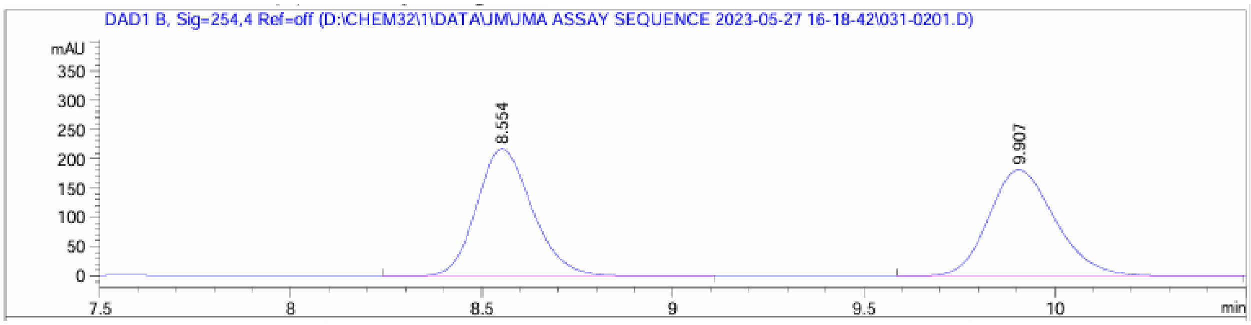

Peak #	RetTime [min]	Type	Width [min]	Area [mAU*s]	Height [mAU]	Area %
1	8.554	BB	0.1553	2180.57910	216.90115	50.0874
2	9.907	BB	0.1861	2172.97266	180.97580	49.9126

(*S*)-2-((Benzyloxy)methyl)-1-(4-methoxyphenyl)-4-methylpent-4-en-1-one (**3ci**)

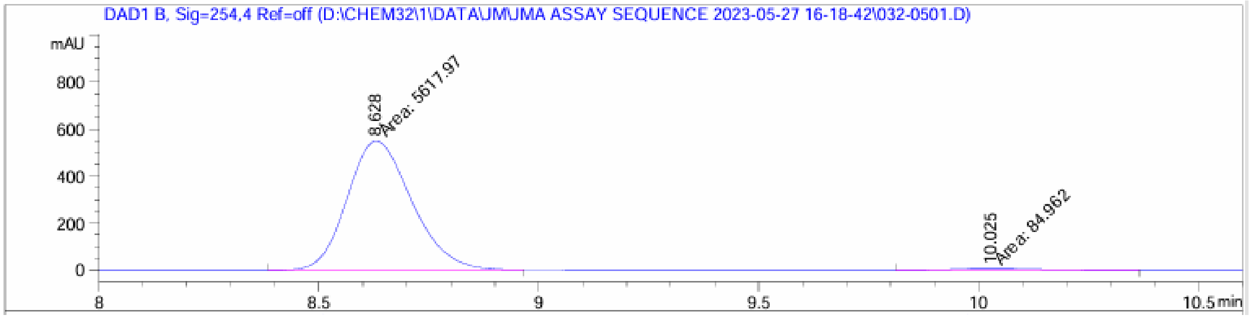

Peak #	RetTime [min]	Type	Width [min]	Area [mAU*s]	Height [mAU]	Area %
1	8.628	MF	0.1702	5617.96973	550.05383	98.5102
2	10.025	MM	0.2156	84.96195	6.56649	1.4898

2-((1,3-Dithian-2-yl)methyl)-3-(benzyloxy)-1-(4-methoxyphenyl)propan-1-one (*rac*-3cj)

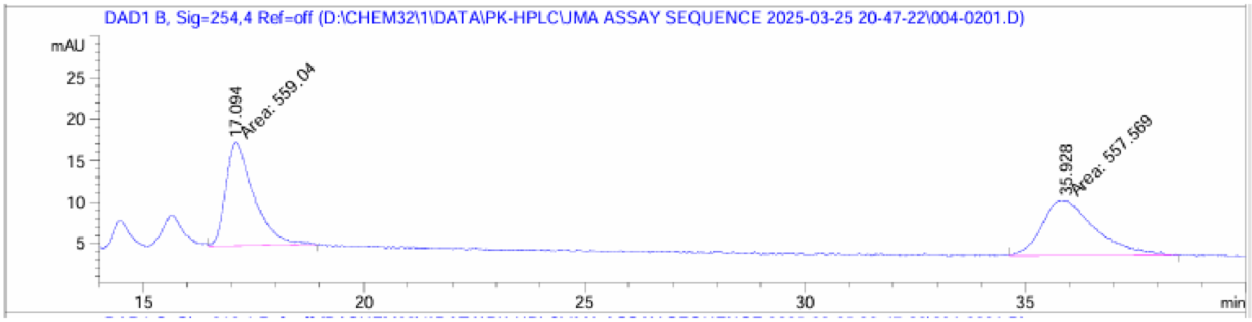

Peak #	RetTime [min]	Type	Width [min]	Area [mAU*s]	Height [mAU]	Area %
1	17.094	FM	0.7440	559.03973	12.52383	50.0659
2	35.928	MM	1.3948	557.56885	6.66247	49.9341

(*S*)-2-((1,3-Dithian-2-yl)methyl)-3-(benzyloxy)-1-(4-methoxyphenyl)propan-1-one (3cj)

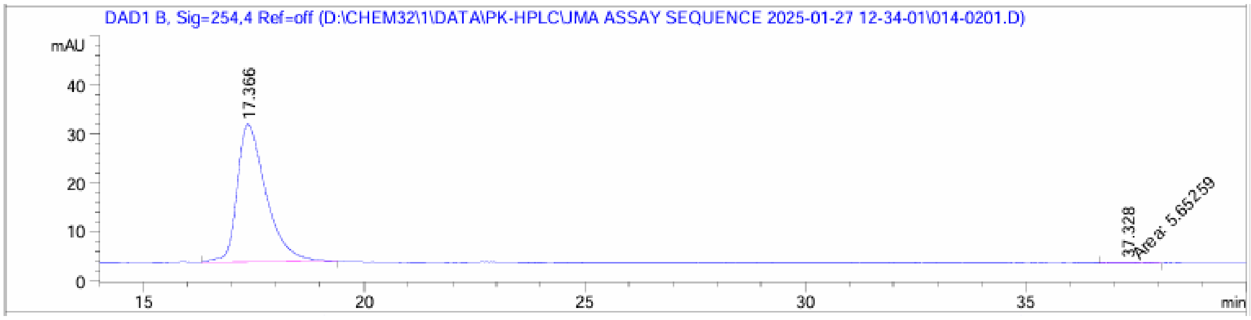

Peak #	RetTime [min]	Type	Width [min]	Area [mAU*s]	Height [mAU]	Area %
1	17.366	BB	0.6544	1280.09631	28.05114	99.5604
2	37.328	MM	0.7040	5.65259	1.33813e-1	0.4396

3-(Benzyloxy)-1-(2-fluoro-4-methoxyphenyl)-2-methylpropan-1-one (*rac*-**3de)**

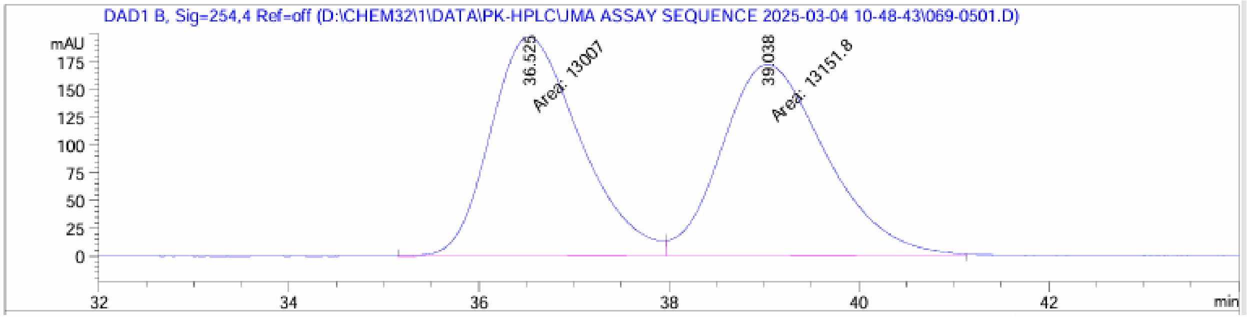

Peak #	RetTime [min]	Type	Width [min]	Area [mAU*s]	Height [mAU]	Area %
1	36.525	MF	1.0983	1.30070e4	197.37422	49.7233
2	39.038	FM	1.2729	1.31518e4	172.20787	50.2767

(*S*)- 3-(Benzyloxy)-1-(2-fluoro-4-methoxyphenyl)-2-methylpropan-1-one (3de)

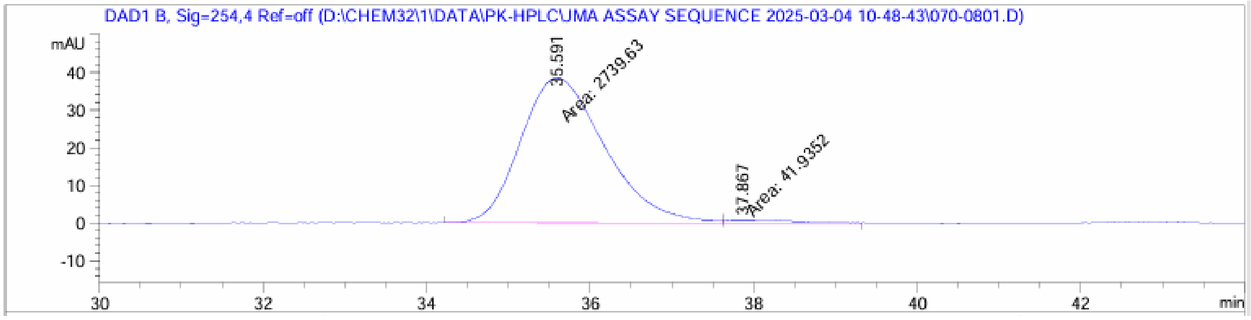

Peak #	RetTime [min]	Type	Width [min]	Area [mAU*s]	Height [mAU]	Area %
1	35.591	MF	1.1909	2739.63037	38.34019	98.4924
2	37.867	FM	0.8446	41.93524	8.27510e-1	1.5076

3-(Benzyloxy)-1-(2-bromo-4-methoxyphenyl)-2-methylpropan-1-one (*rac*-**3ee)**

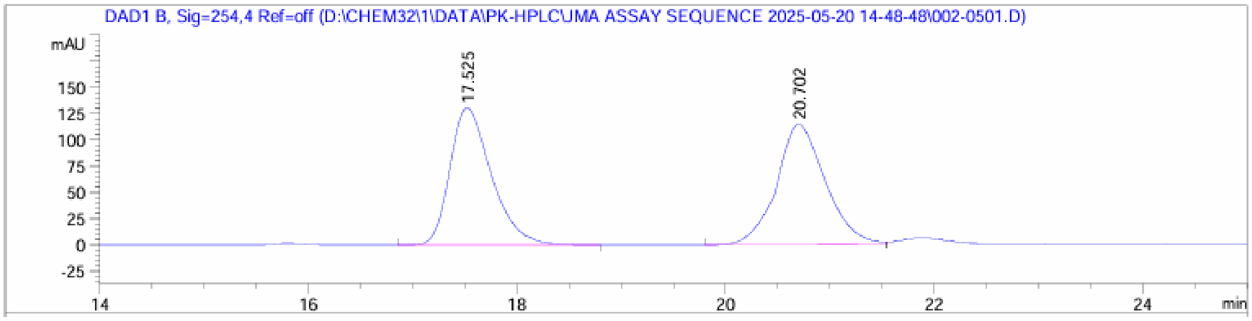

Peak #	RetTime [min]	Type	Width [min]	Area [mAU*s]	Height [mAU]	Area %
1	17.525	BB	0.4193	3594.36353	130.53281	49.0571
2	20.702	BV	0.4935	3732.54004	114.73305	50.9429

(*S*)-3-(Benzyloxy)-1-(2-bromo-4-methoxyphenyl)-2-methylpropan-1-one (3ee)

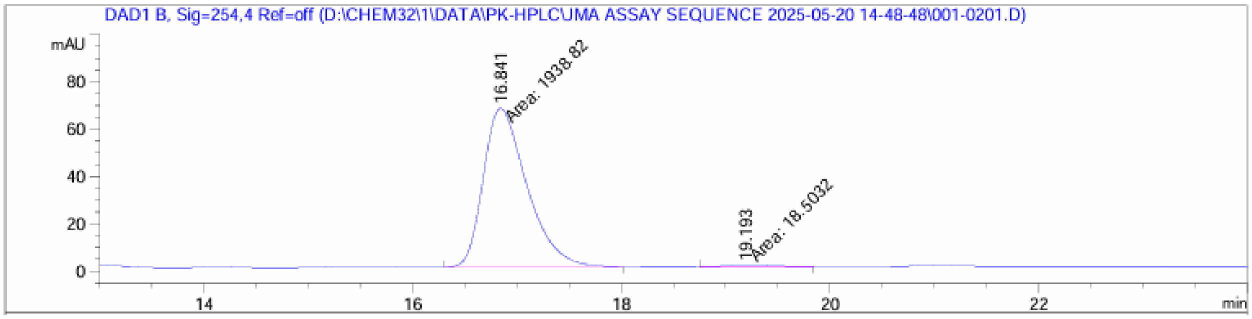

Peak #	RetTime [min]	Type	Width [min]	Area [mAU*s]	Height [mAU]	Area %
1	16.841	MF	0.4825	1938.81897	66.97419	99.0547
2	19.193	FM	0.5208	18.50315	5.92082e-1	0.9453

3-(Benzyloxy)-1-(2-fluoro-3-methoxyphenyl)-2-methylpropan-1-one (*rac*-3fe)

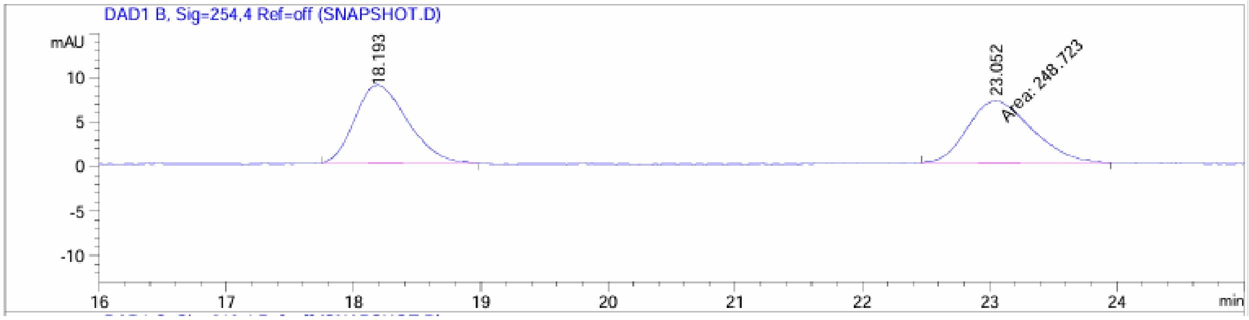

Peak #	RetTime [min]	Type	Width [min]	Area [mAU*s]	Height [mAU]	Area %
1	18.193	BB	0.4128	251.14259	8.75223	50.2420
2	23.052	MM	0.5924	248.72311	6.99728	49.7580

(*S*)-3-(Benzyloxy)-1-(2-fluoro-3-methoxyphenyl)-2-methylpropan-1-one (3fe)

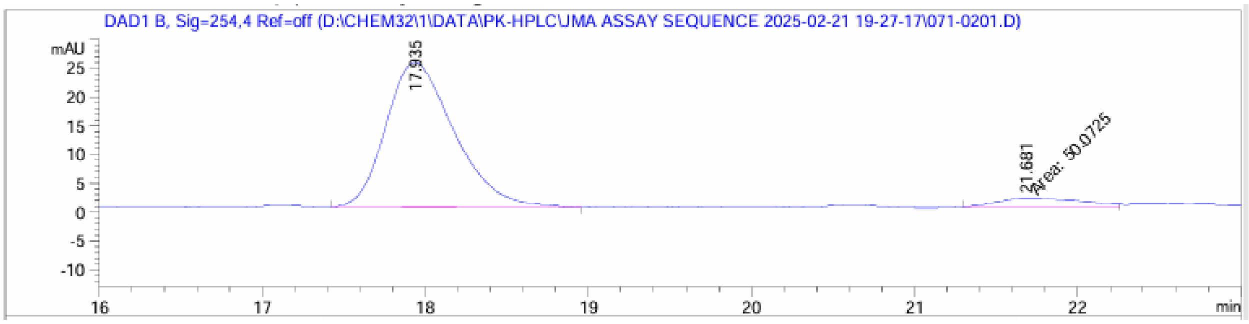

Peak #	RetTime [min]	Type	Width [min]	Area [mAU*s]	Height [mAU]	Area %
1	17.935	BB	0.4465	726.97180	25.05698	93.5560
2	21.681	MM	0.5710	50.07255	1.46162	6.4440

3-(Benzyloxy)-1-(4-fluoro-2-methylphenyl)-2-methylpropan-1-one (*rac*-3ge)

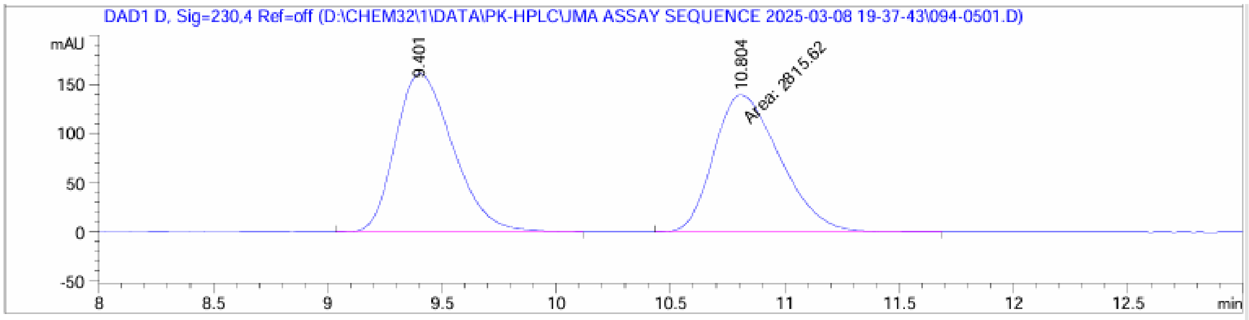

Peak #	RetTime [min]	Type	Width [min]	Area [mAU*s]	Height [mAU]	Area %
1	9.401	BB	0.2731	2834.78247	161.24229	50.1695
2	10.804	MM	0.3366	2815.62256	139.41838	49.8305

(*S*)-3-(Benzyloxy)-1-(4-fluoro-2-methylphenyl)-2-methylpropan-1-one (3ge)

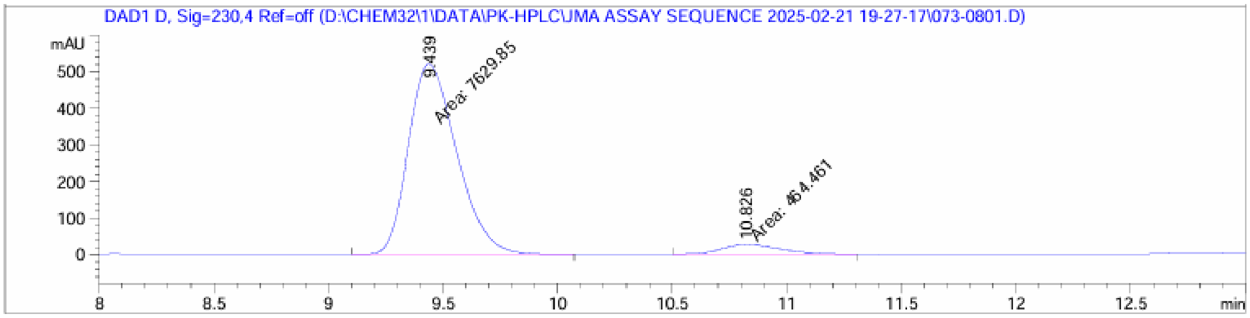

Peak #	RetTime [min]	Type	Width [min]	Area [mAU*s]	Height [mAU]	Area %
1	9.439	MM	0.2442	7629.84766	520.78369	94.2619
2	10.826	MM	0.2918	464.46097	26.53304	5.7381

3-(Benzyloxy)-1-(4-fluoro-2-methoxyphenyl)-2-methylpropan-1-one (*rac*-3he)

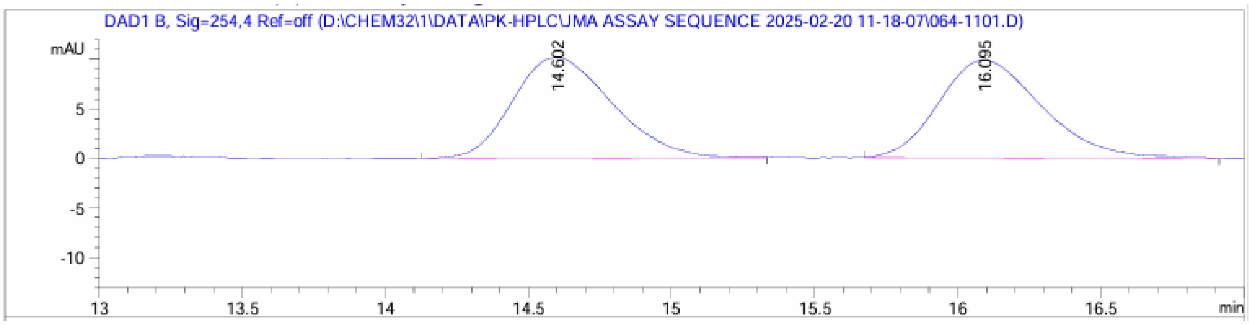

Peak #	RetTime [min]	Type	Width [min]	Area [mAU*s]	Height [mAU]	Area %
1	14.602	BB	0.3411	245.82196	10.20390	49.9471
2	16.095	BB	0.3782	246.34305	9.83612	50.0529

(*S*)-3-(Benzyloxy)-1-(4-fluoro-2-methoxyphenyl)-2-methylpropan-1-one (3he)

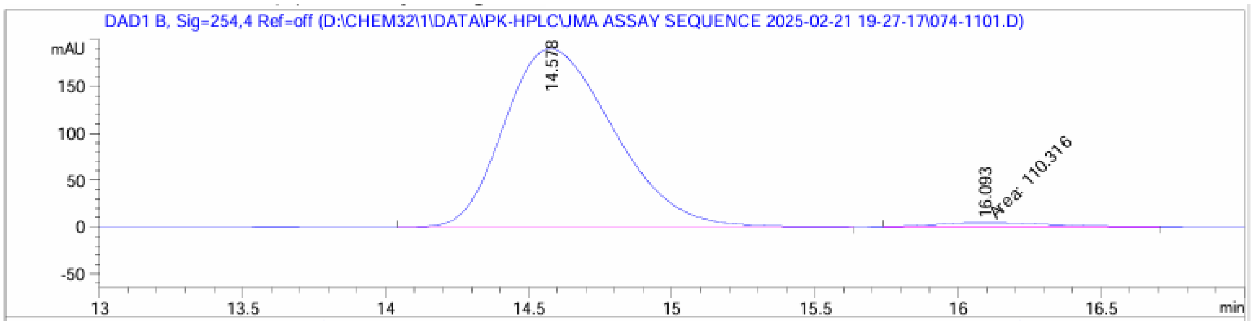

Peak #	RetTime [min]	Type	Width [min]	Area [mAU*s]	Height [mAU]	Area %
1	14.578	BB	0.4153	5046.63428	190.36815	97.8608
2	16.093	MM	0.4282	110.31593	4.29378	2.1392

3-(Benzyloxy)-1-(5-bromo-2-methoxyphenyl)-2-methylpropan-1-one (*rac*-**3ie**)

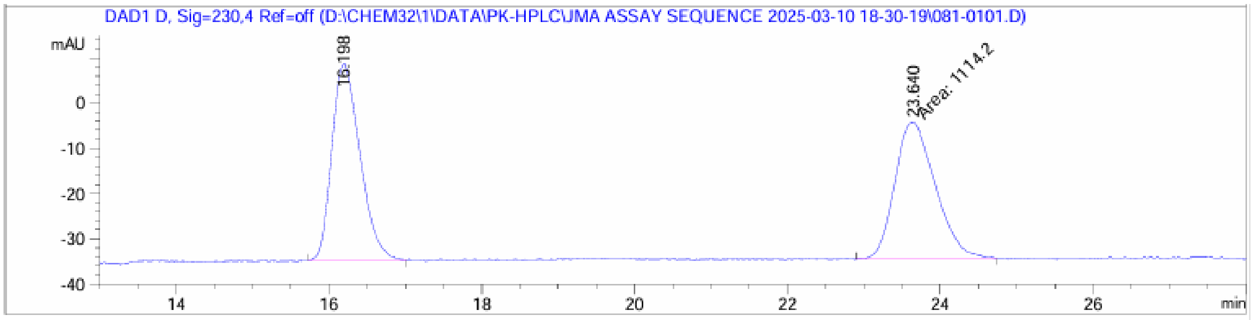

Peak #	RetTime [min]	Type	Width [min]	Area [mAU*s]	Height [mAU]	Area %
1	16.198	BV	0.3826	1117.55432	43.66252	50.0752
2	23.640	MM	0.6138	1114.19971	30.25459	49.9248

(*S*)-3-(Benzyloxy)-1-(5-bromo-2-methoxyphenyl)-2-methylpropan-1-one (3ie)

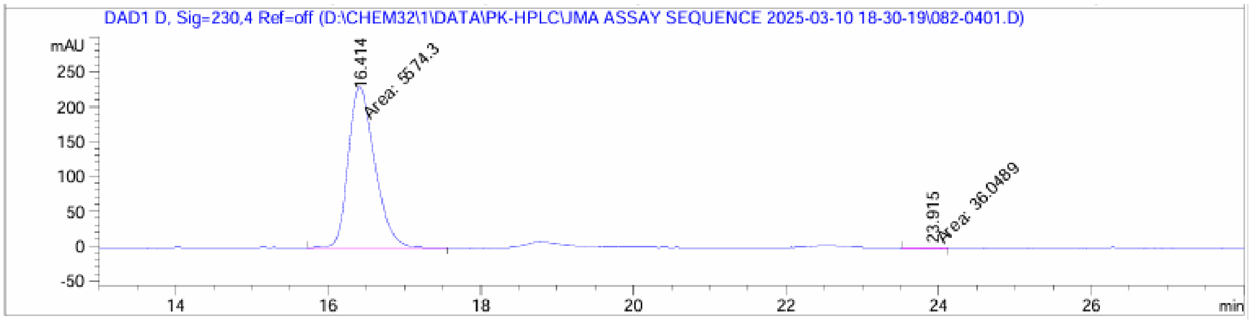

Peak #	RetTime [min]	Type	Width [min]	Area [mAU*s]	Height [mAU]	Area %
1	16.414	MM	0.4023	5574.30078	230.94879	99.3575
2	23.915	MM	0.5134	36.04888	1.17028	0.6425

3-(Benzyloxy)-1-(4-bromo-3-methoxyphenyl)-2-methylpropan-1-one (*rac*-3je)

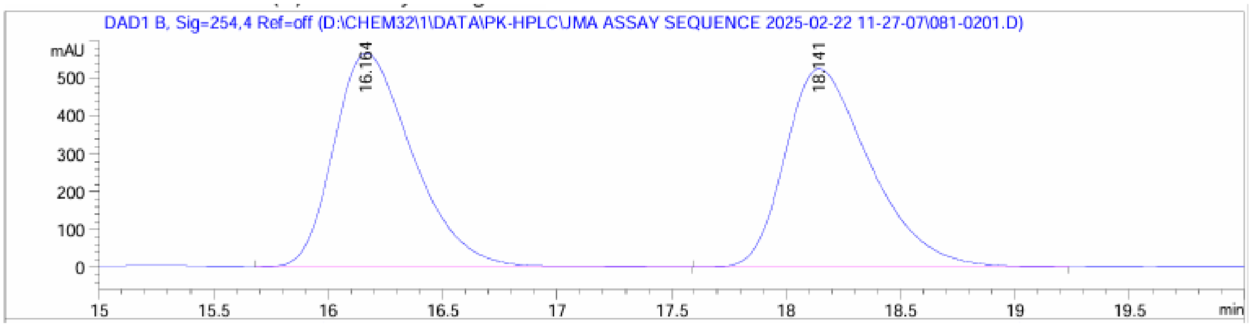

Peak #	RetTime [min]	Type	Width [min]	Area [mAU*s]	Height [mAU]	Area %
1	16.164	BB	0.3689	1.36568e4	567.30139	50.0963
2	18.141	BB	0.3936	1.36043e4	526.20447	49.9037

(*S*)-3-(Benzyloxy)-1-(4-bromo-3-methoxyphenyl)-2-methylpropan-1-one (3je)

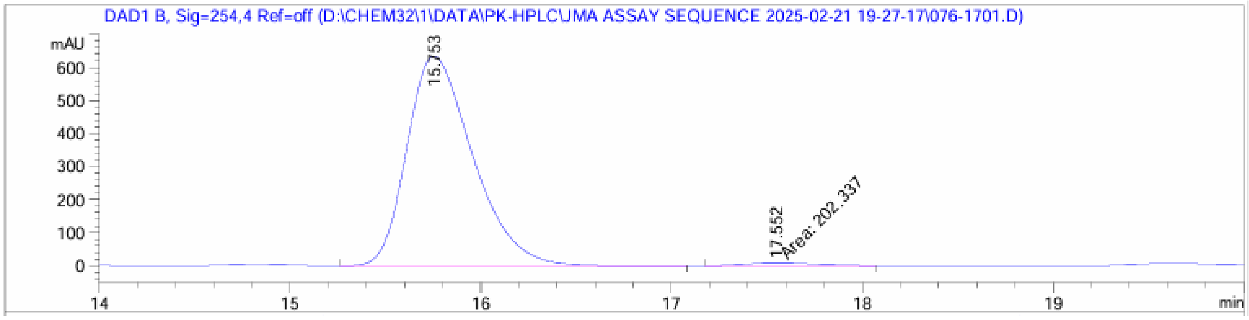

Peak #	RetTime [min]	Type	Width [min]	Area [mAU*s]	Height [mAU]	Area %
1	15.753	BB	0.3650	1.51724e4	634.35956	98.6840
2	17.552	MM	0.4140	202.33688	8.14486	1.3160

3-(Benzyloxy)-1-(3-chloro-5-methylphenyl)-2-methylpropan-1-one (*rac*-3ke)

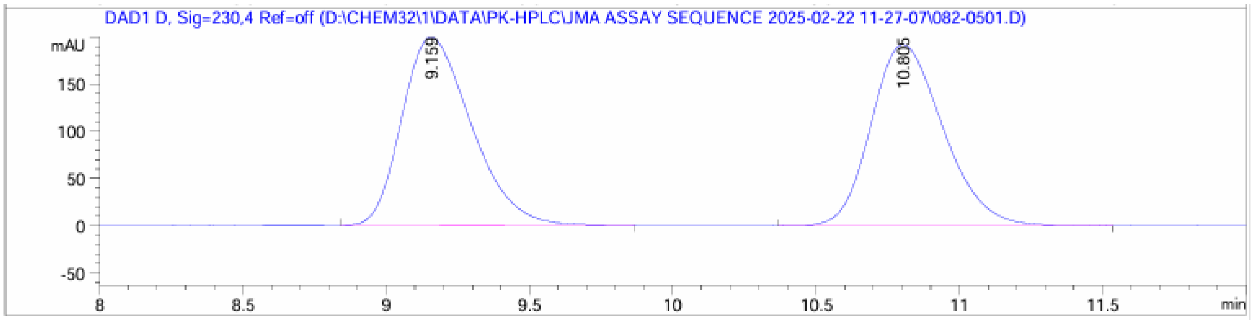

Peak #	RetTime [min]	Type	Width [min]	Area [mAU*s]	Height [mAU]	Area %
1	9.159	VB	0.2637	3386.15942	199.82388	50.2219
2	10.805	BB	0.2701	3356.23267	191.85020	49.7781

(*S*)-3-(Benzyloxy)-1-(3-chloro-5-methylphenyl)-2-methylpropan-1-one (3ke)

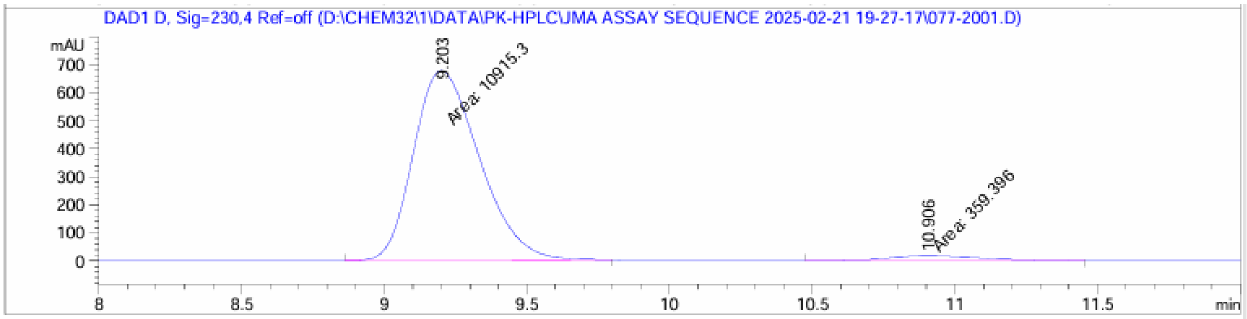

Peak #	RetTime [min]	Type	Width [min]	Area [mAU*s]	Height [mAU]	Area %
1	9.203	MM	0.2683	1.09153e4	678.14240	96.8124
2	10.906	MM	0.3570	359.39624	16.77741	3.1876

3-(Benzyloxy)-1-(3-chloro-4-hydroxy-5-methoxyphenyl)-2-methylpropan-1-one (*rac*-**3le**)

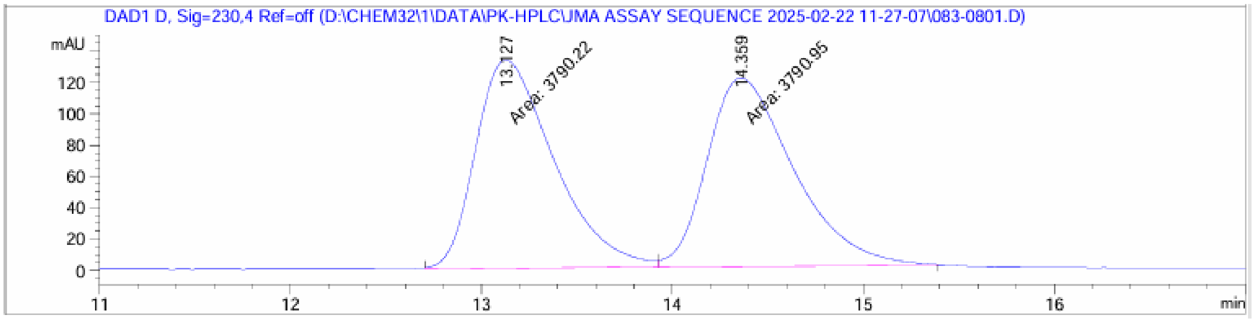

Peak #	RetTime [min]	Type	Width [min]	Area [mAU*s]	Height [mAU]	Area %
1	13.127	MF	0.4752	3790.21997	132.93552	49.9952
2	14.359	FM	0.5252	3790.94604	120.30843	50.0048

(*S*)-3-(Benzyloxy)-1-(3-chloro-4-hydroxy-5-methoxyphenyl)-2-methylpropan-1-one (3le)

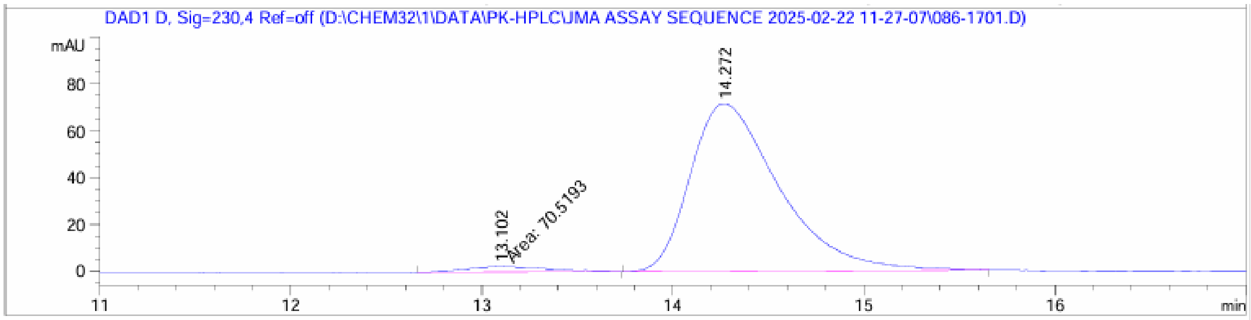

Peak #	RetTime [min]	Type	Width [min]	Area [mAU*s]	Height [mAU]	Area %
1	13.102	MM	0.5013	70.51926	2.34437	2.9582
2	14.272	BB	0.4715	2313.32983	71.49970	97.0418

3-(Benzyloxy)-2-methyl-1-(3,4,5-trimethoxyphenyl)propan-1-one (*rac*-3me)

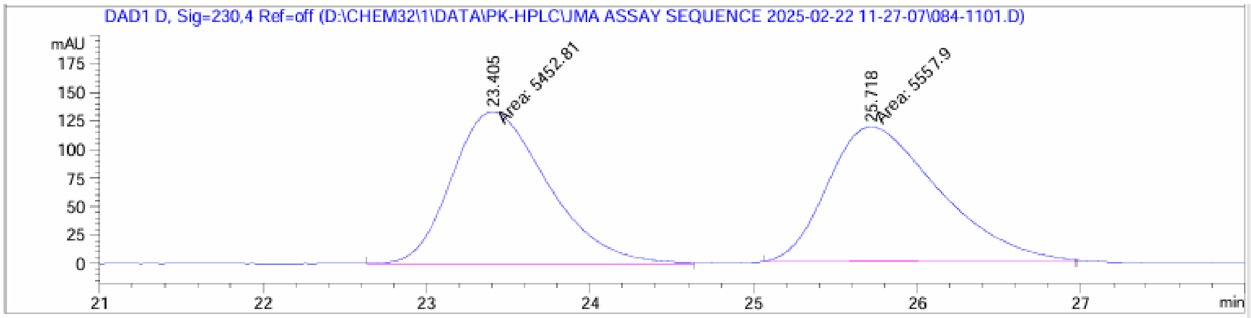

Peak #	RetTime [min]	Type	Width [min]	Area [mAU*s]	Height [mAU]	Area %
1	23.405	MM	0.6809	5452.81348	133.47427	49.5228
2	25.718	MM	0.7863	5557.89648	117.80183	50.4772

(*S*)-3-(Benzyloxy)-2-methyl-1-(3,4,5-trimethoxyphenyl)propan-1-one (3me)

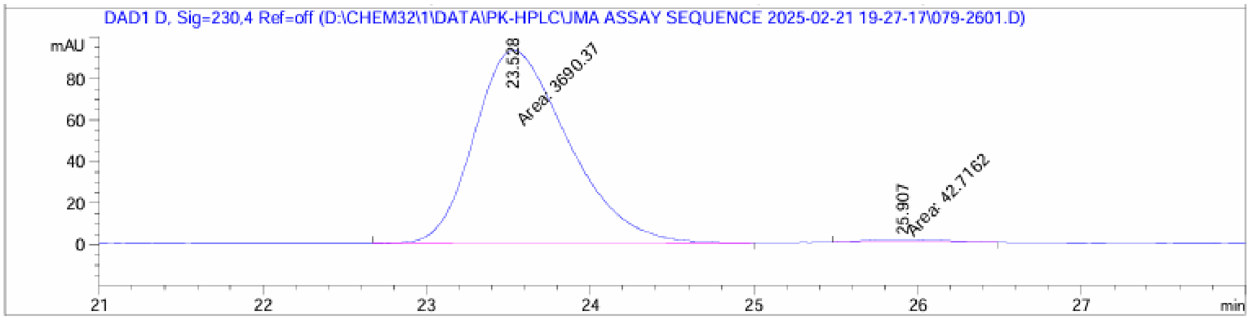

Peak #	RetTime [min]	Type	Width [min]	Area [mAU*s]	Height [mAU]	Area %
1	23.528	MM	0.6627	3690.37256	92.81611	98.8557
2	25.907	MM	0.5351	42.71618	1.33050	1.1443

(*E*)-1-(Benzyloxy)-5-(4-methoxyphenyl)-2-methylhex-4-en-3-one (*rac*-3ne)

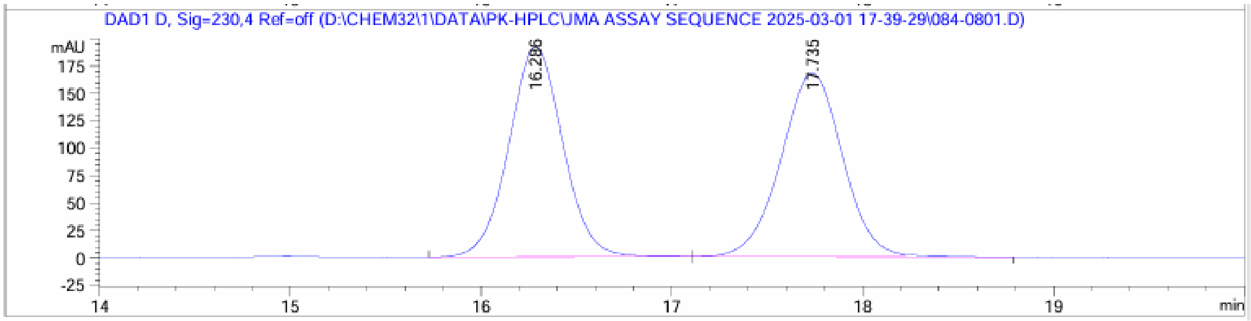

Peak #	RetTime [min]	Type	Width [min]	Area [mAU*s]	Height [mAU]	Area %
1	16.286	BB	0.3012	3760.57373	191.45416	49.9239
2	17.735	BB	0.3471	3772.04028	167.27206	50.0761

(*S*,*E*)-1-(Benzyloxy)-5-(4-methoxyphenyl)-2-methylhex-4-en-3-one (3ne)

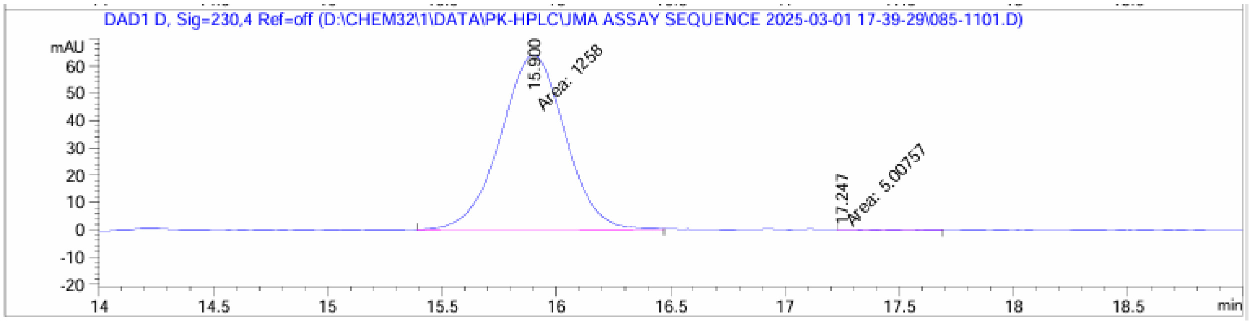

Peak #	RetTime [min]	Type	Width [min]	Area [mAU*s]	Height [mAU]	Area %
1	15.900	MM	0.3276	1258.00195	63.99985	99.6035
2	17.247	MM	0.1972	5.00757	4.23291e-1	0.3965

(*E*)-1-(Benzyloxy)-2-methyl-5-(p-tolyl)hex-4-en-3-one (*rac*-**3oe**)

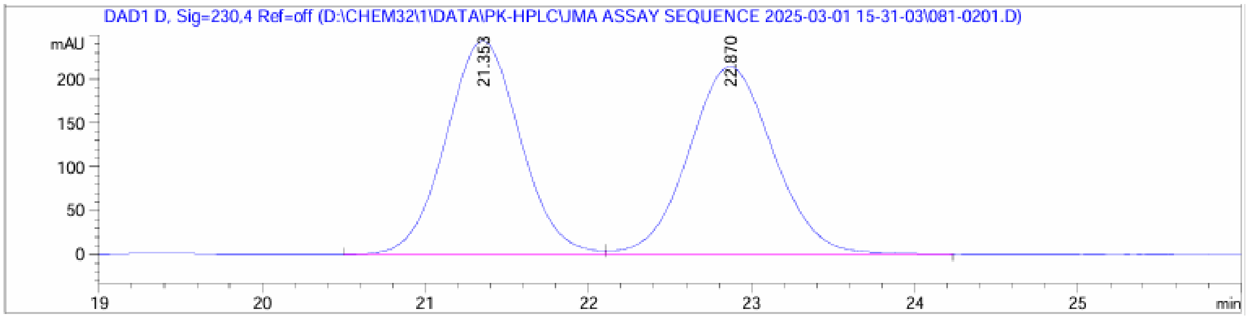

Peak #	RetTime [min]	Type	Width [min]	Area [mAU*s]	Height [mAU]	Area %
1	21.353	BV	0.4810	7697.40234	243.40103	49.8770
2	22.870	VB	0.5576	7735.36621	214.04486	50.1230

(*S*,*E*)-1-(Benzyloxy)-2-methyl-5-(*p*-tolyl)hex-4-en-3-one (3oe)

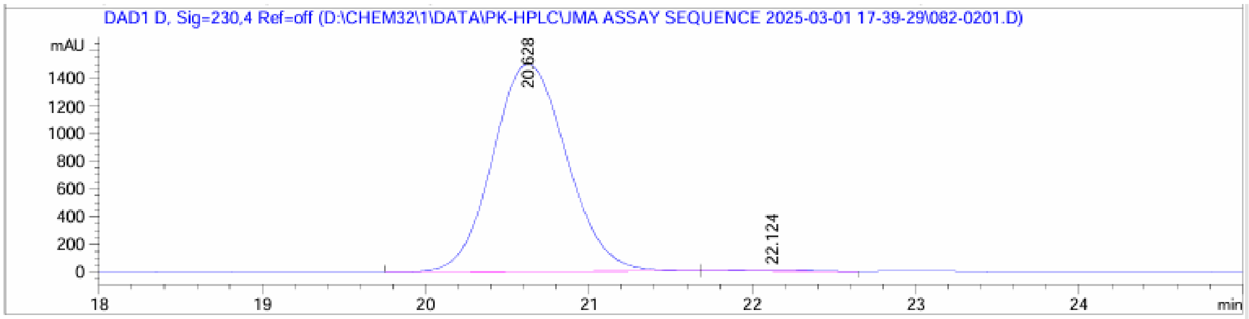

Peak #	RetTime [min]	Type	Width [min]	Area [mAU*s]	Height [mAU]	Area %
1	20.628	BB	0.4923	4.71436e4	1501.65979	99.4091
2	22.124	BB	0.3253	280.21182	11.06907	0.5909

(*E*)-1-(Benzyloxy)-2-methyl-5-phenylhex-4-en-3-one (*rac*-3pe)

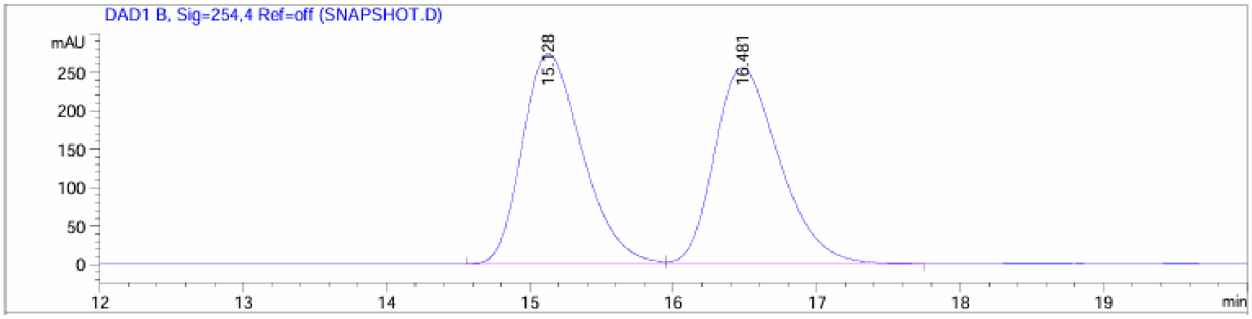

Peak #	RetTime [min]	Type	Width [min]	Area [mAU*s]	Height [mAU]	Area %
1	15.128	BV	0.4502	7968.19629	273.27353	49.7797
2	16.481	VB	0.4852	8038.72607	255.38525	50.2203

(*S*,*E*)-1-(Benzyloxy)-2-methyl-5-phenylhex-4-en-3-one (3pe)

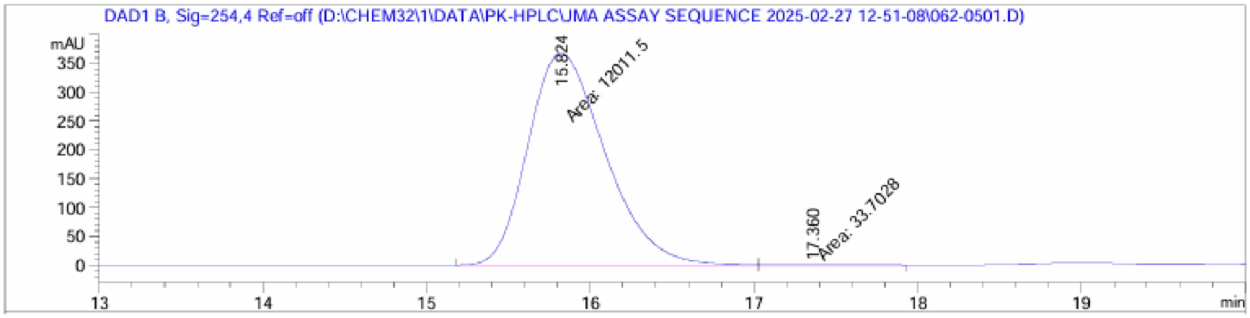

Peak #	RetTime [min]	Type	Width [min]	Area [mAU*s]	Height [mAU]	Area %
1	15.824	MF	0.5449	1.20115e4	367.36783	99.7202
2	17.360	FM	0.5612	33.70282	1.00094	0.2798

(*E*)-1-(Benzyloxy)-5-(4-fluorophenyl)-2-methylhex-4-en-3-one (*rac*-3qe)

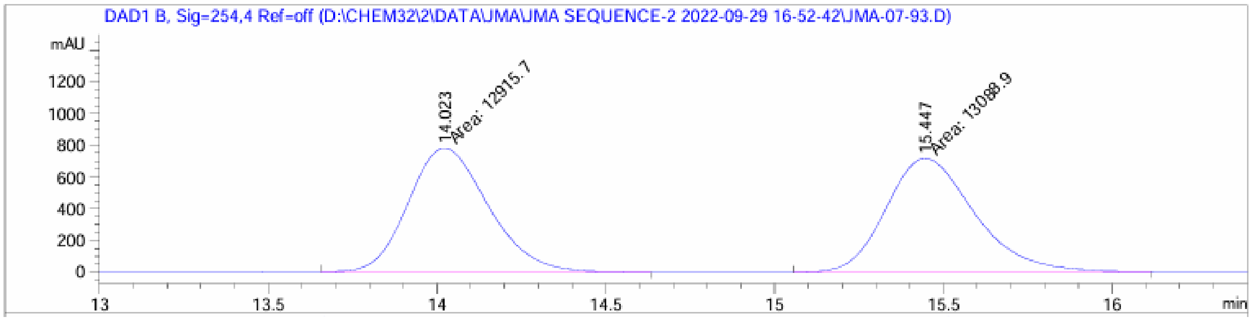

Peak #	RetTime [min]	Type	Width [min]	Area [mAU*s]	Height [mAU]	Area %
1	14.023	MM	0.2751	1.29157e4	782.58331	49.6671
2	15.447	MM	0.3042	1.30889e4	717.11823	50.3329

(*S*,*E*)-1-(Benzyloxy)-5-(4-fluorophenyl)-2-methylhex-4-en-3-one (3qe)

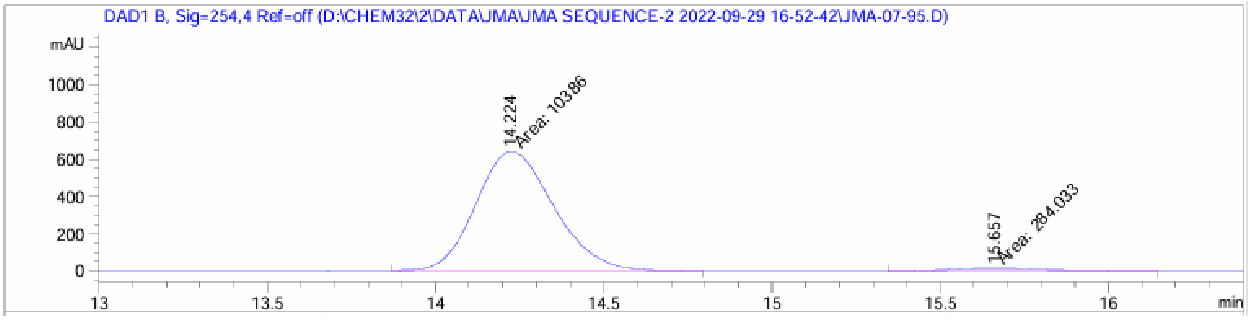

Peak #	RetTime [min]	Type	Width [min]	Area [mAU*s]	Height [mAU]	Area %
1	14.224	MM	0.2693	1.03860e4	642.69989	97.3380
2	15.657	MM	0.3117	284.03326	15.18612	2.6620

(*E*)-1-(Benzyloxy)-5-(4-bromophenyl)-2-methylhex-4-en-3-one (*rac*-3re)

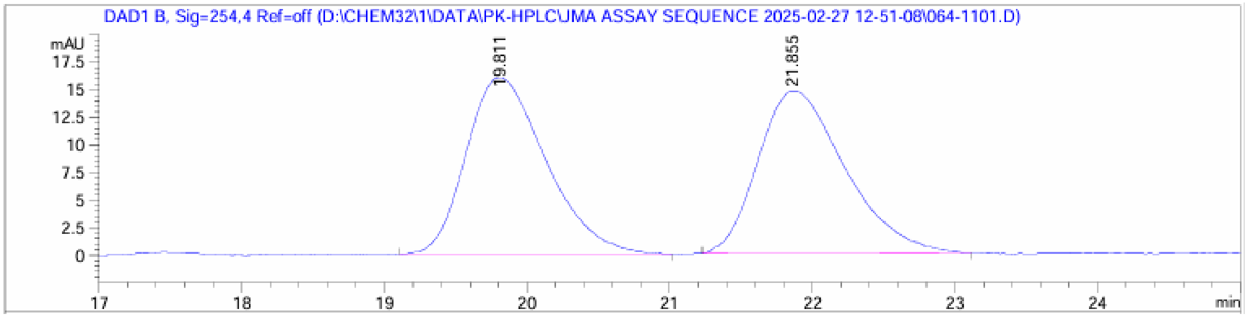

Peak #	RetTime [min]	Type	Width [min]	Area [mAU*s]	Height [mAU]	Area %
1	19.811	BB	0.5223	624.00842	16.02655	50.0987
2	21.855	BB	0.5115	621.54919	14.69415	49.9013

(*S*,*E*)-1-(Benzyloxy)-5-(4-bromophenyl)-2-methylhex-4-en-3-one (3re)

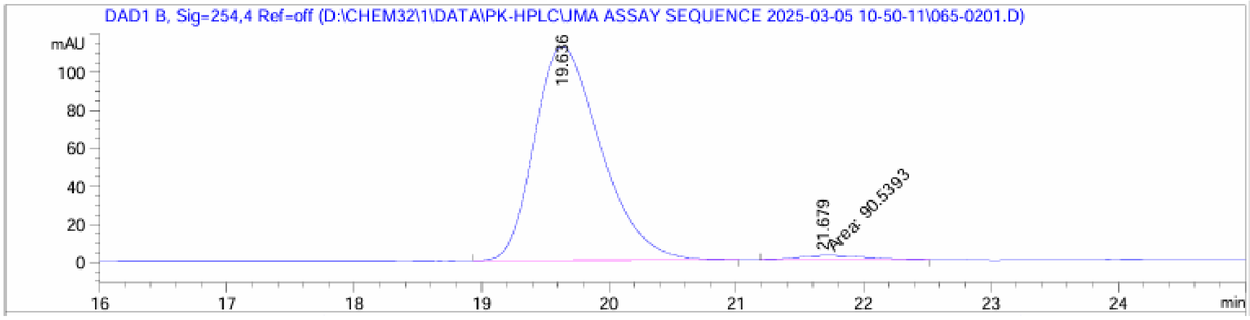

Peak #	RetTime [min]	Type	Width [min]	Area [mAU*s]	Height [mAU]	Area %
1	19.636	BB	0.5569	4081.27930	112.59811	97.8297
2	21.679	MM	0.6512	90.53930	2.31734	2.1703

(*E*)-5-(Benzo[*d*][1,3]dioxol-5-yl)-1-(benzyloxy)-2-methylhex-4-en-3-one (*rac*-**3se**)

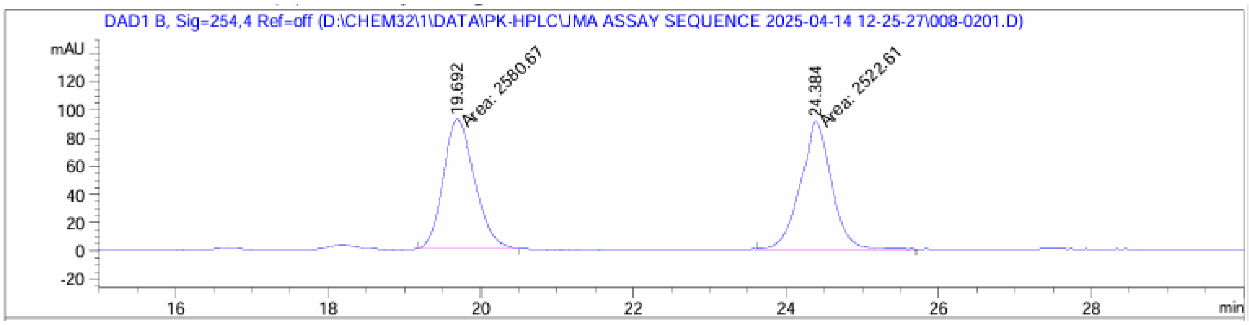

Peak #	RetTime [min]	Type	Width [min]	Area [mAU*s]	Height [mAU]	Area %
1	19.692	MM	0.4707	2580.66577	91.37148	50.5688
2	24.384	MM	0.4642	2522.61328	90.57114	49.4312

(*S*,*E*)-5-(Benzo[*d*][1,3]dioxol-5-yl)-1-(benzyloxy)-2-methylhex-4-en-3-one (3se)

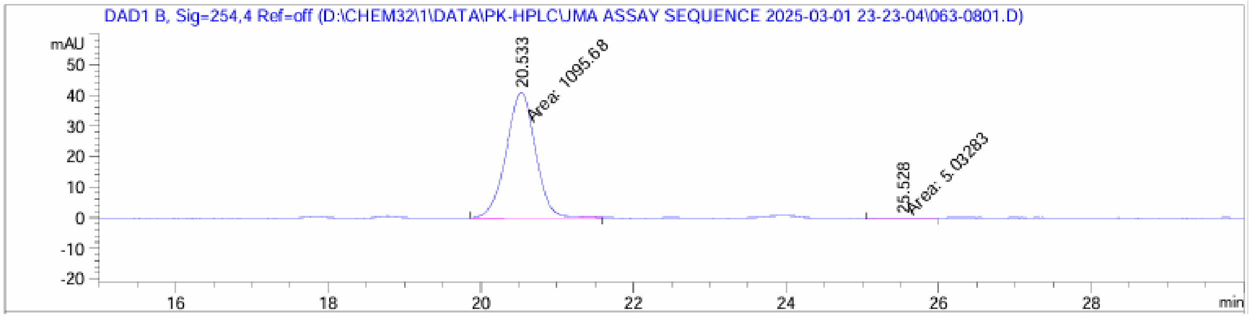

Peak #	RetTime [min]	Type	Width [min]	Area [mAU*s]	Height [mAU]	Area %
1	20.533	MM	0.4448	1095.68030	41.05573	99.5428
2	25.528	MM	0.5376	5.03283	1.56028e-1	0.4572

(*E*)-1-(Benzyloxy)-2-methyl-5-(naphthalen-2-yl)hex-4-en-3-one (*rac*-3te)

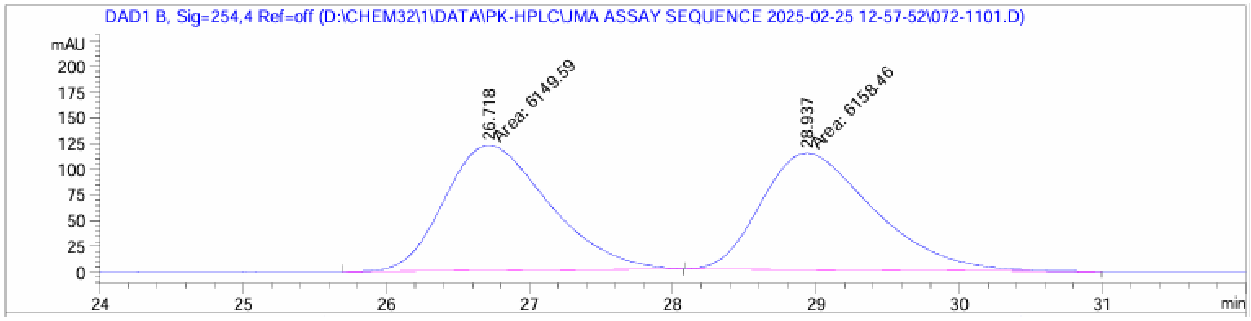

Peak #	RetTime [min]	Type	Width [min]	Area [mAU*s]	Height [mAU]	Area %
1	26.718	MM	0.8429	6149.59082	121.59467	49.9640
2	28.937	MM	0.9081	6158.45752	113.03287	50.0360

(*S*,*E*)-1-(Benzyloxy)-2-methyl-5-(naphthalen-2-yl)hex-4-en-3-one (3te)

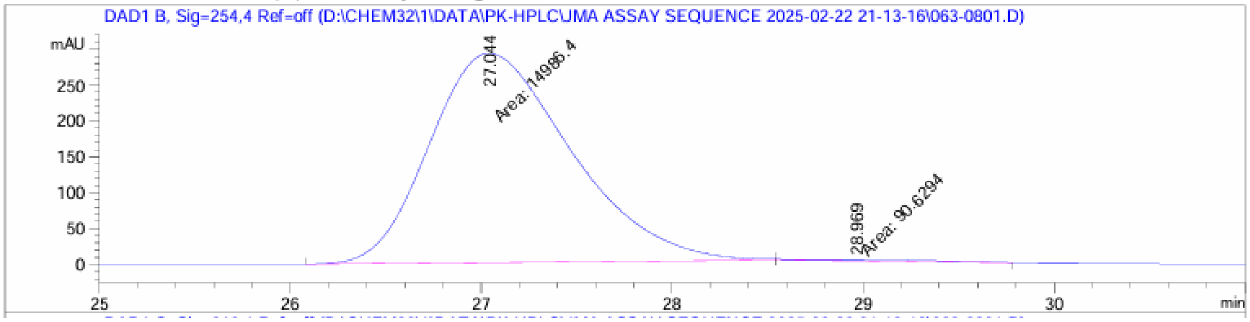

Peak #	RetTime [min]	Type	Width [min]	Area [mAU*s]	Height [mAU]	Area %
1	27.044	MM	0.8562	1.49864e4	291.70990	99.3989
2	28.969	MM	0.9054	90.62942	1.66822	0.6011

(*E*)-5-(Benzyloxy)-4-methyl-1-phenylpent-1-en-3-one (*rac*-3ue)

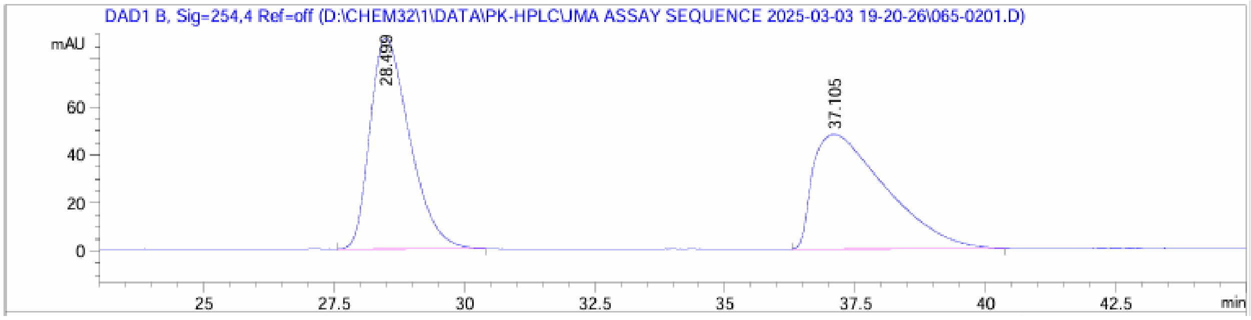

Peak #	RetTime [min]	Type	Width [min]	Area [mAU*s]	Height [mAU]	Area %
1	28.499	BB	0.7690	4623.18848	87.41247	50.0628
2	37.105	BB	1.1452	4611.58643	47.47690	49.9372

(*S*,*E*)-5-(Benzyloxy)-4-methyl-1-phenylpent-1-en-3-one (3ue)

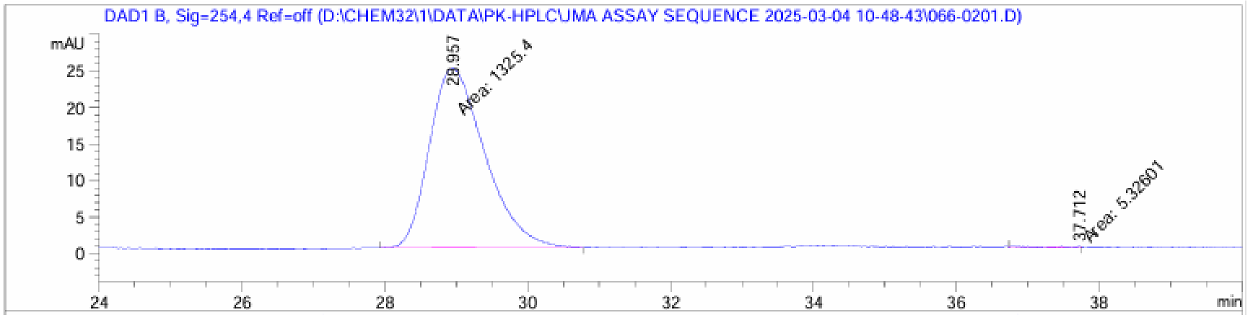

Peak #	RetTime [min]	Type	Width [min]	Area [mAU*s]	Height [mAU]	Area %
1	28.957	MM	0.8990	1325.39502	24.57146	99.5998
2	37.712	MM	0.5689	5.32601	1.56028e-1	0.4002

(*E*)-1-(Benzyloxy)-2-methyl-5-phenylnon-4-en-3-one (*rac*-3ve)

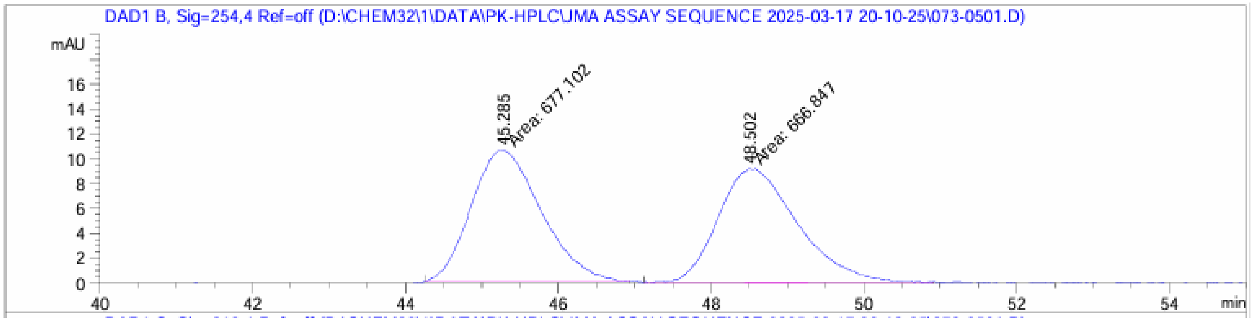

Peak #	RetTime [min]	Type	Width [min]	Area [mAU*s]	Height [mAU]	Area %
1	45.285	MM	1.0647	677.10248	10.59969	50.3815
2	48.502	FM	1.2077	666.84705	9.20245	49.6185

(*S*,*E*)-1-(Benzyloxy)-2-methyl-5-phenylnon-4-en-3-one (3ve)

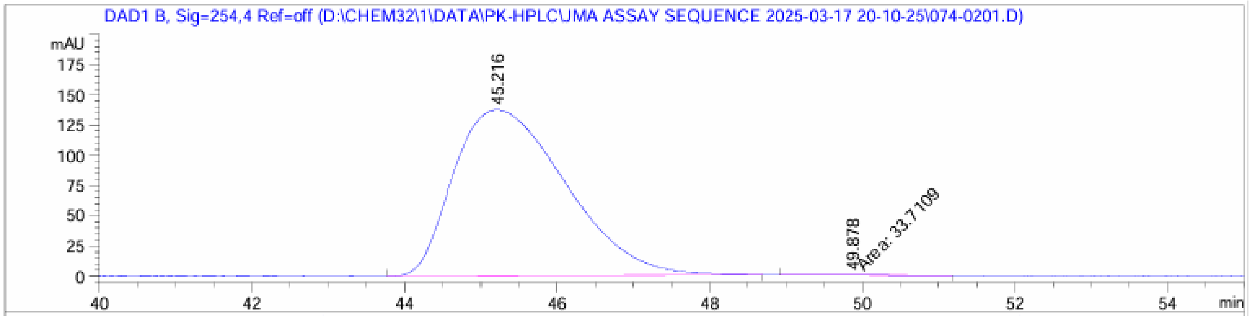

Peak #	RetTime [min]	Type	Width [min]	Area [mAU*s]	Height [mAU]	Area %
1	45.216	BB	1.2365	1.39830e4	137.02805	99.7595
2	49.878	MM	1.1208	33.71091	5.01304e-1	0.2405

(*E*)-5-(Benzyloxy)-1-cyclopropyl-4-methyl-1-phenylpent-1-en-3-one (*rac*-3we)

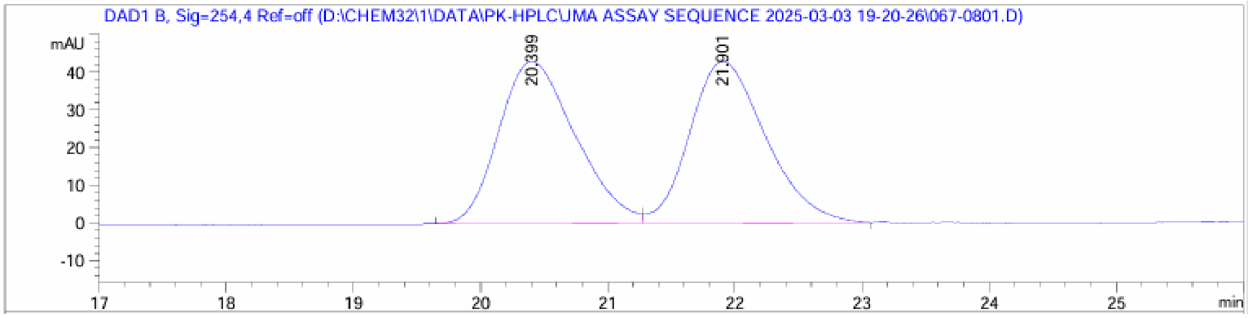

Peak #	RetTime [min]	Type	Width [min]	Area [mAU*s]	Height [mAU]	Area %
1	20.399	BV	0.6213	1792.66248	42.91748	50.2622
2	21.901	VB	0.6123	1773.96204	42.73730	49.7378

(*S,E*)-5-(Benzyloxy)-1-cyclopropyl-4-methyl-1-phenylpent-1-en-3-one (3we)

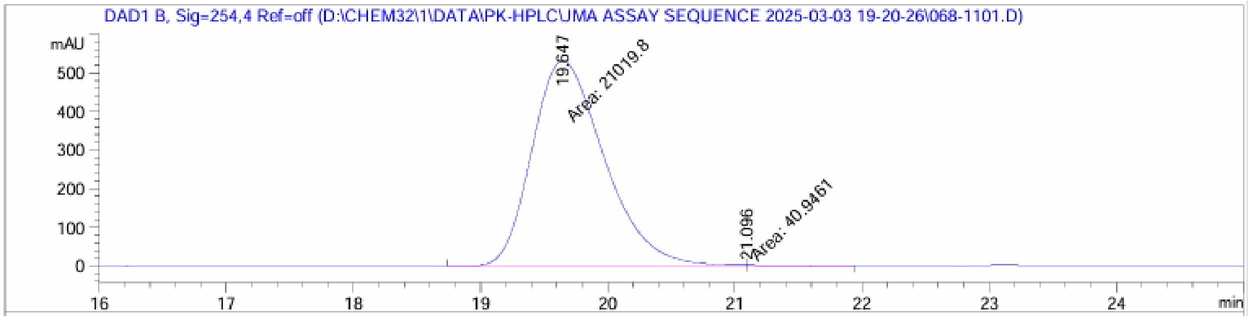

Peak #	RetTime [min]	Type	Width [min]	Area [mAU*s]	Height [mAU]	Area %
1	19.647	MF	0.6590	2.10198e4	531.58624	99.8056
2	21.096	FM	0.3259	40.94613	2.09416	0.1944

(4-Methoxyphenyl)(tetrahydrothiophen-3-yl)methanone (*rac*-**3ck**)

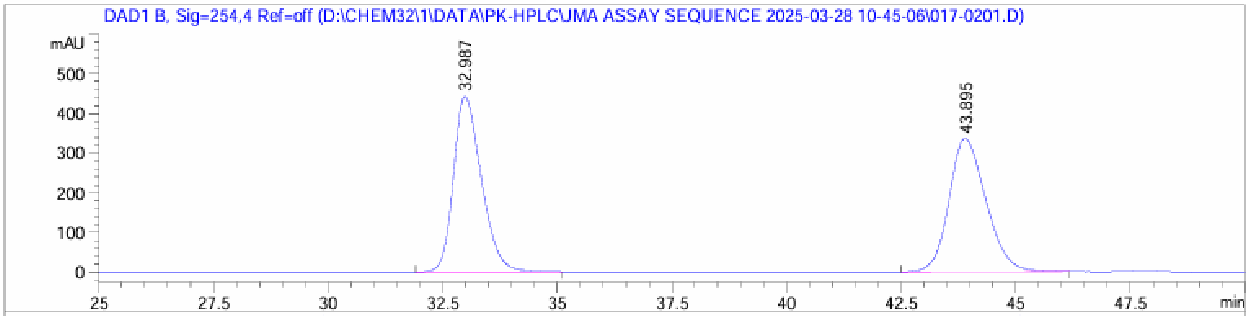

Peak #	RetTime [min]	Type	Width [min]	Area [mAU*s]	Height [mAU]	Area %
1	32.987	BB	0.6427	1.88475e4	442.67542	49.9739
2	43.895	BB	0.8347	1.88672e4	336.19208	50.0261

(*R*)-(4-Methoxyphenyl)(tetrahydrothiophen-3-yl)methanone (3ck)

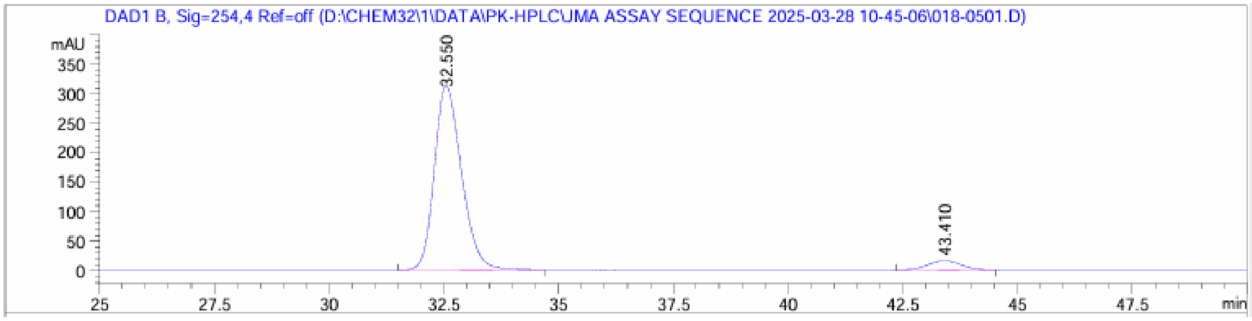

Peak #	RetTime [min]	Type	Width [min]	Area [mAU*s]	Height [mAU]	Area %
1	32.550	BB	0.6260	1.29547e4	313.55896	93.8753
2	43.410	BB	0.6310	845.20166	16.40793	6.1247

(1-Benzylpyrrolidin-3-yl)(4-methoxyphenyl)methanone (*rac*-3cl)

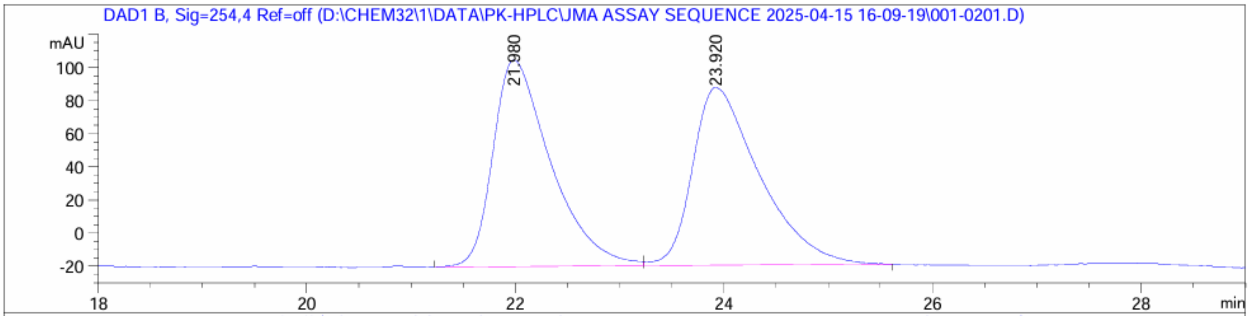

Peak #	RetTime [min]	Type	Width [min]	Area [mAU*s]	Height [mAU]	Area %
1	21.980	BV	0.5590	4800.61035	124.75043	49.9736
2	23.920	VB	0.6221	4805.67920	107.38121	50.0264

(*R*)-(1-Benzylpyrrolidin-3-yl)(4-methoxyphenyl)methanone (3cl)

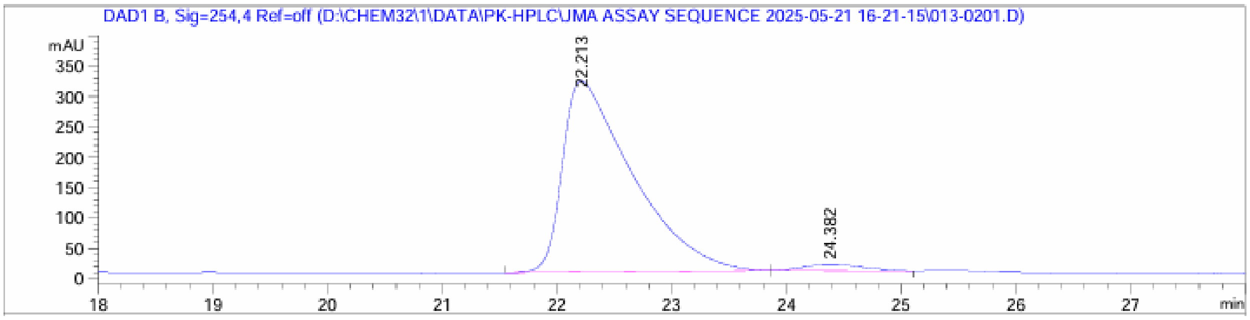

Peak #	RetTime [min]	Type	Width [min]	Area [mAU*s]	Height [mAU]	Area %
1	22.213	BB	0.6083	1.33052e4	314.01767	97.3104
2	24.382	BB	0.5162	367.75293	10.56200	2.6896

(4-Methoxyphenyl)(tetrahydrofuran-3-yl)methanone (*rac*-3cm)

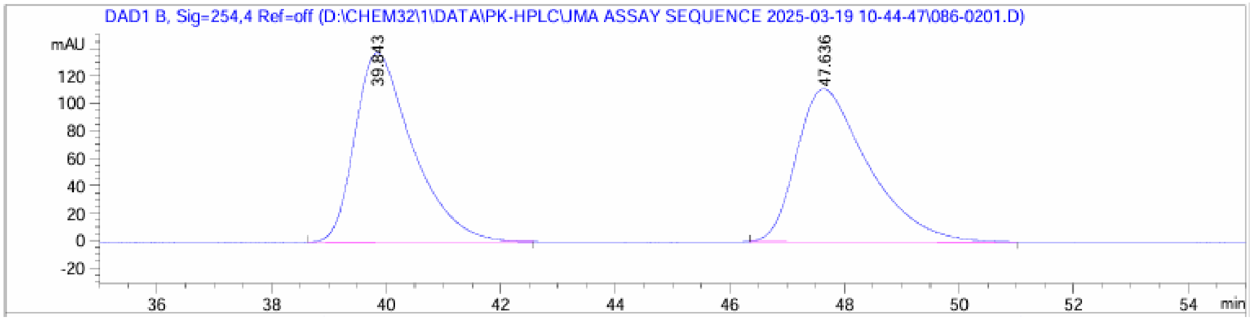

Peak #	RetTime [min]	Type	Width [min]	Area [mAU*s]	Height [mAU]	Area %
1	39.843	BB	0.9954	9681.09668	137.83249	50.0344
2	47.636	BB	1.1725	9667.77441	111.39832	49.9656

(*R*)-(4-Methoxyphenyl)(tetrahydrofuran-3-yl)methanone (3cm)

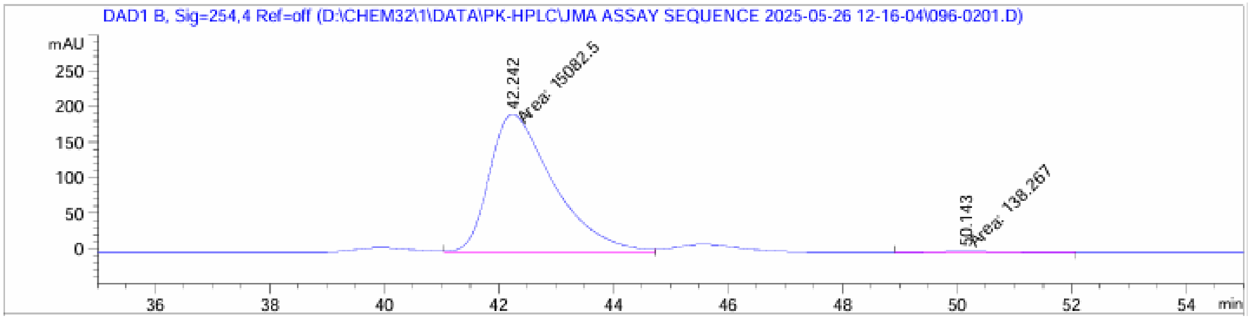

Peak #	RetTime [min]	Type	Width [min]	Area [mAU*s]	Height [mAU]	Area %
1	42.242	MF	1.2962	1.50825e4	193.93124	99.0916
2	50.143	MM	1.4648	138.26672	1.57322	0.9084

4-Methoxyphenyl 3-(benzyloxy)-2-methylpropanoate (*rac*-4)

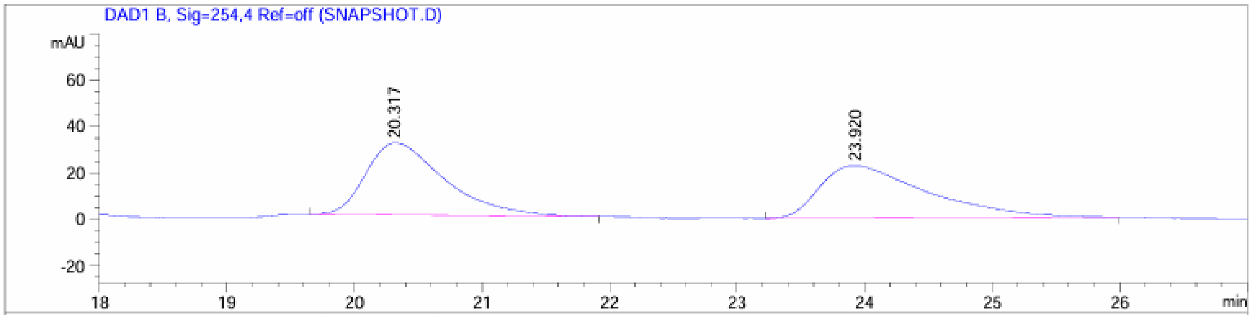

Peak #	RetTime [min]	Type	Width [min]	Area [mAU*s]	Height [mAU]	Area %
1	20.317	BB	0.5882	1324.03235	31.00482	50.1626
2	23.920	BB	0.7103	1315.44812	22.49362	49.8374

4-Methoxyphenyl (*S*)-3-(benzyloxy)-2-methylpropanoate (4)

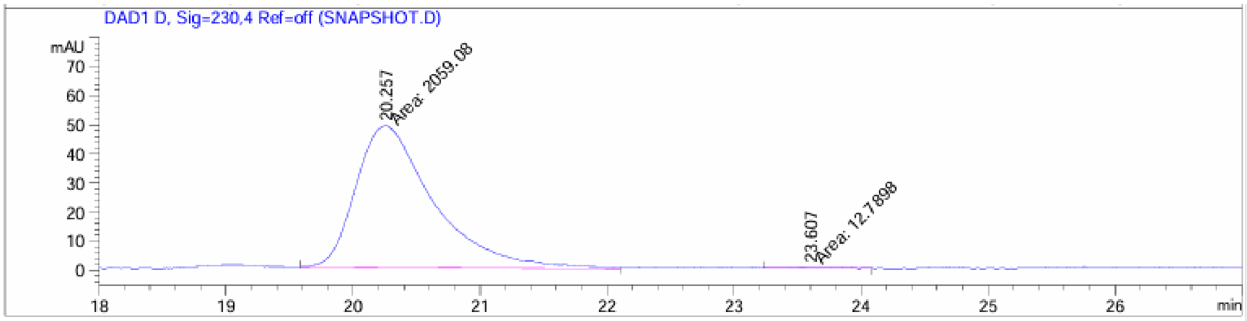

Peak #	RetTime [min]	Type	Width [min]	Area [mAU*s]	Height [mAU]	Area %
1	20.257	MM	0.7044	2059.08057	48.71708	99.3827
2	23.607	MM	0.4790	12.78977	4.45016e-1	0.6173

4-(Benzyloxy)-1-(4-methoxyphenyl)-3-methylbutan-2-one (*rac*-5)

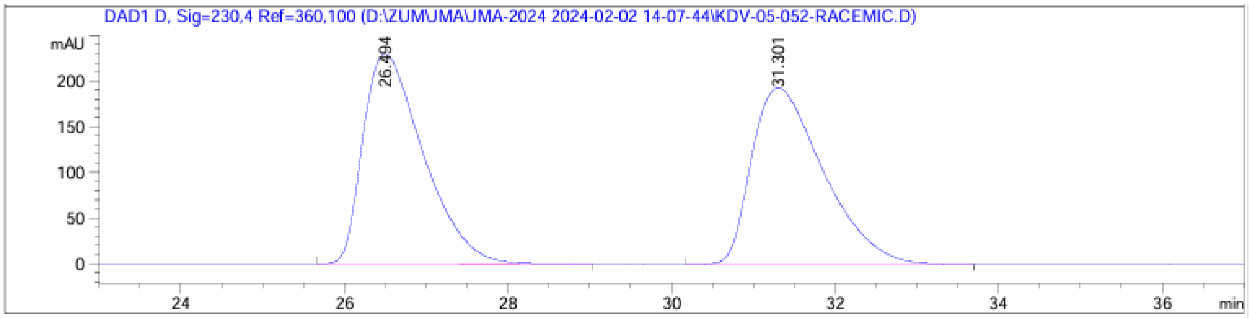

Peak #	RetTime [min]	Type	Width [min]	Area [mAU*s]	Height [mAU]	Area %
1	26.494	BB	0.7855	1.17688e4	229.80214	50.0914
2	31.301	BB	0.9365	1.17258e4	193.08974	49.9086

(*S*)-4-(Benzyloxy)-1-(4-methoxyphenyl)-3-methylbutan-2-one (5)

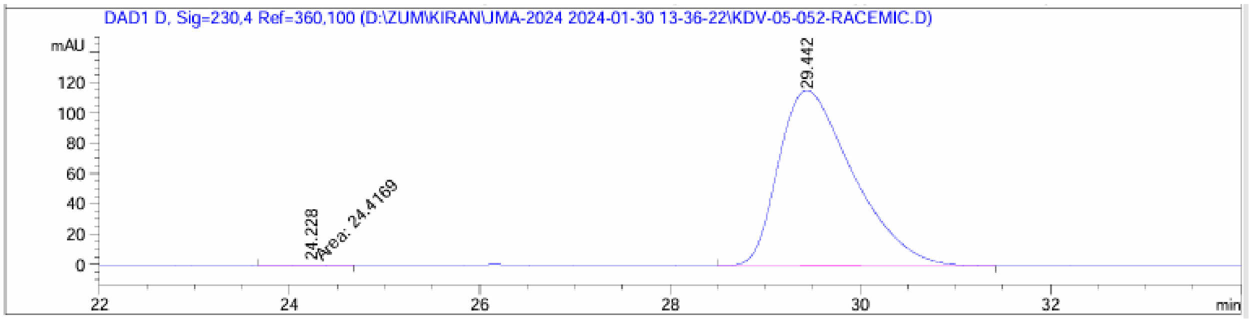

Peak #	RetTime [min]	Type	Width [min]	Area [mAU*s]	Height [mAU]	Area %
1	24.228	MM	0.7060	24.41686	5.76400e-1	0.3875
2	29.442	BB	0.8326	6276.17041	115.37809	99.6125

1-(3-(Benzyloxy)-2-methylpropyl)-4-methoxybenzene (*rac*-6)

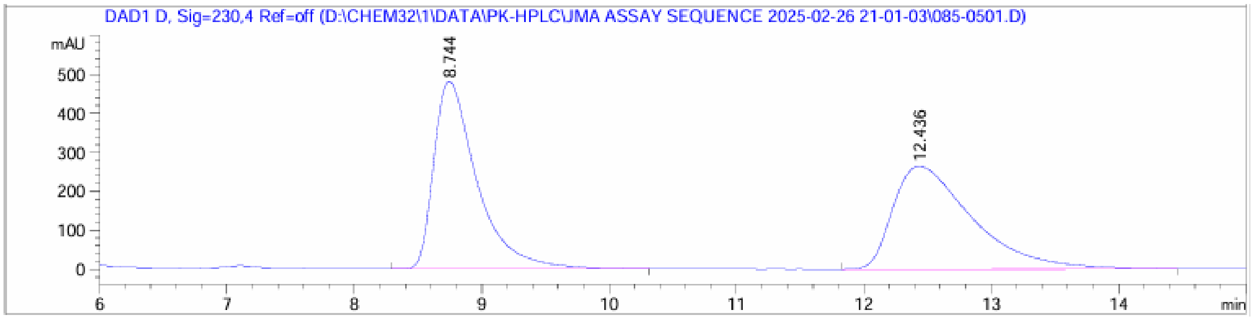

Peak #	RetTime [min]	Type	Width [min]	Area [mAU*s]	Height [mAU]	Area %
1	8.744	BB	0.3507	1.13499e4	481.98163	49.9475
2	12.436	BB	0.6375	1.13738e4	263.55243	50.0525

(*R*)-1-(3-(Benzyloxy)-2-methylpropyl)-4-methoxybenzene (6)

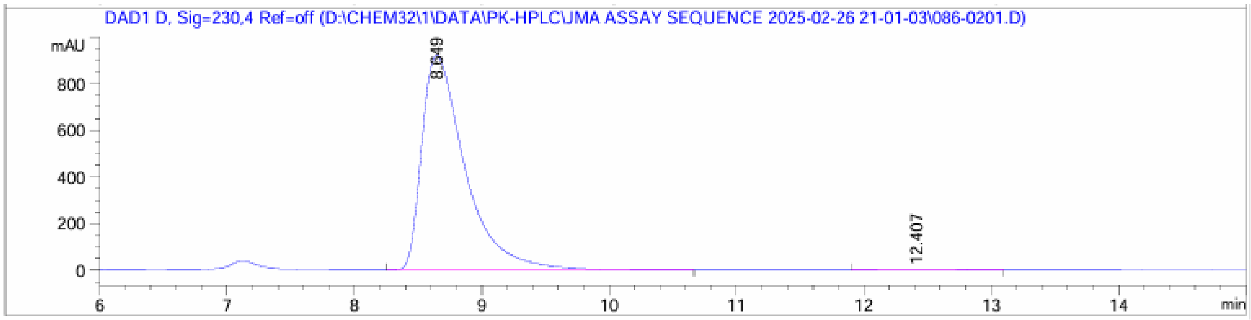

Peak #	RetTime [min]	Type	Width [min]	Area [mAU*s]	Height [mAU]	Area %
1	8.649	BB	0.3548	2.18259e4	920.06726	99.5850
2	12.407	BB	0.3923	90.95574	2.80481	0.4150

3-(Benzyloxy)-1-(4-methoxyphenyl)-2-methylpropan-1-ol (*rac*-7)

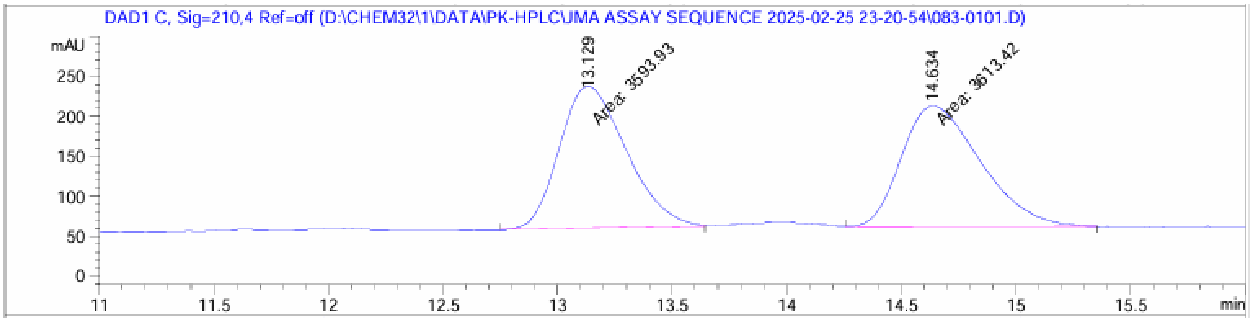

Peak #	RetTime [min]	Type	Width [min]	Area [mAU*s]	Height [mAU]	Area %
1	13.129	MM	0.3373	3593.93164	177.57408	49.8648
2	14.634	MM	0.3990	3613.42090	150.94986	50.1352

(1*R*,2*S*)-3-(Benzyloxy)-1-(4-methoxyphenyl)-2-methylpropan-1-ol (7)

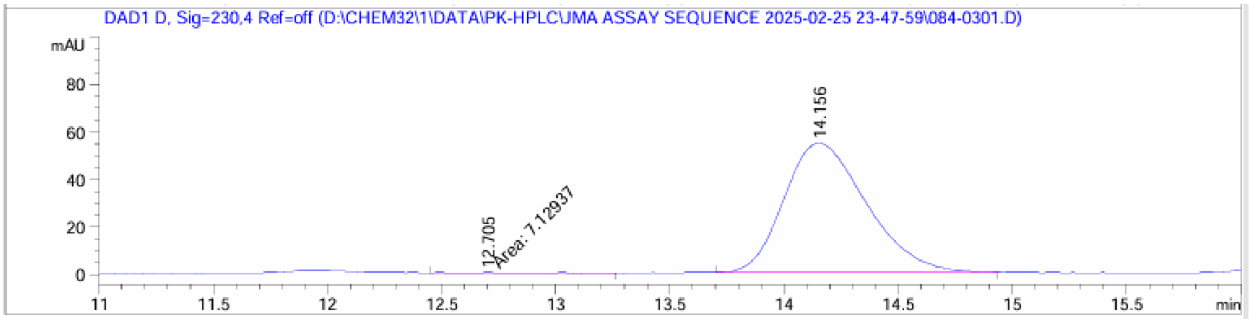

Peak #	RetTime [min]	Type	Width [min]	Area [mAU*s]	Height [mAU]	Area %
1	12.705	MM	0.3697	7.12937	3.21371e-1	0.5199
2	14.156	BV	0.3821	1364.21716	54.48143	99.4801

3-(Benzyloxy)-1-(4-isopropylphenyl)-2-methylpropan-1-one (*rac*-**3xe**)

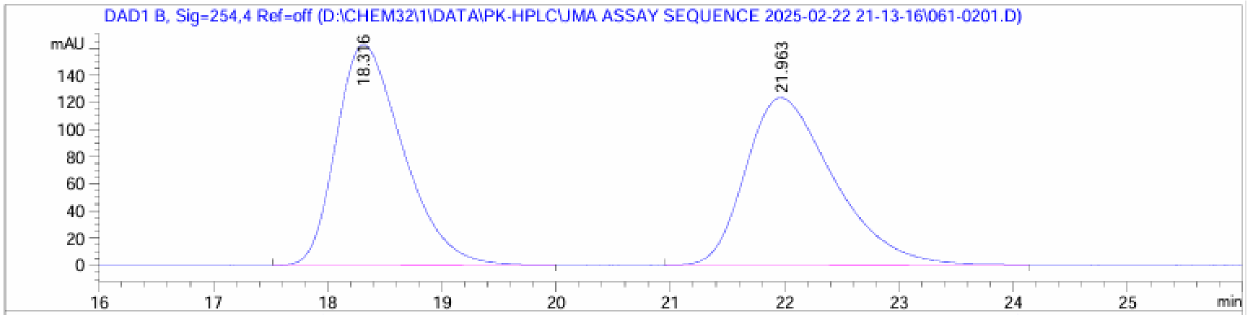

Peak #	RetTime [min]	Type	Width [min]	Area [mAU*s]	Height [mAU]	Area %
1	18.316	BB	0.6191	6569.13867	162.00275	50.0116
2	21.963	BB	0.7992	6566.08057	122.91641	49.9884

(*S*)-3-(Benzyloxy)-1-(4-isopropylphenyl)-2-methylpropan-1-one (3xe)

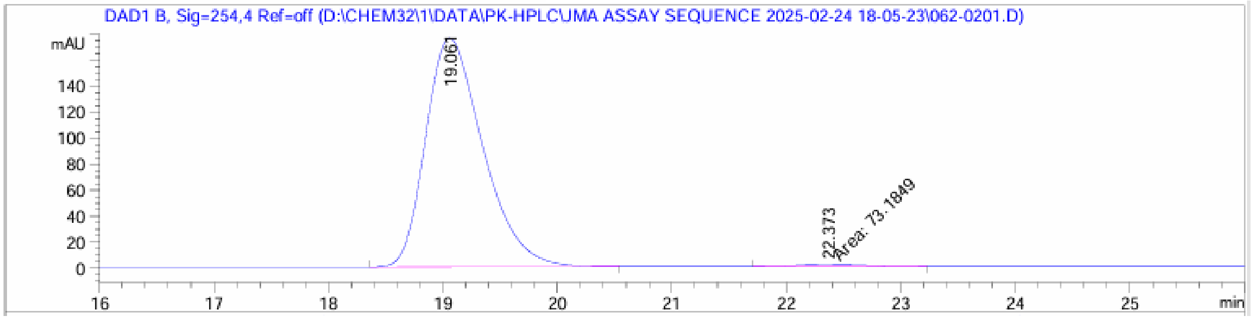

Peak #	RetTime [min]	Type	Width [min]	Area [mAU*s]	Height [mAU]	Area %
1	19.061	BB	0.5328	6115.22510	176.26251	98.8174
2	22.373	MM	0.6684	73.18493	1.82484	1.1826

3-(4-Isopropylphenyl)-2-methylpropan-1-ol (*rac*-**9**)

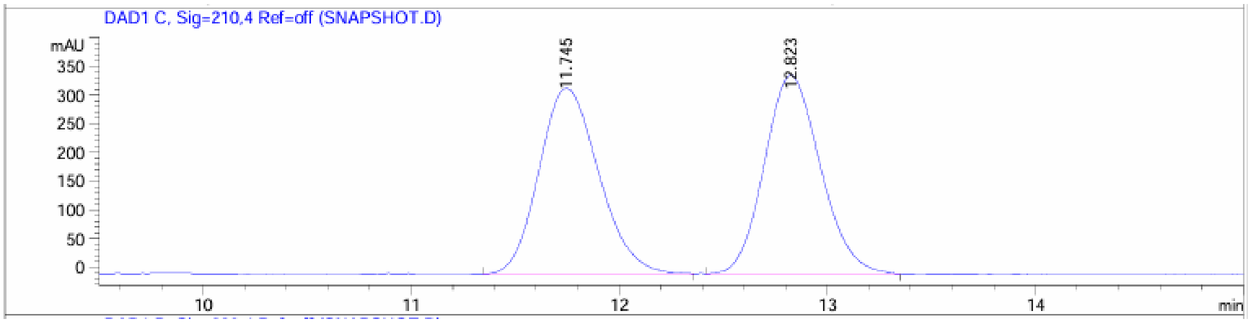

Peak #	RetTime [min]	Type	Width [min]	Area [mAU*s]	Height [mAU]	Area %
1	11.745	VV	0.3038	6475.58008	323.15781	49.9406
2	12.823	VV	0.2869	6490.97754	345.93454	50.0594

(*R*)-3-(4-Isopropylphenyl)-2-methylpropan-1-ol (9)

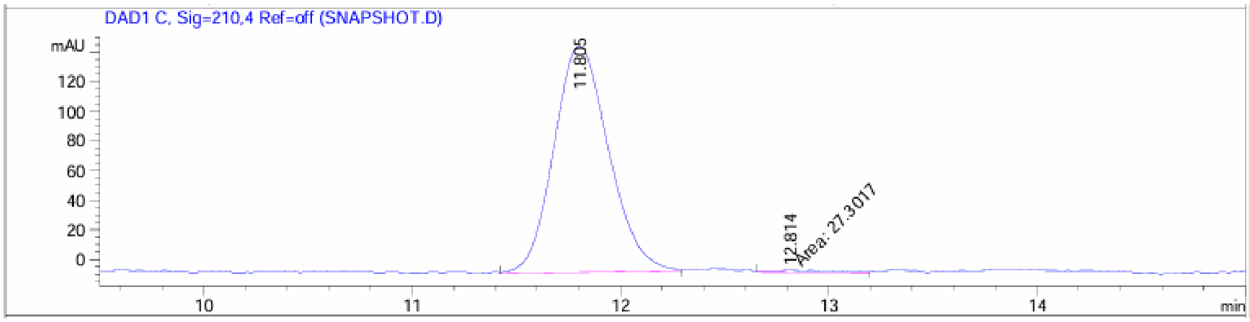

Peak #	RetTime [min]	Type	Width [min]	Area [mAU*s]	Height [mAU]	Area %
1	11.805	VV	0.2816	2792.13452	152.47707	99.0317
2	12.814	MM	0.2947	27.30174	1.54405	0.9683
